# Insights into gut microbiomes in stem cell transplantation by comprehensive shotgun long-read sequencing

**DOI:** 10.1038/s41598-024-53506-1

**Published:** 2024-02-19

**Authors:** Philipp Spohr, Sebastian Scharf, Anna Rommerskirchen, Birgit Henrich, Paul Jäger, Gunnar W. Klau, Rainer Haas, Alexander Dilthey, Klaus Pfeffer

**Affiliations:** 1 Chair Algorithmic Bioinformatics, Faculty of Mathematics and Natural Sciences, Heinrich Heine University Düsseldorf, Düsseldorf, Germany; 2 Institute of Medical Microbiology and Hospital Hygiene, Heinrich Heine University Düsseldorf, University Hospital Düsseldorf, Düsseldorf, Germany; 3 Department of Hematology, Immunology, and Clinical Immunology, Heinrich Heine University Düsseldorf, University Hospital Düsseldorf, Düsseldorf, Germany; 4 Center for Digital Medicine, Düsseldorf, Germany

**Keywords:** Microbiome stability, Whole-genome sequencing (WGS), Metagenomics, Bacteriome, Mycobiome, Archaeome, Eukaryome, Virome, Leukemia, Bioinformatics pipeline, Oxford Nanopore, Hematopoietic stem cell transplantation (alloHSCT), Clinical microbiology, Classification and taxonomy

## Abstract

The gut microbiome is a diverse ecosystem, dominated by bacteria; however, fungi, phages/viruses, archaea, and protozoa are also important members of the gut microbiota. Exploration of taxonomic compositions beyond bacteria as well as an understanding of the interaction between the bacteriome with the other members is limited using 16S rDNA sequencing. Here, we developed a pipeline enabling the simultaneous interrogation of the gut microbiome (bacteriome, mycobiome, archaeome, eukaryome, DNA virome) and of antibiotic resistance genes based on optimized long-read shotgun metagenomics protocols and custom bioinformatics. Using our pipeline we investigated the longitudinal composition of the gut microbiome in an exploratory clinical study in patients undergoing allogeneic hematopoietic stem cell transplantation (alloHSCT; n = 31). Pre-transplantation microbiomes exhibited a 3-cluster structure, characterized by *Bacteroides* spp. /*Phocaeicola* spp., mixed composition and *Enterococcus* abundances. We revealed substantial inter-individual and temporal variabilities of microbial domain compositions, human DNA, and antibiotic resistance genes during the course of alloHSCT. Interestingly, viruses and fungi accounted for substantial proportions of microbiome content in individual samples. In the course of HSCT, bacterial strains were stable or newly acquired. Our results demonstrate the disruptive potential of alloHSCTon the gut microbiome and pave the way for future comprehensive microbiome studies based on long-read metagenomics.

## Introduction

Allogeneic hematopoietic stem cell transplantation (alloHSCT) is a potentially curative treatment for patients with high-risk hematological malignancies encompassing acute myeloid leukemia, high-risk myelodysplastic syndromes (MDS), and lymphoid leukemia^1–4^. It is well-established that the gut bacteriome can be associated with specific outcomes of alloHSCT, including the occurrence of adverse events such as graft-versus-host-disease (GvHD) or life-threatening infections. Recently, adverse outcomes, including the risk of GvHD, have been linked to reduced bacterial diversity of the gut microbiome^5–8^, while the risk of bloodstream infections has been linked to the domination of individual taxa within the gut bacteriome^5,6,8,9^. A set of microbial taxa, including *Enterobacteriaceae*, *Clostridiales,* and *Blautia* have been implicated in alloHSCT success^6,10–13^. While a number of explanations for the observed associations have been put forward, including modulation of the immune system by microbiota-derived components and microbiome-host crosstalk at the level of metabolites, the observed associations—apart from an association of overall diversity with outcome, which has been replicated in international multi-center studies^14^—often remain inconsistent^5,15^ and incompletely understood.

The vast majority of microbiome studies were performed using ribosomal DNA (rDNA) based sequencing methods designed for bacterial and fungal 16/23S, 18/28S or ITS region rDNA sequences, respectively, thus neglecting the non-fungal eukaryome, and the DNA virome^16–23^. rDNA sequencing is a well-established, scalable, and cost-effective technology; it has, however, important limitations as it bears potential amplification-induced biases in bacteriome/mycobiome composition estimates and challenges in reliably assigning accurate species—or genus-level labels^24,25^. 16S/18S rDNA sequencing is thus ill-suited to characterize the potential contributions to alloHSCT outcomes of other domains of microbial life. For example, in the context of infection prevention, fungi and protozoan parasites like *Cryptosporidium* spp. and *Toxoplasma gondii* can become relevant in the clinical management of alloHSCT^26,27^; and *Candida* has been associated with GvHD^14^ and survival^28,29^. With regard to viruses, increases in persistent DNA viruses and reduced bacteriophage richness were observed to be associated with enteric GvHD^30^.

A challenge in characterizing associations between microbiome and alloHSCT outcomes consists in the highly dynamic nature of the gut microbiome over the course of alloHSCT^31^. The gut microbiome undergoes substantial temporal variation even in healthy control individuals^32^; In the context of alloHSCT, patient microbiomes have often been impacted by multiple cycles of cytostatic and antiinfective therapeutic treatment prior to the initiation of alloHSCT^33^, and continue to be biased by the effects of anti-bacterial, anti-fungal, and anti-viral prophylaxis, in addition to the effects of myeloablation and the subsequent establishment of a “new” immune system by the transplanted allograft^34^.

Longitudinal studies taking the aforementioned aspects into account are sparse, thus, we developed a novel workflow to enable whole-microbiome profiling of patient microbiomes, covering all domains of microbial life (with the exception of RNA viruses)^35^. Interrogation of non-bacterial domains of microbial life from shotgun metagenomics is challenging^36–38^; reasons for this include contamination^39,40^ and coverage gaps in the relevant reference databases, likely remaining despite significant recent expansion efforts^41,42^. We thus chose to implement the shotgun metagenomics step using long-read sequencing, based on the Oxford Nanopore technology, as the accuracy of taxonomic assignment generally increases with read length^43^, and assembled a comprehensive 292 Gb reference database (MetaGut database v 1.0) as the basis of the bioinformatics workflow. Furthermore, k-mer-based classification is known to be sensitive to mis-classification in the presence of out-of-database genomes^43,44^, which, the utilization of a large reference database notwithstanding, remains a relevant concern in the gut microbiome context. We thus propose to complement initial k-mer-based classification with a mapping-based verification approach to reduce the rate of false-positive taxonomic detections. Tailored bioinformatics increased the analytical accuracy and reduced the rate of false-positive taxonomic detections. Moreover, bioinformatic tools for the enumeration of antibiotic resistance genes were implemented. Based on this, we applied the pipeline to characterize microbiome dynamics over the course of alloHSCT in an explorative clinical study including patients (n = 31) before alloHSCT, during the phase of leukopenia, and hematological reconstitution. In addition, a comprehensive characterization of gut microbiomes was performed for a group of healthy volunteers (n = 11) and compared to patient microbiomes pre-Tx. Our pipeline proved to be a valuable tool for the comprehensive characterization of microbiomes. Furthermore, a 3-cluster structure of pre-Tx patient microbiomes was detected and the acquisition and replacement of bacterial strains during the course of alloHCST could be successfully monitored.

## Results

### A pipeline for the accurate and comprehensive characterization of gut microbiomes

**Figure 1:**
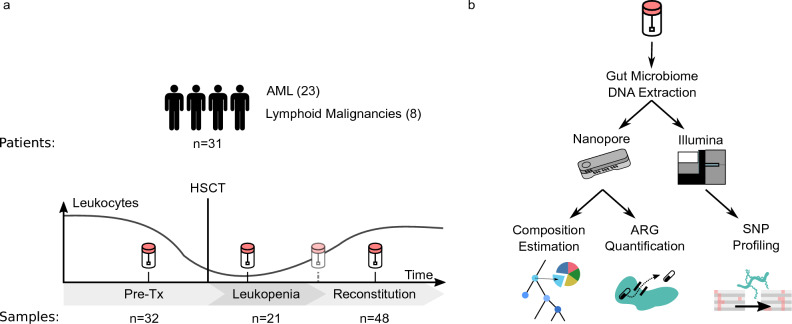
Overview of the exploratory clinical study (**a**) and the workflow (**b**). (**a**) 31 patients undergoing alloHSCT were recruited for an exploratory study and stool samples were collected longitudinally before transplantation (pre-Tx), during leukopenia (defined as white blood count of ≤ 1,000/µL), and during reconstitution for each patient. (**b**) The workflow comprises optimized protocols for sample preparation and DNA extraction, long-read metagenomics using the Oxford Nanopore technology, and custom bioinformatics for taxon validation, quantification of antibiotic resistance genes/crAssphage sequences, and tracking of strain dynamics based on short-read Illumina data.

To enable the characterization of the gut microbiome encompassing the bacteriome, mycobiome, archaeome, DNA virome (bacteriophages/viruses), and protozoa of hematological patients from stool samples at high-resolution, we developed an integrated method for robust microbiome characterization (Fig. 1). It comprises the following components: (i) protocols for stool sample processing and DNA extraction, based on a modified version of the Human Microbiome Project (HMP) protocol^45,46^ for robust sample handling and DNA extraction and suitable for processing samples at different degrees of stool consistency (Methods); (ii) long-read sequencing and compositional analysis of microbiota, based on the Oxford Nanopore platform and a custom bioinformatics approach integrating Kraken2-based read assignments^47^ with a newly developed mapping-based validation to ensure that sufficient-quality pairwise alignments exist between reads and the taxonomic entities they are assigned to; taxa with low rates of mapping-based read validation are flagged and not included in downstream analyses and visualizations (see Methods for details); (iii) targeted antibiotic resistance gene (ARG); and (iv) crAssphage analyses, based on read mapping to specific databases comprising (a) ARGs and (b) recently assembled crAssphage strain sequences^48^; (v) longitudinal tracking of strain dynamics: short-read Illumina sequencing data are used to detect single-nucleotide variants (SNVs) in bacterial reference genomes, and the temporal dynamics of the detected SNVs over multiple samples from the same individual are investigated to infer the maintenance, acquisition and loss of bacterial strains. Extraction of sufficient amounts of DNA for metagenomic sequencing was challenging during the period immediately following alloHSCT, when patient stool samples are known to vary in consistency, rendering the exhibit very low biomass. We thus incorporated a modified version of the HMP gut microbiome protocol^45,46^, which proved to work well in our study across the course of alloHSCT. We initially carried out three experiments as a validation for the protocol and bioinformatic pipeline:

First, we applied the workflow to a well-defined microbial community standard (Zymo Gut Microbiome Standard) and observed good concordance at the genus level between the theoretical and the inferred composition and lesser concordance at the species level (Pearson’s r = 0.82/0.67, for comparison a minimap2-based abundance estimation leads to r = 0.84 for both levels; Supplementary Fig. 1). However, an elevated proportion of false-positive species hits, accounting for 26.02% of total abundance were driven by reads from *Veillonella rogosae* and *Prevotella corporis* (which do not contain genomes in the database) being misassigned to different species of their respective genera (Supplementary Table 1).

Second, we applied the workflow to a cohort of 10 healthy volunteers. Here, we observed good agreement of high-level compositional metrics with the WGS-based component of LifeLines-DEEP^49^ (Supplementary Fig. 2). Of note, at a median frequency of 25.47% (range 9.10%–51.72%), the inferred abundance of *Bacteroides spp.* was higher in the investigated cohort of healthy volunteers compared to the LifeLines-DEEP cohort with a median frequency of 14.99% (range 0.80%–48.94%).

Third, we assessed the agreement between Nanopore and Illumina based sequencing. We used Kraken2 with our default database on short-reads we had for a subset of samples. For each sample that generated at least 10.000 reads with both platforms we resampled to a fixed amount of 10.000 reads and then compared the resulting compositions (Supplementary Fig. 12). We observed an overall agreement (Pearson’s r of 0.88) between the composition vectors.

### Reliable characterization of gut microbiomes over the course of alloHSCT

To characterize the microbiomes of alloHSCT patients and to investigate the dynamic changes of the gut microbiome over the course of alloHSCT, we recruited a cohort of 31 patients, diagnosed with defined hematological malignancies (see Supplementary Note 1 and Methods for a description of the cohort and recruitment process, and Supplementary Table 2 for patient characteristics), undergoing alloHSCT at Düsseldorf University Hospital.

**Figure 2:**
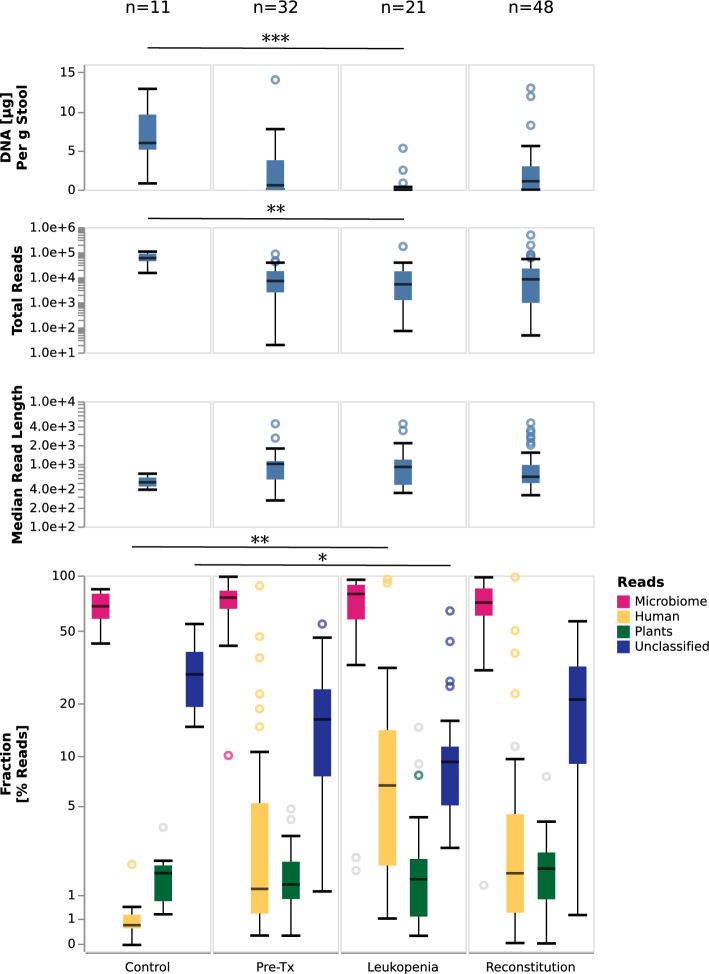
DNA amounts, sequencing metadata, and high-level stool sample compositions. Shown are the amounts of extracted DNA per gram of stool; the number of generated Nanopore sequencing reads; median read lengths; and the relative proportions of microbial-, human-, and plant-assigned reads, as well as the proportions of reads remaining unclassified by the initial Kraken2-based step. Gray circles in the bottom panel indicate outliers for which less than 80% of the reads are in taxa validated by the mapping-based validation step. For each panel, the most significant difference between included categories is indicated (Mann–Whitney U test; * [*p* < 1.19 × 10^−3^], ** [*p* < 2.38 × 10^−4^] or *** [*p* < 2.38 × 10^−5^]). Raw data is shown in Supplementary Table 3 and Supplementary Table 4.

Based on 101 stool samples collected longitudinally at defined time points over the course of alloHSCT (pre-Tx, leukopenia, reconstitution, see Fig. 1), we assessed the ability of the workflow to enable microbiome characterization at different stages of the treatment cycle (Fig. 2). First, we observed that DNA yields in the hematological cohort differed significantly from the healthy cohort (patients 0.47 µg/g stool, healthy 5.95 µg/g stool; *p* = 0.000011) and also displayed significant variation between treatment time points (Kruskall–Wallis *p*-value: 0.0031 for the 3 treatment phases). DNA yields were typically lowest during leukopenia (median = 0.06 µg/g stool), compared to the pre-Tx and reconstitution periods (medians = 0.6 µg/g stool and 1.1 µg/g stool, respectively). Second, the utilized protocols enabled the generation of more than 100,000 reads per sample (89 samples with >  = 100,000 reads). We observed that median read counts were lowest for samples taken during leukopenia (median = 128,330). Third, the median of the median sample read lengths was 653 base pairs (bp) with the longest read spanning 888,591 bp. DNA extraction and sequencing data statistics are summarized in Supplementary Table 3. Fourth, at median per-species validation rates of 77.79% (bacteria) and 50.00% (viruses) across treatment periods and in the healthy cohort showed generally high rates of mapping-based validation (Supplementary Fig. 3). Fungi exhibited lower validation rates (median = 5.33%); we observed pronounced differences in fungal validation rates between the healthy and alloHSCT samples, and also between the characterized time points, with the healthy samples generally exhibiting the lowest fungal validation rates (Supplementary Fig. 3).

Human-derived DNA is often not reported in microbiome studies; we however noted that the amount of human DNA differed significantly between the control group and the patient cohort, and also between the sampling time points within the hematological cohort. The relative proportion of human DNA was highest during the leukopenic period, possibly reflecting the effect of myeloablative therapy on the gut mucosa (Fig. 2). However, the increase in human DNA proportions might, at least in parts, reflect the decrease of microbiota after myeloablation and anti-infective therapy. We also observed that the per-read validation rates of the human reads were lowest for the control samples, i.e. indicating that a substantial proportion of the reads assigned by Kraken2 to the “Homo sapiens” species in these samples may reflect false-positive assignments (Supplementary Fig. 3).

Per-species validation rates for archaea and non-fungal eukaryotes were close to zero in most control and alloHSCT samples and also across the characterized time points. Analyses of the occurrence and abundance of individual species were thus limited to the bacterial, viral and fungal components of the microbiome. Of note, the presence of plant-based DNA could also sometimes be validated, in particular during the leukopenic period, the most frequently validated plant DNA was *Cucumis sativus* (Cucumber) and different *Triticum* spp. (wheat), *Pisum sativum* (pea) and *Musa* spp. (banana) (Supplementary Fig. 3).

### alloHSCT microbiomes exhibited dynamic and diverse compositions

**Figure 3:**
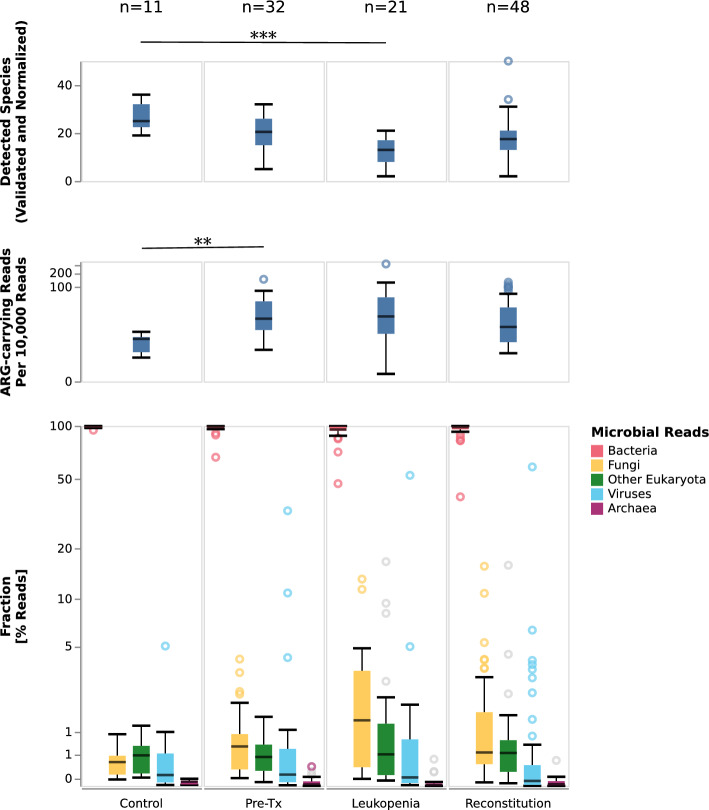
Diversity, ARG-carrying reads, and microbiome composition in stool samples from controls and alloHSCT patients at indicated phases. Shown are the numbers of detected genera, normalized to the sample with the lowest read count; the rate of reads that carry an ARG element; and the relative proportions of bacterial-, fungal-, archaeal-assigned reads as well as the proportion of reads assigned to DNA viruses (incl. bacteriophages), and non-fungal eukaryotes. Gray circles in the bottom panel indicate outliers for which less than 80% of the reads are in taxa validated by the mapping-based validation step. For each panel, the most significant difference between included categories is indicated (Mann–Whitney U test; * [*p* < 1.19 × 10^−3^], ** [*p* < 2.38 × 10^−4^] or *** [*p* < 2.38 × 10^−5^]). Raw data is shown in Supplementary Table 3 and Supplementary Table 4.

Having established and validated our workflow as an informative method, we investigated high-level gut microbiome compositions (Fig. 3). DNA yield was lower in the patient cohort overall and specifically during leukopenia, possibly reflecting the impact of conditioning and antiinfective therapy as well as the stool consistency. The number of normalized species was highest in the samples from the control group and lowest for samples during leukopenia (Fig. 3, top panel). Besides, ARG-reads significantly differed between control samples (low) and alloHSCT samples (high) (Fig. 3, middle panel). The frequency of ARG reads of the control samples was significantly lower compared to alloHSCT samples (*p* = 0.0006281). Bacteria accounted for > 90% of microbial reads in 89/101 hematological samples and in 11/11 of control samples; bacteria thus dominated the large majority of the investigated samples. At median relative abundances of 0.53%, 0.11% and 0.02%, fungi, viruses, and archaea accounted for low, but non-negligible proportions of the characterized alloHSCT and control microbiomes; median abundances of these groups were generally comparable between control and alloHSCT samples and between alloHSCT time points, with the exception of fungi, which showed an increased abundance in the alloHSCT samples in general and during leukopenia in particular (median = 1.32% vs 0.36% for control) (Fig. 3, bottom panel). Non-fungal eukaryotes were also estimated to account for small fractions of the characterized microbiomes (median = 0.49%).

The dominance of bacteria in most samples notwithstanding, individual microbiome samples were found to exhibit increased frequencies of non-bacterial taxa. For example, in two alloHSCT samples from one patient, the abundance of viral DNA was found to be ≥ 50%, accounted for by species belonging to the genus crAssphage; in two additional samples from different patients, it was ≥ 10% (Supplementary Table 14). Interestingly, in one control sample, viral abundance approached 5% (Fig. 3, bottom panel). Similarly, in five samples, fungal DNA was found at abundances ≥ 5%, accounted for mostly by the species *Saccharomyces cerevisiae*, *Candida albicans*, and *[Candida] glabrata* (Supplementary Table 6). In all of these instances, taxon presence was validated with read-mapping based verification.

### Pre-Tx alloHSCT microbiomes could be grouped into 3 distinct clusters

**Figure 4:**
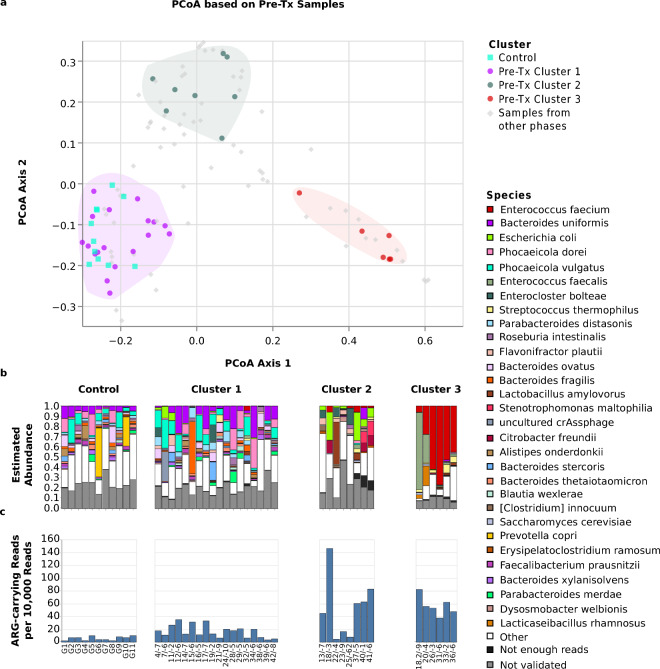
Microbiome structures and compositions of pre-Tx alloHSCT samples and healthy controls. The Figure shows (**a**) the positions of pre-Tx and control samples, displayed as colored dots, in the joint PCoA space of all samples, as well as the positions of pre-Tx Cluster 1, Cluster 2, and Cluster 3 in PCoA space (shaded areas), (**b**) bar plots visualizing the microbiome compositions of healthy control and pre-Tx alloHSCT samples, stratified by pre-Tx cluster membership, and (**c**) the rate of reads that carry an ARG element, separately for control and pre-Tx alloHSCT samples and stratified by pre-Tx cluster membership. The bar plots show the 30 species that attained the highest aggregated sum of frequency across all time points, and, of these, within each sample, only the species that 1.) were assigned at least 5 reads to and 2.) passed the mapping-based taxon validation step were depicted. The combined abundances of species with fewer than 5 reads is shown in the category “Not enough reads”; the category “Not validated” shows the combined abundances of species with more than 5 Kraken2-assigned reads that did not pass the mapping-based taxon validation step. Sample labels below the bar plots specify patient or control sample ID, followed, for alloHSCT samples, by the day of sampling relative to the stem cell transplantation time point.

To investigate the structure of the pre-transplantation gut microbiome in alloHSCT patients, we carried out a PCoA analysis and found that pre-Tx alloHSCT and control samples fell into 3 distinct clusters (Fig. 4).

Cluster 1, comprising 18 pre-Tx alloHSCT samples from distinct patients and all 11 control samples, was characterized by high abundances of species belonging to the genera of *Bacteroides* and *Phocaeicola,* accompanied by relatively high normalized numbers of detected species per sample; specifically, *Bacteroides uniformis, Phocaeicola vulgatus and Phocaeicola dorei* accounted for ≥ 25% of reads in Cluster 1 samples, while the median number of detected normalized and validated species per sample was 21.5, depicting a relatively high diversity. Cluster 3, comprising 6 pre-Tx alloHSCT samples, was characterized by *Enterococcus* spp. domination and exhibited the lowest diversity in terms of the number of detected species per sample; in 5/6 Cluster 3 samples, *Enterococcus faecium* accounted for ≥ 25% of sequencing reads, at a median number of 7.5 detected normalized and validated species per sample. Cluster 2, comprising 8 pre-Tx alloHSCT samples, was the most diverse in terms of the number of detected normalized species per sample (median = 26) and was also characterized by more uniform species abundance distributions. In Cluster 2, a median of 7.5 species were required to account for ≥ 50% of sequencing reads, compared to medians of 5.5 and 1 species in Clusters 1 and 3, respectively. Notably, Cluster 2 species contained in individual samples included *Citrobacter freundii* (max. abundance 12.6%)*, Lactobacillus amylovorus* (max. abundance 41.4%), and *Escherichia coli* (max. abundance 27.5%)*;* by contrast, the genera characteristic for Cluster 1 and 3 *(Bacteroides/Phocaeicola* and *Enterococcus*) were present at only low abundances in Cluster 2 (abundances ≤ 10% for 7/8 samples of Cluster 2) (Supplementary Table 15).

Suggestive structural differences between the 3 pre-Tx clusters were also apparent at the level of the fungal and viral microbiome components (Fig. 7). *Saccharomyces cerevisiae* (validated presence in 19 samples and > 10% relative within-fungal abundance in 15 samples) and *Candida albicans* (validated presence in 3 samples and > 10% within-fungal relative abundance in 3 samples) were the most abundant fungal species detected during the pre-Tx period; the presence of *Candida albicans*, however, was limited to Cluster 2 and 3 samples. At the level of the DNA virome, *Skunavirus* dominated (> 50% relative abundance) 8 of 14 Cluster 2 and Cluster 3 samples, but only 2 of 18 Cluster 1 samples. Conversely, crAssphage was found mostly in Cluster 1 samples (6 validated detections in total, 5 of which occurred in Cluster 1 samples); and 2 Cluster 1 samples were crAssphage-dominated, but no samples from Clusters 2 or 3.

### Microbiomes in leukopenia exhibited decreased diversity and increased abundances of fungi and various bacterial genera

We proceeded to investigate the structure of microbiome samples collected during the leukopenic period, reflecting the combined effects of myeloablation, anti-infective prophylaxis, and recent alloHSCT. Consistent with an assumed bottleneck effect of anti-infective prophylaxis on the population of gut microbes, the leukopenic period exhibited the lowest median number of normalized detected and validated microbial species per sample (13; compared to 20.5 and 17.5 for the pre-Tx and reconstitution periods, across all clusters, respectively; Fig. 3). The proportion of the microbiome accounted for by fungal taxa (mycobiome), however, was substantially increased during leukopenia (median per sample: 1.32% of reads, compared to 0.65 and 0.54 for pre-Tx and reconstitution, respectively; Fig. 2); the main detected fungal genera were *Candida albicans* (2.06% mean absolute abundance across leukopenia samples) and *Saccharomyces cerevisiae* (1.97% mean absolute abundance across leukopenia samples).

**Figure 5:**
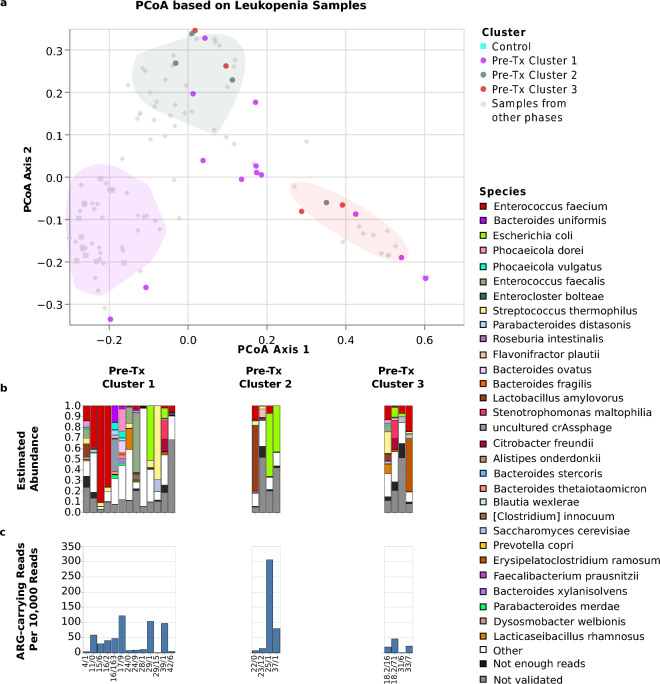
Microbiome structures and compositions during leukopenia. The Figure shows, for alloHSCT samples collected during the leukopenic period, (**a**) sample positions in the joint PCoA space of all samples, (**b**) bar plots visualizing sample microbiome compositions, and (**c**) rates of reads carrying an ARG element. All visualizations are stratified by pre-Tx cluster membership of the corresponding patients; in the top panel, pre-Tx cluster membership is indicated by dot color. The bar plots show the 30 species that attained the highest mean frequency across all time points, and, of these, within each sample, only the species that 1.) were assigned at least 5 reads and 2.) passed the mapping-based taxon validation step were depicted. The combined abundance of species with fewer than 5 reads is shown in the category “Not enough reads”; the category “Not validated” shows the combined abundance of species with more than 5 Kraken2-assigned reads that did not pass the mapping-based validation step. Sample labels below the bar plots specify patient ID, followed by the day of sampling relative to the stem cell transplantation time point.

Changes in microbiome composition compared to the pre-Tx period were also apparent at the level of individual bacterial taxa; *Bacteroides uniformis*, *Enterococcus faecium*, *Phocaeicola vulgatus*, and *Escherichia coli* were detected at abundances ≥ 5% in 4.76%, 47.62%, 9.52%, and 28.58% of samples during leukopenia, respectively, compared to 40.63%, 28.13%, 34.38%, and 18.75% for pre-Tx samples (Supplementary Table 5 and Figs. 4 and 5). Of note, the observed overall expansion of *Enterococcus faecium* during leukopenia was driven by samples from patients assigned to pre-Tx Cluster 1; patients assigned to the *Enterococcus*-associated pre-Tx Cluster 3, by contrast, generally showed a reduction in mean *Enterococcus faecium* abundance (44.99% pre-Tx, 11.30% leukopenia). Interestingly, changes at the level of individual species similar to bacteria were also detected for specific fungi: *Saccharomyces cerevisiae* was detected at an abundance of ≥ 5% in 53.13% of pre-Tx samples and 71.43% of leukopenia samples showing a slight increase. A decrease can be observed for uncultured crAssphage (18.75% of pre-Tx samples vs. 9.52% of leukopenia samples) (Supplementary Table 6, Supplementary Table 7, Supplementary Table 15).

### The reconstitution microbiomes were characterized by increased similarity to the pre-Tx microbiomes

**Figure 6:**
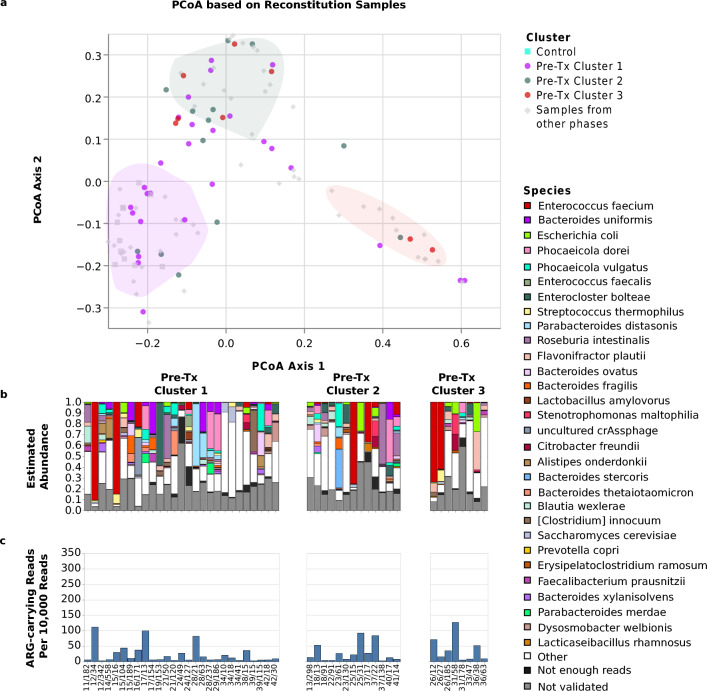
Microbiome structures and composition during reconstitution. The figure shows, for alloHSCT samples collected during the reconstitution period, (**a**) sample positions in the joint PCoA space of all samples, (**b**) bar plots visualizing sample microbiome compositions, and (**c**) rates of reads carrying an ARG element. All visualizations are stratified by pre-Tx cluster membership of the corresponding patients; in the top panel, pre-Tx cluster membership is indicated by dot color. The bar plots show the 30 species that attained the highest mean frequency across all time points, and, of these, within each sample, only the species that 1.) were assigned at least 5 reads and 2.) passed the mapping-based taxon validation step were depicted. The combined abundance of species with fewer than 5 reads is shown in the category “Not enough reads”; the category “Not validated” shows the combined abundance of species with more than 5 Kraken2-assigned reads that did not pass the mapping-based validation step. Sample labels below the bar plots specify patient ID, followed by the day of sampling relative to the stem cell transplantation time point.

**Figure 7:**
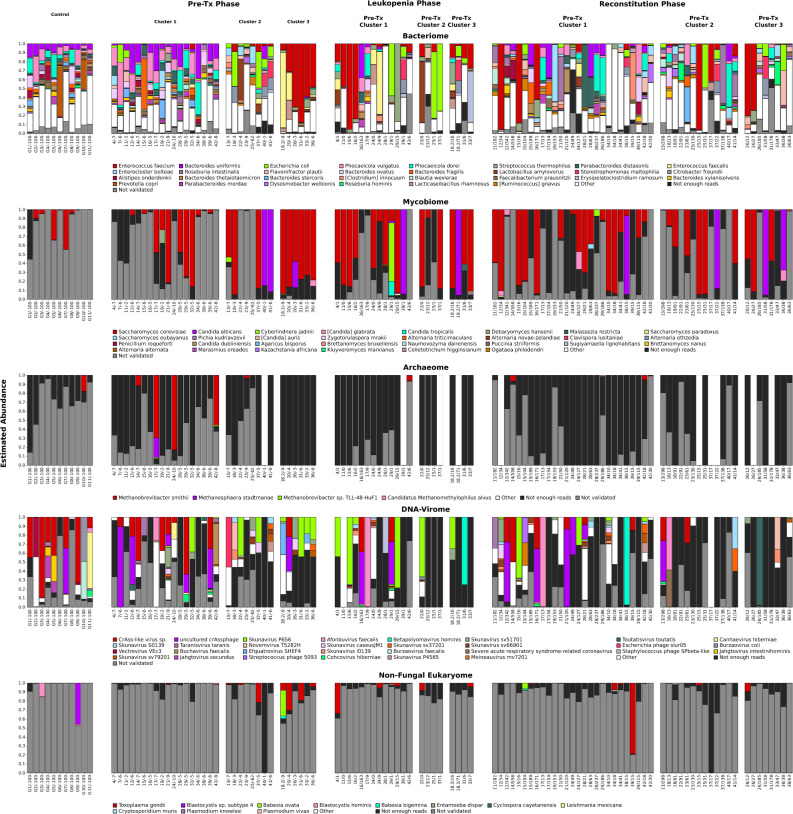
Integrated microbiome analysis of all characterized samples. Shown are, for each sample, bar plots that visualize the respective compositions of the bacterial, fungal, archaeal, DNA-viral, and non-fungal eukaryotic components of the sample microbiome. Normalization was applied independently to each bar plot. The bar plots only show the 30 (for bacteria, fungi and DNA viruses), 4 (for archaea), and 11 (for non-fungal eukaryotes) species that attained the highest mean relative frequency within the considered taxonomic category across all timepoints, and, of these, within each sample, only the species that (**a**) were assigned at least 5 reads by Kraken2, and (**b**) passed the mapping-based taxon validation step. The combined abundance of species within each taxonomic category with fewer than 5 reads is shown in the category “not enough reads”; the category “not validated” shows the combined abundance of species with more than 5 reads that did not pass the mapping-based validation step. Sample labels below the bar plots specify healthy control ID or the patient ID, followed by the day of sampling relative to the transplantation event.

Compared to samples collected during leukopenia, samples during the reconstitution period collectively showed similarity to pre-Tx microbiome samples. During the leukopenic period, approximately 52% (11/21) of collected samples were found to be within one of the 3 cluster spaces defined based on pre-Tx samples; during reconstitution, this fraction increased to 83% (40/48; Fig. 6). In addition, reconstitution microbiomes also resembled pre-Tx microbiomes at the level of observed overall abundances of bacteria, fungi, and DNA viruses (Fig. 3); at the level of bacterial species detected at ≥ 5% abundance (Supplementary Table 5); and at the level of the fungal and DNA viral species that exhibited the highest rates of validated detection at ≥ 5% relative abundance, i.e. *Saccharomyces cerevisiae* and *Candida albicans* for fungi, as well as as *Skunavirus* and crAssphages for DNA viruses (Supplementary Table 6, Supplementary Table 7, Fig. 7).

Bacterial species exhibiting notable differences compared to the pre-Tx period included *Roseburia intestinalis*, which was detected in approximately twice as many samples at abundances ≥ 5% during reconstitution than during pre-Tx (13% compared to 6%), and which accounted for ≥ 50% of overall microbial abundance in individual reconstitution samples; *Parabacteroides distasonis*, which exhibited dominance (≥ 50% abundance) in one reconstitution sample (Supplementary Table 14), but overall decreased detection at ≥ 5% abundance (10% during reconstitution samples compared to 25% for pre-Tx samples); and *Phocaeicola vulgatus* and *Bacteroides uniformis*, which also exhibited decreased detection rates at the ≥ 5% abundance threshold (15% and 17% for reconstitution samples compared to 34% and 41% for pre-Tx samples, respectively; Supplementary Table 5). While similar differences were not detected for fungal species Supplementary Table 6), a few viral species exhibited exclusive detection at > 5% validated relative abundance in reconstitution samples compared to pre-Tx samples (Supplementary Table 7); while these detections were typically limited to individual samples, in some instances the underlying viral species dominated the viral microbiome component (observed e.g., in the case of *Betapolyomavirus hominis* and *Afonbuvirus faecalis*; Fig. 7).

### Pre-Tx clusters were not associated with longitudinal microbiome trajectories or clinical outcomes

Pre-Tx cluster assignment did not predict the cluster membership of samples from the same patient over the course of alloHSCT, neither during the leukopenic nor during the reconstitution period (Figs. 5, 6); that is, pre-Tx cluster membership was not associated with the microbiome trajectory at the level of individual patients. Similarly, the pre-Tx detection of *Candida albicans* or uncultured crAssphage was not associated with the detection of these genera during leukocytopenia or reconstitution (Fig. 7, Supplementary Fig. 7).

Furthermore, while the proportion of patients with adverse outcomes (relapse and death) or GvHD varied between the 3 clusters (44%, 25% and 67% for adverse outcomes in Cluster 1, 2 and 3, respectively; 11%, 12%, and 17% for GvHD), these differences were not statistically significant (*p* = 0.297 for serious outcomes; *p* = 0.938 for GvHD; Pearsons’ chi-squared test). Further, we did not observe an association between cluster membership and the type of hematological disease patients were treated for (Supplementary Table 8).

### Analyses of antibiotic resistance, viral strain diversity and the tracking of individual marker taxa

We further investigated whether the whole-genome microbiome sequencing component could enable inferences about the presence of antibiotic resistance genes (ARGs), viral strain diversity, and the abundances of individual bacterial, fungal, or DNA-viral marker taxa which were described in previous publications (Supplementary Table 9. Literature List Marker taxa) or which were of particular interest. First, we investigated the detection of ARGs by quantifying the proportion of sequencing reads that aligned against known antibiotic resistance genes; the observed rates of ARG-carrying reads (Fig. 4) were relatively similar for pre-Tx and leukopenia alloHSCT samples (median = 20.52 ARG-carrying reads per 10.000 reads for pre-Tx samples; 23.00 for leukopenia samples), but substantially lower in the reconstitution (median = 13.39) and control (median = 7.01) samples. Of note, we observed lower rates of ARG-carrying reads in pre-Tx Cluster 1 samples than in Cluster 2 and Cluster 3 samples (Fig. 4; *p* = 0.00102); consistent with the observation that pre-Tx Clusters 2 and 3 contained microbiomes which were more divergent from the characterized control samples, possibly due to prior exposure to anti-infective medications. Next, prompted by the observation that crAssphages accounted for substantial proportions of total microbiome content in individual samples and by the availability of a large number of resolved crAssphage strain genomes from a recent publication^48^, we investigated the detectability and relative abundances of different crAssphage strains using a mapping-based approach. This analysis showed that (i) mapping against a database containing only crAssphage genomes resulted in substantially higher estimated crAssphage abundances in many samples than classifying against the comprehensive MetaGut database (Supplementary Fig. 4), (ii) read mapping enabled sensitive detection and differentiation between different crAssphage strains (Supplementary Fig. 5), and (iii) many reads classified as crAssphage in the mapping-based analysis were assigned to other taxa by the Kraken2-based read assignment process (Supplementary Fig. 6). Of note, while these results confirmed the applicability of the generated long-read data to crAssphage-focused analyses, they also suggested that substantial crAssphage strain diversity is currently not represented in the comprehensive MetaGut database. Third, we investigated the longitudinal changes of the abundances of predefined marker taxa. To this end, we assembled a list of taxa reported to be associated with alloHSCT outcomes from the literature (Methods); and, to extend this analysis we added selected fungal and viral taxa accounting for substantial proportions of microbiome content (Methods, Supplementary Table 9). We then carried out a longitudinal analysis of the presence and abundance of the selected marker taxa over time (Supplementary Fig. 7), and found that the generated abundance estimates were informative for changes over time, based on largely non-overlapping abundance confidence intervals between time points, demonstrating, for example, fluctuations in the abundances of *Akkermansia muciniphilia* and *Blautia*.

### Investigation of bacterial strain dynamics showed replacement of strains over the course of alloHSCT in some individuals

**Figure 8:**
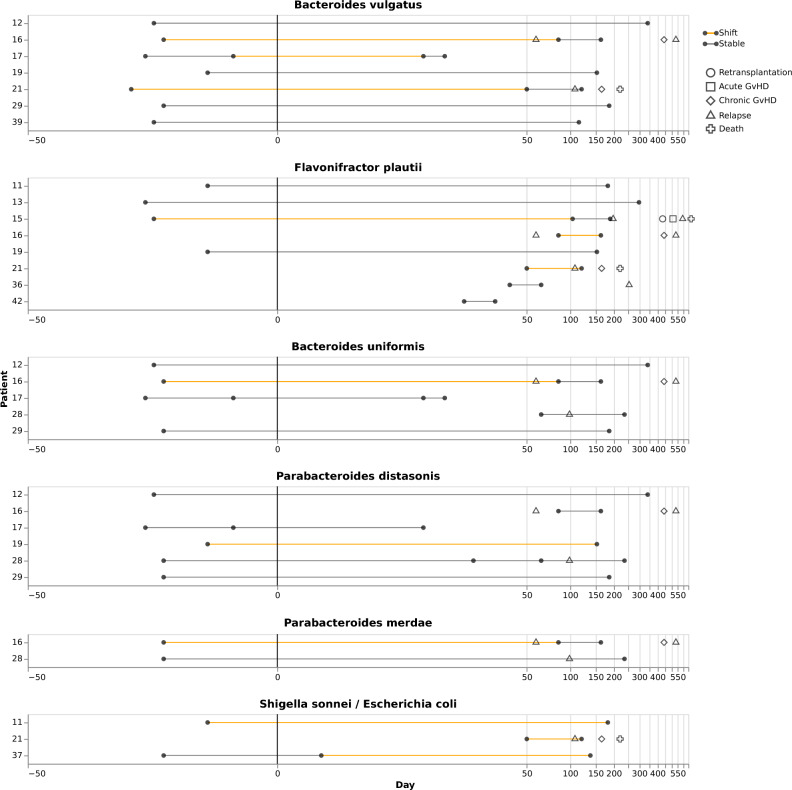
Strain replacement analysis. The Figure visualizes strain dynamics for combinations of bacterial species and sampling timepoints for which sufficient Illumina short-read data was available. Each species is represented by a main panel; main panels are labeled with patient IDs on the y-axis and sampling times on the x-axis. Each dot represents one sampling event, and each horizontal line represents the period between two sampling events. Horizontal lines are colored according to whether a strain replacement event was determined to have taken place between the two sampling events (orange) enclosing the line. Additional symbols indicate adverse events.

Finally, we investigated whether the significant impact of myeloablation, antiinfective therapy, and alloHSCT on the gut microbiome was associated with bacterial strain replacement, potentially indicating re-colonization of ecological niches in the microbiome. We developed a method to approximate species-level core genome average identities across samples based on short-read sequencing data, generated Illumina data for a subset of our samples with sufficient DNA yield (Methods), and applied the developed method for the detection of strain replacement events. Based on the generated data, we could characterize longitudinal strain dynamics in 31 cases, representing 6 bacterial species and 14 patients (Fig. 8). Of 44 considered intervals, 12 were classified as representing a strain-switching event; of these, 8 spanned an alloHSCT and 2 a relapse event; compared to 19 of the 29 alloHSCT or relapse intervals not representing a strain-switching event. While the rate of strain-switching events was thus increased for intervals spanning an alloHSCT or relapse event, the observed difference was not found to be statistically significant (*p* = 0.17; Fisher’s exact test). This investigation demonstrates that individual strains can be replaced by other strains of the same species during the course of alloHSCT.

## Discussion

Associations between the microbiome and alloHSCT outcomes are incompletely understood, in particular with respect to the role of non-bacterial domains of microbial life; studying these, however, is complicated by limitations of established technologies for microbiome characterization, such as 16S or 18S rDNA sequencing. We thus developed a method based on long-read shotgun metagenomics enabling interrogation of alloHSCT patient microbiomes across almost all domains of microbial life (bacteriome, archaeome, mycobiome, non-human/non-fungal eukaryome, and DNA-virome). It comprises robust protocols for sample preparation and DNA extraction as well as a specifically developed mapping-based bioinformatics approach to reduce the false-positive taxon detection rate associated with k-mer-based read classification.

We used the method to interrogate patient microbiomes in an explorative clinical study, and found that pre-Tx patient microbiomes fell into 3 clusters (see Fig. 4), which can be categorized as “*Bacteroides*- and *Phocaeicola*-dominated” (Cluster 1), “heterogeneous” (Cluster 2), and “*Enterococcus*-dominated” (Cluster 3). The included control samples clustered with the pre-Tx alloHSCT samples of Cluster 1. Interestingly, Cluster 1 exhibited high abundances of *Bacteroides*, often considered an important component of an “intact gut microbiome” and comprising many species known to be commensals and beneficial for human gut health^50^. Cluster 1 could thus be interpreted as least affected by alloHSCT-related microbiome dysbiosis; Clusters 2 and 3, by contrast, may be interpreted as more dysbiotic microbiome states^5^. Suggestive differences between the clusters were also detected at the level of viral and fungal microbiome components; for example, *Candida albicans* (of which an increase in HSCT is associated with adverse outcomes^28^) was detected more often in Cluster 2 and 3 samples , and *uncultured* crAssphage more often in Cluster 1 samples. Consistent with the interpretation of Cluster 1 as least dysbiotic, Cluster 1 also exhibited the lowest rates of antibiotic resistance genes, and patients in Cluster 1 had a lower median number of pre-alloHSCT treatment cycles (median = 2 treatment cycles) compared to patients in Clusters 2 and 3 (combined median = 4.5 treatment cycles; *p* = 0.01; Mann–Whitney U test). A meta study from 2020 previously showed a significant relation between antibiotics administration and acute GvHD^51^. Future studies are required to investigate whether pre-Tx cluster membership is predictive of alloHSCT treatment success since the statistical power necessary to reliably detect such associations cannot be achieved with the 31 patients recruited for our study.

Further applying the pipeline to longitudinally collected samples, we found that patient microbiomes developed dynamically over the course of alloHSCT. Consistent with previous studies^12,31^, we observed a reduction in microbiome diversity during leukopenia and partial recovery during the reconstitution period. Of note, the leukopenic period was also characterized by a marked increase in fungal abundances (see Fig. 3), and expansion of bacterial genera like *Enterococcus* (also observed in^5^) and *Streptococcus.* It should also be noted that the acquisition of samples during leukopenia was challenging and sometimes outright not possible. Therefore the analyzed samples throughout this period might represent a biased selection. During the reconstitution period, patient microbiomes collectively showed a return in similarity to pre-Tx states; at the level of individual patient trajectories, however, pre-Tx cluster membership was not predictive of microbiome composition during leukocytopenia (see Fig. 5) or reconstitution (see Fig. 6). The analyzed reconstitution samples exhibited significant heterogeneity with respect to the time point of sampling (Supplementary Table 3), and individual patients were also represented with multiple samples in this analysis (Supplementary Table 3). It may also reflect the heterogeneity of alloHSCT patient journeys and the significant effects of these on the microbiome^33^. Consistently, the enriched incidence of bacterial strain replacement around stem cell transplantation and relapse events further emphasizes the potential microbiome disruption in treated patients.

Intriguingly, our study also confirmed the relevance of non-bacterial taxa in the context of alloHSCT. First, we demonstrated robust detection of non-bacterial microbiome components in alloHSCT microbiomes, including, in particular, fungal (*Saccharomyces and Candida*) and viral (crAssphage) taxa. Archaea and non-fungal eukaryotes were also detected, but at lower relative abundances (see Fig. 3). In this context, the mapping-based validation component was instrumental in enabling the distinction between confident hits and likely false-positives. Second, while the absolute proportion of non-bacterial taxa was small in most sampled patient microbiomes, viruses and fungi accounted for more than 50% and 10% of total microbial reads in individual microbiomes, respectively. Third, varying overall proportions of non-bacterial abundances, differences between the investigated treatment phases with respect to the validated detection of species at ≥ 5% abundance (in the case of viruses), as well as individual microbiome trajectories indicated that the non-bacterial components of the microbiome also exhibited high inter-patient and temporal plasticity. Interestingly, *Saccharomyces cerevisiae* and *Candida albicans* were detected at high relative abundances throughout the course of alloHSCT, potentially indicating relative stability of these microbiome components over the course of alloHSCT. Of note, in contrast to earlier studies based on 16S or 18S rDNA sequencing, employing an unbiased metagenomic approach, our workflow enabled the unbiased measurement of the relative quantitative abundances of different types of microbial life. To the best of our knowledge, our study is one of the first few studies to employ shotgun metagenomics in the context of alloHSCT^5,30,52^, and, of these, the first to explicitly interrogate the mycobiome and DNA virome. Further studies are required to better characterize the relative stability of the fungal and viral microbiome components and the interplay between these and the bacterial microbiome, for instance at the level of bacteriophage-host relationships^53^.

With respect to our pipeline, potential directions for future work include the further improvement of sample handling and DNA extraction protocols, potentially focused on extracting high-molecular weight DNA while retaining robustness, as well as improvements to the bioinformatics analysis components. In the current implementation, significant proportions of reads remained unclassified (Fig. 2); furthermore, the observed mapping-based validation rates for archaea, non-fungal eukaryotes, and (although to a lesser extent) fungi suggested substantial rates of residual mis-classification within these taxonomic groupings (Supplementary Fig. 3). Classification accuracy may benefit from incorporating gut-specific reference databases^54,55^ or the usage of high-quality environment specific databases used in a taxonomy-agnostic way^56^. Moreover, the currently employed validation approach, based on using the proportion of reads with high-quality pairwise alignments as a proxy for distinguishing between likely true- and false-positive taxon detections, could be complemented with approaches based on horizontal genome coverage (which we explored with promising results for a likely false-positive eukaryotic hit, *Toxoplasma*; Supplementary Note 2), or analysis of k-mer distributions^57^. It is noteworthy that significant fractions of reads classified as human by Kraken2 did not validate in some samples (Supplementary Fig. 3). While we did not attempt an in-depth investigation, an improved understanding of this phenomenon may contribute to further reducing the rate of false-positive read assignments. An important feature of the current version of our workflow is that the functional analysis of microbiome samples covers the detection of ARGs; future versions could also incorporate analyses of metabolic pathways^58^ and virulence factors^52^. Now, after successfully having established a workflow, we can attempt to map established microbiome biomarkers associated with alloHSCT outcomes—such as the quantitative definition of “dominance” by^31^ as ≥ 30% abundance—onto the microbiome measurements produced by our method in future studies with sufficient patient numbers. As a prerequisite, a future calibration study should address factors such as the utilized extraction protocol, DNA sequencing technologies, and downstream bioinformatics, which influence the accuracy of microbiome measurements^59^). Importantly, in contrast to rDNA amplification-based approaches, our method enables the simultaneous, unbiased and quantitative measurement of human-derived DNA and the exploration of its biomarker potential, e.g., with respect to potential associations between human-derived DNA and mucosal integrity during leukopenia. Conceivably, such analyses could also include cell-of-origin analyses, leveraging the methylation detection capability of the Oxford Nanopore technology^60^.

In summary, we have presented a method for characterizing microbiomes simultaneously for their bacteriome, archaeome, mycobiome, non-human/non-fungal eukaryome, and DNA virome composition. Applying it in an exploratory study to a cohort of alloHSCT patients, we carried out one of the first investigations of an all-kingdom microbiome in the context of alloHSCT based on shotgun metagenomics, confirmed the potential relevance of non-bacterial microbiome components, and identified a 3-cluster structure of pre-Tx patient microbiomes. Furthermore, we could demonstrate that microbial strains can be newly acquired or replaced during the course of alloHCST. These findings can serve as an important basis for future studies, aiming at a more comprehensive characterization of the role of the microbiome in alloHSCT-patients based on shotgun metagenomics.

## Materials and methods

### Patient recruitment and sampling scheme

Patient recruitment for collection of clinical alloHSCT samples was carried out between June 2018 and December 2019. Participation in the study was offered to all patients undergoing alloHSCT at the Department of Hematology, Immunology, and Clinical Immunology at University Hospital Düsseldorf, according to the following inclusion criteria: persons of legal age that are about to receive an allogeneic stem cell transplantation at the UKD and are able to consent. A full description of the recruited cohort is given in Supplementary Note 1; patient characteristics and treatment histories are summarized in Supplementary Table 2 and Supplementary Table 8. Fecal samples from alloHSCT patients were collected prior to transplantation, during the leukopenic period (defined as white blood count of ≤ 1,000/µL), and after reconstitution. For some patients more than one sample per time point was collected which is shown in Supplementary Fig. 11. Stool samples were collected in standard stool collection tubes (feces tubes 76 × 20 mm; Sarstedt), stored at 4 °C if possible and transported for further processing to the Institute for Medical Microbiology and Hospital Hygiene of Heinrich Heine University Düsseldorf as fast as possible.

A convenience control cohort of 10 individuals not diagnosed with hematological malignancies was recruited from employees of Düsseldorf University Hospital between February 2021 and June 2021. Control samples were collected from each individual after inclusion into the study and processed using the same protocols also applied to the alloHSCT samples.

This study was approved by the ethics committee of the Medical Faculty of Heinrich Heine University Düsseldorf (2019–509, Registration Number 2019055096) and all methods were carried out in accordance with relevant guidelines and regulations. Informed consent was obtained from all subjects.

### Sample preparation and sequencing

Upon receipt, 1–4 aliquots from each stool sample of approximately 1 mL or 1 g of specimen each were placed into 1–4 labeled PowerBead Tubes containing 750 µL C1 buffer solution (Qiagen) each.

DNA extraction was carried out using a modified version of the HMP protocol^45^. Briefly, specimens were homogenized 30–40 s by vortexing and placed into a heating block at 65 °C and 95 °C for 10 min respectively. Samples were then stored at − 80 °C until DNA extraction. Aliquots for DNA extraction were thawed at 4 °C or room temperature (RT) and gently mixed by vortexing. 60 µL of C1 solution was added and briefly vortexed or inverted several times. Aliquots were vortexed at maximum speed for 10 min and centrifuged at 10,000 × g for 30 s at RT. From this step onwards, the DNeasy Power Soil Kit was used as per protocol. The concentration of the extracted DNA was determined by fluorometry using the Invitrogen Qubit 4 Fluorometer (Thermo Fisher Scientific) and aliquots were stored at -80 °C until the Nanopore and Illumina sequencing was performed.

Long-read Nanopore sequencing of all samples was carried out on the PromethION and MinION devices, using FLO-MIN106 R9-Version and FLO-PRO002 R9.4-Version flow cells, the SQK-LSK109 ligation sequencing kit, and the EXP-NBD104 or EXP-NBD114 barcoding kits for barcoded sequencing of native DNA, according to protocol NBE_9065_v109_revAA_14Aug2019. Basecalling was carried out using Guppy.

83 genomic DNA samples, selected to cover at least one pre-post pair per patient, were used for short-read Illumina sequencing. The samples were quantified by fluorometric assay (Qubit DNA HS Assay, Thermo Fisher Scientific) and their quality measured by capillary electrophoresis using the Fragment Analyzer and the ‘DNF-488 HS Genomic DNA Kit’ (Agilent Technologies, Inc.). Although the initial concentration of some samples was low, with some of them not measurable at all, we processed all of them, since the library preparation kit is also suitable for small input quantities. Library preparation was performed using the ‘Nextera XT DNA Library Preparation Kit—Document # 15,031,942 v05 (Illumina, San Diego, USA.). Depending on whether the concentration of the sample could be determined, either 1 ng gDNA or 5 µL of the sample volume was used for the tagmentation step. Library amplification for final enrichment was performed with 13 cycles. Bead purified libraries were normalized and subsequently sequenced on the HiSeq3000 system (Illumina, San Diego, USA) with a read setup of 2 × 151 bp. The bcl2fastq2 tool was used to convert the bcl files to fastq files as well for adapter trimming and demultiplexing.

DNA extraction and sequencing data generation metrics are summarized in Supplementary Table 3.

### Analysis of long-read sequencing data

Long-read sequencing data were analyzed using a comprehensive reference database (“MetaGut v. 1.0 database”) comprising 292 giga base pairs of sequence and 1.5 million microbial genomes from all domains of microbial life (Supplementary Table 10). We constructed a custom Kraken2 database by using the Kraken2 build scripts for all domains, modified to download all assemblies marked as “representative genomes” (as opposed to just “Chromosome” or “Full Genome” assemblies) for fungi and protozoa. In addition, we included the *Mus musculus* reference genome (GCF_000001635.27_GRCm39). A full breakdown of database contents is shown in Supplementary File 1. Read classification and compositional analysis were carried out using a 2-step approach that combined an initial k-mer-based read assignment step with mapping-based validation. During the first step, all reads of a sample were classified using Kraken2^47^, yielding an initial estimate of sample composition. During the second step, the presence of species (and genera) detected during the first step was validated using minimap2^61^.

Briefly, to validate the presence of a species in a sample corresponding to node *n* in the reference database taxonomy, the following steps were carried out: (i) All sample reads assigned to node *n* and its descendants were collected; (ii) a combined reference genome for node *n* was created by linearly concatenating the reference genomes of all leaf-level descendants of node *n*; (iii) the collected reads for node *n* were mapped against the combined reference genome for node *n*; (iv) the proportion of mapping-validated reads was determined, where “mapping-validated” was defined as ≥ 70% of a read being covered by read-to-reference alignments with ≥ 70% identity; (v) finally, the species corresponding to node *n* was defined as “validated” in a sample if ≥ 20% of the sample reads assigned to *n* achieved mapping-based validation. To increase computational efficiency, validation was carried out on a random subsample of 100,000 reads in each sample (or on the full sample if it contained fewer reads).

Of note, due to low per-species validation rates of archaea and non-fungal eukaryotes (see Results), analyses of the occurrence and abundance of individual species were limited to the bacterial, viral and fungal components of the microbiome.

### Determination of read validation thresholds

The utilized read validation thresholds were defined empirically and based on an analysis of the Zymo Gut Microbiome Standard (https://www.zymoresearch.de/products/zymobiomics-gut-microbiome-standard). Briefly, the community standard was long-read-sequenced, employing the same protocols used for fecal samples. The generated reads were classified against the MetaGut database using Kraken2, and the assignments of individual reads were validated, using the 70%/70% criterion defined in the previous section, by mapping against (a) a database constructed from the Zymo-provided reference genomes of the species in the Zymo standard, and (b) the MetaGut reference database.

The analysis could not be conducted for the species *Veillonella rogosae* and *Prevotella corporis* since they were not present in the database.

When using the the Zymo-provided reference genomes for read validation (i.e. representing the assumed case that the genomes of all taxa present in the sample are perfectly represented in the reference database; but see below), per-genus read validation rates varied between 82.4% and 99.3%, with the exception of *Candida albicans, Salmonella enterica*, *Clostridium perfringens* and *Enterococcus faecalis* (Supplementary Table 11). 0 of the 2 and 3 of the 5 reads assigned to *Clostridium perfringens* and *Enterococcus faecalis*, respectively, could be validated, indicating that these represent false-positives; this is consistent with an expected absolute read count (relative theoretical abundance x number of generated reads) for these species of 1 and 0, respectively. *Candida albicans and Salmonella enterica* exhibited read validation rates of 50.2% and 69.1%; we hypothesized that these could be explained by a mismatch between the *Candida* and *Salmonella* genome present in the sequenced Zymo sample and the Zymo-provided reference genome. We tested and confirmed this hypothesis by de novo assembly of the Zymo sequencing data, followed by mapping of the assembled contigs against the database constructed from the Zymo-provided reference genomes using FastANI^62^, weighted by assembled contig length; in this analysis, *Candida albicans* appeared as a clear outlier (FastANI estimates of 95.65%; Supplementary Table 12), indicating genetic divergence. No matching contigs were assembled for *Salmonella enterica*.

When using our comprehensive reference database for read validation (i.e. representing the case of potential divergence between the genomes in the sample and the next-closest database genomes) per-species read validation rates varied between 81 and 100% for species truly present in the Zymo standard (excluding *Enterococcus* and *Clostridium* due to low abundance), and between 0 and 100% for false-positive species (Supplementary Table 11). We hypothesized that per-taxon read validation rates would vary with the genetic distance between the sequenced and next-closest database genomes and confirmed this hypothesis by carrying out a correlation analysis (Pearson’s r = 0.484 for the correlation between read validation rate and FastANI estimate for all species excluding *Enterococcus faecalis* and *Clostridium perfringens*; *p* = 0.094).

Based on these results, we decided that pragmatically employing a per-taxon read validation rate threshold of 20% would conservatively enable us to filter out false-positive classification results even in the presence of genetic divergence between the genomes present in the sequenced samples and the genomes present in our database.

### Benchmarking of read validation method

To validate the validation method we generated a ground truth set consisting of reads and high-confidence assignments to references. We used the Zymo Gut Microbiome Standard and constructed a database by combining the Zymo-provided reference genomes with 185 additional contigs generated by the above mentioned de novo assembly (using Flye 2.9.1-b1780)^63^. To select suitable contigs we calculated the FastANI distances to the Zymo-provided references and selected references that had a) a similarity of 90% or higher to any reference b) a decrease in at least 1% to the next fitting reference and c) a length of at least 10,000 base pairs. The best match was used to label the contig.

We then mapped the reads exceeding a length of 500 base pairs using minimap2 requiring 80% of the read to align with at least 80% identity. This way we were able to assign 94% of the reads to a species in the Zymo Gut Microbiome Standard.

To quantify precision and recall of our validation we then applied Kraken2 with our full database on the readset (baseline) and performed the validation on species level. In addition, we removed each of the 17 species contained in the Zymo Gut Microbiome Standard from the database (rebuilding the entire index structure every time using the Kraken2 ‘fast-build’ option). Refer to Supplementary Table 13 for a full presentation of the results.

The baseline experiment on species level already detects 1641 taxa i.e. the majority of detected Kraken2 species are false positive. Since many of those are discarded by default due to low read counts and not considered for the validation we focus exclusively on those taxa for which validation is performed.

An ideal classifier would label reads from a species which is no longer present in the database as either “unclassified” or as belonging to a taxon that is an ancestor of the species. However, we observe that the majority of the reads gets reassigned to different orthogonal species producing numerous additional taxa that are not observed in the baseline experiment. Manual inspection reveals that many misassignments are to closely related species or entries that are only distant due to the underlying taxonomy and its labeling (for example *E. Coli* reads getting assigned to species of the genus *Shigella*).

Overall the recall for FP taxa is consistently high, varying between 75 and 99%. The recall for reads is significantly lower, varying between 5 and 10%. We hypothesize that this is due to the already high amount of false positive species assignments in the baseline experiment and the large amount of closely related species contained in the database that remains a challenge for LCA based approaches.

The precision of our filter is constantly 100% across all experiments, thus we never filter any real Zymo species.

### “Kitome” and contamination analysis

To rule out a significant contribution of environmental contamination we also performed DNA extraction without stool followed by a sequencing run with three barcodes employing the default wetlab protocol. Here, the amount of DNA was below the detection limit of the Qubit Fluorometric analysis. Within the few obtained sequence reads a small amount of human reads and reads belonging to physiological skin microbiota such as Micrococcus luteus and Cutibacterium acnes could be validated. We thus conclude that the potential impact of environmental / Kitome contamination in our samples can be neglected.

### Zymo and LifeLines-DEEP compositional analysis

Zymo community standard long-read sequencing data (see previous section) were mapped to Zymo-provided reference genomes using minimap2 and classified using the default workflow and the MetaGut database. Results were analyzed visually and by correlation analysis against the Zymo-provided expected abundance distribution. Whole-genome short-read sequencing data were obtained for 20 samples randomly selected from the Lifelines-DEEP cohort study (http://www.lifelines.nl), and classified using Kraken2 against the MetaGut v. 1.0 database using an absolute read cutoff of 5 reads and excluding reads classified as Viridiplantae.

### Microbiome cluster analysis

To investigate high-level microbiome structures, a PCoA analysis of all samples (alloHSCT and control cohorts), based on the Bray–Curtis distance calculated on the fractional compositional estimates as reported by our pipeline post filtering, was carried out. Microbiome clusters were identified by performing k-means clustering on the PCoA components and scanning k for 2 through 6 for the best silhouette value which was achieved for k = 3.

### Diversity analysis

Reported numbers of species were measured after downsampling each sample to the smallest number of reads observed in any sample (n = 1029 reads). Reads mapping to any taxon within Chordata or Viridiplantae were ignored.

### ARG gene detection

Long-read sequencing data were mapped using minimap2 against the CARD database^64^, comprising 2614 antibiotic resistance elements. A read was counted as carrying a specific ARG if a pairwise alignment with length ≥ 500 and < 50 undefined “N” characters was detected between the read and the ARG sequence. The ARG gene detection pipeline was applied to all reads from a sample, independent of other pipeline components.

### crAssphage analysis

Long-read sequencing data were mapped using minimap2 against resolved crAssphage sequences^48^ A read was counted as emanating from a crAssphage genome if a pairwise alignment with ≥ 70% of query cover at 70% identity was detected between the read and a crAssphage reference sequence; the fraction of primary alignments divided by the number of query reads was used as an estimate for crAssphage abundance. To identify reads that could be confidently assigned to individual strains, filtering based on mapping quality (MQ ≥ 5) was carried out, enabling assignment of 58% of reads. The crAssphage analysis component was applied to all reads from a sample, independent of other pipeline components.

### Tracking of marker taxa

A comprehensive list of marker taxa reported to be associated with alloHSCT outcomes was assembled from the literature (Supplementary Table 9), and complemented with selected fungal and bacterial marker taxa detected in this study. The final list of taxa is summarized in Supplementary Table 9. Longitudinal abundance dynamics were assessed visually, with confidence intervals in the plots representing binomial fraction confidence intervals.

### Detection of strain replacement events

Analysis of strain dynamics was based on (i) a novel measure (“aANI-lowFreq”) robustly approximating average nucleotide identities between the majority bacterial strain of a species present in a sample *a* and bacterial strains of the same species present in another sample *b* from short-read sequencing data and robust even in the presence of low-frequency strains; (ii) empirical calibration of expected aANI-lowFreq values for samples carrying different dominant bacterial strains using short-read data from different patients, based on the assumptions that (a) bacterial species will vary in their aANI-lowFreq distributions and (b) that different patients will typically be colonized by different bacterial strains; (iii) visual assessment of aANI-lowFreq distances for within-patient longitudinal samples from the same patients; a strain replacement event was assumed to have taken place if the observed within-patient aANI-lowFreq distance falls within the observed between-patient distribution. We note that our approach was optimized for reducing the rate of false-positive strain replacement events, potentially trading off sensitivity against precision. We also note that we experimented with MetaSNV 2^65^; however, the subpopulation module failed due to insufficient substructure detection on each species.

aANI-lowFreq is based on counting, over shared regions of species-specific core genomes, the number of bacterial SNVs exclusive to sample *a*, i.e. not also found in sample *b*. As a strain replacement event is most clearly indicated by the presence of a novel majority bacterial strain in *a* that is not present in *b*, *a*-exclusive SNVs were defined as positions that carried an allele with ≥ 50% frequency supported by a minimum of 10 sequencing reads in sample *a* and ≥ 10% frequency in sample *b*; the 10% threshold was chosen to allow for the presence of sequencing errors and mis-aligned reads, and, at the utilized threshold on read coverage (see below), often translated to rejecting alleles that were observed on a single sequencing read on sample *b*. A set of species-specific core genome sequences was obtained from the proGenomes2 representative species level reference database^66^; a position in one of these core genome sequences was defined as “shared” if both the individual position as well the surrounding core genome reference genome contig across ≥ 80% of its positions achieved ≥ 10 × coverage in *a* and *b*. The aANI-lowFreq distance between *a* and *b* was defined as the count of *a*-exclusive SNVs divided by the length of the genomic regions classified as “shared” between *a* and *b*.

aANI-lowFreq was validated using simulation by obtaining 100 randomly selected NCBI RefSeq genomes for *Enterococcus faecium*, *Phocaeicola vulgatus*, *Escherichia coli*, and *Bacteroides uniformis*, as well as 72 genomes for *Lactobacillus gasseri*. For each genome, paired-end 2 × 100 bp short-read sequencing data at different coverage levels (5, 10, 20, 50, 200, and 1000) were simulated using dwgsim 0.1.14^67^. For each species and combination of coverage levels, we generated 50 random pairs of different genomes as well as 15 random pairs of identical genomes; for each pair, the aANI-lowFreq distance was calculated based on the simulated short reads, and a reference ANI (based on the NCBI RefSeq genome sequences) was obtained using FastANI^62^. The simulations demonstrated that from genome-wide coverages of ≥ 20x, substantial core genome proportions were classified as “shared” (Supplementary Fig. 8), and good approximation of FastANI by aANI-lowFreq, at slight levels of ANI underestimation (demonstrating conservativeness of aANI-lowFreq; Supplementary Fig. 9).

For detection of strain replacement events within the alloHSCT cohort, only species and sample pairs with at least 50% of the corresponding core genome were considered, and only species for which at least 10 between-patient aANI-lowFreq distances were available for calibration. For the visual assessment step (Supplementary Fig. 10), within-patient and between-patient aANI-lowFreq distances were plotted alongside each other, together with aANI-lowFreq distances from the RefSeq-based simulations, when available.

### Supplementary Information

Supplementary Figure 1.

Supplementary Figure 2.

Supplementary Figure 3.

Supplementary Figure 4.

Supplementary Figure 5.

Supplementary Figure 6.

Supplementary Figure 7.

Supplementary Figure 8.

Supplementary Figure 9.

Supplementary Figure 10.

Supplementary Figure 11.

Supplementary Figure 12.

Supplementary Information 1.

Supplementary Information 2.

Supplementary Information 3.

Supplementary Information 4.

Supplementary Table 2.

Supplementary Table 3.

Supplementary Table 4.

Supplementary Table 5.

Supplementary Table 6.

Supplementary Table 7.

Supplementary Table 8.

Supplementary Table 9.

Supplementary Table 10.

Supplementary Table 11.

Supplementary Table 12.

Supplementary Table 13.

Supplementary Table 14.

Supplementary Table 15.

## Data Availability

The sequencing data—filtered for human DNA—is available at SRA under BioProject Identifier: PRJNA929328. Raw sequencing data and additional patient metadata are available upon reasonable request from the corresponding author.

## Code availability

The bioinformatics workflow was made available under an Open Source license as a reproducible Snakemake^68^ workflow with the DOI: https://doi.org/10.5281/zenodo.8121116. Each step of the pipeline is executed within its own Conda environment, enabling finely-grained assignment of memory and CPU resources. Downstream analysis was carried out using a set of Jupyter Notebooks^69^, also available at the DOI given above.
