# Supplementary figures and images for: Insights into gut microbiomes in stem cell transplantation by comprehensive shotgun long-read sequencing
Source: Sci Rep. 2024 Feb 19;14:4068. doi: 10.1038/s41598-024-53506-1 (PMC10876974; doi:10.1038/s41598-024-53506-1)

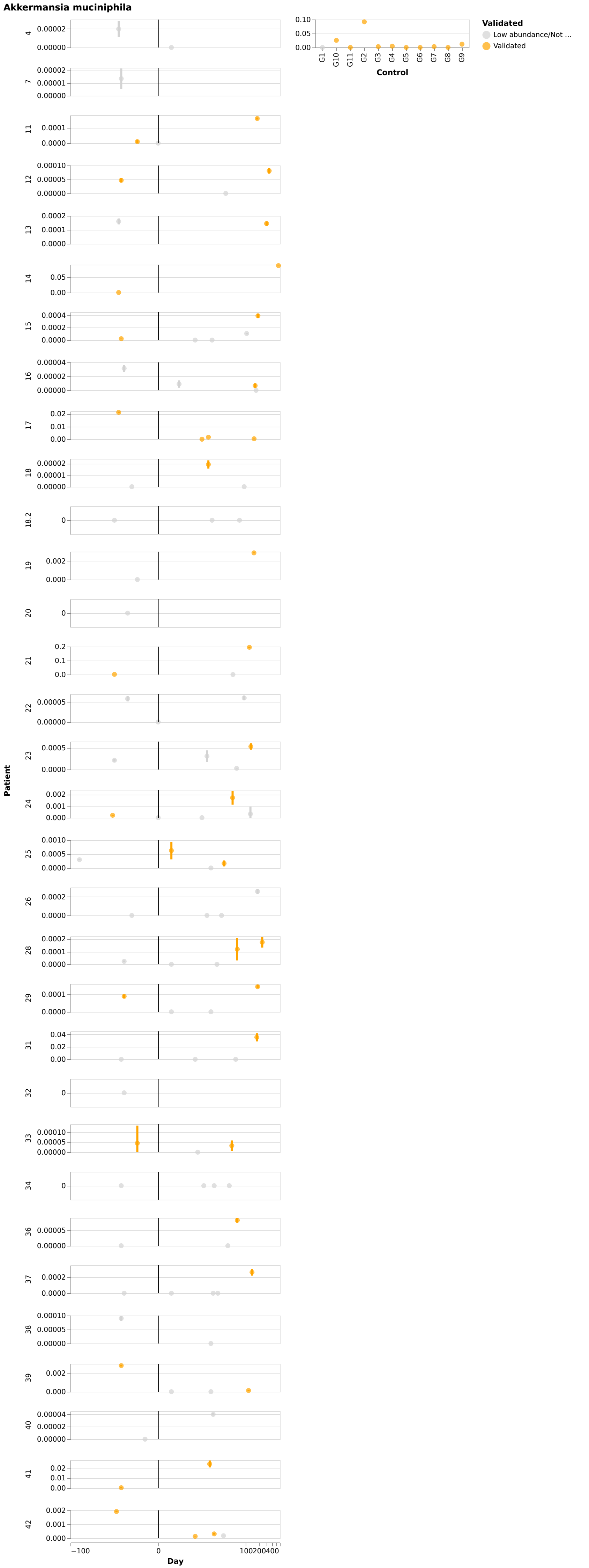

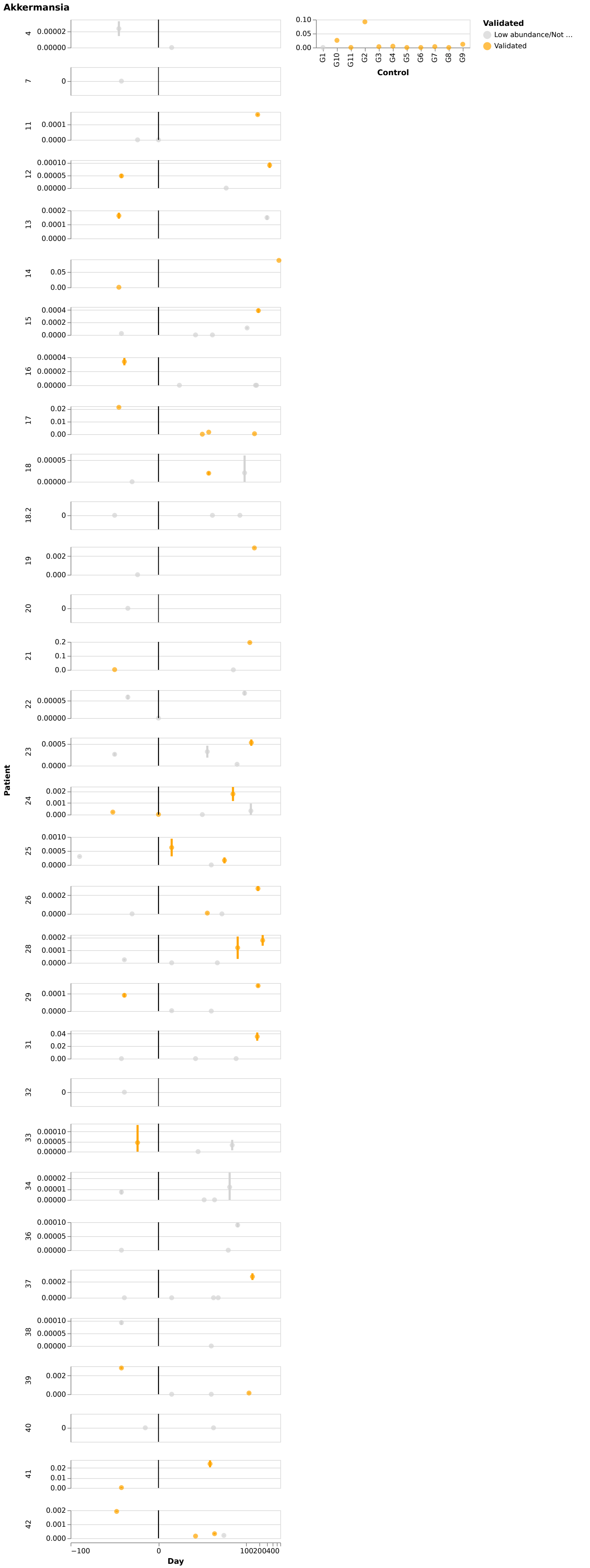

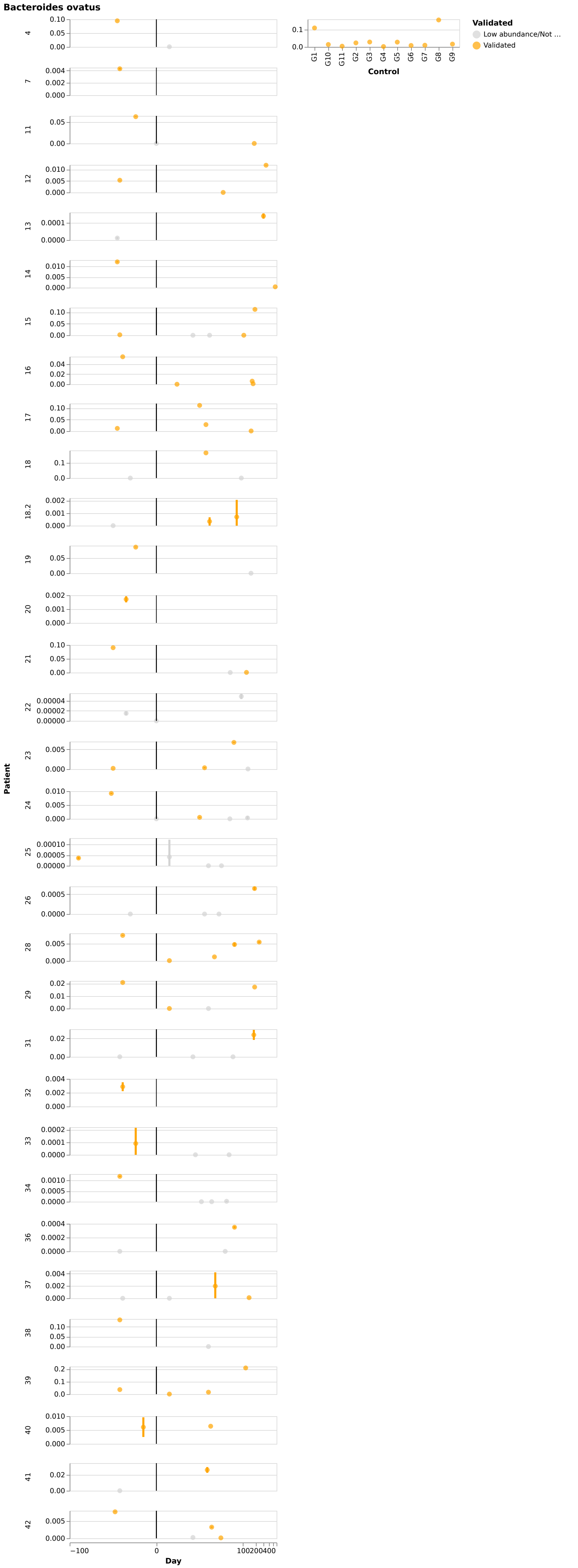

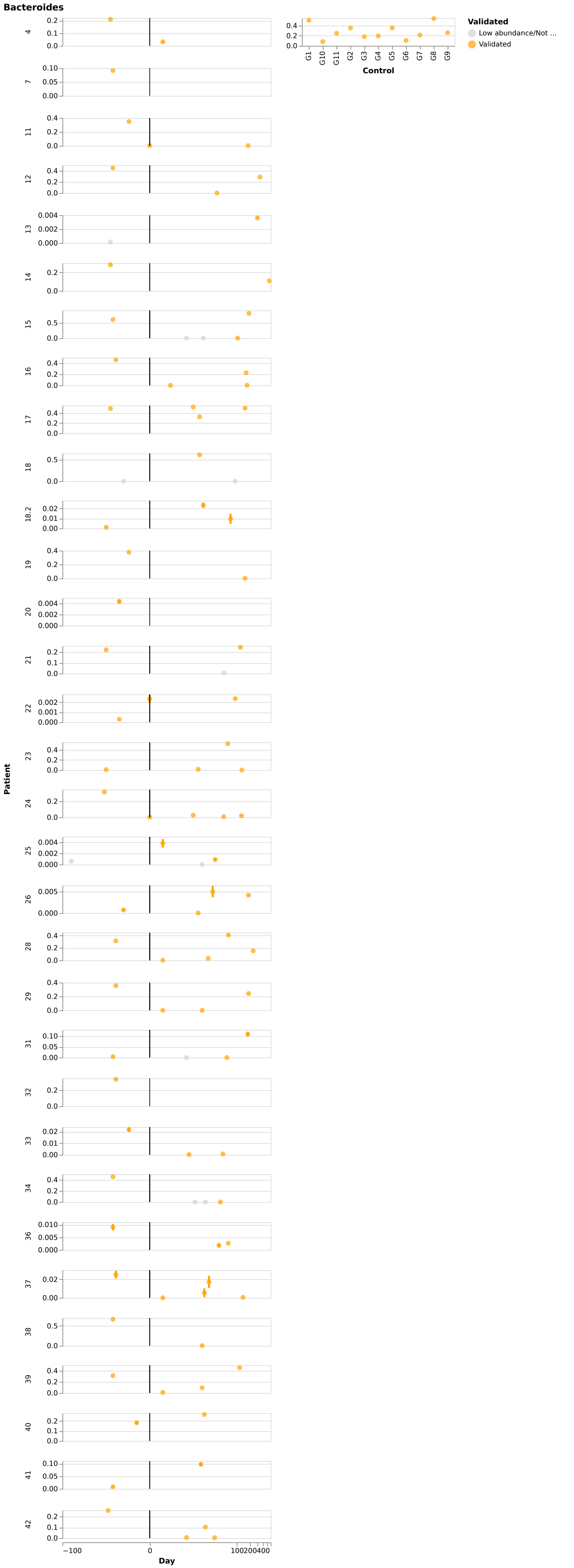

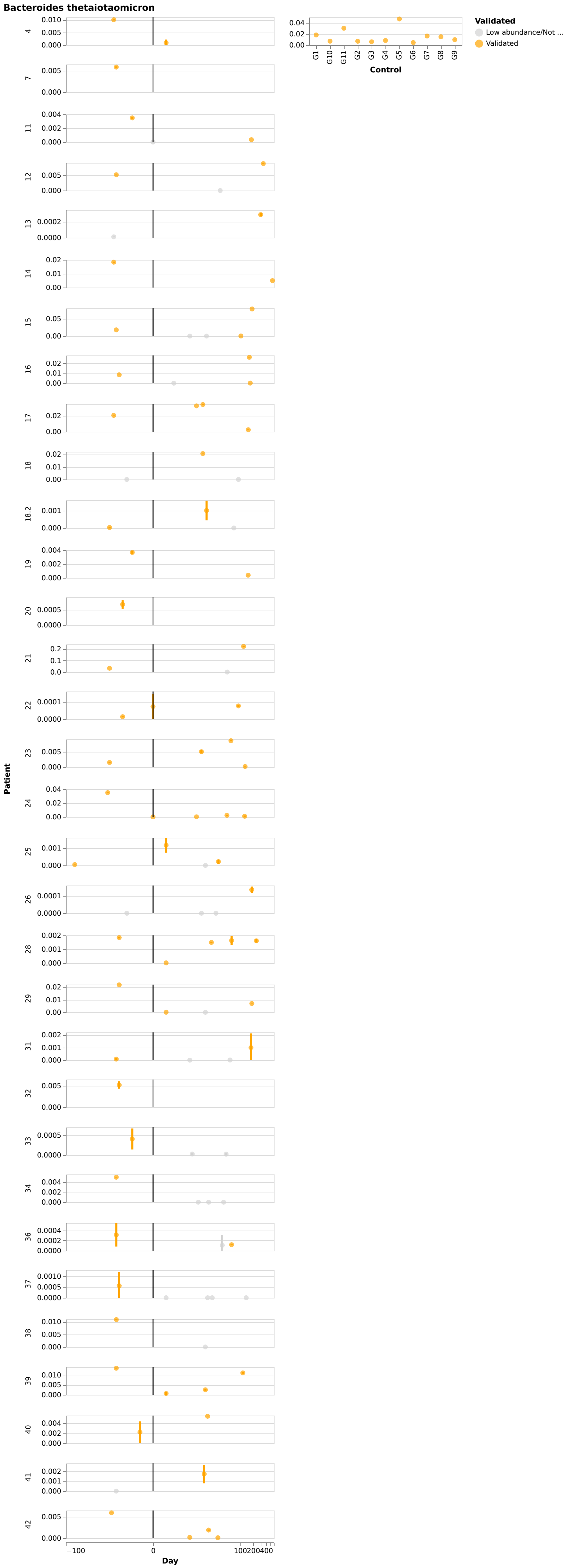

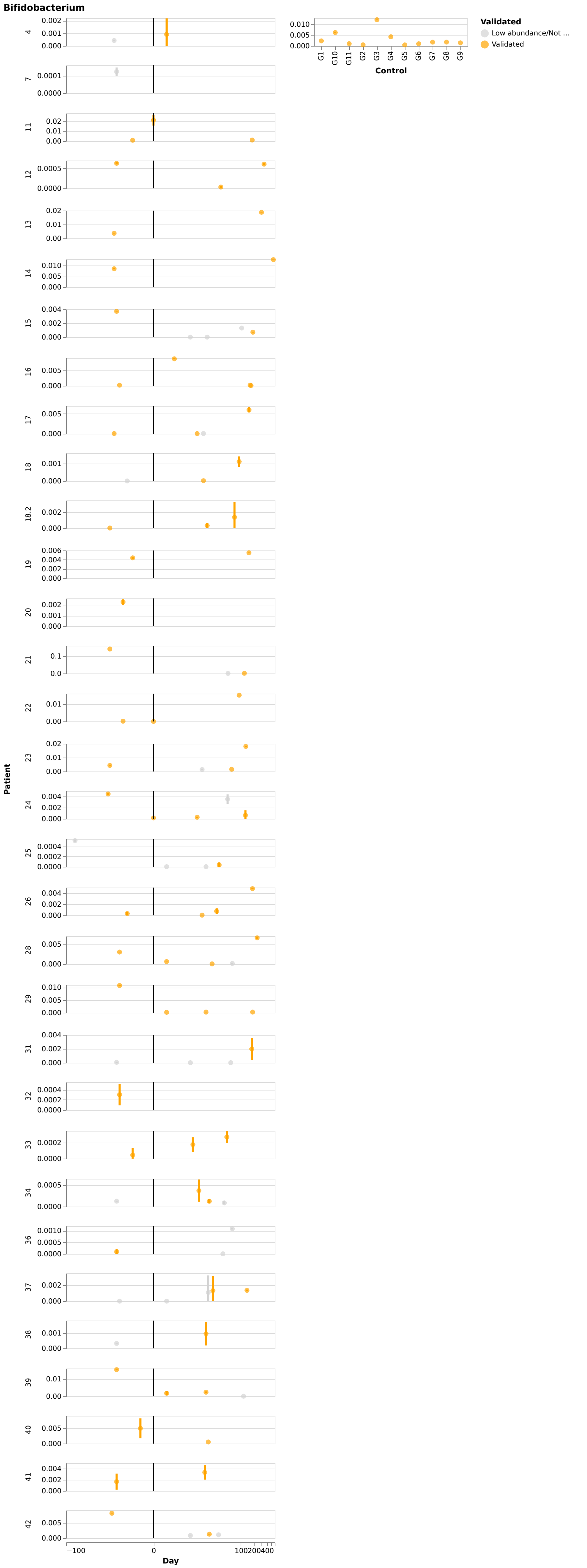

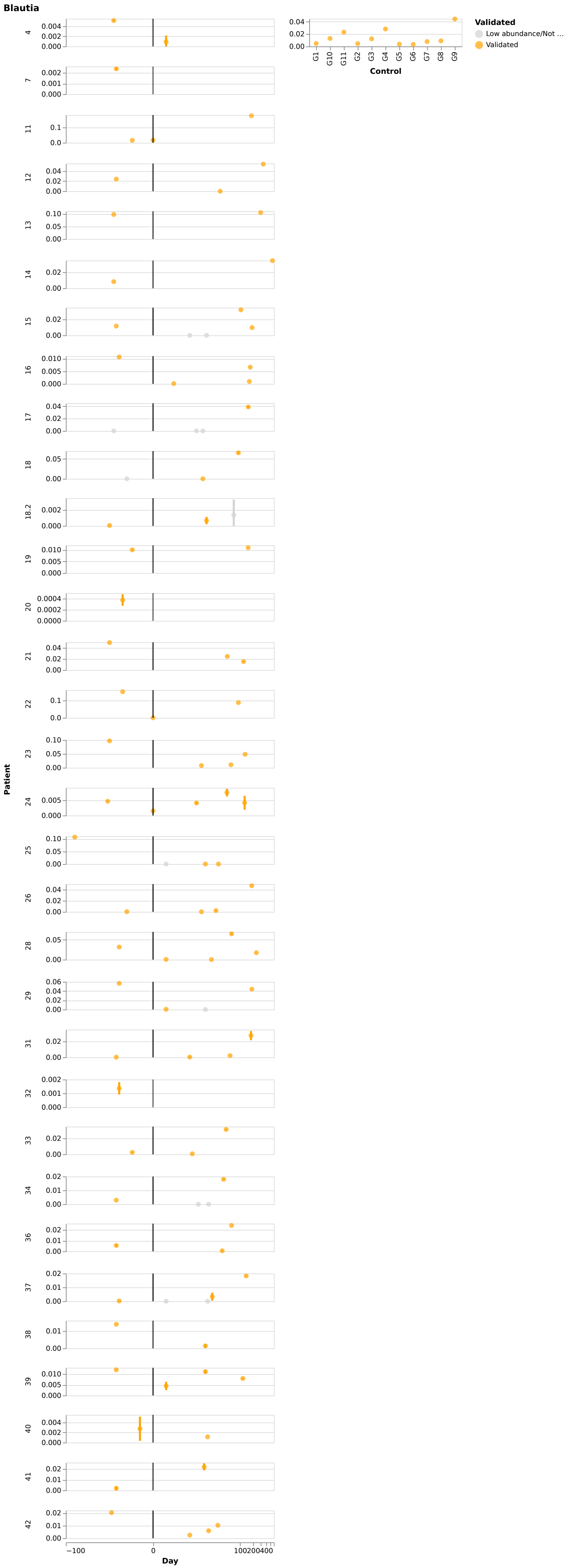

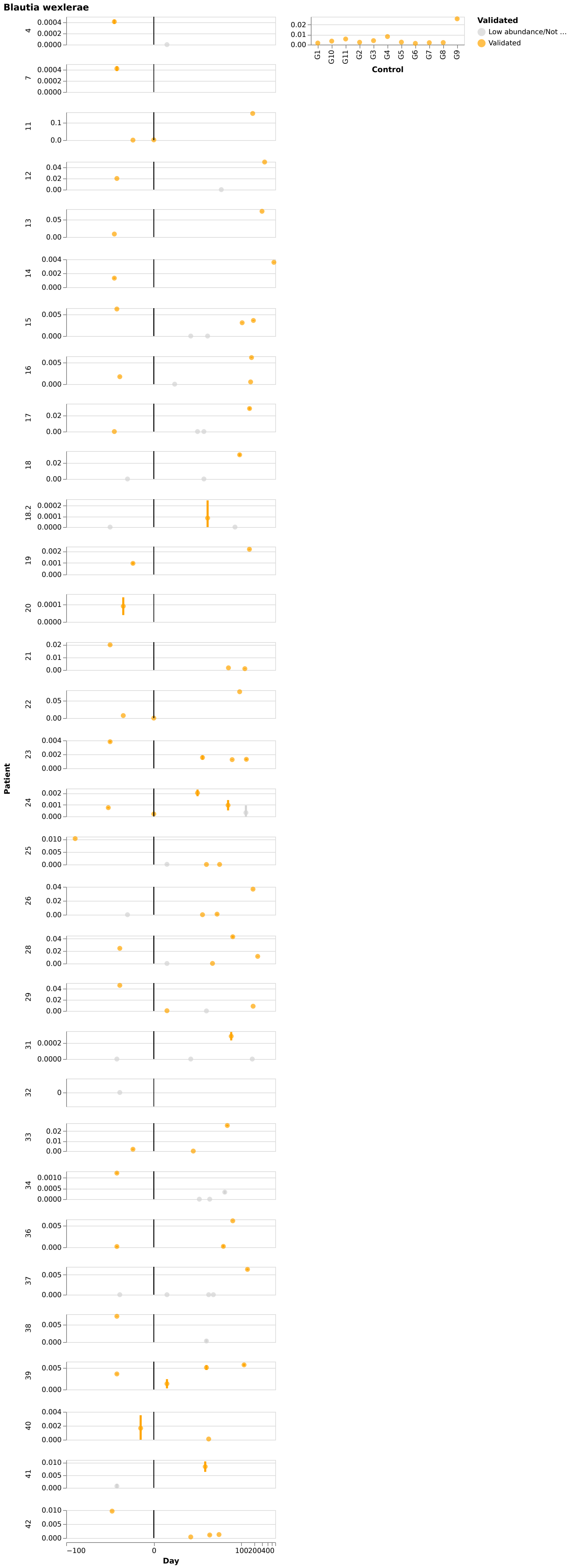

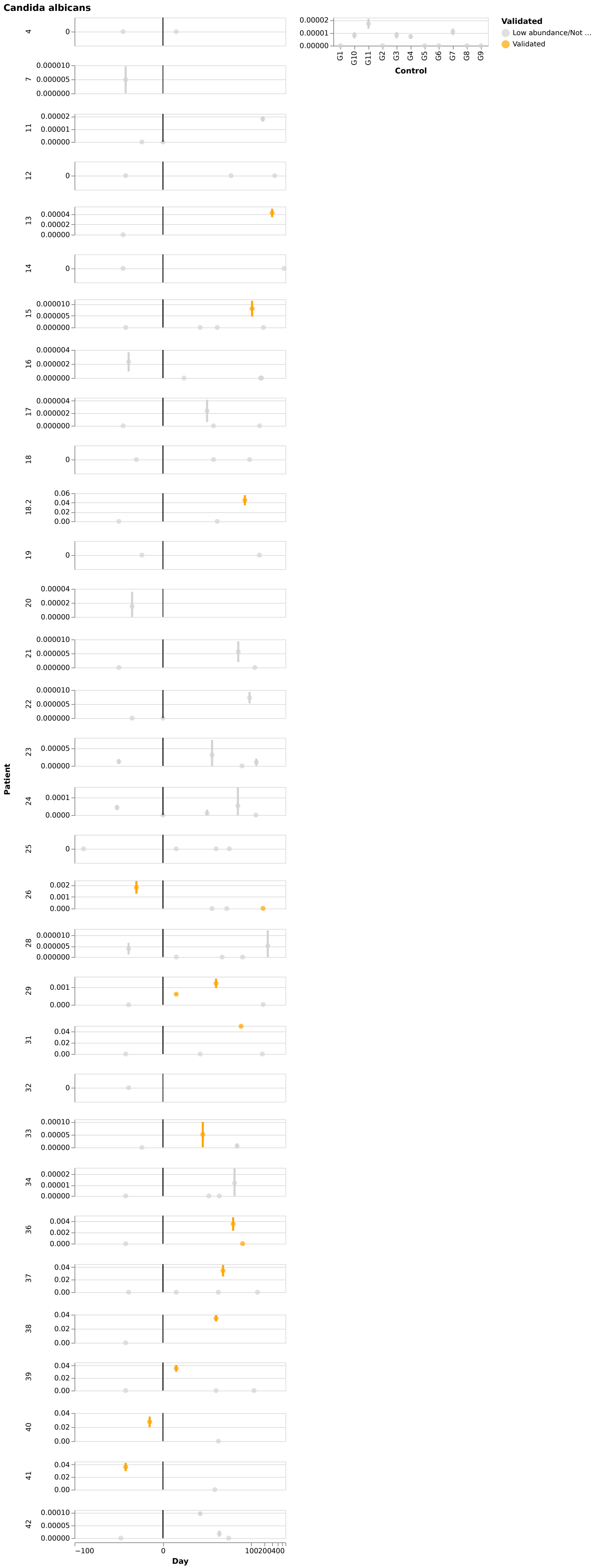

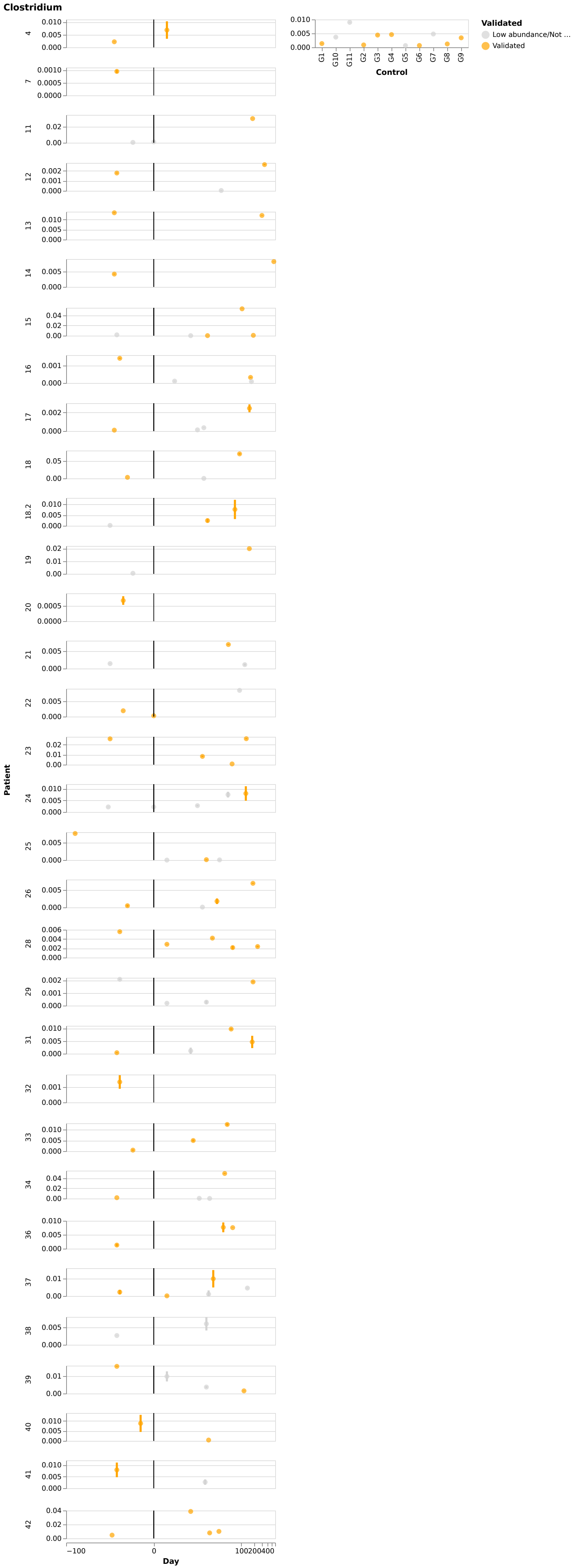

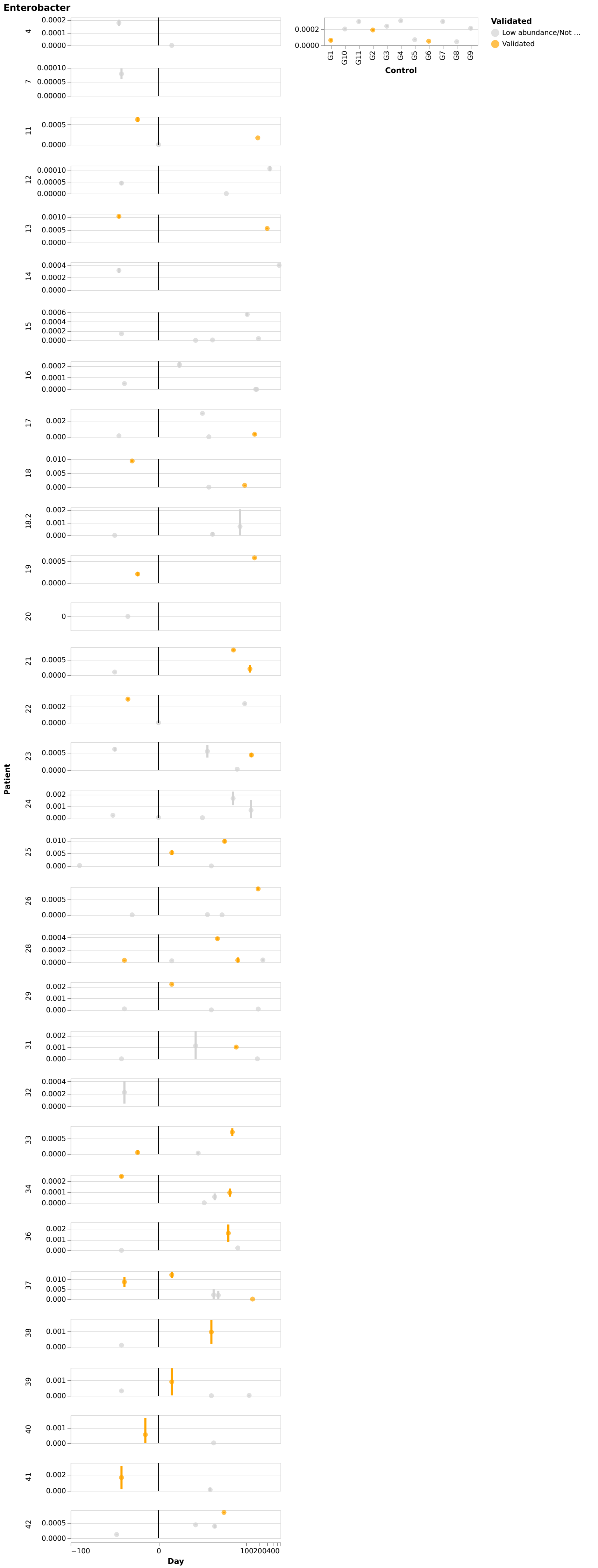

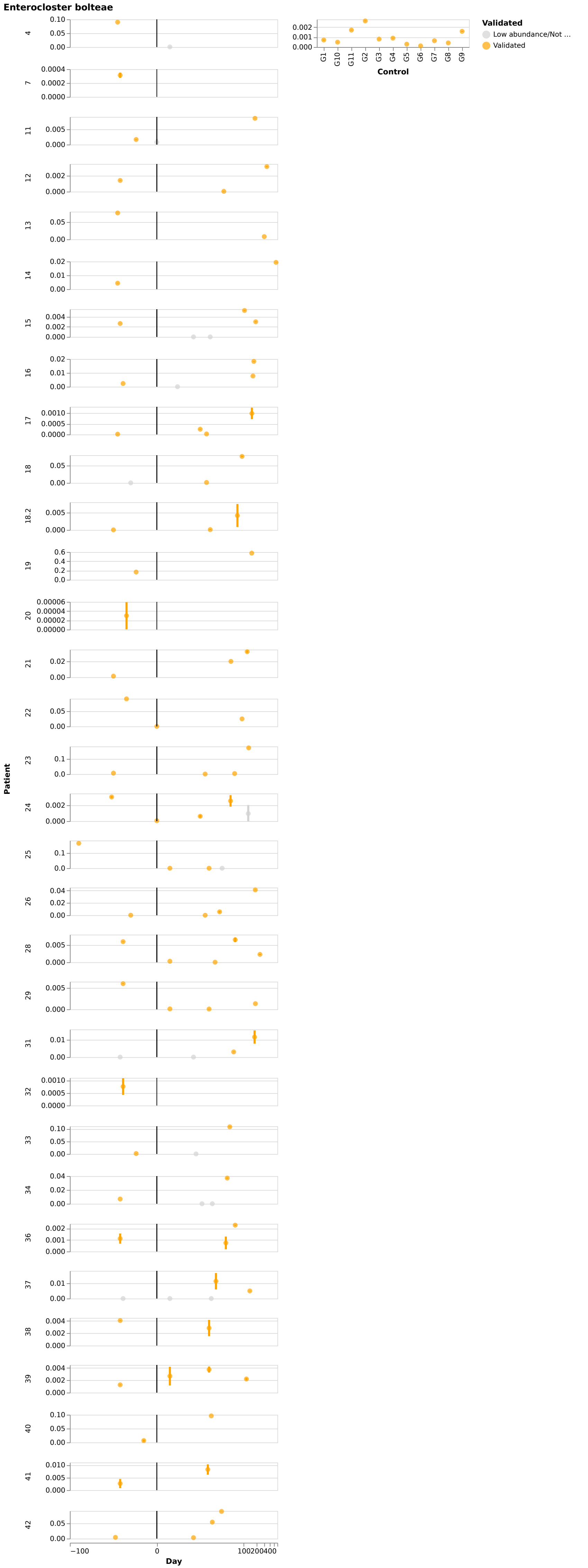

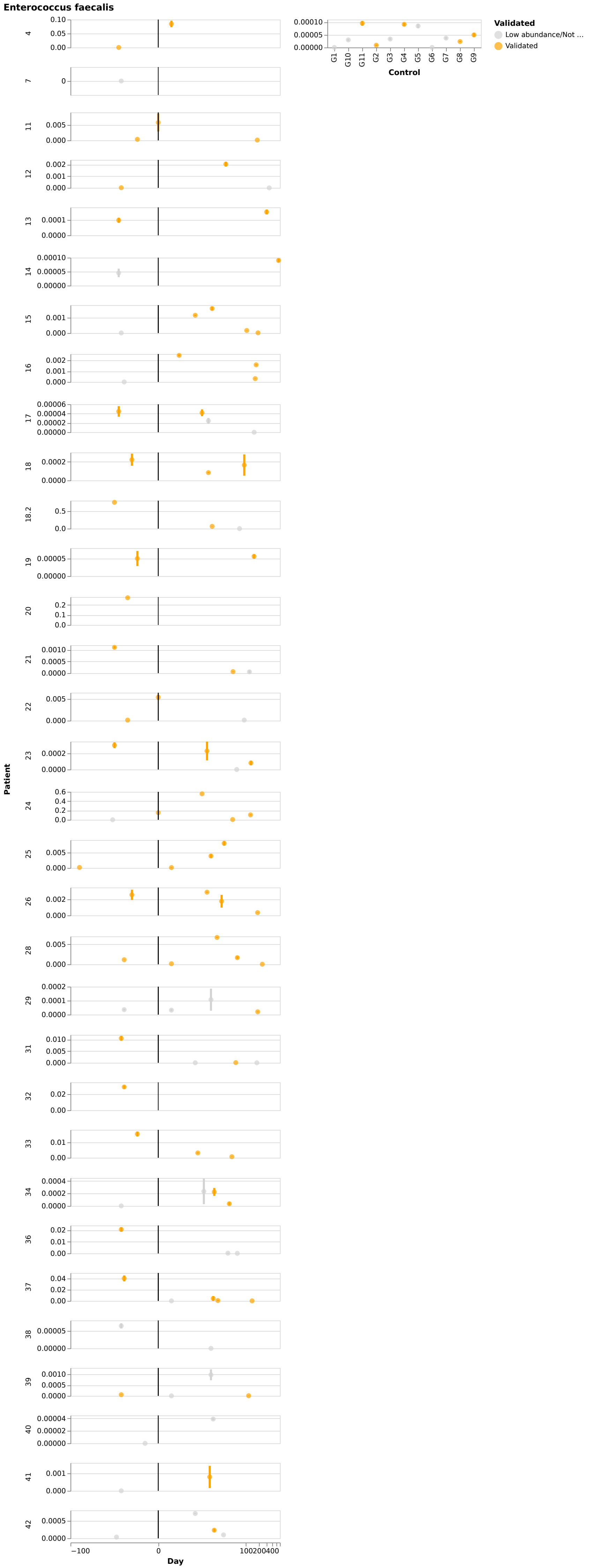

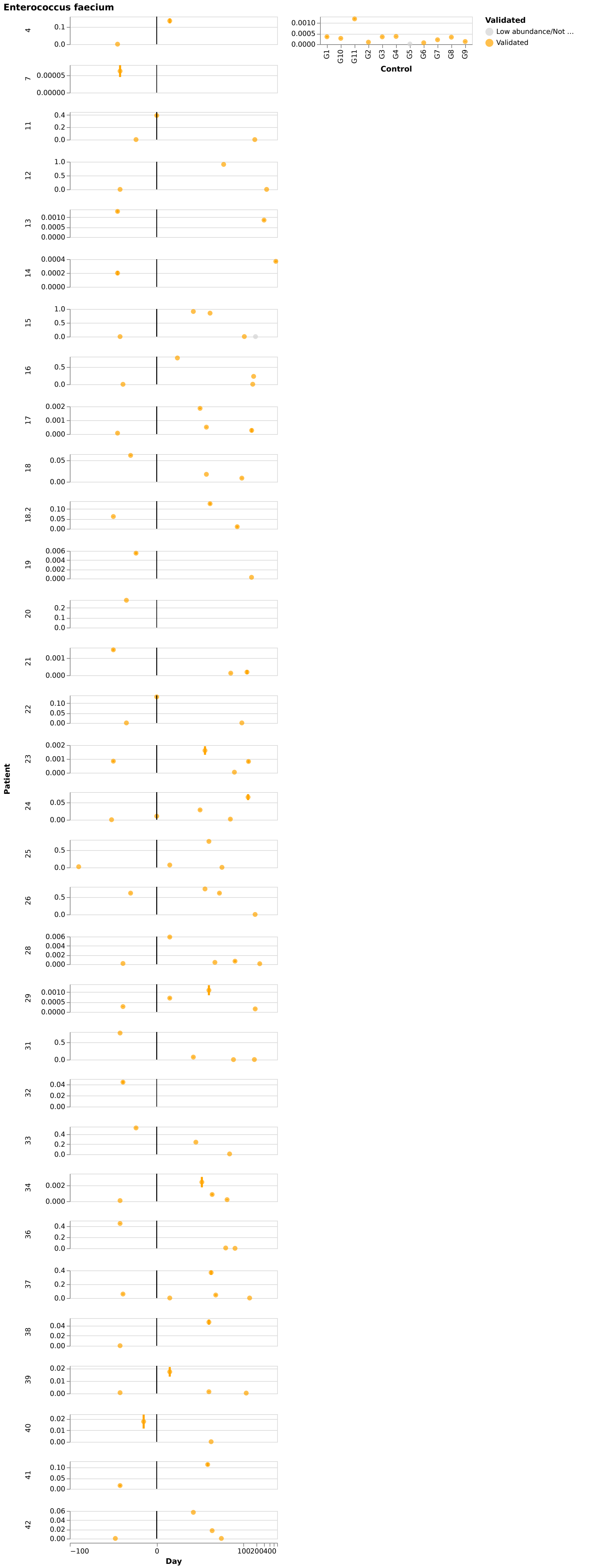

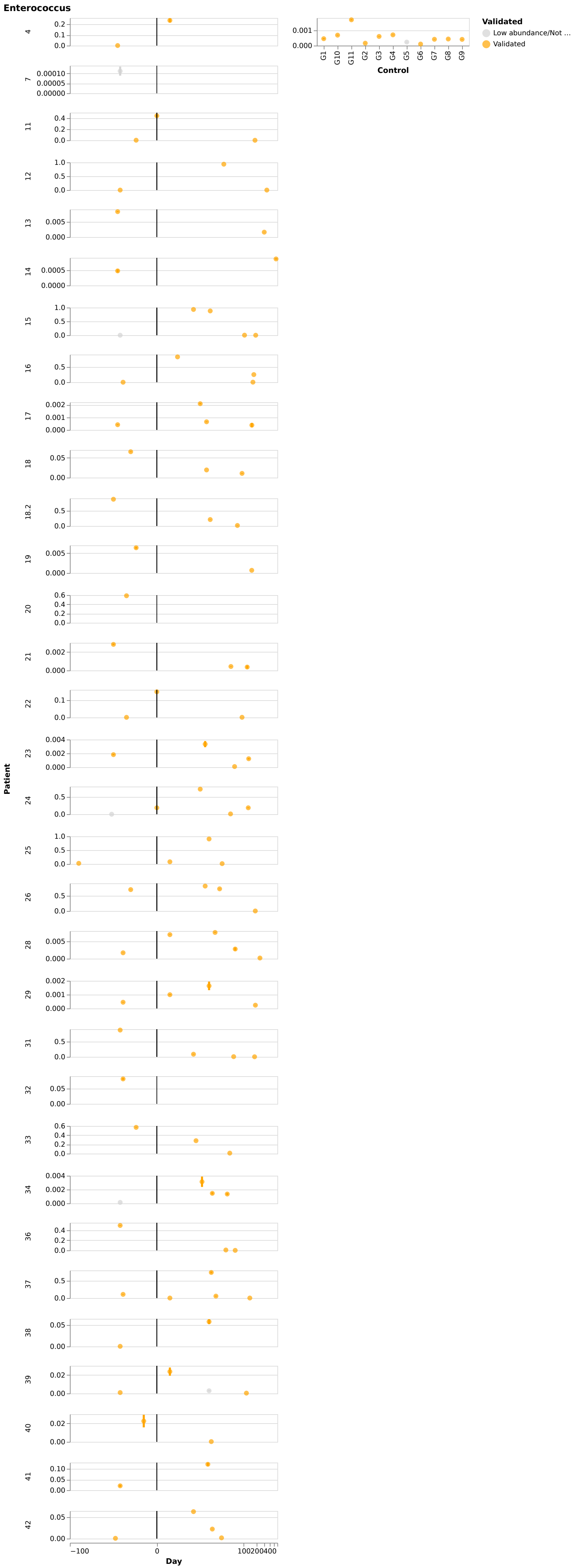

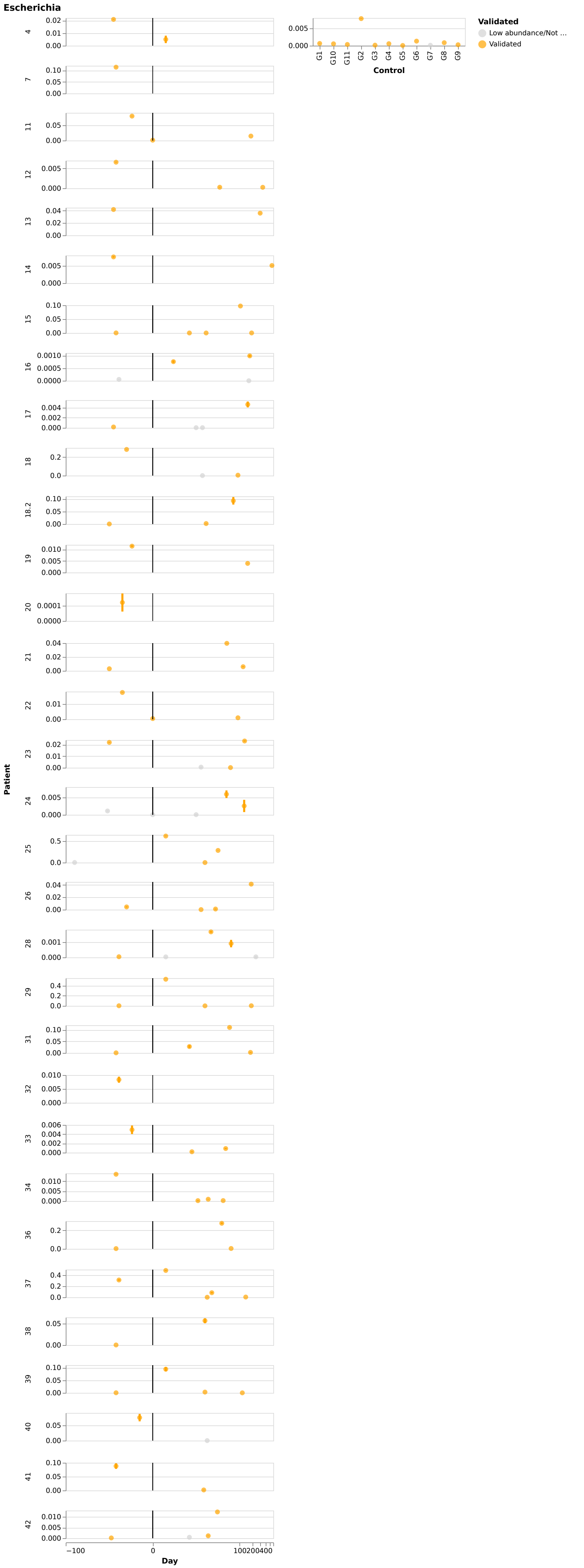

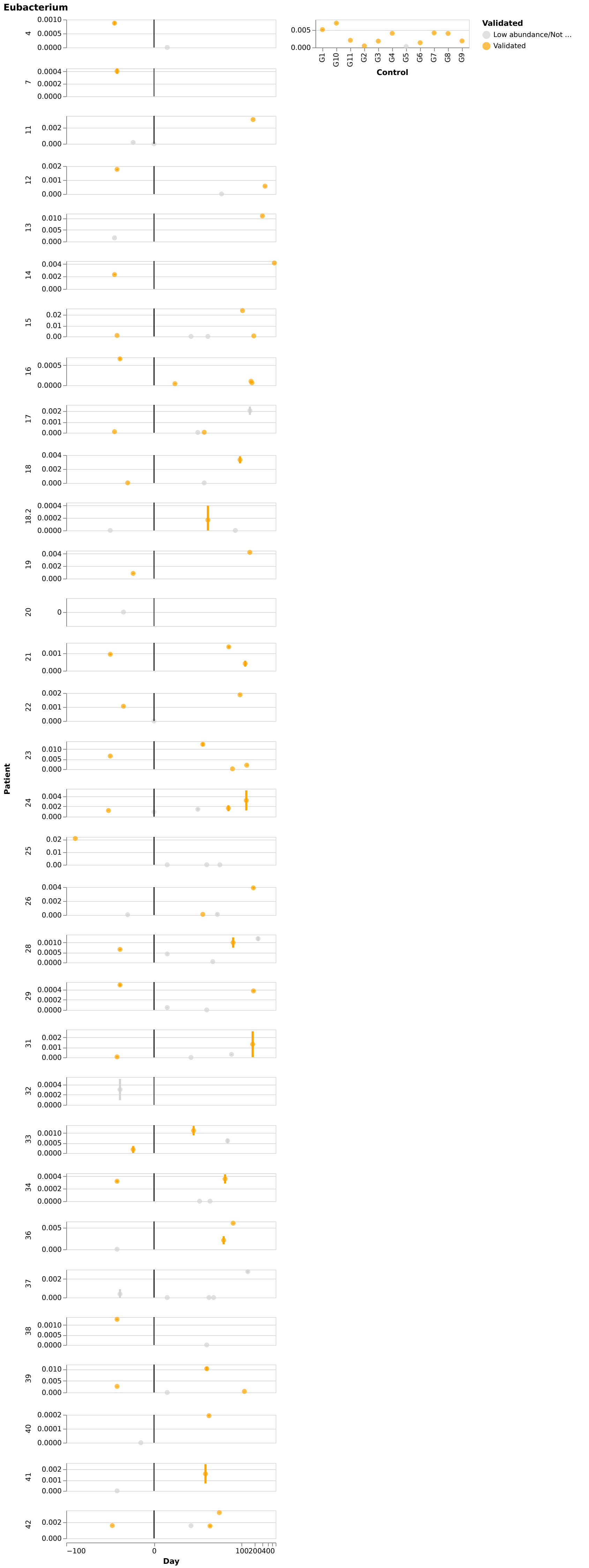

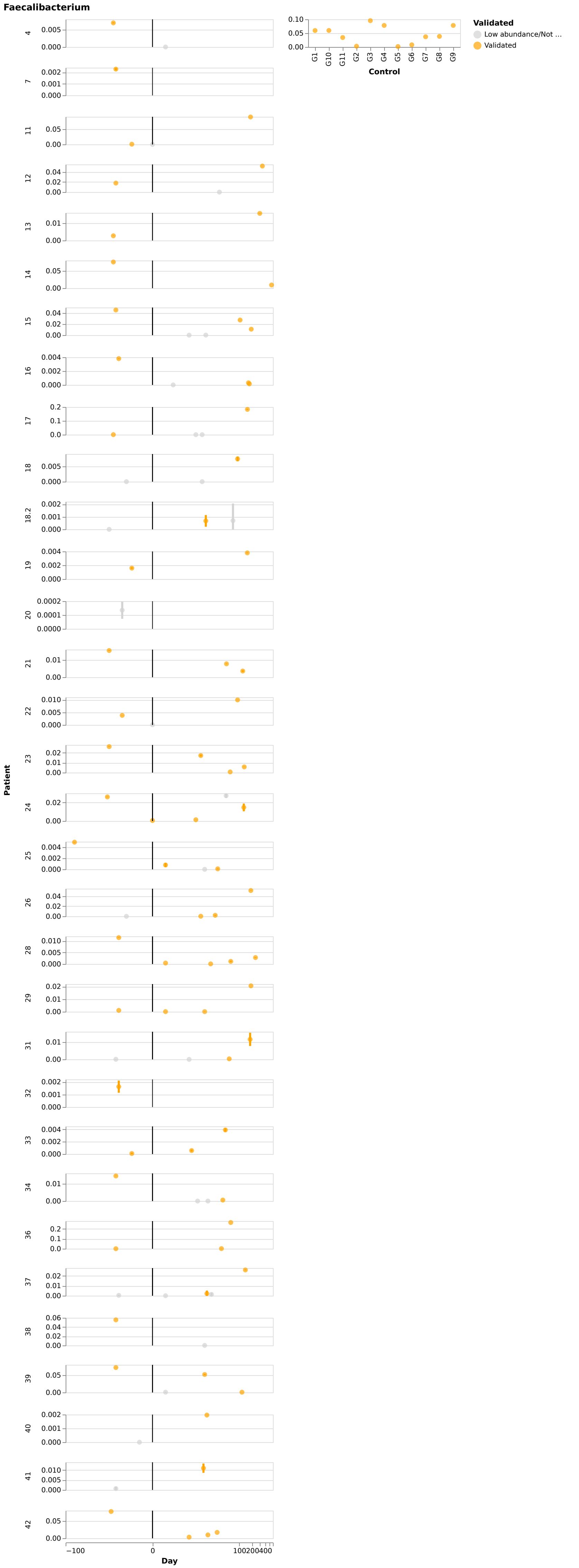

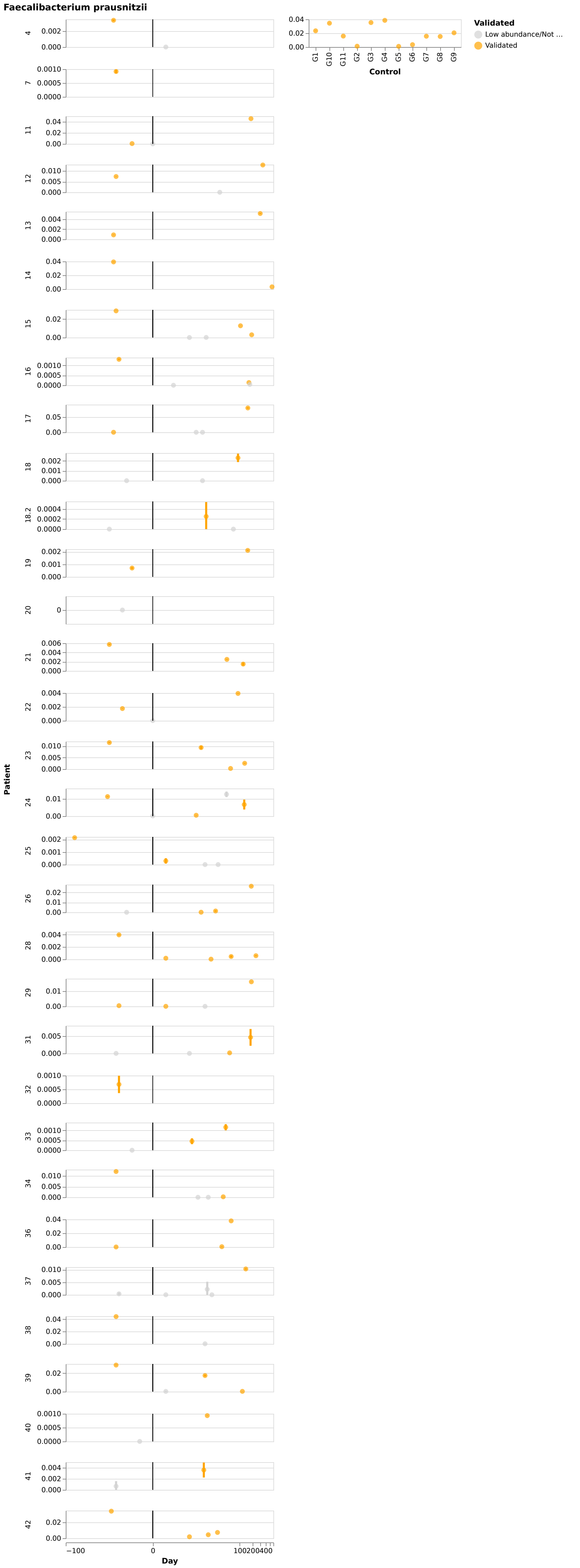

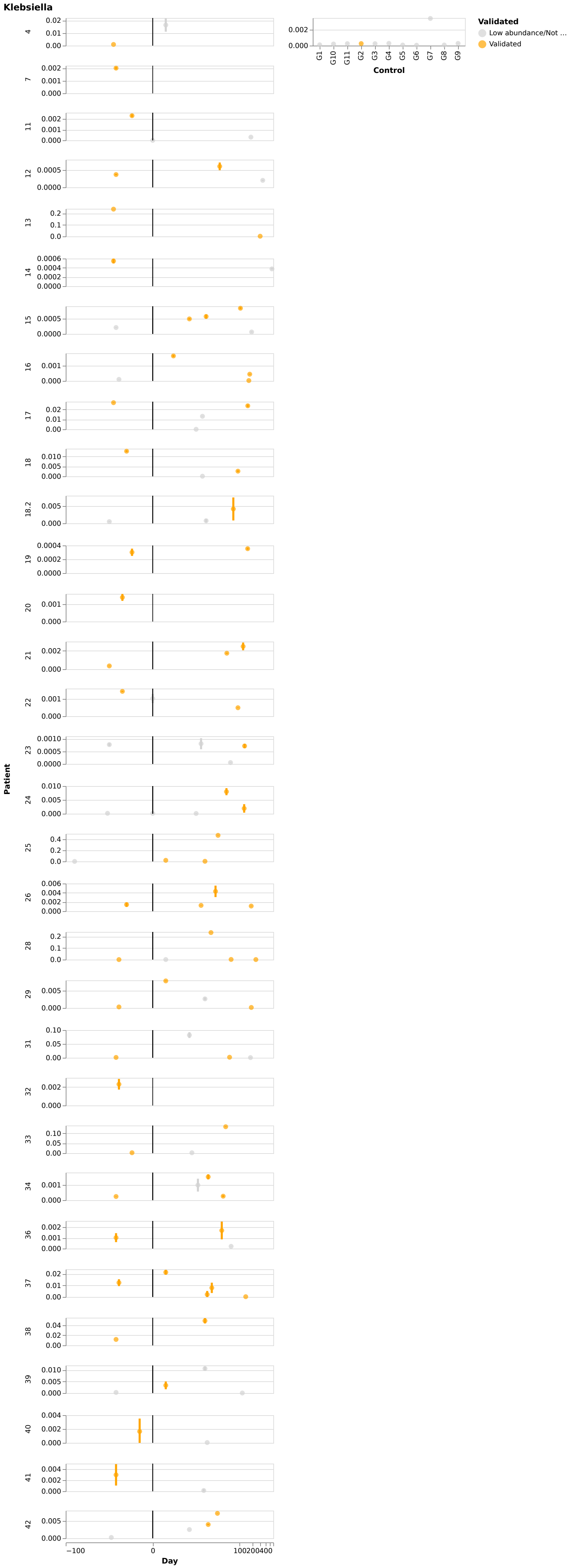

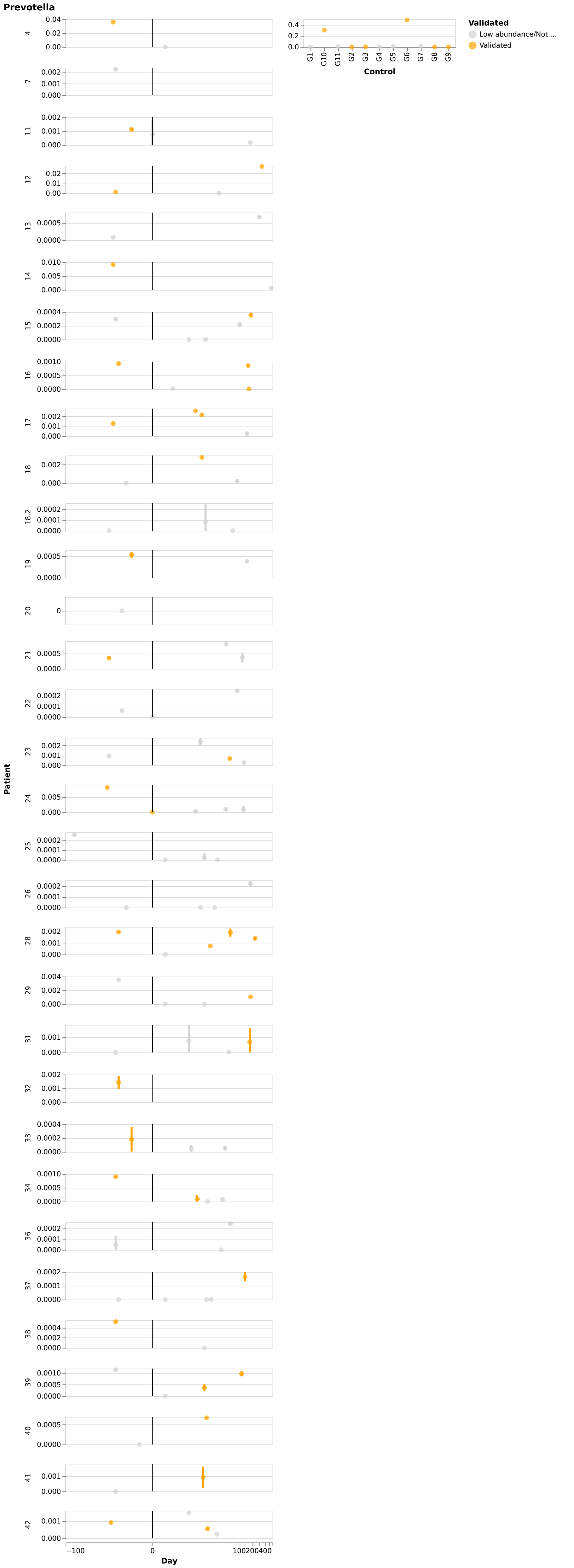

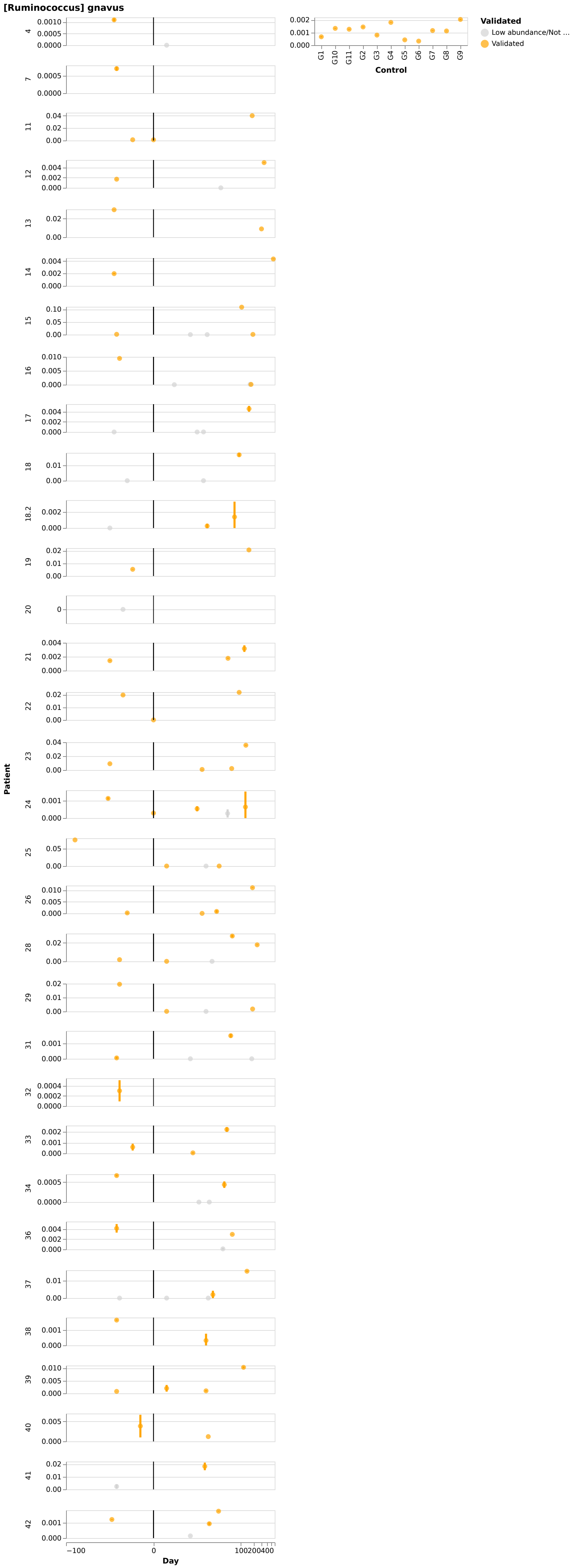

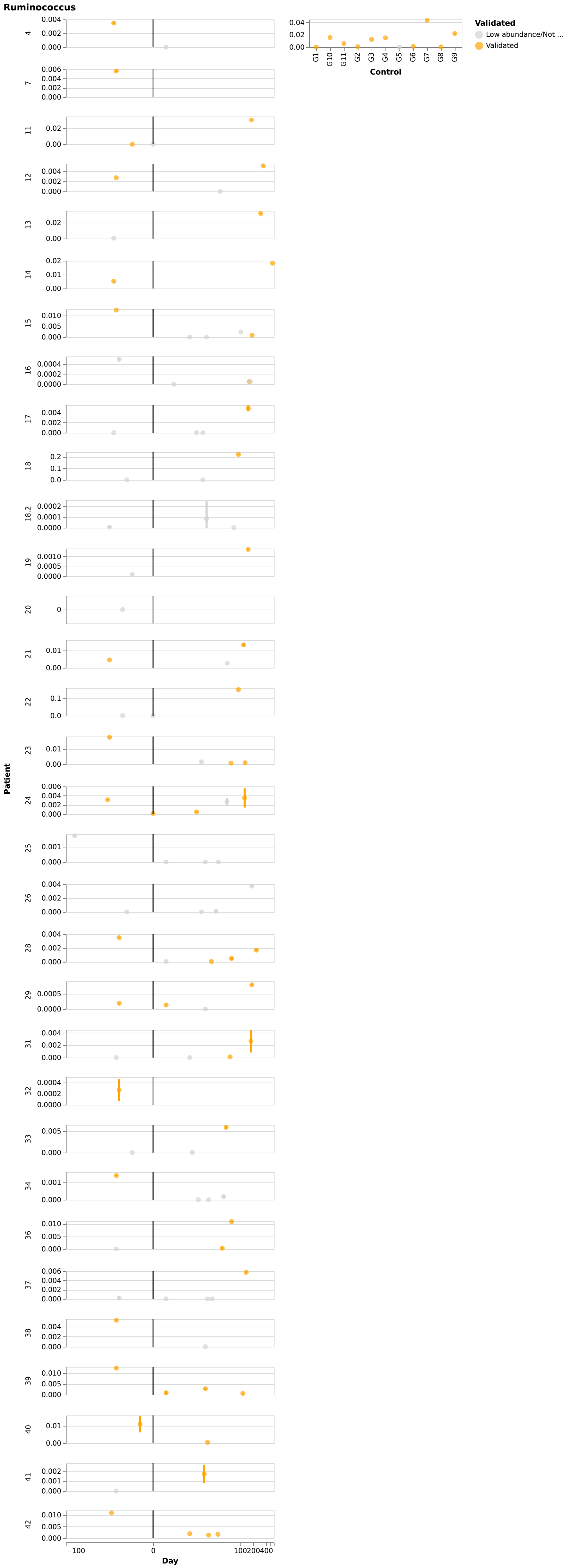

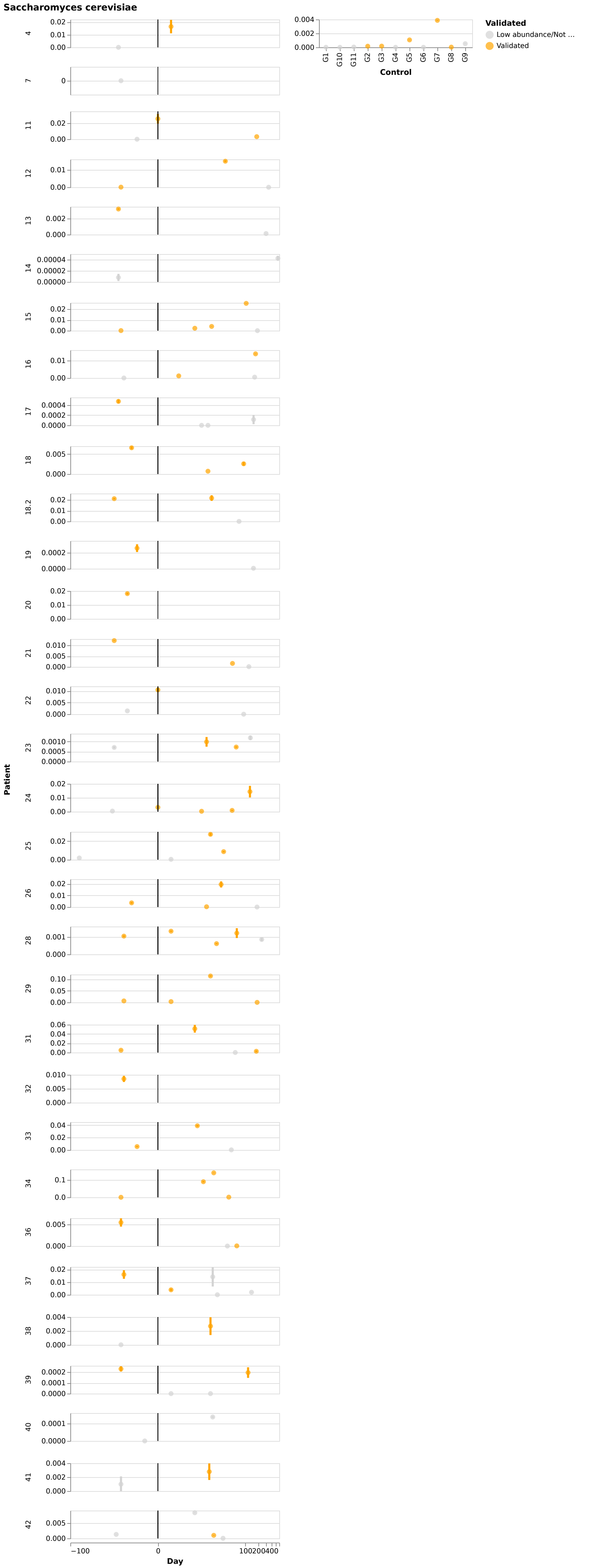

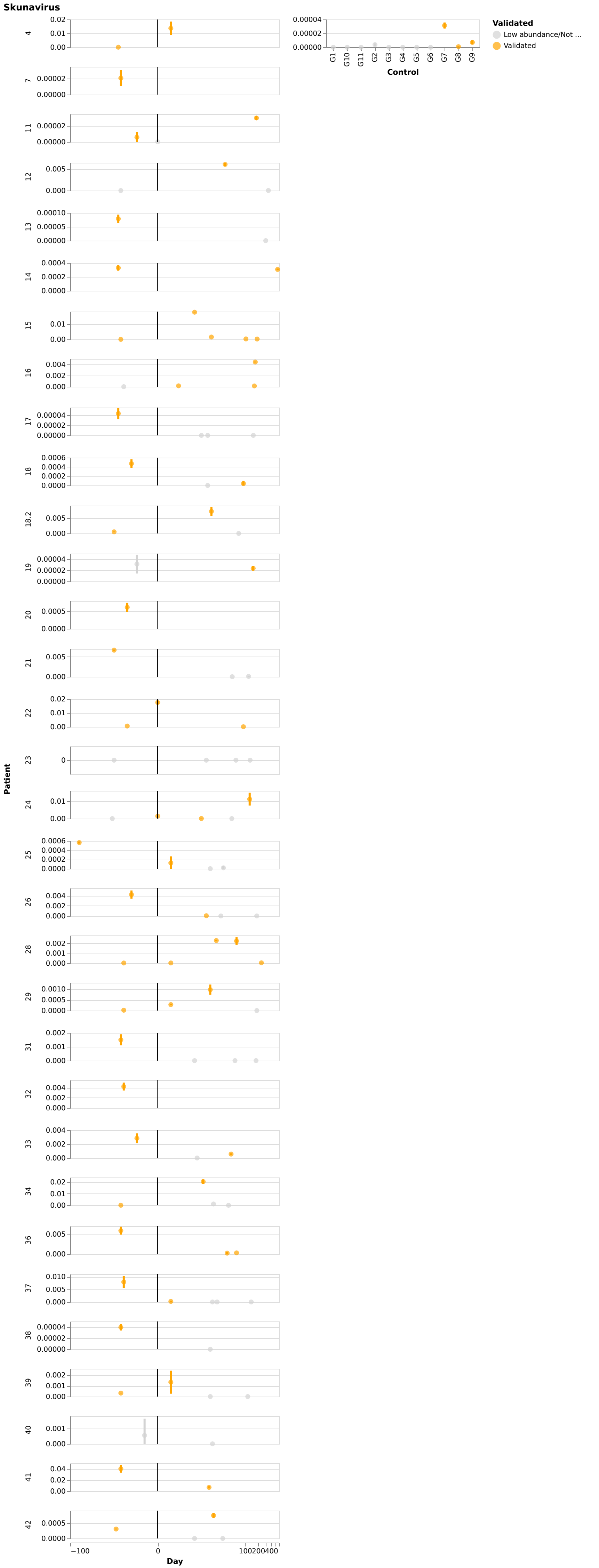

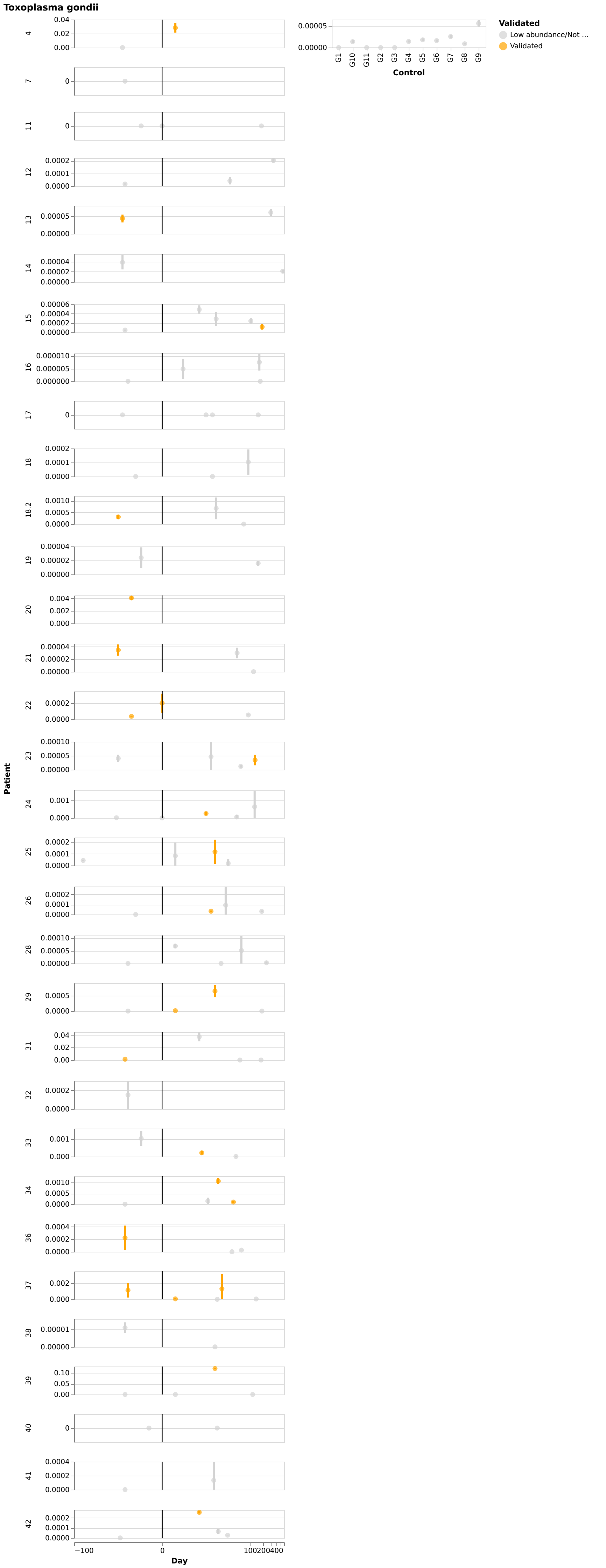

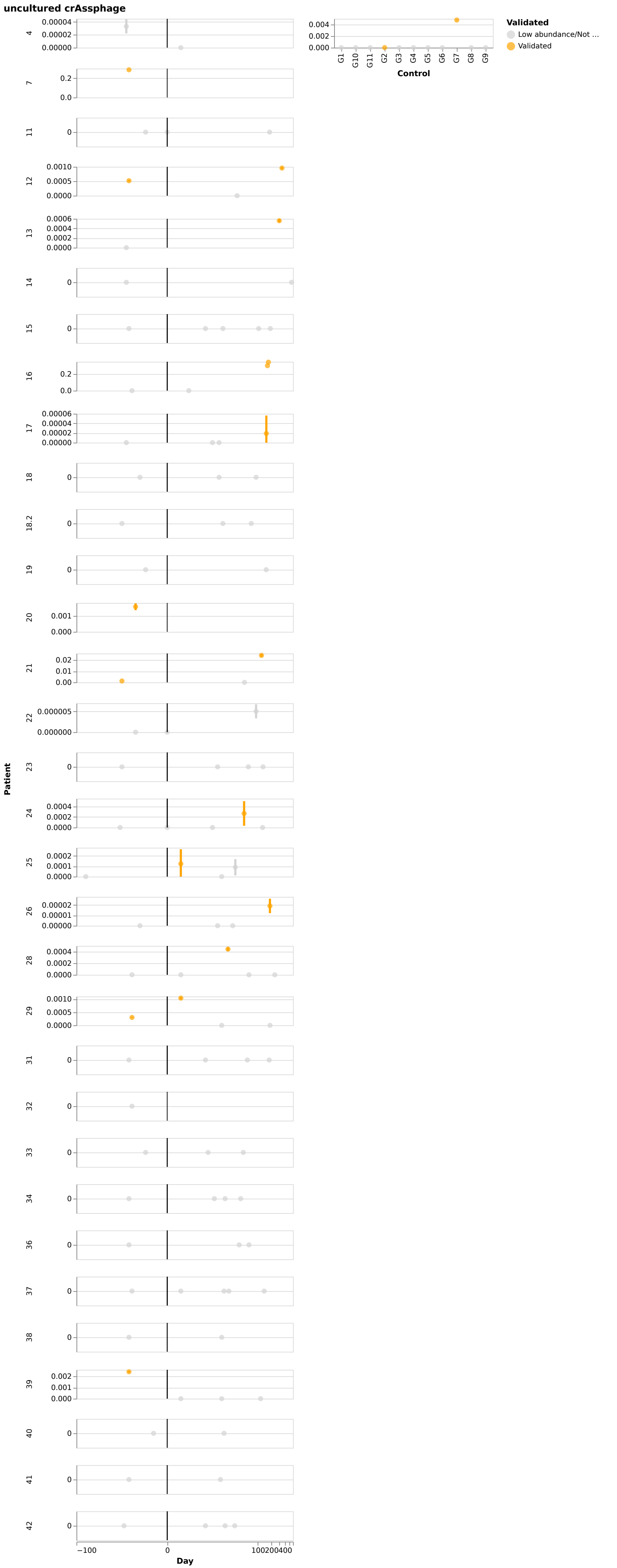

Supplement: Supplementary file 7 — Supplementary Figure 7. [file 41598_2024_53506_MOESM7_ESM.pdf]

11\_182

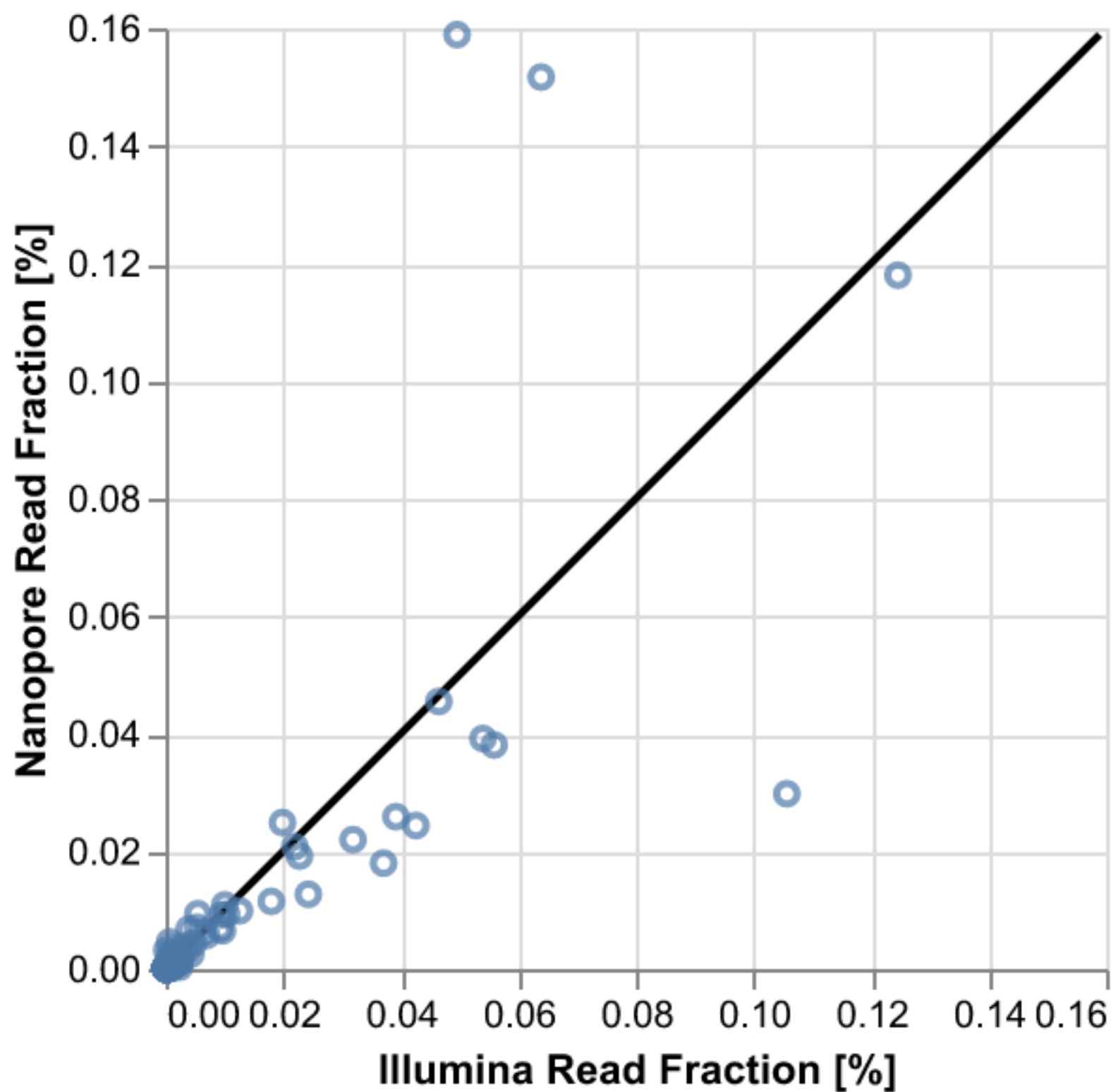

11\_-2

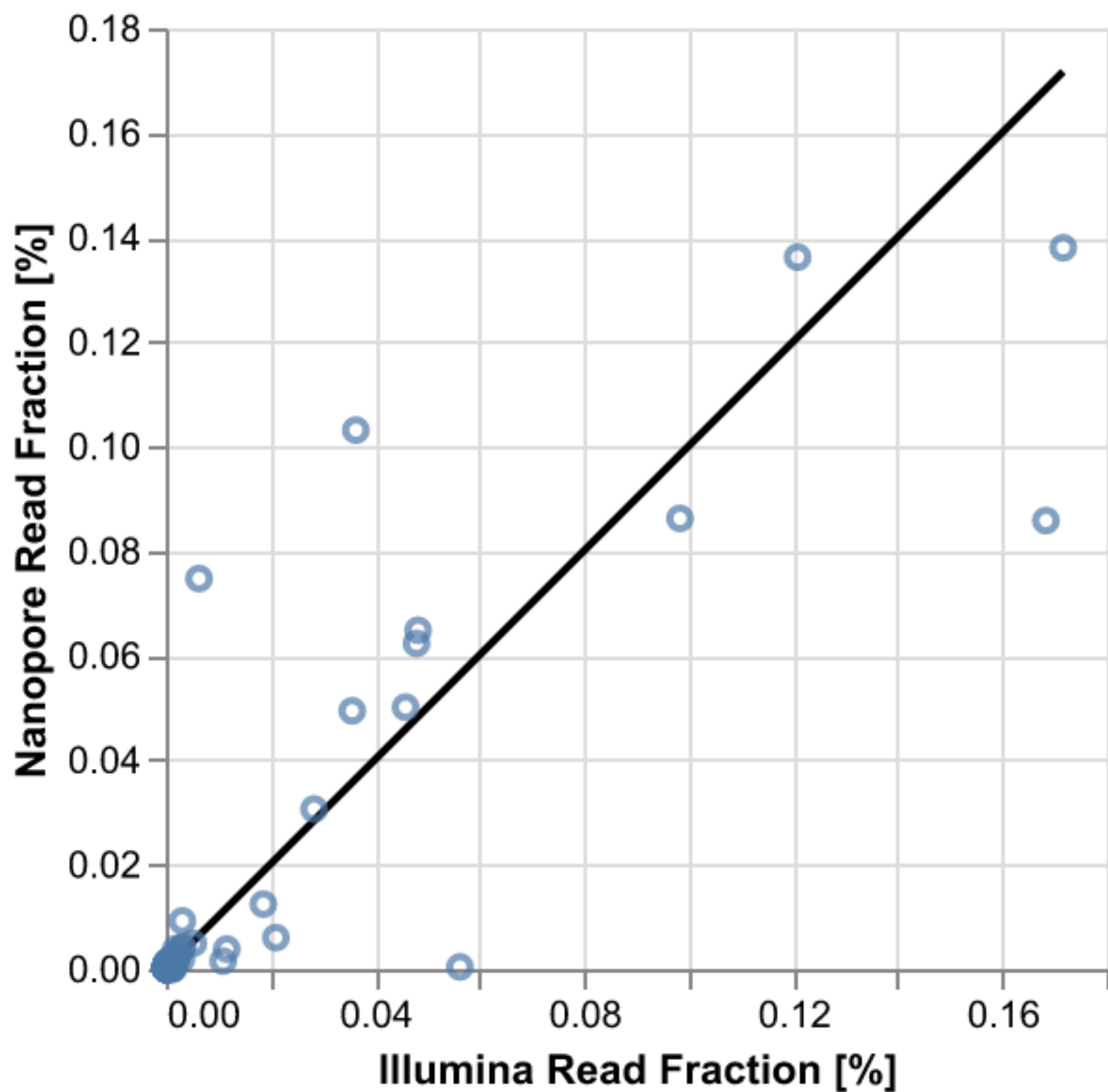

12\_342

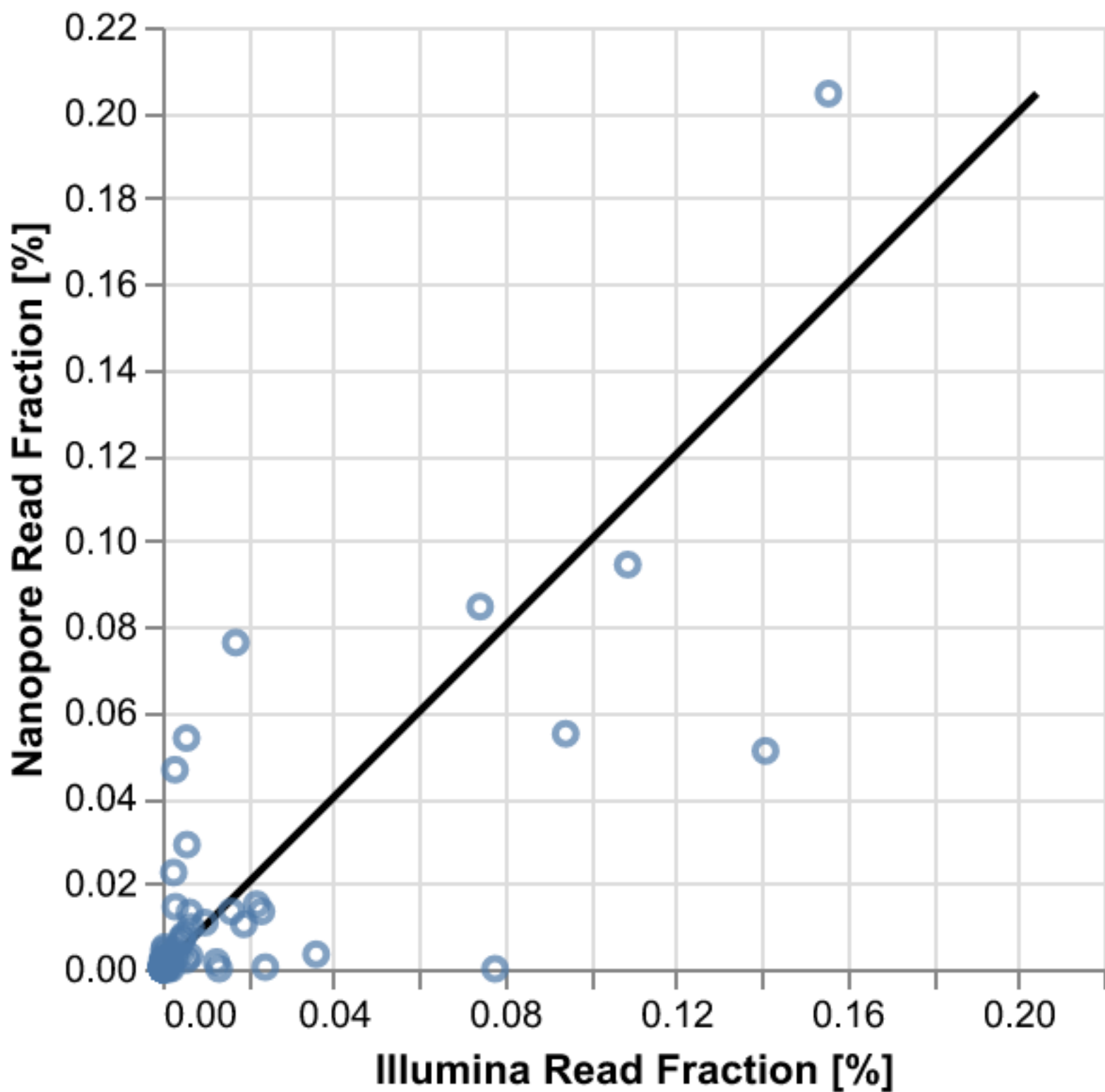

12\_34

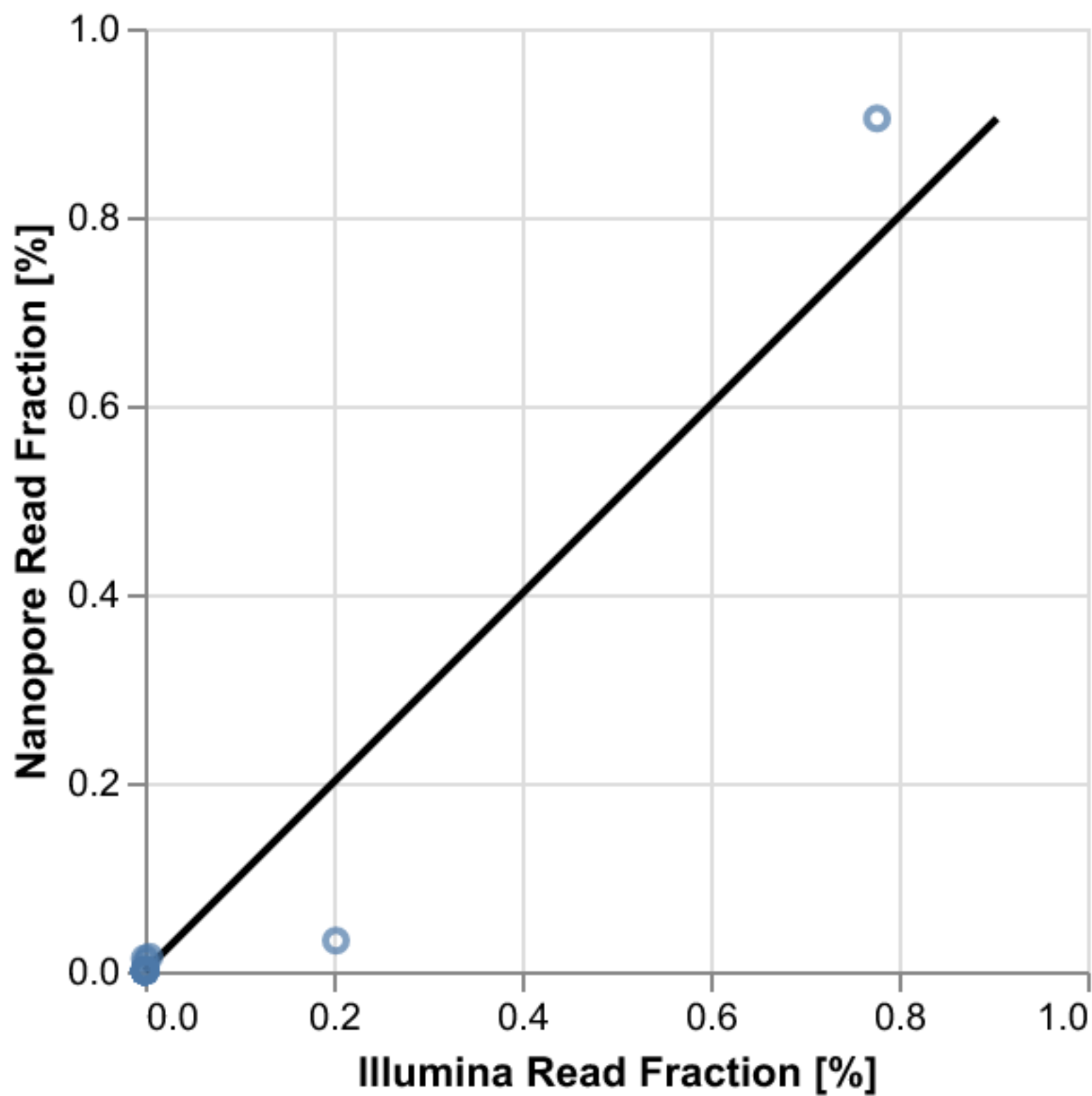

12\_-6

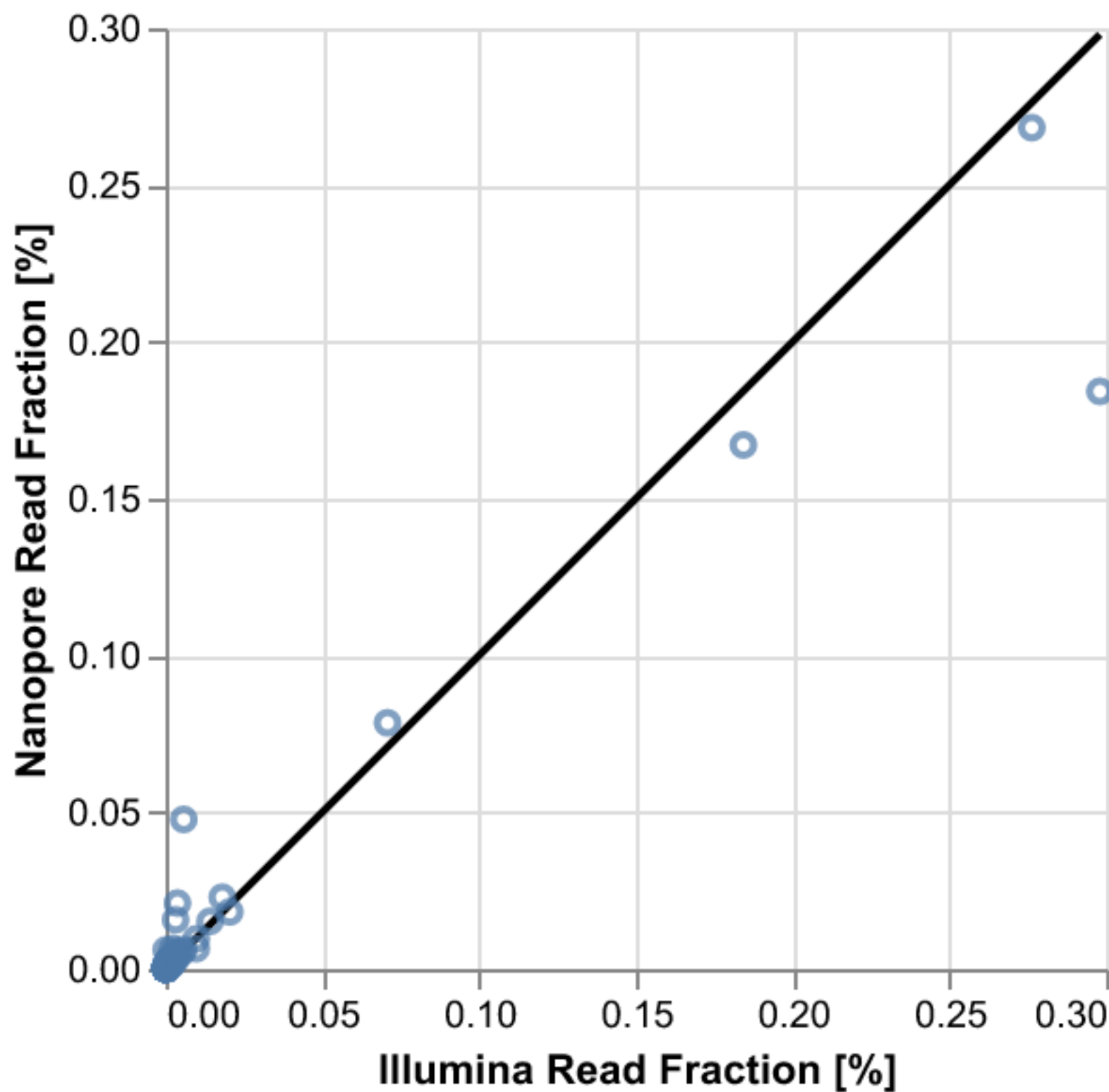

13\_298

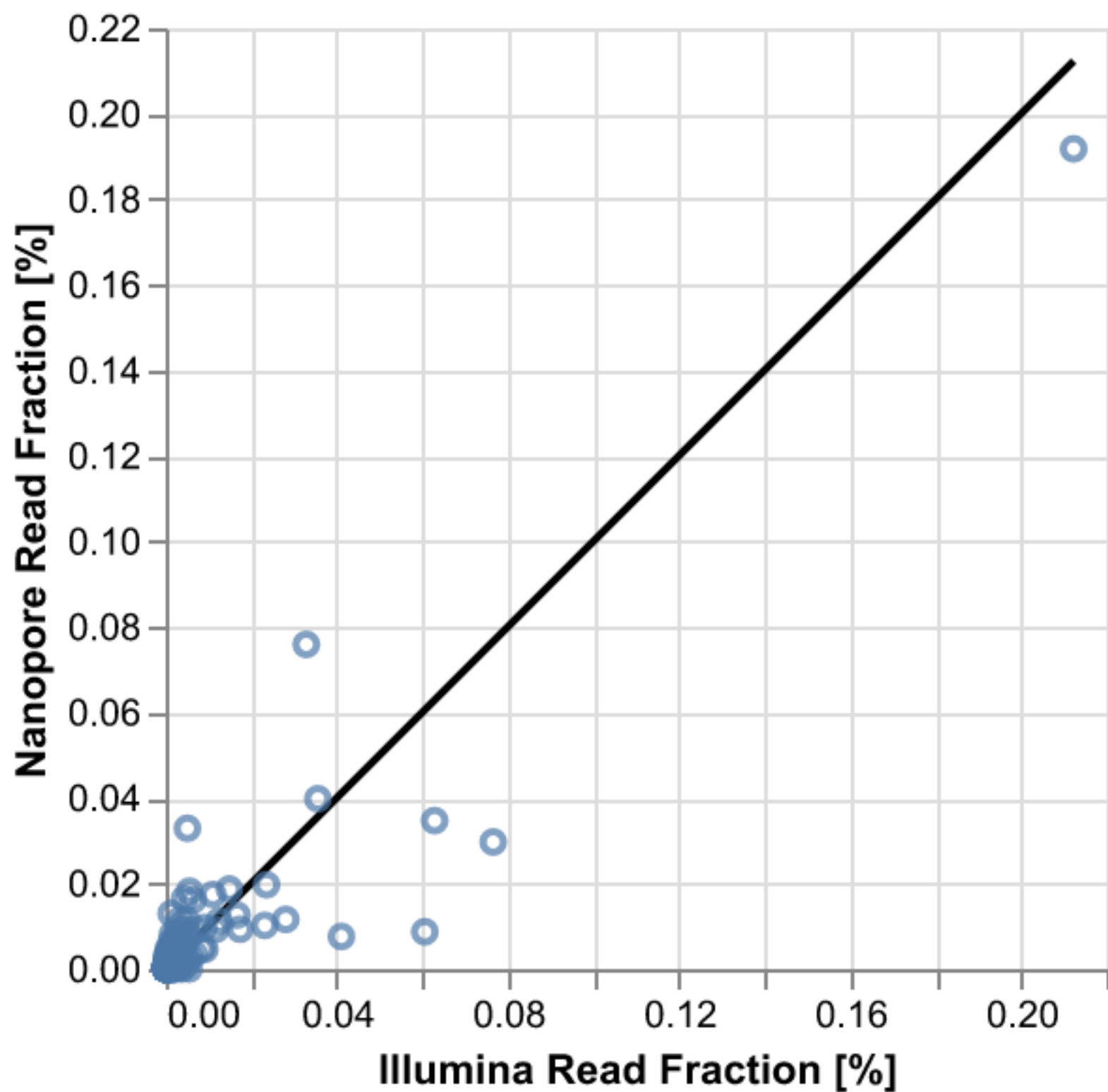

**13\_-7**

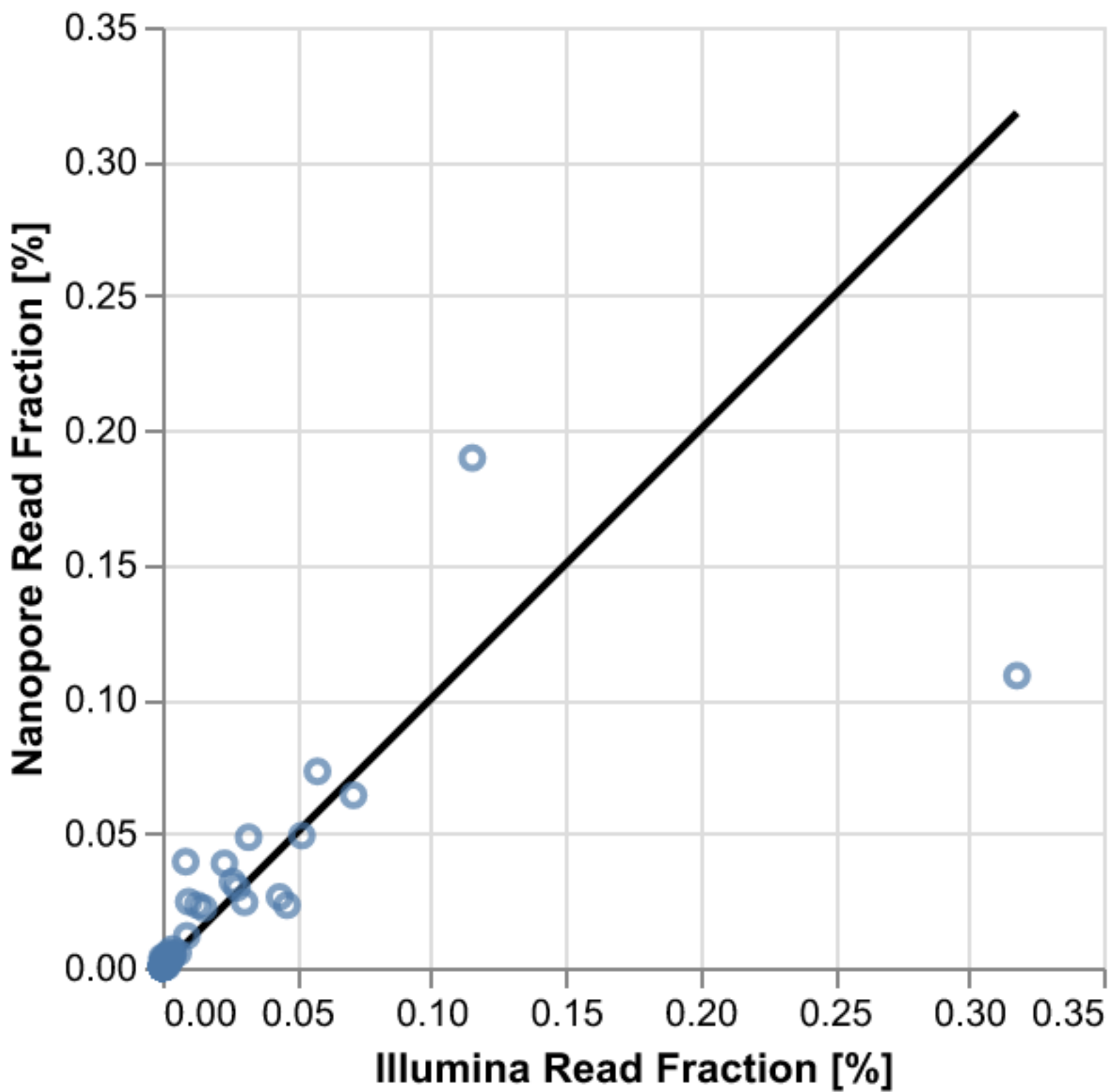

14\_-7

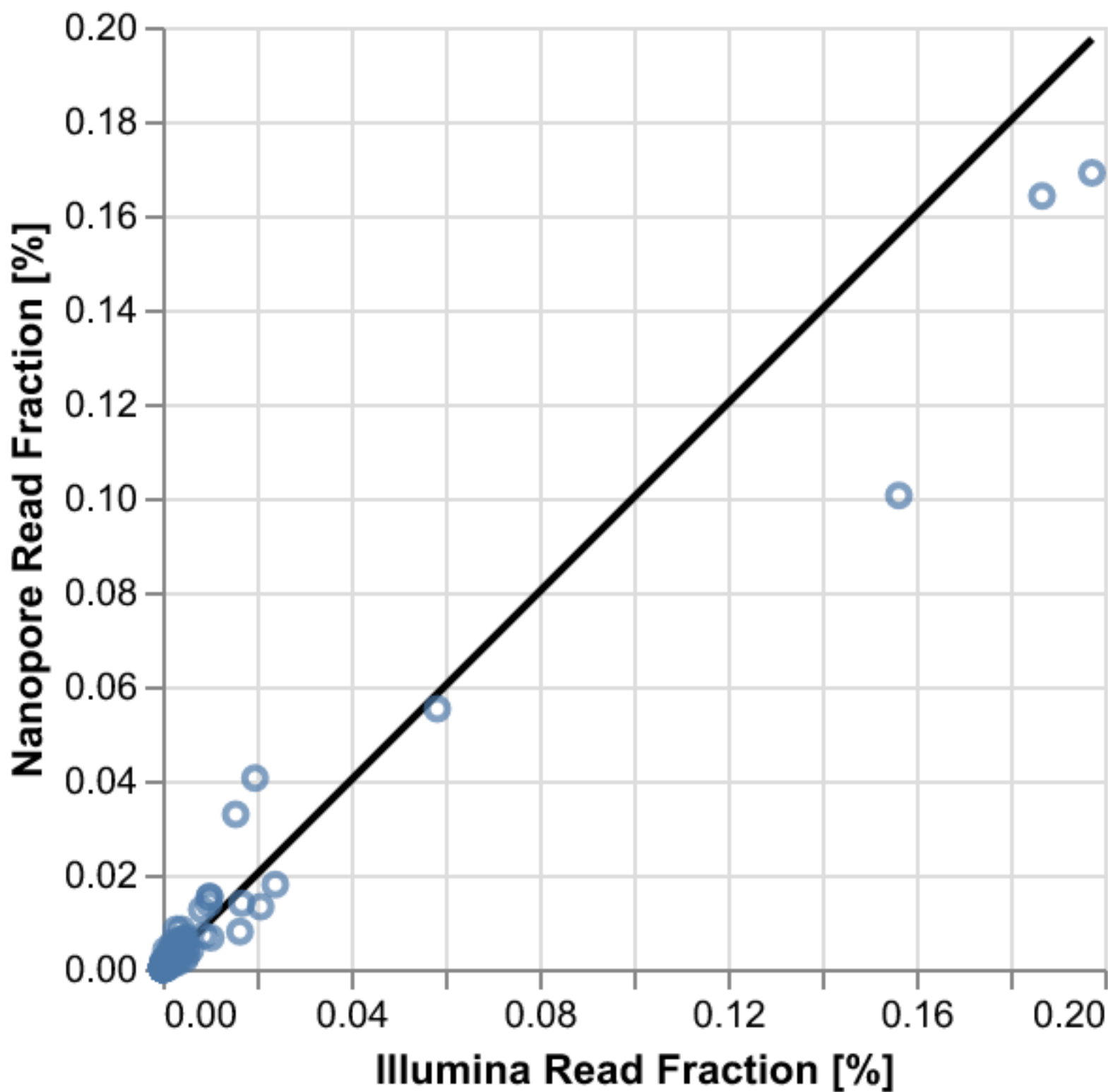

15\_104

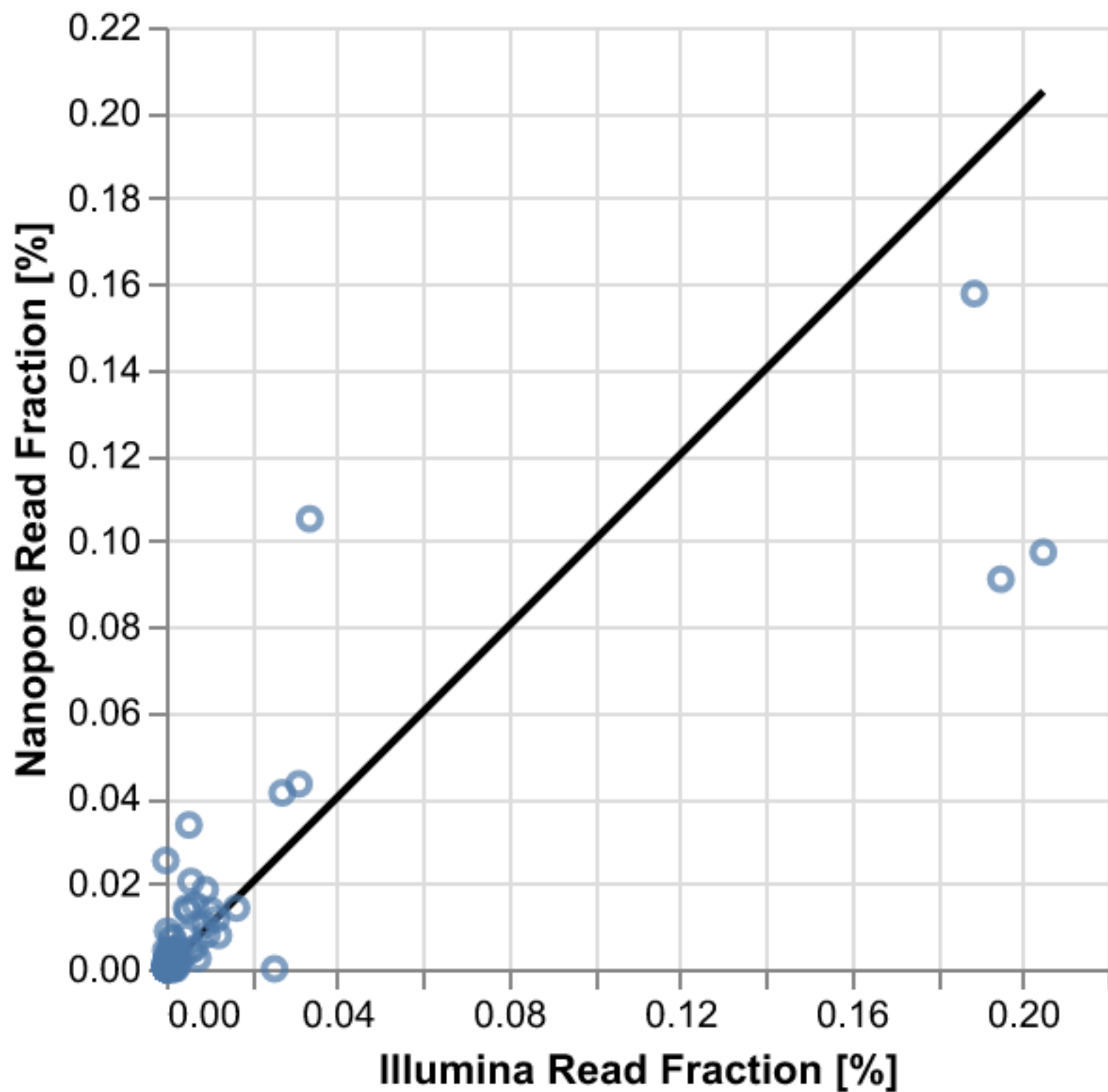

15\_16

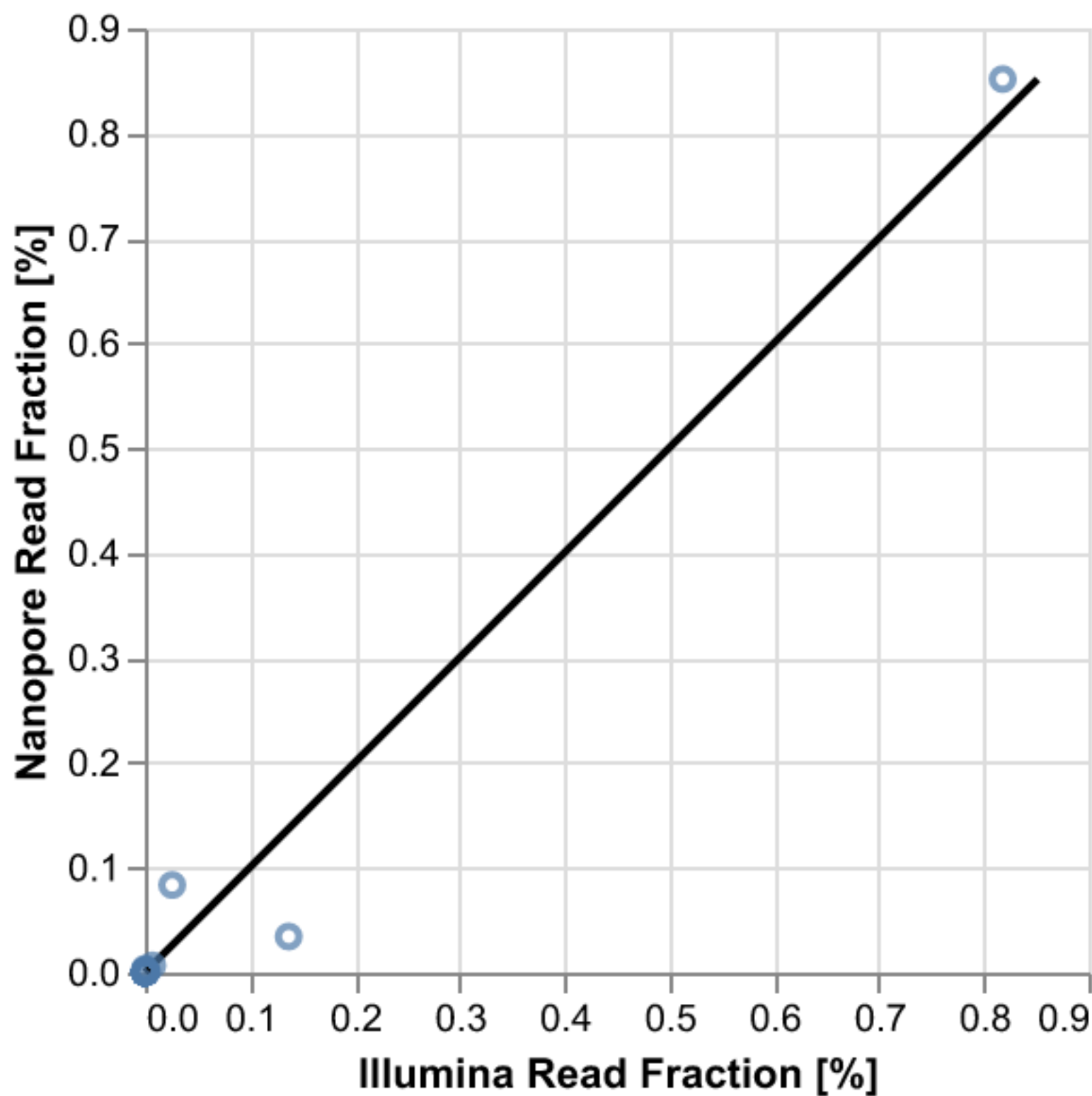

**15\_189**

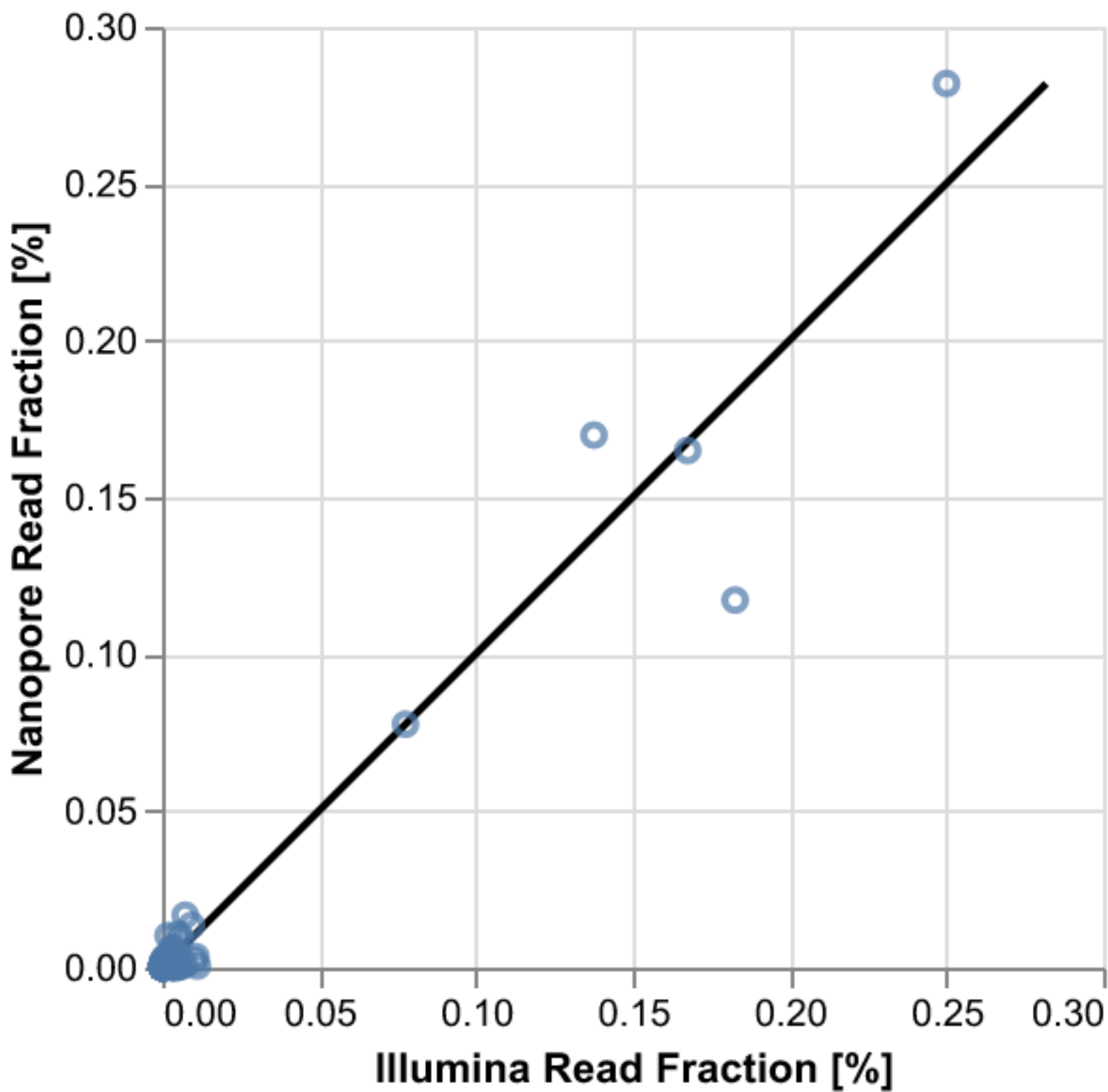

15\_-6

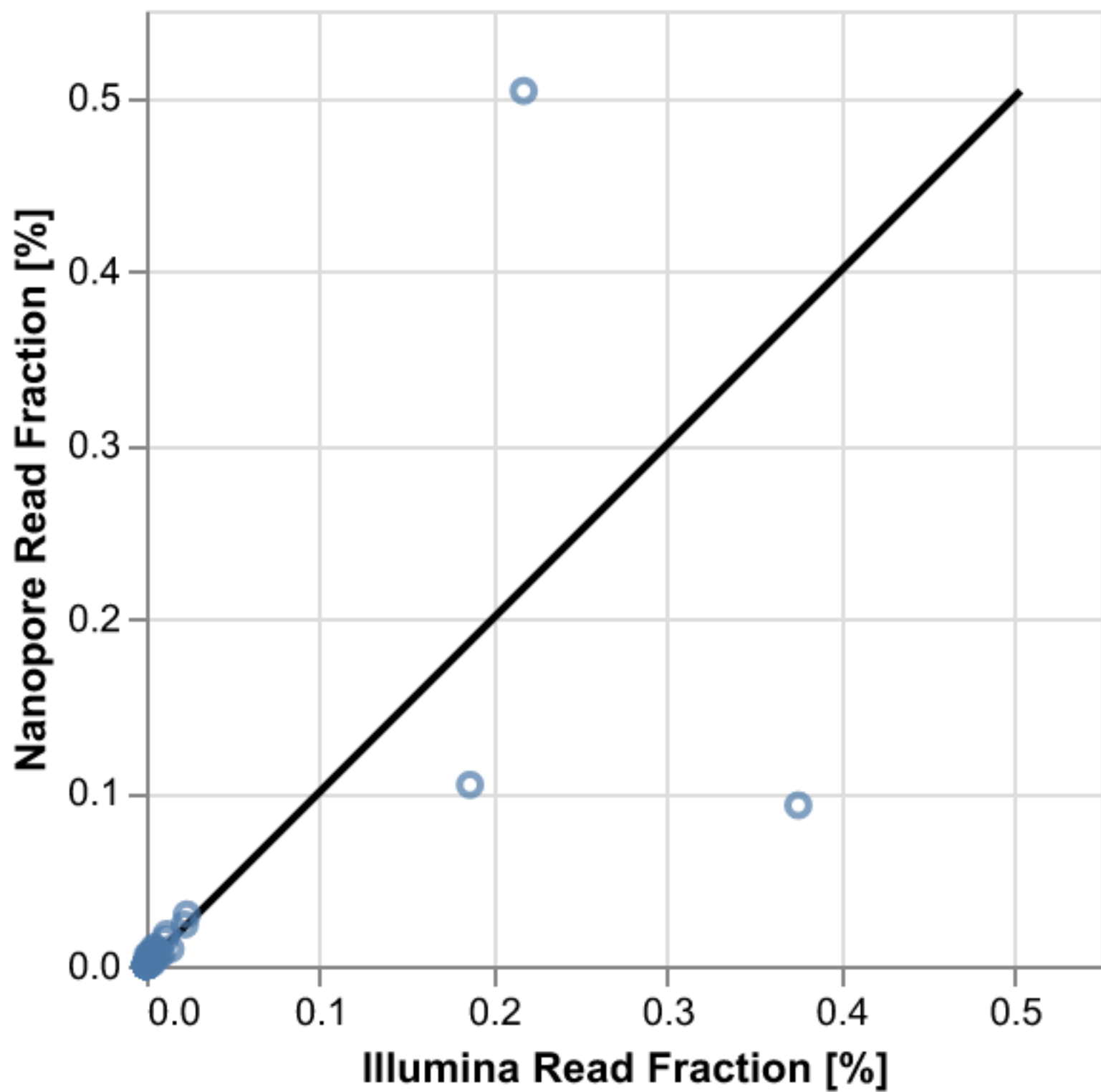

15\_6

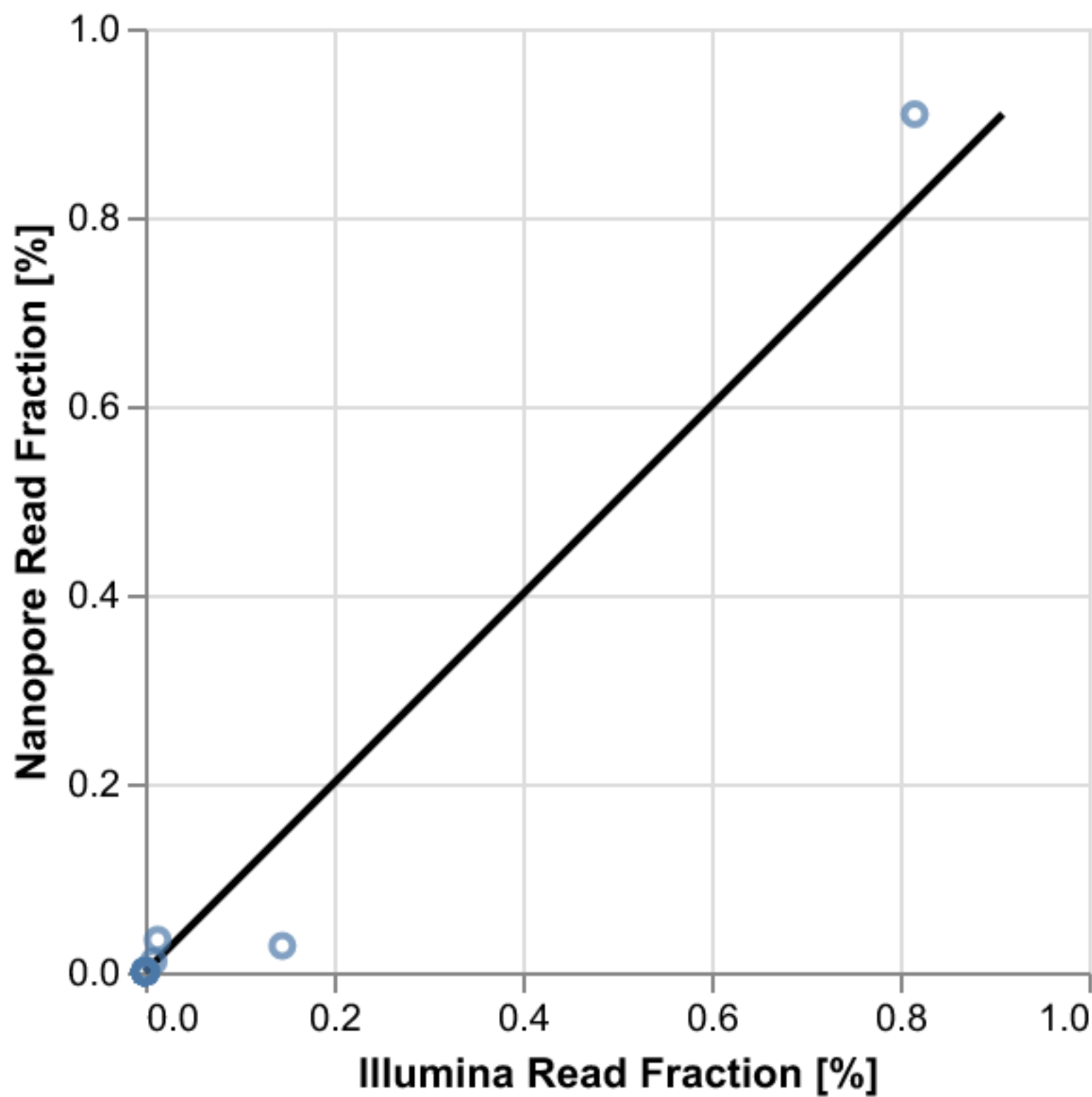

**16\_163**

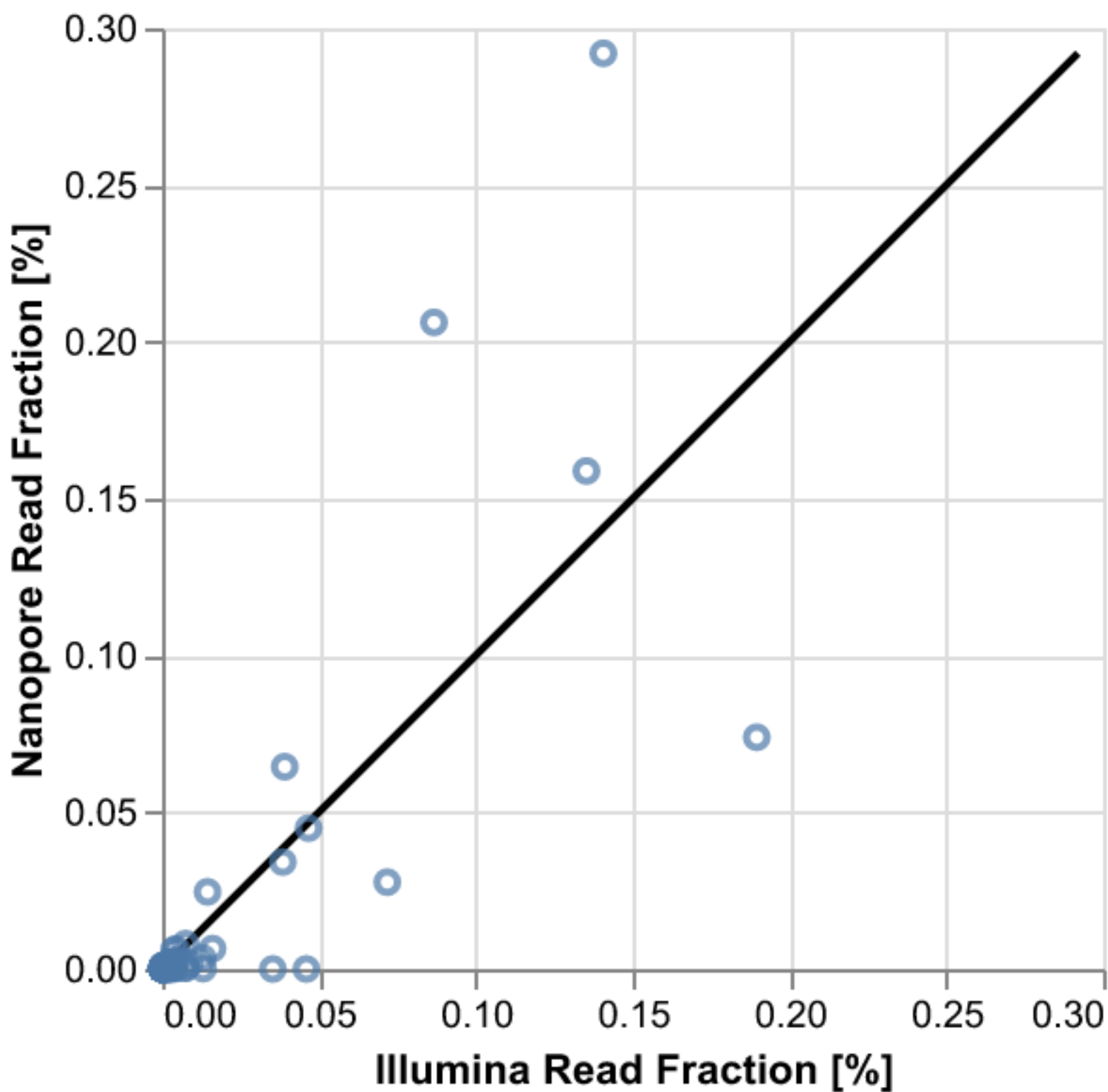

16\_171

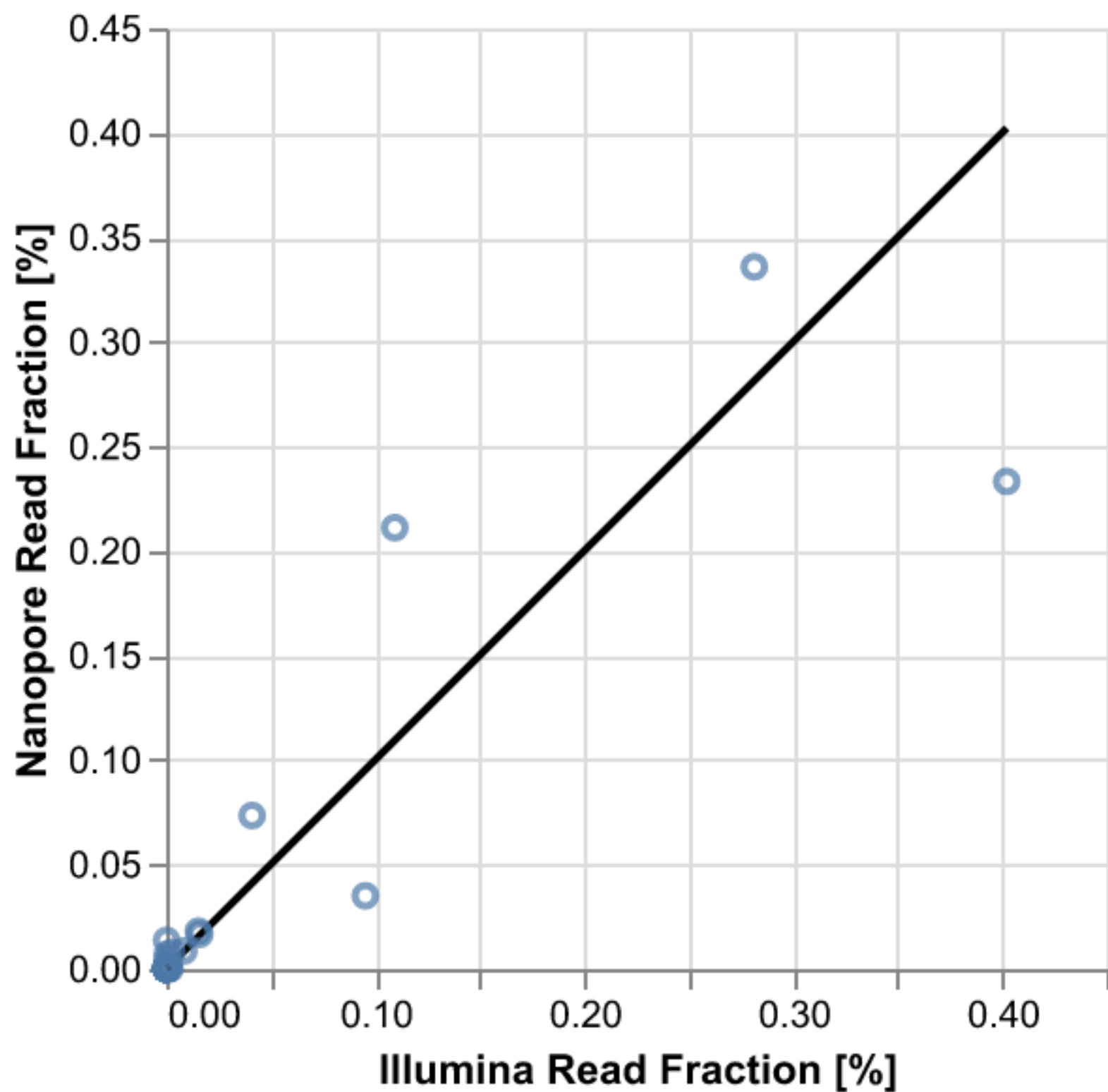

16\_2

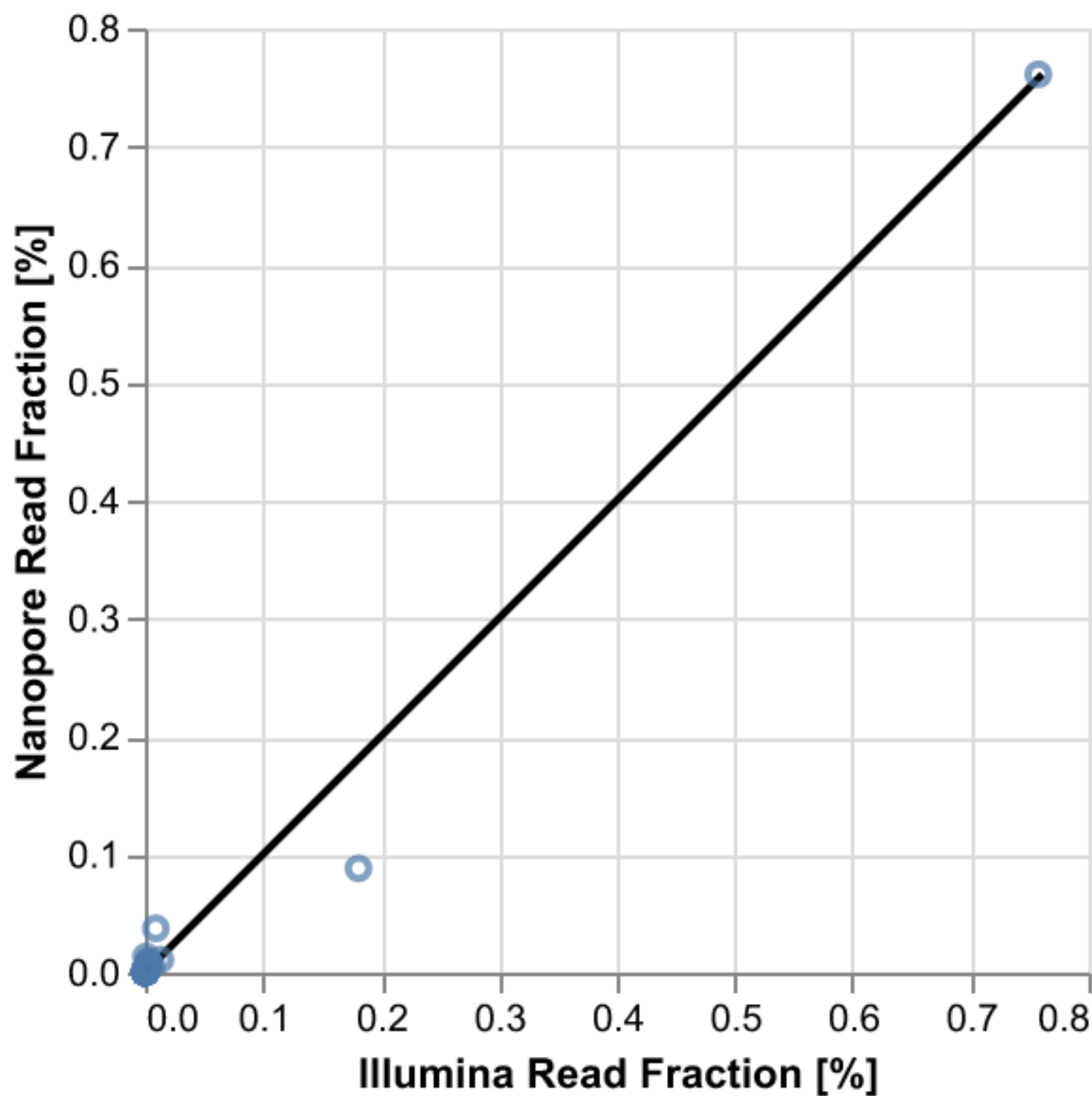

16\_-5

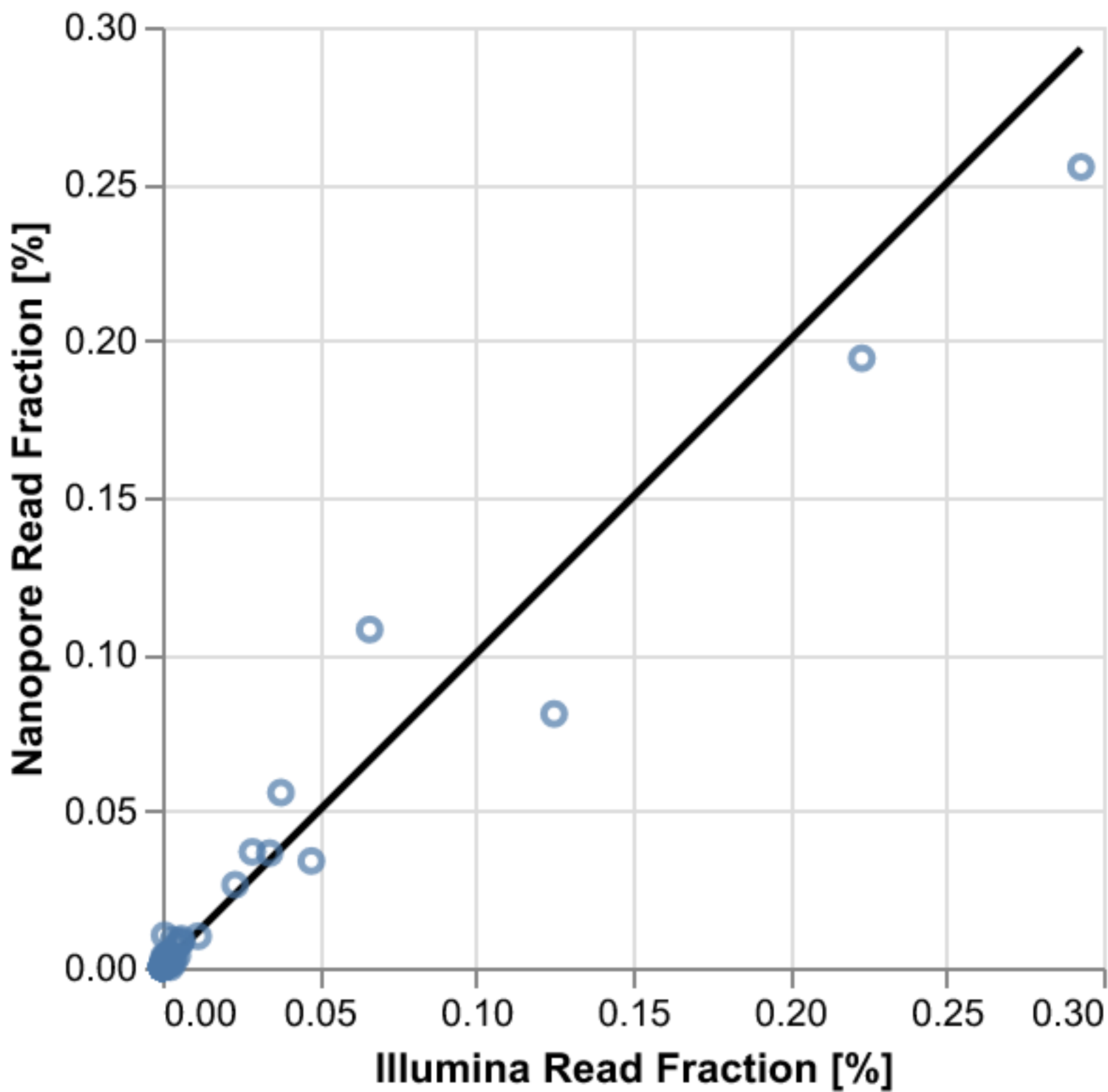

17\_13

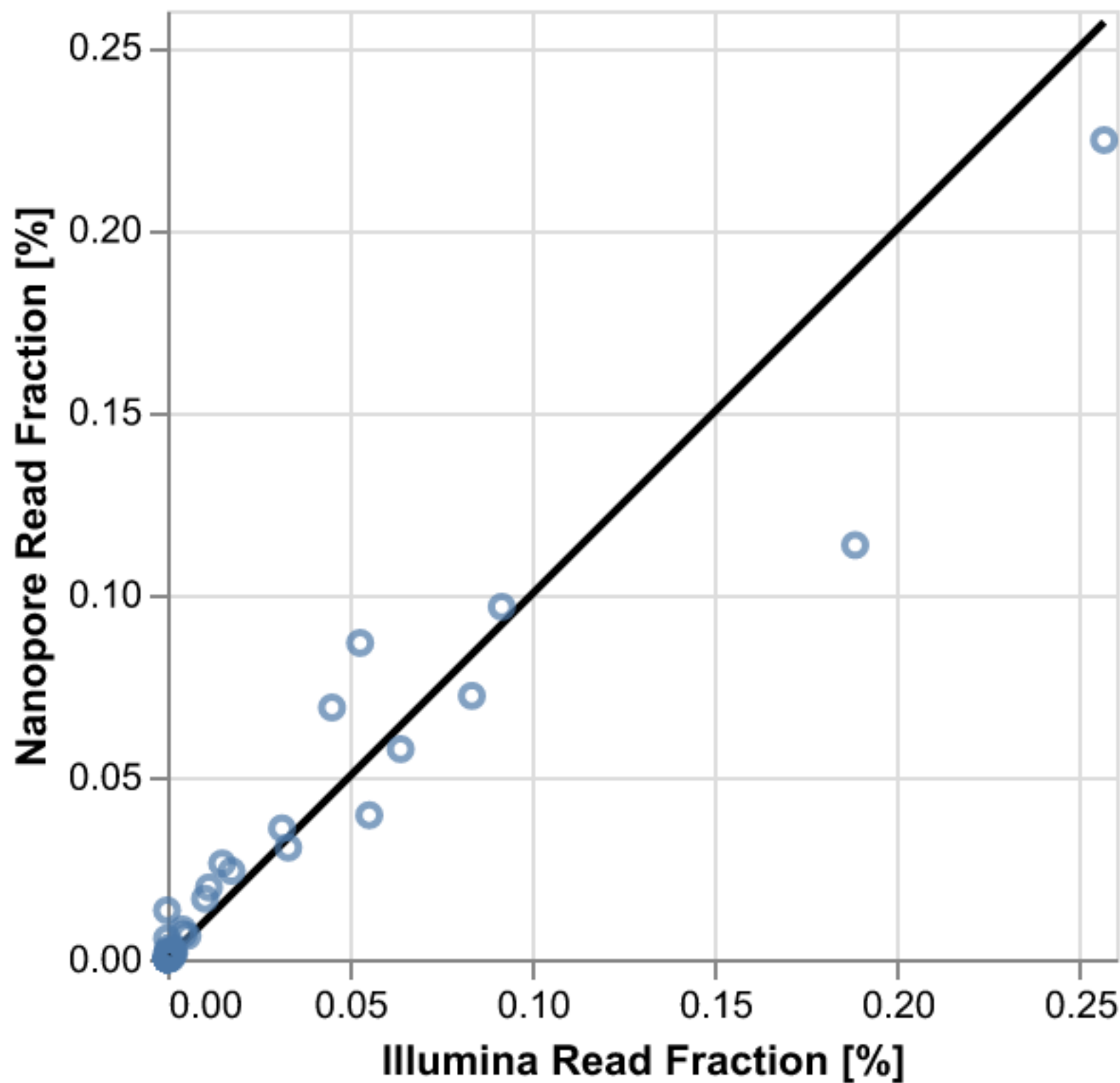

17\_-1

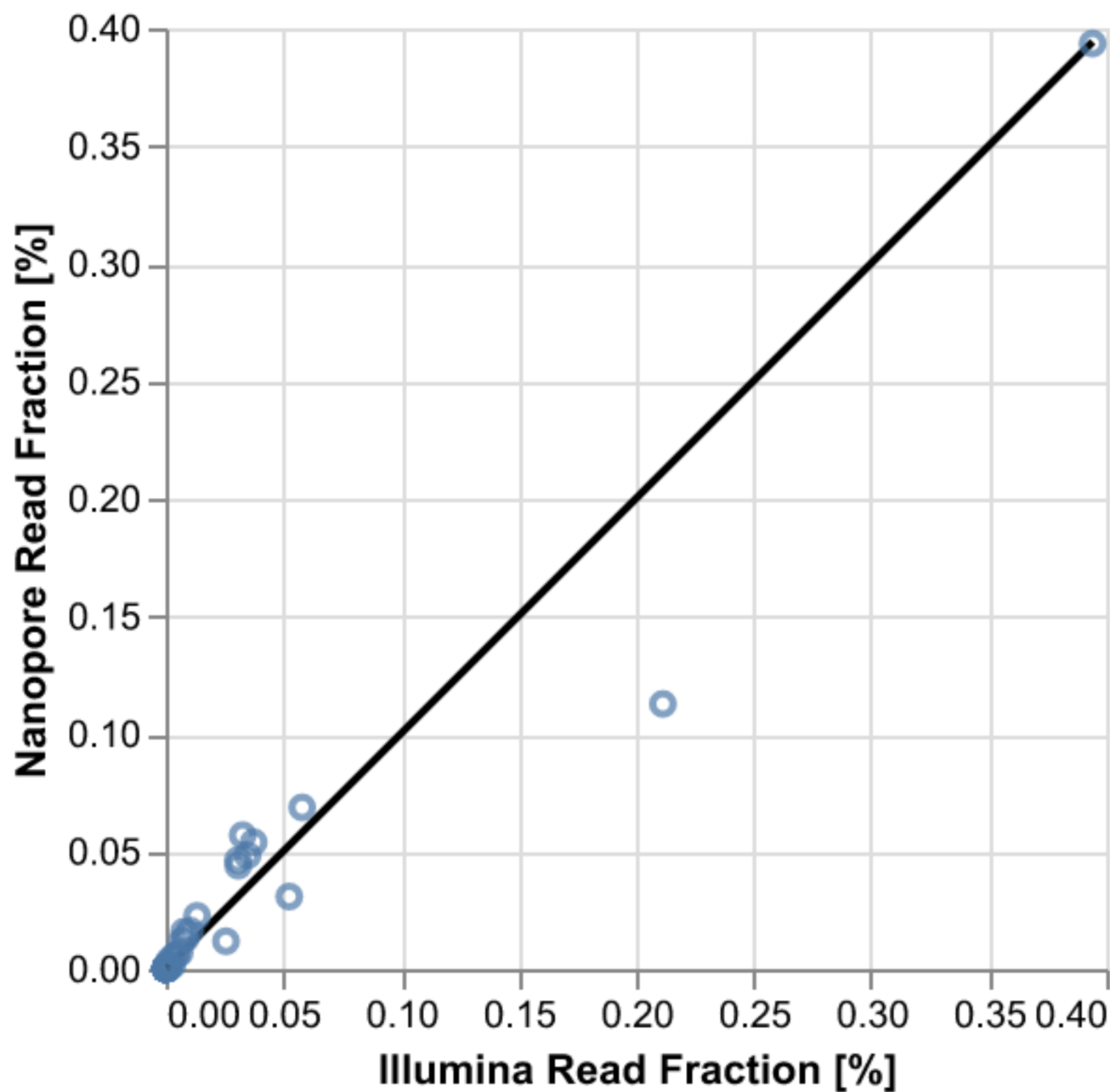

17.2\_154

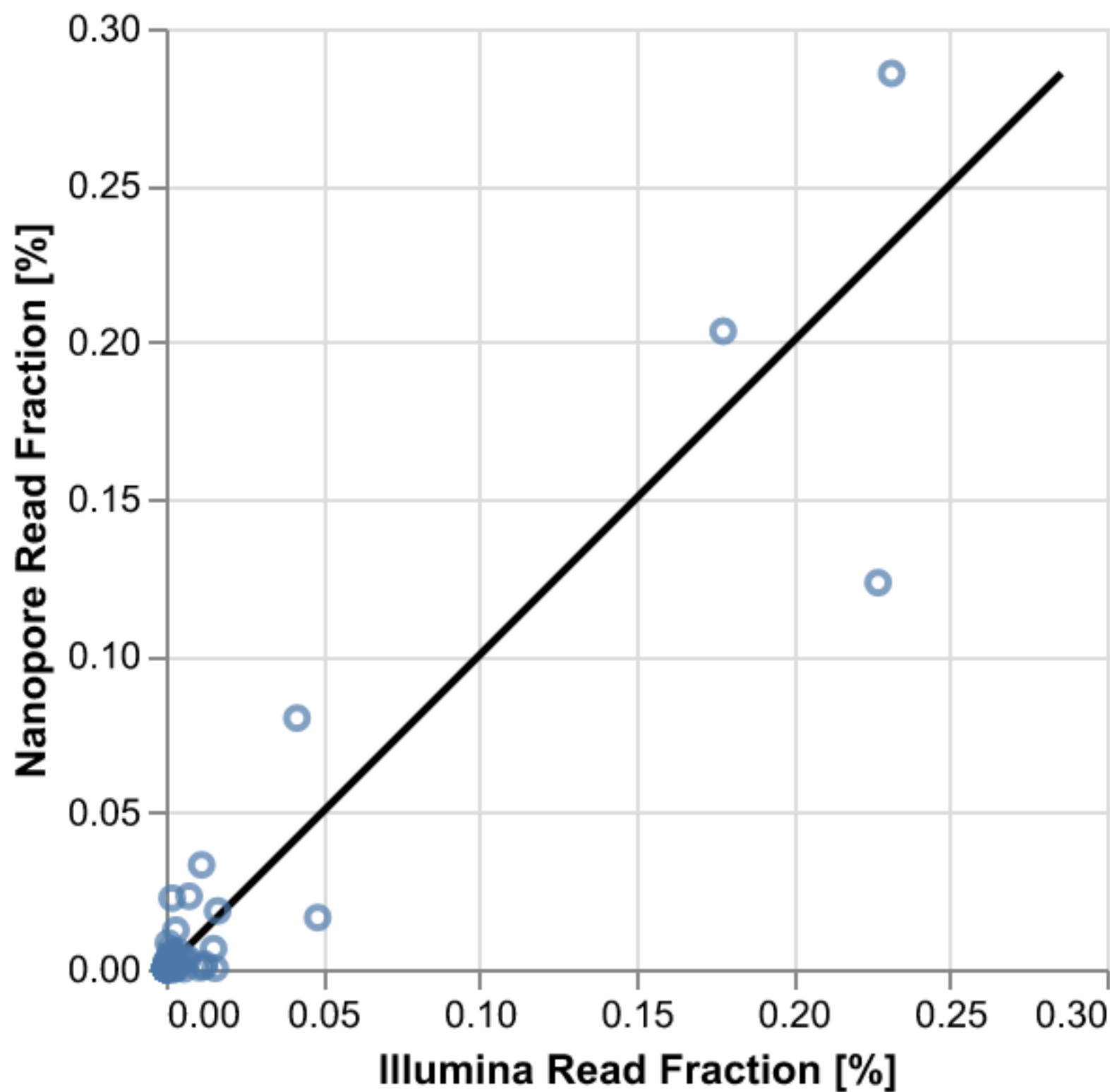

17\_-7

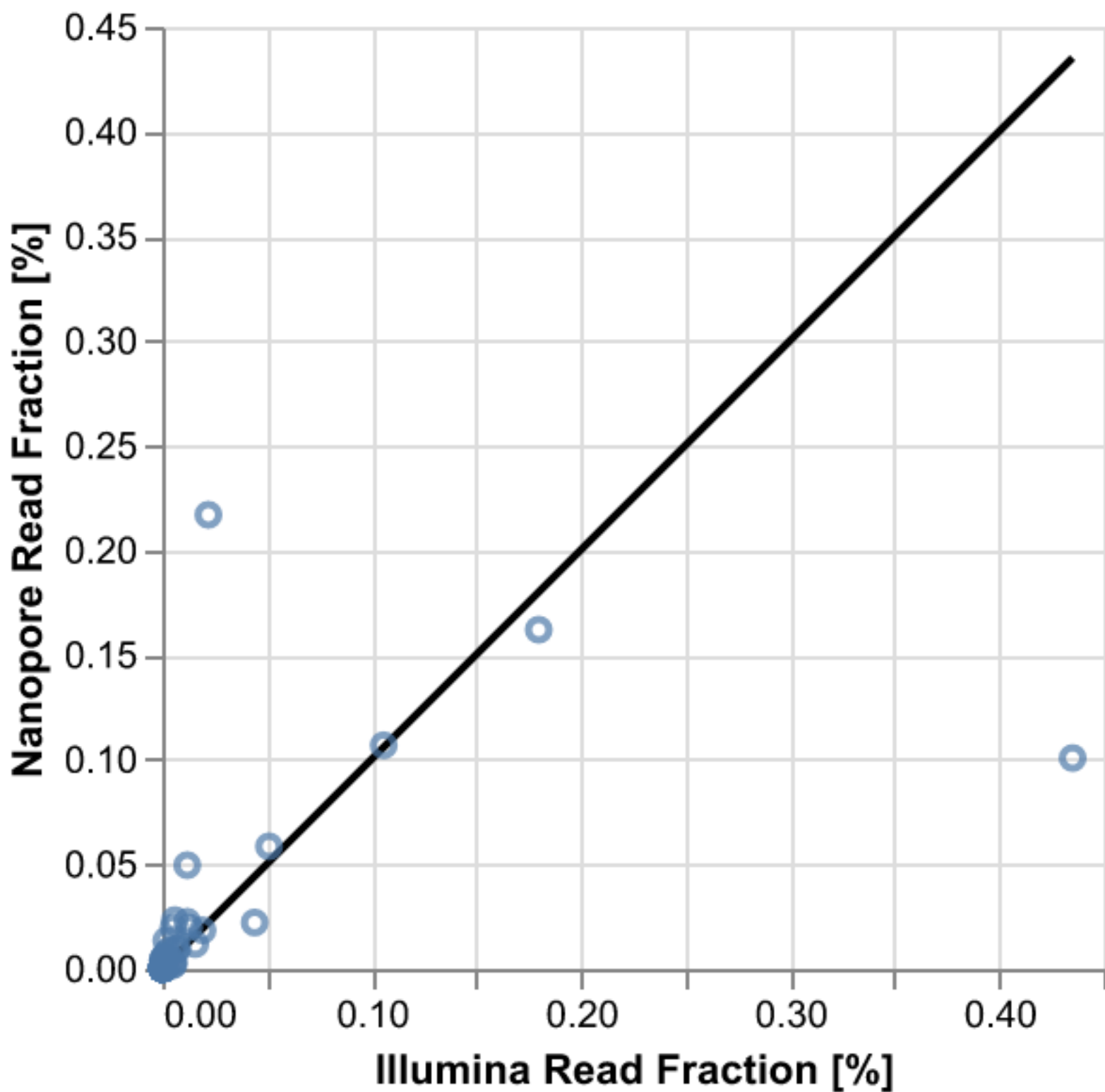

**17\_9**

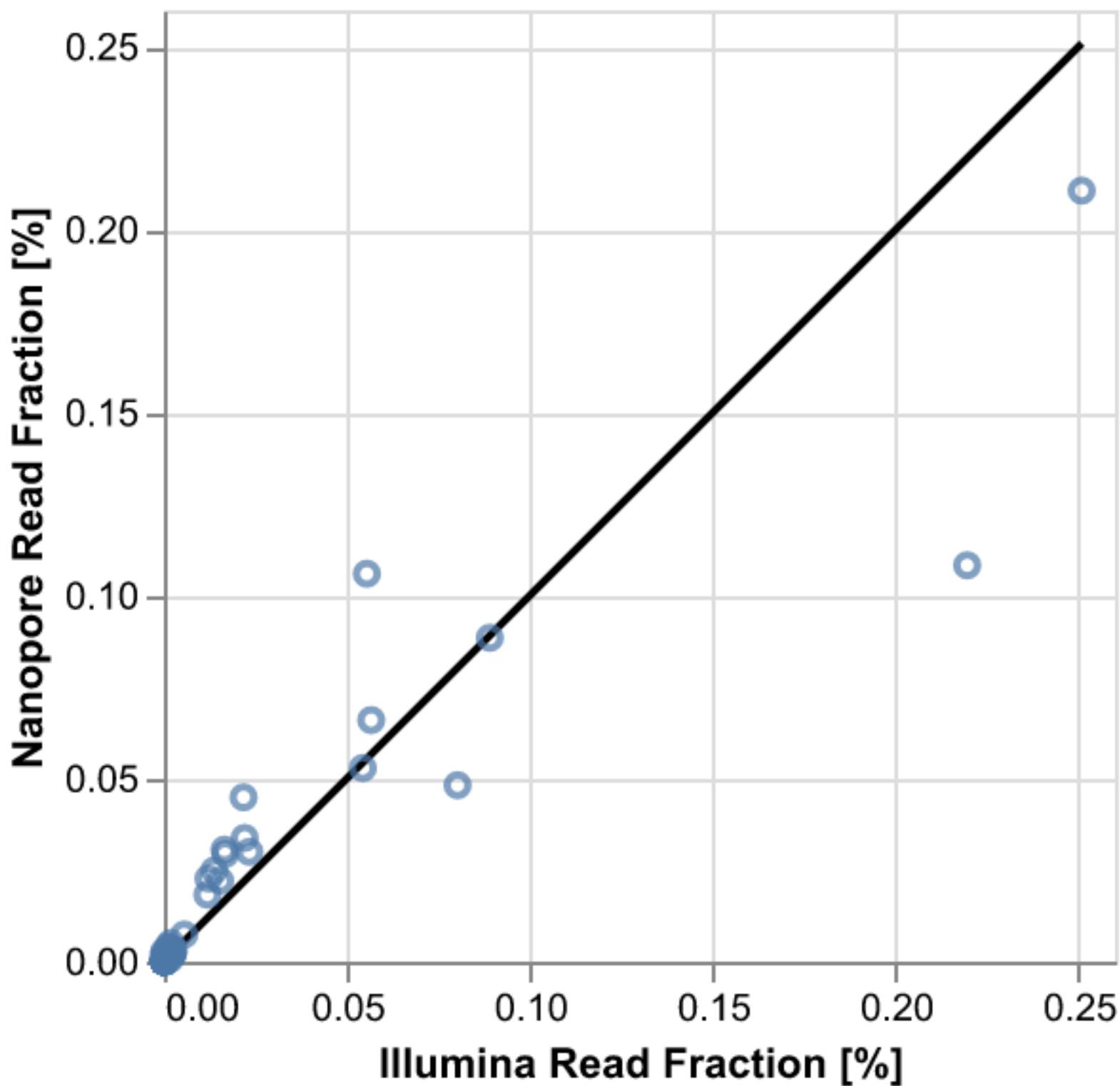

**18\_13**

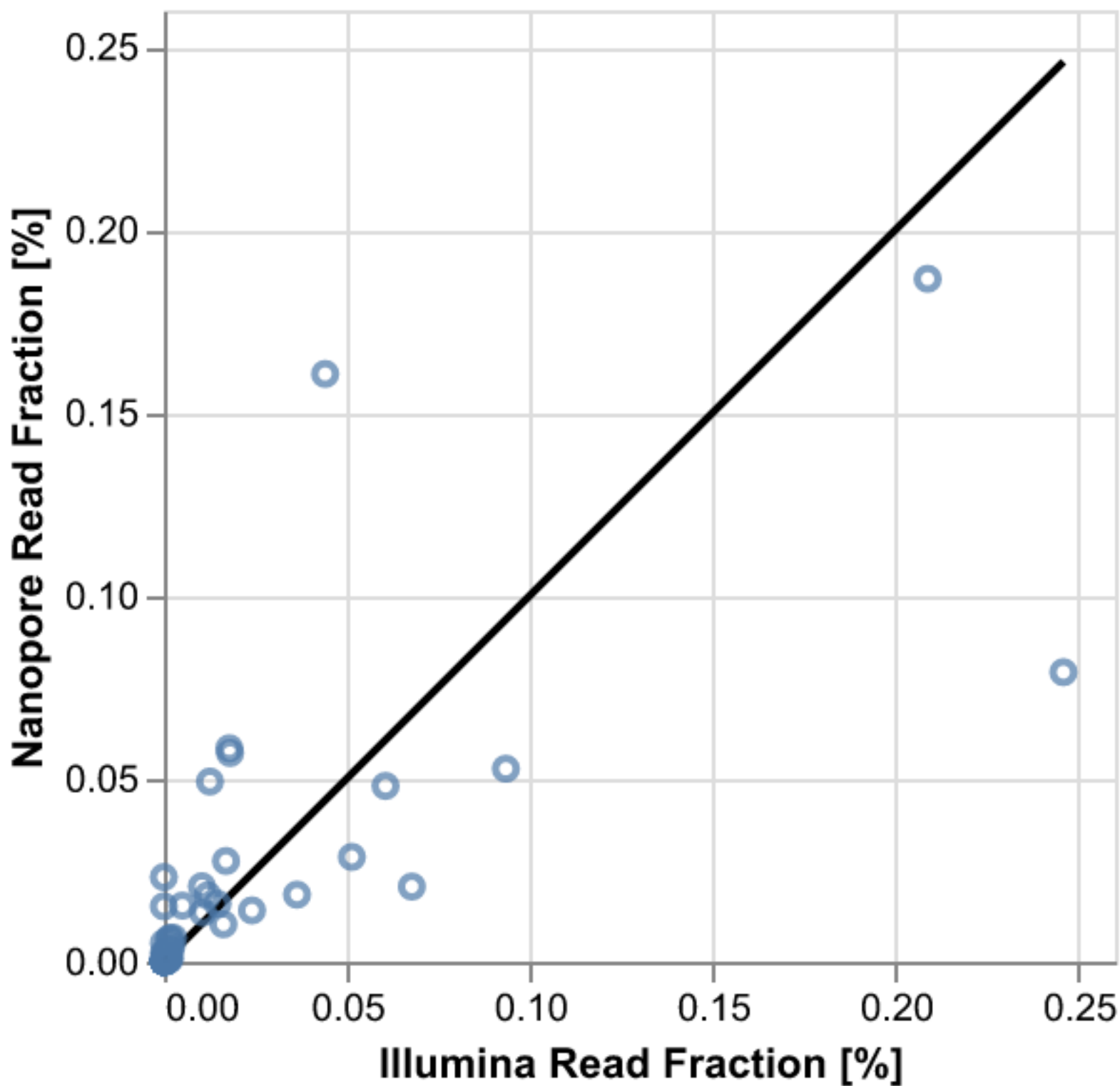

18.2\_16

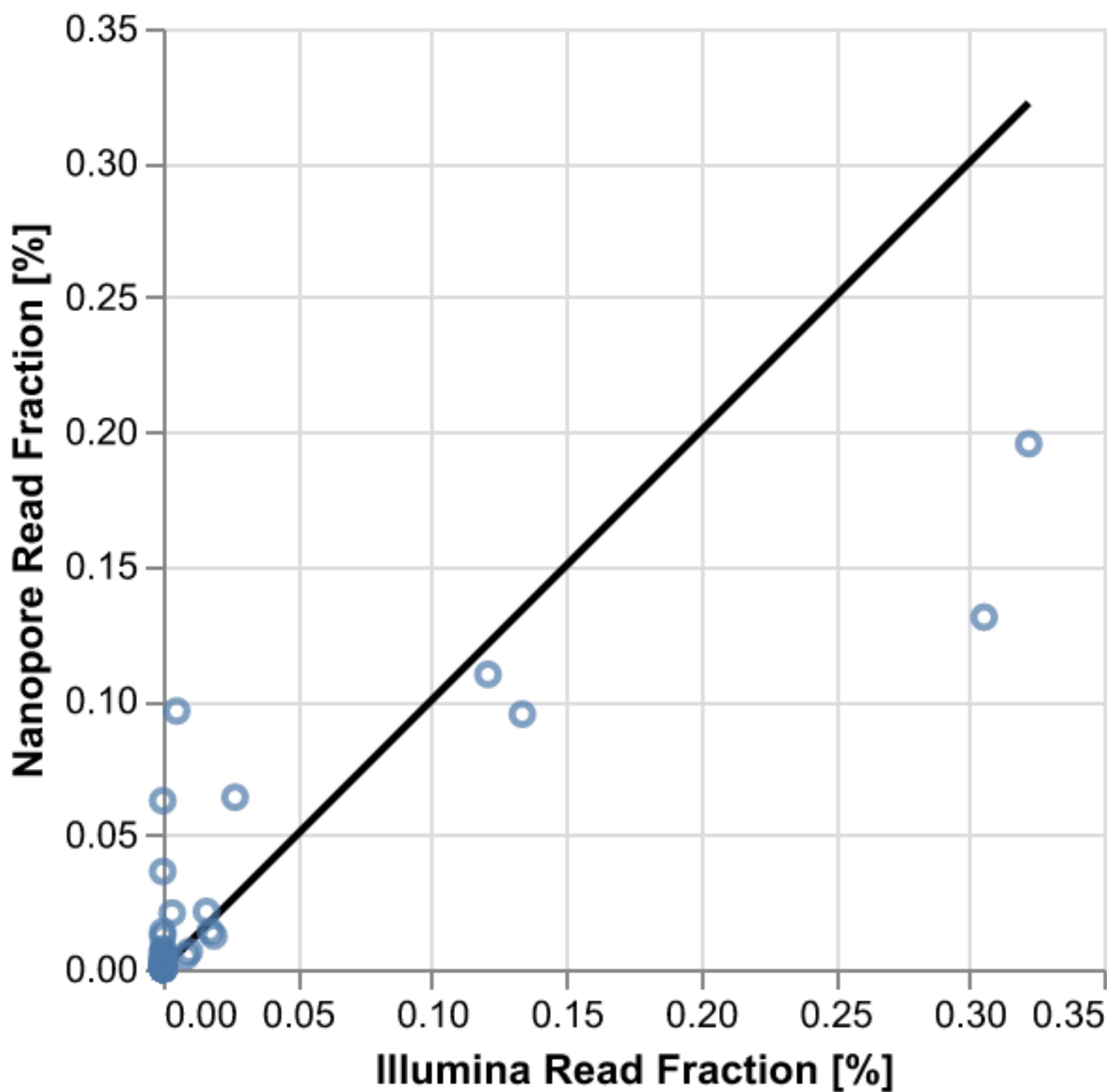

18\_-3

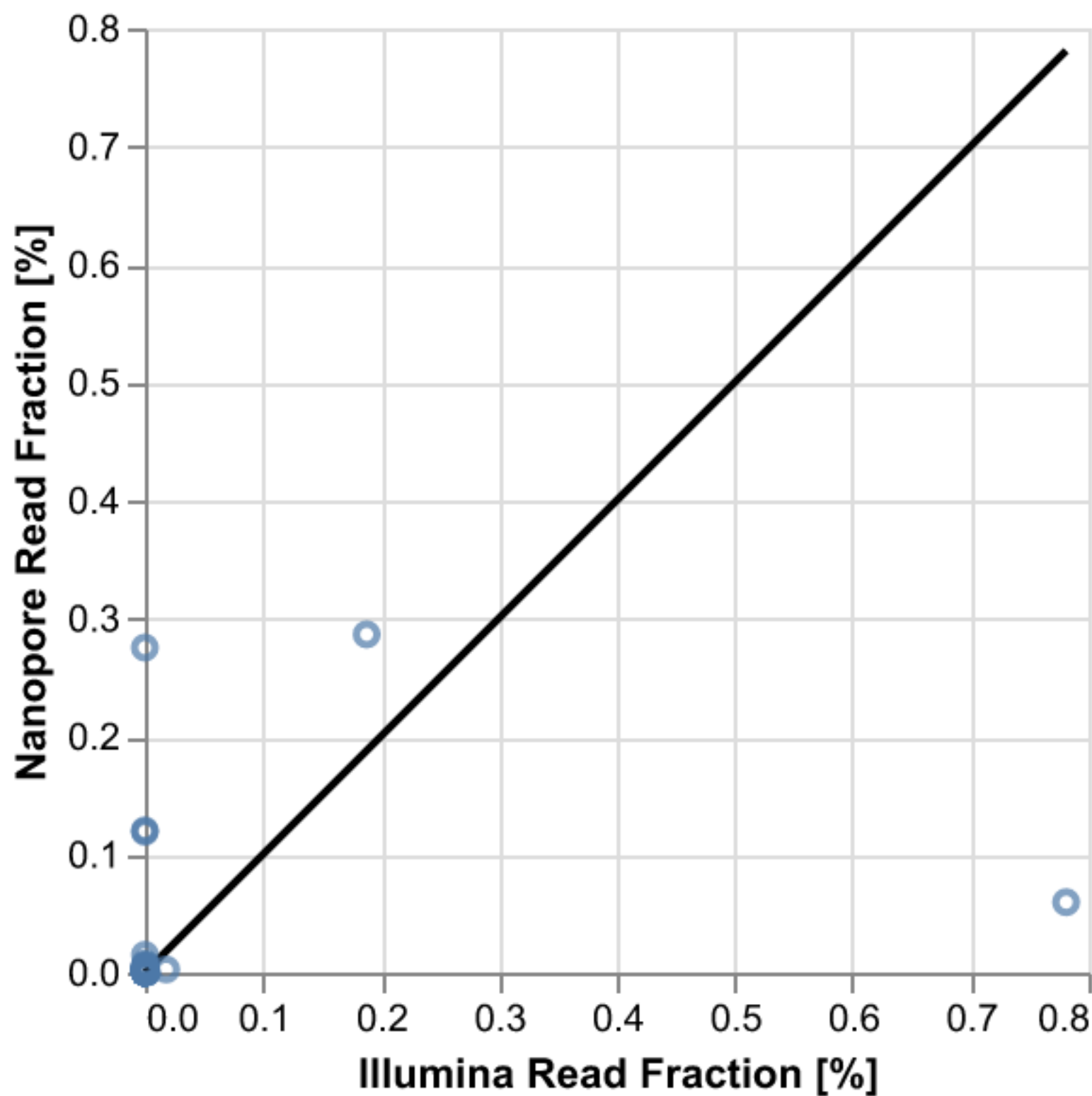

18\_91

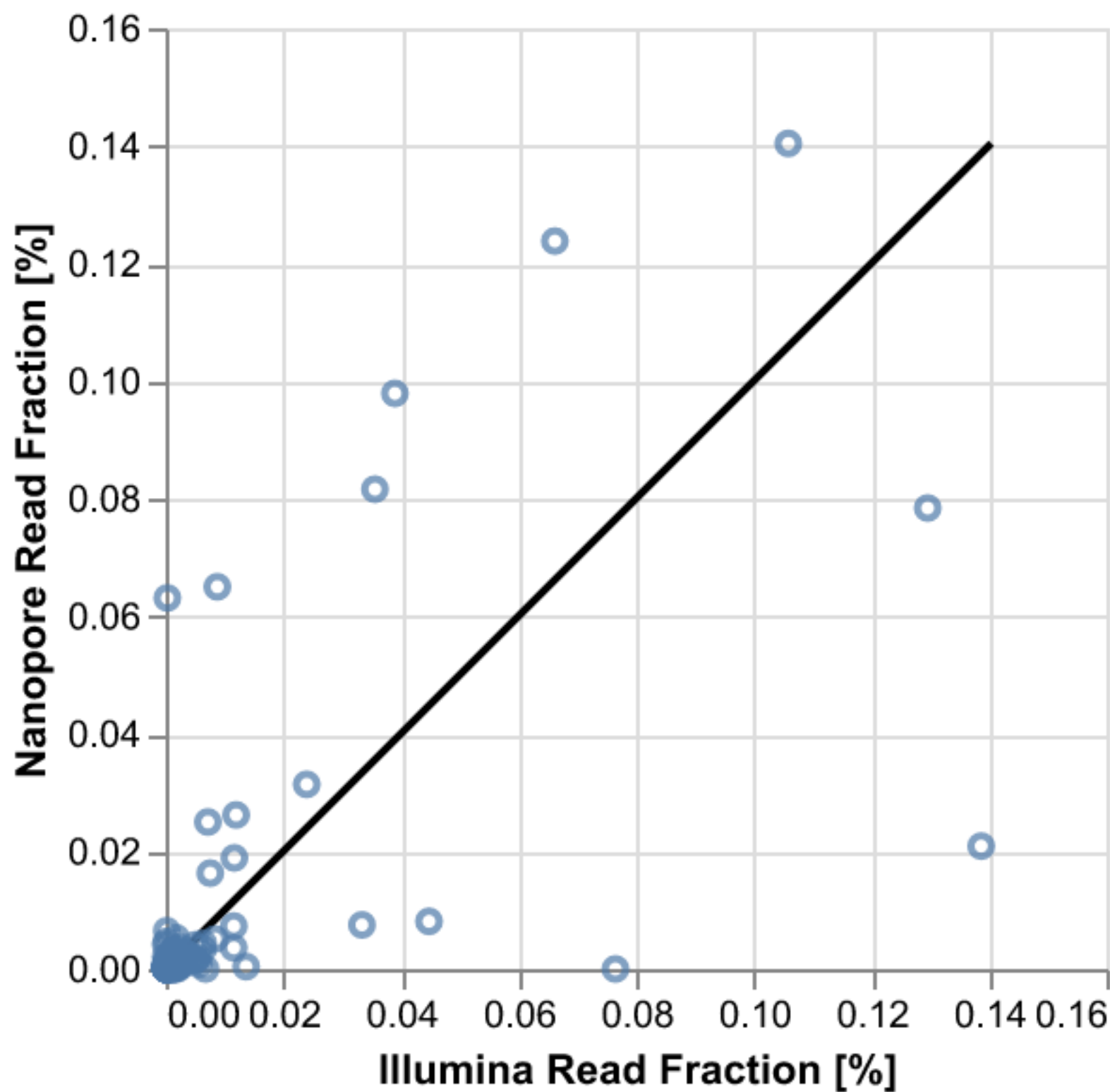

19\_153

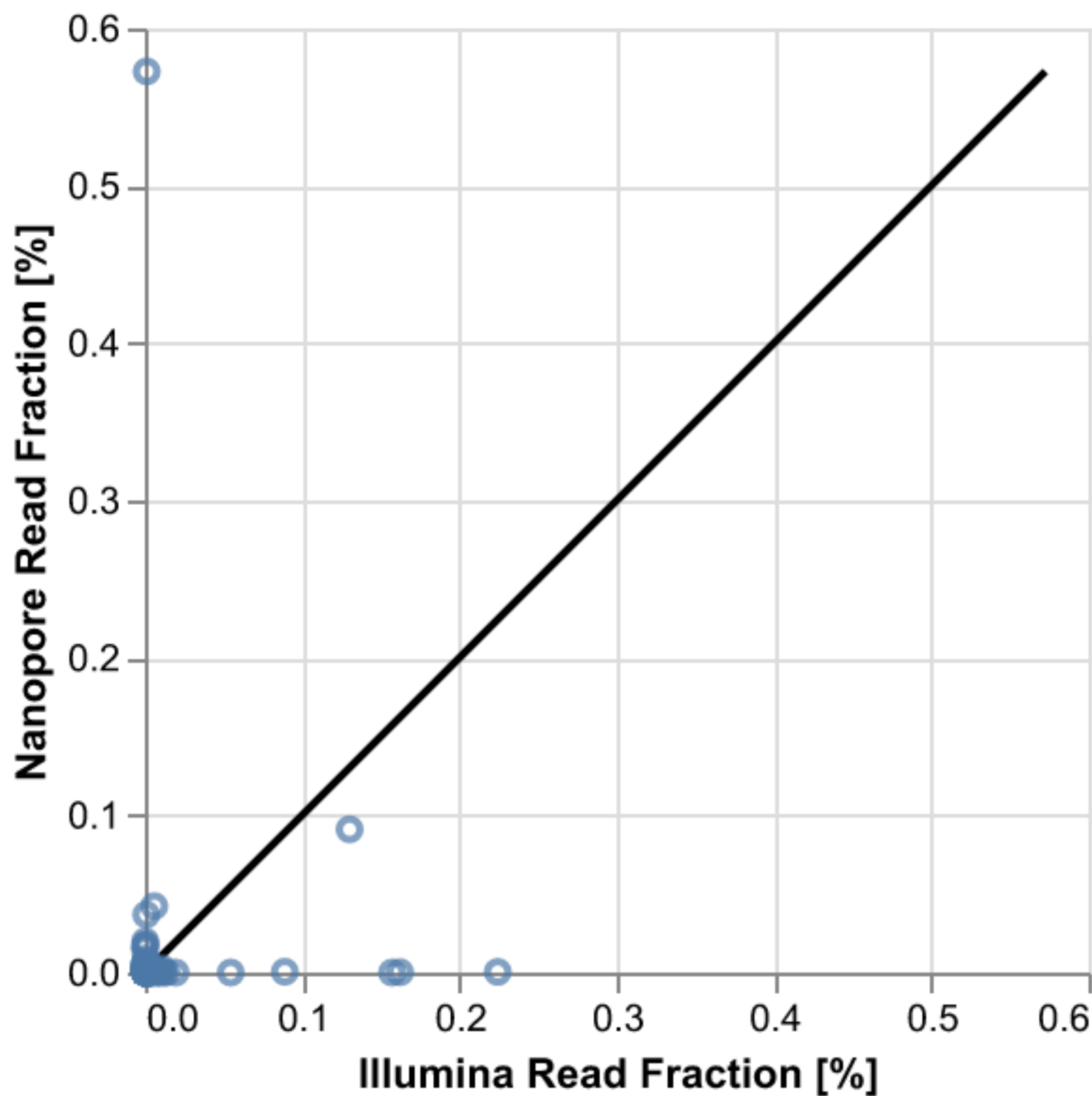

**19\_-2**

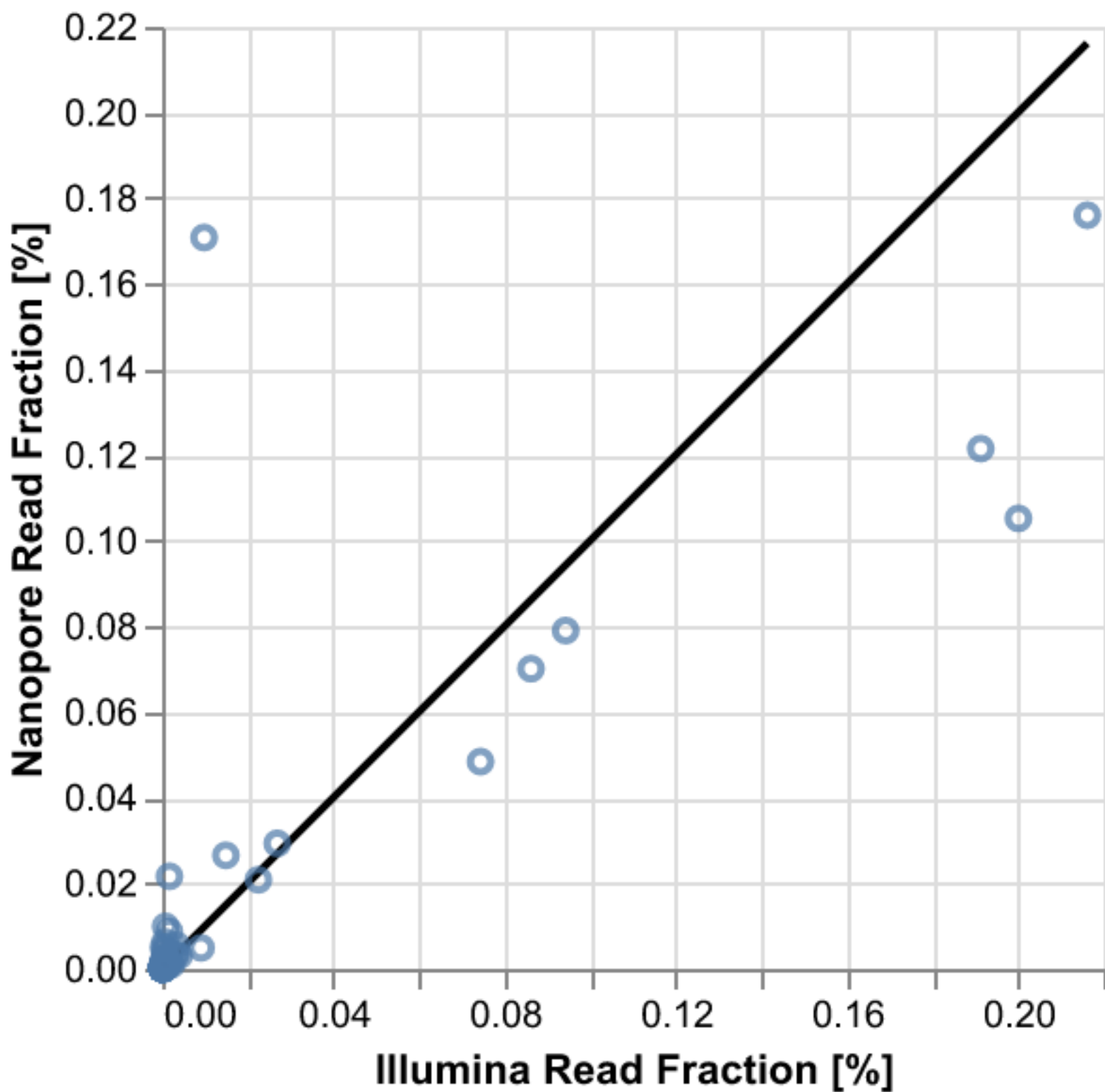

21\_120

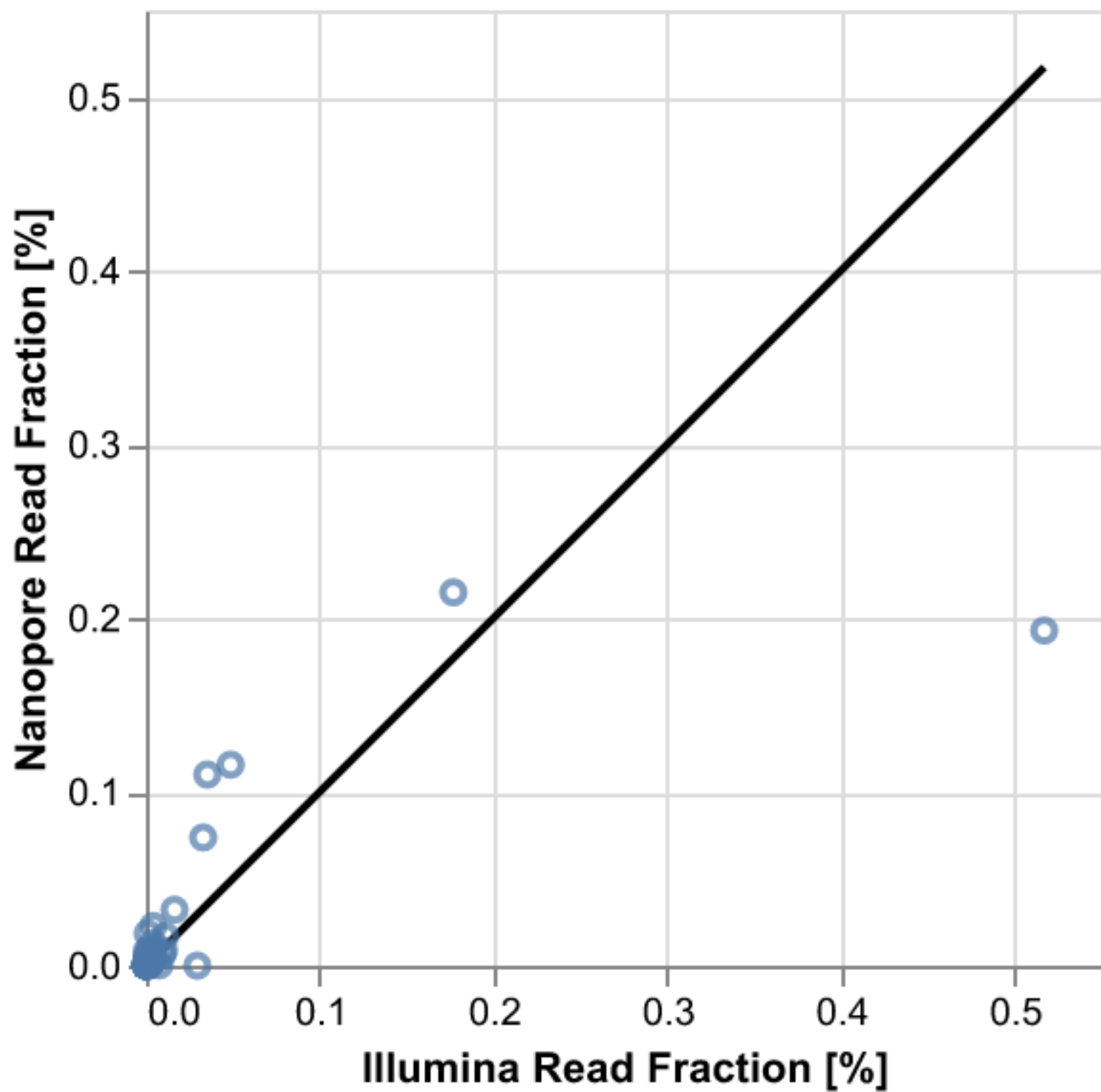

21\_-3

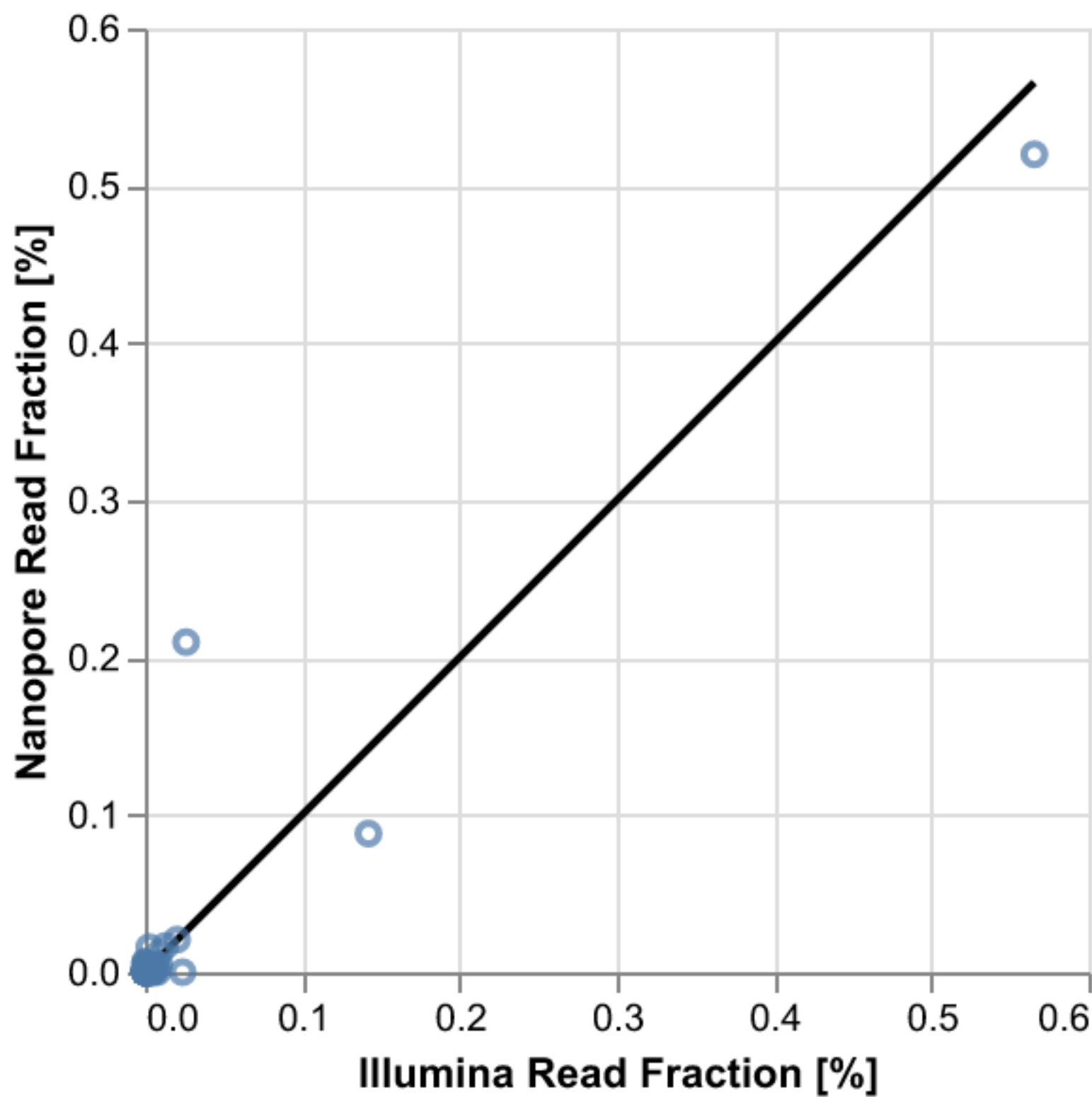

21\_50

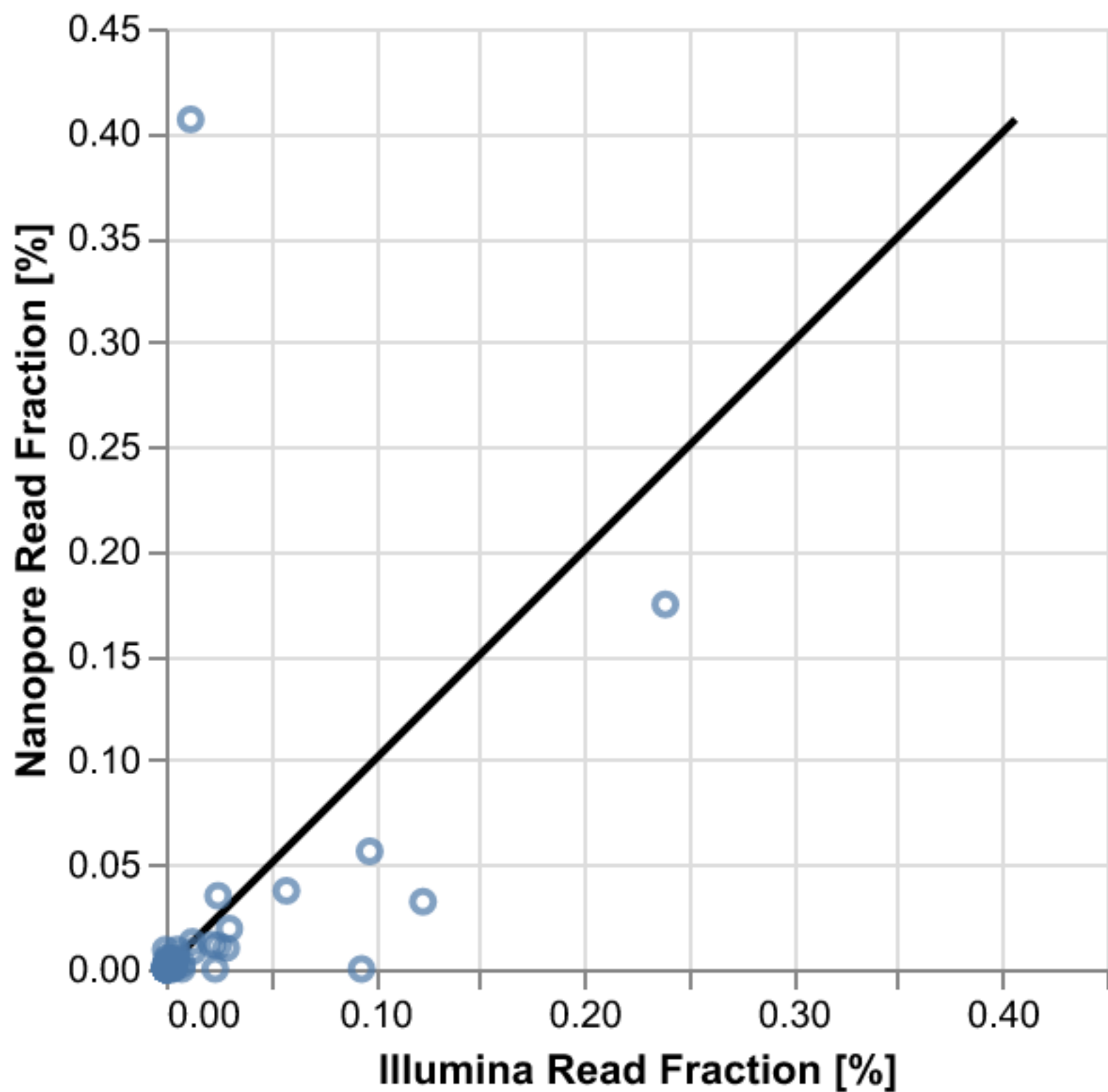

21\_-9

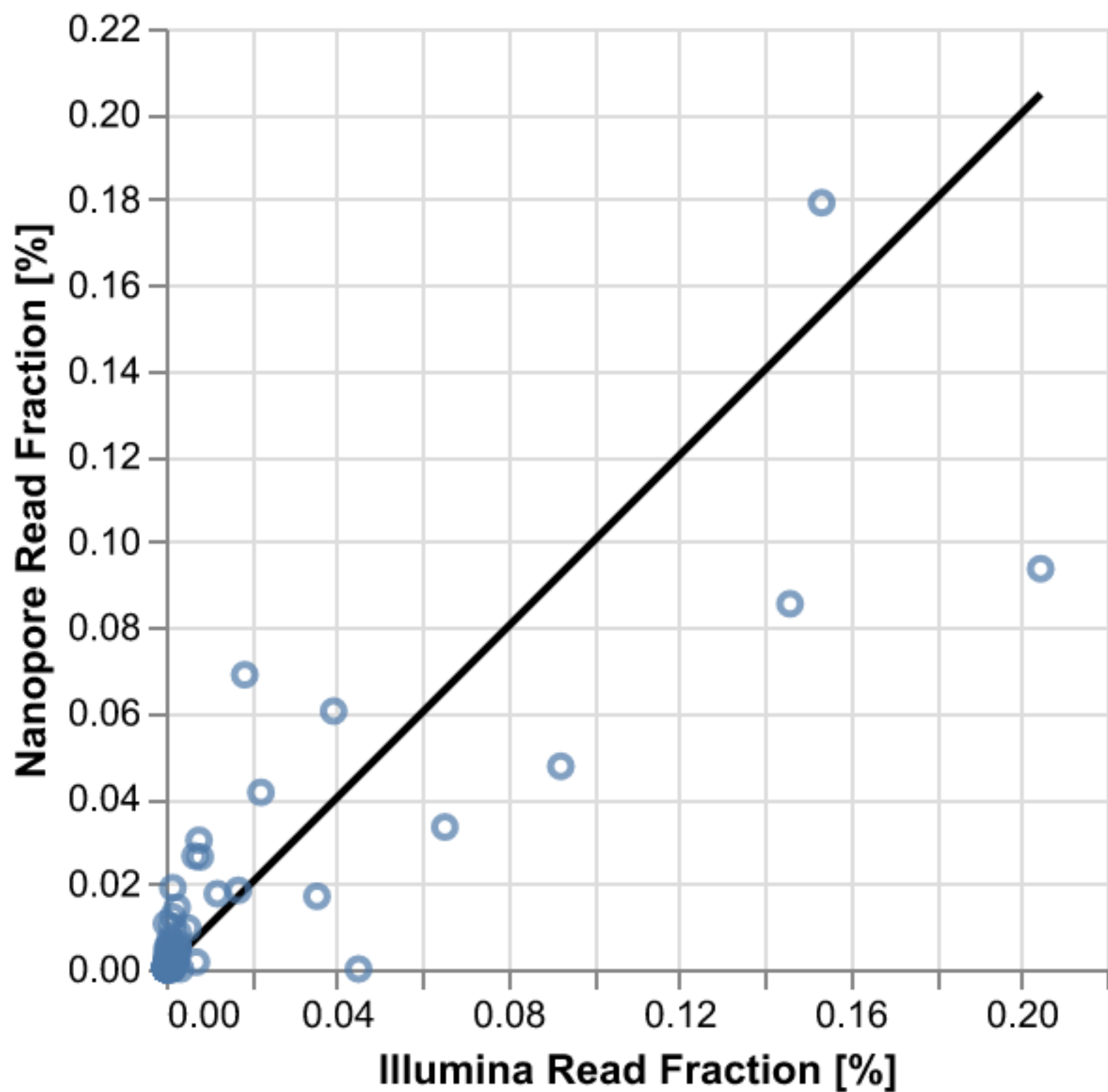

23\_-9

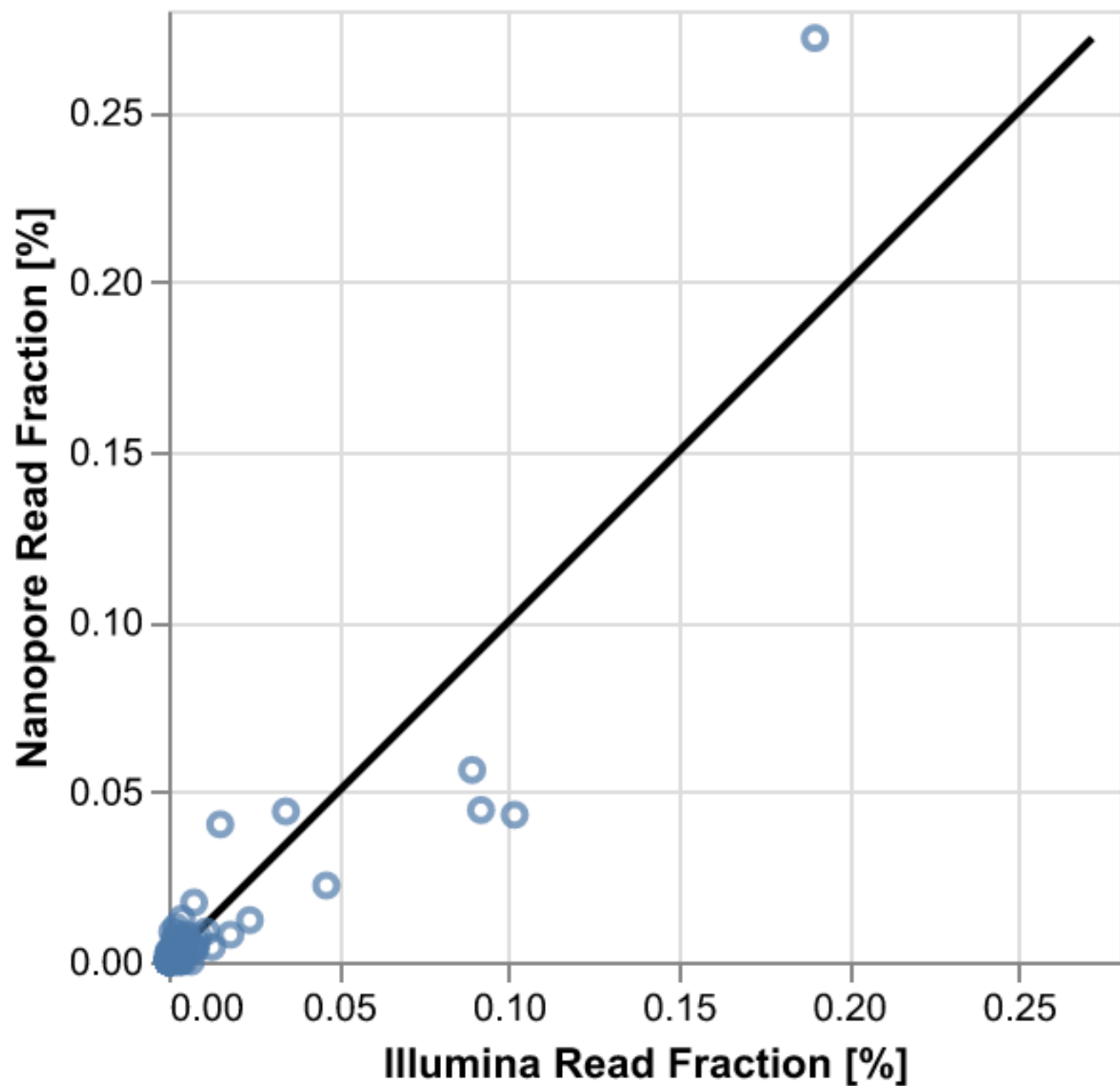

**24\_0**

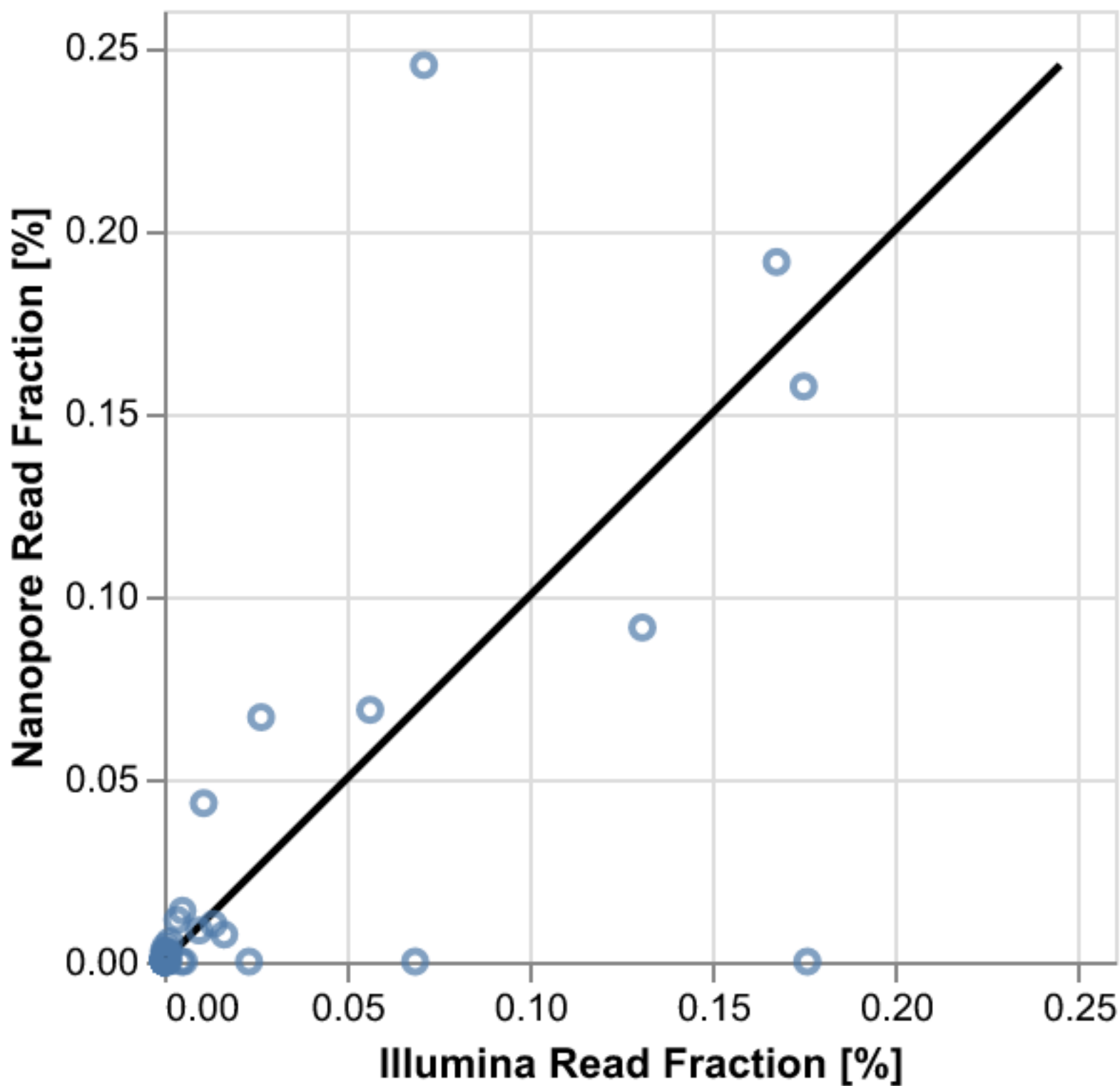

24\_-10

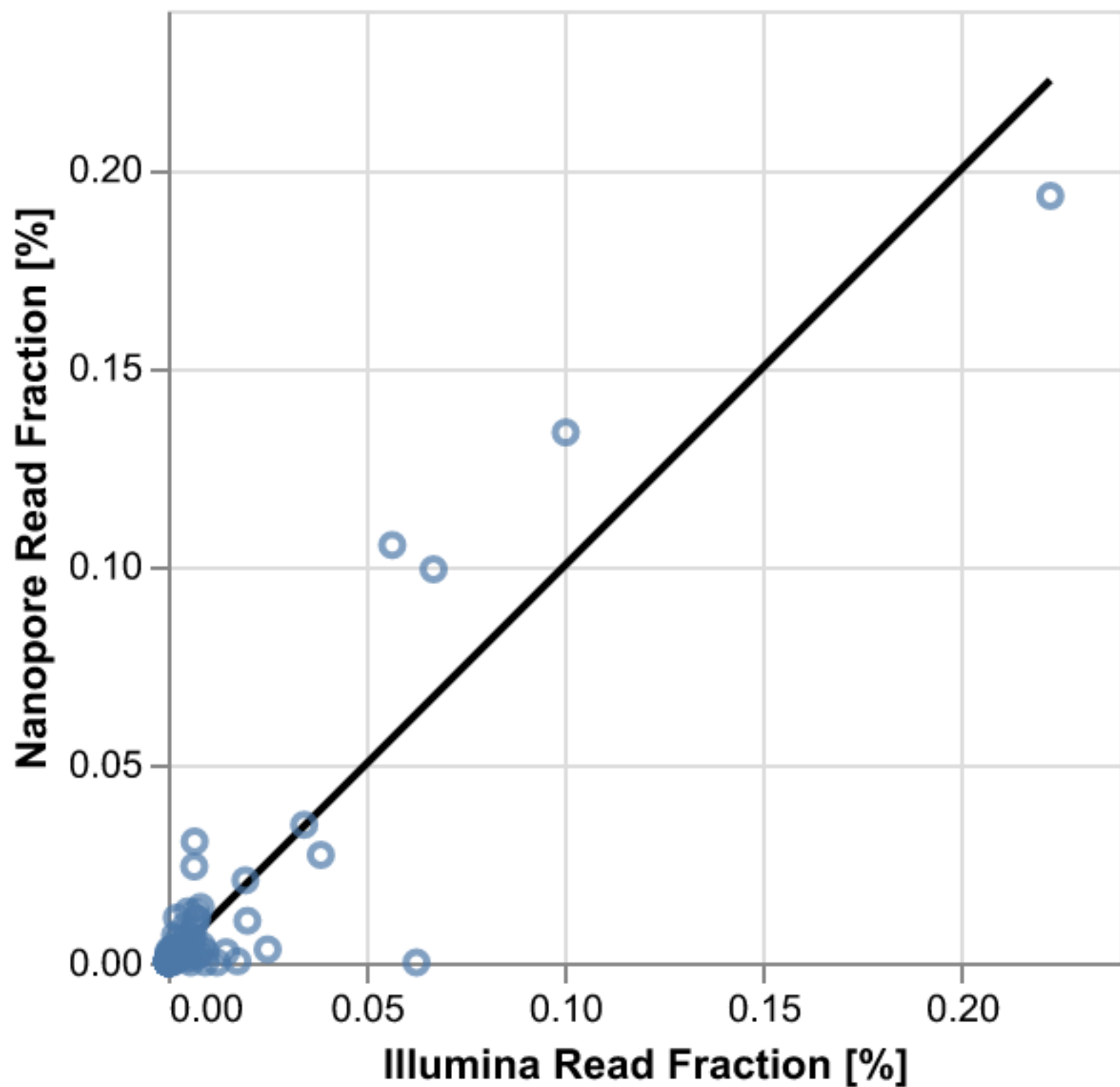

24\_49

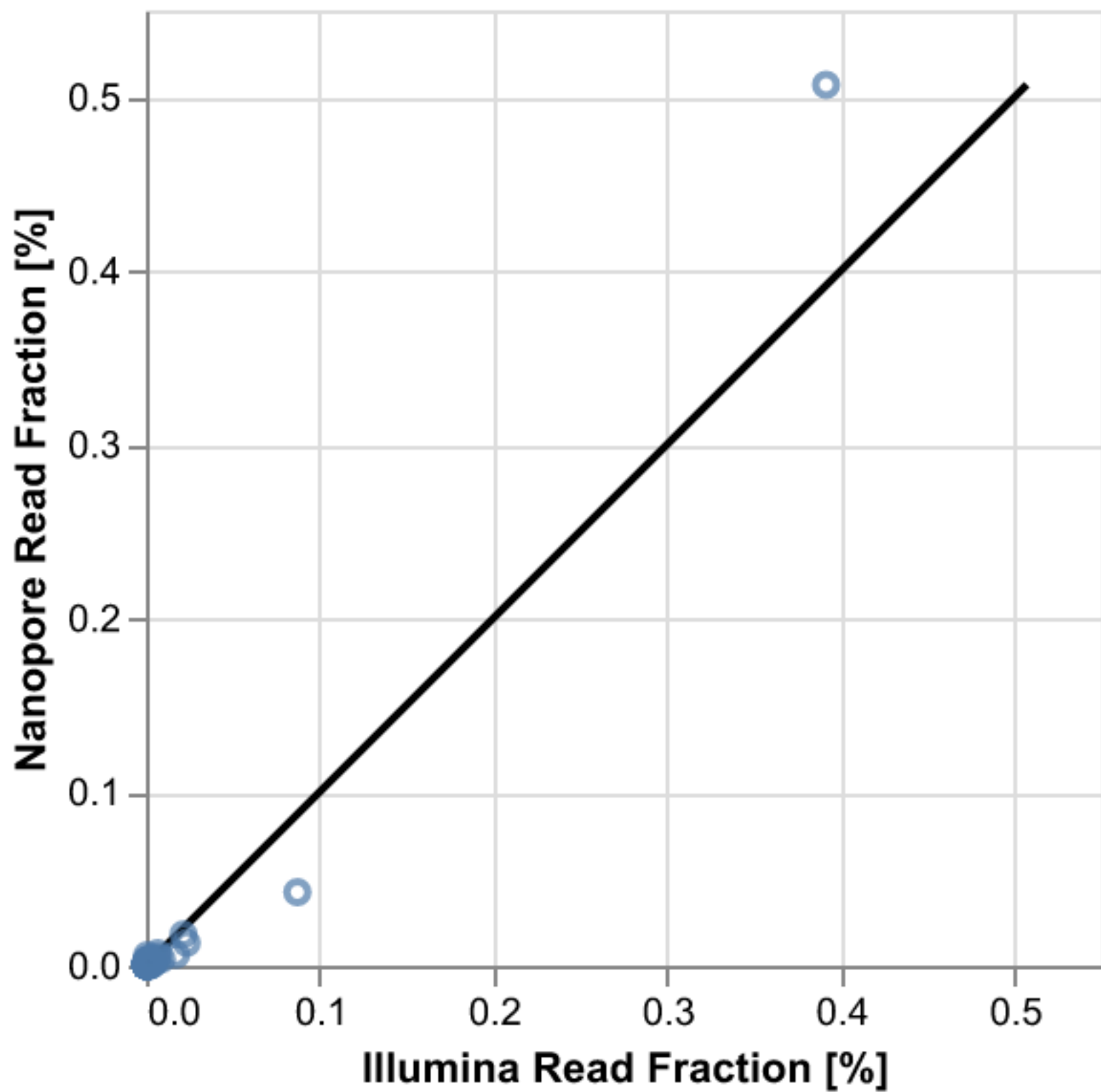

24\_9

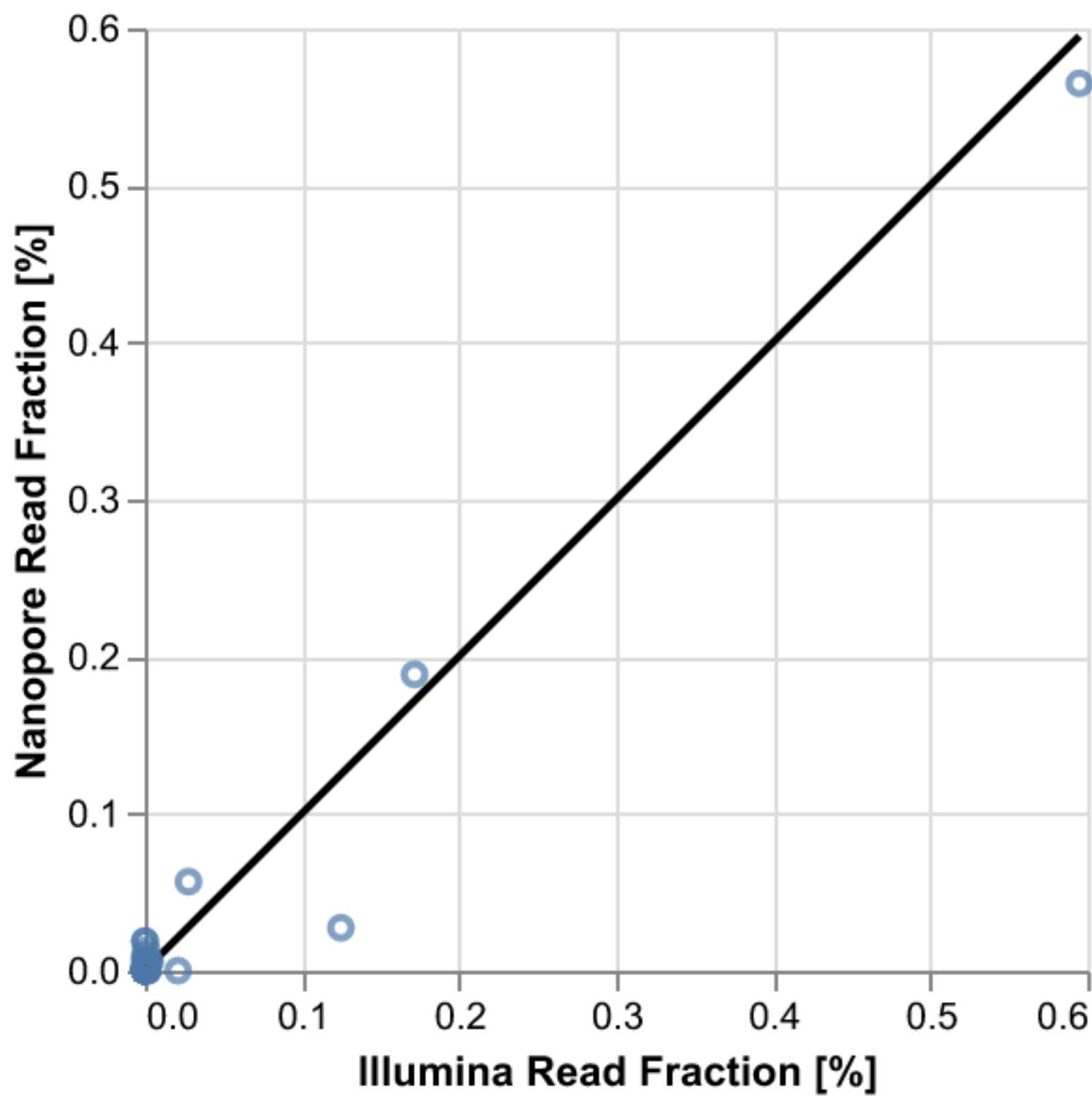

**26\_185**

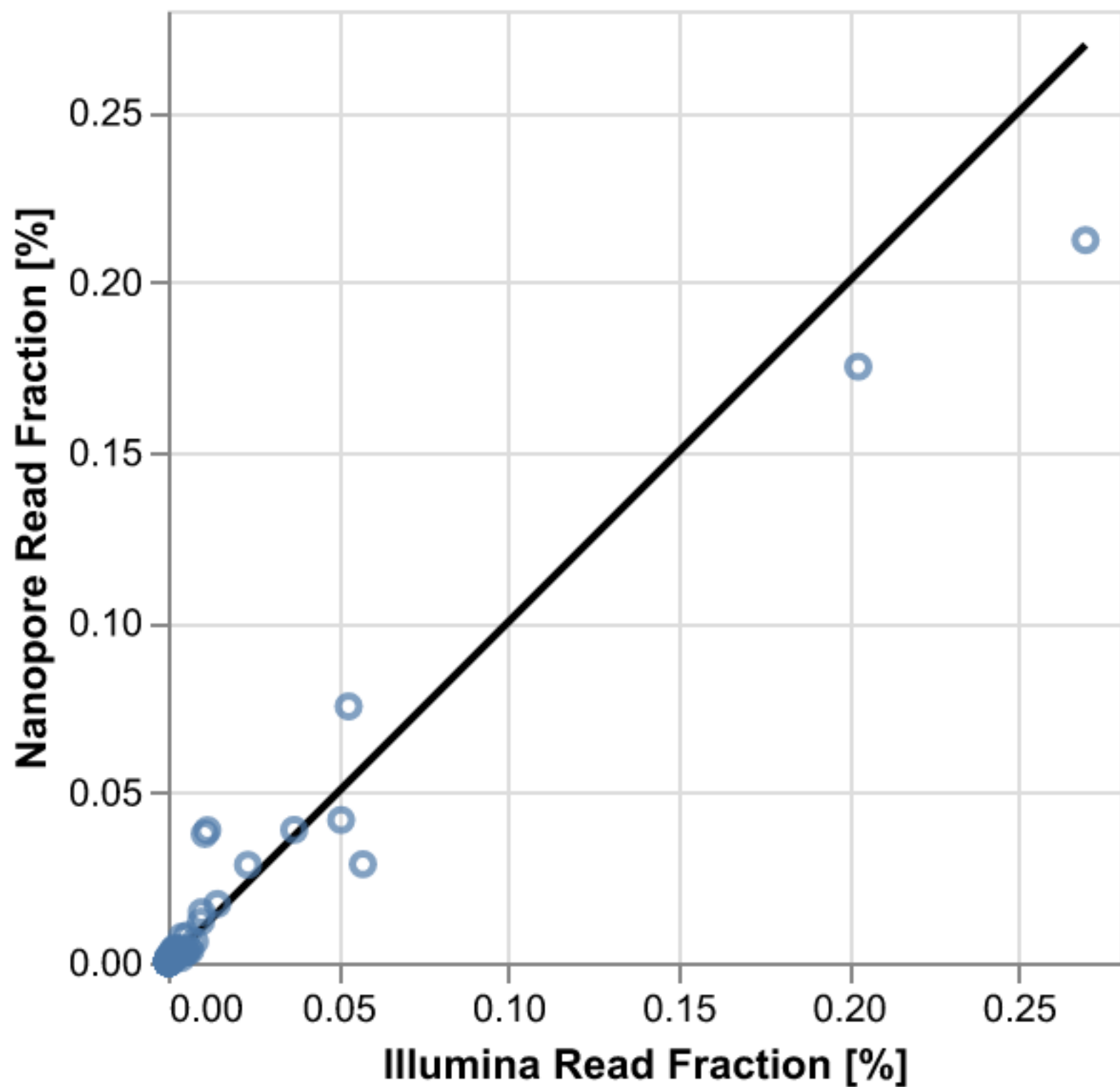

26\_27

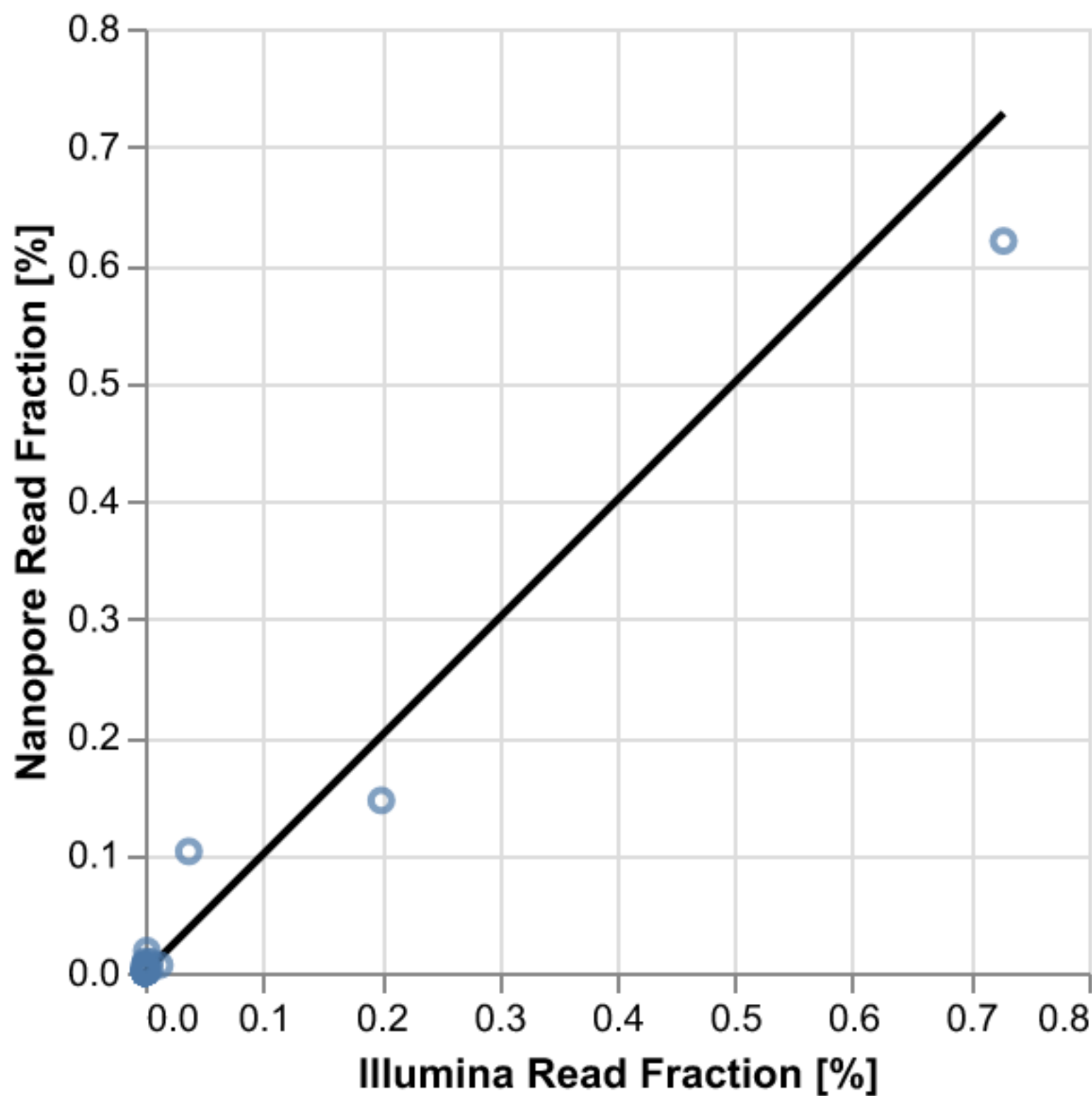

26\_-3

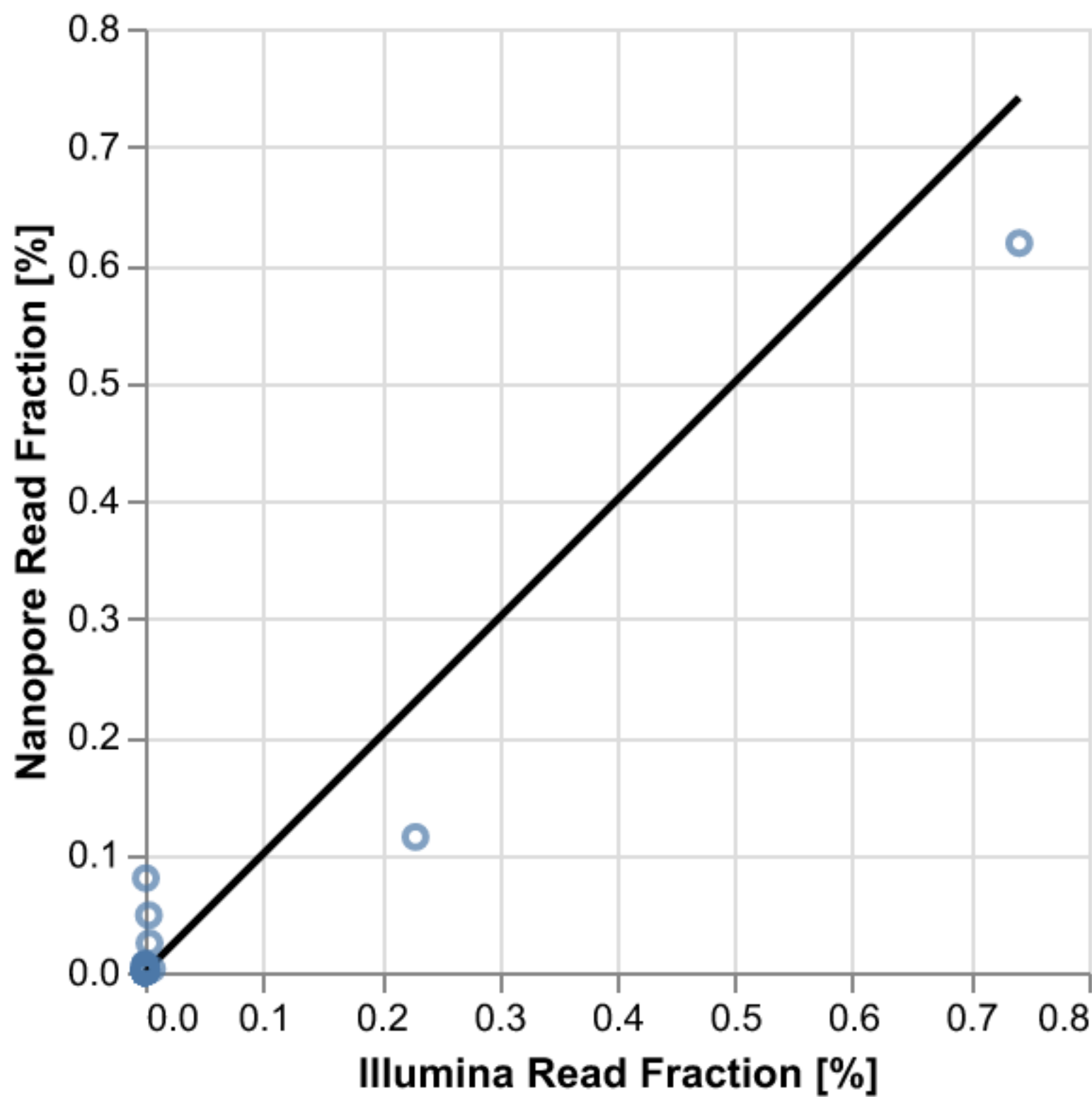

28\_1

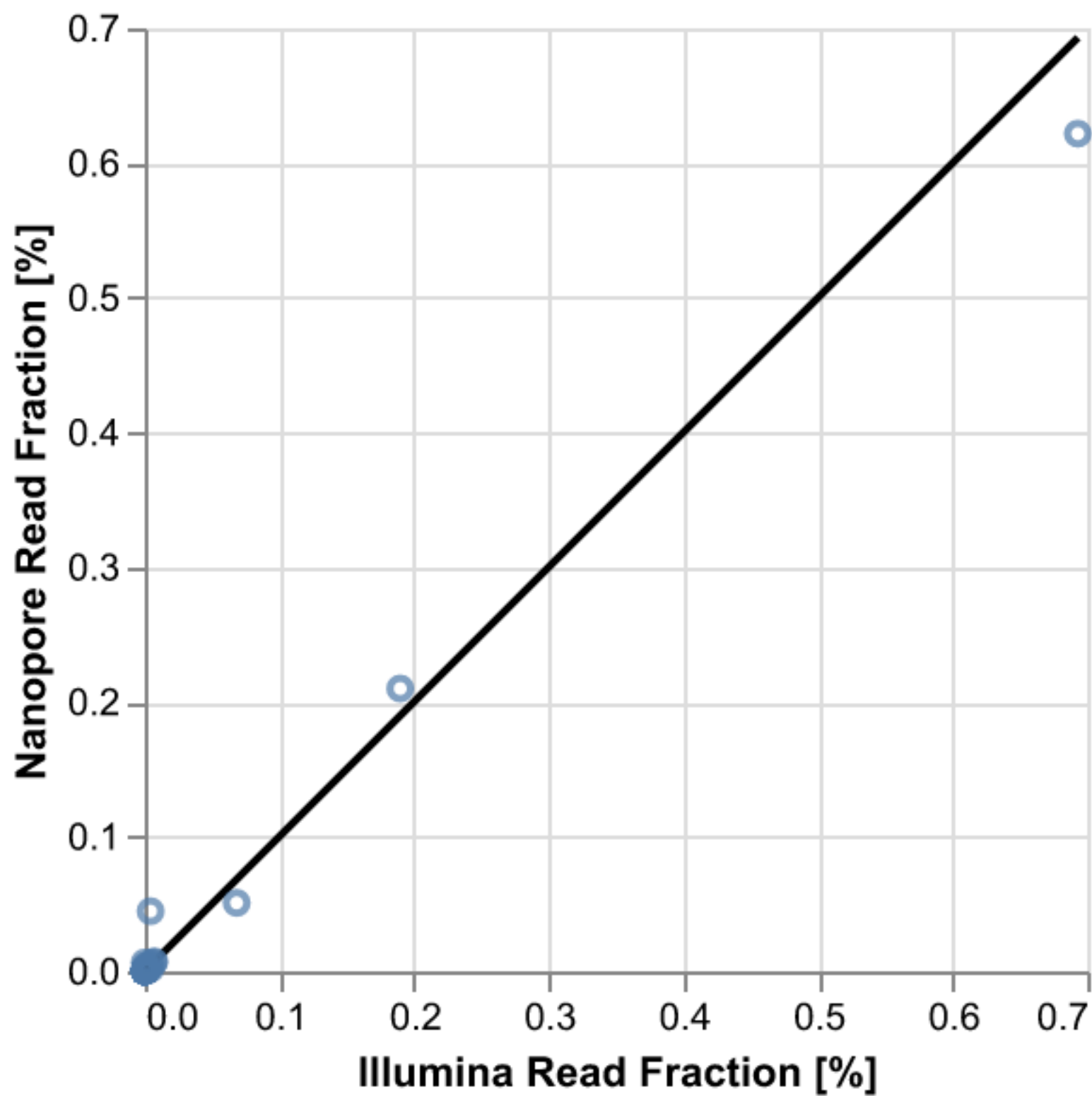

28\_21

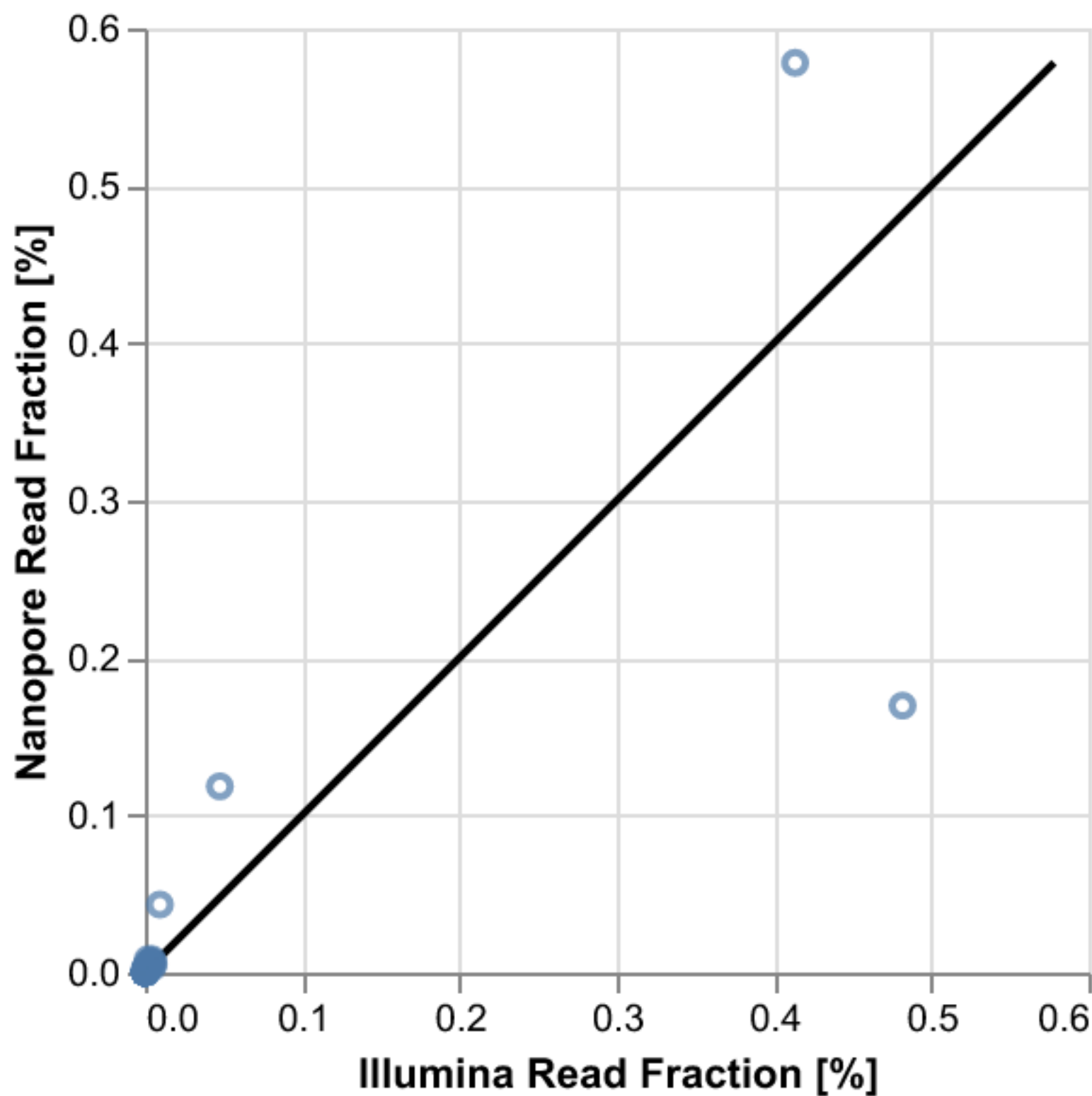

28\_237

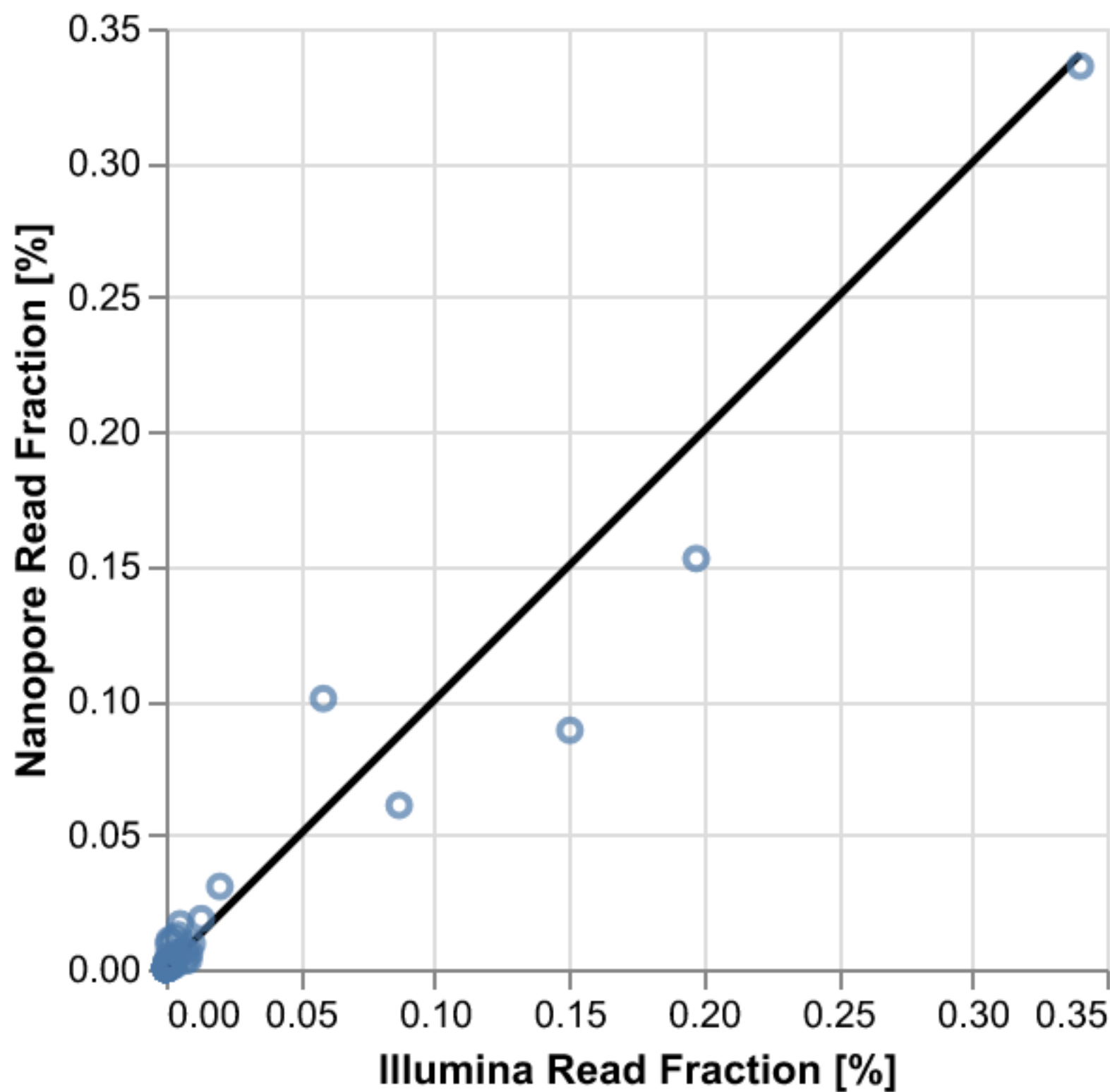

28\_-5

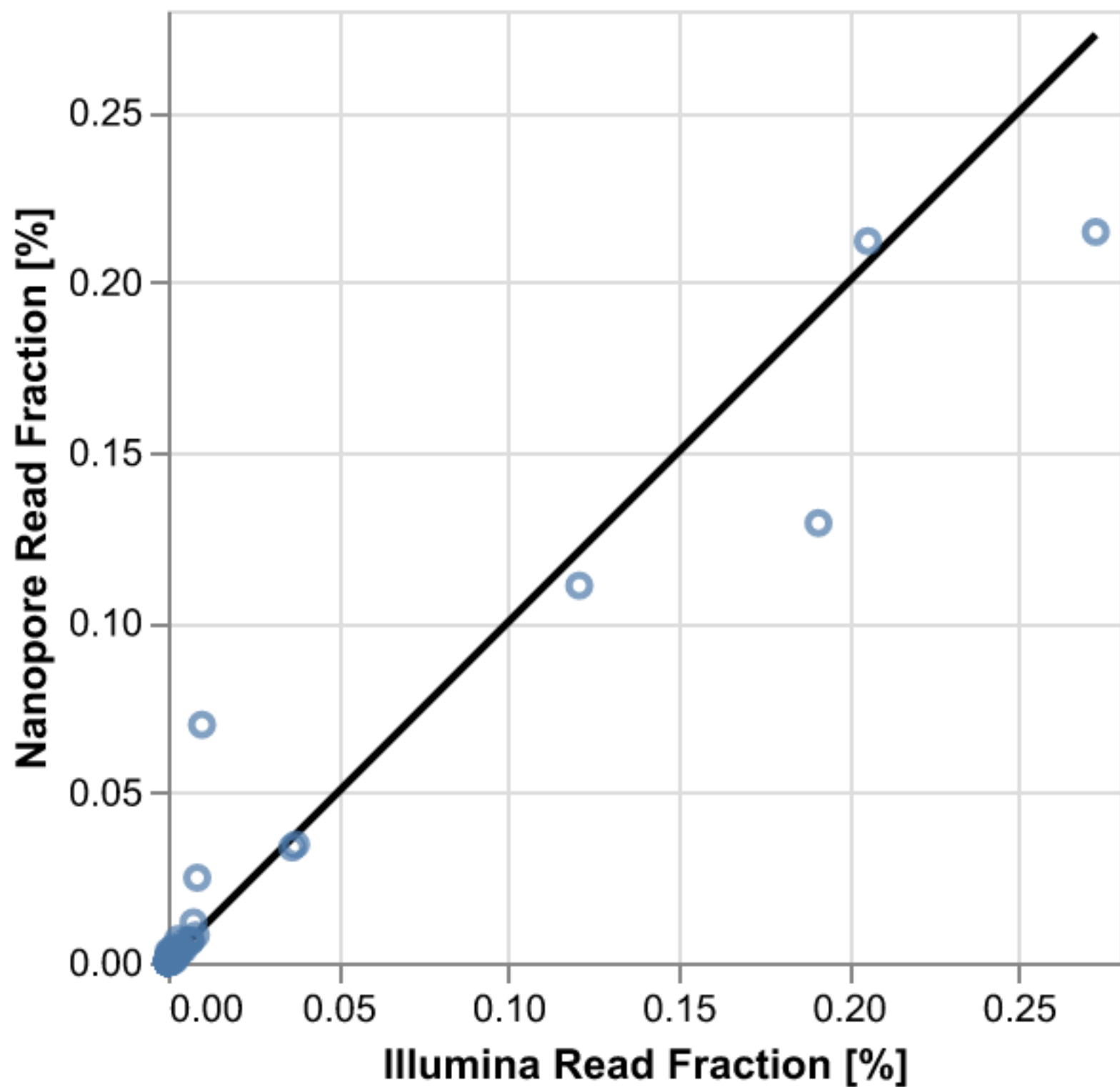

28\_63

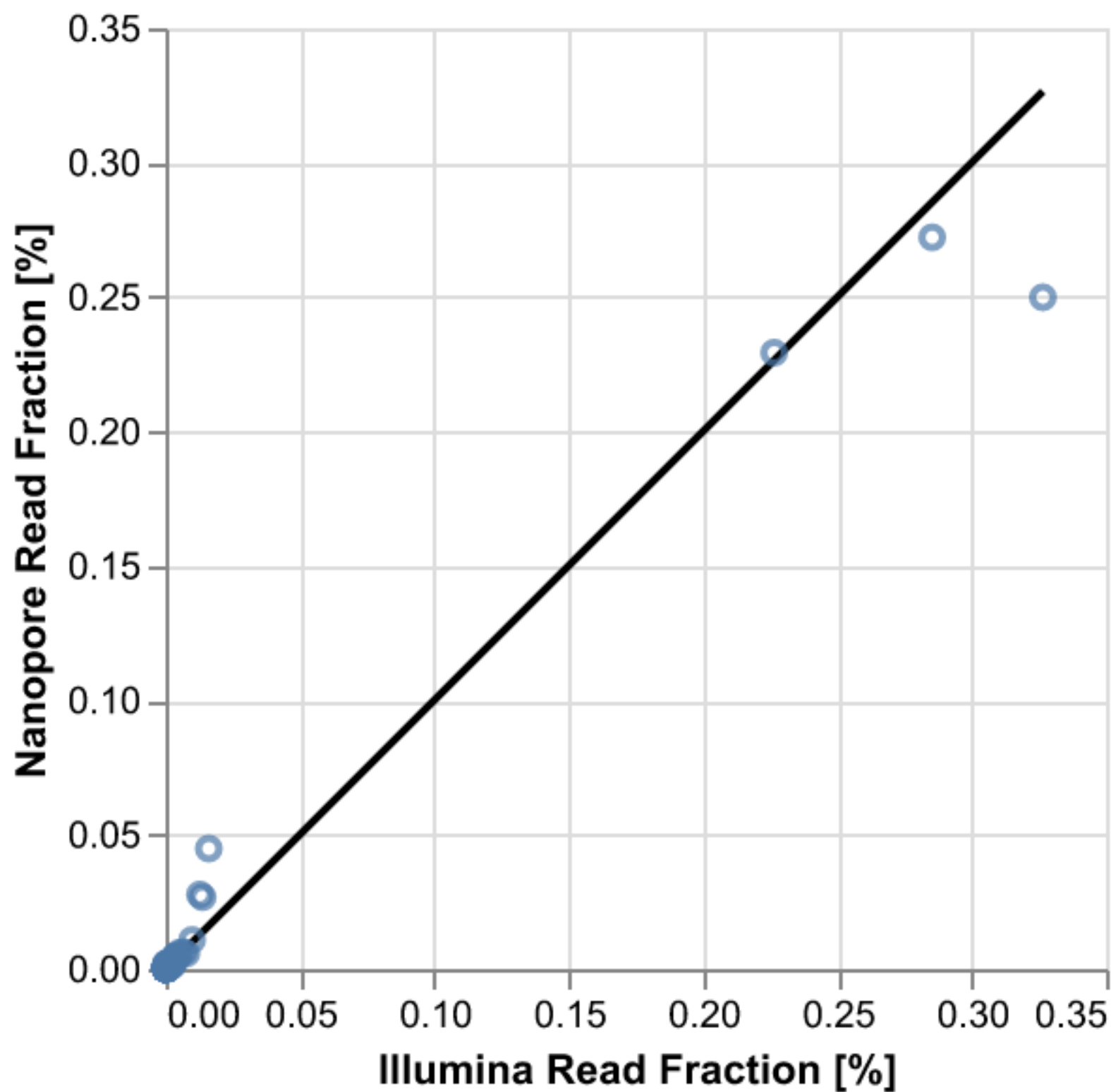

29\_15

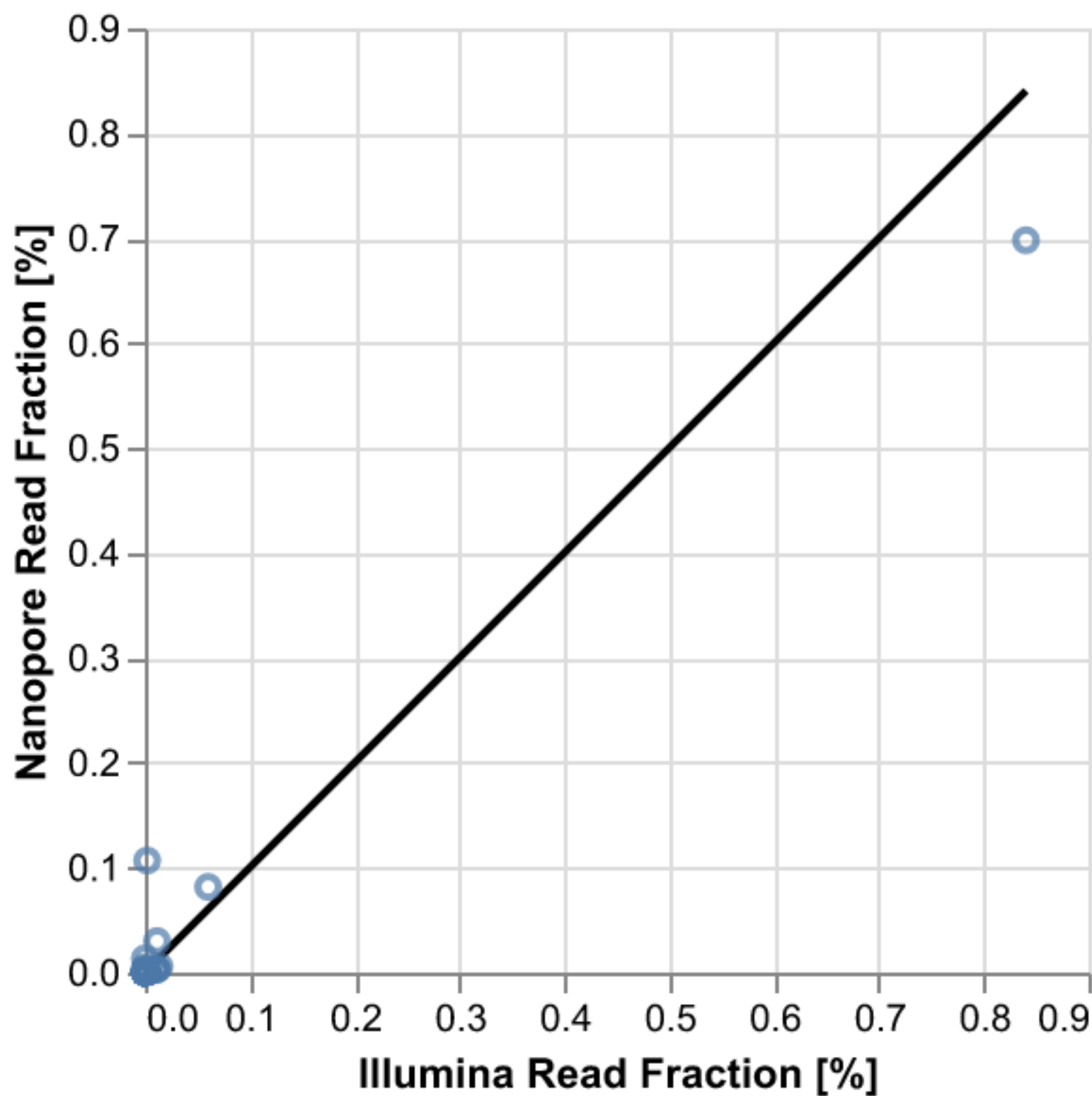

29\_186

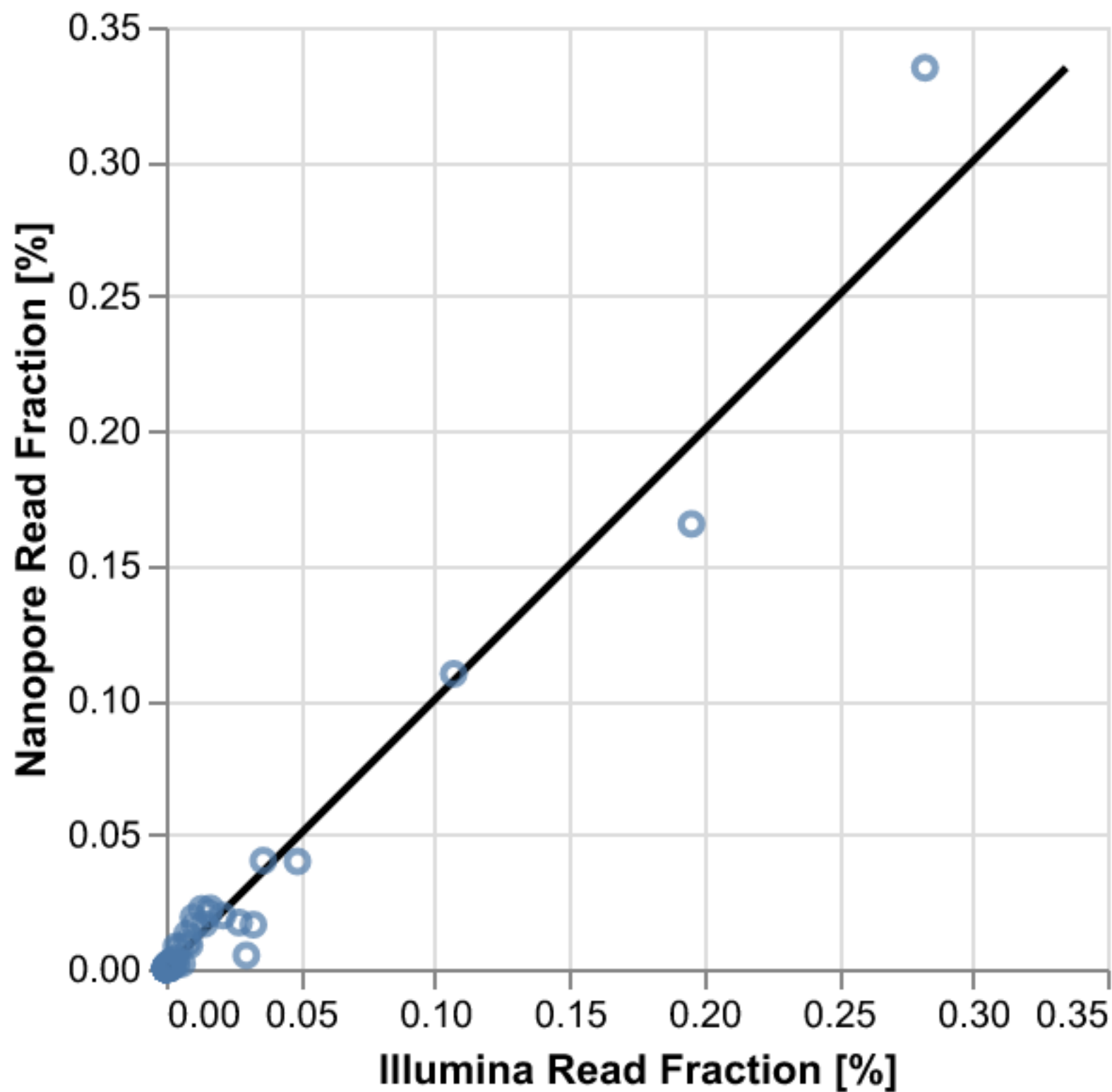

29\_1

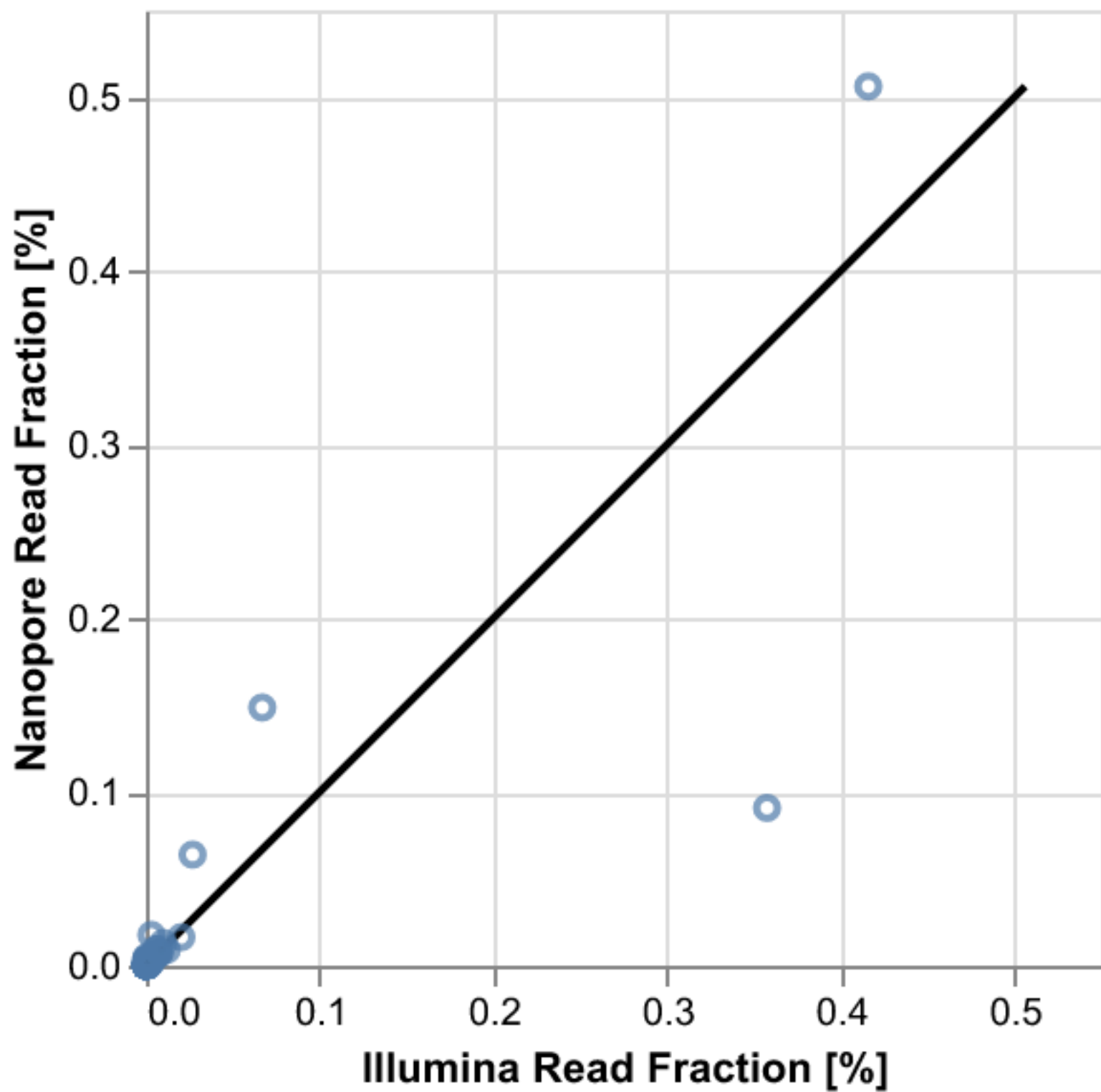

**29\_-5**

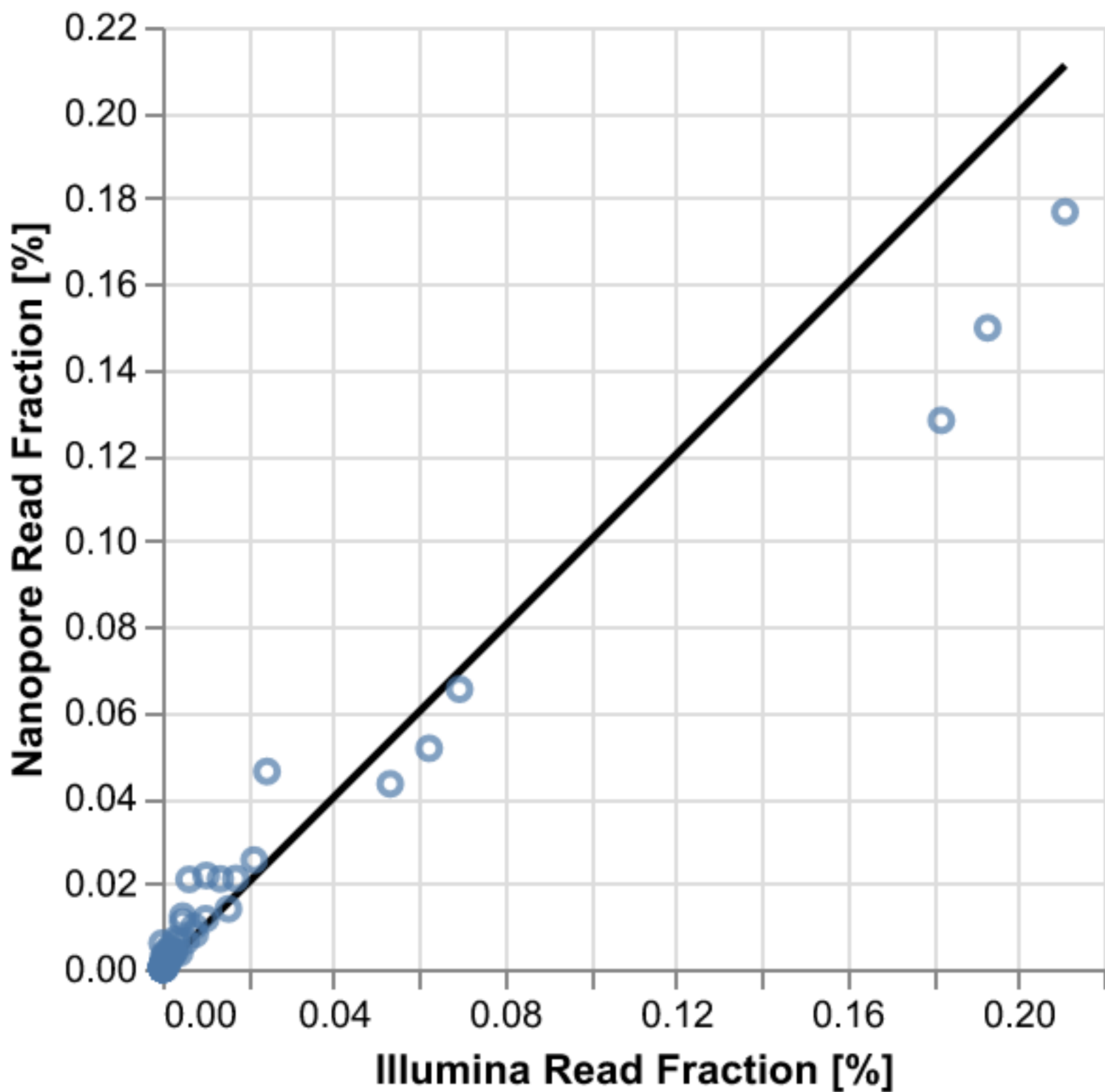

31\_58

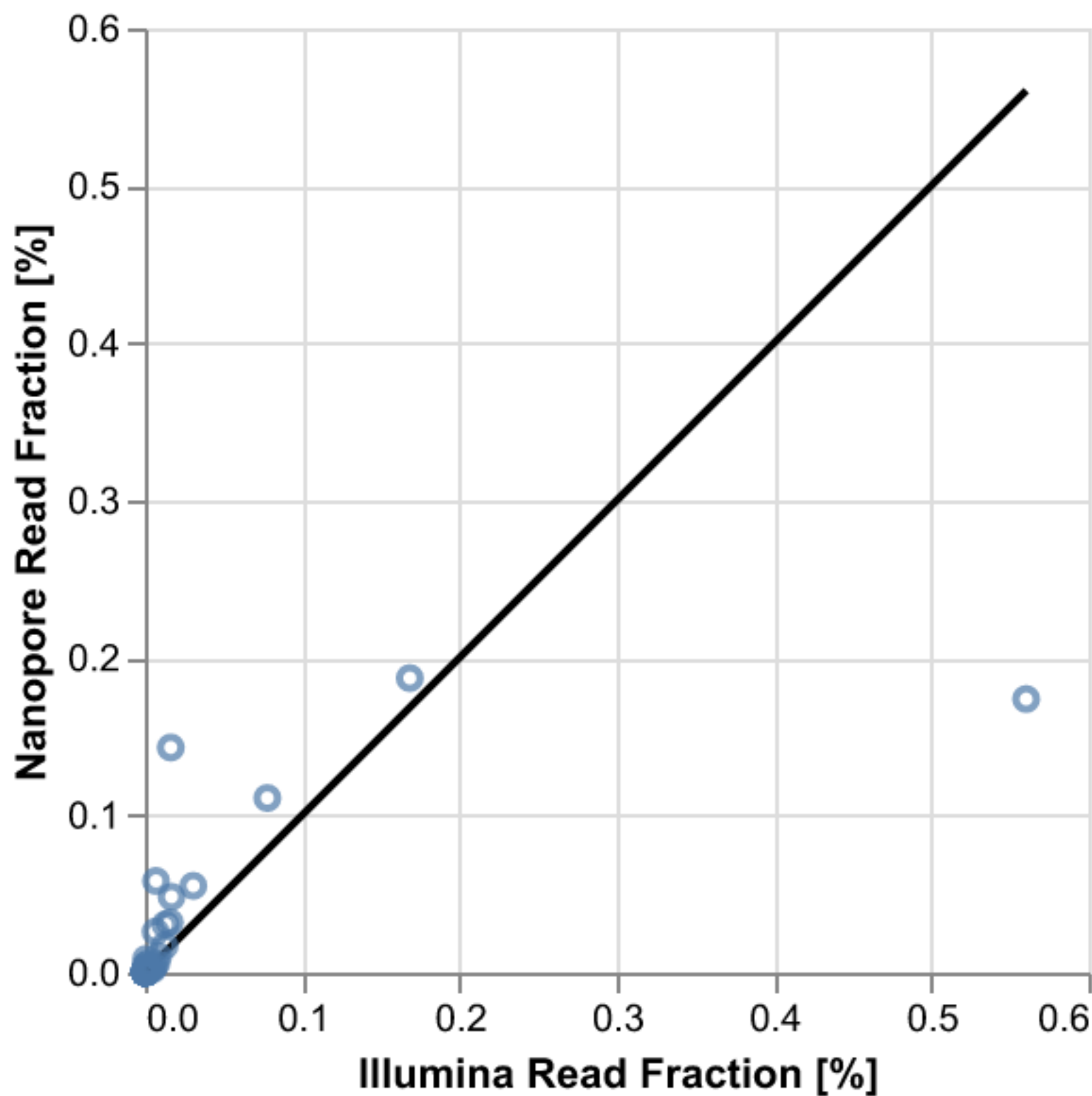

31\_-6

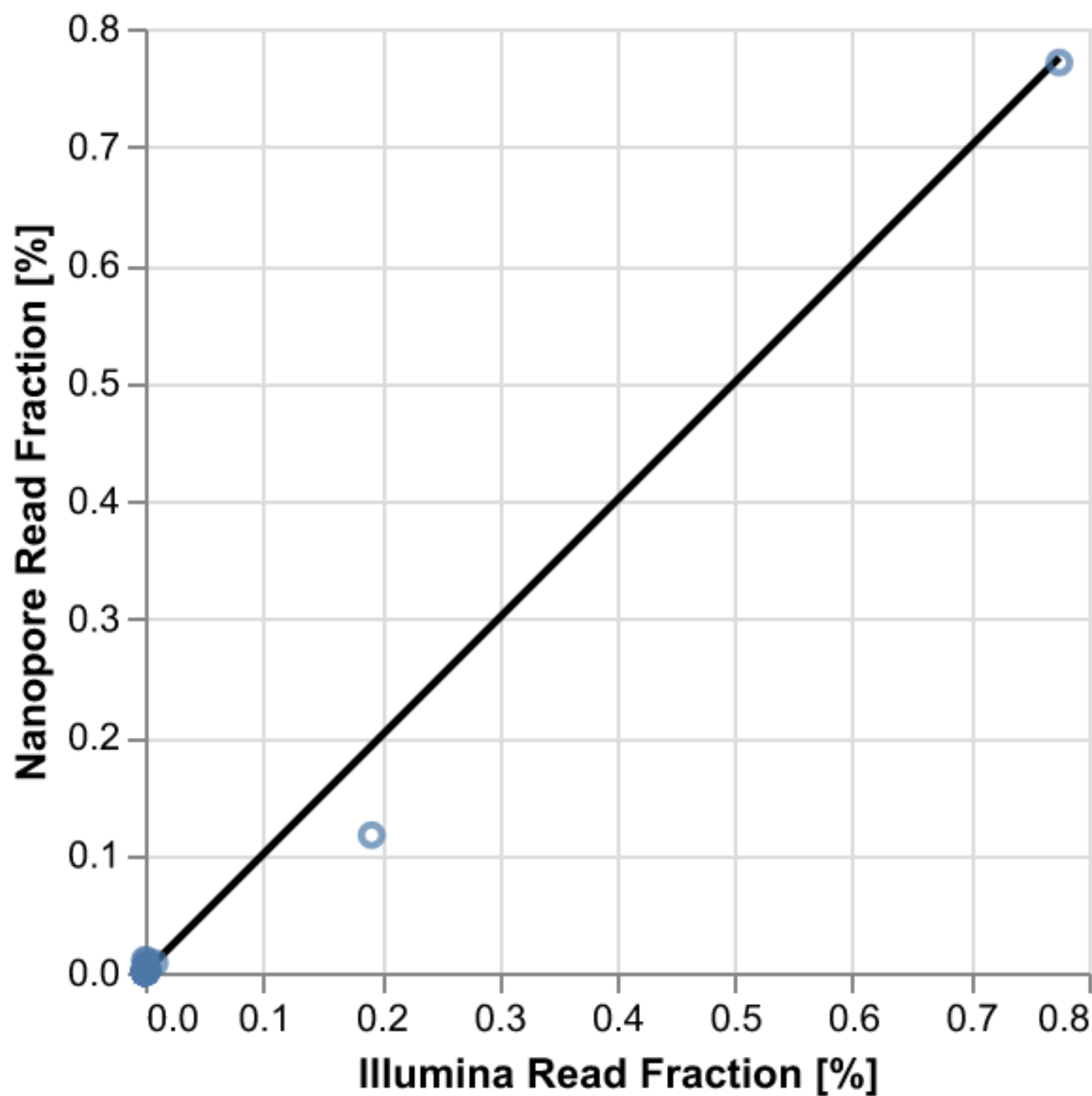

34\_10

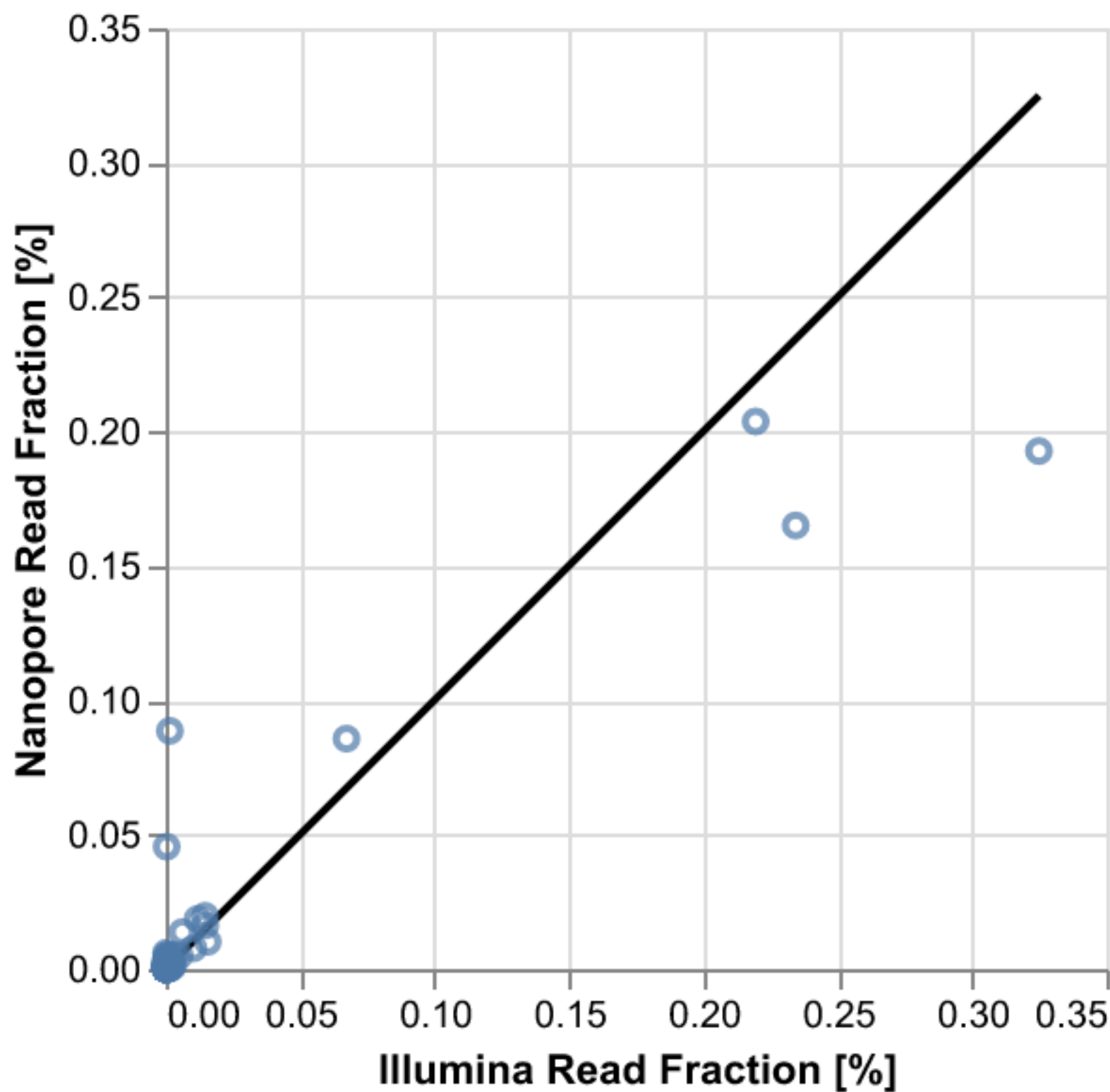

34\_18

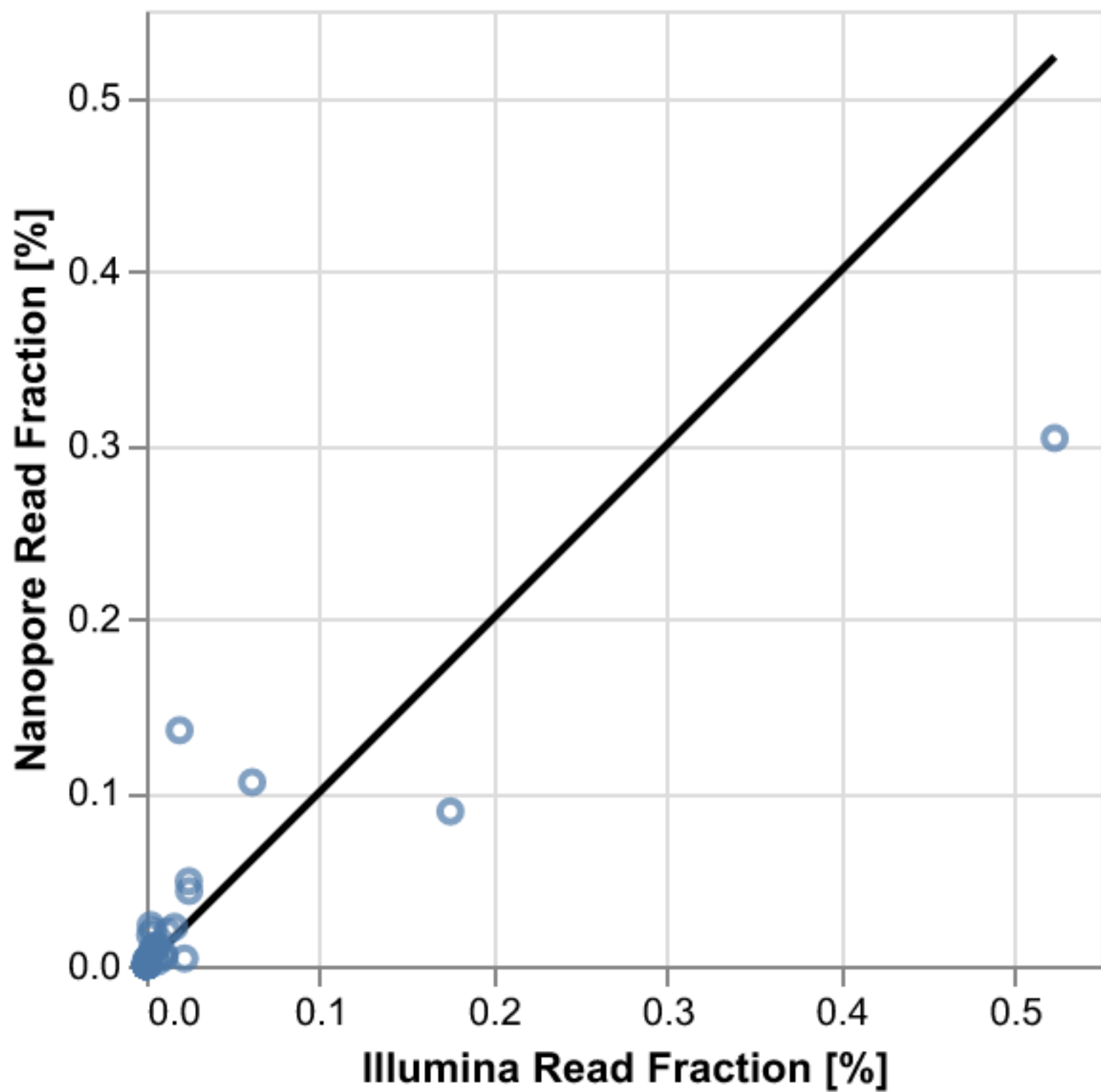

34\_41

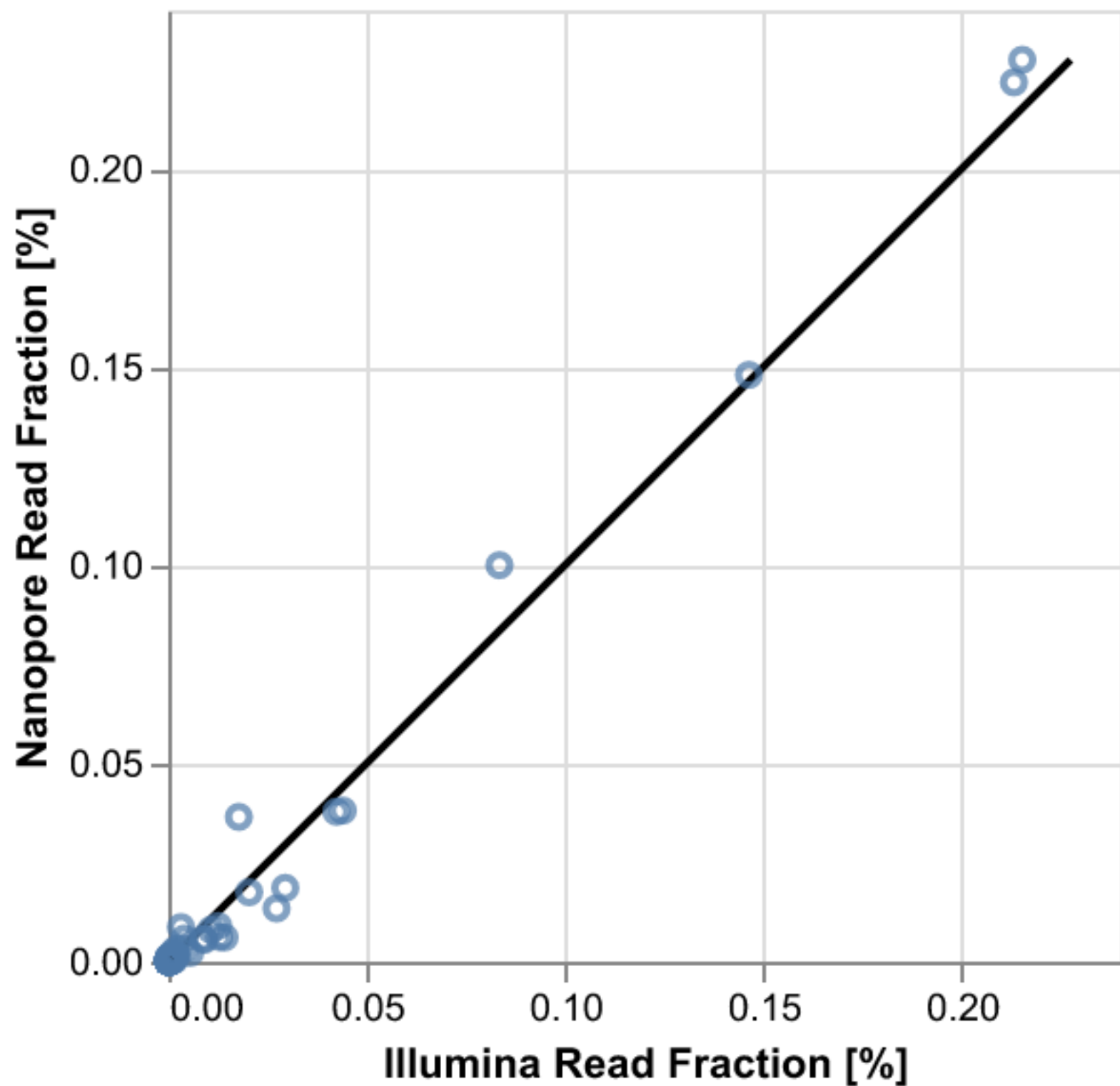

34\_-6

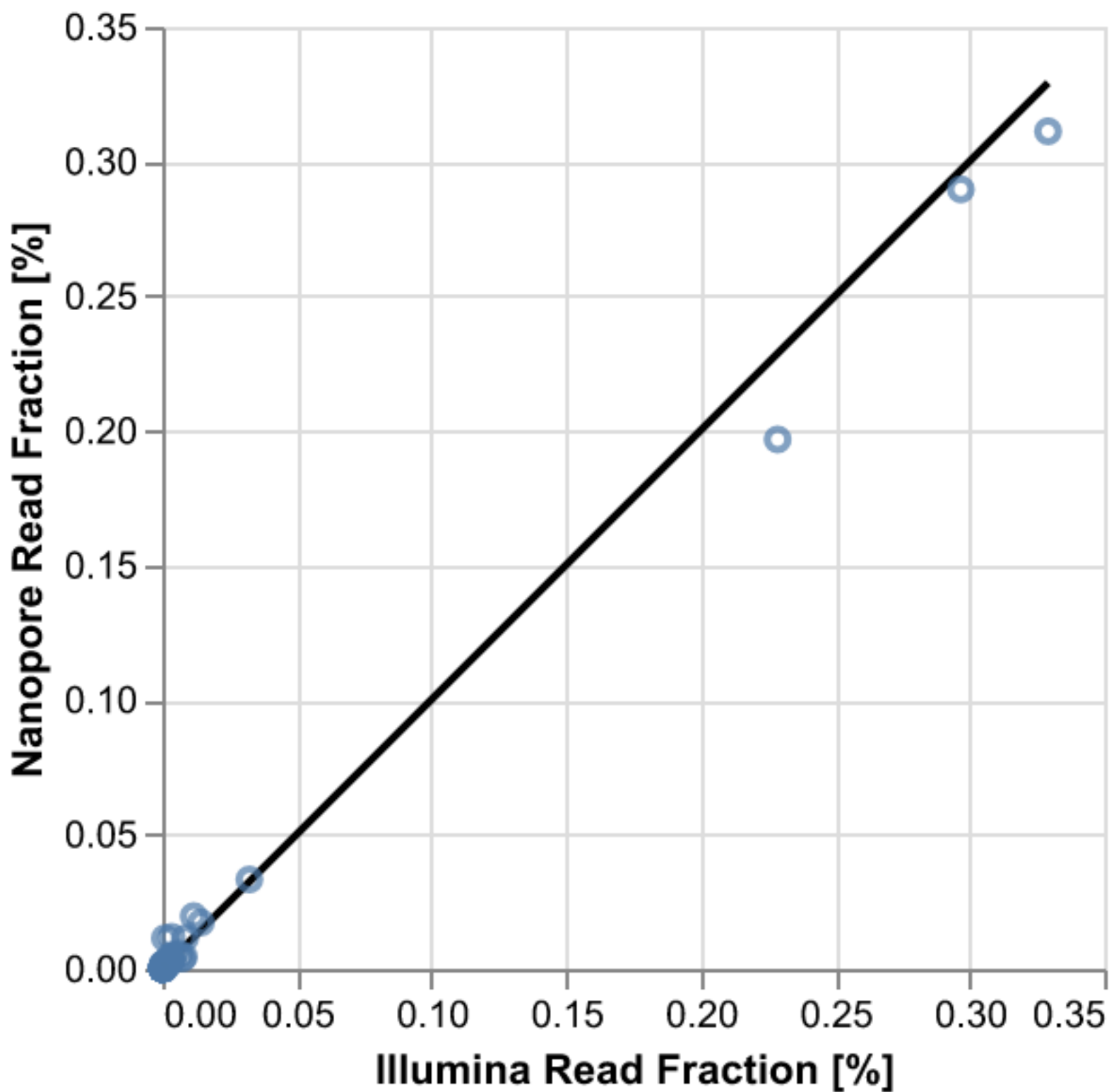

36\_-4

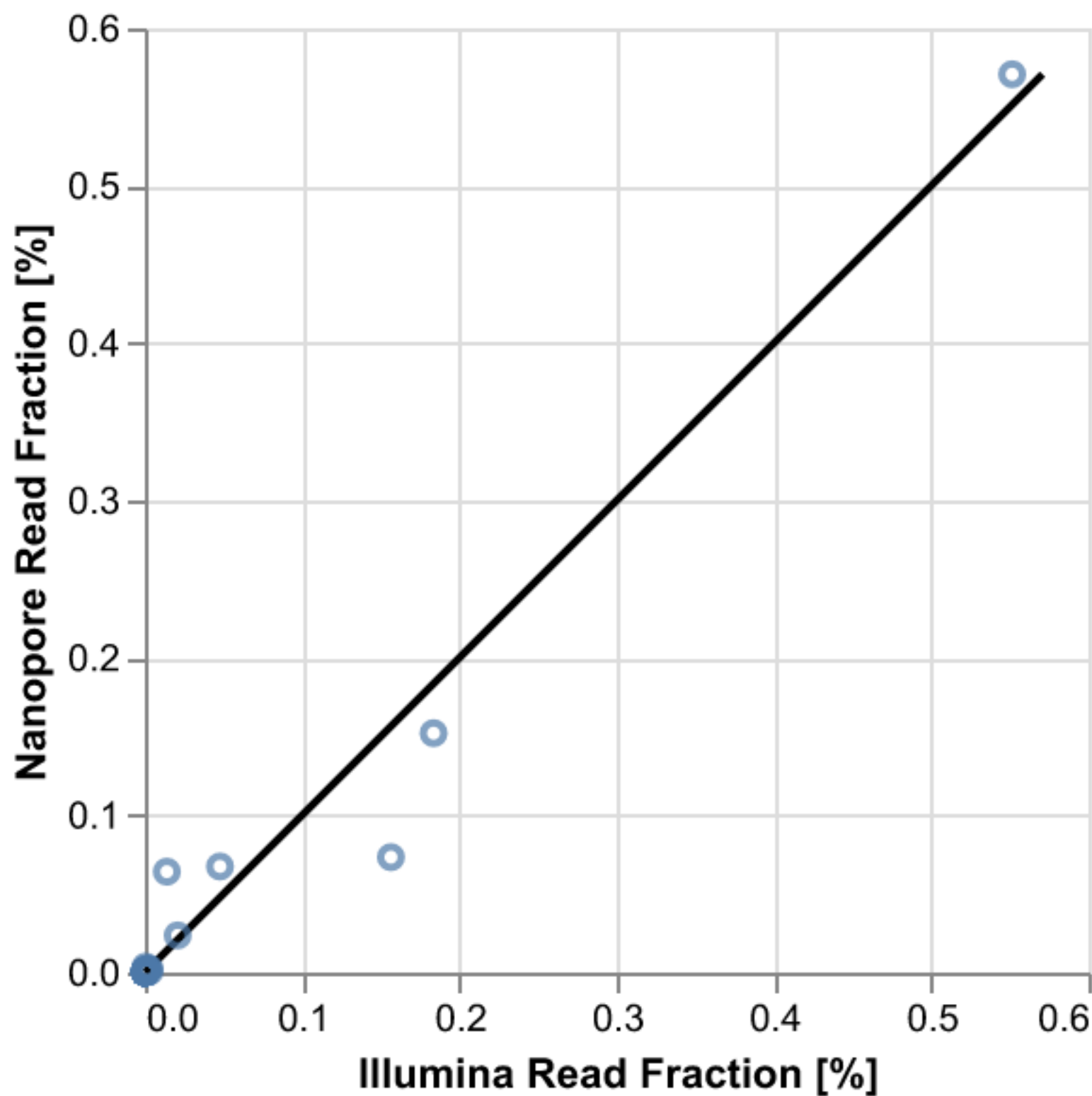

36\_63

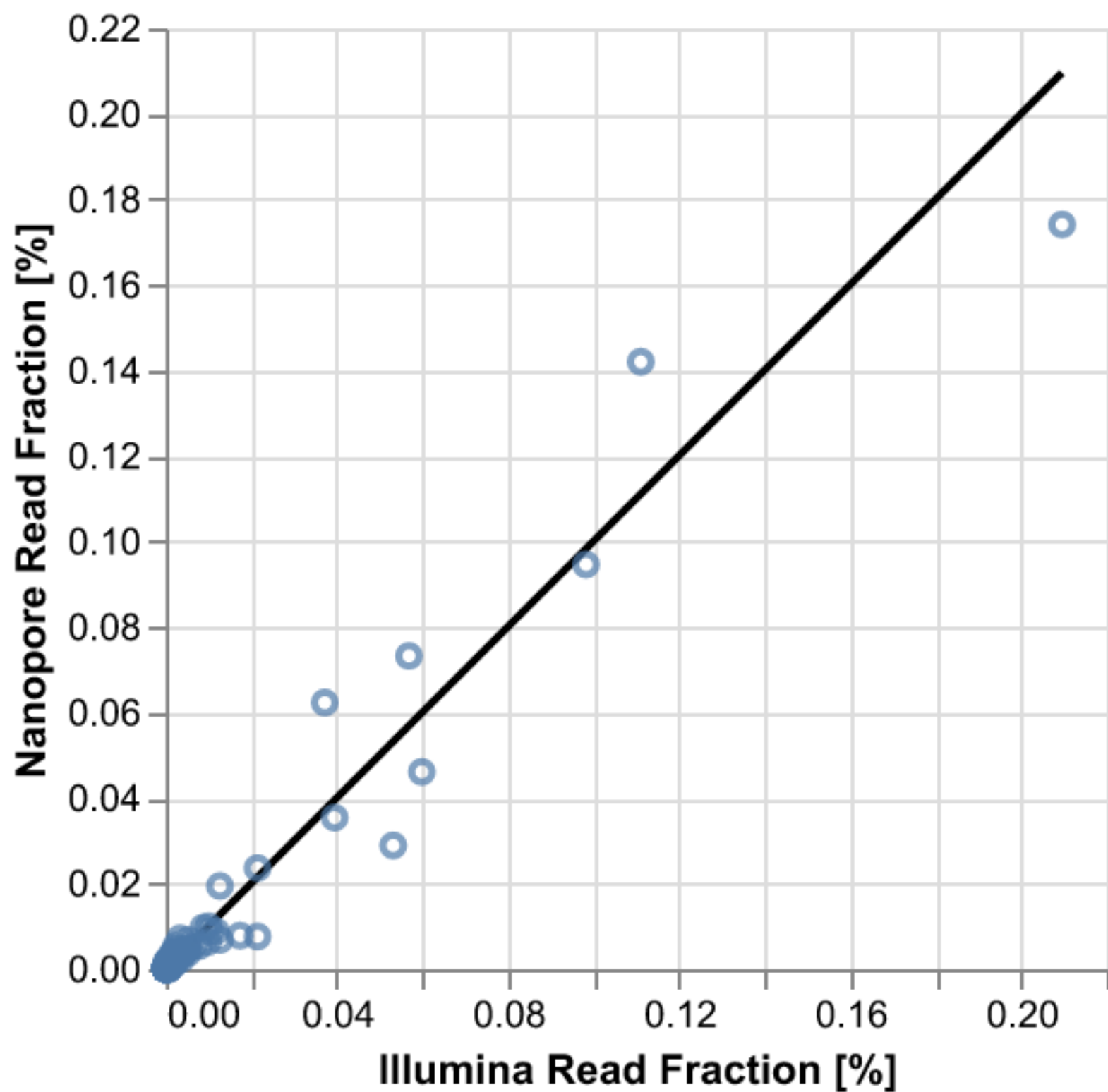

36\_-6

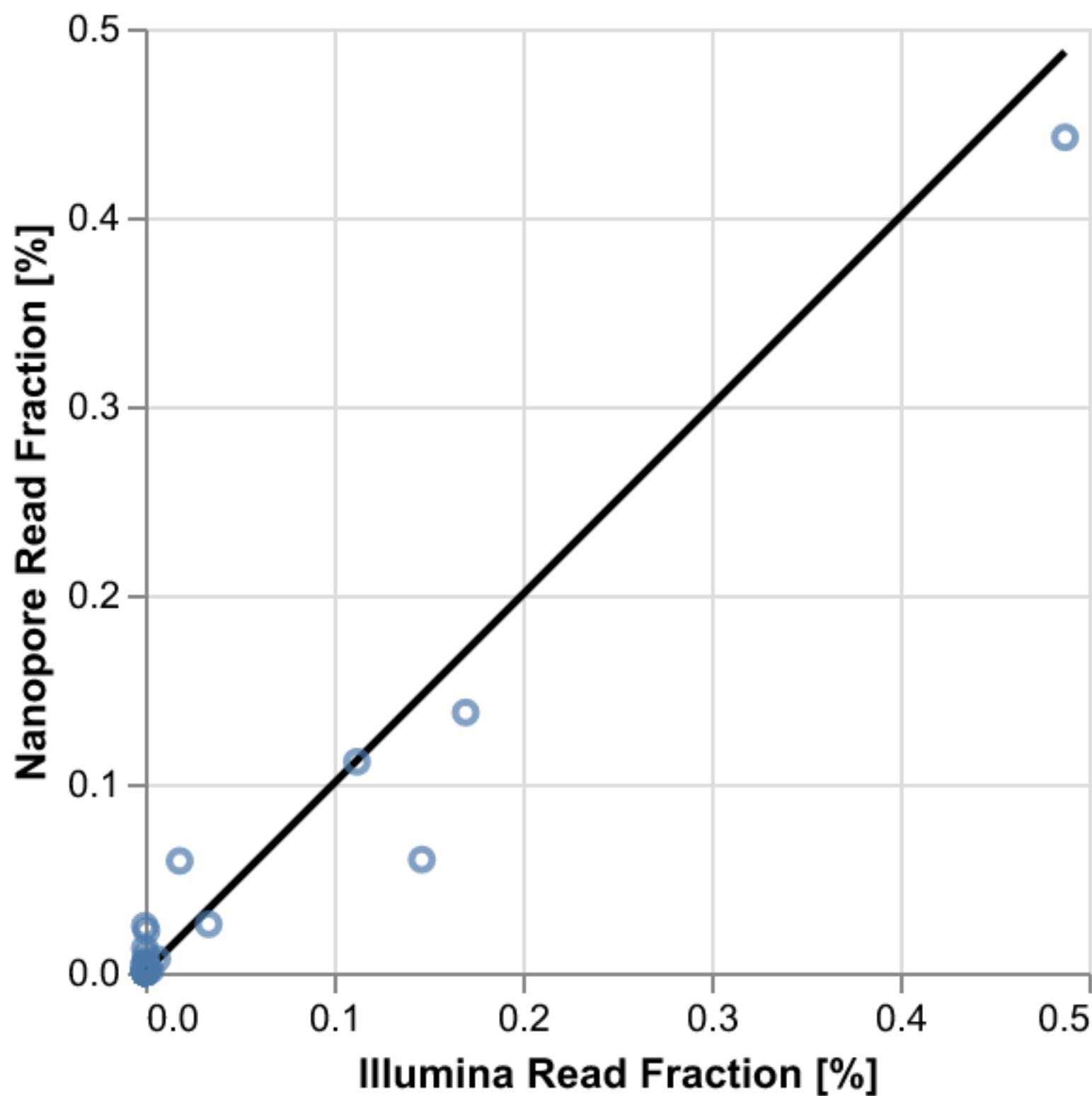

37\_138

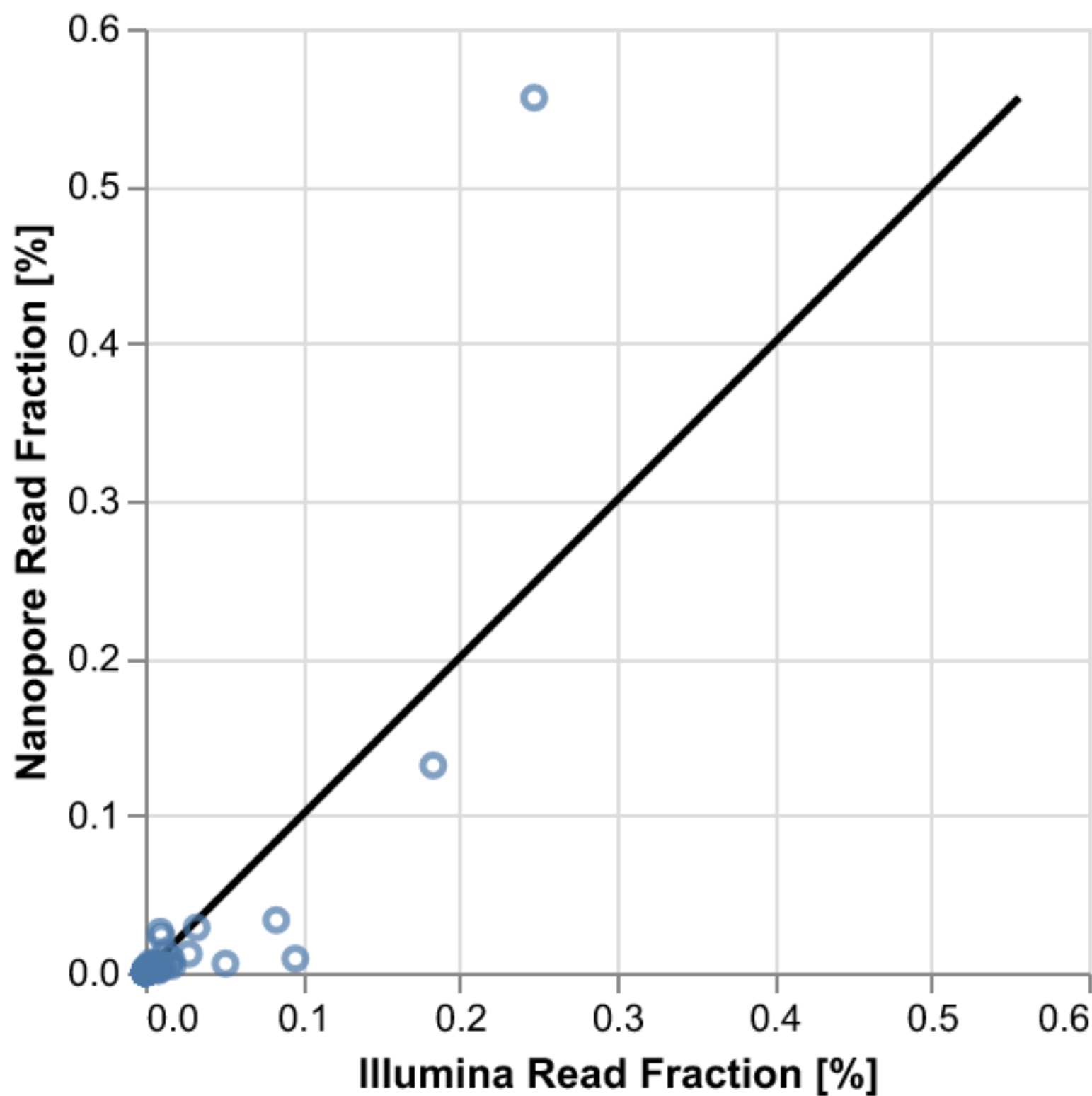

37\_1

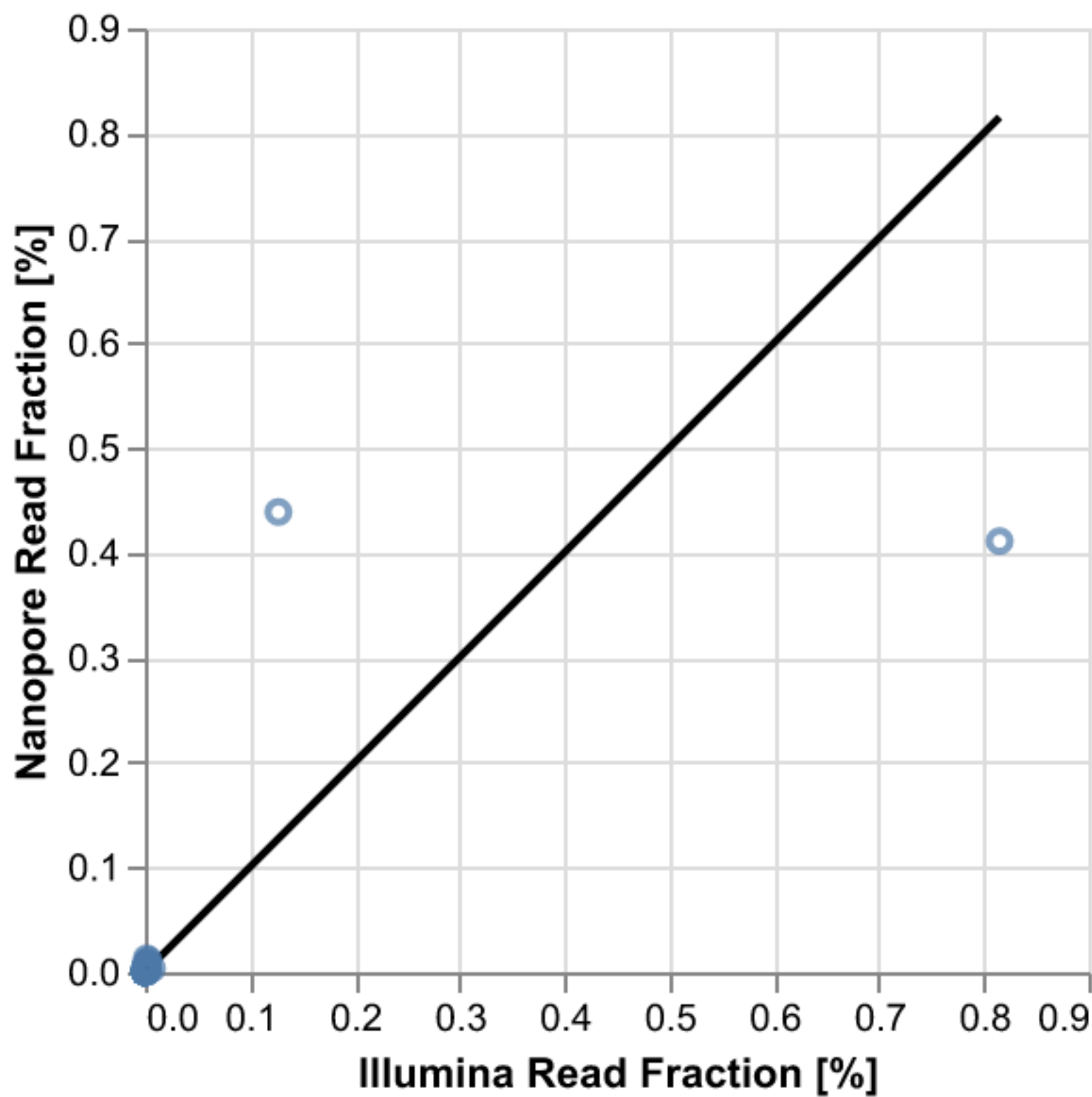

39\_115

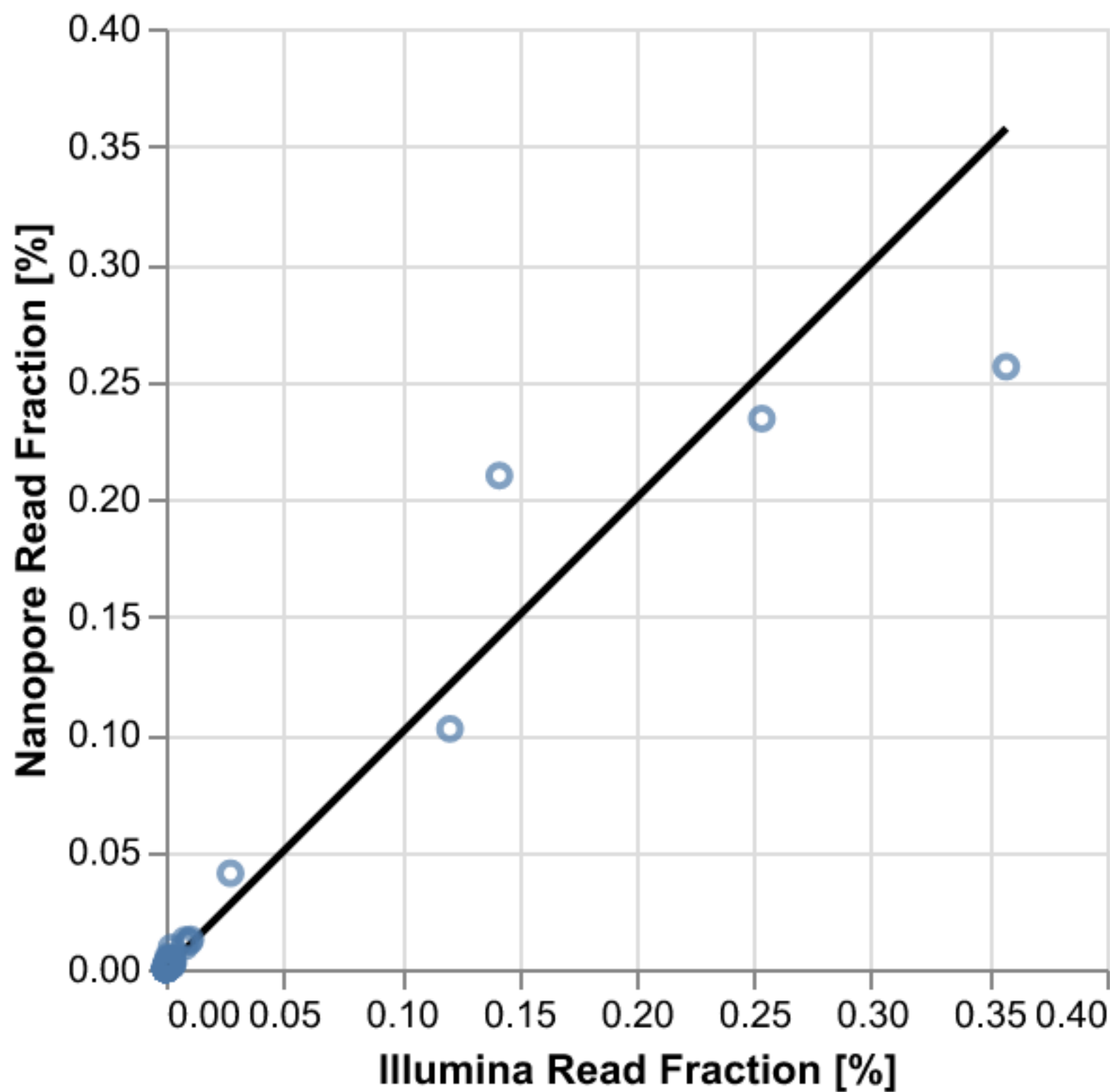

39\_15

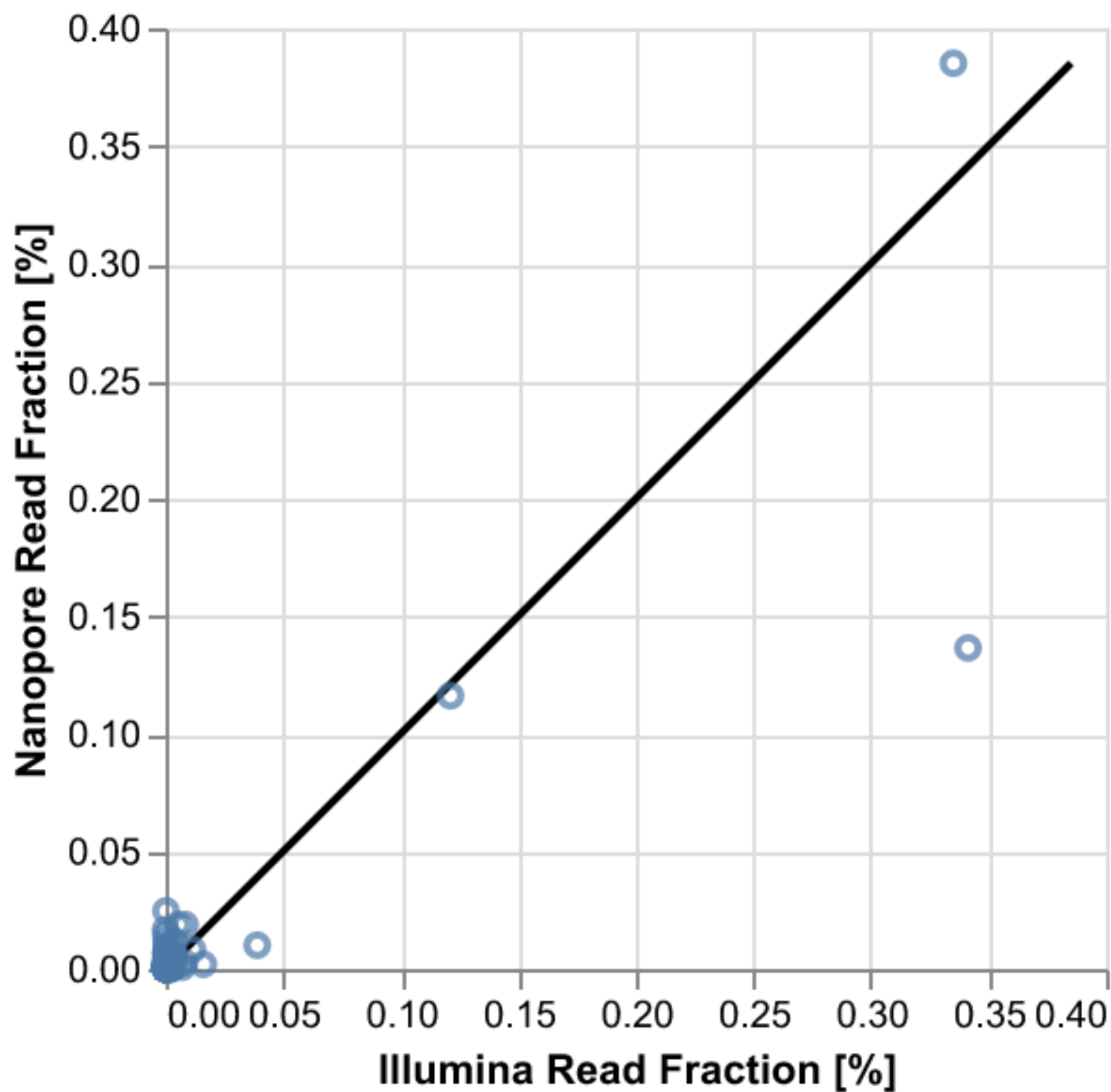

**39\_-6**

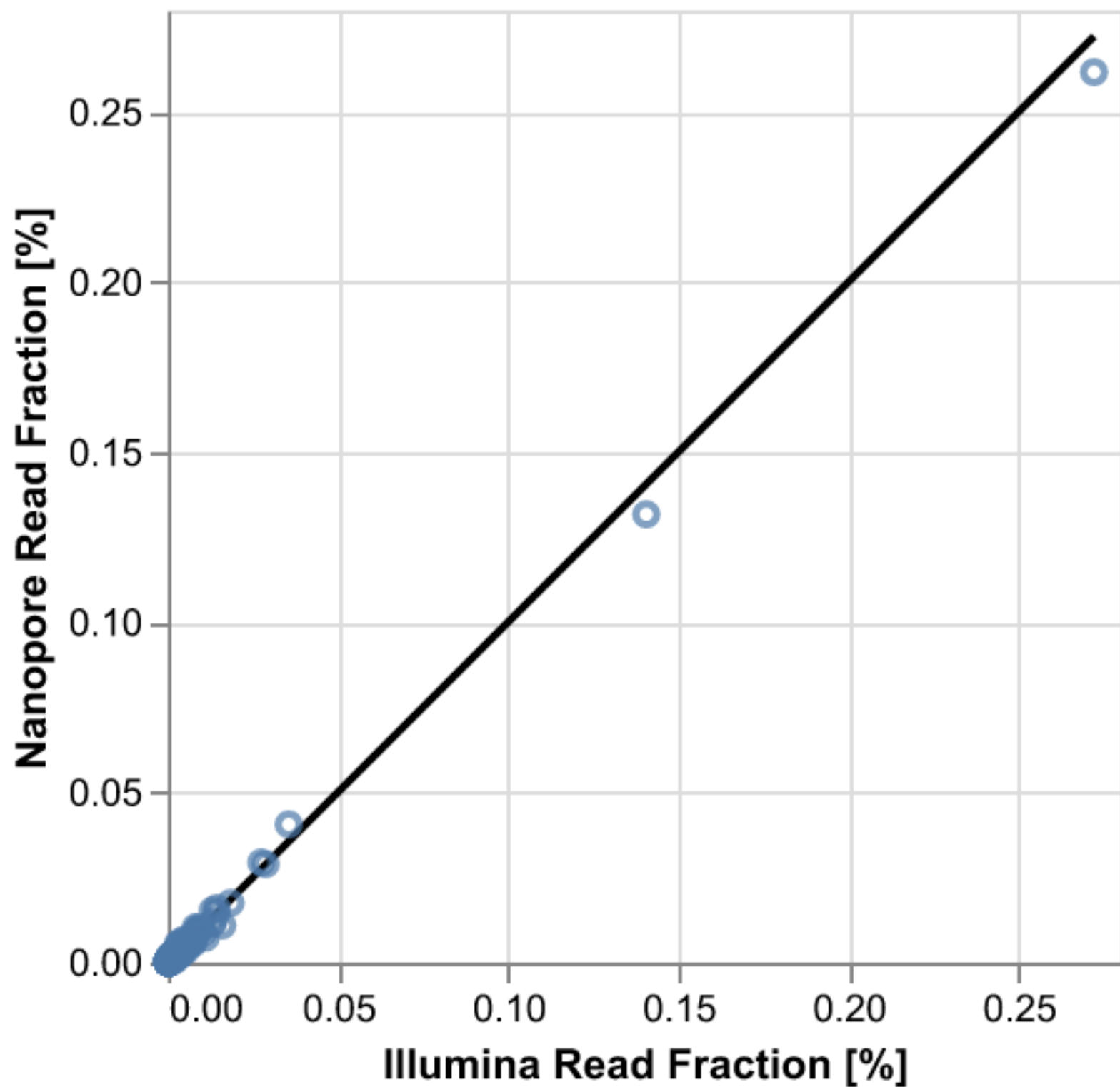

42\_18

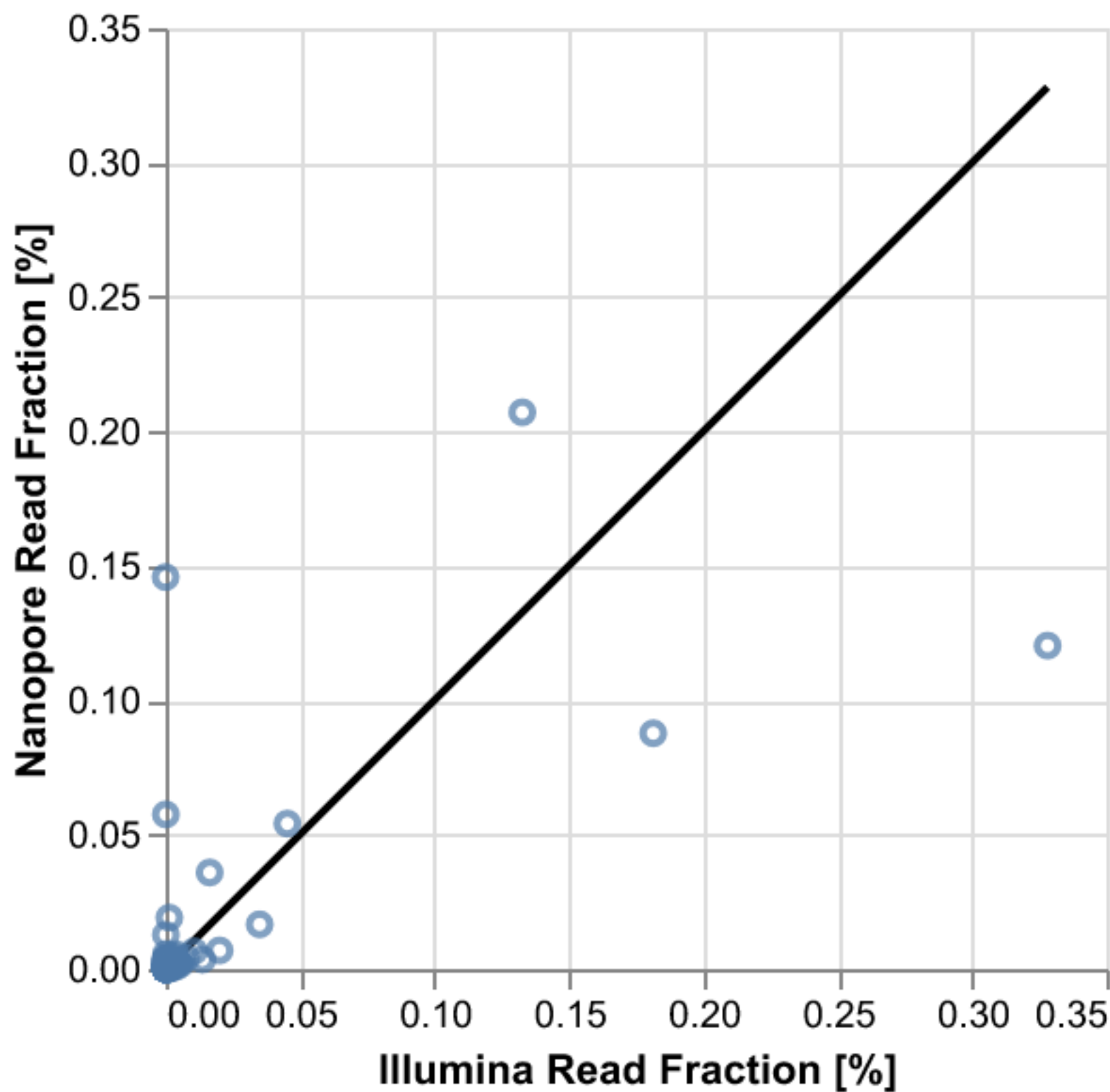

42\_30

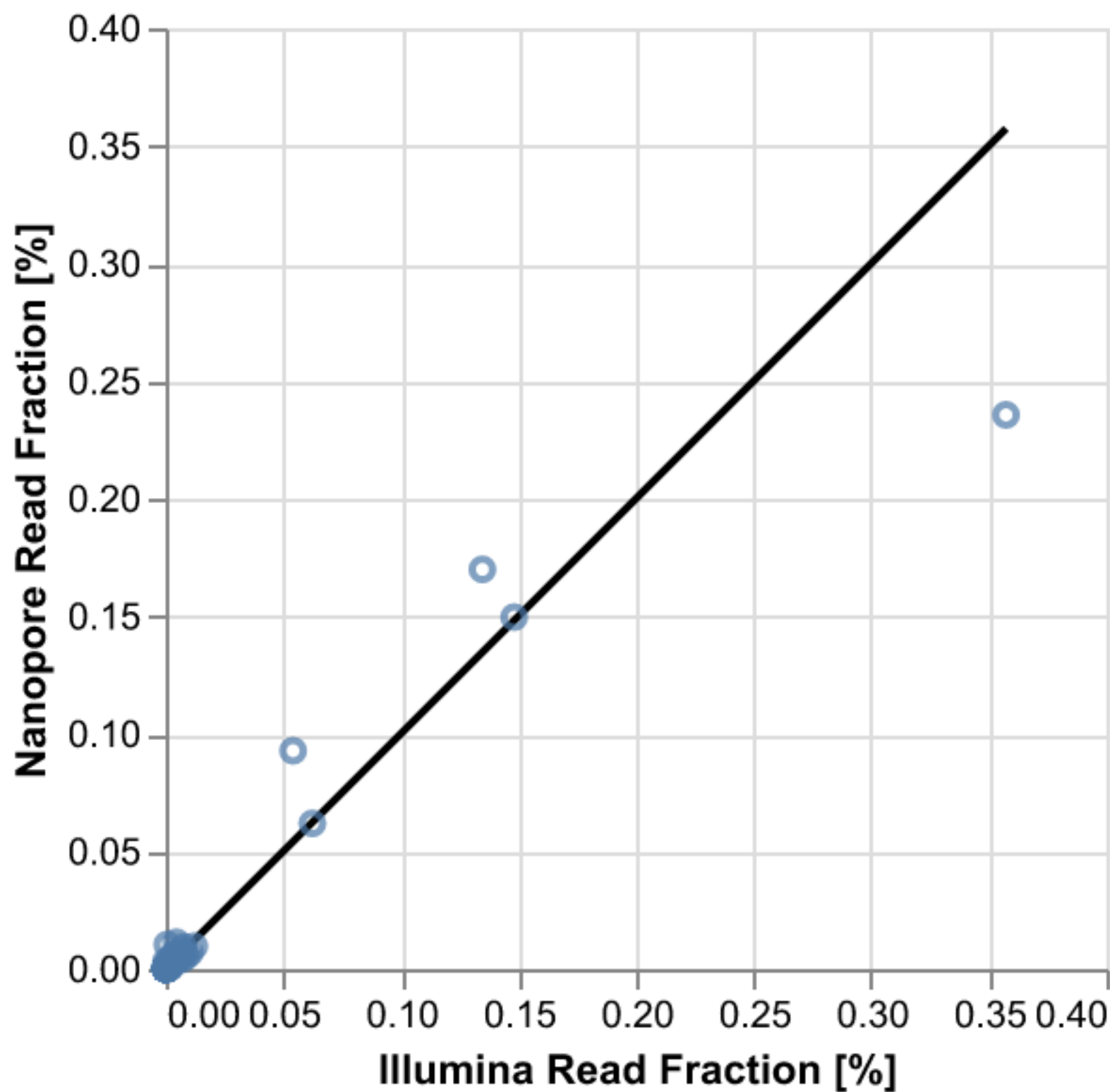

42\_6

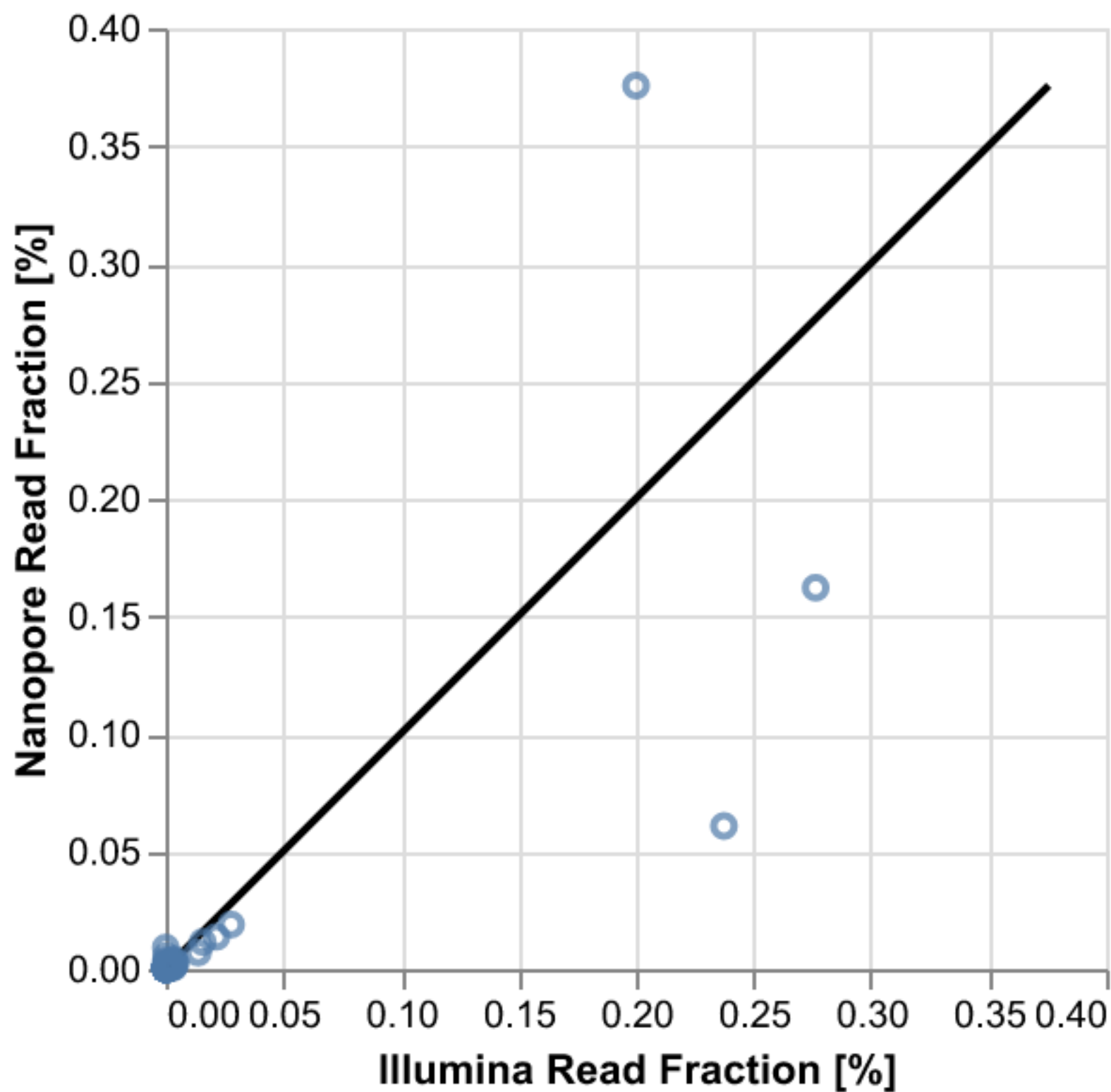

42\_-8

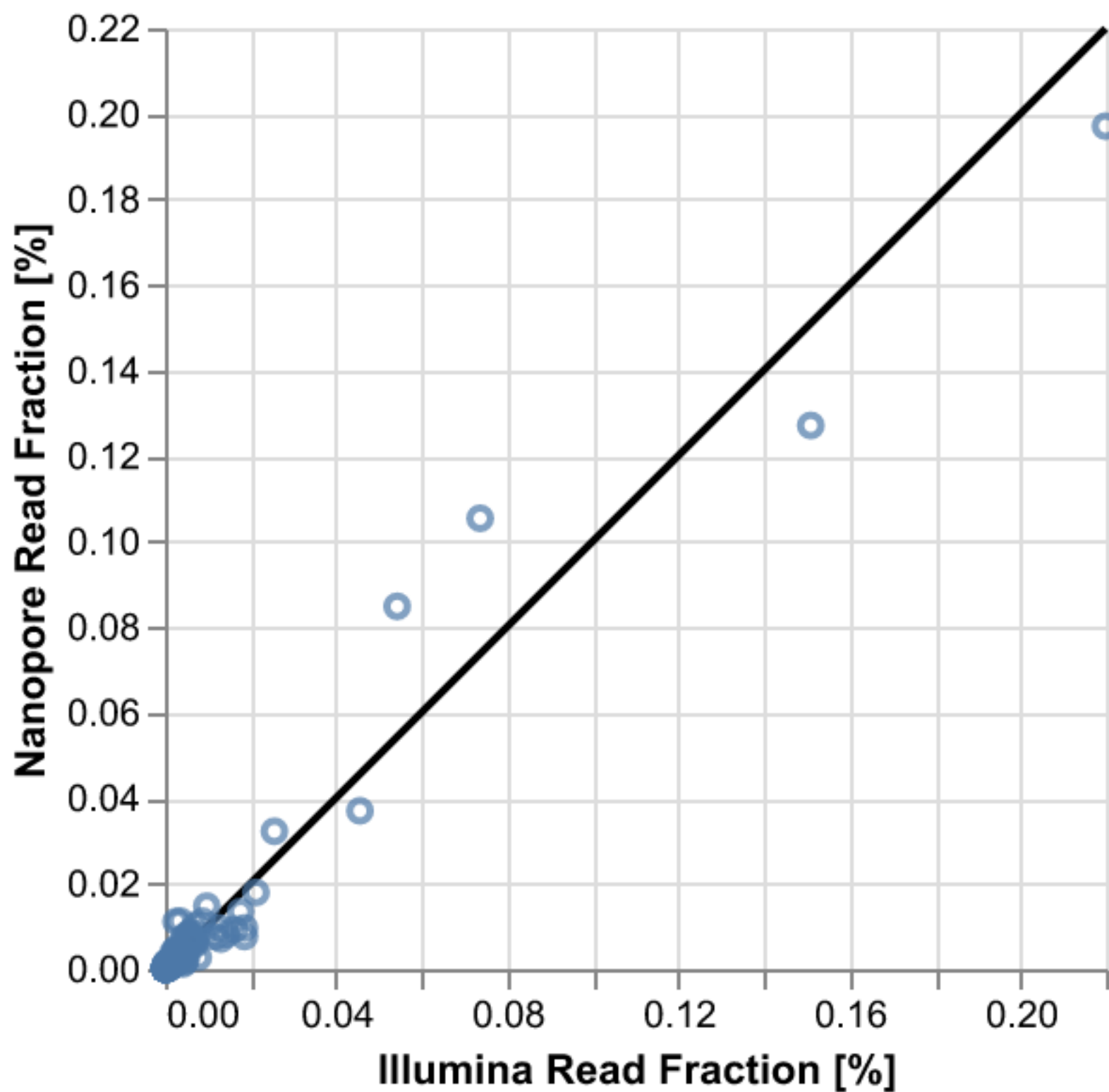

4\_-7

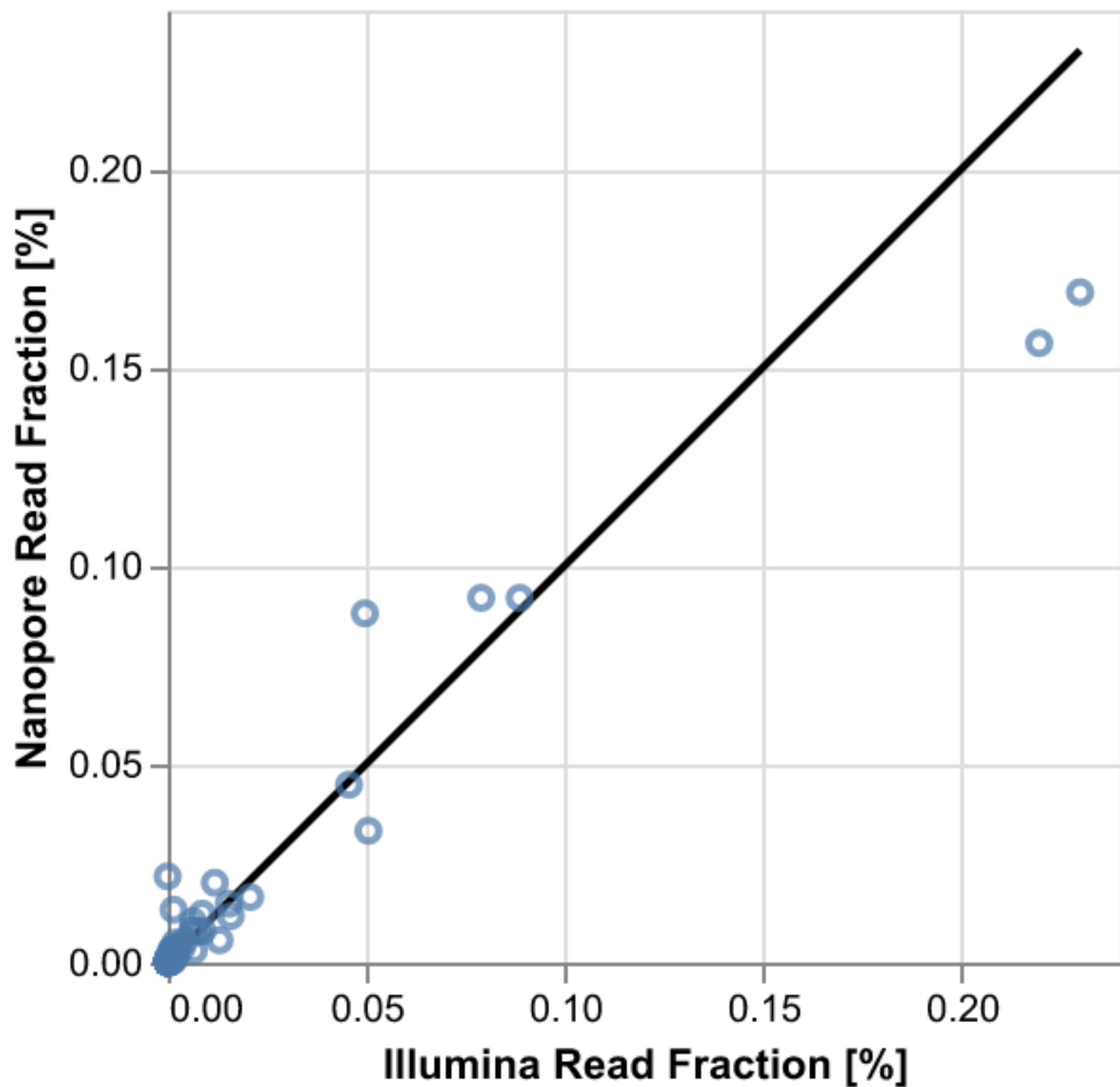

**7\_-6**

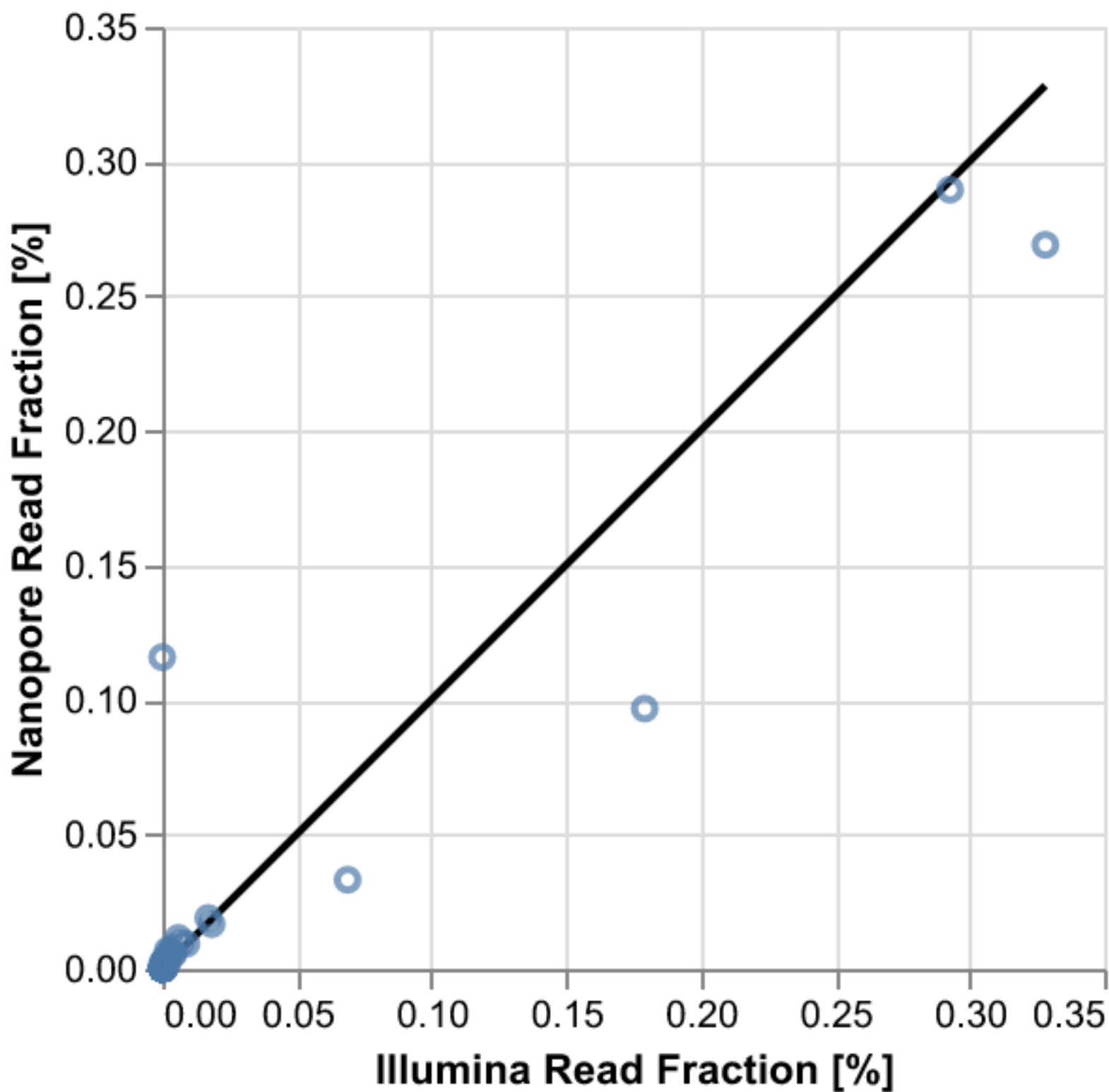

Supplement: Supplementary file 12 — Supplementary Figure 12. [file 41598_2024_53506_MOESM12_ESM.pdf]
