# Supplementary material for: Insights into gut microbiomes in stem cell transplantation by comprehensive shotgun long-read sequencing
Source: Sci Rep. 2024 Feb 19;14:4068. doi: 10.1038/s41598-024-53506-1 (PMC10876974; doi:10.1038/s41598-024-53506-1)
Supplement: Supplementary file 13 — Supplementary Information 1. [file 41598_2024_53506_MOESM13_ESM.doc]

100.00 30552518307 4308697 R 1 root

99.69 30457235506 13639487 R1 131567 cellular organisms

64.91 19831958554 7554489 D 2759 Eukaryota

45.10 13780392428 30938 K 33090 Viridiplantae

44.96 13737335351 0 P 35493 Streptophyta

44.96 13737335351 0 P1 131221 Streptophytina

44.96 13737335351 332867 P2 3193 Embryophyta

44.66 13646238065 0 P3 58023 Tracheophyta

44.66 13646238065 0 P4 78536 Euphyllophyta

44.66 13646238065 0 P5 58024 Spermatophyta

44.66 13646238065 469919 C 3398 Magnoliopsida

44.35 13549474658 12748697 C1 1437183 Mesangiospermae

27.22 8317445320 0 C2 71240 eudicotyledons

27.22 8317445320 0 C3 91827 Gunneridae

27.22 8317445320 9527151 C4 1437201 Pentapetalae

16.49 5037073219 4726945 C5 71275 rosids

11.65 3559634803 3774252 C6 91835 fabids

5.53 1689165536 0 O 72025 Fabales

5.53 1689165536 0 F 3803 Fabaceae

5.53 1689165536 0 F1 3814 Papilionoideae

5.53 1689165536 1961062 F2 2231393 50 kb inversion clade

4.05 1236771385 1313412 F3 2231382 NPAAA clade

2.09 638499597 0 F4 2233855 indigoferoid/millettioid clade

2.09 638499597 7602680 F5 163735 Phaseoleae

0.83 254958378 16535774 G 3913 Vigna

0.29 88793531 88793531 S 3917 Vigna unguiculata

0.25 75698578 0 S 157791 Vigna radiata

0.25 75698578 75698578 S1 3916 Vigna radiata var. radiata

0.24 73930495 73930495 S 3914 Vigna angularis

0.61 184926583 0 G 3846 Glycine

0.61 184926583 147676449 G1 1462606 Glycine subgen. Soja

0.06 19414884 19414884 S 3848 Glycine soja

0.06 17835250 17835250 S 3847 Glycine max

0.33 101631255 0 G 3820 Cajanus

0.33 101631255 101631255 S 3821 Cajanus cajan

0.29 89380701 0 G 3883 Phaseolus

0.29 89380701 89380701 S 3885 Phaseolus vulgaris

1.95 596958376 0 F4 2233838 Hologalegina

1.95 596958376 2713801 F5 2233839 IRL clade

1.16 354138201 0 F6 163743 Fabeae

1.16 354138201 0 G 3887 Pisum

1.16 354138201 354138201 S 3888 Pisum sativum

0.51 156807875 840782 F6 163742 Trifolieae

0.28 86646438 0 G 3877 Medicago

0.28 86646438 86646438 S 3880 Medicago truncatula

0.23 69320655 0 G 3898 Trifolium

0.23 69320655 69320655 S 57577 Trifolium pratense

0.27 83298499 0 F6 163722 Cicereae

0.27 83298499 0 G 3826 Cicer

0.27 83298499 83298499 S 3827 Cicer arietinum

1.05 321344317 0 F3 2231387 dalbergioids sensu lato

1.05 321344317 0 F4 163725 Dalbergieae

1.05 321344317 0 F5 2231390 Pterocarpus clade

1.05 321344317 242202514 G 3817 Arachis

0.14 41911015 41911015 S 3818 Arachis hypogaea

0.10 31592480 31592480 S 130453 Arachis duranensis

0.02 5638308 5638308 S 130454 Arachis ipaensis

0.42 129088772 0 F3 2231384 genistoids sensu lato

0.42 129088772 0 F4 2231385 core genistoids

0.42 129088772 0 F5 163729 Genisteae

0.42 129088772 0 G 3869 Lupinus

0.42 129088772 129088772 S 3871 Lupinus angustifolius

2.10 642560862 229217 O 3744 Rosales

1.50 458169104 639202 F 3745 Rosaceae

0.86 262863626 841631 F1 171637 Amygdaloideae

0.52 157870584 0 F2 721813 Maleae

0.52 157870584 83577662 G 3749 Malus

0.12 37239360 37239360 S 3750 Malus domestica

0.12 37053562 37053562 S 3752 Malus sylvestris

0.34 104151411 0 F2 721805 Amygdaleae

0.34 104151411 30364980 G 3754 Prunus

0.11 32162641 32162641 S 102107 Prunus mume

0.07 21337283 21337283 S 3760 Prunus persica

0.07 20286507 20286507 S 3755 Prunus dulcis

0.64 194666276 2420889 F1 171638 Rosoideae

0.32 97166291 619467 F2 721789 Potentilleae

0.16 48414355 0 F3 1184124 Fragariinae

0.16 48414355 0 G 3746 Fragaria

0.16 48414355 0 S 57918 Fragaria vesca

0.16 48414355 48414355 S1 101020 Fragaria vesca subsp. vesca

0.16 48132469 0 F3 1184125 Potentilleae incertae sedis

0.16 48132469 0 G 23204 Potentilla

0.16 48132469 48132469 S 57926 Potentilla anserina

0.31 95079096 0 F2 1176516 Rosoideae incertae sedis

0.31 95079096 0 G 3764 Rosa

0.31 95079096 95079096 S 74649 Rosa chinensis

0.35 107192265 0 F 3481 Cannabaceae

0.35 107192265 0 G 3482 Cannabis

0.35 107192265 107192265 S 3483 Cannabis sativa

0.25 76970276 0 F 3608 Rhamnaceae

0.25 76970276 0 F1 325284 Paliureae

0.25 76970276 0 G 72171 Ziziphus

0.25 76970276 0 S 326968 Ziziphus jujuba

0.25 76970276 76970276 S1 714518 Ziziphus jujuba var. spinosa

1.83 558084868 434874 O 3502 Fagales

1.07 328098043 16237249 F 16714 Juglandaceae

0.64 196270801 21570838 G 16718 Juglans

0.29 88075796 88075796 S 51240 Juglans regia

0.28 86624167 86624167 S 2249226 Juglans microcarpa x Juglans regia

0.38 115589993 0 G 13402 Carya

0.38 115589993 115589993 S 32201 Carya illinoinensis

0.75 229551951 0 F 3503 Fagaceae

0.75 229551951 71951941 G 3511 Quercus

0.27 83373359 83373359 S 97700 Quercus lobata

0.24 74226651 74226651 S 38942 Quercus robur

1.04 316292183 171304 O 3646 Malpighiales

0.74 227522169 238104 F 3977 Euphorbiaceae

0.45 137896736 0 F1 235629 Acalyphoideae

0.45 137896736 173513 F2 235880 Acalypheae

0.24 72113632 0 G 3984 Mercurialis

0.24 72113632 72113632 S 3986 Mercurialis annua

0.21 65609591 0 G 3987 Ricinus

0.21 65609591 65609591 S 3988 Ricinus communis

0.29 89387329 0 F1 235631 Crotonoideae

0.29 89387329 0 F2 235883 Manihoteae

0.29 89387329 0 G 3982 Manihot

0.29 89387329 89387329 S 3983 Manihot esculenta

0.29 88598710 0 F 3688 Salicaceae

0.29 88598710 0 F1 238069 Saliceae

0.29 88598710 0 G 3689 Populus

0.29 88598710 88598710 S 3694 Populus trichocarpa

0.92 280668962 0 O 71239 Cucurbitales

0.92 280668962 1520940 F 3650 Cucurbitaceae

0.74 224858270 1463630 F1 1003877 Benincaseae

0.57 173681222 0 G 102210 Benincasa

0.57 173681222 173681222 S 102211 Benincasa hispida

0.16 49713418 0 G 3655 Cucumis

0.16 49713418 49713418 S 3659 Cucumis sativus

0.18 54289752 0 F1 1003878 Cucurbiteae

0.18 54289752 0 G 3660 Cucurbita

0.18 54289752 0 S 3663 Cucurbita pepo

0.18 54289752 54289752 S1 3664 Cucurbita pepo subsp. pepo

0.23 69088140 0 O 233875 Celastrales

0.23 69088140 0 F 4305 Celastraceae

0.23 69088140 0 G 123484 Tripterygium

0.23 69088140 69088140 S 458696 Tripterygium wilfordii

4.34 1325608956 417598 C6 91836 malvids

1.84 561964757 0 O 41938 Malvales

1.84 561964757 572390 F 3629 Malvaceae

1.62 493731307 0 F1 214907 Malvoideae

1.62 493731307 206099644 G 3633 Gossypium

0.47 143707389 143707389 S 3635 Gossypium hirsutum

0.27 81919879 81919879 S 29729 Gossypium arboreum

0.20 62004395 62004395 S 29730 Gossypium raimondii

0.22 67661060 0 F1 214909 Byttnerioideae

0.22 67661060 0 G 3640 Theobroma

0.22 67661060 67661060 S 3641 Theobroma cacao

1.07 327119636 0 O 3699 Brassicales

1.07 327119636 1463681 F 3700 Brassicaceae

0.62 188715386 0 F1 981071 Brassiceae

0.62 188715386 99191853 G 3705 Brassica

0.15 46995517 46995517 S 3708 Brassica napus

0.08 25421911 0 S 3712 Brassica oleracea

0.08 25421911 25421911 S1 109376 Brassica oleracea var. oleracea

0.06 17106105 17106105 S 3711 Brassica rapa

0.45 136940569 1658900 F1 980083 Camelineae

0.35 105489959 0 G 71323 Camelina

0.35 105489959 105489959 S 90675 Camelina sativa

0.10 29791710 0 G 3701 Arabidopsis

0.10 29791710 29791710 S 3702 Arabidopsis thaliana

0.93 285073038 32087 O 41944 Myrtales

0.71 216380740 0 F 3931 Myrtaceae

0.71 216380740 2193714 F1 1699513 Myrtoideae

0.44 133463796 0 F2 1699524 Eucalypteae

0.44 133463796 0 G 3932 Eucalyptus

0.44 133463796 133463796 S 71139 Eucalyptus grandis

0.26 80723230 0 F2 1699523 Myrteae

0.26 80723230 0 F3 1705102 Australasian group

0.26 80723230 0 G 178132 Rhodamnia

0.26 80723230 80723230 S 178133 Rhodamnia argentea

0.22 68660211 0 F 3928 Lythraceae

0.22 68660211 0 G 22662 Punica

0.22 68660211 68660211 S 22663 Punica granatum

0.49 151033927 93312 O 41937 Sapindales

0.29 88240558 0 F 4011 Anacardiaceae

0.29 88240558 0 G 23461 Mangifera

0.29 88240558 88240558 S 29780 Mangifera indica

0.21 62700057 0 F 23513 Rutaceae

0.21 62700057 0 F1 1728959 Aurantioideae

0.21 62700057 0 G 2706 Citrus

0.21 62700057 62700057 S 2711 Citrus sinensis

0.48 147102515 0 C6 91834 rosids incertae sedis

0.48 147102515 0 O 403667 Vitales

0.48 147102515 0 F 3602 Vitaceae

0.48 147102515 0 F1 2304100 Viteae

0.48 147102515 46505643 G 3603 Vitis

0.17 51623165 51623165 S 96939 Vitis riparia

0.16 48973707 48973707 S 29760 Vitis vinifera

10.35 3161267098 1194001 C5 71274 asterids

6.89 2103558146 595335 C6 91888 lamiids

4.84 1477954045 99614 O 4069 Solanales

4.55 1390790851 2624947 F 4070 Solanaceae

3.51 1071474172 3484401 F1 424551 Solanoideae

2.05 624951742 0 F2 424564 Capsiceae

2.05 624951742 0 G 4071 Capsicum

2.05 624951742 624951742 S 4072 Capsicum annuum

1.45 443038029 0 F2 424574 Solaneae

1.45 443038029 14062695 G 4107 Solanum

0.95 289417099 42646682 G1 49274 Solanum subgen. Lycopersicon

0.43 131205969 131205969 S 28526 Solanum pennellii

0.38 115564448 115564448 S 4081 Solanum lycopersicum

0.46 139558235 139558235 S 172797 Solanum stenotomum

1.04 316691732 0 F1 424554 Nicotianoideae

1.04 316691732 0 F2 424562 Nicotianeae

1.04 316691732 0 G 4085 Nicotiana

1.04 316691732 316691732 S 49451 Nicotiana attenuata

0.28 87063580 0 F 4118 Convolvulaceae

0.28 87063580 0 F1 267213 Ipomoeeae

0.28 87063580 0 G 4119 Ipomoea

0.28 87063580 87063580 S 35885 Ipomoea triloba

1.47 448112922 235421 O 4143 Lamiales

0.65 197907149 0 F 4144 Oleaceae

0.65 197907149 0 F1 426106 Oleeae

0.65 197907149 0 G 4145 Olea

0.65 197907149 0 S 4146 Olea europaea

0.65 197907149 0 S1 158383 Olea europaea subsp. europaea

0.65 197907149 197907149 S2 158386 Olea europaea var. sylvestris

0.61 185629063 0 F 4136 Lamiaceae

0.61 185629063 0 F1 216706 Nepetoideae

0.61 185629063 0 F2 216718 Mentheae

0.61 185629063 0 F3 2836339 Salviinae

0.61 185629063 15119923 G 21880 Salvia

0.38 115036039 0 G1 2026555 Salvia subgen. Calosphace

0.38 115036039 0 G2 2026556 core Calosphace

0.38 115036039 115036039 S 180675 Salvia splendens

0.18 55473101 0 G1 2291027 Salvia incertae sedis

0.18 55473101 55473101 S 49212 Salvia hispanica

0.21 64341289 0 F 4180 Pedaliaceae

0.21 64341289 0 G 4181 Sesamum

0.21 64341289 64341289 S 4182 Sesamum indicum

0.58 176895844 0 O 4055 Gentianales

0.58 176895844 0 F 24966 Rubiaceae

0.58 176895844 0 F1 169618 Ixoroideae

0.58 176895844 0 F2 1968429 Gardenieae complex

0.58 176895844 0 F3 1968428 Bertiereae - Coffeeae clade

0.58 176895844 0 F4 169640 Coffeeae

0.58 176895844 73892527 G 13442 Coffea

0.24 73457137 73457137 S 13443 Coffea arabica

0.10 29546180 29546180 S 49369 Coffea eugenioides

3.10 946882896 52373 C6 91882 campanulids

2.84 868427913 0 O 4209 Asterales

2.84 868427913 1206917 F 4210 Asteraceae

1.31 399576801 188447 F1 102804 Asteroideae

1.01 309923338 0 F2 911341 Heliantheae alliance

1.01 309923338 0 F3 102814 Heliantheae

1.01 309923338 0 G 4231 Helianthus

1.01 309923338 309923338 S 4232 Helianthus annuus

0.29 89465016 0 F2 102809 Astereae

0.29 89465016 0 F3 877976 North American clade

0.29 89465016 0 F4 2841728 Conyzinae

0.29 89465016 0 G 41574 Erigeron

0.29 89465016 89465016 S 72917 Erigeron canadensis

1.11 339770560 0 F1 219120 Cichorioideae

1.11 339770560 0 F2 219121 Cichorieae

1.11 339770560 0 F3 745062 Lactucinae

1.11 339770560 0 G 4235 Lactuca

1.11 339770560 339770560 S 4236 Lactuca sativa

0.42 127873635 0 F1 219103 Carduoideae

0.42 127873635 0 F2 102818 Cardueae

0.42 127873635 0 F3 742010 Carduinae

0.42 127873635 0 G 4264 Cynara

0.42 127873635 0 S 4265 Cynara cardunculus

0.42 127873635 0 S1 309979 Cynara cardunculus subsp. cardunculus

0.42 127873635 127873635 S2 59895 Cynara cardunculus var. scolymus

0.26 78402610 0 O 4036 Apiales

0.26 78402610 0 O1 364270 Apiineae

0.26 78402610 0 F 4037 Apiaceae

0.26 78402610 0 F1 241778 Apioideae

0.26 78402610 0 F2 241789 Scandiceae

0.26 78402610 0 F3 241799 Daucinae

0.26 78402610 0 G 4038 Daucus

0.26 78402610 0 G1 1873447 Daucus sect. Daucus

0.26 78402610 0 S 4039 Daucus carota

0.26 78402610 78402610 S1 79200 Daucus carota subsp. sativus

0.36 109632055 0 O 41945 Ericales

0.36 109632055 0 F 25692 Balsaminaceae

0.36 109632055 0 G 35939 Impatiens

0.36 109632055 109632055 S 253017 Impatiens glandulifera

0.36 109577852 0 O 3524 Caryophyllales

0.36 109577852 0 F 1804623 Chenopodiaceae

0.36 109577852 0 F1 1804621 Betoideae

0.36 109577852 0 G 3554 Beta

0.36 109577852 0 S 161934 Beta vulgaris

0.36 109577852 109577852 S1 3555 Beta vulgaris subsp. vulgaris

14.75 4506777339 0 C2 4447 Liliopsida

14.75 4506777339 403698 C3 1437197 Petrosaviidae

13.77 4207712588 831150 C4 4734 commelinids

11.18 3414675231 91379 O 38820 Poales

10.92 3336939105 3053642 F 4479 Poaceae

8.57 2618591724 726131 F1 359160 BOP clade

8.09 2470878047 3562963 F2 147368 Pooideae

6.90 2109238460 0 F3 1648038 Triticodae

6.90 2109238460 26451119 F4 147389 Triticeae

5.39 1645524840 317011678 F5 1648030 Triticinae

4.14 1266137438 580775553 G 4564 Triticum

0.98 299969669 299969669 S 4565 Triticum aestivum

0.79 241675595 241675595 S 85692 Triticum dicoccoides

0.47 143716621 143716621 S 4572 Triticum urartu

0.20 62375724 0 G 4480 Aegilops

0.20 62375724 0 S 37682 Aegilops tauschii

0.20 62375724 62375724 S1 200361 Aegilops tauschii subsp. strangulata

1.43 437262501 0 F5 1648017 Hordeinae

1.43 437262501 0 G 4512 Hordeum

1.43 437262501 0 S 4513 Hordeum vulgare

1.43 437262501 437262501 S1 112509 Hordeum vulgare subsp. vulgare

0.95 289761262 0 F3 1648037 Poodae

0.95 289761262 0 F4 147387 Poeae

0.95 289761262 0 F5 1652081 Poeae Chloroplast Group 2 (Poeae type)

0.95 289761262 0 F6 2948571 Loliodinae

0.95 289761262 0 F7 640630 Loliinae

0.95 289761262 0 G 4520 Lolium

0.95 289761262 289761262 S 89674 Lolium rigidum

0.22 68315362 0 F3 2822797 Stipodae

0.22 68315362 0 F4 147385 Brachypodieae

0.22 68315362 0 G 15367 Brachypodium

0.22 68315362 68315362 S 15368 Brachypodium distachyon

0.48 146987546 0 F2 147367 Oryzoideae

0.48 146987546 0 F3 147380 Oryzeae

0.48 146987546 0 F4 1648021 Oryzinae

0.48 146987546 1542513 G 4527 Oryza

0.27 81023257 0 S 4530 Oryza sativa

0.27 81023257 81023257 S1 39947 Oryza sativa Japonica Group

0.21 64421776 64421776 S 4533 Oryza brachyantha

2.34 715293739 0 F1 147370 PACMAD clade

2.34 715293739 2609745 F2 147369 Panicoideae

1.28 391653043 0 F3 1648036 Panicodae

1.28 391653043 3064101 F4 147428 Paniceae

0.89 272665190 0 F5 1293365 Panicinae

0.89 272665190 7141306 G 4539 Panicum

0.60 184048083 0 G1 2100771 Panicum sect. Hiantes

0.60 184048083 184048083 S 38727 Panicum virgatum

0.27 81475801 0 G1 2100772 Panicum sect. Panicum

0.27 81475801 81475801 S 206008 Panicum hallii

0.38 115923752 0 F5 1293361 Cenchrinae

0.38 115923752 63397892 G 4554 Setaria

0.09 26465037 26465037 S 4555 Setaria italica

0.09 26060823 26060823 S 4556 Setaria viridis

1.05 321030951 0 F3 1648033 Andropogonodae

1.05 321030951 1960679 F4 147429 Andropogoneae

0.70 213794924 0 F5 1648029 Tripsacinae

0.70 213794924 0 G 4575 Zea

0.70 213794924 213794924 S 4577 Zea mays

0.34 105275348 0 F5 1648028 Sorghinae

0.34 105275348 0 G 4557 Sorghum

0.34 105275348 105275348 S 4558 Sorghum bicolor

0.25 77644747 0 F 4613 Bromeliaceae

0.25 77644747 0 F1 1909378 Bromelioideae

0.25 77644747 0 G 4614 Ananas

0.25 77644747 77644747 S 4615 Ananas comosus

1.45 443073926 138682 O 4618 Zingiberales

1.15 351693223 0 F 4642 Zingiberaceae

1.15 351693223 0 G 4650 Zingiber

1.15 351693223 351693223 S 94328 Zingiber officinale

0.30 91242021 0 F 4637 Musaceae

0.30 91242021 0 G 4640 Musa

0.30 91242021 0 S 4641 Musa acuminata

0.30 91242021 91242021 S1 214687 Musa acuminata subsp. malaccensis

1.14 349132281 0 O 40551 Arecales

1.14 349132281 4434695 F 4710 Arecaceae

0.73 222337761 0 F1 169697 Arecoideae

0.73 222337761 0 F2 169705 Cocoseae

0.73 222337761 0 F3 169729 Elaeidinae

0.73 222337761 0 G 51952 Elaeis

0.73 222337761 222337761 S 51953 Elaeis guineensis

0.40 122359825 0 F1 169700 Coryphoideae

0.40 122359825 0 F2 169748 Phoeniceae

0.40 122359825 0 G 4719 Phoenix

0.40 122359825 122359825 S 42345 Phoenix dactylifera

0.59 180822863 0 O 73496 Asparagales

0.59 180822863 0 F 40552 Asparagaceae

0.59 180822863 0 F1 703533 Asparagoideae

0.59 180822863 0 G 4685 Asparagus

0.59 180822863 180822863 S 4686 Asparagus officinalis

0.39 117838190 0 O 40548 Dioscoreales

0.39 117838190 0 F 4671 Dioscoreaceae

0.39 117838190 0 G 4672 Dioscorea

0.39 117838190 0 S 29710 Dioscorea cayenensis

0.39 117838190 117838190 S1 55577 Dioscorea cayenensis subsp. rotundata

1.23 374877309 0 O 41768 Ranunculales

1.23 374877309 0 F 3465 Papaveraceae

1.23 374877309 0 F1 1462614 Papaveroideae

1.23 374877309 0 G 3468 Papaver

1.23 374877309 374877309 S 3469 Papaver somniferum

1.11 337625993 0 O 232378 Proteales

1.11 337625993 3048414 F 4328 Proteaceae

0.58 176642958 0 G 54954 Telopea

0.58 176642958 176642958 S 54955 Telopea speciosissima

0.52 157934621 0 G 4329 Macadamia

0.52 157934621 157934621 S 60698 Macadamia integrifolia

0.32 96293488 0 O 261007 Nymphaeales

0.32 96293488 0 F 4410 Nymphaeaceae

0.32 96293488 0 G 4418 Nymphaea

0.32 96293488 96293488 S 210225 Nymphaea colorata

0.30 90764419 0 P3 3208 Bryophyta

0.30 90764419 0 P4 404260 Bryophytina

0.30 90764419 0 C 3214 Bryopsida

0.30 90764419 0 C1 114656 Funariidae

0.30 90764419 0 O 3215 Funariales

0.30 90764419 0 F 3216 Funariaceae

0.30 90764419 0 G 37414 Physcomitrium

0.30 90764419 90764419 S 3218 Physcomitrium patens

0.14 43026139 898 P 3041 Chlorophyta

0.08 25462308 0 P1 2692248 core chlorophytes

0.08 25462308 0 C 3166 Chlorophyceae

0.08 25462308 0 C1 2812636 CS clade

0.08 25462308 0 O 3042 Chlamydomonadales

0.08 25462308 0 F 3051 Chlamydomonadaceae

0.08 25462308 0 G 3052 Chlamydomonas

0.08 25462308 25462308 S 3055 Chlamydomonas reinhardtii

0.06 17562933 0 C 1035538 Mamiellophyceae

0.06 17562933 2887 O 13792 Mamiellales

0.04 11378911 1716 F 1525212 Bathycoccaceae

0.02 7030892 13823 G 70447 Ostreococcus

0.01 3666876 3666876 S 70448 Ostreococcus tauri

0.01 3350193 0 G1 2268852 unclassified Ostreococcus

0.01 3350193 0 S 242159 Ostreococcus sp. 'lucimarinus'

0.01 3350193 3350193 S1 436017 Ostreococcus lucimarinus CCE9901

0.01 4346303 0 G 41874 Bathycoccus

0.01 4346303 4346303 S 41875 Bathycoccus prasinos

0.02 6181135 0 F 41873 Mamiellaceae

0.02 6181135 0 G 38832 Micromonas

0.02 6181135 6181135 S 296587 Micromonas commoda

17.38 5309428480 366129 D1 33154 Opisthokonta

12.72 3887349228 104169 K 4751 Fungi

12.37 3780583685 531796 K1 451864 Dikarya

9.92 3029883014 52462 P 4890 Ascomycota

9.83 3002206755 208034 P1 716545 saccharomyceta

8.99 2746668847 75909 P2 147538 Pezizomycotina

8.77 2678184678 2530037 P3 716546 leotiomyceta

3.98 1214519388 176828 P4 715989 sordariomyceta

3.38 1033944215 1031951 C 147550 Sordariomycetes

2.10 642022526 341476 C1 222543 Hypocreomycetidae

1.41 429412552 496907 O 5125 Hypocreales

0.67 205712664 132540 F 110618 Nectriaceae

0.61 187661533 14633345 G 5506 Fusarium

0.15 46660204 6915767 G1 171627 Fusarium fujikuroi species complex

0.03 10036191 10036191 S 42677 Fusarium subglutinans

0.03 8305728 8305728 S 1567544 Fusarium tjaetaba

0.02 7416626 7416626 S 192010 Fusarium mangiferae

0.02 5540902 0 S 948311 Fusarium proliferatum

0.02 5540902 5540902 S1 1227346 Fusarium proliferatum ET1

0.02 5034817 0 S 5127 Fusarium fujikuroi

0.02 5034817 5034817 S1 1279085 Fusarium fujikuroi IMI 58289

0.01 3410173 0 S 117187 Fusarium verticillioides

0.01 3410173 3410173 S1 334819 Fusarium verticillioides 7600

0.13 39241496 5119880 G1 569360 Fusarium sambucinum species complex

0.03 9760477 9760477 S 36050 Fusarium poae

0.03 9620311 9620311 S 56646 Fusarium venenatum

0.02 7375131 0 S 5518 Fusarium graminearum

0.02 7375131 7375131 S1 229533 Fusarium graminearum PH-1

0.02 7365697 0 S 101028 Fusarium pseudograminearum

0.02 7365697 7365697 S1 1028729 Fusarium pseudograminearum CS3096

0.09 28718740 2681333 G1 232080 Fusarium solani species complex

0.04 13171050 13171050 S 169388 Fusarium solani

0.04 12866357 0 S 2747968 Fusarium vanettenii

0.04 12866357 12866357 S1 660122 Fusarium vanettenii 77-13-4

0.07 21359783 2143003 G1 450425 Fusarium incarnatum-equiseti species complex

0.03 9882830 9882830 S 2675880 Fusarium flagelliforme

0.03 9333950 9333950 S 231269 Fusarium coffeatum

0.07 20958525 5142959 G1 171631 Fusarium oxysporum species complex

0.04 10953178 2092309 S 5507 Fusarium oxysporum

0.02 4675027 0 S1 59765 Fusarium oxysporum f. sp. lycopersici

0.02 4675027 4675027 S2 426428 Fusarium oxysporum f. sp. lycopersici 4287

0.01 4185842 4185842 S1 660029 Fusarium oxysporum NRRL 32931

0.02 4862388 0 S 2502994 Fusarium odoratissimum

0.02 4862388 4862388 S1 1089451 Fusarium odoratissimum NRRL 54006

0.04 12481314 0 G1 2954887 Fusarium redolens species complex

0.04 12481314 12481314 S 48865 Fusarium redolens

0.01 3608126 3608126 S 1042133 Fusarium musae

0.06 17918591 0 G 1079112 Ilyonectria

0.06 17918591 17918591 S 1079257 Ilyonectria robusta

0.24 73743396 0 F 5129 Hypocreaceae

0.24 73743396 3360099 G 5543 Trichoderma

0.04 11450042 0 S 5544 Trichoderma harzianum

0.04 11450042 11450042 S1 983964 Trichoderma harzianum CBS 226.95

0.04 11249711 0 S 29875 Trichoderma virens

0.04 11249711 11249711 S1 413071 Trichoderma virens Gv29-8

0.03 10190828 0 S 101201 Trichoderma asperellum

0.03 10190828 10190828 S1 1042311 Trichoderma asperellum CBS 433.97

0.03 9828643 9828643 S 398673 Trichoderma gamsii

0.03 9289624 0 S 63577 Trichoderma atroviride

0.03 9289624 9289624 S1 452589 Trichoderma atroviride IMI 206040

0.03 9225170 0 S 51453 Trichoderma reesei

0.03 9225170 9225170 S1 431241 Trichoderma reesei QM6a

0.03 9149279 9149279 S 58853 Trichoderma citrinoviride

0.19 59555227 118152 F 34397 Clavicipitaceae

0.12 36778818 6098958 G 5529 Metarhizium

0.03 10062363 0 S 92637 Metarhizium acridum

0.03 10062363 10062363 S1 655827 Metarhizium acridum CQMa 102

0.03 8660907 0 S 92629 Metarhizium album

0.03 8660907 8660907 S1 1081103 Metarhizium album ARSEF 1941

0.02 6294692 0 S 568076 Metarhizium robertsii

0.02 6294692 6294692 S1 655844 Metarhizium robertsii ARSEF 23

0.02 5661898 0 S 500148 Metarhizium brunneum

0.02 5661898 5661898 S1 1276141 Metarhizium brunneum ARSEF 3297

0.04 13539362 0 G 243023 Pochonia

0.04 13539362 0 S 280754 Pochonia chlamydosporia

0.04 13539362 13539362 S1 1380566 Pochonia chlamydosporia 170

0.03 9118895 0 G 124426 Ustilaginoidea

0.03 9118895 9118895 S 1159556 Ustilaginoidea virens

0.15 45193304 52548 F 474942 Ophiocordycipitaceae

0.07 21773829 313675 G 1052105 Purpureocillium

0.04 11171609 11171609 S 33203 Purpureocillium lilacinum

0.03 10288545 10288545 S 2060973 Purpureocillium takamizusanense

0.05 14039051 0 G 42367 Hirsutella

0.05 14039051 14039051 S 111463 Hirsutella rhossiliensis

0.03 9327876 0 G 98402 Drechmeria

0.03 9327876 9327876 S 98403 Drechmeria coniospora

0.10 29048422 144355 F 474943 Cordycipitaceae

0.06 18969931 71259 G 45234 Cordyceps

0.03 9802508 0 S 114497 Cordyceps fumosorosea

0.03 9802508 9802508 S1 1081104 Cordyceps fumosorosea ARSEF 2679

0.03 9096164 0 S 73501 Cordyceps militaris

0.03 9096164 9096164 S1 983644 Cordyceps militaris CM01

0.03 9934136 0 G 5581 Beauveria

0.03 9934136 0 S 176275 Beauveria bassiana

0.03 9934136 9934136 S1 655819 Beauveria bassiana ARSEF 2860

0.03 8378209 0 O1 162454 Hypocreales incertae sedis

0.03 8378209 0 G 45244 Emericellopsis

0.03 8378209 8378209 S 2614577 Emericellopsis atlantica

0.02 7284423 0 F 103887 Bionectriaceae

0.02 7284423 0 G 241409 Geosmithia

0.02 7284423 7284423 S 1094350 Geosmithia morbida

0.65 199213336 52325 O 1028384 Glomerellales

0.54 166233396 0 F 681950 Glomerellaceae

0.54 166233396 1825142 G 5455 Colletotrichum

0.16 49398086 12619504 G1 2707338 Colletotrichum gloeosporioides species complex

0.04 12149384 12149384 S 474922 Colletotrichum gloeosporioides

0.03 8727708 8727708 S 690259 Colletotrichum siamense

0.03 8290391 8290391 S 1215731 Colletotrichum aenigma

0.02 7611099 7611099 S 690256 Colletotrichum fructicola

0.09 27527549 3858665 G1 2707335 Colletotrichum acutatum species complex

0.04 12445426 12445426 S 145971 Colletotrichum lupini

0.04 11223458 11223458 S 1209932 Colletotrichum scovillei

0.05 15814688 0 G1 2707339 Colletotrichum truncatum species complex

0.05 15814688 15814688 S 5467 Colletotrichum truncatum

0.05 15559603 0 G1 2707336 Colletotrichum boninense species complex

0.05 15559603 15559603 S 1095194 Colletotrichum karsti

0.05 14383573 0 G1 2707349 Colletotrichum spaethianum species complex

0.05 14383573 14383573 S 700344 Colletotrichum spaethianum

0.05 14380805 0 G1 2707350 Colletotrichum destructivum species complex

0.05 14380805 0 S 80884 Colletotrichum higginsianum

0.05 14380805 14380805 S1 759273 Colletotrichum higginsianum IMI 349063

0.04 13731943 13731943 S 1209926 Colletotrichum orchidophilum

0.04 13612007 0 G1 2707348 Colletotrichum graminicola species complex

0.04 13612007 0 S 31870 Colletotrichum graminicola

0.04 13612007 13612007 S1 645133 Colletotrichum graminicola M1.001

0.11 32927615 6586 F 1033978 Plectosphaerellaceae

0.07 21370315 5983818 G 1036719 Verticillium

0.02 7414539 0 S 27337 Verticillium dahliae

0.02 7414539 7414539 S1 498257 Verticillium dahliae VdLs.17

0.01 4061536 4061536 S 1051616 Verticillium nonalfalfae

0.01 3910422 0 S 1051613 Verticillium alfalfae

0.01 3910422 3910422 S1 526221 Verticillium alfalfae VaMs.102

0.04 11550714 0 G 1401161 Sodiomyces

0.04 11550714 0 S 1302862 Sodiomyces alkalinus

0.04 11550714 11550714 S1 1314773 Sodiomyces alkalinus F11

0.04 13055162 0 O 5592 Microascales

0.04 13055162 0 F 5593 Microascaceae

0.04 13055162 0 G 41687 Scedosporium

0.04 13055162 13055162 S 563466 Scedosporium apiospermum

0.69 212220839 100679 C1 222544 Sordariomycetidae

0.26 80891852 35230 O 5139 Sordariales

0.13 39306503 88521 F 35718 Chaetomiaceae

0.03 10559862 0 G 2609811 Thermothielavioides

0.03 10559862 0 S 2587410 Thermothielavioides terrestris

0.03 10559862 10559862 S1 578455 Thermothielavioides terrestris NRRL 8126

0.03 9985779 0 G 1920207 Thermothelomyces

0.03 9985779 0 S 78579 Thermothelomyces thermophilus

0.03 9985779 9985779 S1 573729 Thermothelomyces thermophilus ATCC 42464

0.03 9896300 0 G 5149 Chaetomium

0.03 9896300 0 S 38033 Chaetomium globosum

0.03 9896300 9896300 S1 306901 Chaetomium globosum CBS 148.51

0.03 8776041 0 G 2944547 Thermochaetoides

0.03 8776041 0 S 209285 Thermochaetoides thermophila

0.03 8776041 8776041 S1 759272 Thermochaetoides thermophila DSM 1495

0.10 31153169 440420 F 5148 Sordariaceae

0.06 19389257 3583144 G 5140 Neurospora

0.03 8177976 0 S 5141 Neurospora crassa

0.03 8177976 8177976 S1 367110 Neurospora crassa OR74A

0.02 7628137 0 S 40127 Neurospora tetrasperma

0.02 7628137 7628137 S1 510951 Neurospora tetrasperma FGSC 2508

0.04 11323492 0 G 5146 Sordaria

0.04 11323492 0 S 5147 Sordaria macrospora

0.04 11323492 11323492 S1 771870 Sordaria macrospora k-hell

0.03 10396950 0 F 2609812 Podosporaceae

0.03 10396950 0 G 5144 Podospora

0.03 10396950 0 S 2587412 Podospora anserina

0.03 10396950 10396950 S1 515849 Podospora anserina S mat+

0.15 46967384 13791 O 639021 Magnaporthales

0.11 35045169 0 F 2528436 Pyriculariaceae

0.11 35045169 1667132 G 48558 Pyricularia

0.04 11882040 11882040 S 1578925 Pyricularia pennisetigena

0.04 11291414 11291414 S 148305 Pyricularia grisea

0.03 10204583 0 S 318829 Pyricularia oryzae

0.03 10204583 10204583 S1 242507 Pyricularia oryzae 70-15

0.04 11908424 0 F 81093 Magnaporthaceae

0.04 11908424 0 G 29849 Gaeumannomyces

0.04 11908424 0 S 36779 Gaeumannomyces tritici

0.04 11908424 11908424 S1 644352 Gaeumannomyces tritici R3-111a-1

0.15 44606808 13770 O 5114 Diaporthales

0.10 31855646 0 F 767018 Diaporthaceae

0.10 31855646 267364 G 36922 Diaporthe

0.05 16408564 16408564 S 83186 Diaporthe citri

0.05 15179718 15179718 S 748121 Diaporthe batatas

0.04 12737392 0 F 399129 Cryphonectriaceae

0.04 12737392 0 F1 218105 Cryphonectria-Endothia species complex

0.04 12737392 0 G 5115 Cryphonectria

0.04 12737392 0 S 5116 Cryphonectria parasitica

0.04 12737392 12737392 S1 660469 Cryphonectria parasitica EP155

0.08 24779693 0 O 5151 Ophiostomatales

0.08 24779693 9026 F 5152 Ophiostomataceae

0.05 16180758 3633265 G 29907 Sporothrix

0.02 6352776 0 S 545650 Sporothrix brasiliensis

0.02 6352776 6352776 S1 1398154 Sporothrix brasiliensis 5110

0.02 6194717 0 S 29908 Sporothrix schenckii

0.02 6194717 6194717 S1 1397361 Sporothrix schenckii 1099-18

0.03 8589909 0 G 360145 Grosmannia

0.03 8589909 0 S 226899 Grosmannia clavigera

0.03 8589909 8589909 S1 655863 Grosmannia clavigera kw1407

0.05 14874423 0 O 1775898 Togniniales

0.05 14874423 0 F 1756146 Togniniaceae

0.05 14874423 0 G 65412 Phaeoacremonium

0.05 14874423 0 S 223192 Phaeoacremonium minimum

0.05 14874423 14874423 S1 1286976 Phaeoacremonium minimum UCRPA7

0.55 166673012 31670 C1 222545 Xylariomycetidae

0.50 152847635 39057 O 37989 Xylariales

0.27 81295950 113512 F 2033035 Hypoxylaceae

0.16 48170255 4044586 G 42360 Daldinia

0.03 10290005 10290005 S 326644 Daldinia caldariorum

0.03 8900784 8900784 S 114800 Daldinia vernicosa

0.03 8785085 8785085 S 326645 Daldinia childiae

0.03 8337944 8337944 S 326647 Daldinia decipiens

0.03 7811851 7811851 S 103429 Daldinia loculata

0.07 22321369 318916 G 326606 Annulohypoxylon

0.04 11099942 11099942 S 327061 Annulohypoxylon truncatum

0.04 10902511 10902511 S 1927788 Annulohypoxylon maeteangense

0.03 10690814 0 G 42308 Hypoxylon

0.03 10690814 10690814 S 63214 Hypoxylon fragiforme

0.10 30643418 10859 F 1812776 Sporocadaceae

0.05 15853629 0 G 37840 Pestalotiopsis

0.05 15853629 0 S 393283 Pestalotiopsis fici

0.05 15853629 15853629 S1 1229662 Pestalotiopsis fici W106-1

0.05 14778930 0 G 152317 Truncatella

0.05 14778930 14778930 S 152316 Truncatella angustata

0.05 14421161 0 F 1682405 Microdochiaceae

0.05 14421161 0 G 67608 Microdochium

0.05 14421161 14421161 S 1682393 Microdochium trichocladiopsis

0.04 13233626 0 F 37990 Xylariaceae

0.04 13233626 0 G 37991 Xylaria

0.04 13233626 13233626 S 326684 Xylaria bambusicola

0.04 13214423 0 F 1812770 Pseudomassariaceae

0.04 13214423 0 G 1812772 Pseudomassariella

0.04 13214423 13214423 S 1141098 Pseudomassariella vexata

0.05 13793707 0 C2 1830229 Xylariomycetidae incertae sedis

0.05 13793707 0 G 2983819 Neoarthrinium

0.05 13793707 13793707 S 1658444 Neoarthrinium moseri

0.04 11995887 0 C1 147551 Sordariomycetes incertae sedis

0.04 11995887 0 F 265081 Thyridiaceae

0.04 11995887 0 G 265082 Thyridium

0.04 11995887 11995887 S 1093900 Thyridium curvatum

0.59 180398345 18393 C 147548 Leotiomycetes

0.54 164323001 100381 O 5178 Helotiales

0.25 75801044 236006 F 28983 Sclerotiniaceae

0.21 64602313 9167581 G 33196 Botrytis

0.04 10768938 0 S 40559 Botrytis cinerea

0.04 10768938 10768938 S1 332648 Botrytis cinerea B05.10

0.03 10333998 10333998 S 87229 Botrytis porri

0.03 9862464 9862464 S 1463999 Botrytis sinoallii

0.03 9374567 9374567 S 139641 Botrytis byssoidea

0.03 9080332 9080332 S 1964551 Botrytis fragariae

0.02 6014433 6014433 S 2478750 Botrytis deweyae

0.04 10962725 0 G 5179 Sclerotinia

0.04 10962725 0 S 5180 Sclerotinia sclerotiorum

0.04 10962725 10962725 S1 665079 Sclerotinia sclerotiorum 1980 UF-70

0.06 19423387 0 F 47743 Hyaloscyphaceae

0.06 19423387 0 G 47747 Hyaloscypha

0.06 19423387 0 G1 186449 Hyaloscypha hepaticicola/Rhizoscyphus ericae species complex

0.06 19423387 0 S 2482752 Hyaloscypha bicolor

0.06 19423387 19423387 S1 1095630 Hyaloscypha bicolor E

0.05 15343307 0 F 2755564 Mollisiaceae

0.05 15343307 0 G 86026 Mollisia

0.05 15343307 15343307 S 149040 Mollisia scopiformis

0.04 12320297 0 F 5181 Helotiaceae

0.04 12320297 0 G 101851 Glarea

0.04 12320297 0 S 101852 Glarea lozoyensis

0.04 12320297 12320297 S1 1116229 Glarea lozoyensis ATCC 20868

0.04 11844654 0 F 2793945 Drepanopezizaceae

0.04 11844654 0 G 2081418 Drepanopeziza

0.04 11844654 0 S 698440 Drepanopeziza brunnea

0.04 11844654 0 S1 698441 Drepanopeziza brunnea f. sp. 'multigermtubi'

0.04 11844654 11844654 S2 1072389 Drepanopeziza brunnea f. sp. 'multigermtubi' MB_m1

0.03 10604141 0 F 2589077 Lachnaceae

0.03 10604141 0 G 47830 Lachnellula

0.03 10604141 10604141 S 1316788 Lachnellula hyalina

0.03 10597253 0 F 2656784 Pleuroascaceae

0.03 10597253 0 G 2656786 Venustampulla

0.03 10597253 10597253 S 2656787 Venustampulla echinocandica

0.03 8288537 0 F 2907085 Amorphothecaceae

0.03 8288537 0 G 5100 Amorphotheca

0.03 8288537 0 S 5101 Amorphotheca resinae

0.03 8288537 8288537 S1 857342 Amorphotheca resinae ATCC 22711

0.05 16056951 0 C1 221903 Leotiomycetes incertae sedis

0.05 16056951 0 F 34379 Pseudeurotiaceae

0.05 16056951 871968 G 78156 Pseudogymnoascus

0.03 8315554 8315554 S 342668 Pseudogymnoascus verrucosus

0.02 6869429 6869429 S 655981 Pseudogymnoascus destructans

2.97 907529069 159804 C 147545 Eurotiomycetes

2.27 692427128 139980 C1 451871 Eurotiomycetidae

1.92 585311294 246616 O 5042 Eurotiales

1.66 507058289 362730 F 1131492 Aspergillaceae

1.40 428698464 56075113 G 5052 Aspergillus

0.13 40726367 7944617 G1 2720871 Aspergillus subgen. Circumdati

0.03 10608675 0 S 306088 Aspergillus steynii

0.03 10608675 10608675 S1 1392250 Aspergillus steynii IBT 23096

0.03 9054657 0 S 33178 Aspergillus terreus

0.03 9054657 9054657 S1 341663 Aspergillus terreus NIH2624

0.02 6206454 0 S 5053 Aspergillus aculeatus

0.02 6206454 6206454 S1 690307 Aspergillus aculeatus ATCC 16872

0.01 3740187 0 S 5061 Aspergillus niger

0.01 3740187 3740187 S1 425011 Aspergillus niger CBS 513.88

0.01 1586835 0 S 5059 Aspergillus flavus

0.01 1586835 1586835 S1 332952 Aspergillus flavus NRRL3357

0.01 1584942 0 S 5062 Aspergillus oryzae

0.01 1584942 1584942 S1 510516 Aspergillus oryzae RIB40

0.11 35085586 463961 G1 2720870 Aspergillus subgen. Nidulantes

0.03 9231057 0 S 162425 Aspergillus nidulans

0.03 9231057 9231057 S1 227321 Aspergillus nidulans FGSC A4

0.03 9227652 0 S 75750 Aspergillus sydowii

0.03 9227652 9227652 S1 1036612 Aspergillus sydowii CBS 593.65

0.03 8936609 0 S 46472 Aspergillus versicolor

0.03 8936609 8936609 S1 1036611 Aspergillus versicolor CBS 583.65

0.02 7226307 0 S 138278 Aspergillus ochraceoroseus

0.02 7226307 7226307 S1 1392256 Aspergillus ochraceoroseus IBT 24754

0.07 21104063 531261 G1 2720872 Aspergillus subgen. Fumigati

0.03 8127831 0 S 5057 Aspergillus clavatus

0.03 8127831 8127831 S1 344612 Aspergillus clavatus NRRL 1

0.02 6703555 0 S 746128 Aspergillus fumigatus

0.02 6703555 6703555 S1 330879 Aspergillus fumigatus Af293

0.02 5741416 0 S 36630 Aspergillus fischeri

0.02 5741416 5741416 S1 331117 Aspergillus fischeri NRRL 181

0.04 12095338 12095338 S 209559 Aspergillus alliaceus

0.04 11475821 11475821 S 1220188 Aspergillus tanneri

0.04 11151315 11151315 S 138277 Aspergillus melleus

0.04 10739795 10739795 S 1810919 Aspergillus mulundensis

0.03 10288439 0 S 319627 Aspergillus sclerotioniger

0.03 10288439 10288439 S1 1450535 Aspergillus sclerotioniger CBS 115572

0.03 9675402 0 S 319626 Aspergillus homomorphus

0.03 9675402 9675402 S1 1450537 Aspergillus homomorphus CBS 101889

0.03 9563731 0 G1 2720873 Aspergillus subgen. Cremei

0.03 9563731 0 S 5066 Aspergillus wentii

0.03 9563731 9563731 S1 1073089 Aspergillus wentii DTO 134E9

0.03 9288692 0 S 301854 Aspergillus ibericus

0.03 9288692 9288692 S1 1448316 Aspergillus ibericus CBS 121593

0.03 9032202 9032202 S 1220207 Aspergillus puulaauensis

0.03 9000120 9000120 S 109264 Aspergillus bombycis

0.03 8981917 0 S 979771 Aspergillus saccharolyticus

0.03 8981917 8981917 S1 1450539 Aspergillus saccharolyticus JOP 1030-1

0.03 8893569 0 S 51019 Aspergillus heteromorphus

0.03 8893569 8893569 S1 1448321 Aspergillus heteromorphus CBS 117.55

0.03 8419111 8419111 S 61420 Aspergillus caelatus

0.03 8109423 8109423 S 41047 Aspergillus thermomutatus

0.03 8090120 8090120 S 132259 Aspergillus pseudotamarii

0.02 7625959 7625959 S 182096 Aspergillus chevalieri

0.02 7091911 7091911 S 1517512 Aspergillus pseudoviridinutans

0.02 7043377 0 G1 2720874 Aspergillus subgen. Aspergillus

0.02 7043377 0 S 41413 Aspergillus glaucus

0.02 7043377 7043377 S1 1160497 Aspergillus glaucus CBS 516.65

0.02 6890378 0 S 446911 Aspergillus uvarum

0.02 6890378 6890378 S1 1448315 Aspergillus uvarum CBS 121591

0.02 6847409 6847409 S 91492 Aspergillus udagawae

0.02 6739828 0 S 396024 Aspergillus ruber

0.02 6739828 6739828 S1 1388766 Aspergillus ruber CBS 135680

0.02 6724106 0 S 34381 Aspergillus japonicus

0.02 6724106 6724106 S1 1448312 Aspergillus japonicus CBS 114.51

0.02 6495384 6495384 S 75553 Aspergillus viridinutans

0.02 6493102 0 S 41068 Aspergillus campestris

0.02 6493102 6493102 S1 1392248 Aspergillus campestris IBT 28561

0.02 6465536 6465536 S 41067 Aspergillus candidus

0.02 6364291 0 S 340412 Aspergillus novofumigatus

0.02 6364291 6364291 S1 1392255 Aspergillus novofumigatus IBT 16806

0.02 6227396 6227396 S 293939 Aspergillus lentulus

0.02 5494944 0 S 288669 Aspergillus vadensis

0.02 5494944 5494944 S1 1448311 Aspergillus vadensis CBS 113365

0.02 4942315 4942315 S 1506151 Aspergillus pseudonomiae

0.02 4626736 0 S 1340029 Aspergillus eucalypticola

0.02 4626736 4626736 S1 1448314 Aspergillus eucalypticola CBS 122712

0.02 4601662 0 S 41061 Aspergillus nomiae

0.02 4601662 4601662 S1 1509407 Aspergillus nomiae NRRL 13137

0.01 4534718 4534718 S 1341132 Aspergillus welwitschiae

0.01 4019712 0 S 487661 Aspergillus aculeatinus

0.01 4019712 4019712 S1 1448322 Aspergillus aculeatinus CBS 121060

0.01 3447500 3447500 S 5068 Aspergillus tubingensis

0.01 3328446 0 S 1196635 Aspergillus neoniger

0.01 3328446 3328446 S1 1448310 Aspergillus neoniger CBS 115656

0.01 3287932 3287932 S 1069201 Aspergillus luchuensis

0.01 3253972 0 S 319631 Aspergillus costaricensis

0.01 3253972 3253972 S1 1448317 Aspergillus costaricaensis CBS 115574

0.01 3034369 0 S 319630 Aspergillus piperis

0.01 3034369 3034369 S1 1448313 Aspergillus piperis CBS 112811

0.01 2743368 0 S 1191711 Aspergillus brunneoviolaceus

0.01 2743368 2743368 S1 1450534 Aspergillus brunneoviolaceus CBS 621.78

0.01 2577989 0 S 1191702 Aspergillus fijiensis

0.01 2577989 2577989 S1 1448319 Aspergillus fijiensis CBS 313.89

0.23 70402510 2920524 G 5073 Penicillium

0.03 10185849 10185849 S 1835702 Penicillium arizonense

0.03 9305765 9305765 S 69781 Penicillium oxalicum

0.03 8635887 0 G1 254878 Penicillium chrysogenum species complex

0.03 8635887 0 S 1108849 Penicillium rubens

0.03 8635887 8635887 S1 500485 Penicillium rubens Wisconsin 54-1255

0.03 8623478 8623478 S 60172 Penicillium solitum

0.03 8424666 8424666 S 27334 Penicillium expansum

0.03 8149236 8149236 S 5078 Penicillium griseofulvum

0.02 7351262 7351262 S 5082 Penicillium roqueforti

0.02 6805843 0 S 36651 Penicillium digitatum

0.02 6805843 6805843 S1 1170230 Penicillium digitatum Pd1

0.02 7594585 0 G 70110 Penicilliopsis

0.02 7594585 0 S 41063 Penicilliopsis zonata

0.02 7594585 7594585 S1 1073090 Penicilliopsis zonata CBS 506.65

0.23 69125122 5745 F 28568 Trichocomaceae

0.20 60559703 100630 G 5094 Talaromyces

0.10 29298465 268383 G1 2752537 Talaromyces sect. Talaromyces

0.03 10394433 10394433 S 1196081 Talaromyces amestolkiae

0.03 10269458 0 S 28564 Talaromyces stipitatus

0.03 10269458 10269458 S1 441959 Talaromyces stipitatus ATCC 10500

0.03 8366191 0 S 37727 Talaromyces marneffei

0.03 8366191 8366191 S1 441960 Talaromyces marneffei ATCC 18224

0.04 11606556 0 G1 2752543 Talaromyces sect. Bacilispori

0.04 11606556 11606556 S 1131652 Talaromyces proteolyticus

0.04 10696671 0 G1 2752542 Talaromyces sect. Islandici

0.04 10696671 10696671 S 121627 Talaromyces rugulosus

0.03 8857381 0 G1 2752540 Talaromyces sect. Trachyspermi

0.03 8857381 8857381 S 1441469 Talaromyces atroroseus

0.03 8559674 0 G 1132856 Rasamsonia

0.03 8559674 0 S 68825 Rasamsonia emersonii

0.03 8559674 8559674 S1 1408163 Rasamsonia emersonii CBS 393.64

0.03 8881267 0 F 1131624 Thermoascaceae

0.03 8881267 0 G 33202 Paecilomyces

0.03 8881267 8881267 S 264951 Paecilomyces variotii

0.35 106975854 113540 O 33183 Onygenales

0.12 36124338 434884 F 34384 Arthrodermataceae

0.05 14755621 3992411 G 5550 Trichophyton

0.02 4744150 0 S 5551 Trichophyton rubrum

0.02 4744150 4744150 S1 559305 Trichophyton rubrum CBS 118892

0.01 3027617 0 S 63417 Trichophyton verrucosum

0.01 3027617 3027617 S1 663202 Trichophyton verrucosum HKI 0517

0.01 2991443 0 S 63400 Trichophyton benhamiae

0.01 2991443 2991443 S1 663331 Trichophyton benhamiae CBS 112371

0.02 7134483 0 G 63399 Arthroderma

0.02 7134483 7134483 S 74035 Arthroderma uncinatum

0.02 6968589 0 G 34392 Microsporum

0.02 6968589 0 S 63405 Microsporum canis

0.02 6968589 6968589 S1 554155 Microsporum canis CBS 113480

0.02 6830761 0 G 1915381 Nannizzia

0.02 6830761 0 S 63402 Nannizzia gypsea

0.02 6830761 6830761 S1 535722 Nannizzia gypsea CBS 118893

0.10 30440911 150963 F 299071 Ajellomycetaceae

0.05 16282052 8384959 G 229219 Blastomyces

0.02 4620528 0 S 1681229 Blastomyces gilchristii

0.02 4620528 4620528 S1 559298 Blastomyces gilchristii SLH14081

0.01 3276565 0 S 5039 Blastomyces dermatitidis

0.01 3276565 3276565 S1 559297 Blastomyces dermatitidis ER-3

0.05 14007896 2940751 G 5036 Histoplasma

0.02 5737661 0 S 5037 Histoplasma capsulatum

0.02 5737661 5737661 S1 447093 Histoplasma capsulatum G186AR

0.02 5329484 0 G1 2617678 unclassified Histoplasma

0.02 5329484 5329484 S 2059318 Histoplasma mississippiense (nom. inval.)

0.08 25092548 22318 F 33184 Onygenaceae

0.04 11491231 4977210 G 5500 Coccidioides

0.01 3490066 0 S 5501 Coccidioides immitis

0.01 3490066 3490066 S1 246410 Coccidioides immitis RS

0.01 3023955 0 S 199306 Coccidioides posadasii

0.01 3023955 3023955 S1 222929 Coccidioides posadasii C735 delta SOWgp

0.02 6965231 0 G 33187 Uncinocarpus

0.02 6965231 0 S 33188 Uncinocarpus reesii

0.02 6965231 6965231 S1 336963 Uncinocarpus reesii 1704

0.02 6613768 0 G 1387562 Ophidiomyces

0.02 6613768 6613768 S 1387563 Ophidiomyces ophidiicola

0.05 15204517 0 O1 1593277 Onygenales incertae sedis

0.05 15204517 2267431 G 38946 Paracoccidioides

0.02 6770002 0 S 1048829 Paracoccidioides lutzii

0.02 6770002 6770002 S1 502779 Paracoccidioides lutzii Pb01

0.02 6167084 0 S 121759 Paracoccidioides brasiliensis

0.02 6167084 6167084 S1 502780 Paracoccidioides brasiliensis Pb18

0.70 214942137 5883 C1 451870 Chaetothyriomycetidae

0.67 204117866 14538 O 34395 Chaetothyriales

0.64 194956550 1174461 F 43219 Herpotrichiellaceae

0.20 62338742 211215 G 5583 Exophiala

0.04 12625786 0 S 1033840 Exophiala aquamarina

0.04 12625786 12625786 S1 1182545 Exophiala aquamarina CBS 119918

0.04 11659953 11659953 S 215243 Exophiala oligosperma

0.03 10227281 10227281 S 91928 Exophiala spinifera

0.03 9950387 9950387 S 348802 Exophiala xenobiotica

0.03 9367668 9367668 S 212818 Exophiala mesophila

0.03 8296452 0 S 5970 Exophiala dermatitidis

0.03 8296452 8296452 S1 858893 Exophiala dermatitidis NIH/UT8656

0.17 51474980 2816056 G 82105 Cladophialophora

0.04 12867178 12867178 S 569365 Cladophialophora immunda

0.03 9707277 0 S 1182553 Cladophialophora psammophila

0.03 9707277 9707277 S1 1182543 Cladophialophora psammophila CBS 110553

0.03 9076234 0 S 89940 Cladophialophora bantiana

0.03 9076234 9076234 S1 1442370 Cladophialophora bantiana CBS 173.52

0.03 8663118 0 S 86049 Cladophialophora carrionii

0.03 8663118 8663118 S1 1279043 Cladophialophora carrionii CBS 160.54

0.03 8345117 0 S 470704 Cladophialophora yegresii

0.03 8345117 8345117 S1 1182544 Cladophialophora yegresii CBS 114405

0.14 42800983 7298528 G 40354 Fonsecaea

0.03 10444359 10444359 S 1367422 Fonsecaea erecta

0.03 10001286 0 S 979981 Fonsecaea multimorphosa

0.03 10001286 10001286 S1 1442371 Fonsecaea multimorphosa CBS 102226

0.02 6100127 6100127 S 856822 Fonsecaea nubica

0.01 4571883 4571883 S 254056 Fonsecaea monophora

0.01 4384800 0 S 40355 Fonsecaea pedrosoi

0.01 4384800 4384800 S1 1442368 Fonsecaea pedrosoi CBS 271.37

0.06 17200007 29907 G 43220 Capronia

0.03 8989951 0 S 43228 Capronia epimyces

0.03 8989951 8989951 S1 1182542 Capronia epimyces CBS 606.96

0.03 8180149 0 S 43229 Capronia coronata

0.03 8180149 8180149 S1 1182541 Capronia coronata CBS 617.96

0.03 10239758 0 G 5587 Rhinocladiella

0.03 10239758 0 S 86056 Rhinocladiella mackenziei

0.03 10239758 10239758 S1 1442369 Rhinocladiella mackenziei CBS 650.93

0.03 9727619 0 G 5600 Phialophora

0.03 9727619 9727619 S 1664694 Phialophora attinorum

0.03 9146778 0 F 1341112 Cyphellophoraceae

0.03 9146778 0 G 226991 Cyphellophora

0.03 9146778 0 S 293227 Cyphellophora europaea

0.03 9146778 9146778 S1 1220924 Cyphellophora europaea CBS 101466

0.04 10818388 0 O 146291 Verrucariales

0.04 10818388 0 F 146292 Verrucariaceae

0.04 10818388 0 G 364710 Endocarpon

0.04 10818388 0 S 364733 Endocarpon pusillum

0.04 10818388 10818388 S1 1263415 Endocarpon pusillum Z07020

1.70 519584918 0 P4 715962 dothideomyceta

1.70 519584918 217931 C 147541 Dothideomycetes

1.00 305467892 31428 C1 451868 Pleosporomycetidae

0.92 281981973 284648 O 92860 Pleosporales

0.68 209135023 353322 O1 715340 Pleosporineae

0.45 136744995 525414 F 28556 Pleosporaceae

0.26 80686209 3090499 G 5598 Alternaria

0.13 38677957 11551433 G1 2499258 Alternaria sect. Infectoriae

0.02 5729630 5729630 S 1187951 Alternaria ventricosa

0.01 3694468 3694468 S 283354 Alternaria metachromatica

0.01 3636253 3636253 S 430562 Alternaria novae-zelandiae

0.01 3600621 3600621 S 1187925 Alternaria hordeiaustralica

0.01 3521079 3521079 S 566460 Alternaria viburni

0.01 3474424 3474424 S 297637 Alternaria triticimaculans

0.01 3470049 3470049 S 181014 Alternaria ethzedia

0.07 20293470 6662063 G1 2499237 Alternaria sect. Alternaria

0.02 5406007 5406007 S 1187904 Alternaria burnsii

0.01 4486364 4486364 S 156630 Alternaria arborescens

0.01 3739036 0 G2 187734 Alternaria alternata complex

0.01 3739036 3739036 S 5599 Alternaria alternata

0.04 10772320 0 G1 2499266 Alternaria sect. Ulocladioides

0.04 10772320 10772320 S 119953 Alternaria atra

0.03 7851963 0 G1 2499270 Alternaria sect. Pseudoalternaria

0.03 7851963 7851963 S 1187941 Alternaria rosae

0.12 35164020 9142191 G 33194 Bipolaris

0.02 7399885 0 S 5016 Bipolaris maydis

0.02 7399885 7399885 S1 665024 Bipolaris maydis ATCC 48331

0.02 7196324 0 S 45130 Bipolaris sorokiniana

0.02 7196324 7196324 S1 665912 Bipolaris sorokiniana ND90Pr

0.02 7130156 0 S 101162 Bipolaris oryzae

0.02 7130156 7130156 S1 930090 Bipolaris oryzae ATCC 44560

0.01 2334336 0 S 40125 Bipolaris victoriae

0.01 2334336 2334336 S1 930091 Bipolaris victoriae FI3

0.01 1961128 0 S 5017 Bipolaris zeicola

0.01 1961128 1961128 S1 930089 Bipolaris zeicola 26-R-13

0.03 10205121 0 G 5027 Pyrenophora

0.03 10205121 0 S 45151 Pyrenophora tritici-repentis

0.03 10205121 10205121 S1 426418 Pyrenophora tritici-repentis Pt-1C-BFP

0.03 10164231 0 G 91493 Exserohilum

0.03 10164231 0 S 93612 Exserohilum turcicum

0.03 10164231 10164231 S1 671987 Exserohilum turcica Et28A

0.14 41321915 334671 F 683158 Didymellaceae

0.04 10717873 0 G 5453 Ascochyta

0.04 10717873 10717873 S 5454 Ascochyta rabiei

0.03 10612398 0 G 749461 Boeremia

0.03 10612398 10612398 S 749465 Boeremia exigua

0.03 10066253 0 G 301206 Macroventuria

0.03 10066253 10066253 S 301207 Macroventuria anomochaeta

0.03 9590720 0 G 55170 Didymella

0.03 9590720 0 S 100019 Didymella exigua

0.03 9590720 9590720 S1 1150837 Didymella exigua CBS 183.55

0.04 11249747 0 F 5020 Phaeosphaeriaceae

0.04 11249747 0 G 1351751 Parastagonospora

0.04 11249747 0 S 13684 Parastagonospora nodorum

0.04 11249747 11249747 S1 321614 Parastagonospora nodorum SN15

0.03 9921152 0 F 221670 Cucurbitariaceae

0.03 9921152 0 G 45141 Cucurbitaria

0.03 9921152 0 S 45142 Cucurbitaria berberidis

0.03 9921152 9921152 S1 1168544 Cucurbitaria berberidis CBS 394.84

0.03 9543892 0 F 34374 Leptosphaeriaceae

0.03 9543892 0 G 5021 Leptosphaeria

0.03 9543892 0 G1 220671 Leptosphaeria maculans species complex

0.03 9543892 0 S 5022 Leptosphaeria maculans

0.03 9543892 0 S1 225342 Leptosphaeria maculans 'brassicae' group

0.03 9543892 9543892 S2 985895 Leptosphaeria maculans JN3

0.08 25951714 5561 O1 1255046 Massarineae

0.05 13880960 0 F 1208339 Trematosphaeriaceae

0.05 13880960 0 G 100048 Trematosphaeria

0.05 13880960 13880960 S 390896 Trematosphaeria pertusa

0.04 12065193 0 F 221678 Didymosphaeriaceae

0.04 12065193 0 G 125369 Paraphaeosphaeria

0.04 12065193 12065193 S 1460663 Paraphaeosphaeria sporulosa

0.05 16098940 0 F 717954 Lindgomycetaceae

0.05 16098940 0 G 741162 Lindgomyces

0.05 16098940 16098940 S 673940 Lindgomyces ingoldianus

0.04 12137547 0 O1 147498 Pleosporales incertae sedis

0.04 12137547 0 G 1450170 Aaosphaeria

0.04 12137547 0 S 1450171 Aaosphaeria arxii

0.04 12137547 12137547 S1 1450172 Aaosphaeria arxii CBS 175.79

0.03 9929558 0 F 548648 Dothidotthiaceae

0.03 9929558 0 G 548651 Dothidotthia

0.03 9929558 0 S 548649 Dothidotthia symphoricarpi

0.03 9929558 9929558 S1 1392245 Dothidotthia symphoricarpi CBS 119687

0.03 8444543 0 F 55176 Sporormiaceae

0.03 8444543 0 G 45153 Westerdykella

0.03 8444543 8444543 S 318751 Westerdykella ornata

0.05 14105735 0 O 603422 Mytilinidiales

0.05 14105735 0 F 281242 Mytilinidiaceae

0.05 14105735 0 G 574786 Mytilinidion

0.05 14105735 14105735 S 574789 Mytilinidion resinicola

0.03 9348756 0 O 1111111 Venturiales

0.03 9348756 0 F 1111112 Sympoventuriaceae

0.03 9348756 0 G 1494215 Verruconis

0.03 9348756 9348756 S 253628 Verruconis gallopava

0.50 153636661 30627 C1 451867 Dothideomycetidae

0.36 109588437 23238 O 2726947 Mycosphaerellales

0.29 87945835 70479 F 93133 Mycosphaerellaceae

0.06 19503478 1838885 G 29002 Cercospora

0.03 8871936 8871936 S 122368 Cercospora beticola

0.03 8792657 8792657 S 84275 Cercospora kikuchii

0.05 14718893 0 G 131324 Pseudocercospora

0.05 14718893 0 S 1873960 Pseudocercospora fijiensis

0.05 14718893 14718893 S1 383855 Pseudocercospora fijiensis CIRAD86

0.04 12630132 0 G 2897311 Fulvia

0.04 12630132 12630132 S 5499 Fulvia fulva

0.04 12090667 0 G 395590 Zasmidium

0.04 12090667 0 S 395010 Zasmidium cellare

0.04 12090667 12090667 S1 1080233 Zasmidium cellare ATCC 36951

0.04 11265959 0 G 1047167 Zymoseptoria

0.04 11265959 0 S 1047171 Zymoseptoria tritici

0.04 11265959 11265959 S1 336722 Zymoseptoria tritici IPO323

0.03 9416962 0 G 112497 Ramularia

0.03 9416962 9416962 S 112498 Ramularia collo-cygni

0.03 8249265 0 G 237179 Sphaerulina

0.03 8249265 0 S 85929 Sphaerulina musiva

0.03 8249265 8249265 S1 692275 Sphaerulina musiva SO2202

0.04 13389179 1360 F 668547 Teratosphaeriaceae

0.02 6950547 0 G 483074 Baudoinia

0.02 6950547 0 S 1709381 Baudoinia panamericana

0.02 6950547 6950547 S1 717646 Baudoinia panamericana UAMH 10762

0.02 6437272 0 G 2072583 Neohortaea

0.02 6437272 6437272 S 245834 Neohortaea acidophila

0.03 8230185 0 F 744530 Dissoconiaceae

0.03 8230185 0 G 112488 Dissoconium

0.03 8230185 0 S 112489 Dissoconium aciculare

0.03 8230185 8230185 S1 1314786 Dissoconium aciculare CBS 342.82

0.11 33519610 0 O 5014 Dothideales

0.11 33519610 0 F 1570301 Saccotheciaceae

0.11 33519610 338488 G 5579 Aureobasidium

0.03 9160818 0 S 5580 Aureobasidium pullulans

0.03 9160818 9160818 S1 1043002 Aureobasidium pullulans EXF-150

0.03 8117297 0 S 46634 Aureobasidium melanogenum

0.03 8117297 8117297 S1 1043003 Aureobasidium melanogenum CBS 110374

0.03 8014690 0 S 1042127 Aureobasidium subglaciale

0.03 8014690 8014690 S1 1043005 Aureobasidium subglaciale EXF-2481

0.03 7888317 0 S 559561 Aureobasidium namibiae

0.03 7888317 7888317 S1 1043004 Aureobasidium namibiae CBS 147.97

0.03 10497987 0 O 2726946 Cladosporiales

0.03 10497987 0 F 452563 Cladosporiaceae

0.03 10497987 0 G 5498 Cladosporium

0.03 10497987 10497987 S 29917 Cladosporium cladosporioides

0.20 60262434 6598 C1 159987 Dothideomycetes incertae sedis

0.11 33445218 15095 O 451869 Botryosphaeriales

0.08 23784864 264487 F 45131 Botryosphaeriaceae

0.04 13336894 0 G 66739 Lasiodiplodia

0.04 13336894 13336894 S 45133 Lasiodiplodia theobromae

0.03 10183483 0 G 66735 Diplodia

0.03 10183483 10183483 S 236234 Diplodia corticola

0.03 9645259 0 F 1450293 Aplosporellaceae

0.03 9645259 0 G 462253 Aplosporella

0.03 9645259 0 S 462254 Aplosporella prunicola

0.03 9645259 9645259 S1 1176127 Aplosporella prunicola CBS 121167

0.03 9753034 0 O 716585 Acrospermales

0.03 9753034 0 F 152637 Acrospermaceae

0.03 9753034 0 G 470095 Pseudovirgaria

0.03 9753034 9753034 S 470096 Pseudovirgaria hyperparasitica

0.03 8845511 0 G 2810619 Coniosporium

0.03 8845511 0 S 61459 Coniosporium apollinis

0.03 8845511 8845511 S1 1168221 Coniosporium apollinis CBS 100218

0.03 8212073 0 O 2714147 Eremomycetales

0.03 8212073 0 F 241722 Eremomycetaceae

0.03 8212073 0 G 258075 Eremomyces

0.03 8212073 0 S 1341166 Eremomyces bilateralis

0.03 8212073 8212073 S1 1392243 Eremomyces bilateralis CBS 781.70

0.09 26520044 0 C 147547 Lecanoromycetes

0.09 26520044 0 C1 1520881 OSLEUM clade

0.09 26520044 0 C2 388435 Lecanoromycetidae

0.09 26520044 0 O 5197 Lecanorales

0.09 26520044 708 O1 157822 Lecanorineae

0.06 17143580 0 F 78060 Parmeliaceae

0.06 17143580 6018098 G 112415 Letharia

0.02 5864936 5864936 S 112416 Letharia columbiana

0.02 5260546 5260546 S 560253 Letharia lupina

0.03 9375756 0 F 56478 Ramalinaceae

0.03 9375756 0 G 93111 Bacidia

0.03 9375756 9375756 S 2732470 Bacidia gigantensis

0.02 7501222 0 C 1217819 Xylonomycetes

0.02 7501222 0 O 1217820 Xylonales

0.02 7501222 0 F 1217822 Xylonaceae

0.02 7501222 0 G 1217823 Xylona

0.02 7501222 0 S 1217826 Xylona heveae

0.02 7501222 7501222 S1 1328760 Xylona heveae TC161

0.18 56325374 0 C 147549 Pezizomycetes

0.18 56325374 2529 O 5185 Pezizales

0.10 30264783 0 F 40289 Tuberaceae

0.10 30264783 0 G 36048 Tuber

0.10 30264783 0 S 39416 Tuber melanosporum

0.10 30264783 30264783 S1 656061 Tuber melanosporum Mel28

0.09 26058062 0 F 5192 Morchellaceae

0.09 26058062 0 G 5193 Morchella

0.09 26058062 2298398 G1 1051054 Morchella sect. Distantes

0.04 11983877 11983877 S 1174677 Morchella sextelata

0.04 11775787 11775787 S 1174673 Morchella importuna

0.04 12082886 0 C 189478 Orbiliomycetes

0.04 12082886 0 O 189479 Orbiliales

0.04 12082886 0 F 47021 Orbiliaceae

0.04 12082886 0 G 47022 Orbilia

0.04 12082886 0 S 2813651 Orbilia oligospora

0.04 12082886 12082886 S1 756982 Orbilia oligospora ATCC 24927

0.84 255329874 0 P2 147537 Saccharomycotina

0.84 255329874 0 C 4891 Saccharomycetes

0.84 255329874 334737 O 4892 Saccharomycetales

0.31 94068377 40716 O1 2916678 CUG-Ser1 clade

0.21 65592997 115279 F 766764 Debaryomycetaceae

0.09 26781833 10365 F1 1535325 Candida/Lodderomyces clade

0.07 22873311 324574 G 5475 Candida

0.01 3974566 0 S 5482 Candida tropicalis

0.01 3974566 3974566 S1 294747 Candida tropicalis MYA-3404

0.01 3883784 3883784 S 5480 Candida parapsilosis

0.01 3839360 0 S 273371 Candida orthopsilosis

0.01 3839360 3839360 S1 1136231 Candida orthopsilosis Co 90-125

0.01 3701754 0 S 5476 Candida albicans

0.01 3701754 3701754 S1 237561 Candida albicans SC5314

0.01 3624420 0 S 42374 Candida dubliniensis

0.01 3624420 3624420 S1 573826 Candida dubliniensis CD36

0.01 3524853 3524853 S 497107 Candida oxycetoniae

0.01 3898157 0 G 36913 Lodderomyces

0.01 3898157 0 S 36914 Lodderomyces elongisporus

0.01 3898157 3898157 S1 379508 Lodderomyces elongisporus NRRL YB-4239

0.03 8486891 672 G 766733 Scheffersomyces

0.02 4694781 0 S 4924 Scheffersomyces stipitis

0.02 4694781 4694781 S1 322104 Scheffersomyces stipitis CBS 6054

0.01 3791438 3791438 S 45513 Scheffersomyces spartinae

0.03 8446038 6136 G 412764 Spathaspora

0.01 4516963 4516963 S 561895 [Candida] subhashii

0.01 3922939 0 S 340170 Spathaspora passalidarum

0.01 3922939 3922939 S1 619300 Spathaspora passalidarum NRRL Y-27907

0.02 7234987 136550 G 4958 Debaryomyces

0.01 3597788 0 S 4959 Debaryomyces hansenii

0.01 3597788 3597788 S1 284592 Debaryomyces hansenii CBS767

0.01 3500649 3500649 S 58627 Debaryomyces fabryi

0.01 4171791 0 G 1539666 Suhomyces

0.01 4171791 0 S 46583 Suhomyces tanzawaensis

0.01 4171791 4171791 S1 984487 Suhomyces tanzawaensis NRRL Y-17324

0.01 3618409 0 G 507510 Hyphopichia

0.01 3618409 0 S 717740 Hyphopichia burtonii

0.01 3618409 3618409 S1 984485 Hyphopichia burtonii NRRL Y-1933

0.01 3391690 0 G 766765 Yamadazyma

0.01 3391690 0 S 2315449 Yamadazyma tenuis

0.01 3391690 3391690 S1 590646 Yamadazyma tenuis ATCC 10573

0.01 3346079 0 G 766728 Meyerozyma

0.01 3346079 0 S 4929 Meyerozyma guilliermondii

0.01 3346079 3346079 S1 294746 Meyerozyma guilliermondii ATCC 6260

0.08 23655031 13252 F 27319 Metschnikowiaceae

0.05 14984593 0 F1 2937349 Metschnikowiaceae incertae sedis

0.05 14984593 954151 G 2964429 Candida/Metschnikowiaceae

0.01 4067491 4067491 S 45357 [Candida] haemuloni

0.01 3857399 3857399 S 498019 [Candida] auris

0.01 3063072 3063072 S 418784 [Candida] pseudohaemulonii

0.01 3042480 3042480 S 1231522 [Candida] duobushaemulonis

0.02 4758577 0 G 27320 Metschnikowia

0.02 4758577 0 S 27322 Metschnikowia bicuspidata

0.02 4758577 0 S1 280587 Metschnikowia bicuspidata var. bicuspidata

0.02 4758577 4758577 S2 869754 Metschnikowia bicuspidata var. bicuspidata NRRL YB-4993

0.01 3898609 0 G 36910 Clavispora

0.01 3898609 0 S 36911 Clavispora lusitaniae

0.01 3898609 3898609 S1 306902 Clavispora lusitaniae ATCC 42720

0.02 4779633 0 O2 2925792 CUG-Ser1 clade incertae sedis

0.02 4779633 0 G 766748 Babjeviella

0.02 4779633 0 S 45609 Babjeviella inositovora

0.02 4779633 4779633 S1 984486 Babjeviella inositovora NRRL Y-12698

0.25 76985296 158044 F 4893 Saccharomycetaceae

0.03 10376351 2525 G 71245 Kazachstania

0.01 3666781 3666781 S 61262 Kazachstania barnettii

0.01 3360331 0 S 432096 Kazachstania africana

0.01 3360331 3360331 S1 1071382 Kazachstania africana CBS 2517

0.01 3346714 0 S 588726 Kazachstania naganishii

0.01 3346714 3346714 S1 1071383 Kazachstania naganishii CBS 8797

0.03 10136479 298674 G 4930 Saccharomyces

0.01 3411226 3411226 S 1080349 Saccharomyces eubayanus

0.01 3222549 3222549 S 27291 Saccharomyces paradoxus

0.01 3204030 0 S 4932 Saccharomyces cerevisiae

0.01 3204030 3204030 S1 559292 Saccharomyces cerevisiae S288C

0.03 8516201 2817 G 33170 Eremothecium

0.01 3007350 0 S 45285 Eremothecium cymbalariae

0.01 3007350 3007350 S1 931890 Eremothecium cymbalariae DBVPG#7215

0.01 2771026 0 S 33169 Eremothecium gossypii

0.01 2771026 2771026 S1 284811 Eremothecium gossypii ATCC 10895

0.01 2735008 2735008 S 45286 Eremothecium sinecaudum

0.02 7420095 1650 G 113604 Tetrapisispora

0.01 3847980 0 S 1071379 Tetrapisispora blattae

0.01 3847980 3847980 S1 1071380 Tetrapisispora blattae CBS 6284

0.01 3570465 0 S 113608 Tetrapisispora phaffii

0.01 3570465 3570465 S1 1071381 Tetrapisispora phaffii CBS 4417

0.02 7347548 5907 G 278028 Naumovozyma

0.01 3925649 0 S 27289 Naumovozyma dairenensis

0.01 3925649 3925649 S1 1071378 Naumovozyma dairenensis CBS 421

0.01 3415992 0 S 27288 Naumovozyma castellii

0.01 3415992 3415992 S1 1064592 Naumovozyma castellii CBS 4309

0.02 6777931 1891 G 300275 Lachancea

0.01 3509926 3509926 S 1245769 Lachancea lanzarotensis

0.01 3266114 0 S 381046 Lachancea thermotolerans

0.01 3266114 3266114 S1 559295 Lachancea thermotolerans CBS 6340

0.02 6534264 10009 G 4910 Kluyveromyces

0.01 3289643 3289643 S 28985 Kluyveromyces lactis

0.01 3234612 0 S 4911 Kluyveromyces marxianus

0.01 3234612 3234612 S1 1003335 Kluyveromyces marxianus DMKU3-1042

0.02 5822558 2529 G 4948 Torulaspora

0.01 2915010 2915010 S 48254 Torulaspora globosa

0.01 2905019 2905019 S 4950 Torulaspora delbrueckii

0.01 4050184 0 G 374469 Vanderwaltozyma

0.01 4050184 0 S 36033 Vanderwaltozyma polyspora

0.01 4050184 4050184 S1 436907 Vanderwaltozyma polyspora DSM 70294

0.01 3676834 0 G 374468 Nakaseomyces

0.01 3676834 0 G1 600669 Nakaseomyces/Candida clade

0.01 3676834 3676834 S 5478 [Candida] glabrata

0.01 3151961 0 G 1196389 Zygotorulaspora

0.01 3151961 3151961 S 42260 Zygotorulaspora mrakii

0.01 3016846 0 G 4953 Zygosaccharomyces

0.01 3016846 3016846 S 4956 Zygosaccharomyces rouxii

0.09 26242331 3912 F 1156497 Pichiaceae

0.04 12577453 1193938 G 461281 Ogataea

0.01 2817685 2817685 S 1378263 Ogataea philodendri

0.01 2398032 2398032 S 1937702 Ogataea haglerorum

0.01 2337414 2337414 S 870730 Ogataea angusta

0.01 1989426 1989426 S 460523 Ogataea polymorpha

0.01 1840958 0 S 1005962 Ogataea parapolymorpha

0.01 1840958 1840958 S1 871575 Ogataea parapolymorpha DL-1

0.02 6942931 1190 G 13366 Brettanomyces

0.01 3835670 3835670 S 5007 Brettanomyces bruxellensis

0.01 3106071 3106071 S 13502 Brettanomyces nanus

0.02 6718035 2068 G 4919 Pichia

0.01 3495100 0 S 4926 Pichia membranifaciens

0.01 3495100 3495100 S1 763406 Pichia membranifaciens NRRL Y-2026

0.01 3220867 3220867 S 4909 Pichia kudriavzevii

0.06 19285448 2409 F 34353 Dipodascaceae

0.03 8180602 0 G 27316 Galactomyces

0.03 8180602 8180602 S 56416 Galactomyces citri-aurantii

0.02 6289153 0 G 4951 Yarrowia

0.02 6289153 0 S 4952 Yarrowia lipolytica

0.02 6289153 6289153 S1 284591 Yarrowia lipolytica CLIB122

0.02 4813284 0 G 1232588 Saprochaete

0.02 4813284 4813284 S 2606893 Saprochaete ingens

0.05 15626624 1612 F 115784 Phaffomycetaceae

0.03 8714658 35885 G 599737 Wickerhamomyces

0.02 4603344 4603344 S 1041607 Wickerhamomyces ciferrii

0.01 4075429 0 S 4927 Wickerhamomyces anomalus

0.01 4075429 4075429 S1 683960 Wickerhamomyces anomalus NRRL Y-366-8

0.01 3978807 0 G 604195 Cyberlindnera

0.01 3978807 0 S 4903 Cyberlindnera jadinii

0.01 3978807 3978807 S1 983966 Cyberlindnera jadinii NRRL Y-1542

0.01 2931547 0 G 460517 Komagataella

0.01 2931547 0 S 460519 Komagataella phaffii

0.01 2931547 2931547 S1 644223 Komagataella phaffii GS115

0.03 7894047 96 O1 241407 Saccharomycetales incertae sedis

0.01 4266073 0 G 1910789 Diutina

0.01 4266073 4266073 S 5481 Diutina rugosa

0.01 3627878 0 G 317045 Kuraishia

0.01 3627878 0 S 317047 Kuraishia capsulata

0.01 3627878 3627878 S1 1382522 Kuraishia capsulata CBS 1993

0.02 7302255 182 F 410830 Trichomonascaceae

0.02 4827837 0 G 410829 Sugiyamaella

0.02 4827837 4827837 S 796027 Sugiyamaella lignohabitans

0.01 2474236 0 G 45787 Wickerhamiella

0.01 2474236 2474236 S 45607 Wickerhamiella sorbophila

0.02 4584821 0 O1 2926619 CUG-Ser2 clade

0.02 4584821 0 F 44277 Ascoideaceae

0.02 4584821 0 G 27308 Ascoidea

0.02 4584821 0 S 54195 Ascoidea rubescens

0.02 4584821 4584821 S1 1344418 Ascoidea rubescens DSM 1968

0.01 3005938 0 F 34365 Saccharomycodaceae

0.01 3005938 0 G 36034 Saccharomycodes

0.01 3005938 3005938 S 36035 Saccharomycodes ludwigii

0.09 27623797 767 P1 451866 Taphrinomycotina

0.05 13776631 0 C 147554 Schizosaccharomycetes

0.05 13776631 0 O 34346 Schizosaccharomycetales

0.05 13776631 0 F 4894 Schizosaccharomycetaceae

0.05 13776631 44732 G 4895 Schizosaccharomyces

0.01 3680275 3680275 S 4896 Schizosaccharomyces pombe

0.01 3406544 0 S 866546 Schizosaccharomyces cryophilus

0.01 3406544 3406544 S1 653667 Schizosaccharomyces cryophilus OY26

0.01 3350152 0 S 4897 Schizosaccharomyces japonicus

0.01 3350152 3350152 S1 402676 Schizosaccharomyces japonicus yFS275

0.01 3294928 0 S 4899 Schizosaccharomyces octosporus

0.01 3294928 3294928 S1 483514 Schizosaccharomyces octosporus yFS286

0.02 5445793 0 C 147553 Pneumocystidomycetes

0.02 5445793 0 O 37987 Pneumocystidales

0.02 5445793 0 F 44281 Pneumocystidaceae

0.02 5445793 199214 G 4753 Pneumocystis

0.01 1974973 0 S 42068 Pneumocystis jirovecii

0.01 1974973 1974973 S1 1408657 Pneumocystis jirovecii RU7

0.01 1660899 0 S 4754 Pneumocystis carinii

0.01 1660899 1660899 S1 1408658 Pneumocystis carinii B80

0.01 1610707 0 S 263815 Pneumocystis murina

0.01 1610707 1610707 S1 1069680 Pneumocystis murina B123

0.01 4418565 0 P2 452562 Taphrinomycotina incertae sedis

0.01 4418565 0 G 5605 Saitoella

0.01 4418565 0 S 5606 Saitoella complicata

0.01 4418565 4418565 S1 698492 Saitoella complicata NRRL Y-17804

0.01 3982041 0 C 147555 Taphrinomycetes

0.01 3982041 0 O 5008 Taphrinales

0.01 3982041 0 F 27330 Protomycetaceae

0.01 3982041 0 G 27331 Protomyces

0.01 3982041 3982041 S 2754530 Protomyces lactucae-debilis

2.46 750168875 93385 P 5204 Basidiomycota

1.86 569502131 31826 P1 5302 Agaricomycotina

1.49 455333837 96469 C 155619 Agaricomycetes

0.82 249374203 19400 C1 452333 Agaricomycetidae

0.41 124680438 11518 O 5338 Agaricales

0.18 56328860 2518 O1 2982316 Marasmiineae

0.06 17115548 0 F 2024004 Mycenaceae

0.06 17115548 0 G 41247 Mycena

0.06 17115548 17115548 S 2126181 Mycena indigotica

0.05 15400057 0 F 862241 Physalacriaceae

0.05 15400057 0 G 866547 Guyanagaster

0.05 15400057 0 S 856835 Guyanagaster necrorhizus

0.05 15400057 15400057 S1 1450536 Guyanagaster necrorhizus MCA 3950

0.04 11986092 0 F 654128 Marasmiaceae

0.04 11986092 0 G 34448 Marasmius

0.04 11986092 11986092 S 181124 Marasmius oreades

0.04 11824645 0 F 72117 Omphalotaceae

0.04 11824645 0 G 5352 Lentinula

0.04 11824645 11824645 S 5353 Lentinula edodes

0.15 46675418 4067 O1 2982305 Agaricineae

0.05 14243654 0 F 330899 Hydnangiaceae

0.05 14243654 0 G 29882 Laccaria

0.05 14243654 0 S 29883 Laccaria bicolor

0.05 14243654 14243654 S1 486041 Laccaria bicolor S238N-H82

0.04 12486764 0 F 40562 Strophariaceae

0.04 12486764 0 G 71950 Psilocybe

0.04 12486764 12486764 S 181762 Psilocybe cubensis

0.04 11101600 0 F 184208 Psathyrellaceae

0.04 11101600 0 G 184431 Coprinopsis

0.04 11101600 0 S 5346 Coprinopsis cinerea

0.04 11101600 11101600 S1 240176 Coprinopsis cinerea okayama7#130

0.03 8839333 0 F 5339 Agaricaceae

0.03 8839333 0 G 5340 Agaricus

0.03 8839333 0 S 5341 Agaricus bisporus

0.03 8839333 0 S1 192523 Agaricus bisporus var. bisporus

0.03 8839333 8839333 S2 936046 Agaricus bisporus var. bisporus H97

0.04 11570744 0 O1 2985477 Schizophyllineae

0.04 11570744 0 F 5332 Schizophyllaceae

0.04 11570744 0 G 5333 Schizophyllum

0.04 11570744 0 S 5334 Schizophyllum commune

0.04 11570744 11570744 S1 578458 Schizophyllum commune H4-8

0.03 10093898 0 O1 2983527 Pleurotineae

0.03 10093898 0 F 104366 Pleurotaceae

0.03 10093898 0 G 5320 Pleurotus

0.03 10093898 10093898 S 5322 Pleurotus ostreatus

0.41 124674365 5740 O 68889 Boletales

0.33 100584111 0 O1 227332 Suillineae

0.33 100584111 0 F 227336 Suillaceae

0.33 100584111 9846154 G 5379 Suillus

0.05 14391986 14391986 S 48578 Suillus paluster

0.04 13260636 13260636 S 48586 Suillus subalutaceus

0.04 12038633 12038633 S 1904413 Suillus clintonianus

0.04 12005751 12005751 S 48587 Suillus subaureus

0.04 10724614 10724614 S 1912939 Suillus fuscotomentosus

0.03 10443204 10443204 S 48563 Suillus bovinus

0.03 9714134 9714134 S 1912936 Suillus discolor

0.03 8158999 8158999 S 116603 Suillus plorans

0.08 24084514 588 O1 227334 Coniophorineae

0.04 12992072 0 F 80634 Coniophoraceae

0.04 12992072 0 G 80635 Coniophora

0.04 12992072 0 S 80637 Coniophora puteana

0.04 12992072 12992072 S1 741705 Coniophora puteana RWD-64-598 SS2

0.04 11091854 0 F 389951 Serpulaceae

0.04 11091854 0 G 80744 Serpula

0.04 11091854 0 S 85982 Serpula lacrymans

0.04 11091854 0 S1 341189 Serpula lacrymans var. lacrymans

0.04 11091854 11091854 S2 578457 Serpula lacrymans var. lacrymans S7.9

0.67 205863165 42876 C1 355688 Agaricomycetes incertae sedis

0.39 119910643 90330 O 5303 Polyporales

0.09 26109790 112729 F 1769247 Fomitopsidaceae

0.05 14917739 0 G 2983002 Neoantrodia

0.05 14917739 14917739 S 139415 Neoantrodia serialis

0.04 11079322 0 G 2066992 Rhodofomes

0.04 11079322 11079322 S 34475 Rhodofomes roseus

0.08 24944631 9796 F 5317 Polyporaceae

0.04 13160744 0 G 5324 Trametes

0.04 13160744 0 S 5325 Trametes versicolor

0.04 13160744 13160744 S1 717944 Trametes versicolor FP-101664 SS1

0.04 11774091 0 G 114154 Dichomitus

0.04 11774091 0 S 114155 Dichomitus squalens

0.04 11774091 11774091 S1 732165 Dichomitus squalens LYAD-421 SS1

0.04 13507868 0 F 378192 Epitheliaceae

0.04 13507868 0 G 378193 Epithele

0.04 13507868 13507868 S 378194 Epithele typhae

0.04 12579241 0 F 396331 Phanerochaetaceae

0.04 12579241 0 G 5305 Phanerochaete

0.04 12579241 0 S 231932 Phanerochaete carnosa

0.04 12579241 12579241 S1 650164 Phanerochaete carnosa HHB-10118-sp

0.04 11602764 0 F 2028212 Laetiporaceae

0.04 11602764 0 G 5629 Laetiporus

0.04 11602764 0 S 5630 Laetiporus sulphureus

0.04 11602764 11602764 S1 1314785 Laetiporus sulphureus 93-53

0.04 11110305 0 F 2983313 Postiaceae

0.04 11110305 0 G 83235 Postia

0.04 11110305 0 S 104341 Postia placenta

0.04 11110305 11110305 S1 670580 Postia placenta MAD-698-R-SB12

0.04 11000866 0 F 40465 Sparassidaceae

0.04 11000866 0 G 40466 Sparassis

0.04 11000866 11000866 S 139825 Sparassis crispa

0.03 8964848 0 F 2983427 Fibroporiaceae

0.03 8964848 0 G 599838 Fibroporia

0.03 8964848 8964848 S 599839 Fibroporia radiculosa

0.10 29681738 180 O 36064 Cantharellales

0.06 18282971 0 F 57201 Cantharellaceae

0.06 18282971 0 G 36065 Cantharellus

0.06 18282971 18282971 S 1750568 Cantharellus anzutake

0.04 11398587 0 F 5250 Ceratobasidiaceae

0.04 11398587 0 G 1322061 Rhizoctonia

0.04 11398587 11398587 S 456999 Rhizoctonia solani

0.08 23541135 1631 O 452342 Russulales

0.05 14069649 0 F 103376 Stereaceae

0.05 14069649 0 G 5644 Stereum

0.05 14069649 0 S 40492 Stereum hirsutum

0.05 14069649 14069649 S1 721885 Stereum hirsutum FP-91666 SS1

0.03 9469855 0 F 40420 Bondarzewiaceae

0.03 9469855 0 G 13562 Heterobasidion

0.03 9469855 0 G1 256003 Heterobasidion annosum species complex

0.03 9469855 0 S 984962 Heterobasidion irregulare

0.03 9469855 9469855 S1 747525 Heterobasidion irregulare TC 32-1

0.04 12334152 0 O 139380 Hymenochaetales

0.04 12334152 0 F 40424 Hymenochaetaceae

0.04 12334152 0 G 167346 Fomitiporia

0.04 12334152 0 S 208960 Fomitiporia mediterranea

0.04 12334152 12334152 S1 694068 Fomitiporia mediterranea MF3/22

0.03 10219329 0 O 452339 Gloeophyllales

0.03 10219329 0 F 452340 Gloeophyllaceae

0.03 10219329 0 G 40443 Gloeophyllum

0.03 10219329 0 S 104355 Gloeophyllum trabeum

0.03 10219329 10219329 S1 670483 Gloeophyllum trabeum ATCC 11539

0.03 10133292 0 O 452338 Corticiales

0.03 10133292 0 F 908827 Punctulariaceae

0.03 10133292 0 G 133746 Punctularia

0.03 10133292 0 S 202698 Punctularia strigosozonata

0.03 10133292 10133292 S1 741275 Punctularia strigosozonata HHB-11173 SS5

0.35 105656121 11550 C 155616 Tremellomycetes

0.25 75931367 6608 O 5234 Tremellales

0.21 62713628 25251 F 1884633 Cryptococcaceae

0.12 35748160 71625 G 490731 Kwoniella

0.03 7708144 0 S 324769 Kwoniella bestiolae

0.03 7708144 7708144 S1 1296100 Kwoniella bestiolae CBS 10118

0.02 7602009 0 S 324770 Kwoniella dejecticola

0.02 7602009 7602009 S1 1296121 Kwoniella dejecticola CBS 10117

0.02 7163303 0 S 463800 Kwoniella mangrovensis

0.02 7163303 7163303 S1 1296122 Kwoniella mangroviensis CBS 8507

0.02 6754067 6754067 S 1734106 Kwoniella shandongensis

0.02 6449012 0 S 453459 Kwoniella pini

0.02 6449012 6449012 S1 1296096 Kwoniella pini CBS 10737

0.09 26940217 2355604 G 5206 Cryptococcus

0.04 10928947 0 G1 1897064 Cryptococcus neoformans species complex

0.04 10928947 568764 S 5207 Cryptococcus neoformans

0.02 5410760 4492005 S1 40410 Cryptococcus neoformans var. neoformans

0.00 472452 472452 S2 214684 Cryptococcus neoformans var. neoformans JEC21

0.00 446303 446303 S2 283643 Cryptococcus neoformans var. neoformans B-3501A

0.02 4949423 0 S1 178876 Cryptococcus neoformans var. grubii

0.02 4949423 4949423 S2 235443 Cryptococcus neoformans var. grubii H99

0.02 5184068 0 G1 1884637 Cryptococcus gattii species complex

0.02 5184068 0 S 37769 Cryptococcus gattii VGI

0.02 5184068 5184068 S1 367775 Cryptococcus gattii WM276

0.01 4318375 0 S 104669 Cryptococcus amylolentus

0.01 4318375 4318375 S1 1295533 Cryptococcus amylolentus CBS 6039

0.01 4153223 0 S 5619 Cryptococcus wingfieldii

0.01 4153223 4153223 S1 1295528 Cryptococcus wingfieldii CBS 7118

0.03 7656983 0 F 5215 Tremellaceae

0.03 7656983 0 G 105767 Tremella

0.03 7656983 0 S 5217 Tremella mesenterica

0.03 7656983 7656983 S1 578456 Tremella mesenterica DSM 1558

0.02 5554148 0 F 1910893 Cuniculitremaceae

0.02 5554148 0 G 4998 Kockovaella

0.02 5554148 5554148 S 4999 Kockovaella imperatae

0.07 21579302 0 O 1851469 Trichosporonales

0.07 21579302 23185 F 1759442 Trichosporonaceae

0.03 7834852 0 G 105983 Apiotrichum

0.03 7834852 7834852 S 105984 Apiotrichum porosum

0.02 7627669 0 G 5552 Trichosporon

0.02 7627669 0 S 82508 Trichosporon asahii

0.02 7627669 0 S1 189963 Trichosporon asahii var. asahii

0.02 7627669 7627669 S2 1186058 Trichosporon asahii var. asahii CBS 2479

0.02 6093596 0 G 1838142 Cutaneotrichosporon

0.02 6093596 6093596 S 879819 Cutaneotrichosporon oleaginosum

0.03 8133902 0 O 90886 Filobasidiales

0.03 8133902 0 F 5408 Filobasidiaceae

0.03 8133902 0 G 5209 Filobasidium

0.03 8133902 8133902 S 5210 Filobasidium floriforme

0.03 8480347 0 C 452332 Dacrymycetes

0.03 8480347 0 O 28997 Dacrymycetales

0.03 8480347 0 F 5254 Dacrymycetaceae

0.03 8480347 0 G 139276 Dacryopinax

0.03 8480347 8480347 S 1858805 Dacryopinax primogenitus

0.31 93332226 46942 P1 452284 Ustilaginomycotina

0.14 42242324 0 C 5257 Ustilaginomycetes

0.14 42242324 0 O 5267 Ustilaginales

0.14 42242324 253023 F 5268 Ustilaginaceae

0.04 12612855 5328 G 5269 Ustilago

0.02 6580600 6580600 S 120017 Ustilago hordei

0.02 6026927 0 S 5270 Ustilago maydis

0.02 6026927 6026927 S1 237631 Ustilago maydis 521

0.04 12501681 3092 G 63298 Pseudozyma

0.02 6785881 0 S 84751 Pseudozyma flocculosa

0.02 6785881 6785881 S1 1277687 Pseudozyma flocculosa PF-1

0.02 5712708 0 S 327079 Pseudozyma hubeiensis

0.02 5712708 5712708 S1 1305764 Pseudozyma hubeiensis SY62

0.02 5979970 0 G 63265 Sporisorium

0.02 5979970 5979970 S 280036 Sporisorium graminicola

0.02 5513525 0 G 63261 Moesziomyces

0.02 5513525 5513525 S 84753 Moesziomyces antarcticus

0.02 5381270 0 G 1804794 Kalmanozyma

0.02 5381270 0 S 1392244 Kalmanozyma brasiliensis

0.02 5381270 5381270 S1 1365824 Kalmanozyma brasiliensis GHG001

0.13 40932593 9522 C 452283 Exobasidiomycetes

0.04 11378305 101 O 5404 Exobasidiales

0.02 5964493 0 F 62920 Cryptobasidiaceae

0.02 5964493 0 G 215249 Acaromyces

0.02 5964493 5964493 S 215250 Acaromyces ingoldii

0.02 5413711 0 F 190068 Brachybasidiaceae

0.02 5413711 0 G 215251 Meira

0.02 5413711 5413711 S 1280837 Meira miltonrushii

0.03 10565832 0 O 162475 Microstromatales

0.03 10565832 1511 O1 162477 Microstromatales incertae sedis

0.02 5375659 0 G 1981958 Pseudomicrostroma

0.02 5375659 5375659 S 1684307 Pseudomicrostroma glucosiphilum

0.02 5188662 0 G 561108 Jaminaea

0.02 5188662 5188662 S 1569628 Jaminaea rosea

0.02 7556691 0 O 742846 Ceraceosorales

0.02 7556691 0 F 742847 Ceraceosoraceae

0.02 7556691 0 G 401624 Ceraceosorus

0.02 7556691 7556691 S 1522189 Ceraceosorus guamensis

0.02 5954115 0 O 62913 Georgefischeriales

0.02 5954115 0 F 62919 Tilletiariaceae

0.02 5954115 0 G 5280 Tilletiaria

0.02 5954115 0 S 5281 Tilletiaria anomala

0.02 5954115 5954115 S1 1037660 Tilletiaria anomala UBC 951

0.02 5468128 0 O 62914 Entylomatales

0.02 5468128 0 O1 162479 Entylomatales incertae sedis

0.02 5468128 0 G 1500560 Tilletiopsis

0.02 5468128 5468128 S 58919 Tilletiopsis washingtonensis

0.03 10110367 0 C 1538075 Malasseziomycetes

0.03 10110367 0 O 162474 Malasseziales

0.03 10110367 0 F 742845 Malasseziaceae

0.03 10110367 13226 G 55193 Malassezia

0.01 2822162 0 S 76773 Malassezia globosa

0.01 2822162 2822162 S1 425265 Malassezia globosa CBS 7966

0.01 2580403 2580403 S 77020 Malassezia pachydermatis

0.01 2381895 0 S 76777 Malassezia sympodialis

0.01 2381895 2381895 S1 1230383 Malassezia sympodialis ATCC 42132

0.01 2312681 2312681 S 76775 Malassezia restricta

0.27 81136085 553 P1 29000 Pucciniomycotina

0.21 65061399 0 C 162484 Pucciniomycetes

0.21 65061399 3311 O 5258 Pucciniales

0.13 39373711 0 F 5262 Pucciniaceae

0.13 39373711 31488 G 5296 Puccinia

0.07 19876498 0 S 27350 Puccinia striiformis

0.07 19876498 19876498 S1 168172 Puccinia striiformis f. sp. tritici

0.06 19465725 0 S 5297 Puccinia graminis

0.06 19465725 0 S1 56615 Puccinia graminis f. sp. tritici

0.06 19465725 19465725 S2 418459 Puccinia graminis f. sp. tritici CRL 75-36-700-3

0.08 25684377 0 F 5259 Melampsoraceae

0.08 25684377 0 G 5260 Melampsora

0.08 25684377 0 S 203908 Melampsora laricis-populina

0.08 25684377 25684377 S1 747676 Melampsora larici-populina 98AG31

0.04 11847428 0 C 162481 Microbotryomycetes

0.04 11847428 0 O 231213 Sporidiobolales

0.04 11847428 0 F 1799696 Sporidiobolaceae

0.04 11847428 19121 G 5533 Rhodotorula

0.02 6204795 0 S 5286 Rhodotorula toruloides

0.02 6204795 6204795 S1 1130832 Rhodotorula toruloides NP11

0.02 5623512 0 S 29898 Rhodotorula graminis

0.02 5623512 5623512 S1 578459 Rhodotorula graminis WP1

0.01 4226705 0 C 432025 Mixiomycetes

0.01 4226705 0 O 432026 Mixiales

0.01 4226705 0 F 165795 Mixiaceae

0.01 4226705 0 G 34348 Mixia

0.01 4226705 0 S 34349 Mixia osmundae

0.01 4226705 4226705 S1 764103 Mixia osmundae IAM 14324

0.02 6105048 0 P1 2204096 Wallemiomycotina

0.02 6105048 0 C 431957 Wallemiomycetes

0.02 6105048 0 O 431958 Wallemiales

0.02 6105048 0 F 431959 Wallemiaceae

0.02 6105048 4158 G 148959 Wallemia

0.01 3095352 0 S 1708541 Wallemia mellicola

0.01 3095352 3095352 S1 671144 Wallemia mellicola CBS 633.66

0.01 3005538 0 S 245174 Wallemia ichthyophaga

0.01 3005538 3005538 S1 1299270 Wallemia ichthyophaga EXF-994

0.35 106661374 1920 K1 112252 Fungi incertae sedis

0.22 66186024 1542 P 1913637 Mucoromycota

0.10 29398153 0 P1 451507 Mucoromycotina

0.10 29398153 0 C 2212703 Mucoromycetes

0.10 29398153 7778 O 4827 Mucorales

0.06 17799152 8423 O1 1344963 Mucorineae

0.03 10528129 0 F 34489 Mucoraceae

0.03 10528129 0 G 4830 Mucor

0.03 10528129 10528129 S 36080 Mucor circinelloides

0.02 7262600 0 F 1344955 Rhizopodaceae

0.02 7262600 0 G 4842 Rhizopus

0.02 7262600 0 S 58291 Rhizopus microsporus

0.02 7262600 7262600 S1 1340429 Rhizopus microsporus ATCC 52813

0.04 11591223 0 F 1344966 Phycomycetaceae

0.04 11591223 0 G 4836 Phycomyces

0.04 11591223 0 S 4837 Phycomyces blakesleeanus

0.04 11591223 11591223 S1 763407 Phycomyces blakesleeanus NRRL 1555(-)

0.09 26250763 0 P1 214504 Glomeromycotina

0.09 26250763 0 C 214506 Glomeromycetes

0.09 26250763 0 O 36750 Glomerales

0.09 26250763 0 F 36751 Glomeraceae

0.09 26250763 0 G 1129544 Rhizophagus

0.09 26250763 0 S 588596 Rhizophagus irregularis

0.09 26250763 26250763 S1 747089 Rhizophagus irregularis DAOM 181602=DAOM 197198

0.03 10535566 0 P1 1137986 Mortierellomycotina

0.03 10535566 0 C 2212732 Mortierellomycetes

0.03 10535566 0 O 214503 Mortierellales

0.03 10535566 0 F 4854 Mortierellaceae

0.03 10535566 0 G 299330 Lobosporangium

0.03 10535566 10535566 S 64571 Lobosporangium transversale

0.07 22320930 0 P 4761 Chytridiomycota

0.07 22320930 0 P1 2683659 Chytridiomycota incertae sedis

0.07 22320930 320 C 451435 Chytridiomycetes

0.03 8342548 0 O 2231171 Synchytriales

0.03 8342548 0 F 286113 Synchytriaceae

0.03 8342548 0 G 286114 Synchytrium

0.03 8342548 8342548 S 1806994 Synchytrium microbalum

0.02 7362556 0 O 34478 Spizellomycetales

0.02 7362556 0 F 34479 Spizellomycetaceae

0.02 7362556 0 G 4815 Spizellomyces

0.02 7362556 0 S 109760 Spizellomyces punctatus

0.02 7362556 7362556 S1 645134 Spizellomyces punctatus DAOM BR117

0.02 6615506 0 O 451442 Rhizophydiales

0.02 6615506 0 O1 1142503 Rhizophydiales incertae sedis

0.02 6615506 0 G 100474 Batrachochytrium

0.02 6615506 0 S 109871 Batrachochytrium dendrobatidis

0.02 6615506 6615506 S1 684364 Batrachochytrium dendrobatidis JAM81

0.04 11568764 999 P 6029 Microsporidia

0.02 5058648 84 P1 6032 Apansporoblastina

0.01 2817557 0 F 36734 Unikaryonidae

0.01 2817557 18368 G 6033 Encephalitozoon

0.00 731590 0 S 6035 Encephalitozoon cuniculi

0.00 731590 731590 S1 284813 Encephalitozoon cuniculi GB-M1

0.00 691414 0 S 58839 Encephalitozoon intestinalis

0.00 691414 691414 S1 876142 Encephalitozoon intestinalis ATCC 50506

0.00 690842 0 S 27973 Encephalitozoon hellem

0.00 690842 690842 S1 907965 Encephalitozoon hellem ATCC 50504

0.00 685343 0 S 571949 Encephalitozoon romaleae

0.00 685343 685343 S1 1178016 Encephalitozoon romaleae SJ-2008

0.01 2241007 14 F 27974 Nosematidae

0.00 1289162 0 G 27977 Nosema

0.00 1289162 1289162 S 40302 Nosema ceranae

0.00 951831 0 G 42398 Vittaforma

0.00 951831 0 S 42399 Vittaforma corneae

0.00 951831 951831 S1 993615 Vittaforma corneae ATCC 50505

0.02 4698525 12 P1 469895 Microsporidia incertae sedis

0.01 2290322 982 G 586132 Nematocida

0.00 1150750 1150750 S 1912982 Nematocida major

0.00 1138590 0 S 586133 Nematocida parisii

0.00 1138590 1138590 S1 881290 Nematocida parisii ERTm1

0.01 1691977 0 G 1633384 Mitosporidium

0.01 1691977 1691977 S 1485682 Mitosporidium daphniae

0.00 716214 0 F 174683 Ordosporidae

0.00 716214 0 G 174684 Ordospora

0.00 716214 0 S 174685 Ordospora colligata

0.00 716214 716214 S1 1354746 Ordospora colligata OC4

0.01 1810592 0 P1 6036 Pansporoblastina

0.01 1810592 0 F 35232 Pleistophoridae

0.01 1810592 0 G 35235 Vavraia

0.01 1810592 0 S 103449 Vavraia culicis

0.01 1810592 1810592 S1 948595 Vavraia culicis subsp. floridensis

0.02 6583736 0 P 1913638 Zoopagomycota

0.02 6583736 0 P1 451828 Kickxellomycotina

0.02 6583736 0 C 2219690 Kickxellomycetes

0.02 6583736 0 O 4861 Kickxellales

0.02 6583736 0 F 4862 Kickxellaceae

0.02 6583736 0 G 4867 Linderina

0.02 6583736 6583736 S 61395 Linderina pennispora

4.65 1421713123 0 K 33208 Metazoa

4.65 1421713123 0 K1 6072 Eumetazoa

4.65 1421713123 0 K2 33213 Bilateria

4.65 1421713123 0 K3 33511 Deuterostomia

4.65 1421713123 0 P 7711 Chordata

4.65 1421713123 0 P1 89593 Craniata

4.65 1421713123 0 P2 7742 Vertebrata

4.65 1421713123 0 P3 7776 Gnathostomata

4.65 1421713123 0 P4 117570 Teleostomi

4.65 1421713123 0 P5 117571 Euteleostomi

4.65 1421713123 0 P6 8287 Sarcopterygii

4.65 1421713123 0 P7 1338369 Dipnotetrapodomorpha

4.65 1421713123 0 P8 32523 Tetrapoda

4.65 1421713123 0 P9 32524 Amniota

4.65 1421713123 0 C 40674 Mammalia

4.65 1421713123 0 C1 32525 Theria

4.65 1421713123 0 C2 9347 Eutheria

4.65 1421713123 0 C3 1437010 Boreoeutheria

4.65 1421713123 2283309 C4 314146 Euarchontoglires

2.36 720096889 0 O 9443 Primates

2.36 720096889 0 O1 376913 Haplorrhini

2.36 720096889 0 O2 314293 Simiiformes

2.36 720096889 0 O3 9526 Catarrhini

2.36 720096889 0 O4 314295 Hominoidea

2.36 720096889 0 F 9604 Hominidae

2.36 720096889 0 F1 207598 Homininae

2.36 720096889 0 G 9605 Homo

2.36 720096889 720096889 S 9606 Homo sapiens

2.29 699332925 0 C5 314147 Glires

2.29 699332925 0 O 9989 Rodentia

2.29 699332925 0 O1 1963758 Myomorpha

2.29 699332925 0 O2 337687 Muroidea

2.29 699332925 0 F 10066 Muridae

2.29 699332925 0 F1 39107 Murinae

2.29 699332925 0 G 10088 Mus

2.29 699332925 0 G1 862507 Mus

2.29 699332925 699332925 S 10090 Mus musculus

1.55 475028846 4669 D1 2698737 Sar

0.95 290662954 6674 D2 33630 Alveolata

0.75 228244469 5470 P 5794 Apicomplexa

0.43 131666445 124 C 1280412 Conoidasida

0.42 127677096 0 C1 5796 Coccidia

0.42 127677096 0 O 75739 Eucoccidiorida

0.42 127677096 2854 O1 423054 Eimeriorina

0.21 63121662 5344 F 5799 Eimeriidae

0.16 49894495 1666513 G 5800 Eimeria

0.04 10769378 10769378 S 44415 Eimeria mitis

0.03 9806233 9806233 S 51315 Eimeria necatrix

0.03 9552104 9552104 S 5804 Eimeria maxima

0.03 9052187 9052187 S 5802 Eimeria tenella

0.03 9048080 9048080 S 5801 Eimeria acervulina

0.04 13221823 0 G 44417 Cyclospora

0.04 13221823 13221823 S 88456 Cyclospora cayetanensis

0.18 53544025 49513 F 5809 Sarcocystidae

0.06 18707202 0 G 5810 Toxoplasma

0.06 18707202 0 S 5811 Toxoplasma gondii

0.06 18707202 18707202 S1 508771 Toxoplasma gondii ME49

0.06 17512321 0 G 94642 Besnoitia

0.06 17512321 17512321 S 94643 Besnoitia besnoiti

0.06 17274989 0 G 29175 Neospora

0.06 17274989 0 S 29176 Neospora caninum

0.06 17274989 17274989 S1 572307 Neospora caninum Liverpool

0.04 11008555 0 F 35082 Cryptosporidiidae

0.04 11008555 1552969 G 5806 Cryptosporidium

0.01 2641712 0 S 5808 Cryptosporidium muris

0.01 2641712 2641712 S1 441375 Cryptosporidium muris RN66

0.01 2517579 2517579 S 857276 Cryptosporidium ubiquitum

0.01 2182591 2182591 S 93969 Cryptosporidium meleagridis

0.00 1122501 0 S 5807 Cryptosporidium parvum

0.00 1122501 1122501 S1 353152 Cryptosporidium parvum Iowa II

0.00 991203 0 S 237895 Cryptosporidium hominis

0.00 991203 991203 S1 353151 Cryptosporidium hominis TU502

0.01 3989225 0 C1 35086 Gregarinasina

0.01 3989225 0 O 35087 Eugregarinorida

0.01 3989225 0 F 35088 Gregarinidae

0.01 3989225 0 G 35089 Gregarina

0.01 3989225 3989225 S 110365 Gregarina niphandrodes

0.32 96572554 1291 C 422676 Aconoidasida

0.23 71229648 0 O 5819 Haemosporida

0.23 71229648 0 F 1639119 Plasmodiidae

0.23 71229648 869930 G 5820 Plasmodium

0.14 42291239 924947 G1 418103 Plasmodium (Plasmodium)

0.02 6756279 6756279 S 77519 Plasmodium gonderi

0.02 6410851 0 S 52288 Plasmodium inui

0.02 6410851 6410851 S1 1237626 Plasmodium inui San Antonio 1

0.02 6181596 6181596 S 5855 Plasmodium vivax

0.02 5684513 5684513 S 5858 Plasmodium malariae

0.02 5462644 0 S 5827 Plasmodium cynomolgi

0.02 5462644 5462644 S1 1120755 Plasmodium cynomolgi strain B

0.02 5451393 0 S 5850 Plasmodium knowlesi

0.02 5451393 5451393 S1 5851 Plasmodium knowlesi strain H

0.02 5419016 5419016 S 5857 Plasmodium fragile

0.04 11167489 1365645 G1 418101 Plasmodium (Vinckeia)

0.01 2663817 0 S 5825 Plasmodium chabaudi

0.01 2663817 2663817 S1 31271 Plasmodium chabaudi chabaudi

0.01 2489990 2489990 S 5861 Plasmodium yoelii

0.01 2416646 0 S 5860 Plasmodium vinckei

0.01 2416646 2416646 S1 54757 Plasmodium vinckei vinckei

0.01 2231391 0 S 5821 Plasmodium berghei

0.01 2231391 2231391 S1 5823 Plasmodium berghei ANKA

0.02 6408921 6408921 S 208452 Plasmodium coatneyi

0.02 5421506 1333210 G1 418107 Plasmodium (Laverania)

0.01 1936162 1936162 S 5854 Plasmodium reichenowi

0.00 1196190 1196190 S 880535 Plasmodium sp. gorilla clade G2

0.00 955944 955944 S 647221 Plasmodium gaboni

0.02 5070563 97405 G1 418104 Plasmodium (Haemamoeba)

0.01 2491126 2491126 S 5849 Plasmodium gallinaceum

0.01 2482032 2482032 S 85471 Plasmodium relictum

0.08 25341615 1441 O 5863 Piroplasmida

0.05 14429067 0 F 32594 Babesiidae

0.05 14429067 87462 G 5864 Babesia

0.01 3851028 3851028 S 189622 Babesia ovata

0.01 3509812 3509812 S 5866 Babesia bigemina

0.01 2543877 0 S 5865 Babesia bovis

0.01 2543877 2543877 S1 484906 Babesia bovis T2Bo

0.01 2433838 0 G1 323723 unclassified Babesia

0.01 2433838 2433838 S 462227 Babesia sp. Xinjiang

0.01 2003050 0 S 5868 Babesia microti

0.01 2003050 2003050 S1 1133968 Babesia microti strain RI

0.04 10911107 0 F 27994 Theileriidae

0.04 10911107 35565 G 5873 Theileria

0.01 3535739 0 S 5872 Theileria equi

0.01 3535739 3535739 S1 1537102 Theileria equi strain WA

0.01 2715745 0 S 68886 Theileria orientalis

0.01 2715745 2715745 S1 869250 Theileria orientalis strain Shintoku

0.01 2341923 0 S 5875 Theileria parva

0.01 2341923 2341923 S1 333668 Theileria parva strain Muguga

0.01 2282135 2282135 S 5874 Theileria annulata

0.15 44366888 0 P 5878 Ciliophora

0.15 44366888 0 P1 431838 Intramacronucleata

0.15 44366888 2458 C 6020 Oligohymenophorea

0.08 24251392 2004 O 31277 Hymenostomatida

0.06 19596803 0 O1 37093 Tetrahymenina

0.06 19596803 0 F 291294 Tetrahymenidae

0.06 19596803 0 G 5890 Tetrahymena

0.06 19596803 0 S 5911 Tetrahymena thermophila

0.06 19596803 19596803 S1 312017 Tetrahymena thermophila SB210

0.02 4652585 0 O1 37090 Ophryoglenina

0.02 4652585 0 G 5931 Ichthyophthirius

0.02 4652585 4652585 S 5932 Ichthyophthirius multifiliis

0.07 20113038 0 O 33825 Peniculida

0.07 20113038 0 F 340080 Parameciidae

0.07 20113038 0 G 5884 Paramecium

0.07 20113038 0 S 5888 Paramecium tetraurelia

0.07 20113038 20113038 S1 412030 Paramecium tetraurelia strain d4-2

0.06 18044923 0 P 2497438 Perkinsozoa

0.06 18044923 0 P1 27997 Perkinsea

0.06 18044923 0 O 27998 Perkinsida

0.06 18044923 0 F 27999 Perkinsidae

0.06 18044923 0 G 28000 Perkinsus

0.06 18044923 0 S 31276 Perkinsus marinus

0.06 18044923 18044923 S1 423536 Perkinsus marinus ATCC 50983

0.60 184263291 4209 D2 33634 Stramenopiles

0.45 138845965 19407 P 4762 Oomycota

0.28 84346155 0 O 4776 Peronosporales

0.28 84346155 41774 F 4777 Peronosporaceae

0.21 64701479 453657 G 4783 Phytophthora

0.10 30196987 0 S 4787 Phytophthora infestans

0.10 30196987 30196987 S1 403677 Phytophthora infestans T30-4

0.06 18530798 18530798 S 67593 Phytophthora sojae

0.05 15520037 0 S 4792 Phytophthora parasitica

0.05 15520037 15520037 S1 761204 Phytophthora parasitica INRA-310

0.06 19602902 0 G 4780 Plasmopara

0.06 19602902 19602902 S 4781 Plasmopara halstedii

0.18 54480403 0 O 4763 Saprolegniales

0.18 54480403 22133 F 4764 Saprolegniaceae

0.09 28805298 44019 G 100860 Aphanomyces

0.05 16064249 16064249 S 112090 Aphanomyces astaci

0.04 12697030 12697030 S 157072 Aphanomyces invadans

0.08 25652972 1022907 G 4769 Saprolegnia

0.04 13173428 0 S 101203 Saprolegnia parasitica

0.04 13173428 13173428 S1 695850 Saprolegnia parasitica CBS 223.65

0.04 11456637 0 S 112098 Saprolegnia diclina

0.04 11456637 11456637 S1 1156394 Saprolegnia diclina VS20

0.12 36843237 347 D3 2696291 Ochrophyta

0.06 18055479 275 P 2836 Bacillariophyta

0.03 9928838 0 C 33836 Coscinodiscophyceae

0.03 9928838 0 C1 33846 Thalassiosirophycidae

0.03 9928838 0 O 33847 Thalassiosirales

0.03 9928838 0 F 29202 Thalassiosiraceae

0.03 9928838 0 G 35127 Thalassiosira

0.03 9928838 0 S 35128 Thalassiosira pseudonana

0.03 9928838 9928838 S1 296543 Thalassiosira pseudonana CCMP1335

0.03 8126366 0 C 33849 Bacillariophyceae

0.03 8126366 0 C1 33850 Bacillariophycidae

0.03 8126366 0 O 38748 Naviculales

0.03 8125052 0 F 38749 Phaeodactylaceae

0.03 8125052 0 G 2849 Phaeodactylum

0.03 8125052 0 S 2850 Phaeodactylum tricornutum

0.03 8125052 8125052 S1 556484 Phaeodactylum tricornutum CCAP 1055/1

0.00 1314 0 F 67474 Naviculaceae

0.00 1314 0 G 67475 Haslea

0.00 1314 1314 S 67476 Haslea ostrearia

0.04 11230186 0 C 35675 Pelagophyceae

0.04 11230186 0 O 54409 Pelagomonadales

0.04 11230186 0 G 44055 Aureococcus

0.04 11230186 11230186 S 44056 Aureococcus anophagefferens

0.02 7557225 0 C 5747 Eustigmatophyceae

0.02 7557225 0 O 425074 Eustigmatales

0.02 7557225 0 F 425072 Monodopsidaceae

0.02 7557225 0 G 5748 Nannochloropsis

0.02 7557225 0 S 72520 Nannochloropsis gaditana

0.02 7557225 7557225 S1 1093141 Nannochloropsis gaditana CCMP526

0.03 8569880 0 C 2683628 Bigyra

0.03 8569880 0 C1 2683629 Opalozoa

0.03 8569880 0 O 42740 Opalinata

0.03 8569880 0 F 2547934 Blastocystidae

0.03 8569880 1662 G 12967 Blastocystis

0.01 4568565 4568565 S 12968 Blastocystis hominis

0.01 3999653 0 G1 944171 Blastocystis sp. subtypes

0.01 3999653 3999653 S 944170 Blastocystis sp. subtype 4

0.00 97932 0 D2 543769 Rhizaria

0.00 97932 0 P 136419 Cercozoa

0.00 97932 0 C 29197 Chlorarachniophyceae

0.00 97932 0 G 227085 Bigelowiella

0.00 97932 97932 S 227086 Bigelowiella natans

0.37 114052082 250 D1 2611352 Discoba

0.28 86850758 0 P 33682 Euglenozoa

0.28 86850758 0 C 5653 Kinetoplastea

0.28 86850758 0 C1 2704647 Metakinetoplastina

0.28 86850758 0 O 2704949 Trypanosomatida

0.28 86850758 12662 F 5654 Trypanosomatidae

0.15 44757927 26086 F1 1286322 Leishmaniinae

0.12 36366420 230879 G 5658 Leishmania

0.08 24525910 2383227 G1 38568 Leishmania

0.03 7843982 5950478 G2 38574 Leishmania donovani species complex

0.00 961553 0 S 5671 Leishmania infantum

0.00 961553 961553 S1 435258 Leishmania infantum JPCM5

0.00 931951 931951 S 5661 Leishmania donovani

0.02 7420324 0 G2 38582 Leishmania mexicana species complex

0.02 7420324 0 S 5665 Leishmania mexicana

0.02 7420324 7420324 S1 929439 Leishmania mexicana MHOM/GT/2001/U1103

0.02 6878377 0 G2 38581 Leishmania major species complex

0.02 6878377 0 S 5664 Leishmania major

0.02 6878377 6878377 S1 347515 Leishmania major strain Friedlin

0.04 11609631 5587847 G1 37616 Viannia

0.01 3045504 0 G2 37617 Leishmania braziliensis species complex

0.01 3045504 0 S 5660 Leishmania braziliensis

0.01 3045504 3045504 S1 420245 Leishmania braziliensis MHOM/BR/75/M2904

0.01 2976280 0 G2 38579 Leishmania guyanensis species complex

0.01 2976280 2976280 S 5679 Leishmania panamensis

0.03 8365421 0 G 5683 Leptomonas

0.03 8365421 8365421 S 157538 Leptomonas pyrrhocoris

0.14 42080169 156424 G 5690 Trypanosoma

0.06 17745222 7463 G1 669453 Trypanosoma with unspecified subgenus

0.02 6083416 6083416 S 67003 Trypanosoma theileri

0.02 5965957 5965957 S 71804 Trypanosoma grayi

0.02 5688386 5688386 S 83891 Trypanosoma conorhini

0.03 10446722 0 G1 47570 Schizotrypanum

0.03 10446722 0 S 5693 Trypanosoma cruzi

0.03 10446722 10446722 S1 353153 Trypanosoma cruzi strain CL Brener

0.03 8071479 0 G1 39700 Trypanozoon

0.03 8071479 4870062 S 5691 Trypanosoma brucei

0.01 2031644 0 S1 5702 Trypanosoma brucei brucei

0.01 2031644 2031644 S2 185431 Trypanosoma brucei brucei TREU927

0.00 1169773 0 S1 31285 Trypanosoma brucei gambiense

0.00 1169773 1169773 S2 679716 Trypanosoma brucei gambiense DAL972

0.02 5660322 0 G1 39701 Herpetosoma

0.02 5660322 5660322 S 5698 Trypanosoma rangeli

0.09 27201074 0 P 5752 Heterolobosea

0.09 27201074 0 P1 2601529 Tetramitia

0.09 27201074 0 P2 2601530 Eutetramitia

0.09 27201074 0 F 5765 Vahlkampfiidae

0.09 27201074 310405 G 5761 Naegleria

0.03 10387382 10387382 S 5762 Naegleria gruberi

0.03 8523606 8523606 S 51637 Naegleria lovaniensis

0.03 7979681 7979681 S 5763 Naegleria fowleri

0.21 64495120 302 D1 554915 Amoebozoa

0.18 54020294 1099 P 2605435 Evosea

0.12 35717412 0 C 142796 Eumycetozoa

0.12 35717412 6615 C1 33083 Dictyostelia

0.08 24490673 5074 O 2058181 Acytosteliales

0.05 16718199 3180 F 2058183 Acytosteliaceae

0.03 8920812 0 G 133407 Acytostelium

0.03 8920812 0 S 361139 Acytostelium subglobosum

0.03 8920812 8920812 S1 1410327 Acytostelium subglobosum LB1

0.03 7794207 7064 G 2058189 Heterostelium

0.03 7787143 0 S 2086695 Heterostelium album

0.03 7787143 7787143 S1 670386 Heterostelium album PN500

0.03 7767400 0 F 2058184 Cavenderiaceae

0.03 7767400 0 G 2058187 Cavenderia

0.03 7767400 7767400 S 261658 Cavenderia fasciculata

0.04 11220124 0 O 2058949 Dictyosteliales

0.04 11220124 0 F 2058185 Dictyosteliaceae

0.04 11220124 25381 G 5782 Dictyostelium

0.02 5909880 5909880 S 5786 Dictyostelium purpureum

0.02 5284863 3225 S 44689 Dictyostelium discoideum

0.02 5281638 5281638 S1 352472 Dictyostelium discoideum AX4

0.06 18301783 0 P1 555406 Archamoebae

0.06 18301783 0 O 2682482 Mastigamoebida

0.06 18301783 0 F 33084 Entamoebidae

0.06 18301783 1992777 G 5758 Entamoeba

0.03 8378939 0 S 33085 Entamoeba invadens

0.03 8378939 8378939 S1 370355 Entamoeba invadens IP1

0.02 4593160 0 S 46681 Entamoeba dispar

0.02 4593160 4593160 S1 370354 Entamoeba dispar SAW760

0.01 1775535 0 S 5759 Entamoeba histolytica

0.01 1775535 1775535 S1 294381 Entamoeba histolytica HM-1:IMSS

0.01 1561372 0 S 412467 Entamoeba nuttalli

0.01 1561372 1561372 S1 1076696 Entamoeba nuttalli P19

0.03 10474524 0 P 555280 Discosea

0.03 10474524 0 O 1485168 Longamoebia

0.03 10474524 0 O1 555407 Centramoebida

0.03 10474524 0 F 33677 Acanthamoebidae

0.03 10474524 0 G 5754 Acanthamoeba

0.03 10474524 0 S 5755 Acanthamoeba castellanii

0.03 10474524 10474524 S1 1257118 Acanthamoeba castellanii str. Neff

0.08 23889082 0 D1 2608109 Haptista

0.08 23889082 0 P 2830 Haptophyta

0.08 23889082 0 P1 2608131 Prymnesiophyceae

0.08 23889082 0 O 73020 Isochrysidales

0.08 23889082 0 F 418966 Noelaerhabdaceae

0.08 23889082 0 G 2902 Emiliania

0.08 23889082 0 S 2903 Emiliania huxleyi

0.08 23889082 23889082 S1 280463 Emiliania huxleyi CCMP1516

0.07 22348215 6 D1 2611341 Metamonada

0.06 19025876 0 P 5719 Parabasalia

0.06 19025876 0 O 37104 Trichomonadida

0.06 19025876 0 F 181550 Trichomonadidae

0.06 19025876 0 G 5721 Trichomonas

0.06 19025876 0 S 5722 Trichomonas vaginalis

0.06 19025876 19025876 S1 412133 Trichomonas vaginalis G3

0.01 3322333 0 P 207245 Fornicata

0.01 3322333 0 O 5738 Diplomonadida

0.01 3322333 0 F 5739 Hexamitidae

0.01 3322333 0 F1 68459 Giardiinae

0.01 3322333 0 G 5740 Giardia

0.01 3322333 3322333 S 5741 Giardia intestinalis

0.07 21841203 175 C 3027 Cryptophyceae

0.07 21638240 0 O 589342 Pyrenomonadales

0.07 21638240 0 F 589343 Geminigeraceae

0.07 21638240 0 G 55528 Guillardia

0.07 21638240 131662 S 55529 Guillardia theta

0.07 21506578 21506578 S1 905079 Guillardia theta CCMP2712

0.00 202788 27 O 589350 Cryptomonadales

0.00 111252 0 F 2896 Cryptomonadaceae

0.00 111252 0 G 3030 Cryptomonas

0.00 111252 111252 S 2898 Cryptomonas paramecium

0.00 91509 0 F 589351 Hemiselmidaceae

0.00 91509 0 G 77924 Hemiselmis

0.00 91509 91509 S 464988 Hemiselmis andersenii

0.03 7970663 0 D1 554296 Apusozoa

0.03 7970663 0 O 2925400 Apusomonadida

0.03 7970663 0 F 172820 Apusomonadidae

0.03 7970663 0 G 877559 Thecamonas

0.03 7970663 0 S 529818 Thecamonas trahens

0.03 7970663 7970663 S1 461836 Thecamonas trahens ATCC 50062

0.02 4957946 0 P 2763 Rhodophyta

0.02 4957946 0 C 2797 Bangiophyceae

0.02 4957946 0 O 265318 Cyanidiales

0.02 4957946 0 F 265316 Cyanidiaceae

0.02 4957946 0 G 45156 Cyanidioschyzon

0.02 4957946 0 S 45157 Cyanidioschyzon merolae

0.02 4957946 4957946 S1 280699 Cyanidioschyzon merolae strain 10D

33.70 10294820860 19719498 D 2 Bacteria

16.49 5037782144 16814339 P 1224 Proteobacteria

8.04 2455016238 10086051 C 1236 Gammaproteobacteria

1.97 600445685 9771392 O 91347 Enterobacterales

0.95 291174852 24138871 F 543 Enterobacteriaceae

0.18 53911568 1012599 F1 2890311 Klebsiella/Raoultella group

0.15 44860036 13143705 G 570 Klebsiella

0.03 9789013 9426548 S 573 Klebsiella pneumoniae

0.00 250267 214387 S1 72407 Klebsiella pneumoniae subsp. pneumoniae

0.00 11824 11824 S2 1328324 Klebsiella pneumoniae subsp. pneumoniae KPNIH27

0.00 11818 11818 S2 272620 Klebsiella pneumoniae subsp. pneumoniae MGH 78578

0.00 5816 5816 S2 1123862 Klebsiella pneumoniae subsp. pneumoniae Kp13

0.00 3106 3106 S2 1193292 Klebsiella pneumoniae subsp. pneumoniae 1084

0.00 1025 1025 S2 1392499 Klebsiella pneumoniae subsp. pneumoniae 1158

0.00 608 608 S2 1308539 Klebsiella pneumoniae subsp. pneumoniae ATCC 43816

0.00 413 413 S2 1125630 Klebsiella pneumoniae subsp. pneumoniae HS11286

0.00 293 293 S2 1094170 Klebsiella pneumoniae subsp. pneumoniae KPNIH10

0.00 283 283 S2 1225181 Klebsiella pneumoniae subsp. pneumoniae KPNIH24

0.00 239 239 S2 484021 Klebsiella pneumoniae subsp. pneumoniae NTUH-K2044

0.00 153 153 S2 1328325 Klebsiella pneumoniae subsp. pneumoniae KPR0928

0.00 142 142 S2 1406314 Klebsiella pneumoniae subsp. pneumoniae PittNDM01

0.00 92 92 S2 990925 Klebsiella pneumoniae subsp. pneumoniae KPX

0.00 68 68 S2 1087440 Klebsiella pneumoniae subsp. pneumoniae KPNIH1

0.00 86291 622 S1 39831 Klebsiella pneumoniae subsp. rhinoscleromatis

0.00 85669 85669 S2 861365 Klebsiella pneumoniae subsp. rhinoscleromatis SB3432

0.00 6997 6997 S1 1365186 Klebsiella pneumoniae KP-1

0.00 5413 5413 S1 1328373 Klebsiella pneumoniae MGH 39

0.00 4250 4250 S1 574 Klebsiella pneumoniae subsp. ozaenae

0.00 1983 1983 S1 1049565 Klebsiella pneumoniae KCTC 2242

0.00 1675 1675 S1 1244085 Klebsiella pneumoniae CG43

0.00 1611 1611 S1 1304922 Klebsiella pneumoniae 500_1420

0.00 1356 1356 S1 1392500 Klebsiella pneumoniae HK787

0.00 1252 1252 S1 1420012 Klebsiella pneumoniae 30660/NJST258_1

0.00 479 479 S1 1420013 Klebsiella pneumoniae 30684/NJST258_2

0.00 444 444 S1 1284798 Klebsiella pneumoniae DMC1097

0.00 235 235 S1 1284804 Klebsiella pneumoniae UHKPC07

0.00 188 188 S1 941259 Klebsiella pneumoniae U-0608239

0.00 17 17 S1 1087446 Klebsiella pneumoniae FCF1305

0.00 7 7 S1 1087447 Klebsiella pneumoniae FCF3SP

0.01 4019379 3752206 S 1463165 Klebsiella quasipneumoniae

0.00 205669 205669 S1 1667327 Klebsiella quasipneumoniae subsp. quasipneumoniae

0.00 61504 61504 S1 1463164 Klebsiella quasipneumoniae subsp. similipneumoniae

0.01 3735454 3726673 S 548 Klebsiella aerogenes

0.00 7913 7913 S1 935296 Klebsiella aerogenes EA1509E

0.00 868 868 S1 1028307 Klebsiella aerogenes KCTC 2190

0.01 3720113 3717873 S 571 Klebsiella oxytoca

0.00 2209 2209 S1 1333852 Klebsiella oxytoca KONIH1

0.00 31 31 S1 795470 Klebsiella oxytoca KOX105

0.01 2741386 2716240 S 1134687 Klebsiella michiganensis

0.00 14815 14815 S1 1308980 Klebsiella michiganensis HKOPL1

0.00 6902 6902 S1 1006551 Klebsiella michiganensis KCTC 1686

0.00 3429 3429 S1 1191061 Klebsiella michiganensis E718

0.01 2399826 2083436 S 244366 Klebsiella variicola

0.00 203047 203047 S1 2489014 Klebsiella variicola subsp. tropica

0.00 93323 93323 S1 2590157 Klebsiella variicola subsp. variicola

0.00 20020 20020 S1 640131 Klebsiella variicola At-22

0.01 1979982 1209814 G1 2608929 unclassified Klebsiella

0.00 159323 159323 S 2834819 Klebsiella sp. A52

0.00 141011 141011 S 2851540 Klebsiella sp. PL-2018

0.00 88459 88459 S 2873296 Klebsiella sp. CTHL.F3a

0.00 73967 73967 S 2787706 Klebsiella sp. BDA134-6

0.00 52662 52662 S 2675710 Klebsiella sp. WP3-W18-ESBL-02

0.00 49331 49331 S 2267618 Klebsiella sp. P1CD1

0.00 43388 43388 S 2675713 Klebsiella sp. WP4-W18-ESBL-05

0.00 41068 41068 S 2697371 Klebsiella sp. MPUS7

0.00 39023 39023 S 2488567 Klebsiella sp. FDAARGOS_511

0.00 30667 30667 S 1905288 Klebsiella sp. LTGPAF-6F

0.00 27997 27997 S 2675711 Klebsiella sp. WP3-S18-ESBL-05

0.00 9332 9332 S 2015795 Klebsiella sp. LY

0.00 3042 3042 S 2897413 Klebsiella sp. KPN54798

0.00 2877 2877 S 2686364 Klebsiella sp. PCX

0.00 2132 2132 S 2675718 Klebsiella sp. WP7-S18-ESBL-04

0.00 2127 2127 S 1934254 Klebsiella sp. M5al

0.00 1255 1255 S 2675716 Klebsiella sp. WP7-S18-CRE-02

0.00 1212 1212 S 2675717 Klebsiella sp. WP7-S18-CRE-03

0.00 573 573 S 2829836 Klebsiella sp. P1927

0.00 397 397 S 2829835 Klebsiella sp. P1954

0.00 186 186 S 160057 Klebsiella sp. KCL-2

0.00 139 139 S 576 Klebsiella sp.

0.00 1318400 1318400 S 2153354 Klebsiella huaxiensis

0.00 872970 872970 S 2058152 Klebsiella grimontii

0.00 558299 558299 S 2489010 Klebsiella africana

0.00 533309 533309 S 2026240 Klebsiella quasivariicola

0.00 48200 48200 S 2587529 Klebsiella pasteurii

0.03 8038933 3329770 G 160674 Raoultella

0.01 1767705 1742515 S 54291 Raoultella ornithinolytica

0.00 25190 25190 S1 1286170 Raoultella ornithinolytica B6

0.00 1237140 1237140 S 575 Raoultella planticola

0.00 813163 171 G1 2627600 unclassified Raoultella

0.00 753865 753865 S 2923366 Raoultella sp. HC6

0.00 30834 30834 S 2259647 Raoultella sp. X13

0.00 28293 28293 S 2799495 Raoultella sp. XY-1

0.00 590765 590765 S 577 Raoultella terrigena

0.00 300390 300390 S 1259973 Raoultella electrica

0.17 51526027 11435116 G 547 Enterobacter

0.09 27902405 6202829 G1 354276 Enterobacter cloacae complex

0.02 6432667 6256989 S 550 Enterobacter cloacae

0.00 96160 0 S1 69219 Enterobacter cloacae subsp. dissolvens

0.00 96160 96160 S2 1104326 Enterobacter cloacae subsp. dissolvens SDM

0.00 78371 75328 S1 336306 Enterobacter cloacae subsp. cloacae

0.00 3043 3043 S2 716541 Enterobacter cloacae subsp. cloacae ATCC 13047

0.00 1147 1147 S1 1333850 Enterobacter cloacae ECNIH2

0.01 4097540 3749411 S 158836 Enterobacter hormaechei

0.00 182486 182486 S1 299766 Enterobacter hormaechei subsp. steigerwaltii

0.00 82199 80467 S1 1812934 Enterobacter hormaechei subsp. hoffmannii

0.00 995 995 S2 1333851 Enterobacter hormaechei subsp. hoffmannii ECNIH3

0.00 737 737 S2 1333849 Enterobacter hormaechei subsp. hoffmannii ECR091

0.00 70144 70144 S1 1296536 Enterobacter hormaechei subsp. xiangfangensis

0.00 8669 8669 S1 301105 Enterobacter hormaechei subsp. hormaechei

0.00 4631 4631 S1 301102 Enterobacter hormaechei subsp. oharae

0.01 2443829 2441213 S 61645 Enterobacter asburiae

0.00 2616 2616 S1 1421338 Enterobacter asburiae L1

0.01 1882349 1882349 S 69218 Enterobacter cancerogenus

0.01 1849924 1849924 S 1812935 Enterobacter roggenkampii

0.01 1630530 1630530 S 299767 Enterobacter ludwigii

0.00 1054234 1054234 S 208224 Enterobacter kobei

0.00 1018903 43305 G2 2757714 unclassified Enterobacter cloacae complex

0.00 363373 363373 S 2027919 Enterobacter cloacae complex sp.

0.00 129869 129869 S 1329841 Enterobacter sp. BIDMC 29

0.00 96332 96332 S 2969431 Enterobacter cloacae complex sp. R_G8

0.00 89798 89798 S 1915310 Enterobacter cloacae complex sp. ECNIH7

0.00 64556 64556 S 1686399 Enterobacter sp. BIDMC100

0.00 61896 61896 S 2077136 Enterobacter cloacae complex sp. FDA-CDC-AR_0164

0.00 54646 54646 S 2077137 Enterobacter cloacae complex sp. FDA-CDC-AR_0132

0.00 50602 50602 S 2912628 Enterobacter cloacae complex sp. ECL414

0.00 20370 20370 S 2912630 Enterobacter cloacae complex sp. ECL405

0.00 11968 11968 S 2876028 Enterobacter cloacae complex sp. ECL352

0.00 7478 7478 S 2850078 Enterobacter cloacae complex sp. ECL72

0.00 5451 5451 S 2850492 Enterobacter cloacae complex sp. ECL78

0.00 4588 4588 S 2850494 Enterobacter cloacae complex sp. ECL68

0.00 4154 4154 S 1329836 Enterobacter sp. BWH 37

0.00 2621 2621 S 2850493 Enterobacter cloacae complex sp. ECL112

0.00 2331 2331 S 2912629 Enterobacter cloacae complex sp. ECL411

0.00 1662 1662 S 2912631 Enterobacter cloacae complex sp. ECL404

0.00 1099 1099 S 1329816 Enterobacter sp. MGH 7

0.00 1096 1096 S 2798729 Enterobacter cloacae complex sp. AR_0002

0.00 911 911 S 1329823 Enterobacter sp. MGH 14

0.00 797 797 S 1329812 Enterobacter sp. MGH 3

0.00 537588 537588 S 2494701 Enterobacter chengduensis

0.00 457288 457288 S 2497875 Enterobacter chuandaensis

0.00 294724 294724 S 2071710 Enterobacter sichuanensis

0.02 7632636 737306 G1 2608935 unclassified Enterobacter

0.00 1176384 1176384 S 2724468 Enterobacter sp. JUb54

0.00 1123275 1123275 S 2742676 Enterobacter sp. RHBSTW-00994

0.00 1068782 1068782 S 1914861 Enterobacter sp. SA187

0.00 768644 768644 S 2742639 Enterobacter sp. RHBSTW-00175

0.00 615492 615492 S 2976431 Enterobacter sp. CP102

0.00 298068 298068 S 2596949 Enterobacter sp. E76

0.00 258838 258838 S 2836161 Enterobacter sp. SGAir0187

0.00 257118 257118 S 2831890 Enterobacter sp. JBIWA003

0.00 220080 220080 S 2866201 Enterobacter sp. Colony194

0.00 172343 172343 S 2831891 Enterobacter sp. JBIWA005

0.00 154505 154505 S 2705458 Enterobacter sp. SES19

0.00 119856 119856 S 2500132 Enterobacter sp. N18-03635

0.00 76446 76446 S 2565914 Enterobacter sp. 18A13

0.00 66248 66248 S 2720029 Enterobacter sp. DNB-S2

0.00 66208 66208 S 2742673 Enterobacter sp. RHBSTW-00975

0.00 59148 59148 S 1868135 Enterobacter sp. HK169

0.00 54442 54442 S 1560339 Enterobacter sp. E20

0.00 51929 51929 S 1827481 Enterobacter sp. ODB01

0.00 44135 44135 S 2747372 Enterobacter sp. DSM 30060

0.00 43079 43079 S 2831892 Enterobacter sp. JBIWA008

0.00 37799 37799 S 2742656 Enterobacter sp. RHBSTW-00593

0.00 37284 37284 S 1848517 Enterobacter sp. LU1

0.00 34078 34078 S 648691 Enterobacter sp. YSU

0.00 29544 29544 S 2894203 Enterobacter sp. AN-K1

0.00 21524 21524 S 1977566 Enterobacter sp. Crenshaw

0.00 16996 16996 S 2980499 Enterobacter sp. 155105

0.00 13617 13617 S 2742646 Enterobacter sp. RHBSTW-00422

0.00 3014 3014 S 2923088 Enterobacter sp. JH25

0.00 2450 2450 S 2051905 Enterobacter sp. CRENT-193

0.00 2328 2328 S 42895 Enterobacter sp.

0.00 1521 1521 S 211595 Enterobacter sp. RFL1396

0.00 147 147 S 1632810 Enterobacter sp. 247

0.00 8 8 S 1027278 Enterobacter sp. W001

0.00 1520970 1520970 S 539813 Enterobacter mori

0.00 840426 840426 S 2494702 Enterobacter huaxiensis

0.00 795502 795502 S 2478464 Enterobacter oligotrophicus

0.00 738851 738851 S 881260 Enterobacter bugandensis

0.00 660121 660121 S 885040 Enterobacter soli

0.12 36617282 11475869 G 544 Citrobacter

0.04 12809383 2878011 G1 1344959 Citrobacter freundii complex

0.02 6376203 6325990 S 546 Citrobacter freundii

0.00 42216 42216 S1 1006003 Citrobacter freundii ATCC 8090 = MTCC 1658 = NBRC 12681

0.00 7997 7997 S1 1333848 Citrobacter freundii CFNIH1

0.00 1130113 1130113 S 1639133 Citrobacter portucalensis

0.00 836796 836796 S 67827 Citrobacter werkmanii

0.00 719766 719766 S 57706 Citrobacter braakii

0.00 384561 23 G2 2816438 unclassified Citrobacter freundii complex

0.00 331867 331867 S 2066049 Citrobacter freundii complex sp. CFNIH2

0.00 27533 27533 S 1686384 Citrobacter sp. BIDMC107

0.00 23313 23313 S 2077147 Citrobacter freundii complex sp. CFNIH3

0.00 1825 1825 S 2529121 Citrobacter sp. ABFQG

0.00 257271 257271 S 67826 Citrobacter sedlakii

0.00 226662 226662 S 133448 Citrobacter youngae

0.02 4645932 731018 G1 2644389 unclassified Citrobacter

0.00 1112355 1112355 S 1573676 Citrobacter sp. R56

0.00 1007840 1007840 S 2742624 Citrobacter sp. RHB25-C09

0.00 417751 417751 S 2781952 Citrobacter sp. BDA59-3

0.00 102548 102548 S 1439319 Citrobacter sp. MGH 55

0.00 97444 97444 S 1703250 Citrobacter sp. CRE-46

0.00 96624 96624 S 1920110 Citrobacter sp. CFNIH10

0.00 78933 78933 S 184912 Citrobacter sp. TSA-1

0.00 77547 77547 S 2742657 Citrobacter sp. RHBSTW-00599

0.00 75209 75209 S 1702170 Citrobacter sp. FDAARGOS_156

0.00 74339 74339 S 2742677 Citrobacter sp. RHBSTW-01013

0.00 74078 74078 S 2742653 Citrobacter sp. RHBSTW-00524

0.00 70009 70009 S 2742625 Citrobacter sp. RHB35-C17

0.00 66821 66821 S 2742626 Citrobacter sp. RHB35-C21

0.00 63182 63182 S 2742675 Citrobacter sp. RHBSTW-00986

0.00 54873 54873 S 2942480 Citrobacter sp. XT1-2-2

0.00 48503 48503 S 2566012 Citrobacter sp. CF971

0.00 45987 45987 S 2742627 Citrobacter sp. RHB36-C18

0.00 45597 45597 S 2683822 Citrobacter sp. 172116965

0.00 44757 44757 S 2861802 Citrobacter sp. Colony219

0.00 35970 35970 S 2697370 Citrobacter sp. LUTT5

0.00 35444 35444 S 2742655 Citrobacter sp. RHBSTW-00570

0.00 28294 28294 S 2716879 Citrobacter sp. Y3

0.00 23892 23892 S 2861801 Citrobacter sp. Colony322

0.00 20823 20823 S 2861800 Citrobacter sp. Colony475

0.00 17618 17618 S 2742678 Citrobacter sp. RHBSTW-01044

0.00 15425 15425 S 2742671 Citrobacter sp. RHBSTW-00944

0.00 13931 13931 S 2742636 Citrobacter sp. RHBSTW-00127

0.00 13672 13672 S 2742670 Citrobacter sp. RHBSTW-00903

0.00 10682 10682 S 2742641 Citrobacter sp. RHBSTW-00229

0.00 10062 10062 S 2742654 Citrobacter sp. RHBSTW-00535

0.00 8640 8640 S 2576406 Citrobacter sp. TBCP-5362

0.00 8361 8361 S 2742630 Citrobacter sp. RHBSTW-00021

0.00 4685 4685 S 2712024 Citrobacter sp. SX212

0.00 4672 4672 S 2742629 Citrobacter sp. RHBSTW-00017

0.00 3229 3229 S 2742647 Citrobacter sp. RHBSTW-00424

0.00 1859 1859 S 2742621 Citrobacter sp. RHB20-C16

0.00 1773 1773 S 2742620 Citrobacter sp. RHB20-C15

0.00 1066 1066 S 2712025 Citrobacter sp. SX206

0.00 194 194 S 1036018 Citrobacter sp. 36-4CPA

0.00 120 120 S 1896336 Citrobacter sp.

0.00 54 54 S 2742622 Citrobacter sp. RHB21-C01

0.00 51 51 S 2742623 Citrobacter sp. RHB21-C05

0.01 1540481 605210 S 35703 Citrobacter amalonaticus

0.00 935271 935271 S1 1261127 Citrobacter amalonaticus Y19

0.00 1459581 1459581 S 67824 Citrobacter farmeri

0.00 1142965 1131298 S 67825 Citrobacter rodentium

0.00 8513 8513 S1 637910 Citrobacter rodentium ICC168

0.00 3154 3154 S1 1218085 Citrobacter rodentium NBRC 105723 = DSM 16636

0.00 1034367 1032586 S 545 Citrobacter koseri

0.00 1781 1781 S1 290338 Citrobacter koseri ATCC BAA-895

0.00 935723 935723 S 2562449 Citrobacter tructae

0.00 653780 653780 S 2546350 Citrobacter arsenatis

0.00 639794 639794 S 1563222 Citrobacter pasteurii

0.00 279407 279407 S 1748967 Citrobacter cronae

0.09 28749221 2161423 G 561 Escherichia

0.07 20184502 19150894 S 562 Escherichia coli

0.00 146413 135796 S1 83334 Escherichia coli O157:H7

0.00 3444 3444 S2 1328859 Escherichia coli O157:H7 str. SS17

0.00 3116 3116 S2 155864 Escherichia coli O157:H7 str. EDL933

0.00 1600 1600 S2 1330457 Escherichia coli O157:H7 str. SS52

0.00 781 781 S2 502346 Escherichia coli O157:H7 str. TW14588

0.00 505 505 S2 1343836 Escherichia coli O157:H7 str. F8092B

0.00 409 409 S2 996639 Escherichia coli O157:H7 str. EC10

0.00 321 321 S2 444450 Escherichia coli O157:H7 str. EC4115

0.00 224 224 S2 386585 Escherichia coli O157:H7 str. Sakai

0.00 217 217 S2 544404 Escherichia coli O157:H7 str. TW14359

0.00 41550 41550 S1 2848144 Escherichia coli O158:H23

0.00 35290 35290 S1 2848143 Escherichia coli O85:H1

0.00 31108 31108 S1 2848145 Escherichia coli O170:H18

0.00 30345 30345 S1 2027293 Escherichia coli O8:H8

0.00 26691 26691 S1 2603836 Escherichia coli O10:H32

0.00 26266 23052 S1 930406 Escherichia coli O157:H16

0.00 3214 3214 S2 1446608 Escherichia coli O157:H16 str. 98-3133

0.00 25137 21572 S1 244320 Escherichia coli O55:H7

0.00 1843 1843 S2 701177 Escherichia coli O55:H7 str. CB9615

0.00 1722 1722 S2 1048689 Escherichia coli O55:H7 str. RM12579

0.00 22778 0 S1 861906 Escherichia coli O44:H18

0.00 22778 22778 S2 216592 Escherichia coli 042

0.00 20759 20759 S1 2778655 Escherichia coli O167:H26

0.00 19705 19705 S1 585397 Escherichia coli ED1a

0.00 19280 19280 S1 2810407 Escherichia coli O126:H45

0.00 18257 18257 S1 2491679 Escherichia coli O22:H8

0.00 18226 18226 S1 2773707 Escherichia coli O19:H7

0.00 18095 18095 S1 409438 Escherichia coli SE11

0.00 17634 17634 S1 696406 Escherichia coli UMNK88

0.00 16154 16154 S1 941322 Escherichia coli O25b:H4-ST131

0.00 14062 14062 S1 1446746 Escherichia coli O6:H16

0.00 13960 13960 S1 2874051 Escherichia coli O99:H6

0.00 13831 13831 S1 2048777 Escherichia coli O15:H11

0.00 13776 13776 S1 2763104 Escherichia coli O150:H6

0.00 13701 13701 S1 362663 Escherichia coli 536

0.00 13630 13630 S1 1050617 Escherichia coli UMNF18

0.00 13203 13203 S1 1392854 Escherichia coli M8

0.00 11300 11300 S1 2773705 Escherichia coli O18ac:H14

0.00 11213 11213 S1 1045010 Escherichia coli O157

0.00 10647 10647 S1 2810404 Escherichia coli O7:H4

0.00 10085 0 S1 1603259 Escherichia coli O139:H28

0.00 10085 10085 S2 331111 Escherichia coli O139:H28 str. E24377A

0.00 9950 9715 S1 1055538 Escherichia coli O145

0.00 235 235 S2 1055544 Escherichia coli O145 str. RM9872

0.00 9622 9622 S1 2778657 Escherichia coli O9:H10

0.00 9534 0 S1 1446738 Escherichia coli O39:NM

0.00 9534 9534 S2 1446739 Escherichia coli O39:NM str. F8704-2

0.00 9157 7045 S1 1038927 Escherichia coli O104:H4

0.00 929 929 S2 1048254 Escherichia coli O104:H4 str. C227-11

0.00 412 412 S2 1133853 Escherichia coli O104:H4 str. 2009EL-2071

0.00 407 407 S2 1134782 Escherichia coli O104:H4 str. 2009EL-2050

0.00 364 364 S2 1133852 Escherichia coli O104:H4 str. 2011C-3493

0.00 9145 9145 S1 2778654 Escherichia coli O152:H23

0.00 9056 9056 S1 2810405 Escherichia coli O89m:H10

0.00 8838 8838 S1 481805 Escherichia coli ATCC 8739

0.00 8351 0 S1 2233553 Escherichia coli O43

0.00 8351 8351 S2 1055541 Escherichia coli O43 str. RM10042

0.00 8337 3672 S1 83333 Escherichia coli K-12

0.00 3074 3074 S2 511145 Escherichia coli str. K-12 substr. MG1655

0.00 598 598 S2 879462 Escherichia coli str. K-12 substr. MG1655star

0.00 486 486 S2 316407 Escherichia coli str. K-12 substr. W3110

0.00 231 231 S2 595496 Escherichia coli BW2952

0.00 176 176 S2 679895 Escherichia coli BW25113

0.00 39 39 S2 1245474 Escherichia coli ER2796

0.00 33 33 S2 316385 Escherichia coli str. K-12 substr. DH10B

0.00 28 28 S2 1403831 Escherichia coli str. K-12 substr. MC4100

0.00 8328 8328 S1 2861806 Escherichia coli O141:H4

0.00 8116 8116 S1 2048781 Escherichia coli O27:H7

0.00 7740 7740 S1 2778656 Escherichia coli O20:H12

0.00 7175 7175 S1 2773704 Escherichia coli O15:H12

0.00 7137 7137 S1 2773706 Escherichia coli O68:H12

0.00 7115 7115 S1 2027295 Escherichia coli O8:H28

0.00 7004 7004 S1 2861805 Escherichia coli O139:H1

0.00 6919 6919 S1 941323 Escherichia coli VR50

0.00 6170 6170 S1 340186 Escherichia coli E110019

0.00 6091 6091 S1 1078032 Escherichia coli O45:H2

0.00 6072 6072 S1 2048778 Escherichia coli O178:H19

0.00 6020 6020 S1 745156 Escherichia coli 1303

0.00 5698 5698 S1 316435 Escherichia coli Nissle 1917

0.00 5607 0 S1 1450174 Escherichia coli O119

0.00 5607 5607 S2 397448 Escherichia coli O119:H6

0.00 5506 4399 S1 1078034 Escherichia coli O145:H28

0.00 315 315 S2 1248902 Escherichia coli O145:H28 str. RM13514

0.00 304 304 S2 1248823 Escherichia coli O145:H28 str. RM12581

0.00 296 296 S2 1248903 Escherichia coli O145:H28 str. RM12761

0.00 192 192 S2 1248915 Escherichia coli O145:H28 str. RM13516

0.00 5127 5127 S1 2969654 Escherichia coli O7:H15

0.00 4892 4892 S1 1358422 Escherichia coli PCN061

0.00 4591 4591 S1 2778653 Escherichia coli O112ab:H8

0.00 4379 4379 S1 1446701 Escherichia coli O169:H41

0.00 4375 4375 S1 1382700 Escherichia coli PMV-1

0.00 4323 4323 S1 585057 Escherichia coli IAI39

0.00 4298 4298 S1 2072463 Escherichia coli O78

0.00 4194 4194 S1 1412834 Escherichia coli FAP1

0.00 4130 4130 S1 2810406 Escherichia coli O89m:H9

0.00 4103 4103 S1 1446600 Escherichia coli O15:H18

0.00 4009 0 S1 1072458 Escherichia coli O7:K1

0.00 4009 4009 S2 1072459 Escherichia coli O7:K1 str. CE10

0.00 3933 3933 S1 2697517 Escherichia coli O84:H7

0.00 3810 3810 S1 1502658 Escherichia coli O2:H6

0.00 3739 3739 S1 1954351 Escherichia coli APEC O2-211

0.00 3702 3702 S1 585055 Escherichia coli 55989

0.00 3685 3685 S1 1355100 Escherichia coli JJ1886

0.00 3651 3651 S1 405955 Escherichia coli APEC O1

0.00 3539 1190 S1 376725 Escherichia coli O103:H2

0.00 2349 2349 S2 585395 Escherichia coli O103:H2 str. 12009

0.00 3277 3277 S1 991919 Escherichia coli O145:NM

0.00 3104 3104 S1 1446707 Escherichia coli O25:NM

0.00 3033 3033 S1 1078021 Escherichia coli O113:H21

0.00 2923 2923 S1 2048776 Escherichia coli O128:H27

0.00 2847 2847 S1 2605620 Escherichia coli O80:H26

0.00 2845 2845 S1 991915 Escherichia coli O121:H19

0.00 2651 2651 S1 2810409 Escherichia coli H20

0.00 2461 2461 S1 2079157 Escherichia coli O2:H1

0.00 2459 2459 S1 941280 Escherichia coli O25b:H4

0.00 2404 2404 S1 591946 Escherichia coli LF82

0.00 2186 2186 S1 2592065 Escherichia coli O1:H42

0.00 2183 1259 S1 168927 Escherichia coli O111:H-

0.00 924 924 S2 585396 Escherichia coli O111:H- str. 11128

0.00 2100 2100 S1 585034 Escherichia coli IAI1

0.00 2044 2044 S1 2126982 Escherichia coli O18:H1

0.00 2003 2003 S1 2491878 Escherichia coli O4:H5

0.00 1976 1976 S1 714962 Escherichia coli IHE3034

0.00 1904 1904 S1 1401688 Escherichia coli APEC O18

0.00 1879 1879 S1 758831 Escherichia coli ECC-1470

0.00 1854 1854 S1 2491692 Escherichia coli O39:H21

0.00 1844 1844 S1 869729 Escherichia coli UM146

0.00 1834 1834 S1 439855 Escherichia coli SMS-3-5

0.00 1830 1830 S1 1200752 Escherichia coli NCCP15648

0.00 1750 1750 S1 199310 Escherichia coli CFT073

0.00 1667 0 S1 685037 Escherichia coli O83:H1

0.00 1667 1667 S2 685038 Escherichia coli O83:H1 str. NRG 857C

0.00 1666 1666 S1 2067421 Escherichia coli O25:H4

0.00 1665 1665 S1 183192 Escherichia coli O157:H-

0.00 1617 1617 S1 1274814 Escherichia coli APEC O78

0.00 1602 1602 S1 1311757 Escherichia coli ACN001

0.00 1584 1584 S1 585056 Escherichia coli UMN026

0.00 1564 0 S1 1055539 Escherichia coli O91

0.00 1564 1564 S2 1055545 Escherichia coli O91 str. RM7190

0.00 1561 1561 S1 1392858 Escherichia coli M12

0.00 1532 1532 S1 566546 Escherichia coli W

0.00 1474 1474 S1 2814546 Escherichia coli O1:HNT

0.00 1453 1453 S1 344610 Escherichia coli 53638

0.00 1451 568 S1 244319 Escherichia coli O26:H11

0.00 883 883 S2 573235 Escherichia coli O26:H11 str. 11368

0.00 1380 1380 S1 2874053 Escherichia coli O25:H1

0.00 1373 1373 S1 577675 Escherichia coli O91:H21

0.00 1371 1371 S1 431946 Escherichia coli SE15

0.00 1346 1032 S1 168807 Escherichia coli O127:H6

0.00 314 314 S2 574521 Escherichia coli O127:H6 str. E2348/69

0.00 1344 13 S1 404399 Escherichia coli O26

0.00 1106 1106 S2 1055534 Escherichia coli O26 str. RM10386

0.00 225 225 S2 1055533 Escherichia coli O26 str. RM8426

0.00 1250 1250 S1 316401 Escherichia coli ETEC H10407

0.00 1218 1218 S1 2605619 Escherichia coli O16:H48

0.00 1216 1216 S1 913088 Escherichia coli TW11681

0.00 1209 1209 S1 2048780 Escherichia coli O25:H16

0.00 1195 1195 S1 1329907 Escherichia coli APEC IMT5155

0.00 1157 1157 S1 331112 Escherichia coli HS

0.00 1142 1142 S1 469008 Escherichia coli BL21(DE3)

0.00 1118 17 S1 1055537 Escherichia coli O121

0.00 1101 1101 S2 1055543 Escherichia coli O121 str. RM8352

0.00 1023 1023 S1 563770 Escherichia coli Vir68

0.00 978 978 S1 2778652 Escherichia coli O100:H21

0.00 976 976 S1 344601 Escherichia coli B171

0.00 956 956 S1 2874052 Escherichia coli O16:H6

0.00 819 819 S1 595495 Escherichia coli KO11FL

0.00 778 0 S1 1055536 Escherichia coli O103

0.00 778 778 S2 1055540 Escherichia coli O103 str. RM8385

0.00 744 744 S1 373045 Escherichia coli O111:NM

0.00 735 735 S1 413997 Escherichia coli B str. REL606

0.00 622 622 S1 866789 Escherichia coli DSM 30083 = JCM 1649 = ATCC 11775

0.00 575 575 S1 1001989 Escherichia coli PCN033

0.00 542 0 S1 1055535 Escherichia coli O111

0.00 542 542 S2 1055542 Escherichia coli O111 str. RM9322

0.00 541 541 S1 2048775 Escherichia coli O114:H49

0.00 521 521 S1 2822132 Escherichia coli O128ac:H12

0.00 496 496 S1 1322345 Escherichia coli ATCC 25922

0.00 485 485 S1 585035 Escherichia coli S88

0.00 383 383 S1 655817 Escherichia coli ABU 83972

0.00 351 351 S1 1441627 Escherichia coli str. Sanji

0.00 330 330 S1 741093 Escherichia coli Xuzhou21

0.00 287 287 S1 1355101 Escherichia coli JJ1887

0.00 268 268 S1 569579 Escherichia coli NU14

0.00 237 237 S1 913091 Escherichia coli TW10598

0.00 202 202 S1 762608 Escherichia coli ETEC 1392/75

0.00 199 199 S1 37762 Escherichia coli B

0.00 199 199 S1 498388 Escherichia coli C

0.00 183 183 S1 536056 Escherichia coli DH1

0.00 181 24 S1 1335302 Escherichia coli O104:H21

0.00 157 157 S2 1335303 Escherichia coli O104:H21 str. CFSAN002236

0.00 179 179 S1 2899430 Escherichia coli O23:H4

0.00 112 112 S1 1619910 Escherichia coli O104:H7

0.00 104 104 S1 1416674 Escherichia coli N37139PS

0.00 101 101 S1 488477 Escherichia coli F18+

0.00 92 92 S1 475609 Escherichia coli chi7122

0.00 92 92 S1 1335916 Escherichia coli LY180

0.00 74 74 S1 866768 Escherichia coli 'BL21-Gold(DE3)pLysS AG'

0.00 74 74 S1 1230480 Escherichia coli EC302/04

0.00 62 62 S1 376724 Escherichia coli O26:H-

0.00 54 54 S1 2079151 Escherichia coli O1:H7

0.00 43 43 S1 1005567 Escherichia coli N1

0.00 41 41 S1 511693 Escherichia coli BL21

0.00 35 35 S1 2491876 Escherichia coli O45:H11

0.00 32 32 S1 885275 Escherichia coli str. 'clone D i14'

0.00 30 30 S1 364106 Escherichia coli UTI89

0.00 22 22 S1 1435461 Escherichia coli KLY

0.00 19 19 S1 1416676 Escherichia coli N40607

0.00 15 15 S1 885276 Escherichia coli str. 'clone D i2'

0.00 6 6 S1 1144303 Escherichia coli J53

0.01 2526029 2520546 S 208962 Escherichia albertii

0.00 5483 5483 S1 1440052 Escherichia albertii KF1

0.01 1913706 1906997 S 564 Escherichia fergusonii

0.00 5513 5513 S1 981367 Escherichia fergusonii ECD227

0.00 1196 1196 S1 585054 Escherichia fergusonii ATCC 35469

0.00 1303139 1303139 S 1499973 Escherichia marmotae

0.00 660422 43 G1 2608889 unclassified Escherichia

0.00 650862 650862 S 2044467 Escherichia sp. E4742

0.00 9078 9078 S 2725997 Escherichia sp. SCLE84

0.00 243 243 S 1884818 Escherichia sp.

0.00 113 113 S 2857061 Escherichia sp. TC-EC600-tetX4

0.00 51 51 S 2860337 Escherichia sp. TM-G17TGC

0.00 32 32 S 299586 Escherichia sp. Sflu5

0.07 20001011 2793312 G 590 Salmonella

0.05 15540121 7487434 S 28901 Salmonella enterica

0.02 5921671 1373194 S1 59201 Salmonella enterica subsp. enterica

0.00 1227261 1222894 S2 119912 Salmonella enterica subsp. enterica serovar Choleraesuis

0.00 2956 2956 S3 321314 Salmonella enterica subsp. enterica serovar Choleraesuis str. SC-B67

0.00 1118 1118 S3 904139 Salmonella enterica subsp. enterica serovar Choleraesuis str. SCSA50

0.00 293 293 S3 938142 Salmonella enterica subsp. enterica serovar Choleraesuis str. ATCC 10708

0.00 256186 0 S2 1242082 Salmonella enterica subsp. enterica serovar India

0.00 256186 256186 S3 1242098 Salmonella enterica subsp. enterica serovar India str. SA20085604

0.00 229467 0 S2 1242085 Salmonella enterica subsp. enterica serovar Macclesfield

0.00 229467 229467 S3 1242107 Salmonella enterica subsp. enterica serovar Macclesfield str. S-1643

0.00 220642 206021 S2 90371 Salmonella enterica subsp. enterica serovar Typhimurium

0.00 4266 4266 S3 568709 Salmonella enterica subsp. enterica serovar Typhimurium str. DT2

0.00 2131 1691 S3 1620419 Salmonella enterica subsp. enterica serovar Typhimurium var. 5-

0.00 440 440 S4 1271862 Salmonella enterica subsp. enterica serovar Typhimurium var. 5- str. CFSAN001921

0.00 2034 2034 S3 1454638 Salmonella enterica subsp. enterica serovar Typhimurium str. USDA-ARS-USMARC-1898

0.00 1410 1410 S3 1029978 Salmonella enterica subsp. enterica serovar Typhimurium str. SARA13

0.00 1082 1082 S3 1008297 Salmonella enterica subsp. enterica serovar Typhimurium str. 798

0.00 445 445 S3 1454647 Salmonella enterica subsp. enterica serovar Typhimurium str. USDA-ARS-USMARC-1810

0.00 435 435 S3 1454636 Salmonella enterica subsp. enterica serovar Typhimurium str. USDA-ARS-USMARC-1896

0.00 369 369 S3 1454643 Salmonella enterica subsp. enterica serovar Typhimurium str. CDC 2011K-1702

0.00 357 357 S3 1454642 Salmonella enterica subsp. enterica serovar Typhimurium str. CDC 2011K-0870

0.00 353 353 S3 1454641 Salmonella enterica subsp. enterica serovar Typhimurium str. CDC 2010K-1587

0.00 293 293 S3 1454634 Salmonella enterica subsp. enterica serovar Typhimurium str. USDA-ARS-USMARC-1880

0.00 281 281 S3 1454630 Salmonella enterica subsp. enterica serovar Typhimurium str. USDA-ARS-USMARC-1808

0.00 252 252 S3 1454644 Salmonella enterica subsp. enterica serovar Typhimurium str. CDC 2009K-1640

0.00 234 0 S3 99287 Salmonella enterica subsp. enterica serovar Typhimurium str. LT2

0.00 234 234 S4 588858 Salmonella enterica subsp. enterica serovar Typhimurium str. 14028S

0.00 189 189 S3 1454639 Salmonella enterica subsp. enterica serovar Typhimurium str. USDA-ARS-USMARC-1899

0.00 127 127 S3 1454640 Salmonella enterica subsp. enterica serovar Typhimurium str. CDC H2662

0.00 111 111 S3 568708 Salmonella enterica subsp. enterica serovar Typhimurium str. D23580

0.00 86 86 S3 990282 Salmonella enterica subsp. enterica serovar Typhimurium str. UK-1

0.00 66 66 S3 1454645 Salmonella enterica subsp. enterica serovar Typhimurium str. CDC 2009K-2059

0.00 55 55 S3 1461749 Salmonella enterica subsp. enterica serovar Typhimurium var. monophasic 4,[5],12:i:-

0.00 26 26 S3 2895798 Salmonella enterica subsp. enterica serovar Typhimurium var. monophasic

0.00 14 14 S3 909946 Salmonella enterica subsp. enterica serovar Typhimurium str. ST4/74

0.00 5 5 S3 216597 Salmonella enterica subsp. enterica serovar Typhimurium str. SL1344

0.00 102976 87216 S2 149539 Salmonella enterica subsp. enterica serovar Enteritidis

0.00 915 915 S3 1412501 Salmonella enterica subsp. enterica serovar Enteritidis str. SA19940857

0.00 876 876 S3 1412618 Salmonella enterica subsp. enterica serovar Enteritidis str. EC20120929

0.00 684 684 S3 696867 Salmonella enterica subsp. enterica serovar Enteritidis str. 18569

0.00 636 636 S3 1412470 Salmonella enterica subsp. enterica serovar Enteritidis str. EC20120008

0.00 632 632 S3 1412600 Salmonella enterica subsp. enterica serovar Enteritidis str. EC20100325

0.00 527 527 S3 1244111 Salmonella enterica subsp. enterica serovar Enteritidis str. EC20110357

0.00 465 465 S3 1412580 Salmonella enterica subsp. enterica serovar Enteritidis str. EC20121986

0.00 390 390 S3 1412562 Salmonella enterica subsp. enterica serovar Enteritidis str. EC20121747

0.00 352 352 S3 1412594 Salmonella enterica subsp. enterica serovar Enteritidis str. EC20121177

0.00 339 339 S3 1412495 Salmonella enterica subsp. enterica serovar Enteritidis str. SA20123395

0.00 323 323 S3 1412582 Salmonella enterica subsp. enterica serovar Enteritidis str. EC20121970

0.00 304 304 S3 1244119 Salmonella enterica subsp. enterica serovar Enteritidis str. EC20110361

0.00 290 290 S3 1412481 Salmonella enterica subsp. enterica serovar Enteritidis str. SA20100239

0.00 256 256 S3 1412555 Salmonella enterica subsp. enterica serovar Enteritidis str. EC20121765

0.00 251 251 S3 1412534 Salmonella enterica subsp. enterica serovar Enteritidis str. EC20120685

0.00 245 245 S3 1243621 Salmonella enterica subsp. enterica serovar Enteritidis str. EC20110356

0.00 234 234 S3 1244118 Salmonella enterica subsp. enterica serovar Enteritidis str. EC20110360

0.00 225 225 S3 886715 Salmonella enterica subsp. enterica serovar Enteritidis str. CDC_2010K_0968

0.00 200 200 S3 1412601 Salmonella enterica subsp. enterica serovar Enteritidis str. EC20120051

0.00 196 196 S3 1412457 Salmonella enterica subsp. enterica serovar Enteritidis str. EC20100101

0.00 192 192 S3 926034 Salmonella enterica subsp. enterica serovar Enteritidis str. 77-1427

0.00 159 159 S3 1412454 Salmonella enterica subsp. enterica serovar Enteritidis str. EC20090698

0.00 156 156 S3 1412493 Salmonella enterica subsp. enterica serovar Enteritidis str. SA19942384

0.00 153 153 S3 1412569 Salmonella enterica subsp. enterica serovar Enteritidis str. EC20120994

0.00 149 149 S3 1412527 Salmonella enterica subsp. enterica serovar Enteritidis str. EC20121826

0.00 147 147 S3 1412494 Salmonella enterica subsp. enterica serovar Enteritidis str. SA19943269

0.00 146 146 S3 1412574 Salmonella enterica subsp. enterica serovar Enteritidis str. EC20122033

0.00 142 142 S3 1244121 Salmonella enterica subsp. enterica serovar Enteritidis str. EC20111175

0.00 140 140 S3 1412593 Salmonella enterica subsp. enterica serovar Enteritidis str. EC20121176

0.00 135 135 S3 1412575 Salmonella enterica subsp. enterica serovar Enteritidis str. EC20122031

0.00 133 133 S3 1412453 Salmonella enterica subsp. enterica serovar Enteritidis str. EC20090641

0.00 132 132 S3 1244112 Salmonella enterica subsp. enterica serovar Enteritidis str. EC20110358

0.00 128 128 S3 998822 Salmonella enterica subsp. enterica serovar Enteritidis str. RM2968

0.00 127 127 S3 1243619 Salmonella enterica subsp. enterica serovar Enteritidis str. EC20110354

0.00 127 127 S3 1412472 Salmonella enterica subsp. enterica serovar Enteritidis str. SA19980677

0.00 126 126 S3 1244122 Salmonella enterica subsp. enterica serovar Enteritidis str. EC20111174

0.00 118 118 S3 1412571 Salmonella enterica subsp. enterica serovar Enteritidis str. EC20121812

0.00 109 109 S3 1412477 Salmonella enterica subsp. enterica serovar Enteritidis str. SA20084384

0.00 109 109 S3 1412535 Salmonella enterica subsp. enterica serovar Enteritidis str. EC20111576

0.00 108 108 S3 1243620 Salmonella enterica subsp. enterica serovar Enteritidis str. EC20110355

0.00 107 107 S3 1412465 Salmonella enterica subsp. enterica serovar Enteritidis str. EC20100134

0.00 107 107 S3 1412500 Salmonella enterica subsp. enterica serovar Enteritidis str. SA19930684

0.00 102 102 S3 1412532 Salmonella enterica subsp. enterica serovar Enteritidis str. EC20120776

0.00 100 100 S3 1244113 Salmonella enterica subsp. enterica serovar Enteritidis str. EC20110359

0.00 98 98 S3 1412609 Salmonella enterica subsp. enterica serovar Enteritidis str. EC20120734

0.00 95 95 S3 1412536 Salmonella enterica subsp. enterica serovar Enteritidis str. EC20120544

0.00 94 94 S3 1412499 Salmonella enterica subsp. enterica serovar Enteritidis str. SA19994216

0.00 88 88 S3 1412466 Salmonella enterica subsp. enterica serovar Enteritidis str. EC20120002

0.00 85 85 S3 1412543 Salmonella enterica subsp. enterica serovar Enteritidis str. EC20120548

0.00 80 80 S3 1412588 Salmonella enterica subsp. enterica serovar Enteritidis str. EC20130345

0.00 77 77 S3 1412456 Salmonella enterica subsp. enterica serovar Enteritidis str. EC20110222

0.00 77 77 S3 1412583 Salmonella enterica subsp. enterica serovar Enteritidis str. EC20121969

0.00 76 76 S3 1412611 Salmonella enterica subsp. enterica serovar Enteritidis str. EC20120773

0.00 70 70 S3 1412531 Salmonella enterica subsp. enterica serovar Enteritidis str. EC20120963

0.00 69 69 S3 1412602 Salmonella enterica subsp. enterica serovar Enteritidis str. EC20120356

0.00 66 66 S3 1412557 Salmonella enterica subsp. enterica serovar Enteritidis str. EC20121542

0.00 66 66 S3 1412541 Salmonella enterica subsp. enterica serovar Enteritidis str. EC20121750

0.00 65 65 S3 1412561 Salmonella enterica subsp. enterica serovar Enteritidis str. EC20121672

0.00 65 65 S3 1412537 Salmonella enterica subsp. enterica serovar Enteritidis str. EC20120528

0.00 65 65 S3 1412514 Salmonella enterica subsp. enterica serovar Enteritidis str. SA20093421

0.00 64 64 S3 1412605 Salmonella enterica subsp. enterica serovar Enteritidis str. EC20120590

0.00 62 62 S3 1412455 Salmonella enterica subsp. enterica serovar Enteritidis str. EC20110221

0.00 62 62 S3 1412468 Salmonella enterica subsp. enterica serovar Enteritidis str. EC20120005

0.00 61 61 S3 1412554 Salmonella enterica subsp. enterica serovar Enteritidis str. EC20121744

0.00 61 61 S3 1412516 Salmonella enterica subsp. enterica serovar Enteritidis str. SA20094383

0.00 61 61 S3 1412485 Salmonella enterica subsp. enterica serovar Enteritidis str. SA19981522

0.00 58 58 S3 1412508 Salmonella enterica subsp. enterica serovar Enteritidis str. SA20092320

0.00 56 56 S3 1412590 Salmonella enterica subsp. enterica serovar Enteritidis str. EC20130347

0.00 55 55 S3 1412564 Salmonella enterica subsp. enterica serovar Enteritidis str. EC20120765

0.00 55 55 S3 1412479 Salmonella enterica subsp. enterica serovar Enteritidis str. SA20094177

0.00 54 54 S3 1412619 Salmonella enterica subsp. enterica serovar Enteritidis str. EC20120969

0.00 54 54 S3 1412464 Salmonella enterica subsp. enterica serovar Enteritidis str. EC20100130

0.00 52 52 S3 1412607 Salmonella enterica subsp. enterica serovar Enteritidis str. EC20120686

0.00 52 52 S3 1412525 Salmonella enterica subsp. enterica serovar Enteritidis str. EC20121541

0.00 51 51 S3 1412517 Salmonella enterica subsp. enterica serovar Enteritidis str. SA20090419

0.00 49 49 S3 1412565 Salmonella enterica subsp. enterica serovar Enteritidis str. EC20121671

0.00 49 49 S3 1412576 Salmonella enterica subsp. enterica serovar Enteritidis str. EC20122026

0.00 49 49 S3 1412528 Salmonella enterica subsp. enterica serovar Enteritidis str. EC20120213

0.00 48 48 S3 1412476 Salmonella enterica subsp. enterica serovar Enteritidis str. SA20094682

0.00 48 48 S3 1412471 Salmonella enterica subsp. enterica serovar Enteritidis str. EC20120009

0.00 47 47 S3 1412597 Salmonella enterica subsp. enterica serovar Enteritidis str. EC20121180

0.00 47 47 S3 1412526 Salmonella enterica subsp. enterica serovar Enteritidis str. EC20121825

0.00 46 46 S3 1412560 Salmonella enterica subsp. enterica serovar Enteritidis str. EC20121748

0.00 46 46 S3 1412467 Salmonella enterica subsp. enterica serovar Enteritidis str. EC20120003

0.00 43 43 S3 1412521 Salmonella enterica subsp. enterica serovar Enteritidis str. SA20090877

0.00 41 41 S3 1412460 Salmonella enterica subsp. enterica serovar Enteritidis str. EC20090135

0.00 41 41 S3 1412452 Salmonella enterica subsp. enterica serovar Enteritidis str. SA19992322

0.00 40 40 S3 1412548 Salmonella enterica subsp. enterica serovar Enteritidis str. EC20120219

0.00 39 39 S3 1412486 Salmonella enterica subsp. enterica serovar Enteritidis str. SA19981857

0.00 38 38 S3 1412530 Salmonella enterica subsp. enterica serovar Enteritidis str. EC20121004

0.00 38 38 S3 1412511 Salmonella enterica subsp. enterica serovar Enteritidis str. SA20093788

0.00 37 37 S3 1412546 Salmonella enterica subsp. enterica serovar Enteritidis str. EC20120240

0.00 37 37 S3 1412596 Salmonella enterica subsp. enterica serovar Enteritidis str. EC20121179

0.00 37 37 S3 1412566 Salmonella enterica subsp. enterica serovar Enteritidis str. EC20120555

0.00 37 37 S3 1412587 Salmonella enterica subsp. enterica serovar Enteritidis str. EC20090195

0.00 37 37 S3 1412482 Salmonella enterica subsp. enterica serovar Enteritidis str. SA20100349

0.00 35 35 S3 1412568 Salmonella enterica subsp. enterica serovar Enteritidis str. EC20120497

0.00 34 34 S3 1412502 Salmonella enterica subsp. enterica serovar Enteritidis str. SA19982831

0.00 33 33 S3 1412567 Salmonella enterica subsp. enterica serovar Enteritidis str. EC20111554

0.00 32 32 S3 1412540 Salmonella enterica subsp. enterica serovar Enteritidis str. EC20121751

0.00 32 32 S3 1412507 Salmonella enterica subsp. enterica serovar Enteritidis str. SA20095309

0.00 31 31 S3 1412489 Salmonella enterica subsp. enterica serovar Enteritidis str. SA20085285

0.00 31 31 S3 1147754 Salmonella enterica subsp. enterica serovar Enteritidis str. LA5

0.00 31 31 S3 1412487 Salmonella enterica subsp. enterica serovar Enteritidis str. SA19960848

0.00 29 29 S3 1412591 Salmonella enterica subsp. enterica serovar Enteritidis str. EC20130348

0.00 27 27 S3 1412615 Salmonella enterica subsp. enterica serovar Enteritidis str. EC20120925

0.00 27 27 S3 1412515 Salmonella enterica subsp. enterica serovar Enteritidis str. SA20094389

0.00 27 27 S3 1412556 Salmonella enterica subsp. enterica serovar Enteritidis str. EC20120496

0.00 27 27 S3 1412616 Salmonella enterica subsp. enterica serovar Enteritidis str. EC20121689

0.00 26 26 S3 1412610 Salmonella enterica subsp. enterica serovar Enteritidis str. EC20120738

0.00 26 26 S3 1412552 Salmonella enterica subsp. enterica serovar Enteritidis str. EC20120469

0.00 25 25 S3 1412483 Salmonella enterica subsp. enterica serovar Enteritidis str. SA20121703

0.00 25 25 S3 1412613 Salmonella enterica subsp. enterica serovar Enteritidis str. EC20120917

0.00 24 24 S3 1412488 Salmonella enterica subsp. enterica serovar Enteritidis str. SA20082034

0.00 24 24 S3 1412599 Salmonella enterica subsp. enterica serovar Enteritidis str. EC20100089

0.00 23 23 S3 1412553 Salmonella enterica subsp. enterica serovar Enteritidis str. EC20120697

0.00 23 23 S3 1412518 Salmonella enterica subsp. enterica serovar Enteritidis str. SA20094642

0.00 22 22 S3 1412513 Salmonella enterica subsp. enterica serovar Enteritidis str. SA20090435

0.00 22 22 S3 1412603 Salmonella enterica subsp. enterica serovar Enteritidis str. EC20120580

0.00 22 22 S3 1412544 Salmonella enterica subsp. enterica serovar Enteritidis str. EC20120505

0.00 21 21 S3 1412598 Salmonella enterica subsp. enterica serovar Enteritidis str. EC20100088

0.00 20 20 S3 1412506 Salmonella enterica subsp. enterica serovar Enteritidis str. SA20083456

0.00 20 20 S3 1412533 Salmonella enterica subsp. enterica serovar Enteritidis str. EC20120775

0.00 20 20 S3 1412505 Salmonella enterica subsp. enterica serovar Enteritidis str. SA20094301

0.00 20 20 S3 1412545 Salmonella enterica subsp. enterica serovar Enteritidis str. EC20120498

0.00 20 20 S3 1412551 Salmonella enterica subsp. enterica serovar Enteritidis str. EC20121753

0.00 19 19 S3 1412573 Salmonella enterica subsp. enterica serovar Enteritidis str. EC20122045

0.00 19 19 S3 1412617 Salmonella enterica subsp. enterica serovar Enteritidis str. EC20120927

0.00 18 18 S3 1412578 Salmonella enterica subsp. enterica serovar Enteritidis str. EC20121990

0.00 18 18 S3 1412585 Salmonella enterica subsp. enterica serovar Enteritidis str. EC20090530

0.00 17 17 S3 1412559 Salmonella enterica subsp. enterica serovar Enteritidis str. EC20120677

0.00 17 17 S3 1412520 Salmonella enterica subsp. enterica serovar Enteritidis str. SA20093543

0.00 16 16 S3 1412539 Salmonella enterica subsp. enterica serovar Enteritidis str. EC20111514

0.00 16 16 S3 1412512 Salmonella enterica subsp. enterica serovar Enteritidis str. SA20093430

0.00 16 16 S3 1412519 Salmonella enterica subsp. enterica serovar Enteritidis str. SA20093950

0.00 15 15 S3 1412550 Salmonella enterica subsp. enterica serovar Enteritidis str. EC20120722

0.00 15 15 S3 1412549 Salmonella enterica subsp. enterica serovar Enteritidis str. EC20111510

0.00 14 14 S3 1412589 Salmonella enterica subsp. enterica serovar Enteritidis str. EC20130346

0.00 14 14 S3 1412524 Salmonella enterica subsp. enterica serovar Enteritidis str. SA20094079

0.00 14 14 S3 1412484 Salmonella enterica subsp. enterica serovar Enteritidis str. SA20094521

0.00 14 14 S3 1412558 Salmonella enterica subsp. enterica serovar Enteritidis str. EC20120968

0.00 13 13 S3 1412474 Salmonella enterica subsp. enterica serovar Enteritidis str. SA19970769

0.00 13 13 S3 1412570 Salmonella enterica subsp. enterica serovar Enteritidis str. EC20120916

0.00 13 13 S3 1412542 Salmonella enterica subsp. enterica serovar Enteritidis str. EC20120970

0.00 12 12 S3 1412461 Salmonella enterica subsp. enterica serovar Enteritidis str. EC20090332

0.00 12 12 S3 1412473 Salmonella enterica subsp. enterica serovar Enteritidis str. SA19970510

0.00 11 11 S3 1412579 Salmonella enterica subsp. enterica serovar Enteritidis str. EC20121989

0.00 11 11 S3 1412509 Salmonella enterica subsp. enterica serovar Enteritidis str. SA20093784

0.00 11 11 S3 1412510 Salmonella enterica subsp. enterica serovar Enteritidis str. SA20093977

0.00 11 11 S3 1412497 Salmonella enterica subsp. enterica serovar Enteritidis str. SA19961622

0.00 11 11 S3 1412463 Salmonella enterica subsp. enterica serovar Enteritidis str. EC20090884

0.00 10 10 S3 1412577 Salmonella enterica subsp. enterica serovar Enteritidis str. EC20122022

0.00 10 10 S3 1412451 Salmonella enterica subsp. enterica serovar Enteritidis str. EC20110223

0.00 10 10 S3 1412529 Salmonella enterica subsp. enterica serovar Enteritidis str. EC20120200

0.00 10 10 S3 1412522 Salmonella enterica subsp. enterica serovar Enteritidis str. SA20093538

0.00 9 9 S3 1412492 Salmonella enterica subsp. enterica serovar Enteritidis str. SA20095440

0.00 9 9 S3 1412584 Salmonella enterica subsp. enterica serovar Enteritidis str. EC20100131

0.00 9 9 S3 1412547 Salmonella enterica subsp. enterica serovar Enteritidis str. EC20120229

0.00 8 8 S3 1412612 Salmonella enterica subsp. enterica serovar Enteritidis str. EC20120774

0.00 8 8 S3 1412480 Salmonella enterica subsp. enterica serovar Enteritidis str. SA20084644

0.00 7 7 S3 550537 Salmonella enterica subsp. enterica serovar Enteritidis str. P125109

0.00 7 7 S3 1412459 Salmonella enterica subsp. enterica serovar Enteritidis str. EC20090193

0.00 7 7 S3 1412475 Salmonella enterica subsp. enterica serovar Enteritidis str. SA20093266

0.00 6 6 S3 1412595 Salmonella enterica subsp. enterica serovar Enteritidis str. EC20121178

0.00 6 6 S3 1412614 Salmonella enterica subsp. enterica serovar Enteritidis str. EC20120918

0.00 4 4 S3 1412478 Salmonella enterica subsp. enterica serovar Enteritidis str. SA20084824

0.00 4 4 S3 1412504 Salmonella enterica subsp. enterica serovar Enteritidis str. SA20094350

0.00 4 4 S3 1412469 Salmonella enterica subsp. enterica serovar Enteritidis str. EC20120007

0.00 3 3 S3 1412538 Salmonella enterica subsp. enterica serovar Enteritidis str. EC20111515

0.00 2 2 S3 1412503 Salmonella enterica subsp. enterica serovar Enteritidis str. SA20094803

0.00 2 2 S3 1412498 Salmonella enterica subsp. enterica serovar Enteritidis str. SA19983126

0.00 2 2 S3 1412604 Salmonella enterica subsp. enterica serovar Enteritidis str. EC20120581

0.00 1 1 S3 1412490 Salmonella enterica subsp. enterica serovar Enteritidis str. SA20083636

0.00 1 1 S3 1412586 Salmonella enterica subsp. enterica serovar Enteritidis str. EC20100100

0.00 1 1 S3 1412462 Salmonella enterica subsp. enterica serovar Enteritidis str. EC20090531

0.00 1 1 S3 1412608 Salmonella enterica subsp. enterica serovar Enteritidis str. EC20120687

0.00 95075 18933 S2 192954 Salmonella enterica subsp. enterica serovar Mbandaka

0.00 76142 76142 S3 984237 Salmonella enterica subsp. enterica serovar Mbandaka str. ATCC 51958

0.00 94282 93255 S2 57743 Salmonella enterica subsp. enterica serovar Weltevreden

0.00 993 993 S3 1173939 Salmonella enterica subsp. enterica serovar Weltevreden str. 1655

0.00 34 34 S3 936157 Salmonella enterica subsp. enterica serovar Weltevreden str. 2007-60-3289-1

0.00 93259 38989 S2 108619 Salmonella enterica subsp. enterica serovar Newport

0.00 20342 20342 S3 1454627 Salmonella enterica subsp. enterica serovar Newport str. CDC 2010K-2159

0.00 6865 6865 S3 930779 Salmonella enterica subsp. enterica serovar Newport str. Levine 15

0.00 5994 5994 S3 930778 Salmonella enterica subsp. enterica serovar Newport str. Levine 1

0.00 4804 4804 S3 1454614 Salmonella enterica subsp. enterica serovar Newport str. CDC 2012K-0938

0.00 3869 3869 S3 1454625 Salmonella enterica subsp. enterica serovar Newport str. CDC 2009K-1331

0.00 3507 3507 S3 1454620 Salmonella enterica subsp. enterica serovar Newport str. USDA-ARS-USMARC-1927

0.00 1918 1918 S3 997339 Salmonella enterica subsp. enterica serovar Newport str. WA_14882

0.00 816 816 S3 1299166 Salmonella enterica subsp. enterica serovar Newport str. CFSAN000827

0.00 722 722 S3 796731 Salmonella enterica subsp. enterica serovar Newport str. CVM 21538

0.00 652 652 S3 858306 Salmonella enterica subsp. enterica serovar Newport str. CVM N18486

0.00 586 586 S3 796730 Salmonella enterica subsp. enterica serovar Newport str. CVM 22425

0.00 488 488 S3 858305 Salmonella enterica subsp. enterica serovar Newport str. CVM 22462

0.00 408 408 S3 1454621 Salmonella enterica subsp. enterica serovar Newport str. USDA-ARS-USMARC-1928

0.00 405 405 S3 877468 Salmonella enterica subsp. enterica serovar Newport str. USMARC-S3124.1

0.00 400 400 S3 796732 Salmonella enterica subsp. enterica serovar Newport str. CVM 22513

0.00 377 377 S3 1454616 Salmonella enterica subsp. enterica serovar Newport str. USDA-ARS-USMARC-1923

0.00 345 345 S3 1454629 Salmonella enterica subsp. enterica serovar Newport str. CDC 2012K-0663

0.00 315 315 S3 1454618 Salmonella enterica subsp. enterica serovar Newport str. USDA-ARS-USMARC-1925

0.00 270 270 S3 1454622 Salmonella enterica subsp. enterica serovar Newport str. USDA-ARS-USMARC-1929

0.00 268 268 S3 1454617 Salmonella enterica subsp. enterica serovar Newport str. USDA-ARS-USMARC-1924

0.00 259 259 S3 858307 Salmonella enterica subsp. enterica serovar Newport str. CVM N1543

0.00 259 259 S3 796733 Salmonella enterica subsp. enterica serovar Newport str. CVM 21550

0.00 202 202 S3 1454619 Salmonella enterica subsp. enterica serovar Newport str. USDA-ARS-USMARC-1926

0.00 199 199 S3 423368 Salmonella enterica subsp. enterica serovar Newport str. SL254

0.00 76387 38780 S2 58712 Salmonella enterica subsp. enterica serovar Anatum

0.00 20987 20987 S3 1454585 Salmonella enterica subsp. enterica serovar Anatum str. USDA-ARS-USMARC-1735

0.00 3510 3510 S3 1454589 Salmonella enterica subsp. enterica serovar Anatum str. USDA-ARS-USMARC-1728

0.00 2817 2817 S3 1454593 Salmonella enterica subsp. enterica serovar Anatum str. USDA-ARS-USMARC-1765

0.00 1943 1943 S3 1454594 Salmonella enterica subsp. enterica serovar Anatum str. USDA-ARS-USMARC-1766

0.00 1680 1680 S3 1454584 Salmonella enterica subsp. enterica serovar Anatum str. USDA-ARS-USMARC-1727

0.00 1537 1537 S3 1399029 Salmonella enterica subsp. enterica serovar Anatum str. CFSAN003961

0.00 944 944 S3 984211 Salmonella enterica subsp. enterica serovar Anatum str. ATCC BAA-1592

0.00 800 800 S3 1454596 Salmonella enterica subsp. enterica serovar Anatum str. USDA-ARS-USMARC-1781

0.00 745 745 S3 1454590 Salmonella enterica subsp. enterica serovar Anatum str. USDA-ARS-USMARC-1175

0.00 738 738 S3 1454588 Salmonella enterica subsp. enterica serovar Anatum str. USDA-ARS-USMARC-1677

0.00 679 679 S3 1454583 Salmonella enterica subsp. enterica serovar Anatum str. USDA-ARS-USMARC-1783

0.00 618 618 S3 1454592 Salmonella enterica subsp. enterica serovar Anatum str. CDC 06-0532

0.00 408 408 S3 1454587 Salmonella enterica subsp. enterica serovar Anatum str. USDA-ARS-USMARC-1676

0.00 201 201 S3 1454586 Salmonella enterica subsp. enterica serovar Anatum str. USDA-ARS-USMARC-1736

0.00 71600 67110 S2 90370 Salmonella enterica subsp. enterica serovar Typhi

0.00 1535 1535 S3 220341 Salmonella enterica subsp. enterica serovar Typhi str. CT18

0.00 1132 1132 S3 497977 Salmonella enterica subsp. enterica serovar Typhi str. 404ty

0.00 1021 1021 S3 209261 Salmonella enterica subsp. enterica serovar Typhi str. Ty2

0.00 461 461 S3 527001 Salmonella enterica subsp. enterica serovar Typhi str. Ty21a

0.00 341 341 S3 1132507 Salmonella enterica subsp. enterica serovar Typhi str. P-stx-12

0.00 65032 65032 S2 2926665 Salmonella enterica subsp. enterica serovar Abeokuta

0.00 61962 59072 S2 70803 Salmonella enterica subsp. enterica serovar Minnesota

0.00 2890 2890 S3 1124956 Salmonella enterica subsp. enterica serovar Minnesota str. ATCC 49284

0.00 60256 40701 S2 913070 Salmonella enterica subsp. enterica serovar Gaminara

0.00 19555 19555 S3 1242094 Salmonella enterica subsp. enterica serovar Gaminara str. SA20063285

0.00 59626 59626 S2 1151001 Salmonella enterica subsp. enterica serovar Napoli

0.00 59497 59497 S2 2500153 Salmonella enterica subsp. enterica serovar Karamoja

0.00 58335 30862 S2 90105 Salmonella enterica subsp. enterica serovar Saintpaul

0.00 27473 27473 S3 702982 Salmonella enterica subsp. enterica serovar Saintpaul str. SARA26

0.00 57715 45356 S2 595 Salmonella enterica subsp. enterica serovar Infantis

0.00 12359 12359 S3 596155 Salmonella enterica subsp. enterica serovar Infantis str. SARB27

0.00 53922 53449 S2 28150 Salmonella enterica subsp. enterica serovar Senftenberg

0.00 473 473 S3 749965 Salmonella enterica subsp. enterica serovar Senftenberg str. 361154004

0.00 51897 0 S2 1242080 Salmonella enterica subsp. enterica serovar Djakarta

0.00 51897 51897 S3 1242091 Salmonella enterica subsp. enterica serovar Djakarta str. S-1087

0.00 49529 47110 S2 211968 Salmonella enterica subsp. enterica serovar Albany

0.00 2419 2419 S3 1173798 Salmonella enterica subsp. enterica serovar Albany str. ATCC 51960

0.00 48684 0 S2 436295 Salmonella enterica subsp. enterica serovar Poona

0.00 48684 48684 S3 1124962 Salmonella enterica subsp. enterica serovar Poona str. ATCC BAA-1673

0.00 48273 0 S2 1243577 Salmonella enterica subsp. enterica serovar Milwaukee

0.00 48273 48273 S3 1243578 Salmonella enterica subsp. enterica serovar Milwaukee str. SA19950795

0.00 48094 3100 S2 598 Salmonella enterica subsp. enterica serovar Rubislaw

0.00 44994 44994 S3 938143 Salmonella enterica subsp. enterica serovar Rubislaw str. ATCC 10717

0.00 46220 27527 S2 115981 Salmonella enterica subsp. enterica serovar Montevideo

0.00 6524 6524 S3 1454607 Salmonella enterica subsp. enterica serovar Montevideo str. CDC 07-0954

0.00 2420 2420 S3 1454610 Salmonella enterica subsp. enterica serovar Montevideo str. CDC 2011K-1674

0.00 1698 1698 S3 763921 Salmonella enterica subsp. enterica serovar Montevideo str. 42N

0.00 1271 1271 S3 1454609 Salmonella enterica subsp. enterica serovar Montevideo str. CDC 2009K-0792

0.00 1248 1248 S3 1454604 Salmonella enterica subsp. enterica serovar Montevideo str. USDA-ARS-USMARC-1904

0.00 937 937 S3 1454603 Salmonella enterica subsp. enterica serovar Montevideo str. USDA-ARS-USMARC-1903

0.00 607 607 S3 1454612 Salmonella enterica subsp. enterica serovar Montevideo str. CDC 2013K-0218

0.00 506 506 S3 1454600 Salmonella enterica subsp. enterica serovar Montevideo str. USDA-ARS-USMARC-1912

0.00 490 490 S3 1454598 Salmonella enterica subsp. enterica serovar Montevideo str. USDA-ARS-USMARC-1901

0.00 469 469 S3 1454599 Salmonella enterica subsp. enterica serovar Montevideo str. USDA-ARS-USMARC-1900

0.00 465 465 S3 1454601 Salmonella enterica subsp. enterica serovar Montevideo str. USDA-ARS-USMARC-1913

0.00 458 458 S3 1454611 Salmonella enterica subsp. enterica serovar Montevideo str. CDC 2012K-1544

0.00 446 446 S3 1454613 Salmonella enterica subsp. enterica serovar Montevideo str. CDC 2010K-0257

0.00 414 414 S3 1454608 Salmonella enterica subsp. enterica serovar Montevideo str. CDC 08-1942

0.00 283 283 S3 859199 Salmonella enterica subsp. enterica serovar Montevideo str. 507440-20

0.00 239 239 S3 1454606 Salmonella enterica subsp. enterica serovar Montevideo str. CDC 86-0391

0.00 218 218 S3 745022 Salmonella enterica subsp. enterica serovar Montevideo str. 531954

0.00 44964 44953 S2 28144 Salmonella enterica subsp. enterica serovar Derby

0.00 11 11 S3 1401650 Salmonella enterica subsp. enterica serovar Derby str. T12

0.00 43213 10655 S2 486994 Salmonella enterica subsp. enterica serovar Hvittingfoss

0.00 32558 32558 S3 1242097 Salmonella enterica subsp. enterica serovar Hvittingfoss str. SA20014981

0.00 37467 31679 S2 58096 Salmonella enterica subsp. enterica serovar Bareilly

0.00 821 821 S3 1182174 Salmonella enterica subsp. enterica serovar Bareilly str. CFSAN000669

0.00 757 757 S3 1173427 Salmonella enterica subsp. enterica serovar Bareilly str. CFSAN000189

0.00 673 673 S3 1182177 Salmonella enterica subsp. enterica serovar Bareilly str. CFSAN000752

0.00 557 557 S3 1183391 Salmonella enterica subsp. enterica serovar Bareilly str. CFSAN000968

0.00 350 350 S3 1182172 Salmonella enterica subsp. enterica serovar Bareilly str. CFSAN000661

0.00 253 253 S3 1208622 Salmonella enterica subsp. enterica serovar Bareilly str. CFSAN001118

0.00 225 225 S3 1173456 Salmonella enterica subsp. enterica serovar Bareilly str. CFSAN000228

0.00 199 199 S3 1208629 Salmonella enterica subsp. enterica serovar Bareilly str. CFSAN001112

0.00 195 195 S3 1173457 Salmonella enterica subsp. enterica serovar Bareilly str. CFSAN000191

0.00 195 195 S3 1182191 Salmonella enterica subsp. enterica serovar Bareilly str. CFSAN000961

0.00 186 186 S3 1182188 Salmonella enterica subsp. enterica serovar Bareilly str. CFSAN000958

0.00 168 168 S3 1182181 Salmonella enterica subsp. enterica serovar Bareilly str. CFSAN000952

0.00 167 167 S3 1208630 Salmonella enterica subsp. enterica serovar Bareilly str. CFSAN001115

0.00 154 154 S3 1182183 Salmonella enterica subsp. enterica serovar Bareilly str. CFSAN000954

0.00 140 140 S3 1182190 Salmonella enterica subsp. enterica serovar Bareilly str. CFSAN000960

0.00 128 128 S3 1182176 Salmonella enterica subsp. enterica serovar Bareilly str. CFSAN000700

0.00 122 122 S3 1173459 Salmonella enterica subsp. enterica serovar Bareilly str. CFSAN000211

0.00 108 108 S3 1183393 Salmonella enterica subsp. enterica serovar Bareilly str. CFSAN000970

0.00 91 91 S3 1173461 Salmonella enterica subsp. enterica serovar Bareilly str. CFSAN000212

0.00 85 85 S3 1208623 Salmonella enterica subsp. enterica serovar Bareilly str. CFSAN001140

0.00 82 82 S3 1182180 Salmonella enterica subsp. enterica serovar Bareilly str. CFSAN000951

0.00 73 73 S3 1182193 Salmonella enterica subsp. enterica serovar Bareilly str. CFSAN000963

0.00 59 59 S3 1182178 Salmonella enterica subsp. enterica serovar Bareilly str. CFSAN000753

0.00 37037 0 S2 181798 Salmonella enterica subsp. enterica serovar Sloterdijk

0.00 37037 37037 S3 938141 Salmonella enterica subsp. enterica serovar Sloterdijk str. ATCC 15791

0.00 36208 36208 S2 58101 Salmonella enterica subsp. enterica serovar Waycross

0.00 35293 5342 S2 57045 Salmonella enterica subsp. enterica serovar Paratyphi B

0.00 24535 24535 S3 224729 Salmonella enterica subsp. enterica serovar Java

0.00 5416 5416 S3 1016998 Salmonella enterica subsp. enterica serovar Paratyphi B str. SPB7

0.00 35045 35045 S2 596 Salmonella enterica subsp. enterica serovar Muenchen

0.00 34359 34359 S2 1962641 Salmonella enterica subsp. enterica serovar Birkenhead

0.00 33525 0 S2 1242079 Salmonella enterica subsp. enterica serovar Apapa

0.00 33525 33525 S3 1242088 Salmonella enterica subsp. enterica serovar Apapa str. SA20060561

0.00 31949 29885 S2 340188 Salmonella enterica subsp. enterica serovar Cerro

0.00 2064 2064 S3 1410916 Salmonella enterica subsp. enterica serovar Cerro str. CFSAN001588

0.00 31340 0 S2 1243583 Salmonella enterica subsp. enterica serovar Onderstepoort

0.00 31340 31340 S3 1243584 Salmonella enterica subsp. enterica serovar Onderstepoort str. SA20060086

0.00 31296 29885 S2 192955 Salmonella enterica subsp. enterica serovar Kentucky

0.00 1373 1373 S3 1242102 Salmonella enterica subsp. enterica serovar Kentucky str. SA20030505

0.00 38 38 S3 439842 Salmonella enterica subsp. enterica serovar Kentucky str. CVM29188

0.00 29213 0 S2 1242084 Salmonella enterica subsp. enterica serovar Krefeld

0.00 29213 29213 S3 1242106 Salmonella enterica subsp. enterica serovar Krefeld str. SA20030536

0.00 29049 29049 S2 1160769 Salmonella enterica subsp. enterica serovar Worthington

0.00 28303 25694 S2 28147 Salmonella enterica subsp. enterica serovar Oranienburg

0.00 2609 2609 S3 997333 Salmonella enterica subsp. enterica serovar Oranienburg str. 0250

0.00 28068 28068 S2 2564349 Salmonella enterica subsp. enterica serovar Dessau

0.00 27291 17829 S2 189201 Salmonella enterica subsp. enterica serovar Cubana

0.00 9462 9462 S3 1271863 Salmonella enterica subsp. enterica serovar Cubana str. CFSAN002050

0.00 26212 0 S2 913085 Salmonella enterica subsp. enterica serovar Wandsworth

0.00 26212 26212 S3 1243595 Salmonella enterica subsp. enterica serovar Wandsworth str. SA20092095

0.00 26034 23296 S2 594 Salmonella enterica subsp. enterica serovar Gallinarum

0.00 1340 1340 S3 550538 Salmonella enterica subsp. enterica serovar Gallinarum str. 287/91

0.00 740 740 S3 909947 Salmonella enterica subsp. enterica serovar Gallinarum str. SG9

0.00 658 658 S3 685040 Salmonella enterica subsp. enterica serovar Gallinarum str. 9184

0.00 24370 24370 S2 46626 Salmonella enterica subsp. enterica serovar Give

0.00 23986 0 S2 260368 Salmonella enterica subsp. enterica serovar Bergen

0.00 23986 23986 S3 1240708 Salmonella enterica subsp. enterica serovar Bergen str. ST350

0.00 23346 23346 S2 149388 Salmonella enterica subsp. enterica serovar Mikawasima

0.00 21877 0 S2 1242081 Salmonella enterica subsp. enterica serovar Hillingdon

0.00 21877 21877 S3 1242096 Salmonella enterica subsp. enterica serovar Hillingdon str. N1529-D3

0.00 21102 21102 S2 2564310 Salmonella enterica subsp. enterica serovar Carmel

0.00 20792 0 S2 915158 Salmonella enterica subsp. enterica serovar Manchester

0.00 20792 20792 S3 1242108 Salmonella enterica subsp. enterica serovar Manchester str. ST278

0.00 20678 20678 S2 593905 Salmonella enterica subsp. enterica serovar Corvallis

0.00 20624 20624 S2 2021403 Salmonella enterica subsp. enterica serovar Adjame

0.00 19804 19804 S2 2583588 Salmonella enterica subsp. enterica serovar 1,4,[5],12:i:-

0.00 19794 19794 S2 1077085 Salmonella enterica subsp. enterica serovar Fresno

0.00 19554 19554 S2 57741 Salmonella enterica subsp. enterica serovar Blockley

0.00 18843 13435 S2 611 Salmonella enterica subsp. enterica serovar Heidelberg

0.00 2971 2971 S3 1160717 Salmonella enterica subsp. enterica serovar Heidelberg str. B182

0.00 725 725 S3 454169 Salmonella enterica subsp. enterica serovar Heidelberg str. SL476

0.00 635 635 S3 1124936 Salmonella enterica subsp. enterica serovar Heidelberg str. 41578

0.00 465 465 S3 1271864 Salmonella enterica subsp. enterica serovar Heidelberg str. CFSAN002069

0.00 223 223 S3 1030005 Salmonella enterica subsp. enterica serovar Heidelberg str. N418

0.00 209 209 S3 1299044 Salmonella enterica subsp. enterica serovar Heidelberg str. CFSAN002064

0.00 180 180 S3 1029997 Salmonella enterica subsp. enterica serovar Heidelberg str. SARA35

0.00 17859 17859 S2 2024273 Salmonella enterica subsp. enterica serovar Sundsvall

0.00 17639 10991 S2 58095 Salmonella enterica subsp. enterica serovar Agona

0.00 6042 6042 S3 454166 Salmonella enterica subsp. enterica serovar Agona str. SL483

0.00 242 242 S3 1406860 Salmonella enterica subsp. enterica serovar Agona str. 24249

0.00 199 199 S3 1124917 Salmonella enterica subsp. enterica serovar Agona str. 460004 2-1

0.00 165 165 S3 1072590 Salmonella enterica subsp. enterica serovar Agona str. 392869-2

0.00 17612 17612 S2 286783 Salmonella enterica subsp. enterica serovar Indiana

0.00 17436 17436 S2 149385 Salmonella enterica subsp. enterica serovar Hadar

0.00 17368 13737 S2 54388 Salmonella enterica subsp. enterica serovar Paratyphi A

0.00 2208 2208 S3 941189 Salmonella enterica subsp. enterica serovar Paratyphi A str. ATCC 11511

0.00 960 960 S3 554290 Salmonella enterica subsp. enterica serovar Paratyphi A str. AKU_12601

0.00 463 463 S3 295319 Salmonella enterica subsp. enterica serovar Paratyphi A str. ATCC 9150

0.00 16427 0 S2 913076 Salmonella enterica subsp. enterica serovar Johannesburg

0.00 16427 16427 S3 1242101 Salmonella enterica subsp. enterica serovar Johannesburg str. ST203

0.00 16033 1 S2 363569 Salmonella enterica subsp. enterica serovar Javiana

0.00 16032 16032 S3 1267753 Salmonella enterica subsp. enterica serovar Javiana str. CFSAN001992

0.00 14767 12025 S2 98360 Salmonella enterica subsp. enterica serovar Dublin

0.00 1140 1140 S3 687860 Salmonella enterica subsp. enterica serovar Dublin str. ATCC 39184

0.00 887 887 S3 909945 Salmonella enterica subsp. enterica serovar Dublin str. SD3246

0.00 715 715 S3 439851 Salmonella enterica subsp. enterica serovar Dublin str. CT_02021853

0.00 14626 13214 S2 570935 Salmonella enterica subsp. enterica serovar Pomona

0.00 1412 1412 S3 941188 Salmonella enterica subsp. enterica serovar Pomona str. ATCC 10729

0.00 14231 0 S2 1243590 Salmonella enterica subsp. enterica serovar Quebec

0.00 14231 14231 S3 1243591 Salmonella enterica subsp. enterica serovar Quebec str. S-1267

0.00 13684 8535 S2 58097 Salmonella enterica subsp. enterica serovar Bovismorbificans

0.00 5149 5149 S3 1320309 Salmonella enterica subsp. enterica serovar Bovismorbificans str. 3114

0.00 13544 13544 S2 2511819 Salmonella enterica subsp. enterica serovar Brancaster

0.00 12672 7176 S2 286782 Salmonella enterica subsp. enterica serovar Stanleyville

0.00 5496 5496 S3 1194159 Salmonella enterica subsp. enterica serovar Stanleyville str. CFSAN000624

0.00 12189 5228 S2 82689 Salmonella enterica subsp. enterica serovar Muenster

0.00 6490 6490 S3 1124958 Salmonella enterica subsp. enterica serovar Muenster str. 0315

0.00 471 471 S3 1124959 Salmonella enterica subsp. enterica serovar Muenster str. 420

0.00 11850 11850 S2 28142 Salmonella enterica subsp. enterica serovar Berta

0.00 11690 0 S2 1242078 Salmonella enterica subsp. enterica serovar Antsalova

0.00 11690 11690 S3 1242087 Salmonella enterica subsp. enterica serovar Antsalova str. S01-0511

0.00 11386 10561 S2 143221 Salmonella enterica subsp. enterica serovar Tennessee

0.00 825 825 S3 1003191 Salmonella enterica subsp. enterica serovar Tennessee str. TXSC_TXSC08-19

0.00 11159 10939 S2 149391 Salmonella enterica subsp. enterica serovar Braenderup

0.00 220 220 S3 930771 Salmonella enterica subsp. enterica serovar Braenderup str. ATCC BAA-664

0.00 10583 0 S2 29482 Salmonella enterica subsp. enterica serovar Abony

0.00 10583 10583 S3 1029983 Salmonella enterica subsp. enterica serovar Abony str. 0014

0.00 9278 9278 S2 192953 Salmonella enterica subsp. enterica serovar Stanley

0.00 9158 7867 S2 605 Salmonella enterica subsp. enterica serovar Pullorum

0.00 1291 1291 S3 1029979 Salmonella enterica subsp. enterica serovar Pullorum str. ATCC 9120

0.00 9138 9138 S2 149387 Salmonella enterica subsp. enterica serovar Brandenburg

0.00 8952 8509 S2 340190 Salmonella enterica subsp. enterica serovar Schwarzengrund

0.00 443 443 S3 439843 Salmonella enterica subsp. enterica serovar Schwarzengrund str. CVM19633

0.00 8519 6682 S2 913074 Salmonella enterica subsp. enterica serovar Inverness

0.00 1837 1837 S3 941187 Salmonella enterica subsp. enterica serovar Inverness str. ATCC 10720

0.00 8164 7854 S2 440524 Salmonella enterica subsp. enterica serovar 4,[5],12:i:-

0.00 310 310 S3 866913 Salmonella enterica subsp. enterica serovar 4,[5],12:i:- str. 08-1736

0.00 8149 5796 S2 600 Salmonella enterica subsp. enterica serovar Thompson

0.00 2070 2070 S3 935705 Salmonella enterica subsp. enterica serovar Thompson str. ATCC 8391

0.00 283 283 S3 1064551 Salmonella enterica subsp. enterica serovar Thompson str. RM6836

0.00 6645 6645 S2 149390 Salmonella enterica subsp. enterica serovar London

0.00 6357 6357 S2 224727 Salmonella enterica subsp. enterica serovar Kottbus

0.00 6090 5107 S2 1243585 Salmonella enterica subsp. enterica serovar Ouakam

0.00 983 983 S3 1243586 Salmonella enterica subsp. enterica serovar Ouakam str. SA20034636

0.00 5822 1862 S2 55400 Salmonella enterica subsp. enterica serovar Borreze

0.00 3960 3960 S3 1242089 Salmonella enterica subsp. enterica serovar Borreze str. SA20041063

0.00 5185 5185 S2 117541 Salmonella enterica subsp. enterica serovar Ohio

0.00 5087 5087 S2 1129117 Salmonella enterica subsp. enterica serovar Nitra

0.00 4936 0 S2 28146 Salmonella enterica subsp. enterica serovar Moscow

0.00 4936 4936 S3 1243579 Salmonella enterica subsp. enterica serovar Moscow str. S-1843

0.00 4748 0 S2 149386 Salmonella enterica subsp. enterica serovar Chester

0.00 4748 4748 S3 941190 Salmonella enterica subsp. enterica serovar Chester str. ATCC 11997

0.00 4670 4670 S2 340189 Salmonella enterica subsp. enterica serovar Manhattan

0.00 4400 4400 S2 486993 Salmonella enterica subsp. enterica serovar Eastbourne

0.00 4173 0 S2 57046 Salmonella enterica subsp. enterica serovar Paratyphi C

0.00 4173 4173 S3 476213 Salmonella enterica subsp. enterica serovar Paratyphi C str. RKS4594

0.00 4155 0 S2 1151172 Salmonella enterica subsp. enterica serovar Abaetetuba

0.00 4155 4155 S3 1208611 Salmonella enterica subsp. enterica serovar Abaetetuba str. ATCC 35640

0.00 3755 3673 S2 48409 Salmonella enterica subsp. enterica serovar Virchow

0.00 82 82 S3 465517 Salmonella enterica subsp. enterica serovar Virchow str. SL491

0.00 3330 0 S2 134047 Salmonella enterica subsp. enterica serovar Bredeney

0.00 3330 3330 S3 1194154 Salmonella enterica subsp. enterica serovar Bredeney str. CFSAN001080

0.00 2066 0 S2 1243599 Salmonella enterica subsp. enterica serovar Yovokome

0.00 2066 2066 S3 1243600 Salmonella enterica subsp. enterica serovar Yovokome str. S-1850

0.00 1964 1964 S2 487004 Salmonella enterica subsp. enterica serovar Uganda

0.00 1929 1929 S2 260678 Salmonella enterica subsp. enterica serovar Goldcoast

0.00 1830 1830 S2 2564534 Salmonella enterica subsp. enterica serovar Hissar

0.00 1746 1746 S2 165302 Salmonella enterica subsp. enterica serovar Reading

0.00 1743 1743 S2 2579247 Salmonella enterica subsp. enterica serovar Rough O:-:-

0.00 1742 1742 S2 356621 Salmonella enterica subsp. enterica serovar Westhampton

0.00 1692 0 S2 604 Salmonella enterica subsp. enterica serovar Gallinarum/Pullorum

0.00 1692 1692 S3 1225522 Salmonella enterica subsp. enterica serovar Gallinarum/Pullorum str. CDC1983-67

0.00 1689 1689 S2 358771 Salmonella enterica subsp. enterica serovar Kedougou

0.00 1652 0 S2 1242083 Salmonella enterica subsp. enterica serovar Koessen

0.00 1652 1652 S3 1242105 Salmonella enterica subsp. enterica serovar Koessen str. S-1501

0.00 1565 1565 S2 486999 Salmonella enterica subsp. enterica serovar Meleagridis

0.00 1542 0 S2 1160741 Salmonella enterica subsp. enterica serovar Crossness

0.00 1542 1542 S3 1242090 Salmonella enterica subsp. enterica serovar Crossness str. 1422-74

0.00 1512 1512 S2 29474 Salmonella enterica subsp. enterica serovar California

0.00 1286 0 S2 29472 Salmonella enterica subsp. enterica serovar Panama

0.00 1286 1286 S3 1124961 Salmonella enterica subsp. enterica serovar Panama str. ATCC 7378

0.00 709 709 S2 1967643 Salmonella enterica subsp. enterica serovar Albert

0.00 701 701 S2 2077273 Salmonella enterica subsp. enterica serovar Lubbock

0.00 661 0 S2 399583 Salmonella enterica subsp. enterica serovar Blegdam

0.00 661 661 S3 1240694 Salmonella enterica subsp. enterica serovar Blegdam str. S-1824

0.00 588 588 S2 932733 Salmonella enterica subsp. enterica serovar Bardo

0.00 554 554 S2 1911681 Salmonella enterica subsp. enterica serovar Hayindogo

0.00 435 435 S2 399587 Salmonella enterica subsp. enterica serovar Rissen

0.00 324 324 S2 367181 Salmonella enterica subsp. enterica serovar Newlands

0.00 112 112 S2 1160757 Salmonella enterica subsp. enterica serovar Lomita

0.00 56 56 S2 179997 Salmonella enterica subsp. enterica serovar Havana

0.00 42 42 S2 149384 Salmonella enterica subsp. enterica serovar Wien

0.00 40 40 S2 1967595 Salmonella enterica subsp. enterica serovar 6,7:c:1,5

0.00 17 17 S2 353569 Salmonella enterica subsp. enterica serovar 4,12:i:-

0.00 1 1 S2 794865 Salmonella enterica subsp. enterica serovar 6,7:d:-

0.00 1163583 286628 S1 59202 Salmonella enterica subsp. salamae

0.00 504908 0 S2 1243601 Salmonella enterica subsp. salamae serovar 55:k:z39

0.00 504908 504908 S3 1243602 Salmonella enterica subsp. salamae serovar 55:k:z39 str. 1315K

0.00 128782 128782 S2 1710356 Salmonella enterica subsp. salamae serovar 57:z29:z42

0.00 115739 115739 S2 2500152 Salmonella enterica subsp. salamae serovar 42:r:-

0.00 76044 76044 S2 2577858 Salmonella enterica subsp. salamae serovar 56:b:[1,5]

0.00 51482 51482 S2 2577863 Salmonella enterica subsp. salamae serovar 56:z10:e,n,x

0.00 368279 100467 S1 59204 Salmonella enterica subsp. diarizonae

0.00 122113 122113 S2 1192842 Salmonella enterica subsp. diarizonae serovar 48:i:z

0.00 65020 0 S2 1243615 Salmonella enterica subsp. diarizonae serovar 65:c:z

0.00 65020 65020 S3 1243616 Salmonella enterica subsp. diarizonae serovar 65:c:z str. SA20044251

0.00 43417 43417 S2 2339227 Salmonella enterica subsp. diarizonae serovar 61:k:1,5,(7)

0.00 21546 21546 S2 1173779 Salmonella enterica subsp. diarizonae serovar 60:r:e,n,x,z15

0.00 15716 0 S2 1243611 Salmonella enterica subsp. diarizonae serovar 50:k:z

0.00 15716 15716 S3 1243612 Salmonella enterica subsp. diarizonae serovar 50:k:z str. MZ0080

0.00 264165 0 S1 59208 Salmonella enterica subsp. VII

0.00 264165 264165 S2 41520 Salmonella enterica subsp. VII serovar 1,40:g,z51:--

0.00 179427 100245 S1 59203 Salmonella enterica subsp. arizonae

0.00 39319 39319 S2 41514 Salmonella enterica subsp. arizonae serovar 62:z4,z23:-

0.00 37187 37187 S2 1243607 Salmonella enterica subsp. arizonae serovar 63:g,z51:-

0.00 2676 0 S2 41515 Salmonella enterica subsp. arizonae serovar 62:z36:-

0.00 2676 2676 S3 1386967 Salmonella enterica subsp. arizonae serovar 62:z36:- str. RKS2983

0.00 147502 10812 S1 59205 Salmonella enterica subsp. houtenae

0.00 63416 63416 S2 2729104 Salmonella enterica subsp. houtenae serovar 43:z4

0.00 39323 39323 S2 1299291 Salmonella enterica subsp. houtenae str. CFSAN000552

0.00 31952 31952 S2 523831 Salmonella enterica subsp. houtenae str. ATCC BAA-1581

0.00 1999 1999 S2 1967611 Salmonella enterica subsp. houtenae serovar 45:g,z51:-

0.00 8060 8060 S1 59207 Salmonella enterica subsp. indica

0.01 1551956 1374847 S 54736 Salmonella bongori

0.00 69152 0 S1 41527 Salmonella bongori serovar 48:z41:--

0.00 69152 69152 S2 1382510 Salmonella bongori serovar 48:z41:-- str. RKS3044

0.00 53595 53595 S1 1173775 Salmonella bongori serovar 40:z35:-

0.00 35873 35873 S1 1197719 Salmonella bongori N268-08

0.00 12167 12167 S1 2577118 Salmonella bongori serovar 48:z81:-

0.00 3124 3124 S1 1299282 Salmonella bongori CFSAN000510

0.00 1606 1606 S1 218493 Salmonella bongori NCTC 12419

0.00 1592 0 S1 59245 Salmonella bongori serovar 66:z41:-

0.00 1592 1592 S2 1243617 Salmonella bongori serovar 66:z41:- str. SA19983605

0.00 115622 7649 G1 2614656 unclassified Salmonella

0.00 68405 68405 S 599 Salmonella sp.

0.00 13662 13662 S 2500543 Salmonella sp. SSDFZ69

0.00 8687 8687 S 2879113 Salmonella sp. JXY0409-18

0.00 6370 6370 S 2874627 Salmonella sp. A7

0.00 2572 2572 S 2500542 Salmonella sp. SSDFZ54

0.00 1431 1431 S 2725417 Salmonella sp. SCFS4

0.00 1271 1271 S 2979871 Salmonella sp. 3C

0.00 721 721 S 1179813 Salmonella sp. 40

0.00 588 588 S 2745893 Salmonella sp. SJTUF14154

0.00 586 586 S 2745890 Salmonella sp. SJTUF14076

0.00 493 493 S 2878546 Salmonella sp. A39

0.00 407 407 S 2745892 Salmonella sp. SJTUF14152

0.00 370 370 S 2745895 Salmonella sp. SJTUF14178

0.00 353 353 S 2876620 Salmonella sp. A29-2

0.00 336 336 S 1179812 Salmonella sp. 14

0.00 295 295 S 2745891 Salmonella sp. SJTUF14146

0.00 241 241 S 2833772 Salmonella sp. SJTUF15034

0.00 210 210 S 2833771 Salmonella sp. SJTUF14523

0.00 207 207 S 2204208 Salmonella sp. FORC89

0.00 194 194 S 2745894 Salmonella sp. SJTUF14170

0.00 193 193 S 2816951 Salmonella sp. SAL-045

0.00 141 141 S 2686305 Salmonella sp. S13

0.00 110 110 S 2816953 Salmonella sp. SAL-020

0.00 105 105 S 1179814 Salmonella sp. 96A-29192

0.00 25 25 S 2816952 Salmonella sp. SAL-007

0.04 12074956 2381422 G 1330547 Kosakonia

0.01 2183376 34659 G1 2632876 unclassified Kosakonia

0.00 875691 875691 S 2725560 Kosakonia sp. SMBL-WEM22

0.00 738504 738504 S 2067958 Kosakonia sp. MUSA4

0.00 534522 534522 S 2492396 Kosakonia sp. CCTCC M2018092

0.01 1911288 1666487 S 1158459 Kosakonia sacchari

0.00 244801 244801 S1 1235834 Kosakonia sacchari SP1

0.01 1839401 1573284 S 208223 Kosakonia cowanii

0.00 266117 266117 S1 1300165 Kosakonia cowanii JCM 10956 = DSM 18146

0.00 1135327 1135327 S 283686 Kosakonia radicincitans

0.00 1008610 1008610 S 551989 Kosakonia arachidis

0.00 842010 842010 S 497725 Kosakonia oryzae

0.00 773522 773522 S 1646340 Kosakonia pseudosacchari

0.03 9778337 2128394 G 83654 Leclercia

0.01 4089049 4089049 S 83655 Leclercia adecarboxylata

0.01 2573200 545081 G1 2627398 unclassified Leclercia

0.00 776941 776941 S 2714951 Leclercia sp. 29361

0.00 260698 260698 S 2681307 Leclercia sp. J807

0.00 241783 241783 S 2282310 Leclercia sp. W6

0.00 222919 222919 S 2282309 Leclercia sp. W17

0.00 188263 188263 S 2681308 Leclercia sp. 119287

0.00 181599 181599 S 2898725 Leclercia sp. G3L

0.00 104411 104411 S 1920114 Leclercia sp. LSNIH1

0.00 51331 51331 S 2681309 Leclercia sp. Colony189

0.00 174 174 S 1898428 Leclercia sp.

0.00 987694 987694 S 2815358 Leclercia pneumoniae

0.02 7532578 1737221 G 413496 Cronobacter

0.01 2398461 2273845 S 28141 Cronobacter sakazakii

0.00 91232 91232 S1 1138308 Cronobacter sakazakii ES15

0.00 25894 25894 S1 290339 Cronobacter sakazakii ATCC BAA-894

0.00 7490 7490 S1 956149 Cronobacter sakazakii SP291

0.00 979094 0 S 1163710 Cronobacter condimenti

0.00 979094 979094 S1 1073999 Cronobacter condimenti 1330

0.00 845922 0 S 413497 Cronobacter dublinensis

0.00 845922 0 S1 413498 Cronobacter dublinensis subsp. dublinensis

0.00 845922 845922 S2 1159554 Cronobacter dublinensis subsp. dublinensis LMG 23823

0.00 603527 0 S 535744 Cronobacter universalis

0.00 603527 603527 S1 1074000 Cronobacter universalis NCTC 9529

0.00 531931 499920 S 413503 Cronobacter malonaticus

0.00 32011 32011 S1 1159491 Cronobacter malonaticus LMG 23826

0.00 253601 0 G1 2649764 unclassified Cronobacter

0.00 253601 253601 S 1906275 Cronobacter sp. JZ38

0.00 182821 0 S 413501 Cronobacter muytjensii

0.00 182821 182821 S1 1159613 Cronobacter muytjensii ATCC 51329

0.02 7061963 0 F1 191675 Enterobacteriaceae incertae sedis

0.02 7061963 6282 F2 84563 ant, tsetse, mealybug, aphid, etc. endosymbionts

0.01 2633302 0 F3 84564 ant endosymbionts

0.01 2633302 269088 G 203804 Candidatus Blochmannia

0.00 1523687 192898 G1 711328 unclassified Candidatus Blochmannia

0.00 397089 397089 S 700220 Blochmannia endosymbiont of Camponotus sp.

0.00 192059 192059 S 2681986 Blochmannia endosymbiont of Camponotus nipponensis

0.00 188433 188433 S 1505596 Blochmannia endosymbiont of Polyrhachis (Hedomyrma) turneri

0.00 184949 184949 S 1505597 Blochmannia endosymbiont of Camponotus (Colobopsis) obliquus

0.00 183298 183298 S 2681987 Blochmannia endosymbiont of Colobopsis nipponica

0.00 161953 161953 S 2945587 Blochmannia endosymbiont of Camponotus modoc

0.00 11570 11570 S 2945589 Blochmannia endosymbiont of Camponotus sp. C-046

0.00 11438 11438 S 2945588 Blochmannia endosymbiont of Camponotus sp. C-003

0.00 250339 250339 S 251540 Candidatus Blochmannia vicinus

0.00 186108 0 S 251535 Candidatus Blochmannia vafer

0.00 186108 186108 S1 859654 Candidatus Blochmannia vafer str. BVAF

0.00 180234 180234 S 251538 Candidatus Blochmannia ocreatus

0.00 111038 111038 S 108080 Candidatus Blochmannia herculeanus

0.00 57789 53133 S 101534 Candidatus Blochmannia pennsylvanicus

0.00 4656 4656 S1 291272 Candidatus Blochmannia pennsylvanicus str. BPEN

0.00 55019 0 S 251542 Candidatus Blochmannia chromaiodes

0.00 55019 55019 S1 1240471 Candidatus Blochmannia chromaiodes str. 640

0.01 1862829 62 F3 146507 aphid secondary symbionts

0.00 1147554 0 G 568987 Candidatus Hamiltonella

0.00 1147554 952912 S 138072 Candidatus Hamiltonella defensa

0.00 189646 189646 S1 672795 Candidatus Hamiltonella defensa (Bemisia tabaci)

0.00 4996 4996 S1 572265 Candidatus Hamiltonella defensa 5AT (Acyrthosiphon pisum)

0.00 440914 440914 S 1199245 secondary endosymbiont of Ctenarytaina eucalypti

0.00 274299 274299 S 134287 secondary endosymbiont of Heteropsylla cubana

0.00 1405666 0 G 801 Symbiopectobacterium

0.00 1405666 1405666 S 2871826 Symbiopectobacterium purcellii

0.00 258571 0 G 1906657 Candidatus Doolittlea

0.00 258571 258571 S 1778262 Candidatus Doolittlea endobia

0.00 245218 0 G 1906661 Candidatus Gullanella

0.00 245218 245218 S 1070130 Candidatus Gullanella endobia

0.00 188326 0 G 1906659 Candidatus Hoaglandella

0.00 188326 188326 S 1778263 Candidatus Hoaglandella endobia

0.00 140467 0 G 1682492 Candidatus Tachikawaea

0.00 140467 140467 S 1410383 Candidatus Tachikawaea gelatinosa

0.00 89632 0 G 1906660 Candidatus Mikella

0.00 89632 89632 S 1778264 Candidatus Mikella endobia

0.00 79474 1378 G 1302410 Candidatus Annandia

0.00 42448 42448 S 1345117 Candidatus Annandia pinicola

0.00 35648 35648 S 1302411 Candidatus Annandia adelgestsuga

0.00 76900 0 G 472825 Candidatus Purcelliella

0.00 76900 76900 S 472834 Candidatus Purcelliella pentastirinorum

0.00 75296 0 F3 199891 mealybug secondary endosymbionts

0.00 75296 75296 S 1835721 secondary endosymbiont of Trabutina mannipara

0.02 6628323 242088 G 158483 Cedecea

0.01 4128567 4128567 S 158822 Cedecea neteri

0.00 1189298 0 G1 2649846 unclassified Cedecea

0.00 1189298 1189298 S 2545798 Cedecea sp. FDAARGOS_727

0.00 1068370 1068370 S 158823 Cedecea lapagei

0.02 5168901 29281 G 1330545 Lelliottia

0.01 3739203 3739203 S 61646 Lelliottia amnigena

0.00 1400417 582442 G1 2642424 unclassified Lelliottia

0.00 446727 446727 S 2153385 Lelliottia sp. WB101

0.00 371248 371248 S 2067959 Lelliottia sp. AC1

0.01 4128691 1057704 G 579 Kluyvera

0.00 1400743 1400743 S 61648 Kluyvera intermedia

0.00 1248262 1248262 S 51288 Kluyvera ascorbata

0.00 224526 0 G1 2619995 unclassified Kluyvera

0.00 224526 224526 S 2873269 Kluyvera sp. CRP

0.00 197146 197146 S 2774055 Kluyvera genomosp. 3

0.00 182 182 S 580 Kluyvera cryocrescens

0.00 125 125 S 2774054 Kluyvera genomosp. 2

0.00 3 3 S 73098 Kluyvera georgiana

0.01 3095281 3385 G 1330546 Pluralibacter

0.01 1665011 1665011 S 61647 Pluralibacter gergoviae

0.00 1426885 1107986 S 1334193 [Enterobacter] lignolyticus

0.00 318899 318899 S1 701347 [Enterobacter] lignolyticus SCF1

0.01 3057823 1120329 G 82976 Buttiauxella

0.00 1193869 1193869 S 82977 Buttiauxella agrestis

0.00 396537 0 G1 2634062 unclassified Buttiauxella

0.00 396537 396537 S 2479367 Buttiauxella sp. 3AFRM03

0.00 347088 347088 S 82989 Buttiauxella ferragutiae

0.01 2870588 0 G 702 Plesiomonas

0.01 2870588 2870588 S 703 Plesiomonas shigelloides

0.01 1902093 3429 G 1903434 Atlantibacter

0.01 1588624 1588624 S 565 Atlantibacter hermannii

0.00 310040 310040 S 255519 Atlantibacter subterranea

0.01 1630479 70979 G 447792 Phytobacter

0.00 1182914 1182914 S 1972431 Phytobacter ursingii

0.00 194354 194354 S 395631 Phytobacter diazotrophicus

0.00 182232 0 G1 2625049 unclassified Phytobacter

0.00 182232 182232 S 1756993 Phytobacter sp. SCO41

0.00 1482662 230413 F1 36866 unclassified Enterobacteriaceae

0.00 79867 79867 S 2675792 Enterobacteriaceae endosymbiont of Neohaemonia nigricornis

0.00 79013 79013 S 2675787 Enterobacteriaceae endosymbiont of Donacia tomentosa

0.00 77492 77492 S 2675791 Enterobacteriaceae endosymbiont of Macroplea mutica

0.00 76330 76330 S 2675790 Enterobacteriaceae endosymbiont of Macroplea appendiculata

0.00 56508 56508 S 2675794 Enterobacteriaceae endosymbiont of Plateumaris consimilis

0.00 55675 55675 S 2675793 Enterobacteriaceae endosymbiont of Plateumaris braccata

0.00 54626 54626 S 2675796 Enterobacteriaceae endosymbiont of Plateumaris rustica

0.00 51326 51326 S 2675795 Enterobacteriaceae endosymbiont of Plateumaris pusilla

0.00 51161 51161 S 2675781 Enterobacteriaceae endosymbiont of Donacia provostii

0.00 51122 51122 S 2675797 Enterobacteriaceae endosymbiont of Plateumaris sericea

0.00 47398 47398 S 2675785 Enterobacteriaceae endosymbiont of Donacia sparganii

0.00 45361 45361 S 2675772 Enterobacteriaceae endosymbiont of Donacia bicoloricornis

0.00 45262 45262 S 2675786 Enterobacteriaceae endosymbiont of Donacia thalassina

0.00 44460 44460 S 2675773 Enterobacteriaceae endosymbiont of Donacia cincticornis

0.00 43620 43620 S 2675789 Enterobacteriaceae endosymbiont of Donacia vulgaris

0.00 41766 41766 S 2675782 Enterobacteriaceae endosymbiont of Donacia proxima

0.00 41347 41347 S 2675779 Enterobacteriaceae endosymbiont of Donacia marginata

0.00 41310 41310 S 2675774 Enterobacteriaceae endosymbiont of Donacia cinerea

0.00 39835 39835 S 2675776 Enterobacteriaceae endosymbiont of Donacia crassipes

0.00 39673 39673 S 2675780 Enterobacteriaceae endosymbiont of Donacia piscatrix

0.00 37815 37815 S 2675784 Enterobacteriaceae endosymbiont of Donacia simplex

0.00 36243 36243 S 2675778 Enterobacteriaceae endosymbiont of Donacia fulgens

0.00 33316 33316 S 2675783 Enterobacteriaceae endosymbiont of Donacia semicuprea

0.00 32668 32668 S 2675775 Enterobacteriaceae endosymbiont of Donacia clavipes

0.00 24728 24728 S 2675777 Enterobacteriaceae endosymbiont of Donacia dentata

0.00 24327 24327 S 2675788 Enterobacteriaceae endosymbiont of Donacia versicolorea

0.00 1436093 0 G 2815296 Jejubacter

0.00 1436093 1436093 S 2579935 Jejubacter calystegiae

0.00 1269445 0 G 158876 Yokenella

0.00 1269445 1269445 S 158877 Yokenella regensburgei

0.00 1178025 0 G 2726810 Scandinavium

0.00 1178025 1178025 S 1851514 Scandinavium goeteborgense

0.00 1167515 0 G 1504576 Pseudocitrobacter

0.00 1167496 1167496 S 2891570 Pseudocitrobacter corydidari

0.00 19 19 S 1398493 Pseudocitrobacter faecalis

0.00 1127315 0 G 1335483 Shimwellia

0.00 1127315 1125314 S 563 Shimwellia blattae

0.00 2001 2001 S1 630626 Shimwellia blattae DSM 4481 = NBRC 105725

0.00 933031 88154 G 620 Shigella

0.00 410688 365858 S 623 Shigella flexneri

0.00 12232 5500 S1 42897 Shigella flexneri 2a

0.00 5017 5017 S2 198214 Shigella flexneri 2a str. 301

0.00 1715 1715 S2 198215 Shigella flexneri 2a str. 2457T

0.00 5329 5329 S1 424720 Shigella flexneri Y

0.00 5270 5270 S1 374923 Shigella flexneri 1a

0.00 5000 5000 S1 1617964 Shigella flexneri 4c

0.00 3531 0 S1 373383 Shigella flexneri 5

0.00 3531 3531 S2 373384 Shigella flexneri 5 str. 8401

0.00 3014 3014 S1 1282357 Shigella flexneri 2003036

0.00 2459 2459 S1 424717 Shigella flexneri 3a

0.00 2004 0 S1 424718 Shigella flexneri 5a

0.00 2004 2004 S2 1086030 Shigella flexneri 5a str. M90T

0.00 1773 1773 S1 1282358 Shigella flexneri Shi06HN006

0.00 1708 1708 S1 591020 Shigella flexneri 2002017

0.00 1344 1344 S1 1435046 Shigella flexneri G1663

0.00 746 746 S1 1935181 Shigella flexneri 1c

0.00 322 322 S1 2049486 Shigella flexneri 7b

0.00 59 59 S1 1288825 Shigella flexneri 2b

0.00 27 27 S1 2924859 Shigella flexneri 7a

0.00 12 12 S1 41434 Shigella flexneri 1b

0.00 198333 195424 S 622 Shigella dysenteriae

0.00 2441 2441 S1 300267 Shigella dysenteriae Sd197

0.00 468 468 S1 984897 Shigella dysenteriae 1

0.00 131301 122804 S 621 Shigella boydii

0.00 4549 4549 S1 344609 Shigella boydii CDC 3083-94

0.00 3948 3948 S1 300268 Shigella boydii Sb227

0.00 97422 93666 S 624 Shigella sonnei

0.00 2979 2979 S1 216599 Shigella sonnei 53G

0.00 777 777 S1 300269 Shigella sonnei Ss046

0.00 7133 0 G1 2629414 unclassified Shigella

0.00 6726 6726 S 2906475 Shigella sp. PIB

0.00 359 359 S 1072658 Shigella sp. MO17

0.00 48 48 S 1072659 Shigella sp. LN126

0.00 924593 0 G 2055880 Pseudescherichia

0.00 924593 924593 S 566 Pseudescherichia vulneris

0.00 813969 1021 G 1177214 Candidatus Profftia

0.00 357285 357285 S 1177216 Candidatus Profftia tarda

0.00 248509 0 G1 2735709 unclassified Candidatus Profftia

0.00 248509 248509 S 2864218 Candidatus Profftia sp. (ex Adelges kitamiensis)

0.00 207154 207154 S 1987921 Candidatus Profftia lariciata

0.00 358417 556 G 401618 Candidatus Riesia

0.00 197742 197289 S 401619 Candidatus Riesia pediculicola

0.00 453 453 S1 515618 Candidatus Riesia pediculicola USDA

0.00 160119 160119 S 428411 Candidatus Riesia pediculischaeffi

0.00 208587 0 G 409304 Candidatus Ishikawaella

0.00 208587 0 S 168169 Candidatus Ishikawaella capsulata

0.00 208587 208587 S1 476281 Candidatus Ishikawaella capsulata Mpkobe

0.00 189672 0 G 1081630 Candidatus Schneideria

0.00 189672 189672 S 1081631 Candidatus Schneideria nysicola

0.00 163870 0 G 1048757 Candidatus Moranella

0.00 163870 159282 S 1048758 Candidatus Moranella endobia

0.00 2347 2347 S1 903503 Candidatus Moranella endobia PCIT

0.00 2241 2241 S1 1234603 Candidatus Moranella endobia PCVAL

0.00 47665 0 G 2055876 Metakosakonia

0.00 47665 0 G1 2639707 unclassified Metakosakonia

0.00 47665 47665 S 2487150 Metakosakonia sp. MRY16-398

0.30 92903966 520531 F 1903411 Yersiniaceae

0.16 47690389 14818518 G 613 Serratia

0.03 8148089 7952716 S 615 Serratia marcescens

0.00 72427 72427 S1 911022 Serratia marcescens subsp. marcescens ATCC 13880

0.00 48790 48790 S1 435998 Serratia marcescens WW4

0.00 46352 46352 S1 273526 Serratia marcescens subsp. marcescens Db11

0.00 14320 14320 S1 1401254 Serratia marcescens SMB2099

0.00 13484 13484 S1 1334564 Serratia marcescens SM39

0.02 4700641 348144 G1 2647522 unclassified Serratia

0.00 1460158 1460158 S 104623 Serratia sp. ATCC 39006

0.00 426556 426556 S 2448483 Serratia sp. 3ACOL1

0.00 376911 376911 S 671990 Serratia sp. FGI94

0.00 369236 369236 S 2420306 Serratia sp. FDAARGOS_506

0.00 244688 244688 S 2447890 Serratia sp. 1D1416

0.00 218994 218994 S 2831894 Serratia sp. JSRIV002

0.00 218481 218481 S 2482769 Serratia sp. P2ACOL2

0.00 163226 163226 S 2831896 Serratia sp. JSRIV006

0.00 144740 144740 S 2831895 Serratia sp. JSRIV004

0.00 136920 136920 S 2831893 Serratia sp. JSRIV001

0.00 121418 121418 S 1327989 Serratia sp. FS14

0.00 99590 99590 S 2773264 Serratia sp. Tan611

0.00 75896 75896 S 2724469 Serratia sp. JUb9

0.00 69248 69248 S 2033438 Serratia sp. MYb239

0.00 62821 62821 S 488142 Serratia sp. SCBI

0.00 48446 48446 S 1758196 Serratia sp. SSNIH1

0.00 31598 31598 S 2485839 Serratia sp. LS-1

0.00 28591 28591 S 1938820 Serratia sp. JKS000199

0.00 27677 27677 S 2785630 Serratia sp. CMO1

0.00 23268 23268 S 2663241 Serratia sp. HRI

0.00 2137 2137 S 768493 Serratia sp. AS13

0.00 1897 1897 S 768490 Serratia sp. AS12

0.01 3760947 3542008 S 82996 Serratia plymuthica

0.00 173639 173639 S1 682634 Serratia plymuthica 4Rx13

0.00 41294 41294 S1 1006598 Serratia plymuthica RVH1

0.00 2290 2290 S1 1348660 Serratia plymuthica S13

0.00 1716 1716 S1 768492 Serratia plymuthica AS9

0.01 2610888 2610888 S 47917 Serratia fonticola

0.01 2156569 2156569 S 28151 Serratia proteamaculans

0.01 1643757 1505601 S 614 Serratia liquefaciens

0.00 138156 138156 S1 1346614 Serratia liquefaciens ATCC 27592

0.00 1470418 1470418 S 61652 Serratia rubidaea

0.00 1301158 1301158 S 618 Serratia odorifera

0.00 1213101 1213101 S 138074 Serratia symbiotica

0.00 1203655 1203655 S 82995 Serratia grimesii

0.00 1152714 1152714 S 42906 Serratia entomophila

0.00 1079487 1079487 S 137545 Serratia quinivorans

0.00 644264 0 S 2338073 Serratia inhibens

0.00 644264 644264 S1 1154756 Serratia inhibens PRI-2C

0.00 580559 580559 S 300181 Serratia ureilytica

0.00 527059 527059 S 2741499 Serratia surfactantfaciens

0.00 462189 462189 S 2597702 Serratia rhizosphaerae

0.00 149577 149577 S 61651 Serratia ficaria

0.00 66799 66799 S 458197 Serratia nematodiphila

0.09 27124925 6859685 G 629 Yersinia

0.01 3416572 1338039 G1 1649845 Yersinia pseudotuberculosis complex

0.00 965752 891268 S 633 Yersinia pseudotuberculosis

0.00 29024 0 S1 109458 Yersinia pseudotuberculosis (type O:1b)

0.00 29024 29024 S2 748672 Yersinia pseudotuberculosis str. PA3606

0.00 22970 22970 S1 273123 Yersinia pseudotuberculosis IP 32953

0.00 11645 11645 S1 349747 Yersinia pseudotuberculosis IP 31758

0.00 9536 9536 S1 502801 Yersinia pseudotuberculosis PB1/+

0.00 1309 1309 S1 502800 Yersinia pseudotuberculosis YPIII

0.00 848099 848099 S 367190 Yersinia similis

0.00 264682 194472 S 632 Yersinia pestis

0.00 12281 12281 S1 349746 Yersinia pestis Angola

0.00 9776 9776 S1 637386 Yersinia pestis Z176003

0.00 4924 0 S1 385964 Yersinia pestis subsp. pestis

0.00 4924 3311 S2 1234662 Yersinia pestis subsp. pestis bv. Medievalis

0.00 1613 1613 S3 547048 Yersinia pestis biovar Medievalis str. Harbin 35

0.00 3895 3895 S1 360102 Yersinia pestis Antiqua

0.00 3851 0 S1 1234659 Yersinia pestis subsp. microtus

0.00 3851 3851 S2 229193 Yersinia pestis biovar Microtus str. 91001

0.00 3611 3611 S1 637382 Yersinia pestis D106004

0.00 2995 2995 S1 1035377 Yersinia pestis A1122

0.00 2919 2919 S1 214092 Yersinia pestis CO92

0.00 2728 2728 S1 1345702 Yersinia pestis 2944

0.00 2553 2553 S1 665028 Yersinia pestis EV76-CN

0.00 2449 2449 S1 386656 Yersinia pestis Pestoides F

0.00 1776 1776 S1 1345707 Yersinia pestis 3770

0.00 1526 1526 S1 377628 Yersinia pestis Nepal516

0.00 1524 1524 S1 1345701 Yersinia pestis 790

0.00 1522 1522 S1 748678 Yersinia pestis str. Pestoides B

0.00 1520 1520 S1 1345710 Yersinia pestis 1045

0.00 1447 1447 S1 1345705 Yersinia pestis 1522

0.00 1429 1429 S1 1345708 Yersinia pestis 8787

0.00 1406 1406 S1 187410 Yersinia pestis KIM10+

0.00 1326 1326 S1 649716 Yersinia pestis Pestoides G

0.00 1288 1288 S1 1345706 Yersinia pestis 3067

0.00 1267 1267 S1 1345704 Yersinia pestis 1413

0.00 1179 1179 S1 1345703 Yersinia pestis 1412

0.00 904 904 S1 1455696 Yersinia pestis EV NIIEG

0.00 82 82 S1 880632 Yersinia pestis Java 9

0.00 32 32 S1 412420 Yersinia pestis CA88-4125

0.01 3207601 2751018 S 630 Yersinia enterocolitica

0.00 327260 327260 S1 1443113 Yersinia enterocolitica LC20

0.00 101767 0 S1 34053 Yersinia enterocolitica (type O:5)

0.00 101767 101767 S2 1262462 Yersinia enterocolitica (type O:5) str. YE53/03

0.00 16602 9540 S1 150053 Yersinia enterocolitica subsp. palearctica

0.00 4697 4697 S2 994476 Yersinia enterocolitica subsp. palearctica 105.5R(r)

0.00 2365 2365 S2 930944 Yersinia enterocolitica subsp. palearctica Y11

0.00 10379 8478 S1 150052 Yersinia enterocolitica subsp. enterocolitica

0.00 1901 1901 S2 393305 Yersinia enterocolitica subsp. enterocolitica 8081

0.00 560 0 S1 34055 Yersinia enterocolitica (type O:9)

0.00 560 560 S2 913028 Yersinia enterocolitica W22703

0.00 15 15 S1 34054 Yersinia enterocolitica (type O:8)

0.01 1925610 1925610 S 29486 Yersinia ruckeri

0.00 1421657 1402609 S 29484 Yersinia frederiksenii

0.00 19048 19048 S1 1454377 Yersinia frederiksenii Y225

0.00 1153691 1153691 S 935293 Yersinia entomophaga

0.00 1091680 1091680 S 29485 Yersinia rohdei

0.00 1091653 0 S 29483 Yersinia aldovae

0.00 1091653 1091653 S1 1453495 Yersinia aldovae 670-83

0.00 1072649 1072649 S 2890317 Yersinia alsatica

0.00 1063474 0 S 33060 Yersinia mollaretii

0.00 1063474 1063474 S1 349967 Yersinia mollaretii ATCC 43969

0.00 1016817 1016817 S 2607663 Yersinia canariae

0.00 988444 988444 S 28152 Yersinia kristensenii

0.00 882117 882117 S 631 Yersinia intermedia

0.00 859446 859446 S 263819 Yersinia aleksiciae

0.00 357016 357016 S 2339259 Yersinia hibernica

0.00 318459 318459 S 419257 Yersinia massiliensis

0.00 174416 1 G1 2653513 unclassified Yersinia (in: Bacteria)

0.00 172493 172493 S 1179669 Yersinia sp. KBS0713

0.00 1922 1922 S 1839800 Yersinia sp. FDAARGOS_228

0.00 140350 0 S 634 Yersinia bercovieri

0.00 140350 140350 S1 349968 Yersinia bercovieri ATCC 43970

0.00 83588 83588 S 1604335 Yersinia rochesterensis

0.03 9253836 1906898 G 34037 Rahnella

0.01 2820025 1711895 S 34038 Rahnella aquatilis

0.00 1008662 1008662 S1 745277 Rahnella aquatilis CIP 78.65 = ATCC 33071

0.00 99468 99468 S1 1151116 Rahnella aquatilis HX2

0.00 1415057 202 G1 2635087 unclassified Rahnella

0.00 1405830 1405830 S 1805933 Rahnella sp. ERMR1:05

0.00 2552 2552 S 657335 Rahnella sp. WMR114

0.00 1610 1610 S 657339 Rahnella sp. WMR104

0.00 1181 1181 S 657338 Rahnella sp. WMR121

0.00 1094 1094 S 657334 Rahnella sp. WMR42

0.00 1034 1034 S 657337 Rahnella sp. WMR66

0.00 813 813 S 360016 Rahnella sp. 'WMR15'

0.00 741 741 S 657336 Rahnella sp. WMR120

0.00 1394250 1394250 S 58169 Rahnella inusitata

0.00 1233916 1233916 S 1510570 Rahnella victoriana

0.00 483690 483690 S 2703885 Rahnella aceris

0.01 1638902 0 G 1565532 Rouxiella

0.01 1638902 1638902 S 1646377 Rouxiella badensis

0.01 1555788 0 G 1745211 Chania

0.01 1555788 0 S 1639108 Chania multitudinisentens

0.01 1555788 1555788 S1 1441930 Chania multitudinisentens RB-25

0.00 1491676 0 G 929812 Gibbsiella

0.00 1491676 1491676 S 929813 Gibbsiella quercinecans

0.00 1427204 0 G 41201 Ewingella

0.00 1427204 1427204 S 41202 Ewingella americana

0.00 1373162 0 G 1964366 Nissabacter

0.00 1373162 0 G1 2636213 unclassified Nissabacter

0.00 1373162 1373162 S 2126321 Nissabacter sp. SGAir0207

0.00 827553 0 G 1927833 Candidatus Fukatsuia

0.00 827553 827553 S 1878942 Candidatus Fukatsuia symbiotica

0.23 71292282 575023 F 1903409 Erwiniaceae

0.12 37620995 3270069 G 53335 Pantoea

0.03 8029976 396163 G1 2630326 unclassified Pantoea

0.01 1564877 1564877 S 592316 Pantoea sp. At-9b

0.00 1401009 1401009 S 2052056 Pantoea sp. MSR2

0.00 1392648 1392648 S 2490851 Pantoea sp. CCBC3-3-1

0.00 1090423 1090423 S 2970818 Pantoea sp. SOD02

0.00 1069017 1069017 S 2575375 Pantoea sp. SO10

0.00 860666 860666 S 2886821 Pantoea sp. Z09

0.00 137860 137860 S 2768165 Pantoea sp. MT58

0.00 117313 117313 S 2787629 Pantoea sp. SM3640

0.02 6721692 0 G1 1654067 Pantoea agglomerans group

0.02 6721692 6721692 S 549 Pantoea agglomerans

0.01 4010738 4010738 S 59814 Pantoea dispersa

0.01 3949709 3702799 S 553 Pantoea ananatis

0.00 87749 87749 S1 1095774 Pantoea ananatis PA13

0.00 56032 56032 S1 1123863 Pantoea ananatis LMG 5342

0.00 53107 53107 S1 706191 Pantoea ananatis LMG 20103

0.00 50022 50022 S1 932677 Pantoea ananatis AJ13355

0.01 2874211 2674600 S 470934 Pantoea vagans

0.00 199611 199611 S1 712898 Pantoea vagans C9-1

0.01 1751859 1751859 S 55209 Pantoea cypripedii

0.01 1742347 1315121 S 66269 Pantoea stewartii

0.00 427226 0 S1 66271 Pantoea stewartii subsp. stewartii

0.00 427226 427226 S2 660596 Pantoea stewartii subsp. stewartii DC283

0.01 1666309 1666309 S 1891675 Pantoea alhagi

0.00 1073338 1073338 S 1076550 Pantoea rwandensis

0.00 1057280 1057280 S 470932 Pantoea deleyi

0.00 987066 987066 S 472693 Pantoea eucrina

0.00 319943 319943 S 1235990 Candidatus Pantoea carbekii

0.00 166458 166458 S 470933 Pantoea eucalypti

0.06 19514734 2098352 G 551 Erwinia

0.01 3212680 4672 G1 2622719 unclassified Erwinia

0.00 1371047 1371047 S 2675378 Erwinia sp. E602

0.00 1254038 1254038 S 2681984 Erwinia sp. J780

0.00 294331 294331 S 2547962 Erwinia sp. QL-Z3

0.00 288208 288208 S 215689 Erwinia sp. Ejp617

0.00 384 384 S 231374 Erwinia sp. Ejp 556

0.01 2881727 2881727 S 1922217 Candidatus Erwinia haradaeae

0.01 1834057 1834057 S 55212 Erwinia rhapontici

0.01 1642850 1642850 S 55211 Erwinia persicina

0.01 1641615 1641615 S 1619313 Erwinia gerundensis

0.00 1474331 1474331 S 65700 Erwinia tracheiphila

0.00 1173002 1173002 S 1324864 [Pantoea] beijingensis

0.00 1094678 1038509 S 552 Erwinia amylovora

0.00 23804 23804 S1 1407064 Erwinia amylovora LA637

0.00 23473 23473 S1 716540 Erwinia amylovora ATCC 49946

0.00 2726 2726 S1 1255309 Erwinia amylovora Ea644

0.00 2669 2669 S1 889211 Erwinia amylovora ATCC BAA-2158

0.00 1288 1288 S1 665029 Erwinia amylovora CFBP1430

0.00 1179 1179 S1 1407063 Erwinia amylovora LA636

0.00 856 856 S1 1407062 Erwinia amylovora LA635

0.00 130 130 S1 1027397 Erwinia amylovora ACW56400

0.00 33 33 S1 1255305 Erwinia amylovora CFBP 2585

0.00 11 11 S1 1255303 Erwinia amylovora Ea356

0.00 1056271 0 S 338565 Erwinia tasmaniensis

0.00 1056271 1056271 S1 465817 Erwinia tasmaniensis Et1/99

0.00 913861 531942 S 182337 Erwinia billingiae

0.00 381919 381919 S1 634500 Erwinia billingiae Eb661

0.00 480938 476479 S 79967 Erwinia pyrifoliae

0.00 2355 2355 S1 634499 Erwinia pyrifoliae Ep1/96

0.00 2104 2104 S1 644651 Erwinia pyrifoliae DSM 12163

0.00 10372 0 S 665097 Erwinia piriflorinigrans

0.00 10372 10372 S1 1161919 Erwinia piriflorinigrans CFBP 5888

0.02 5586600 0 G 32199 Buchnera

0.02 5586600 719575 S 9 Buchnera aphidicola

0.00 141967 141967 S1 118103 Buchnera aphidicola (Melaphis rhois)

0.00 139456 139456 S1 1241859 Buchnera aphidicola (Hyadaphis tataricae)

0.00 137911 0 S1 135842 Buchnera aphidicola (Baizongia pistaciae)

0.00 137911 137911 S2 224915 Buchnera aphidicola str. Bp (Baizongia pistaciae)

0.00 133927 133927 S1 118110 Buchnera aphidicola (Schlechtendalia chinensis)

0.00 124168 124168 S1 1241869 Buchnera aphidicola (Muscaphis stroyani)

0.00 122150 122150 S1 118101 Buchnera aphidicola (Diuraphis noxia)

0.00 121096 121096 S1 1241860 Buchnera aphidicola (Hyperomyzus lactucae)

0.00 120060 118611 S1 98795 Buchnera aphidicola (Myzus persicae)

0.00 601 601 S2 1009859 Buchnera aphidicola str. F009 (Myzus persicae)

0.00 538 538 S2 1009858 Buchnera aphidicola str. G002 (Myzus persicae)

0.00 166 166 S2 1009856 Buchnera aphidicola str. USDA (Myzus persicae)

0.00 144 144 S2 1009857 Buchnera aphidicola str. W106 (Myzus persicae)

0.00 119278 119278 S1 557993 Buchnera aphidicola (Brachycaudus cardui)

0.00 117784 117784 S1 2173854 Buchnera aphidicola (Melanaphis sacchari)

0.00 117641 117641 S1 1258543 Buchnera aphidicola (Lipaphis pseudobrassicae)

0.00 113087 113087 S1 1241836 Buchnera aphidicola (Artemisaphis artemisicola)

0.00 112402 112402 S1 118118 Buchnera aphidicola (Uroleucon sonchi)

0.00 111800 111800 S1 911343 Buchnera aphidicola (Brevicoryne brassicae)

0.00 111244 111244 S1 1241865 Buchnera aphidicola (Macrosiphoniella sanborni)

0.00 110886 110886 S1 2495405 Buchnera aphidicola (Nipponaphis monzeni)

0.00 109919 109919 S1 1265350 Buchnera aphidicola (Aphis glycines)

0.00 108278 107294 S1 98794 Buchnera aphidicola (Schizaphis graminum)

0.00 984 984 S2 198804 Buchnera aphidicola str. Sg (Schizaphis graminum)

0.00 107614 0 S1 668607 Buchnera aphidicola (Acyrthosiphon kondoi)

0.00 107614 107614 S2 1005090 Buchnera aphidicola str. Ak (Acyrthosiphon kondoi)

0.00 107518 0 S1 118117 Buchnera aphidicola (Uroleucon ambrosiae)

0.00 107518 107518 S2 1005057 Buchnera aphidicola str. Ua (Uroleucon ambrosiae)

0.00 105190 105190 S1 1241835 Buchnera aphidicola (Aphis nerii)

0.00 103550 103550 S1 2315802 Buchnera aphidicola (Aphis helianthi)

0.00 103437 103437 S1 1241834 Buchnera aphidicola (Aphis nasturtii)

0.00 102493 102493 S1 118109 Buchnera aphidicola (Rhopalosiphum maidis)

0.00 98730 98730 S1 98793 Buchnera aphidicola (Rhopalosiphum padi)

0.00 96827 96827 S1 1241833 Buchnera aphidicola (Anoecia oenotherae)

0.00 96532 96532 S1 2315801 Buchnera aphidicola (Macrosiphum gaurae)

0.00 95757 95757 S1 1241832 Buchnera aphidicola (Acyrthosiphon lactucae)

0.00 93838 93838 S1 1315998 Buchnera aphidicola (Thelaxes californica)

0.00 91468 91468 S1 571430 Buchnera aphidicola (Aphis fabae)

0.00 87493 87493 S1 1507522 Buchnera aphidicola (Macrosiphum euphorbiae)

0.00 87114 87114 S1 1241878 Buchnera aphidicola (Sarucallis kahawaluokalani)

0.00 79414 79414 S1 1921549 Buchnera aphidicola (Cinara strobi)

0.00 76616 76616 S1 1878935 Buchnera aphidicola (Cinara confinis)

0.00 76031 76031 S1 2315800 Buchnera aphidicola (Stegophylla sp.)

0.00 75708 75708 S1 655384 Buchnera aphidicola (Cinara pseudotaxifoliae)

0.00 75120 75120 S1 1660040 Buchnera aphidicola (Cinara cuneomaculata)

0.00 73449 73449 S1 261317 Buchnera aphidicola (Cinara tujafilina)

0.00 70966 70966 S1 2518976 Buchnera aphidicola (Cinara kochiana kochiana)

0.00 68495 68495 S1 2911030 Buchnera aphidicola (Sipha maydis)

0.00 66555 66555 S1 2518977 Buchnera aphidicola (Cinara laricifoliae)

0.00 64612 64612 S1 1660043 Buchnera aphidicola (Cinara piceae)

0.00 63371 63371 S1 2518980 Buchnera aphidicola (Cinara cf. splendens/pseudotsugae 3390)

0.00 62658 62658 S1 2518979 Buchnera aphidicola (Cinara splendens)

0.00 62011 62011 S1 98804 Buchnera aphidicola (Tuberolachnus salignus)

0.00 59331 59331 S1 1241884 Buchnera aphidicola (Therioaphis trifolii)

0.00 55245 0 S1 261318 Buchnera aphidicola (Cinara cedri)

0.00 55245 55245 S2 372461 Buchnera aphidicola BCc

0.00 50639 50639 S1 2709429 Buchnera aphidicola (Periphyllus lyropictus)

0.00 49030 49030 S1 2518975 Buchnera aphidicola (Cinara curvipes)

0.00 48658 48658 S1 2518974 Buchnera aphidicola (Cinara curtihirsuta)

0.00 47110 47110 S1 98785 Buchnera aphidicola (Aphis gossypii)

0.00 36761 36761 S1 571428 Buchnera aphidicola (Sitobion avenae)

0.00 35010 35010 S1 760016 Buchnera aphidicola (Sitobion miscanthi)

0.00 17632 4992 S1 118099 Buchnera aphidicola (Acyrthosiphon pisum)

0.00 7056 7056 S2 107806 Buchnera aphidicola str. APS (Acyrthosiphon pisum)

0.00 2728 2728 S2 713601 Buchnera aphidicola str. JF99 (Acyrthosiphon pisum)

0.00 2660 2660 S2 561501 Buchnera aphidicola str. Tuc7 (Acyrthosiphon pisum)

0.00 196 196 S2 593275 Buchnera aphidicola str. LSR1 (Acyrthosiphon pisum)

0.00 996 996 S1 2559115 Buchnera aphidicola (Aphis craccivore)

0.00 789 789 S1 466616 Buchnera aphidicola (Aphis craccivora)

0.00 203 203 S1 563178 Buchnera aphidicola str. 5A (Acyrthosiphon pisum)

0.02 5459042 273396 G 2100764 Mixta

0.00 1144222 1144222 S 1615494 Mixta intestinalis

0.00 1046562 1046562 S 1458355 Mixta theicola

0.00 1045712 0 G1 2628878 unclassified Mixta

0.00 1045712 1045712 S 2872648 Mixta sp. X22927

0.00 1004963 1004963 S 665914 Mixta gaviniae

0.00 944187 944187 S 665913 Mixta calida

0.01 2390037 6798 G 82986 Tatumella

0.00 1342584 1342584 S 53336 Tatumella citrea

0.00 1039726 1039726 S 82987 Tatumella ptyseos

0.00 929 929 S 642227 Tatumella morbirosei

0.00 145851 0 G 51228 Wigglesworthia

0.00 145851 0 S 51229 Wigglesworthia glossinidia

0.00 145851 0 S1 36868 Wigglesworthia glossinidia endosymbiont of Glossina morsitans

0.00 145851 145851 S2 1142511 Wigglesworthia glossinidia endosymbiont of Glossina morsitans morsitans (Yale colony)

0.20 60612975 629387 F 1903414 Morganellaceae

0.07 21495551 6395042 G 586 Providencia

0.02 5470770 5351557 S 587 Providencia rettgeri

0.00 119213 119213 S1 1141663 Providencia rettgeri Dmel1

0.01 3239620 3239620 S 126385 Providencia alcalifaciens

0.00 1417275 1417275 S 333962 Providencia heimbachae

0.00 1401837 77697 G1 2633465 unclassified Providencia

0.00 1288752 1288752 S 2828763 Providencia sp. R33

0.00 28923 28923 S 2791982 Providencia sp. 2.29

0.00 2760 2760 S 2603245 Providencia sp. 1701091

0.00 1910 1910 S 2603246 Providencia sp. 1709051003

0.00 1795 1795 S 2603244 Providencia sp. 1701011

0.00 1334120 1334120 S 158850 Providencia rustigianii

0.00 1004290 0 S 516075 Providencia sneebia

0.00 1004290 1004290 S1 1141660 Providencia sneebia DSM 19967

0.00 874507 816840 S 588 Providencia stuartii

0.00 57667 57667 S1 1157951 Providencia stuartii MRSN 2154

0.00 289512 289512 S 333965 Providencia vermicola

0.00 68578 68578 S 2027290 Providencia huaxiensis

0.04 13588463 4197099 G 583 Proteus

0.01 3592707 3555927 S 584 Proteus mirabilis

0.00 19735 19735 S1 1266738 Proteus mirabilis BB2000

0.00 17045 17045 S1 529507 Proteus mirabilis HI4320

0.01 2890223 2890223 S 585 Proteus vulgaris

0.00 1007258 1007258 S 183417 Proteus hauseri

0.00 614403 614403 S 102862 Proteus penneri

0.00 565726 3658 G1 257482 unclassified Proteus

0.00 439402 439402 S 2697019 Proteus sp. ZN5

0.00 121426 121426 S 2883107 Proteus sp. NMG38-2

0.00 1240 1240 S 1232493 Proteus sp. 3M

0.00 443214 65514 S 1574161 Proteus terrae

0.00 377700 377700 S1 626774 Proteus terrae subsp. cibarius

0.00 277833 277833 S 1987580 Proteus columbae

0.03 8243447 230351 G 626 Xenorhabdus

0.01 2072363 1301429 S 40576 Xenorhabdus bovienii

0.00 770934 770934 S1 406818 Xenorhabdus bovienii SS-2004

0.00 1352172 1347232 S 628 Xenorhabdus nematophila

0.00 2677 2677 S1 406817 Xenorhabdus nematophila ATCC 19061

0.00 2263 2263 S1 1437823 Xenorhabdus nematophila AN6/1

0.00 1230165 1230165 S 351679 Xenorhabdus hominickii

0.00 1222397 1222397 S 290110 Xenorhabdus budapestensis

0.00 1133121 1133121 S 351671 Xenorhabdus doucetiae

0.00 1002878 0 S 40577 Xenorhabdus poinarii

0.00 1002878 1002878 S1 1354304 Xenorhabdus poinarii G6

0.02 6606430 0 G 581 Morganella

0.02 6606430 6063821 S 582 Morganella morganii

0.00 542609 527312 S1 180434 Morganella morganii subsp. morganii

0.00 15297 15297 S2 1124991 Morganella morganii subsp. morganii KT

0.02 5776827 476923 G 29487 Photorhabdus

0.00 1381054 0 S 2218628 Photorhabdus laumondii

0.00 1381054 1379707 S1 141679 Photorhabdus laumondii subsp. laumondii

0.00 1347 1347 S2 243265 Photorhabdus laumondii subsp. laumondii TTO1

0.00 1340911 1340911 S 291112 Photorhabdus asymbiotica

0.00 1326208 1326208 S 171438 Photorhabdus akhurstii

0.00 1251731 1251731 S 230089 Photorhabdus thracensis

0.01 2832220 236042 G 637 Arsenophonus

0.00 921994 921994 S 638 Arsenophonus nasoniae

0.00 783451 783451 S 2879119 Arsenophonus apicola

0.00 711144 3304 G1 2627083 unclassified Arsenophonus

0.00 531726 531726 S 1231049 Arsenophonus endosymbiont of Aphis craccivora

0.00 176114 176114 S 235559 Arsenophonus endosymbiont of Aleurodicus dispersus

0.00 179589 179589 S 634113 Candidatus Arsenophonus lipoptenae

0.00 1440650 0 G 158848 Moellerella

0.00 1440650 1440650 S 158849 Moellerella wisconsensis

0.17 51046800 395747 F 1903410 Pectobacteriaceae

0.08 23535889 7367131 G 122277 Pectobacterium

0.01 2804690 1469607 S 554 Pectobacterium carotovorum

0.00 1335083 912008 S1 555 Pectobacterium carotovorum subsp. carotovorum

0.00 188039 188039 S2 561230 Pectobacterium carotovorum subsp. carotovorum PC1

0.00 118048 118048 S2 1150613 Pectobacterium carotovorum subsp. carotovorum PCCS1

0.00 116988 116988 S2 1218933 Pectobacterium carotovorum subsp. carotovorum PCC21

0.01 2199557 2199557 S 180957 Pectobacterium brasiliense

0.01 1653840 1588713 S 1905730 Pectobacterium parmentieri

0.00 65127 65127 S1 561231 Pectobacterium parmentieri WPP163

0.00 1264726 1105192 S 29471 Pectobacterium atrosepticum

0.00 159534 159534 S1 218491 Pectobacterium atrosepticum SCRI1043

0.00 1019587 1019587 S 1201031 Pectobacterium aroidearum

0.00 995829 995829 S 2042057 Pectobacterium polaris

0.00 893933 893933 S 2488639 Pectobacterium versatile

0.00 807205 807205 S 2878098 Pectobacterium colocasium

0.00 760223 0 S 55208 Pectobacterium wasabiae

0.00 760223 760223 S1 1175631 Pectobacterium wasabiae CFBP 3304

0.00 732774 732774 S 78398 Pectobacterium odoriferum

0.00 672860 672860 S 2108399 Pectobacterium punjabense

0.00 649191 649191 S 2485124 Pectobacterium polonicum

0.00 561930 1389 G1 2627739 unclassified Pectobacterium

0.00 445002 445002 S 2738983 Pectobacterium sp. PL64

0.00 115539 115539 S 2935858 Pectobacterium sp. 21LCBS03

0.00 486021 486021 S 2778550 Pectobacterium parvum

0.00 402333 402333 S 2774015 Pectobacterium quasiaquaticum

0.00 264059 264059 S 2204145 Pectobacterium aquaticum

0.06 18486720 5853612 G 204037 Dickeya

0.01 3631691 2647014 S 204042 Dickeya zeae

0.00 337385 337385 S1 1427366 Dickeya zeae EC1

0.00 185012 185012 S1 1224146 Dickeya zeae NCPPB 3531

0.00 132674 132674 S1 1308956 Dickeya zeae MS1

0.00 127550 127550 S1 1224153 Dickeya zeae MK19

0.00 103644 103644 S1 1224147 Dickeya zeae NCPPB 3532

0.00 98412 98412 S1 1223573 Dickeya zeae NCPPB 2538

0.01 2132461 1570562 S 204038 Dickeya dadantii

0.00 307550 0 S1 204040 Dickeya dadantii subsp. dieffenbachiae

0.00 307550 307550 S2 1223574 Dickeya dadantii subsp. dieffenbachiae NCPPB 2976

0.00 135458 135458 S1 1224149 Dickeya dadantii NCPPB 3537

0.00 114071 114071 S1 198628 Dickeya dadantii 3937

0.00 4820 4820 S1 1223572 Dickeya dadantii NCPPB 898

0.01 1943335 1085692 S 556 Dickeya chrysanthemi

0.00 517300 517300 S1 1223569 Dickeya chrysanthemi NCPPB 402

0.00 332347 332347 S1 1223571 Dickeya chrysanthemi NCPPB 516

0.00 5661 5661 S1 561229 Dickeya chrysanthemi Ech1591

0.00 2335 2335 S1 1224148 Dickeya chrysanthemi NCPPB 3533

0.00 1408353 1330853 S 204039 Dickeya dianthicola

0.00 34587 34587 S1 1226343 Dickeya dianthicola GBBC 2039

0.00 34445 34445 S1 1225780 Dickeya dianthicola IPO 980

0.00 4085 4085 S1 1223570 Dickeya dianthicola NCPPB 3534

0.00 2427 2427 S1 1306405 Dickeya dianthicola RNS04.9

0.00 1956 1956 S1 1223568 Dickeya dianthicola NCPPB 453

0.00 1004618 1004618 S 568768 Dickeya poaceiphila

0.00 829569 829569 S 1778540 Dickeya fangzhongdai

0.00 682668 667903 S 1089444 Dickeya solani

0.00 4123 4123 S1 1225786 Dickeya solani IPO 2222

0.00 2875 2875 S1 1224151 Dickeya solani MK10

0.00 2076 2076 S1 1224152 Dickeya solani MK16

0.00 1945 1945 S1 1240086 Dickeya solani RNS 08.23.3.1.A

0.00 1900 1900 S1 1226344 Dickeya solani GBBC 2040

0.00 1846 1846 S1 1231725 Dickeya solani D s0432-1

0.00 611640 5444 G1 2622466 unclassified Dickeya

0.00 505657 505657 S 568766 Dickeya sp. NCPPB 3274

0.00 70705 70705 S 2037915 Dickeya sp. Secpp 1600

0.00 18252 18252 S 1225785 Dickeya sp. DW 0440

0.00 8593 8593 S 1224145 Dickeya sp. MK7

0.00 2989 2989 S 1224144 Dickeya sp. CSL RW240

0.00 222780 222780 S 1240404 Dickeya oryzae

0.00 158127 0 S 2893572 Dickeya parazeae

0.00 158127 158127 S1 590409 Dickeya parazeae Ech586

0.00 7866 7866 S 1401087 Dickeya aquatica

0.02 5265981 1522820 G 71655 Brenneria

0.00 1326262 1326262 S 1109412 Brenneria goodwinii

0.00 1308071 1308071 S 2722756 Brenneria izadpanahii

0.00 1093940 1093940 S 55213 Brenneria rubrifaciens

0.00 12264 0 G1 2634434 unclassified Brenneria

0.00 12264 12264 S 598467 Brenneria sp. EniD312

0.00 2624 0 S 55210 Brenneria nigrifluens

0.00 2624 2624 S1 1121120 Brenneria nigrifluens DSM 30175 = ATCC 13028

0.01 2075532 111282 G 1082702 Lonsdalea

0.00 1002776 1002776 S 1082704 Lonsdalea britannica

0.00 961474 961474 S 1172565 Lonsdalea populi

0.00 1286931 0 G 2884243 Musicola

0.00 1286931 1277289 S 69223 Musicola paradisiaca

0.00 5162 5162 S1 579405 Musicola paradisiaca Ech703

0.00 4480 4480 S1 1224150 Musicola paradisiaca NCPPB 2511

0.04 12863208 618820 F 1903412 Hafniaceae

0.02 6280658 2059901 G 635 Edwardsiella

0.01 1662825 1652769 S 636 Edwardsiella tarda

0.00 4546 4546 S1 667121 Edwardsiella tarda ATCC 15947 = NBRC 105688

0.00 3847 3847 S1 718251 Edwardsiella tarda FL6-60

0.00 1663 1663 S1 498217 Edwardsiella tarda EIB202

0.00 1063408 1063408 S 93378 Edwardsiella hoshinae

0.00 952113 949637 S 67780 Edwardsiella ictaluri

0.00 2476 2476 S1 634503 Edwardsiella ictaluri 93-146

0.00 453985 438368 S 1263550 Edwardsiella piscicida

0.00 15617 15617 S1 1288122 Edwardsiella piscicida C07-087

0.00 56464 151 G1 2676541 unclassified Edwardsiella (in: Bacteria)

0.00 45469 45469 S 1578828 Edwardsiella sp. EA181011

0.00 10844 10844 S 1650654 Edwardsiella sp. LADL05-105

0.00 31962 17650 S 1821960 Edwardsiella anguillarum

0.00 14312 14312 S1 667120 Edwardsiella anguillarum ET080813

0.02 5176589 1417251 G 568 Hafnia

0.01 2435752 2202679 S 569 Hafnia alvei

0.00 233073 233073 S1 1453496 Hafnia alvei FB1

0.00 1227564 1227564 S 546367 Hafnia paralvei

0.00 96022 0 G1 2621980 unclassified Hafnia

0.00 96022 96022 S 1848580 Hafnia sp. CBA7124

0.00 787141 0 G 82982 Obesumbacterium

0.00 787141 787141 S 82983 Obesumbacterium proteus

0.02 5641789 0 F 2812006 Bruguierivoracaceae

0.02 5641789 520692 G 84565 Sodalis

0.01 1898456 1898456 S 2697027 Sodalis ligni

0.00 1159067 34903 S 63612 Sodalis glossinidius

0.00 1124164 1124164 S1 343509 Sodalis glossinidius str. 'morsitans'

0.00 1026008 1026008 S 1239307 Sodalis praecaptivus

0.00 542382 0 S 1486991 Candidatus Sodalis pierantonius

0.00 542382 542382 S1 2342 Candidatus Sodalis pierantonius str. SOPE

0.00 495184 0 G1 2636512 unclassified Sodalis

0.00 495184 495184 S 1929246 Sodalis endosymbiont of Henestaris halophilus

0.02 5138421 92582 F 1903416 Budviciaceae

0.00 1522882 0 G 82984 Pragia

0.00 1522882 1522882 S 82985 Pragia fontium

0.00 1306918 0 G 2810357 Jinshanibacter

0.00 1306918 1306918 S 2498113 Jinshanibacter zhutongyuii

0.00 1183433 0 G 82980 Leminorella

0.00 1183433 1183433 S 158841 Leminorella richardii

0.00 1032606 0 G 2172100 Limnobaculum

0.00 1032606 1032606 S 2172103 Limnobaculum parvum

1.90 581858510 111128 O 72274 Pseudomonadales

1.82 556644363 3198765 F 135621 Pseudomonadaceae

1.69 517297361 123213504 G 286 Pseudomonas

0.43 130565544 21133178 G1 196821 unclassified Pseudomonas

0.01 1691184 1691184 S 2931382 Pseudomonas sp. PIA16

0.01 1640444 1640444 S 1028989 Pseudomonas sp. StFLB209

0.01 1608431 1608431 S 2678260 Pseudomonas sp. Ost2

0.00 1459733 1459733 S 2320270 Pseudomonas sp. DG56-2

0.00 1458719 1458719 S 1981174 Pseudomonas sp. M30-35

0.00 1418798 1418798 S 1856685 Pseudomonas sp. TCU-HL1

0.00 1416479 1416479 S 2893890 Pseudomonas sp. KNUC1026

0.00 1395984 1395984 S 1755504 Pseudomonas sp. DY-1

0.00 1358438 1358438 S 2972478 Pseudomonas sp. LS1212

0.00 1348403 1348403 S 2653853 Pseudomonas sp. SCB32

0.00 1331849 1331849 S 2961896 Pseudomonas sp. In5(2022)

0.00 1330127 1330127 S 2975049 Pseudomonas sp. N3-W

0.00 1323652 1323652 S 2883205 Pseudomonas sp. L5B5

0.00 1323495 1323495 S 2866808 Pseudomonas sp. MM211

0.00 1295934 1295934 S 1930532 Pseudomonas sp. CC6-YY-74

0.00 1285878 1285878 S 2895486 Pseudomonas sp. B21-040

0.00 1264459 1264459 S 2049589 Pseudomonas sp. HLS-6

0.00 1240575 1240575 S 1736226 Pseudomonas sp. Leaf58

0.00 1234436 1234436 S 2498848 Pseudomonas sp. MPC6

0.00 1219474 1219474 S 1881017 Pseudomonas sp. 7SR1

0.00 1218168 1218168 S 2811422 Pseudomonas sp. PDNC002

0.00 1213426 1213426 S 1283291 Pseudomonas sp. URMO17WK12:I11

0.00 1194233 1194233 S 2974552 Pseudomonas sp. GCEP-101

0.00 1156969 1156969 S 2706126 Pseudomonas sp. OIL-1

0.00 1154121 1154121 S 2730848 Pseudomonas sp. ADAK18

0.00 1144652 1144652 S 2705472 Pseudomonas sp. MTM4

0.00 1139019 1139019 S 2866807 Pseudomonas sp. MM213

0.00 1131892 1131892 S 2730847 Pseudomonas sp. ADAK13

0.00 1123967 1123967 S 658630 Pseudomonas sp. CMR5c

0.00 1123652 1123652 S 1294143 Pseudomonas sp. ATCC 13867

0.00 1104380 1104380 S 1357074 Pseudomonas sp. LS44

0.00 1103976 1103976 S 2678259 Pseudomonas sp. Seg1

0.00 1097363 1097363 S 2054919 Pseudomonas sp. S09G 359

0.00 1087563 1087563 S 2726989 Pseudomonas sp. gcc21

0.00 1083470 1083470 S 2745519 Pseudomonas sp. OE 28.3

0.00 1079883 1079883 S 2901380 Pseudomonas sp. NIBR-H-19

0.00 1070623 1070623 S 1573719 Pseudomonas sp. S35

0.00 1064274 1064274 S 2748080 Pseudomonas sp. ABC1

0.00 1040817 1040817 S 2052956 Pseudomonas sp. ACM7

0.00 1035018 1035018 S 2201356 Pseudomonas sp. 31-12

0.00 1027388 1027388 S 2895473 Pseudomonas sp. B21-015

0.00 1020171 1020171 S 2874628 Pseudomonas sp. p1(2021b)

0.00 1016912 1016912 S 1573718 Pseudomonas sp. S34

0.00 987727 987727 S 2073078 Pseudomonas sp. DTU12.3

0.00 986919 986919 S 2735906 Pseudomonas sp. B11D7D

0.00 981300 981300 S 2871095 Pseudomonas sp. DR48

0.00 973298 973298 S 1344094 Pseudomonas sp. HN11

0.00 962830 962830 S 2823875 Pseudomonas sp. Tri1

0.00 961011 961011 S 2604941 Pseudomonas sp. C27(2019)

0.00 957430 957430 S 2944234 Pseudomonas sp. LRP2-20

0.00 939615 939615 S 2895480 Pseudomonas sp. B21-028

0.00 928678 928678 S 2219057 Pseudomonas sp. LG1E9

0.00 928641 928641 S 2726956 Pseudomonas sp. MSPm1

0.00 927338 927338 S 2898441 Pseudomonas sp. J452

0.00 912901 912901 S 2973088 Pseudomonas sp. BW7P1

0.00 910637 910637 S 2815936 Pseudomonas sp. PP3

0.00 907295 907295 S 2025658 Pseudomonas sp. NS1(2017)

0.00 896467 896467 S 2895490 Pseudomonas sp. B21-048

0.00 878002 878002 S 658644 Pseudomonas sp. R5-89-07

0.00 840267 840267 S 2804761 Pseudomonas sp. 15A4

0.00 825721 825721 S 2850559 Pseudomonas sp. HS6

0.00 825448 825448 S 2971912 Pseudomonas sp. CBS

0.00 825139 825139 S 2866592 Pseudomonas sp. Marseille-Q3773

0.00 815809 815809 S 2678257 Pseudomonas sp. St316

0.00 788659 788659 S 2812000 Pseudomonas sp. SDM007

0.00 759626 759626 S 2479392 Pseudomonas sp. LTJR-52

0.00 736735 736735 S 1573712 Pseudomonas sp. R84

0.00 719893 719893 S 2614442 Pseudomonas sp. LPB0260

0.00 714917 714917 S 2678261 Pseudomonas sp. Pc102

0.00 701451 701451 S 2825975 Pseudomonas sp. SCA2728.1_7

0.00 689335 689335 S 2920387 Pseudomonas sp. LS.1a

0.00 640796 640796 S 2590776 Pseudomonas sp. NIBRBAC000502773

0.00 638298 638298 S 1573711 Pseudomonas sp. R76

0.00 629573 629573 S 2749999 Pseudomonas sp. RtIB026

0.00 627601 627601 S 2870750 Pseudomonas sp. CYM-20-01

0.00 605320 605320 S 2774459 Pseudomonas sp. IzPS59

0.00 601285 601285 S 2855434 Pseudomonas sp. AO-1

0.00 571736 571736 S 2895491 Pseudomonas sp. B21-051

0.00 567606 567606 S 2866626 Pseudomonas sp. 2hn

0.00 548682 548682 S 2895471 Pseudomonas sp. B21-010

0.00 535434 535434 S 2837969 Pseudomonas sp. FIT81

0.00 507755 507755 S 2841064 Pseudomonas sp. SK3(2021)

0.00 504172 504172 S 2662033 Pseudomonas sp. NY5710

0.00 488537 488537 S 2895475 Pseudomonas sp. B21-019

0.00 487509 487509 S 1649877 Pseudomonas sp. CCOS 191

0.00 458912 458912 S 2870860 Pseudomonas sp. DNDY-54

0.00 448282 448282 S 2862945 Pseudomonas sp. ArH3a

0.00 444489 444489 S 1827300 Pseudomonas sp. MYb193

0.00 441477 441477 S 2729423 Pseudomonas sp. SK

0.00 441444 441444 S 1207075 Pseudomonas sp. UW4

0.00 441143 441143 S 1855380 Pseudomonas sp. Z003-0.4C(8344-21)

0.00 435185 435185 S 2018067 Pseudomonas sp. FDAARGOS_380

0.00 403103 403103 S 1611770 Pseudomonas sp. MRSN 12121

0.00 390187 390187 S 2895476 Pseudomonas sp. B21-021

0.00 364434 364434 S 2834406 Pseudomonas sp. RC3H12

0.00 363257 363257 S 2605424 Pseudomonas sp. J380

0.00 362745 362745 S 2867408 Pseudomonas sp. 3-2

0.00 357407 357407 S 2054914 Pseudomonas sp. 02C 26

0.00 353855 353855 S 1259844 Pseudomonas sp. FGI182

0.00 353196 353196 S 1898684 Pseudomonas sp. LPH1

0.00 352070 352070 S 2795724 Pseudomonas sp. MPFS

0.00 345036 345036 S 1758730 Pseudomonas sp. BIOMIG1BAC

0.00 343870 343870 S 2895489 Pseudomonas sp. B21-047

0.00 341249 341249 S 1534110 Pseudomonas sp. DR 5-09

0.00 337347 337347 S 2774873 Pseudomonas sp. ADPe

0.00 330664 330664 S 2866278 Pseudomonas sp. S07E 245

0.00 317936 317936 S 2054915 Pseudomonas sp. 09C 129

0.00 314629 314629 S 95619 Pseudomonas sp. M1

0.00 308953 308953 S 2871493 Pseudomonas sp. SC3(2021)

0.00 301831 301831 S 2126069 Pseudomonas sp. LBUM920

0.00 298810 298810 S 2730851 Pseudomonas sp. ADAK22

0.00 294382 294382 S 2005388 Pseudomonas sp. RU47

0.00 291217 291217 S 2749808 Pseudomonas sp. Y5-11

0.00 287251 287251 S 1573720 Pseudomonas sp. S49

0.00 285292 285292 S 2879114 Pseudomonas sp. HS-18

0.00 280893 280893 S 2895485 Pseudomonas sp. B21-036

0.00 280648 280648 S 2697023 Pseudomonas sp. AN-B15

0.00 268767 268767 S 1886807 Pseudomonas sp. TMW 2.1634

0.00 260525 260525 S 2678258 Pseudomonas sp. Cab53

0.00 259533 259533 S 118613 Pseudomonas sp. B10

0.00 250523 250523 S 2559074 Pseudomonas sp. S150

0.00 243714 243714 S 2866386 Pseudomonas sp. ERGC3:05

0.00 240848 240848 S 1173283 Pseudomonas sp. R3-18-08

0.00 237488 237488 S 2749807 Pseudomonas sp. Y39-6

0.00 236315 236315 S 2083054 Pseudomonas sp. LG1D9

0.00 233388 233388 S 1636610 Pseudomonas sp. PONIH3

0.00 225217 225217 S 253237 Pseudomonas sp. phDV1

0.00 223796 223796 S 1500687 Pseudomonas sp. St29

0.00 220150 220150 S 2169583 Pseudomonas sp. SXM-1

0.00 218427 218427 S 2895474 Pseudomonas sp. B21-017

0.00 215408 215408 S 107445 Pseudomonas sp. A2

0.00 207585 207585 S 2738843 Pseudomonas sp. B14-6

0.00 202310 202310 S 2654238 Pseudomonas sp. DTU12.1

0.00 198686 198686 S 2886361 Pseudomonas sp. HN8-3

0.00 197572 197572 S 2664899 Pseudomonas sp. CFSAN084952

0.00 197294 197294 S 2895483 Pseudomonas sp. B21-032

0.00 195815 195815 S 2895472 Pseudomonas sp. B21-012

0.00 193684 193684 S 2083053 Pseudomonas sp. SWI44

0.00 192966 192966 S 676210 Pseudomonas sp. ATCC 43928

0.00 192642 192642 S 1855331 Pseudomonas sp. A214

0.00 190844 190844 S 2793103 Pseudomonas sp. IAC-BECa141

0.00 190233 190233 S 2067572 Pseudomonas sp. NC02

0.00 189535 189535 S 1206777 Pseudomonas sp. Lz4W

0.00 187677 187677 S 2083052 Pseudomonas sp. SWI36

0.00 184846 184846 S 1500686 Pseudomonas sp. Os17

0.00 182208 182208 S 2933816 Pseudomonas sp. BC42

0.00 177089 177089 S 1659194 Pseudomonas sp. GR 6-02

0.00 166506 166506 S 2777367 Pseudomonas sp. OST1909

0.00 166006 166006 S 2069256 Pseudomonas sp. XWY-1

0.00 164486 164486 S 2651048 Pseudomonas sp. CFA

0.00 162645 162645 S 2864101 Pseudomonas sp. So3.2b

0.00 162278 162278 S 1173273 Pseudomonas sp. R2-37-08W

0.00 161437 161437 S 2597770 Pseudomonas sp. BJP69

0.00 160732 160732 S 2762896 Pseudomonas sp. MPDS

0.00 159511 159511 S 658642 Pseudomonas sp. R4-34-07

0.00 159374 159374 S 2884805 Pseudomonas sp. HN2

0.00 156929 156929 S 2898483 Pseudomonas sp. 7-41

0.00 156731 156731 S 2895487 Pseudomonas sp. B21-041

0.00 153954 153954 S 1173288 Pseudomonas sp. R4-39-08

0.00 149995 149995 S 2083055 Pseudomonas sp. LH1G9

0.00 148936 148936 S 645292 Pseudomonas sp. T8

0.00 148473 148473 S 1415630 Pseudomonas sp. TKP

0.00 145426 145426 S 1173270 Pseudomonas sp. R1-43-08

0.00 144017 144017 S 1173284 Pseudomonas sp. R3-52-08

0.00 140045 140045 S 1602166 Pseudomonas sp. St290

0.00 137815 137815 S 2886360 Pseudomonas sp. HN2-3

0.00 136778 136778 S 2870840 Pseudomonas sp. DR208

0.00 127358 127358 S 147212 Pseudomonas sp. SW-3

0.00 127204 127204 S 2961531 Pseudomonas sp. KHPS1

0.00 125573 125573 S 658643 Pseudomonas sp. R4-35-07

0.00 122505 122505 S 2892442 Pseudomonas sp. CIP-10

0.00 118647 118647 S 1173280 Pseudomonas sp. R2-60-08W

0.00 117288 117288 S 69328 Pseudomonas sp. VLB120

0.00 116956 116956 S 2926671 Pseudomonas sp. I3-I5

0.00 113539 113539 S 1702250 Pseudomonas sp. IB20

0.00 113053 113053 S 2895484 Pseudomonas sp. B21-035

0.00 109669 109669 S 2861799 Pseudomonas sp. Colony2

0.00 107452 107452 S 2821559 Pseudomonas sp. IsoF

0.00 104442 104442 S 2813842 Pseudomonas sp. SORT22

0.00 102801 102801 S 2856556 Pseudomonas sp. HD6515

0.00 98611 98611 S 658632 Pseudomonas sp. R11-23-07

0.00 97577 97577 S 2895477 Pseudomonas sp. B21-023

0.00 96360 96360 S 1338689 Pseudomonas sp. JY-Q

0.00 95287 95287 S 2895482 Pseudomonas sp. B21-031

0.00 93176 93176 S 658641 Pseudomonas sp. R2-7-07

0.00 93018 93018 S 2799184 Pseudomonas sp. Eqa60

0.00 90497 90497 S 2587597 Pseudomonas sp. SWI7

0.00 86499 86499 S 2609418 Pseudomonas sp. KUIN-1

0.00 82422 82422 S 1573704 Pseudomonas sp. R32

0.00 77064 77064 S 2083051 Pseudomonas sp. SWI6

0.00 75299 75299 S 2866282 Pseudomonas sp. PS1(2021)

0.00 74515 74515 S 2841063 Pseudomonas sp. SK2

0.00 69968 69968 S 1495331 Pseudomonas sp. WCS374

0.00 68837 68837 S 2678256 Pseudomonas sp. St386

0.00 65156 65156 S 306 Pseudomonas sp.

0.00 59261 59261 S 2895488 Pseudomonas sp. B21-044

0.00 57904 57904 S 2724470 Pseudomonas sp. BIGb0427

0.00 56257 56257 S 2479393 Pseudomonas sp. LTGT-11-2Z

0.00 50995 50995 S 2895470 Pseudomonas sp. B21-009

0.00 48112 48112 S 2829498 Pseudomonas sp. JS425

0.00 36340 36340 S 2662034 Pseudomonas sp. 13159349

0.00 30496 30496 S 2545800 Pseudomonas sp. FDAARGOS_761

0.00 28855 28855 S 1761895 Pseudomonas sp. bs2935

0.00 20587 20587 S 76885 Pseudomonas sp. K-62

0.00 17724 17724 S 2944392 Pseudomonas sp. BYT-5

0.00 16738 16738 S 1329969 Pseudomonas sp. GLE121

0.00 6773 6773 S 2578110 Pseudomonas sp. KBS0707

0.00 6602 6602 S 545908 Pseudomonas sp. LM8

0.00 3102 3102 S 2730845 Pseudomonas sp. ADAK2

0.00 3041 3041 S 2730846 Pseudomonas sp. ADAK7

0.00 2981 2981 S 2816906 Pseudomonas sp. BYT-1

0.00 2863 2863 S 2730850 Pseudomonas sp. ADAK21

0.00 2726 2726 S 2866277 Pseudomonas sp. S11A 273

0.00 2541 2541 S 2730849 Pseudomonas sp. ADAK20

0.00 2491 2491 S 2054916 Pseudomonas sp. S06B 330

0.00 2466 2466 S 2578115 Pseudomonas sp. KBS0802

0.00 2323 2323 S 2587859 Pseudomonas sp. THAF42

0.00 2231 2231 S 2759706 Pseudomonas sp. 29A

0.00 2206 2206 S 2743971 Pseudomonas sp. 43A

0.00 2183 2183 S 2587863 Pseudomonas sp. THAF187a

0.00 1642 1642 S 341029 Pseudomonas sp. CT14

0.00 1596 1596 S 1523522 Pseudomonas sp. ZM1

0.00 1522 1522 S 322535 Pseudomonas sp. S19

0.00 1500 1500 S 115714 Pseudomonas sp. S-47

0.00 1486 1486 S 1449783 Pseudomonas sp. LM12

0.00 1466 1466 S 1573714 Pseudomonas sp. S04

0.00 347 347 S 1524461 Pseudomonas sp. EGD-AKN5

0.00 49 49 S 1123041 Pseudomonas sp. MC1

0.00 12 12 S 180590 Pseudomonas sp. SLT2001

0.18 56405598 1618674 G1 136843 Pseudomonas fluorescens group

0.07 21165130 16090293 S 294 Pseudomonas fluorescens

0.00 1174906 1174906 S1 1038922 Pseudomonas fluorescens Q2-87

0.00 1098348 1098348 S1 1221522 Pseudomonas fluorescens NCIMB 11764

0.00 1017131 1017131 S1 746360 Pseudomonas fluorescens WH6

0.00 751703 751703 S1 743713 Pseudomonas fluorescens R124

0.00 435782 435782 S1 205922 Pseudomonas fluorescens Pf0-1

0.00 261635 261635 S1 216595 Pseudomonas fluorescens SBW25

0.00 192309 192309 S1 463794 Pseudomonas fluorescens BBc6R8

0.00 109954 109954 S1 1037911 Pseudomonas fluorescens A506

0.00 33069 33069 S1 1038923 Pseudomonas fluorescens Q8r1-96

0.02 4699455 4476906 S 380021 Pseudomonas protegens

0.00 215107 215107 S1 1420599 Pseudomonas protegens Cab57

0.00 4672 4672 S1 1124983 Pseudomonas protegens CHA0

0.00 2770 2770 S1 220664 Pseudomonas protegens Pf-5

0.01 3967362 3791154 S 47883 Pseudomonas synxantha

0.00 176208 176208 S1 96901 Pseudomonas synxantha BG33R

0.01 3947671 3947671 S 76758 Pseudomonas orientalis

0.01 2548757 2548757 S 47878 Pseudomonas azotoformans

0.01 2230070 2230070 S 76760 Pseudomonas rhodesiae

0.01 1783130 1519061 S 200451 Pseudomonas poae

0.00 264069 264069 S1 1282356 Pseudomonas poae RE*1-1-14

0.01 1683033 1683033 S 200450 Pseudomonas trivialis

0.00 1465718 1465718 S 29442 Pseudomonas tolaasii

0.00 1336890 1336890 S 47879 Pseudomonas corrugata

0.00 1267719 1267719 S 46679 Pseudomonas mucidolens

0.00 1163388 1163388 S 183795 Pseudomonas mediterranea

0.00 1079789 1079789 S 651740 Pseudomonas cedrina

0.00 1021441 1021441 S 200452 Pseudomonas congelans

0.00 981337 981337 S 78543 Pseudomonas migulae

0.00 978369 978369 S 129817 Pseudomonas brenneri

0.00 925565 925565 S 76761 Pseudomonas veronii

0.00 893070 893070 S 75588 Pseudomonas libanensis

0.00 828940 532765 S 75612 Pseudomonas mandelii

0.00 296175 296175 S1 1147786 Pseudomonas mandelii JR-1

0.00 502871 502871 S 1114970 Pseudomonas ogarae

0.00 317219 317219 S 169669 Pseudomonas extremorientalis

0.11 32378115 1321357 G1 136845 Pseudomonas putida group

0.07 21082249 19333615 S 303 Pseudomonas putida

0.00 672924 672924 S1 1384061 Pseudomonas putida S13.1.2

0.00 181016 181016 S1 1331671 Pseudomonas putida H8234

0.00 148297 148297 S1 1196325 Pseudomonas putida DOT-T1E

0.00 138227 138227 S1 1081940 Pseudomonas putida B6-2

0.00 112924 112924 S1 1042876 Pseudomonas putida S16

0.00 100909 100909 S1 76869 Pseudomonas putida GB-1

0.00 84566 84566 S1 1215088 Pseudomonas putida HB3267

0.00 82063 82063 S1 931281 Pseudomonas putida BIRD-1

0.00 75602 75602 S1 1150601 Pseudomonas putida JB

0.00 68898 68898 S1 1215087 Pseudomonas putida S12

0.00 40941 40941 S1 1193499 Pseudomonas putida SJTE-1

0.00 36942 36942 S1 231023 Pseudomonas putida ND6

0.00 3023 3023 S1 1211579 Pseudomonas putida NBRC 14164

0.00 2302 2302 S1 160488 Pseudomonas putida KT2440

0.01 2617284 2617284 S 47885 Pseudomonas oryzihabitans

0.01 2322153 1275270 S 47880 Pseudomonas fulva

0.00 1046883 1046883 S1 743720 Pseudomonas fulva 12-X

0.00 1300520 1288996 S 76759 Pseudomonas monteilii

0.00 9177 9177 S1 1435044 Pseudomonas monteilii SB3078

0.00 2347 2347 S1 1435058 Pseudomonas monteilii SB3101

0.00 1175877 1175877 S 70775 Pseudomonas plecoglossicida

0.00 1089984 1089984 S 78327 Pseudomonas mosselii

0.00 879778 879778 S 1785161 Pseudomonas wadenswilerensis

0.00 459839 459839 S 2217867 Pseudomonas sp. SGAir0191

0.00 129074 129074 S 1940636 Pseudomonas shirazica

0.10 31185069 7058102 G1 136849 Pseudomonas syringae group

0.03 8883986 0 G2 251695 Pseudomonas syringae group genomosp. 1

0.03 8883986 4699225 S 317 Pseudomonas syringae

0.00 1127276 1285 S1 264449 Pseudomonas syringae group pathovars incertae sedis

0.00 1026609 977475 S2 103796 Pseudomonas syringae pv. actinidiae

0.00 39085 39085 S3 1108972 Pseudomonas syringae pv. actinidiae str. Shaanxi_M228

0.00 3747 3747 S3 1104678 Pseudomonas syringae pv. actinidiae ICMP 9853

0.00 3545 3545 S3 1194418 Pseudomonas syringae pv. actinidiae ICMP 9617

0.00 2317 2317 S3 1104679 Pseudomonas syringae pv. actinidiae ICMP 18708

0.00 440 440 S3 1095103 Pseudomonas syringae pv. actinidiae ICMP 18884

0.00 99382 99382 S2 264451 Pseudomonas syringae pv. cerasicola

0.00 1027571 474364 S1 321 Pseudomonas syringae pv. syringae

0.00 168156 168156 S2 1262350 Pseudomonas syringae pv. syringae SM

0.00 117766 117766 S2 1260626 Pseudomonas syringae pv. syringae B64

0.00 111040 111040 S2 1324932 Pseudomonas syringae pv. syringae HS191

0.00 82576 82576 S2 1324931 Pseudomonas syringae pv. syringae B301D

0.00 73669 73669 S2 205918 Pseudomonas syringae pv. syringae B728a

0.00 992510 992510 S1 1357279 Pseudomonas syringae CC1557

0.00 213400 213400 S1 663959 Pseudomonas syringae pv. avii

0.00 178064 0 S1 59510 Pseudomonas syringae pv. pisi

0.00 178064 178064 S2 1357292 Pseudomonas syringae pv. pisi str. PP1

0.00 133318 133318 S1 1357289 Pseudomonas syringae USA011

0.00 116483 116483 S1 1332075 Pseudomonas syringae UMAF0158

0.00 115574 115574 S1 192087 Pseudomonas syringae pv. atrofaciens

0.00 113536 113536 S1 199201 Pseudomonas syringae pv. lapsa

0.00 90864 90864 S1 1357284 Pseudomonas syringae CC440

0.00 73781 73781 S1 1357287 Pseudomonas syringae UB303

0.00 2384 2384 S1 629264 Pseudomonas syringae Cit 7

0.01 4295810 4295810 S 33069 Pseudomonas viridiflava

0.01 2789684 645921 G2 251698 Pseudomonas syringae group genomosp. 2

0.00 1435422 517667 S 47877 Pseudomonas amygdali

0.00 483755 165686 S1 53707 Pseudomonas amygdali pv. lachrymans

0.00 318069 318069 S2 629260 Pseudomonas amygdali pv. lachrymans str. M301315

0.00 156137 66440 S1 322 Pseudomonas amygdali pv. tabaci

0.00 89697 89697 S2 573066 Pseudomonas amygdali pv. tabaci str. ATCC 11528

0.00 139951 139951 S1 1664860 Pseudomonas amygdali pv. loropetali

0.00 124975 124975 S1 251722 Pseudomonas amygdali pv. aesculi

0.00 12937 12937 S1 129138 Pseudomonas amygdali pv. morsprunorum

0.00 503147 315815 S 29438 Pseudomonas savastanoi

0.00 176251 137655 S1 360920 Pseudomonas savastanoi pv. savastanoi

0.00 38596 38596 S2 693985 Pseudomonas savastanoi pv. savastanoi NCPPB 3335

0.00 11081 0 S1 319 Pseudomonas savastanoi pv. phaseolicola

0.00 11081 11081 S2 264730 Pseudomonas savastanoi pv. phaseolicola 1448A

0.00 205194 205194 S 53410 Pseudomonas ficuserectae

0.01 2595964 2592951 S 36746 Pseudomonas cichorii

0.00 3013 3013 S1 1441629 Pseudomonas cichorii JBC1

0.00 1455644 983066 S 251699 Pseudomonas syringae group genomosp. 7

0.00 237269 237269 S1 129140 Pseudomonas syringae pv. tagetis

0.00 235309 235309 S1 251654 Pseudomonas syringae pv. helianthi

0.00 1393343 1393343 S 1190415 Pseudomonas asturiensis

0.00 871884 621260 S 50340 Pseudomonas fuscovaginae

0.00 250624 250624 S1 1150862 Pseudomonas fuscovaginae UPB0736

0.00 531938 219335 S 53409 Pseudomonas coronafaciens

0.00 229626 0 S1 235277 Pseudomonas coronafaciens pv. oryzae

0.00 229626 229626 S2 563797 Pseudomonas coronafaciens pv. oryzae str. 1_6

0.00 79427 79427 S1 235275 Pseudomonas coronafaciens pv. coronafaciens

0.00 3550 3550 S1 251653 Pseudomonas coronafaciens pv. garcae

0.00 521403 139759 S 251701 Pseudomonas syringae group genomosp. 3

0.00 205689 199880 S1 323 Pseudomonas syringae pv. tomato

0.00 5809 5809 S2 223283 Pseudomonas syringae pv. tomato str. DC3000

0.00 175955 170110 S1 59511 Pseudomonas syringae pv. maculicola

0.00 3081 3081 S2 629265 Pseudomonas syringae pv. maculicola str. ES4326

0.00 2764 2764 S2 698750 Pseudomonas syringae pv. maculicola str. M6

0.00 456581 456581 S 46257 Pseudomonas avellanae

0.00 287941 287941 S 200454 Pseudomonas tremae

0.00 42789 0 S 86840 Pseudomonas cannabina

0.00 42789 42789 S1 757414 Pseudomonas cannabina pv. alisalensis

0.08 25883315 206362 G1 136841 Pseudomonas aeruginosa group

0.04 13403823 13032862 S 287 Pseudomonas aeruginosa

0.00 159170 159170 S1 381754 Pseudomonas aeruginosa PA7

0.00 33837 33837 S1 1415629 Pseudomonas aeruginosa MTB-1

0.00 27287 27287 S1 1443105 Pseudomonas aeruginosa SG17M

0.00 19786 19786 S1 1457392 Pseudomonas aeruginosa PA96

0.00 17120 17120 S1 1280938 Pseudomonas aeruginosa B136-33

0.00 14844 14844 S1 1009714 Pseudomonas aeruginosa PAK

0.00 14827 14827 S1 1093787 Pseudomonas aeruginosa DK2

0.00 13417 13417 S1 1448140 Pseudomonas aeruginosa YL84

0.00 12225 12225 S1 1400868 Pseudomonas aeruginosa VRFPA04

0.00 7733 7733 S1 1427342 Pseudomonas aeruginosa SCV20265

0.00 6197 6197 S1 1352355 Pseudomonas aeruginosa c7447m

0.00 4493 4493 S1 941193 Pseudomonas aeruginosa M18

0.00 3991 3991 S1 1193501 Pseudomonas aeruginosa SJTD-1

0.00 3799 3799 S1 910265 Pseudomonas aeruginosa C-NN2

0.00 3638 3638 S1 798130 Pseudomonas aeruginosa 39016

0.00 3147 3147 S1 1340851 Pseudomonas aeruginosa RP73

0.00 3140 3140 S1 1089456 Pseudomonas aeruginosa NCGM2.S1

0.00 2479 2479 S1 208963 Pseudomonas aeruginosa UCBPP-PA14

0.00 2474 2474 S1 1158200 Pseudomonas aeruginosa TBCF10839

0.00 2064 2064 S1 1163396 Pseudomonas aeruginosa CI27

0.00 1441 1441 S1 388272 Pseudomonas aeruginosa PACS2

0.00 1410 882 S1 208964 Pseudomonas aeruginosa PAO1

0.00 421 421 S2 1147787 Pseudomonas aeruginosa PAO1H2O

0.00 55 55 S2 1367493 Pseudomonas aeruginosa PAO1-VE2

0.00 52 52 S2 1367494 Pseudomonas aeruginosa PAO1-VE13

0.00 1326 1326 S1 1411700 Pseudomonas aeruginosa DHS01

0.00 1063 1063 S1 1408276 Pseudomonas aeruginosa LESlike5

0.00 1051 1051 S1 1408271 Pseudomonas aeruginosa LES400

0.00 962 962 S1 1051003 Pseudomonas aeruginosa 19BR

0.00 912 912 S1 1408275 Pseudomonas aeruginosa LESlike4

0.00 892 892 S1 1408273 Pseudomonas aeruginosa LESB65

0.00 880 880 S1 1408277 Pseudomonas aeruginosa LESlike7

0.00 805 805 S1 1051004 Pseudomonas aeruginosa 213BR

0.00 800 800 S1 1408272 Pseudomonas aeruginosa LES431

0.00 794 794 S1 1408274 Pseudomonas aeruginosa LESlike1

0.00 773 773 S1 652611 Pseudomonas aeruginosa PA14

0.00 627 627 S1 1279008 Pseudomonas aeruginosa PA1R

0.00 494 494 S1 1279007 Pseudomonas aeruginosa PA1

0.00 471 471 S1 557722 Pseudomonas aeruginosa LESB58

0.00 240 240 S1 1123015 Pseudomonas aeruginosa DSM 50071 = NBRC 12689

0.00 229 229 S1 1356855 Pseudomonas aeruginosa DK1

0.00 123 123 S1 1352354 Pseudomonas aeruginosa PAO581

0.01 3347697 2833557 S 300 Pseudomonas mendocina

0.00 269220 269220 S1 1225174 Pseudomonas mendocina S5.2

0.00 244920 244920 S1 1001585 Pseudomonas mendocina NK-01

0.01 2119964 2119964 S 43263 Pseudomonas alcaligenes

0.01 1981473 234 G2 1232139 Pseudomonas oleovorans/pseudoalcaligenes group

0.00 1423104 1423104 S 1149133 Pseudomonas furukawaii

0.00 558135 319375 S 301 Pseudomonas oleovorans

0.00 238760 238760 S1 1182590 Pseudomonas pseudoalcaligenes CECT 5344

0.01 1928111 1928111 S 53408 Pseudomonas citronellolis

0.00 1509480 29493 G2 627141 Pseudomonas nitroreducens/multiresinivorans group

0.00 947593 947593 S 95301 Pseudomonas multiresinivorans

0.00 532394 532394 S 46680 Pseudomonas nitroreducens

0.00 1386405 30 S 53412 Pseudomonas resinovorans

0.00 1386375 1386375 S1 1245471 Pseudomonas resinovorans NBRC 106553

0.05 14998859 13741 G1 136842 Pseudomonas chlororaphis group

0.04 11026474 7634369 S 587753 Pseudomonas chlororaphis

0.01 1688605 1688605 S1 86192 Pseudomonas chlororaphis subsp. aurantiaca

0.00 704014 496295 S1 587851 Pseudomonas chlororaphis subsp. aureofaciens

0.00 207719 207719 S2 1038921 Pseudomonas chlororaphis subsp. aureofaciens 30-84

0.00 579453 579453 S1 1513890 Pseudomonas chlororaphis subsp. piscium

0.00 314758 314758 S1 333 Pseudomonas chlororaphis subsp. chlororaphis

0.00 105275 105275 S1 1037915 Pseudomonas chlororaphis O6

0.01 2492102 2492102 S 296 Pseudomonas fragi

0.00 1466542 1466542 S 86185 Pseudomonas lundensis

0.01 3790413 3790413 S 237610 Pseudomonas psychrotolerans

0.01 3049572 2231303 S 312306 Pseudomonas entomophila

0.00 818269 818269 S1 384676 Pseudomonas entomophila L48

0.01 2643587 2643587 S 104087 Pseudomonas frederiksbergensis

0.01 2412693 2345850 S 930166 Pseudomonas brassicacearum

0.00 66843 0 S1 86264 Pseudomonas brassicacearum subsp. brassicacearum

0.00 66843 66843 S2 994484 Pseudomonas brassicacearum subsp. brassicacearum NFM421

0.01 2389827 2389827 S 515393 Pseudomonas yamanorum

0.01 2338730 2338730 S 319939 Pseudomonas otitidis

0.01 2332330 1873474 S 65741 Pseudomonas knackmussii

0.00 458856 458856 S1 1301098 Pseudomonas knackmussii B13

0.01 1958029 1958029 S 237609 Pseudomonas alkylphenolica

0.01 1954946 1954946 S 198618 Pseudomonas umsongensis

0.01 1759742 1759742 S 191390 Pseudomonas palleroniana

0.01 1755418 1755418 S 157782 Pseudomonas parafulva

0.01 1662241 1662241 S 702115 Pseudomonas arsenicoxydans

0.01 1616254 1616254 S 2599595 Pseudomonas eucalypticola

0.01 1575481 1575481 S 2604832 Pseudomonas lalkuanensis

0.01 1562194 1562194 S 485898 Pseudomonas promysalinigenes

0.01 1560905 1560905 S 2745495 Pseudomonas vanderleydeniana

0.00 1472910 1472910 S 359110 Pseudomonas extremaustralis

0.00 1383071 1383071 S 321846 Pseudomonas simiae

0.00 1378684 1378684 S 198620 Pseudomonas koreensis

0.00 1339152 1339152 S 46677 Pseudomonas agarici

0.00 1336256 1336256 S 437900 Pseudomonas marincola

0.00 1312275 1312275 S 95300 Pseudomonas vancouverensis

0.00 1286424 1286424 S 2684212 Pseudomonas izuensis

0.00 1239558 1239558 S 122355 Pseudomonas psychrophila

0.00 1230156 1230156 S 47884 Pseudomonas taetrolens

0.00 1218244 1218244 S 244566 Pseudomonas lurida

0.00 1200341 1200341 S 216142 Pseudomonas rhizosphaerae

0.00 1198351 1198351 S 86265 Pseudomonas thivervalensis

0.00 1188707 1188707 S 219572 Pseudomonas antarctica

0.00 1184967 1184967 S 2842346 Pseudomonas siliginis

0.00 1184220 1184220 S 2565368 Pseudomonas atacamensis

0.00 1176350 1176350 S 1931241 Pseudomonas phragmitis

0.00 1169813 1169813 S 1853130 Pseudomonas silesiensis

0.00 1156752 1156752 S 1148509 Pseudomonas prosekii

0.00 1155222 907746 S 321662 Pseudomonas moraviensis

0.00 247476 247476 S1 1395516 Pseudomonas moraviensis R28-S

0.00 1144824 1144824 S 1421430 Pseudomonas granadensis

0.00 1138835 1138835 S 157783 Pseudomonas cremoricolorata

0.00 1138043 1138043 S 1788301 Pseudomonas versuta

0.00 1133631 1133631 S 2320867 Pseudomonas cavernae

0.00 1131159 1131159 S 640205 Pseudomonas seleniipraecipitans

0.00 1123060 1123060 S 2518644 Pseudomonas tructae

0.00 1080348 1080348 S 556533 Pseudomonas benzenivorans

0.00 1068297 1068297 S 1392877 Pseudomonas oryzae

0.00 1063580 1063580 S 364197 Pseudomonas pohangensis

0.00 1046502 1046502 S 2830842 Pseudomonas nanhaiensis

0.00 1044616 1044616 S 2745504 Pseudomonas hamedanensis

0.00 987620 987620 S 2505979 Pseudomonas viciae

0.00 983219 983219 S 2842349 Pseudomonas asgharzadehiana

0.00 977384 977384 S 163011 Pseudomonas lini

0.00 955404 955404 S 1499686 Pseudomonas saudiphocaensis

0.00 920497 920497 S 2745490 Pseudomonas xantholysinigenes

0.00 915324 915324 S 2745510 Pseudomonas zeae

0.00 905485 905485 S 395598 Pseudomonas reinekei

0.00 899257 899257 S 289370 Pseudomonas argentinensis

0.00 894454 894454 S 2842355 Pseudomonas fakonensis

0.00 883875 883875 S 2842357 Pseudomonas muyukensis

0.00 839095 839095 S 2842356 Pseudomonas xanthosomae

0.00 839065 839065 S 2731681 Pseudomonas campi

0.00 807330 807330 S 47886 Pseudomonas luteola

0.00 799289 799289 S 2745518 Pseudomonas tritici

0.00 790222 790222 S 2011015 Pseudomonas laurylsulfatiphila

0.00 786243 786243 S 158627 Pseudomonas graminis

0.00 773291 773291 S 1628086 Pseudomonas kribbensis

0.00 747503 747503 S 1691904 Pseudomonas sediminis

0.00 742923 742923 S 2774460 Pseudomonas allokribbensis

0.00 717338 717338 S 2906062 Pseudomonas wenzhouensis

0.00 704135 704135 S 1245526 Pseudomonas guangdongensis

0.00 698219 698219 S 2725477 Pseudomonas tohonis

0.00 654330 654330 S 2774461 Pseudomonas gozinkensis

0.00 651361 651361 S 2842354 Pseudomonas maumuensis

0.00 615465 615465 S 2745511 Pseudomonas tensinigenes

0.00 606505 606505 S 2493633 Pseudomonas hydrolytica

0.00 568646 568646 S 1302376 Candidatus Pseudomonas adelgestsugas

0.00 536408 536408 S 2746321 Pseudomonas phenolilytica

0.00 497803 497803 S 2745514 Pseudomonas salmasensis

0.00 494953 494953 S 53407 Pseudomonas asplenii

0.00 459370 459370 S 1615674 Pseudomonas lactis

0.00 455661 455661 S 2213015 Pseudomonas sichuanensis

0.00 454061 454061 S 2842350 Pseudomonas azerbaijanoriens

0.00 438007 438007 S 2745498 Pseudomonas zarinae

0.00 420105 0 S 101564 Pseudomonas alcaliphila

0.00 420105 420105 S1 741155 Pseudomonas alcaliphila JAB1

0.00 395458 395458 S 2666183 Pseudomonas juntendi

0.00 393524 393524 S 489632 Pseudomonas chengduensis

0.00 392184 392184 S 485870 Pseudomonas anuradhapurensis

0.00 381832 381832 S 1163398 Pseudomonas donghuensis

0.00 380368 380368 S 2745503 Pseudomonas iranensis

0.00 371623 371623 S 554344 Pseudomonas toyotomiensis

0.00 346643 346643 S 2842348 Pseudomonas alvandae

0.00 345620 345620 S 2745509 Pseudomonas monsensis

0.00 338326 338326 S 2056231 Pseudomonas qingdaonensis

0.00 334445 334445 S 2859001 Pseudomonas canavaninivorans

0.00 332974 332974 S 1274359 Pseudomonas sihuiensis

0.00 331768 331768 S 2502979 Pseudomonas khazarica

0.00 287136 287136 S 2681983 Pseudomonas bijieensis

0.00 268899 268899 S 2219225 Pseudomonas asiatica

0.00 254336 254336 S 2853159 Pseudomonas pergaminensis

0.00 238975 238975 S 1583341 Pseudomonas cerasi

0.00 237423 237423 S 1306993 Pseudomonas soli

0.00 226740 226740 S 1785145 Pseudomonas glycinae

0.00 213785 213785 S 2894079 Pseudomonas oryziphila

0.00 204952 204952 S 658629 Pseudomonas sessilinigenes

0.00 199510 199510 S 2815720 Pseudomonas germanica

0.00 165939 165939 S 2045200 Pseudomonas rhizophila

0.00 142885 142885 S 2745512 Pseudomonas shahriarae

0.00 105324 105324 S 2745482 Pseudomonas kermanshahensis

0.00 66836 66836 S 470150 Pseudomonas taiwanensis

0.00 3295 3295 S 1880678 Pseudomonas sivasensis

0.07 20292825 941550 G 2901164 Stutzerimonas

0.06 17115123 937867 G1 136846 Stutzerimonas stutzeri group

0.04 12676962 932966 G2 578833 Stutzerimonas stutzeri subgroup

0.04 11220599 9028490 S 316 Stutzerimonas stutzeri

0.00 999505 999505 S1 1123519 Pseudomonas stutzeri DSM 10701

0.00 810061 810061 S1 644801 Pseudomonas stutzeri RCH2

0.00 295663 295663 S1 1196835 Pseudomonas stutzeri CCUG 29243

0.00 84911 84911 S1 996285 Pseudomonas stutzeri DSM 4166

0.00 1969 1969 S1 379731 Pseudomonas stutzeri A1501

0.00 292127 292127 S 1211807 Stutzerimonas kunmingensis

0.00 231270 231270 S 203192 Stutzerimonas chloritidismutans

0.01 1547898 1547898 S 2968969 Stutzerimonas frequens

0.00 1431305 1248976 S 74829 Stutzerimonas balearica

0.00 182329 182329 S1 1123016 Stutzerimonas balearica DSM 6083

0.00 521091 521091 S 2968968 Stutzerimonas degradans

0.00 1255770 1255770 S 1176257 [Pseudomonas] zhaodongensis

0.00 980382 980382 S 271420 Stutzerimonas xanthomarina

0.02 6683649 48384 G 2901189 Halopseudomonas

0.00 1181884 1181884 S 472181 Halopseudomonas sabulinigri

0.00 1136740 1136740 S 1434072 Halopseudomonas salegens

0.00 1132127 1132127 S 797277 Halopseudomonas litoralis

0.00 1108452 0 G1 2901190 unclassified Halopseudomonas

0.00 1108452 1108452 S 2918528 Halopseudomonas sp. RR6

0.00 1051303 0 G1 136844 Halopseudomonas pertucinogena group

0.00 1051303 1051303 S 857252 Halopseudomonas aestusnigri

0.00 1024759 1024759 S 487184 Halopseudomonas xinjiangensis

0.01 4513212 0 F1 351 Azotobacter group

0.01 4513212 351526 G 352 Azotobacter

0.01 1875504 1421122 S 353 Azotobacter chroococcum

0.00 454382 454382 S1 1328314 Azotobacter chroococcum NCIMB 8003

0.00 1235044 1228458 S 354 Azotobacter vinelandii

0.00 2266 2266 S1 322710 Azotobacter vinelandii DJ

0.00 2220 2220 S1 1283330 Azotobacter vinelandii CA

0.00 2100 2100 S1 1283331 Azotobacter vinelandii CA6

0.00 1051138 1051138 S 69964 Azotobacter salinestris

0.01 2017038 3114 G 2758906 Entomomonas

0.00 1022269 1022269 S 2213226 Entomomonas moraniae

0.00 991655 0 G1 2785330 unclassified Entomomonas

0.00 991655 991655 S 2785331 Entomomonas sp. F2A

0.00 1327458 0 G 1654787 Thiopseudomonas

0.00 1327458 1327458 S 1697053 Thiopseudomonas alkaliphila

0.00 1314055 0 G 1649479 Permianibacter

0.00 1314055 1314055 S 1510150 Permianibacter aggregans

0.08 25103019 0 F 2887365 Marinobacteraceae

0.08 25103019 1736720 G 2742 Marinobacter

0.04 13418795 2714912 G1 83889 unclassified Marinobacter

0.00 1428362 1428362 S 2817656 Marinobacter sp. CA1

0.00 1361606 1361606 S 2488665 Marinobacter sp. NP-4(2019)

0.00 1332954 1332954 S 1415568 Marinobacter sp. LV10R510-11A

0.00 1224393 1224393 S 2304594 Marinobacter sp. Arc7-DN-1

0.00 1126372 1126372 S 2769486 Marinobacter sp. LPB0319

0.00 1115651 1115651 S 2547598 Marinobacter sp. JH2

0.00 808880 808880 S 1749259 Marinobacter sp. LQ44

0.00 643890 643890 S 1671721 Marinobacter sp. CP1

0.00 639196 639196 S 490759 Marinobacter sp. BSs20148

0.00 618060 618060 S 1761794 Marinobacter sp. es.042

0.00 391866 391866 S 2917715 Marinobacter sp. M3C

0.00 4898 4898 S 2587869 Marinobacter sp. THAF197a

0.00 2218 2218 S 2587857 Marinobacter sp. THAF39

0.00 2171 2171 S 2917713 Marinobacter sp. M1C

0.00 1834 1834 S 2917716 Marinobacter sp. M4C

0.00 1532 1532 S 2917714 Marinobacter sp. M2C

0.01 1857132 605870 S 2743 Marinobacter nauticus

0.00 690348 690348 S1 351348 Marinobacter nauticus VT8

0.00 560914 560914 S1 1163748 Marinobacter nauticus ATCC 49840

0.01 1850355 1850355 S 1420917 Marinobacter salarius

0.00 1402233 1402233 S 2603215 Marinobacter fonticola

0.00 1238910 1238910 S 1874317 Marinobacter salinus

0.00 1183077 1183077 S 1420916 Marinobacter similis

0.00 1158594 1158594 S 418719 Marinobacter salsuginis

0.00 637146 637146 S 330734 Marinobacter psychrophilus

0.00 620057 552597 S 1033846 Marinobacter adhaerens

0.00 67460 67460 S1 225937 Marinobacter adhaerens HP15

0.81 247970483 419660 O 135622 Alteromonadales

0.30 92811989 21242 F 267890 Shewanellaceae

0.29 90058553 15944110 G 22 Shewanella

0.07 20553611 1895189 G1 196818 unclassified Shewanella

0.01 1574632 1574632 S 2803864 Shewanella sp. KX20019

0.01 1570042 1570042 S 2589987 Shewanella sp. Scap07

0.00 1320107 1320107 S 2950245 Shewanella sp. NFH-SH190041

0.00 1208725 1208725 S 2864212 Shewanella sp. FJAT-51860

0.00 1190005 1190005 S 2590015 Shewanella sp. SNU WT4

0.00 1174717 1174717 S 2864208 Shewanella sp. FJAT-53870

0.00 1160724 1160724 S 2864206 Shewanella sp. FJAT-53749

0.00 1116237 1116237 S 2908650 Shewanella sp. OM3-2

0.00 1000273 1000273 S 2029986 Shewanella sp. WE21

0.00 964234 964234 S 2698686 Shewanella sp. Arc9-LZ

0.00 571840 571840 S 2864204 Shewanella sp. FJAT-53555

0.00 532371 532371 S 60480 Shewanella sp. MR-4

0.00 465789 465789 S 2575361 Shewanella sp. MEBiC00475

0.00 456760 456760 S 2864213 Shewanella sp. FJAT-52072

0.00 429953 429953 S 2864210 Shewanella sp. FJAT-51649

0.00 406642 406642 S 2864211 Shewanella sp. FJAT-51754

0.00 405218 405218 S 2864202 Shewanella sp. FJAT-52076

0.00 394542 394542 S 2864209 Shewanella sp. FJAT-54031

0.00 384403 384403 S 2864205 Shewanella sp. FJAT-53681

0.00 379698 379698 S 2864207 Shewanella sp. FJAT-53764

0.00 377180 377180 S 94122 Shewanella sp. ANA-3

0.00 355382 355382 S 2864203 Shewanella sp. FJAT-53532

0.00 354196 354196 S 2775245 Shewanella sp. WPAGA9

0.00 312974 312974 S 2059264 Shewanella sp. Pdp11

0.00 164608 164608 S 2589790 Shewanella sp. LC6

0.00 159702 159702 S 2806008 Shewanella sp. LZH-2

0.00 121265 121265 S 351745 Shewanella sp. W3-18-1

0.00 100392 100392 S 1930557 Shewanella sp. FDAARGOS_354

0.00 3283 3283 S 2601269 Shewanella sp. YLB-09

0.00 2528 2528 S 592146 Shewanella sp. 33B

0.01 3800508 3178591 S 62322 Shewanella baltica

0.00 232265 232265 S1 407976 Shewanella baltica OS223

0.00 206056 206056 S1 402882 Shewanella baltica OS185

0.00 65302 65302 S1 693970 Shewanella baltica OS117

0.00 49226 49226 S1 399599 Shewanella baltica OS195

0.00 28408 28408 S1 693974 Shewanella baltica BA175

0.00 17863 17863 S1 693973 Shewanella baltica OS678

0.00 14091 14091 S1 693971 Shewanella baltica OS183

0.00 8706 8706 S1 325240 Shewanella baltica OS155

0.01 3383451 3383451 S 38313 Shewanella algae

0.01 2917465 2917465 S 24 Shewanella putrefaciens

0.01 1796381 0 S 60961 Shewanella woodyi

0.01 1796381 1796381 S1 392500 Shewanella woodyi ATCC 51908

0.01 1690712 1690712 S 225848 Shewanella psychrophila

0.01 1665008 0 S 271097 Shewanella sediminis

0.01 1665008 1665008 S1 425104 Shewanella sediminis HAW-EB3

0.01 1607817 1607817 S 260364 Shewanella marisflavi

0.01 1604600 1604600 S 2593655 Shewanella psychropiezotolerans

0.01 1587995 0 S 404011 Shewanella piezotolerans

0.01 1587995 1587995 S1 225849 Shewanella piezotolerans WP3

0.00 1527359 0 S 271098 Shewanella halifaxensis

0.00 1527359 1527359 S1 458817 Shewanella halifaxensis HAW-EB4

0.00 1490904 0 S 70864 Shewanella pealeana

0.00 1490904 1490904 S1 398579 Shewanella pealeana ATCC 700345

0.00 1476777 1476777 S 238836 Shewanella donghaensis

0.00 1437232 1437232 S 2520507 Shewanella maritima

0.00 1363463 1363463 S 1965282 Shewanella khirikhana

0.00 1363216 0 S 60217 Shewanella violacea

0.00 1363216 1363216 S1 637905 Shewanella violacea DSS12

0.00 1352871 0 S 192073 Shewanella denitrificans

0.00 1352871 1352871 S1 318161 Shewanella denitrificans OS217

0.00 1352786 1352786 S 2487742 Shewanella psychromarinicola

0.00 1326190 1326190 S 1738770 Shewanella inventionis

0.00 1321752 1321752 S 1028752 Shewanella aestuarii

0.00 1278661 1278661 S 2814294 Shewanella avicenniae

0.00 1260162 1260162 S 712036 Shewanella dokdonensis

0.00 1257408 1257408 S 518738 Shewanella vesiculosa

0.00 1235670 1235670 S 2590884 Shewanella glacialimarina

0.00 1199915 1199915 S 150120 Shewanella livingstonensis

0.00 1152899 0 S 70863 Shewanella oneidensis

0.00 1152899 1152899 S1 211586 Shewanella oneidensis MR-1

0.00 1140677 1140677 S 2829809 Shewanella yunxiaonensis

0.00 1098070 1098070 S 2814292 Shewanella cyperi

0.00 1036375 1036375 S 43661 Shewanella benthica

0.00 1028824 1028824 S 1173586 Shewanella litorisediminis

0.00 999172 0 S 60478 Shewanella amazonensis

0.00 999172 999172 S1 326297 Shewanella amazonensis SB2B

0.00 995245 995245 S 256839 Shewanella decolorationis

0.00 922893 0 S 56812 Shewanella frigidimarina

0.00 922893 922893 S1 318167 Shewanella frigidimarina NCIMB 400

0.00 908456 908456 S 332186 Shewanella xiamenensis

0.00 648679 648679 S 2814293 Shewanella sedimentimangrovi

0.00 596515 596515 S 1987584 Shewanella carassii

0.00 485494 485494 S 558541 Shewanella chilikensis

0.00 447710 447710 S 2588449 Shewanella polaris

0.00 389874 389874 S 768528 Shewanella indica

0.00 384425 384425 S 755175 Shewanella seohaensis

0.00 384023 0 S 359303 Shewanella loihica

0.00 384023 384023 S1 323850 Shewanella loihica PV-4

0.00 354583 354583 S 93973 Shewanella japonica

0.00 285722 285722 S 2018305 Shewanella bicestrii

0.00 2893 2893 S 2593656 Shewanella eurypsychrophilus

0.01 2732194 1276 G 2547964 Parashewanella

0.00 1446704 1446704 S 342950 Parashewanella spongiae

0.00 1284214 1284214 S 2547970 Parashewanella tropica

0.22 67073511 22476 F 267888 Pseudoalteromonadaceae

0.21 63878502 15529124 G 53246 Pseudoalteromonas

0.04 13140950 1615525 G1 194690 unclassified Pseudoalteromonas

0.01 1641333 1641333 S 1709477 Pseudoalteromonas sp. R3

0.00 1497085 1497085 S 2822842 Pseudoalteromonas sp. SCSIO 43201

0.00 1425984 1425984 S 116092 Pseudoalteromonas sp. A25

0.00 784360 784360 S 2822846 Pseudoalteromonas sp. SCSIO 43088

0.00 626643 626643 S 2003316 Pseudoalteromonas sp. GCY

0.00 573906 573906 S 1390185 Pseudoalteromonas sp. DL-6

0.00 457677 457677 S 2587843 Pseudoalteromonas sp. THAF3

0.00 379061 379061 S 562716 Pseudoalteromonas sp. CF6-2

0.00 364212 364212 S 1720343 Pseudoalteromonas sp. 1_2015MBL_MicDiv

0.00 352512 352512 S 283699 Pseudoalteromonas sp. Bsw20308

0.00 347978 347978 S 2822847 Pseudoalteromonas sp. SCSIO 43101

0.00 332342 332342 S 2894202 Pseudoalteromonas sp. SCSIO 43095

0.00 324386 324386 S 2490635 Pseudoalteromonas sp. Xi13

0.00 246708 246708 S 2799564 Pseudoalteromonas sp. LC2018020214

0.00 242957 242957 S 87791 Pseudoalteromonas sp. PS1M3

0.00 231996 231996 S 1514074 Pseudoalteromonas sp. NC201

0.00 229425 229425 S 649161 Pseudoalteromonas sp. 3J6

0.00 206139 206139 S 2916633 Pseudoalteromonas sp. KAN5

0.00 197042 197042 S 2699396 Pseudoalteromonas sp. APM04

0.00 191128 191128 S 1761891 Pseudoalteromonas sp. 13-15

0.00 176345 176345 S 2692624 Pseudoalteromonas sp. M8

0.00 144379 144379 S 327511 Pseudoalteromonas sp. A22

0.00 124072 124072 S 2839744 Pseudoalteromonas sp. SiA1

0.00 120889 120889 S 2759705 Pseudoalteromonas sp. MT33b

0.00 108773 108773 S 2752475 Pseudoalteromonas sp. JSTW

0.00 99192 99192 S 234831 Pseudoalteromonas sp. SM9913

0.00 60531 60531 S 2785910 Pseudoalteromonas sp. A41-2

0.00 31558 31558 S 747457 Pseudoalteromonas sp. SANK 73390

0.00 1859 1859 S 2589991 Pseudoalteromonas sp. Scap06

0.00 1791 1791 S 2589992 Pseudoalteromonas sp. Scap25

0.00 1772 1772 S 383749 Pseudoalteromonas sp. BSi20327

0.00 1390 1390 S 336179 Pseudoalteromonas sp. 643A

0.01 4392512 4392512 S 43658 Pseudoalteromonas rubra

0.01 4172665 3727008 S 43657 Pseudoalteromonas luteoviolacea

0.00 445657 445657 S1 1365250 Pseudoalteromonas luteoviolacea DSM 6061

0.01 2327403 2327403 S 43662 Pseudoalteromonas piscicida

0.01 2216418 1465806 S 298657 Pseudoalteromonas spongiae

0.00 750612 750612 S1 1117319 Pseudoalteromonas spongiae UST010723-006

0.01 1564385 1066164 S 247523 Pseudoalteromonas aliena

0.00 498221 498221 S1 1314866 Pseudoalteromonas aliena SW19

0.00 1527061 1527061 S 339617 Pseudoalteromonas viridis

0.00 1478523 1478523 S 314281 Pseudoalteromonas tunicata

0.00 1476510 1473431 S 161398 Pseudoalteromonas phenolica

0.00 3079 3079 S1 1315281 Pseudoalteromonas phenolica O-BC30

0.00 1453573 1453573 S 2518973 Pseudoalteromonas rhizosphaerae

0.00 1420250 0 S 107327 Pseudoalteromonas ulvae

0.00 1420250 1420250 S1 1315306 Pseudoalteromonas ulvae UL12

0.00 1399366 1399366 S 882626 Pseudoalteromonas xiamenensis

0.00 1394805 0 S 182141 Pseudoalteromonas prydzensis

0.00 1394805 1394805 S1 1315282 Pseudoalteromonas prydzensis ACAM 620

0.00 1368757 1368757 S 1348114 Pseudoalteromonas piratica

0.00 1043871 0 S 28107 Pseudoalteromonas espejiana

0.00 1043871 1043871 S1 1314869 Pseudoalteromonas espejiana DSM 9414

0.00 1035522 1026442 S 227 Pseudoalteromonas carrageenovora

0.00 9080 9080 S1 1314868 Pseudoalteromonas carrageenovora IAM 12662

0.00 961295 961295 S 1190813 Pseudoalteromonas shioyasakiensis

0.00 893268 247285 S 166935 Pseudoalteromonas translucida

0.00 632917 632917 S1 1315283 Pseudoalteromonas translucida KMM 520

0.00 13066 13066 S1 326442 Pseudoalteromonas translucida TAC125

0.00 748532 0 S 394751 Pseudoalteromonas arctica

0.00 748532 748532 S1 1117313 Pseudoalteromonas arctica A 37-1-2

0.00 633116 371772 S 176102 Pseudoalteromonas agarivorans

0.00 261344 261344 S1 1312369 Pseudoalteromonas agarivorans DSM 14585

0.00 555521 315045 S 77608 Pseudoalteromonas distincta

0.00 240476 240476 S1 1315279 Pseudoalteromonas paragorgicola KMM 3548

0.00 529360 0 S 206042 Pseudoalteromonas mariniglutinosa

0.00 529360 529360 S1 1315277 Pseudoalteromonas mariniglutinosa NCIMB 1770

0.00 474880 474880 S 151081 Pseudoalteromonas ruthenica

0.00 431979 431979 S 267375 Pseudoalteromonas marina

0.00 369045 369045 S 43660 Pseudoalteromonas undina

0.00 242497 242497 S 28109 Pseudoalteromonas nigrifaciens

0.00 188158 188158 S 288 Pseudoalteromonas atlantica

0.00 175465 0 S 570156 Pseudoalteromonas lipolytica

0.00 175465 175465 S1 1315275 Pseudoalteromonas lipolytica LMEB 39

0.00 161956 161956 S 621376 Pseudoalteromonas donghaensis

0.00 160436 160436 S 137779 Pseudoalteromonas flavipulchra

0.00 149544 149544 S 874454 Pseudoalteromonas arabiensis

0.00 145640 145640 S 152297 Pseudoalteromonas issachenkonii

0.00 116115 116115 S 43659 Pseudoalteromonas tetraodonis

0.01 1950746 4336 G 907197 Psychrosphaera

0.00 1017473 1017473 S 2820710 Psychrosphaera ytuae

0.00 928937 928937 S 1266052 Psychrosphaera aestuarii

0.00 1221787 0 G 2834759 Flocculibacter

0.00 1221787 1221787 S 2744479 Flocculibacter collagenilyticus

0.17 52728936 29982 F 72275 Alteromonadaceae

0.09 28841789 54012 F1 2903219 Alteromonas/Salinimonas group

0.08 23735792 3920651 G 226 Alteromonas

0.02 7306796 2325247 G1 2614992 unclassified Alteromonas

0.01 1551273 1551273 S 2267264 Alteromonas sp. RKMC-009

0.00 1080328 1080328 S 1714845 Alteromonas sp. BL110

0.00 830799 830799 S 2358187 Alteromonas sp. 76-1

0.00 781626 781626 S 2961948 Alteromonas sp. LMIT006

0.00 283678 283678 S 2058133 Alteromonas sp. MB-3u-76

0.00 233922 233922 S 1917158 Alteromonas sp. RW2A1

0.00 153390 153390 S 2785913 Alteromonas sp. B31-7

0.00 58317 58317 S 1777491 Alteromonas sp. Mac1

0.00 4541 4541 S 2795688 Alteromonas sp. KC3

0.00 3675 3675 S 2795689 Alteromonas sp. KC14

0.02 5859565 4279047 S 28108 Alteromonas macleodii

0.00 910929 910929 S1 1004785 Alteromonas macleodii str. 'Black Sea 11'

0.00 355962 355962 S1 1004787 Alteromonas macleodii str. 'Balearic Sea AD45'

0.00 157611 157611 S1 1004788 Alteromonas macleodii str. 'English Channel 673'

0.00 156016 156016 S1 529120 Alteromonas macleodii ATCC 27126

0.01 3525966 3175181 S 314275 Alteromonas mediterranea

0.00 166528 166528 S1 1774373 Alteromonas mediterranea DE

0.00 149795 149795 S1 1300254 Alteromonas mediterranea MED64

0.00 14231 14231 S1 1300256 Alteromonas mediterranea U7

0.00 7613 7613 S1 1300255 Alteromonas mediterranea U4

0.00 5285 5285 S1 1004786 Alteromonas mediterranea DE1

0.00 4249 4249 S1 1300259 Alteromonas mediterranea UM4b

0.00 3084 3084 S1 1300258 Alteromonas mediterranea UM7

0.00 1328111 1328111 S 1858656 Alteromonas pelagimontana

0.00 777553 777553 S 715451 Alteromonas naphthalenivorans

0.00 548863 475986 S 233316 Alteromonas stellipolaris

0.00 72877 72877 S1 1160720 Alteromonas stellipolaris LMG 21856

0.00 468287 468287 S 589873 Alteromonas australica

0.02 5051985 24077 G 288793 Salinimonas

0.00 1337502 1337502 S 2303538 Salinimonas sediminis

0.00 1263439 1263439 S 914153 Salinimonas lutimaris

0.00 1262874 1262874 S 2572577 Salinimonas iocasae

0.00 1164093 1164093 S 2785918 Salinimonas marina

0.02 6440146 19051 G 1621534 Paraglaciecola

0.01 3179715 717 G1 2685791 unclassified Paraglaciecola

0.01 1633215 1633215 S 2686358 Paraglaciecola sp. L3A3

0.01 1545783 1545783 S 2686359 Paraglaciecola sp. L1A13

0.01 1674982 0 S 326544 Paraglaciecola psychrophila

0.01 1674982 1674982 S1 1129794 Paraglaciecola psychrophila 170

0.01 1566398 1566398 S 197222 Paraglaciecola mesophila

0.02 6117172 982191 G 261825 Agarivorans

0.01 2180006 1552 G1 2636026 unclassified Agarivorans

0.00 1405318 1405318 S 2937286 Agarivorans sp. TSD2052

0.00 773136 773136 S 2652721 Agarivorans sp. B2Z047

0.00 1334371 1334371 S 680279 Agarivorans gilvus

0.00 989225 989225 S 1563703 Agarivorans aestuarii

0.00 631379 631379 S 182262 Agarivorans albus

0.01 1787426 0 G 2894574 Saccharobesus

0.01 1787426 1787426 S 2172099 Saccharobesus litoralis

0.01 1535151 0 G 2905123 Paraneptunicella

0.01 1535151 1535151 S 2831148 Paraneptunicella aestuarii

0.00 1494425 0 G 1858563 Alkalimarinus

0.00 1494425 1487330 G1 2625403 unclassified Alkalimarinus

0.00 2394 2394 S 2936273 Alkalimarinus sp. SCSIO 12817

0.00 2379 2379 S 2935863 Alkalimarinus sp. SCSIO 12582

0.00 2322 2322 S 2936274 Alkalimarinus sp. SCSIO 12638

0.00 1476604 0 G 1172191 Catenovulum

0.00 1476604 1476604 S 1740262 Catenovulum sediminis

0.00 1379985 0 G 1751872 Lacimicrobium

0.00 1379985 1379985 S 1526571 Lacimicrobium alkaliphilum

0.00 1322666 0 G 2650549 Hydrocarboniclastica

0.00 1322666 1322666 S 2259620 Hydrocarboniclastica marina

0.00 1295016 0 G 89404 Glaciecola

0.00 1295016 0 S 300231 Glaciecola nitratireducens

0.00 1295016 1295016 S1 1085623 Glaciecola nitratireducens FR1064

0.00 1008574 0 G 2661818 Saliniradius

0.00 1008574 1008574 S 2183582 Saliniradius amylolyticus

0.06 17069633 111153 F 267889 Colwelliaceae

0.03 9667998 23526 G 28228 Colwellia

0.03 8029375 71437 G1 196834 unclassified Colwellia

0.00 1388728 1388728 S 1816218 Colwellia sp. PAMC 20917

0.00 1360745 1360745 S 2497879 Colwellia sp. Arc7-635

0.00 1347997 1347997 S 2689569 Colwellia sp. 20A7

0.00 1345972 1345972 S 2583805 Colwellia sp. M166

0.00 1283055 1283055 S 58049 Colwellia sp. MT41

0.00 1231441 1231441 S 2161872 Colwellia sp. Arc7-D

0.01 1615097 0 S 28229 Colwellia psychrerythraea

0.01 1615097 1615097 S1 167879 Colwellia psychrerythraea 34H

0.02 4676198 2919 G 1518149 Thalassotalea

0.01 3488825 1673 G1 2614972 unclassified Thalassotalea

0.00 1248968 1248968 S 2552945 Thalassotalea sp. HSM 43

0.00 1172976 1172976 S 2594005 Thalassotalea sp. PS06

0.00 1065208 1065208 S 2769490 Thalassotalea sp. LPB0316

0.00 1184454 1184454 S 1763536 Thalassotalea crassostreae

0.00 1327311 0 G 2848171 Cognaticolwellia

0.00 1327311 1327311 S 1967665 Cognaticolwellia beringensis

0.00 1286973 0 G 1407056 Litorilituus

0.00 1286973 1286973 S 718192 Litorilituus sediminis

0.02 6465466 0 F 267891 Moritellaceae

0.02 6465466 171319 G 58050 Moritella

0.01 3724258 1275589 G1 2637987 unclassified Moritella

0.00 1336061 1336061 S 2746230 Moritella sp. 24

0.00 668418 668418 S 2746231 Moritella sp. 5

0.00 223769 223769 S 2746233 Moritella sp. 36

0.00 220421 220421 S 2746232 Moritella sp. 28

0.00 1353644 0 S 90736 Moritella marina

0.00 1353644 1353644 S1 1202962 Moritella marina ATCC 15381

0.00 1216245 1216245 S 69539 Moritella yayanosii

0.02 5075113 0 F 267893 Idiomarinaceae

0.02 5075113 330971 G 135575 Idiomarina

0.01 2029440 2026592 S 135577 Idiomarina loihiensis

0.00 1457 1457 S1 1321370 Idiomarina loihiensis GSL 199

0.00 1391 1391 S1 283942 Idiomarina loihiensis L2TR

0.00 1417323 2662 G1 2614829 unclassified Idiomarina

0.00 873665 873665 S 2100422 Idiomarina sp. OT37-5b

0.00 540996 540996 S 2055892 Idiomarina sp. X4

0.00 765492 765492 S 86102 Idiomarina abyssalis

0.00 531887 531887 S 1096243 Idiomarina piscisalsi

0.01 4072839 0 F 267892 Ferrimonadaceae

0.01 4072839 3087 G 44011 Ferrimonas

0.00 1483037 0 G1 2620587 unclassified Ferrimonas

0.00 1483037 1483037 S 2822844 Ferrimonas sp. SCSIO 43195

0.00 1313325 0 S 44012 Ferrimonas balearica

0.00 1313325 1313325 S1 550540 Ferrimonas balearica DSM 9799

0.00 1273390 1273390 S 2724191 Ferrimonas lipolytica

0.01 2253336 0 F 267894 Psychromonadaceae

0.01 2253336 1485 G 67572 Psychromonas

0.00 1351404 0 S 357794 Psychromonas ingrahamii

0.00 1351404 1351404 S1 357804 Psychromonas ingrahamii 37

0.00 900447 0 G1 2614957 unclassified Psychromonas

0.00 900447 900447 S 314282 Psychromonas sp. CNPT3

0.74 225622064 0 O 135623 Vibrionales

0.74 225622064 648541 F 641 Vibrionaceae

0.62 189360015 35531002 G 662 Vibrio

0.09 26681606 1899529 G1 717610 Vibrio harveyi group

0.03 8443546 8206216 S 670 Vibrio parahaemolyticus

0.00 76576 0 S1 1338033 Vibrio parahaemolyticus O1:Kuk

0.00 76576 76576 S2 1338034 Vibrio parahaemolyticus O1:Kuk str. FDA_R31

0.00 65685 65685 S1 1429044 Vibrio parahaemolyticus UCM-V493

0.00 61381 61381 S1 1211705 Vibrio parahaemolyticus BB22OP

0.00 30334 0 S1 1338031 Vibrio parahaemolyticus O1:K33

0.00 30334 30334 S2 1338032 Vibrio parahaemolyticus O1:K33 str. CDC_K4557

0.00 1830 1830 S1 223926 Vibrio parahaemolyticus RIMD 2210633

0.00 1401 1401 S1 745023 Vibrio parahaemolyticus 10329

0.00 123 123 S1 1300351 Vibrio parahaemolyticus v110

0.01 3968587 3543776 S 680 Vibrio campbellii

0.00 277846 277846 S1 2902295 Vibrio campbellii ATCC BAA-1116

0.00 146965 146965 S1 1224742 Vibrio campbellii CAIM 519 = NBRC 15631 = ATCC 25920

0.01 2644555 2640944 S 663 Vibrio alginolyticus

0.00 3611 3611 S1 1219076 Vibrio alginolyticus NBRC 15630 = ATCC 17749

0.01 2245999 715647 G2 2315253 Vibrio diabolicus subgroup

0.00 1418786 1418786 S 50719 Vibrio diabolicus

0.00 111566 111566 S 150340 Vibrio antiquarius

0.01 1787815 1787815 S 696485 Vibrio owensii

0.01 1559845 1559845 S 669 Vibrio harveyi

0.00 1525073 1519887 S 691 Vibrio natriegens

0.00 5186 5186 S1 1219067 Vibrio natriegens NBRC 15636 = ATCC 14048 = DSM 759

0.00 1381952 1381952 S 512649 Vibrio azureus

0.00 927868 927868 S 190895 Vibrio rotiferianus

0.00 296837 0 S 766224 Vibrio jasicida

0.00 296837 296837 S1 1280002 Vibrio jasicida 090810c

0.06 18019311 469757 G1 2614977 unclassified Vibrio

0.01 1783022 1783022 S 2751178 Vibrio sp. B1FLJ16

0.01 1589561 1589561 S 2785746 Vibrio sp. VB16

0.00 1480337 1480337 S 2819101 Vibrio sp. SCSIO 43136

0.00 1446237 1446237 S 2819096 Vibrio sp. SCSIO 43135

0.00 1339069 1339069 S 2841925 Vibrio sp. OG9-811

0.00 1331448 1331448 S 2587865 Vibrio sp. THAF190c

0.00 1288343 1288343 S 2908646 Vibrio sp. SS-MA-C1-2

0.00 1016947 1016947 S 2819100 Vibrio sp. SCSIO 43140

0.00 848649 848649 S 2714948 Vibrio sp. HDW18

0.00 656410 656410 S 2779363 Vibrio sp. SCSIO 43132

0.00 587593 587593 S 2785124 Vibrio sp. ED004

0.00 572232 572232 S 2589990 Vibrio sp. Scap24

0.00 494766 494766 S 2751179 Vibrio sp. B1REV9

0.00 432929 432929 S 2751176 Vibrio sp. B1ASS3

0.00 402887 402887 S 2942997 Vibrio sp. J383

0.00 367735 367735 S 2819098 Vibrio sp. SCSIO 43153

0.00 340989 340989 S 2751177 Vibrio sp. B1FIG11

0.00 314939 314939 S 2785123 Vibrio sp. ED002

0.00 284974 284974 S 2802577 Vibrio sp. SCSIO 43133

0.00 247778 247778 S 2163016 Vibrio sp. dhg

0.00 244712 244712 S 1116375 Vibrio sp. EJY3

0.00 170272 170272 S 2819099 Vibrio sp. SCSIO 43155

0.00 80976 80976 S 2883075 Vibrio sp. LQ2

0.00 53971 53971 S 2766781 Vibrio sp. sp1

0.00 53019 53019 S 2819104 Vibrio sp. SCSIO 43097

0.00 32991 32991 S 2819103 Vibrio sp. SCSIO 43009

0.00 27109 27109 S 678 Vibrio sp.

0.00 16440 16440 S 452803 Vibrio sp. 23023

0.00 8488 8488 S 452804 Vibrio sp. 09022

0.00 7858 7858 S 2978741 Vibrio sp. J502

0.00 6887 6887 S 452802 Vibrio sp. 0908

0.00 3454 3454 S 2661922 Vibrio sp. THAF191d

0.00 3040 3040 S 2661921 Vibrio sp. THAF191c

0.00 2733 2733 S 2819097 Vibrio sp. SCSIO 43145

0.00 2698 2698 S 2822843 Vibrio sp. SCSIO 43186

0.00 2411 2411 S 2819102 Vibrio sp. SCSIO 43139

0.00 2266 2266 S 2587860 Vibrio sp. THAF64

0.00 2092 2092 S 65384 Vibrio sp. 41

0.00 1050 1050 S 397884 Vibrio sp. 04Ya090

0.00 215 215 S 864336 Vibrio sp. 04Ya108

0.00 27 27 S 203842 Vibrio sp. TC68

0.02 6305358 5847023 S 672 Vibrio vulnificus

0.00 146734 146734 S1 748003 Vibrio vulnificus VVyb1(BT3)

0.00 129029 129029 S1 196600 Vibrio vulnificus YJ016

0.00 89580 89580 S1 1246305 Vibrio vulnificus Env1

0.00 86019 86019 S1 914127 Vibrio vulnificus MO6-24/O

0.00 3581 3581 S1 1219061 Vibrio vulnificus NBRC 15645 = ATCC 27562

0.00 3392 3392 S1 216895 Vibrio vulnificus CMCP6

0.01 4431339 4358354 S 666 Vibrio cholerae

0.00 22401 4556 S1 127906 Vibrio cholerae O1

0.00 9512 7705 S2 686 Vibrio cholerae O1 biovar El Tor

0.00 1807 1807 S3 243277 Vibrio cholerae O1 biovar El Tor str. N16961

0.00 2458 2458 S2 593588 Vibrio cholerae MJ-1236

0.00 2312 2312 S2 1134456 Vibrio cholerae IEC224

0.00 1626 1626 S2 1224154 Vibrio cholerae O1 str. Inaba G4222

0.00 1168 1168 S2 914149 Vibrio cholerae O1 str. 2010EL-1786

0.00 769 769 S2 1433144 Vibrio cholerae O1 str. KW3

0.00 17381 17381 S1 345073 Vibrio cholerae O395

0.00 11707 11707 S1 185332 Vibrio cholerae O37

0.00 8104 8104 S1 1420885 Vibrio cholerae MS6

0.00 3330 3330 S1 948564 Vibrio cholerae C6706

0.00 2995 2995 S1 156539 Vibrio cholerae non-O1/non-O139

0.00 2615 2615 S1 579112 Vibrio cholerae M66-2

0.00 1777 1777 S1 345072 Vibrio cholerae MO10

0.00 1612 1612 S1 412614 Vibrio cholerae 2740-80

0.00 959 959 S1 1093790 Vibrio cholerae H1

0.00 104 104 S1 45888 Vibrio cholerae O139

0.01 4073744 464715 G1 1891919 Vibrio oreintalis group

0.00 1288358 1288358 S 300876 Vibrio europaeus

0.00 1198159 1198159 S 379097 Vibrio sinaloensis

0.00 1122512 0 S 29498 Vibrio tubiashii

0.00 1122512 1122512 S1 1051646 Vibrio tubiashii ATCC 19109

0.01 3113620 3113620 S 674 Vibrio mimicus

0.01 2956201 2823539 S 29494 Vibrio furnissii

0.00 132662 132662 S1 903510 Vibrio furnissii NCTC 11218

0.01 2935731 2652175 S 190893 Vibrio coralliilyticus

0.00 283556 283556 S1 1384040 Vibrio coralliilyticus OCN008

0.01 2784287 2784287 S 676 Vibrio fluvialis

0.01 2161915 2161915 S 170679 Vibrio chagasii

0.01 2053692 2053692 S 47951 Vibrio cyclitrophicus

0.01 2045257 2045257 S 104609 Vibrio penaeicida

0.01 2027063 2027063 S 687 Vibrio gazogenes

0.01 1968293 1968293 S 29497 Vibrio splendidus

0.01 1919412 1919412 S 45658 Vibrio scophthalmi

0.01 1854306 1854306 S 28173 Vibrio nigripulchritudo

0.01 1804531 1804531 S 553239 Vibrio breoganii

0.01 1766341 1303155 S 246167 Vibrio crassostreae

0.00 463186 463186 S1 1191300 Vibrio crassostreae 9CS106

0.01 1714842 1714842 S 1117707 Vibrio quintilis

0.01 1679686 1679686 S 29495 Vibrio navarrensis

0.01 1640796 24158 S 52443 Vibrio tapetis

0.01 1616638 1616638 S1 1671868 Vibrio tapetis subsp. tapetis

0.01 1639416 1639416 S 2572923 Vibrio taketomensis

0.01 1611973 1592020 S 55601 Vibrio anguillarum

0.00 17508 1094 S1 42323 Vibrio anguillarum serovar O1

0.00 12569 12569 S2 882944 Vibrio anguillarum M3

0.00 3845 3845 S2 882102 Vibrio anguillarum 775

0.00 2445 2445 S1 105260 Vibrio anguillarum serovar O2

0.01 1586684 1586684 S 92172 Vibrio aerogenes

0.01 1583795 1583795 S 1481663 Vibrio metoecus

0.01 1538180 1538180 S 1074311 Vibrio alfacsensis

0.01 1536267 0 S 435912 Vibrio porteresiae

0.01 1536267 1536267 S1 1123496 Vibrio porteresiae DSM 19223

0.00 1518120 1518120 S 28169 Vibrio pelagius

0.00 1497873 1497873 S 474394 Vibrio mangrovi

0.00 1450622 1450622 S 1481914 Vibrio ishigakensis

0.00 1441462 1441462 S 1435069 Vibrio tritonius

0.00 1437035 1437035 S 1481923 Vibrio astriarenae

0.00 1401794 1401794 S 552386 Vibrio gallaecicus

0.00 1394877 1394877 S 1381081 Vibrio panuliri

0.00 1364994 1364994 S 1918945 Vibrio spartinae

0.00 1358282 1358282 S 675 Vibrio cincinnatiensis

0.00 1357614 1357614 S 28172 Vibrio metschnikovii

0.00 1351966 1351966 S 2587862 Vibrio aquimaris

0.00 1351091 1351091 S 265668 Vibrio ponticus

0.00 1325346 1325346 S 1328770 Vibrio cortegadensis

0.00 1288997 1288997 S 170651 Vibrio neptunius

0.00 1288889 1288889 S 2711221 Vibrio ziniensis

0.00 1282469 1282469 S 212667 Vibrio fortis

0.00 1264996 1264996 S 296199 Vibrio gigantis

0.00 1256010 1256010 S 689 Vibrio mediterranei

0.00 1217873 1217873 S 664643 Vibrio plantisponsor

0.00 1182847 1182847 S 1824638 Vibrio japonicus

0.00 1171283 1171283 S 398736 Vibrio rhizosphaerae

0.00 1160946 1160946 S 184755 Vibrio ruber

0.00 1145011 1145011 S 673372 Vibrio casei

0.00 1124776 1124776 S 76258 Vibrio rumoiensis

0.00 1123690 1123690 S 413403 Vibrio rarus

0.00 1119820 1119820 S 278860 Vibrio neonatus

0.00 1093844 1093844 S 1918946 Vibrio palustris

0.00 1091532 1091532 S 2479546 Vibrio zhugei

0.00 1040388 1040388 S 990268 Vibrio maritimus

0.00 1028243 1028243 S 190897 Vibrio gallicus

0.00 1015914 1015914 S 2662262 Vibrio algicola

0.00 990824 990824 S 1891186 Vibrio aphrogenes

0.00 964994 964994 S 446374 Vibrio artabrorum

0.00 947088 947088 S 2100116 Vibrio echinoideorum

0.00 931921 931921 S 693153 Vibrio atlanticus

0.00 904876 904876 S 170673 Vibrio kanaloae

0.00 861010 861010 S 2777577 Vibrio bathopelagicus

0.00 802510 802510 S 136468 Vibrio lentus

0.00 741912 741912 S 1534743 Vibrio hyugaensis

0.00 618121 618121 S 198832 Vibrio pomeroyi

0.00 614996 614996 S 1763883 Vibrio cidicii

0.00 530770 530770 S 1516159 Vibrio coralliirubri

0.00 526389 526389 S 1194427 Vibrio toranzoniae

0.00 507824 507824 S 212663 Vibrio tasmaniensis

0.00 484750 484750 S 446372 Vibrio celticus

0.00 460309 460309 S 2025808 Vibrio qinghaiensis

0.00 150530 150530 S 2014742 Vibrio tarriae

0.00 145426 145426 S 650003 Vibrio paracholerae

0.00 62513 62513 S 1208308 Vibrio neocaledonicus

0.07 22569605 304109 G 657 Photobacterium

0.02 6072173 58523 G1 2628852 unclassified Photobacterium

0.01 1558805 1558805 S 2899122 Photobacterium sp. TY1-4

0.01 1548444 1548444 S 2829502 Photobacterium sp. GJ3

0.01 1529876 1529876 S 2912855 Photobacterium sp. CCB-ST2H9

0.00 1376525 1376525 S 2907534 Photobacterium sp. TLY01

0.02 4587476 2565046 S 38293 Photobacterium damselae

0.01 1783375 1783375 S1 85581 Photobacterium damselae subsp. damselae

0.00 239055 221912 S1 38294 Photobacterium damselae subsp. piscicida

0.00 17143 17143 S2 1200302 Photobacterium damselae subsp. piscicida DI21

0.01 1828205 0 S 74109 Photobacterium profundum

0.01 1828205 1828205 S1 298386 Photobacterium profundum SS9

0.01 1783882 0 S 1295392 Photobacterium gaetbulicola

0.01 1783882 1783882 S1 658445 Photobacterium gaetbulicola Gung47

0.01 1687983 1687983 S 1935446 Photobacterium toruni

0.01 1686001 1686001 S 320778 Photobacterium ganghwense

0.01 1580653 1580653 S 680026 Photobacterium swingsii

0.01 1545919 1545919 S 875932 Photobacterium sanguinicancri

0.00 1491947 1491947 S 865757 Photobacterium atrarenae

0.00 1257 1257 S 659 Photobacterium phosphoreum

0.01 4300215 28117 G 511678 Aliivibrio

0.01 2908490 1636463 S 668 Aliivibrio fischeri

0.00 517984 517984 S1 312309 Aliivibrio fischeri ES114

0.00 377810 377810 S1 1088719 Aliivibrio fischeri SR5

0.00 376233 376233 S1 388396 Aliivibrio fischeri MJ11

0.00 1299642 1231452 S 40269 Aliivibrio salmonicida

0.00 68190 68190 S1 316275 Aliivibrio salmonicida LFI1238

0.00 63966 63966 S 80852 Aliivibrio wodanis

0.01 3276455 20326 G 246861 Grimontia

0.01 1667173 0 G1 2644349 unclassified Grimontia

0.01 1667173 1667173 S 2872157 Grimontia sp. 020920N

0.01 1588956 1588956 S 673 Grimontia hollisae

0.01 2434181 775877 G 51366 Salinivibrio

0.00 988170 988170 S 1908198 Salinivibrio kushneri

0.00 345147 0 G1 2636825 unclassified Salinivibrio

0.00 345147 345147 S 2003370 Salinivibrio sp. YCSC6

0.00 324987 324987 S 51367 Salinivibrio costicola

0.01 1682110 0 G 2014233 Thaumasiovibrio

0.01 1682110 1682110 S 1891207 Thaumasiovibrio subtropicus

0.00 705007 0 G 2042066 Paraphotobacterium

0.00 705007 705007 S 1755811 Paraphotobacterium marinum

0.00 645935 0 G 188143 Enterovibrio

0.00 645935 645935 S 1927128 Candidatus Enterovibrio luxaltus

0.59 181146302 344371 O 135614 Xanthomonadales

0.49 149037051 2070294 F 32033 Xanthomonadaceae

0.16 47747595 9970086 G 338 Xanthomonas

0.01 4425232 1954097 S 339 Xanthomonas campestris

0.00 824510 699184 S1 340 Xanthomonas campestris pv. campestris

0.00 25418 25418 S2 1358000 Xanthomonas campestris pv. campestris str. CFBP 4954

0.00 17940 17940 S2 1358001 Xanthomonas campestris pv. campestris str. CFBP 4955

0.00 17563 17563 S2 1358004 Xanthomonas campestris pv. campestris str. CN03

0.00 8133 8133 S2 1358002 Xanthomonas campestris pv. campestris str. CFBP 5130

0.00 7153 7153 S2 1357996 Xanthomonas campestris pv. campestris str. CFBP 1124

0.00 7095 7095 S2 1358009 Xanthomonas campestris pv. campestris str. CFBP 5817

0.00 5650 5650 S2 190485 Xanthomonas campestris pv. campestris str. ATCC 33913

0.00 4999 4999 S2 1358019 Xanthomonas campestris pv. campestris str. CN18

0.00 4631 4631 S2 1281284 Xanthomonas campestris pv. campestris str. CN16

0.00 3521 3521 S2 314565 Xanthomonas campestris pv. campestris str. 8004

0.00 2709 2709 S2 1281282 Xanthomonas campestris pv. campestris str. CN14

0.00 2645 2645 S2 1358015 Xanthomonas campestris pv. campestris str. CN17

0.00 2407 2407 S2 1281283 Xanthomonas campestris pv. campestris str. CN15

0.00 2335 2335 S2 1357995 Xanthomonas campestris pv. campestris str. 147

0.00 2145 2145 S2 1358014 Xanthomonas campestris pv. campestris str. CN10

0.00 2057 2057 S2 1358017 Xanthomonas campestris pv. campestris str. CN12

0.00 1982 1982 S2 1358010 Xanthomonas campestris pv. campestris str. CN01

0.00 1916 1916 S2 1357998 Xanthomonas campestris pv. campestris str. CFBP 1712

0.00 1768 1768 S2 1358003 Xanthomonas campestris pv. campestris str. CFBP 5683

0.00 1691 1691 S2 1357999 Xanthomonas campestris pv. campestris str. CFBP 1869

0.00 1568 1568 S2 1358008 Xanthomonas campestris pv. campestris str. CFBP 1713

0.00 813814 813814 S1 149696 Xanthomonas campestris pv. badrii

0.00 453623 269191 S1 359385 Xanthomonas campestris pv. raphani

0.00 184432 184432 S2 990315 Xanthomonas campestris pv. raphani 756C

0.00 379188 379188 S1 92826 Xanthomonas campestris pv. incanae

0.01 3943168 2415405 S 343 Xanthomonas translucens

0.00 229613 229613 S1 487909 Xanthomonas translucens pv. undulosa

0.00 221517 221517 S1 152263 Xanthomonas translucens pv. cerealis

0.00 218912 218912 S1 227946 Xanthomonas translucens pv. poae

0.00 196129 196129 S1 320326 Xanthomonas translucens pv. arrhenatheri

0.00 194015 194015 S1 134874 Xanthomonas translucens pv. graminis

0.00 184753 172120 S1 134875 Xanthomonas translucens pv. translucens

0.00 12633 12633 S2 1261556 Xanthomonas translucens pv. translucens DSM 18974

0.00 116115 116115 S1 487907 Xanthomonas translucens pv. phleipratensis

0.00 85415 85415 S1 487906 Xanthomonas translucens pv. phlei

0.00 58858 58858 S1 627605 Xanthomonas translucens pv. pistacia

0.00 12337 12337 S1 487908 Xanthomonas translucens pv. secalis

0.00 10099 10099 S1 1099789 Xanthomonas translucens pv. hordei

0.01 3317565 2199417 S 347 Xanthomonas oryzae

0.00 660774 655251 S1 64187 Xanthomonas oryzae pv. oryzae

0.00 3795 3795 S2 342109 Xanthomonas oryzae pv. oryzae MAFF 311018

0.00 933 933 S2 360094 Xanthomonas oryzae pv. oryzae PXO99A

0.00 795 795 S2 1458476 Xanthomonas oryzae pv. oryzae PXO86

0.00 457374 453155 S1 129394 Xanthomonas oryzae pv. oryzicola

0.00 4219 4219 S2 383407 Xanthomonas oryzae pv. oryzicola BLS256

0.01 3146615 2117962 S 56448 Xanthomonas arboricola

0.00 714292 714292 S1 195709 Xanthomonas arboricola pv. juglandis

0.00 159458 159436 S1 69929 Xanthomonas arboricola pv. pruni

0.00 22 22 S2 1045865 Xanthomonas arboricola pv. pruni str. CFBP 5530

0.00 154903 154903 S1 487821 Xanthomonas arboricola pv. corylina

0.01 3105174 0 G1 643453 Xanthomonas citri group

0.01 3105174 1398089 S 346 Xanthomonas citri

0.00 401671 401671 S1 473426 Xanthomonas citri pv. vignicola

0.00 241882 237479 S1 611301 Xanthomonas citri pv. citri

0.00 1617 1617 S2 1137651 Xanthomonas citri subsp. citri Aw12879

0.00 1085 1085 S2 1308548 Xanthomonas citri subsp. citri UI6

0.00 911 911 S2 1308541 Xanthomonas citri subsp. citri A306

0.00 790 790 S2 190486 Xanthomonas citri pv. citri str. 306

0.00 224354 202292 S1 473421 Xanthomonas citri pv. glycines

0.00 16814 16814 S2 1150615 Xanthomonas citri pv. glycines str. 12-2

0.00 2549 2549 S2 1365648 Xanthomonas citri pv. glycines CFBP 2526

0.00 1363 1363 S2 1401257 Xanthomonas citri pv. glycines str. 8ra

0.00 1336 1336 S2 1365649 Xanthomonas citri pv. glycines CFBP 7119

0.00 188388 181801 S1 86040 Xanthomonas citri pv. malvacearum

0.00 4988 4988 S2 1220028 Xanthomonas citri pv. malvacearum X20

0.00 1599 1599 S2 1220027 Xanthomonas citri pv. malvacearum X18

0.00 169299 169299 S1 76802 Xanthomonas citri pv. aurantifolii

0.00 127866 127866 S1 487838 Xanthomonas citri pv. punicae

0.00 113349 113349 S1 454594 Xanthomonas citri pv. mangiferaeindicae

0.00 79896 79896 S1 473423 Xanthomonas citri pv. phaseoli var. fuscans

0.00 72464 0 S1 454595 Xanthomonas citri pv. anacardii

0.00 72464 72464 S2 1437881 Xanthomonas citri pv. anacardii CFBP 2913

0.00 62167 61062 S1 366649 Xanthomonas citri pv. fuscans

0.00 1105 1105 S2 1365651 Xanthomonas citri pv. fuscans CFBP 6988

0.00 25749 25749 S1 487862 Xanthomonas citri pv. durantae

0.01 2295875 1375833 S 56454 Xanthomonas hortorum

0.00 447636 447636 S1 453602 Xanthomonas hortorum pv. pelargonii

0.00 254868 254868 S1 2754056 Xanthomonas hortorum pv. gardneri

0.00 136802 0 S1 487904 Xanthomonas hortorum pv. carotae

0.00 136802 136802 S2 863365 Xanthomonas hortorum pv. carotae str. M081

0.00 80736 80736 S1 707209 Xanthomonas hortorum pv. vitians

0.01 1973908 1973908 S 56458 Xanthomonas sacchari

0.01 1862606 571378 G1 2643310 unclassified Xanthomonas

0.00 403369 403369 S 2724121 Xanthomonas sp. GW

0.00 350555 350555 S 2724123 Xanthomonas sp. SI

0.00 338831 338831 S 2724122 Xanthomonas sp. SS

0.00 187244 187244 S 2081477 Xanthomonas sp. MLO165

0.00 8720 8720 S 2750648 Xanthomonas sp. CPBF 426

0.00 1387 1387 S 1680158 Xanthomonas sp. ISO98C4

0.00 1122 1122 S 2776703 Xanthomonas sp. WG16

0.01 1631397 1631397 S 48664 Xanthomonas fragariae

0.00 1379405 1191579 S 56459 Xanthomonas vasicola

0.00 84773 0 S1 454958 Xanthomonas vasicola pv. musacearum

0.00 84773 84773 S2 1094184 Xanthomonas campestris pv. musacearum NCPPB 4379

0.00 58191 58191 S1 325776 Xanthomonas vasicola pv. vasculorum

0.00 44862 44862 S1 487849 Xanthomonas vasicola pv. arecae

0.00 1329010 1080048 S 56460 Xanthomonas vesicatoria

0.00 248962 248962 S1 925775 Xanthomonas vesicatoria ATCC 35937

0.00 1260840 1260840 S 2053930 Xanthomonas prunicola

0.00 1041282 1018236 S 29447 Xanthomonas albilineans

0.00 23046 23046 S1 380358 Xanthomonas albilineans GPE PC73

0.00 922118 922118 S 56453 Xanthomonas cucurbitae

0.00 904278 904278 S 56464 Xanthomonas theicola

0.00 816412 0 S 56450 Xanthomonas cassavae

0.00 816412 816412 S1 1219375 Xanthomonas cassavae CFBP 4642

0.00 805645 805645 S 56455 Xanthomonas hyacinthi

0.00 788720 788720 S 2259622 Xanthomonas euroxanthea

0.00 753067 304 S 53413 Xanthomonas axonopodis

0.00 690198 0 S1 1982678 Xanthomonas axonopodis subcluster 9.3

0.00 690198 690198 S2 325777 Xanthomonas axonopodis pv. vasculorum

0.00 57995 57995 S1 1101443 Xanthomonas axonopodis pv. commiphoreae

0.00 4570 4570 S1 1304892 Xanthomonas axonopodis Xac29-1

0.00 728171 728171 S 2775159 Xanthomonas hydrangeae

0.00 710407 78608 S 1985254 Xanthomonas phaseoli

0.00 330310 0 S1 92828 Xanthomonas phaseoli pv. dieffenbachiae

0.00 330310 330310 S2 1437877 Xanthomonas phaseoli pv. dieffenbachiae LMG 695

0.00 226606 226606 S1 43353 Xanthomonas phaseoli pv. manihotis

0.00 74883 74883 S1 317013 Xanthomonas phaseoli pv. phaseoli

0.00 539353 254990 S 456327 Xanthomonas euvesicatoria

0.00 107453 0 S1 207721 Xanthomonas euvesicatoria pv. allii

0.00 107453 107453 S2 1437449 Xanthomonas euvesicatoria pv. allii CFBP 6369

0.00 91414 89752 S1 359387 Xanthomonas euvesicatoria pv. alfalfae

0.00 1662 1662 S2 1365647 Xanthomonas euvesicatoria pv. alfalfae CFBP 3836

0.00 71856 0 S1 189396 Xanthomonas euvesicatoria pv. citrumelonis

0.00 71856 71856 S2 981368 Xanthomonas axonopodis pv. citrumelo F1

0.00 13640 13640 S1 316273 Xanthomonas campestris pv. vesicatoria str. 85-10

0.00 97261 95345 S 442694 Xanthomonas perforans

0.00 1916 1916 S1 925776 Xanthomonas perforans 91-118

0.14 41978986 9183664 G 40323 Stenotrophomonas

0.07 21960227 1054951 G1 995085 Stenotrophomonas maltophilia group

0.05 16316559 15772763 S 40324 Stenotrophomonas maltophilia

0.00 266425 266425 S1 868597 Stenotrophomonas maltophilia JV3

0.00 123631 123631 S1 391008 Stenotrophomonas maltophilia R551-3

0.00 99648 99648 S1 1163399 Stenotrophomonas maltophilia D457

0.00 39264 39264 S1 522373 Stenotrophomonas maltophilia K279a

0.00 14828 14828 S1 1190567 Stenotrophomonas maltophilia EPM1

0.02 4588717 1324210 G2 2961925 unclassified Stenotrophomonas maltophilia group

0.00 796287 796287 S 2072414 Stenotrophomonas sp. ESTM1D_MKCIP4_1

0.00 688320 688320 S 2072413 Stenotrophomonas sp. SAU14A_NAIMI4_5

0.00 467813 467813 S 2072405 Stenotrophomonas sp. ZAC14D2_NAIMI4_7

0.00 360247 360247 S 2072412 Stenotrophomonas sp. ZAC14A_NAIMI4_1

0.00 325837 325837 S 2072407 Stenotrophomonas sp. YAU14D1_LEIMI4_1

0.00 302311 302311 S 2072406 Stenotrophomonas sp. ZAC14D2_NAIMI4_6

0.00 162251 162251 S 2072409 Stenotrophomonas sp. SAU14A_NAIMI4_8

0.00 157439 157439 S 2072408 Stenotrophomonas sp. YAU14A_MKIMI4_1

0.00 2097 2097 S 2072410 Stenotrophomonas sp. ZAC14D1_NAIMI4_6

0.00 1905 1905 S 2072411 Stenotrophomonas sp. ZAC14D1_NAIMI4_1

0.02 5881231 430768 G1 196198 unclassified Stenotrophomonas

0.00 1094239 1094239 S 2770322 Stenotrophomonas sp. 169

0.00 973339 973339 S 1904944 Stenotrophomonas sp. LM091

0.00 864619 864619 S 2691571 Stenotrophomonas sp. 364

0.00 690294 690294 S 2742129 Stenotrophomonas sp. NA06056

0.00 376903 376903 S 2005046 Stenotrophomonas sp. WZN-1

0.00 293226 293226 S 2871174 Stenotrophomonas sp. DR822

0.00 165170 165170 S 1827305 Stenotrophomonas sp. MYb57

0.00 157804 157804 S 2040586 Stenotrophomonas sp. Pemsol

0.00 151232 151232 S 2211160 Stenotrophomonas sp. pho

0.00 137245 137245 S 2303750 Stenotrophomonas sp. G4

0.00 134207 134207 S 2886359 Stenotrophomonas sp. SI-NJAU-1

0.00 132553 132553 S 2282124 Stenotrophomonas sp. ASS1

0.00 112250 112250 S 2939415 Stenotrophomonas sp. NY11291

0.00 90931 90931 S 2775920 Stenotrophomonas sp. CW117

0.00 64758 64758 S 2682487 Stenotrophomonas sp. SXG-1

0.00 8050 8050 S 2565559 Stenotrophomonas sp. PAMC25021

0.00 3643 3643 S 1793721 Stenotrophomonas sp. KCTC 12332

0.01 3260073 3260073 S 216778 Stenotrophomonas rhizophila

0.00 1383991 1383991 S 128780 Stenotrophomonas acidaminiphila

0.00 284977 284977 S 2045451 Stenotrophomonas indicatrix

0.00 21833 21833 S 487698 Stenotrophomonas pavanii

0.00 2990 2990 S 83617 Stenotrophomonas nitritireducens

0.10 31417791 4229679 G 68 Lysobacter

0.03 7663510 39416 G1 2635362 unclassified Lysobacter

0.00 1152612 1152612 S 2820808 Lysobacter sp. K5869

0.00 1144416 1144416 S 2904253 Lysobacter sp. 5GHs7-4

0.00 931263 931263 S 2925843 Lysobacter sp. S4-A87

0.00 879665 879665 S 2904252 Lysobacter sp. KIS68-7

0.00 873595 873595 S 2290922 Lysobacter sp. TY2-98

0.00 779013 779013 S 2762611 Lysobacter sp. CW239

0.00 666350 666350 S 2714936 Lysobacter sp. HDW10

0.00 647057 647057 S 2764185 Lysobacter sp. CJ11

0.00 335661 335661 S 2781021 Lysobacter sp. H21R4

0.00 214462 214462 S 2781024 Lysobacter sp. H23M47

0.01 4094081 4094081 S 69 Lysobacter enzymogenes

0.01 3883130 3883130 S 435897 Lysobacter capsici

0.01 1920130 1920130 S 84531 Lysobacter antibioticus

0.01 1669261 1669261 S 453783 Lysobacter soli

0.00 1052551 1052551 S 2763317 Lysobacter solisilvae

0.00 1047418 1047418 S 2591633 Lysobacter alkalisoli

0.00 1033999 1033999 S 1605891 Lysobacter maris

0.00 949554 949554 S 2795387 Lysobacter arenosi

0.00 789409 789409 S 2865112 Lysobacter terrestris

0.00 727189 727189 S 2822368 Lysobacter luteus

0.00 682606 682606 S 1324796 Lysobacter lycopersici

0.00 674419 674419 S 2698682 Lysobacter oculi

0.00 441463 441463 S 262324 Lysobacter gummosus

0.00 335830 335830 S 2781022 Lysobacter ciconiae

0.00 220227 220227 S 2781023 Lysobacter avium

0.00 1738 1738 S 2675059 Lysobacter helvus

0.00 1597 1597 S 2675101 Lysobacter caseinilyticus

0.03 8327095 148502 G 83614 Luteimonas

0.02 4748809 115620 G1 2629088 unclassified Luteimonas

0.00 978970 978970 S 2925842 Luteimonas sp. S4-F44

0.00 815654 815654 S 1896164 Luteimonas sp. JM171

0.00 786817 786817 S 2508168 Luteimonas sp. YGD11-2

0.00 732231 732231 S 2799326 Luteimonas sp. MC1750

0.00 678178 678178 S 2799325 Luteimonas sp. MC1572

0.00 641339 641339 S 2761107 Luteimonas sp. MC1825

0.00 1062658 1062658 S 2901869 Luteimonas fraxinea

0.00 893300 893300 S 2006110 Luteimonas chenhongjianii

0.00 750147 750147 S 2565782 Luteimonas yindakuii

0.00 723679 723679 S 1176533 Luteimonas granuli

0.02 7476589 1079789 G 83618 Pseudoxanthomonas

0.01 1772436 929967 S 314722 Pseudoxanthomonas suwonensis

0.00 842469 842469 S1 743721 Pseudoxanthomonas suwonensis 11-1

0.01 1730225 1730225 S 128785 Pseudoxanthomonas mexicana

0.00 1127110 7 G1 2645906 unclassified Pseudoxanthomonas

0.00 1099039 1099039 S 2571115 Pseudoxanthomonas sp. X-1

0.00 28064 28064 S 2932492 Pseudoxanthomonas sp. F37

0.00 888387 888387 S 266062 Pseudoxanthomonas daejeonensis

0.00 878642 0 S 415229 Pseudoxanthomonas spadix

0.00 878642 878642 S1 1045855 Pseudoxanthomonas spadix BD-a59

0.02 4750311 110628 G 141948 Thermomonas

0.01 2196585 11363 G1 2633315 unclassified Thermomonas

0.00 742237 742237 S 2771436 Thermomonas sp. XSG

0.00 725568 725568 S 2714945 Thermomonas sp. HDW16

0.00 717417 717417 S 2884874 Thermomonas sp. IMCC34681

0.00 926010 926010 S 1463158 Thermomonas carbonis

0.00 803974 803974 S 215691 Thermomonas brevis

0.00 713114 713114 S 2202149 Thermomonas aquatica

0.01 2988836 22917 G 2370 Xylella

0.01 2087897 1847448 S 2371 Xylella fastidiosa

0.00 114617 113089 S1 671135 Xylella fastidiosa subsp. sandyi

0.00 1528 1528 S2 155920 Xylella fastidiosa subsp. sandyi Ann-1

0.00 86371 86371 S1 644357 Xylella fastidiosa subsp. multiplex

0.00 21815 21815 S1 644356 Xylella fastidiosa subsp. fastidiosa

0.00 7529 7529 S1 2783797 Xylella fastidiosa subsp. morus

0.00 4321 4321 S1 1401256 Xylella fastidiosa MUL0034

0.00 1431 1431 S1 160492 Xylella fastidiosa 9a5c

0.00 1406 1406 S1 183190 Xylella fastidiosa Temecula1

0.00 1014 1014 S1 698414 Xylella fastidiosa subsp. pauca

0.00 981 981 S1 405441 Xylella fastidiosa M23

0.00 964 964 S1 405440 Xylella fastidiosa M12

0.00 878022 878022 S 1444770 Xylella taiwanensis

0.00 1443538 0 G 2709666 Pseudolysobacter

0.00 1443538 1443538 S 2511995 Pseudolysobacter antarcticus

0.00 836016 0 G 490567 Arenimonas

0.00 836016 836016 S 370777 Arenimonas daejeonensis

0.10 31764880 244542 F 1775411 Rhodanobacteraceae

0.03 9673844 173973 G 242605 Luteibacter

0.02 5441489 60456 G1 2620188 unclassified Luteibacter

0.00 1430069 1430069 S 2911538 Luteibacter sp. 335

0.00 1393169 1393169 S 2911539 Luteibacter sp. 353

0.00 1322918 1322918 S 2911537 Luteibacter sp. 321

0.00 1234877 1234877 S 2780091 Luteibacter sp. EIF3

0.00 1417690 1417690 S 564369 Luteibacter anthropi

0.00 1378166 0 S 242606 Luteibacter rhizovicinus

0.00 1378166 1378166 S1 1440763 Luteibacter rhizovicinus DSM 16549

0.00 1262526 1262526 S 2589080 Luteibacter pinisoli

0.03 7970410 134550 G 231454 Dyella

0.01 1541062 1541062 S 522259 Dyella terrae

0.00 1456931 1456931 S 1849581 Dyella caseinilytica

0.00 1366613 1366613 S 2763498 Dyella telluris

0.00 1303075 0 S 231455 Dyella japonica

0.00 1303075 1303075 S1 1217721 Dyella japonica A8

0.00 1163213 1163213 S 445710 Dyella thiooxydans

0.00 1004966 0 G1 2634549 unclassified Dyella

0.00 1004966 1004966 S 2501295 Dyella sp. M7H15-1

0.02 5036150 192416 G 75309 Rhodanobacter

0.01 1651839 1651839 S 666685 Rhodanobacter denitrificans

0.00 1173467 1173467 S 582702 Rhodanobacter glycinis

0.00 1034702 0 G1 2621553 unclassified Rhodanobacter

0.00 1034702 1034702 S 2778082 Rhodanobacter sp. FDAARGOS 1247

0.00 983726 983726 S 416169 Rhodanobacter thiooxydans

0.01 3019940 607 G 70411 Frateuria

0.01 1935526 55523 G1 2648894 unclassified Frateuria

0.00 989698 989698 S 2898793 Frateuria sp. 5GH9-34

0.00 890305 890305 S 1542730 Frateuria sp. 5GH9-11

0.00 1083807 0 S 81475 Frateuria aurantia

0.00 1083807 1083807 S1 767434 Frateuria aurantia DSM 6220

0.01 1953819 0 G 1453544 Tahibacter

0.01 1953819 0 G1 2636986 unclassified Tahibacter

0.01 1953819 1953819 S 2976241 Tahibacter sp. W38

0.01 1912040 0 G 2233801 Ahniella

0.01 1912040 1912040 S 2021234 Ahniella affigens

0.00 1229053 0 G 323413 Dokdonella

0.00 1229053 0 S 323415 Dokdonella koreensis

0.00 1229053 1229053 S1 1300342 Dokdonella koreensis DS-123

0.00 725082 0 G 2707020 Aerosticca

0.00 725082 725082 S 2010829 Aerosticca soli

0.44 135352358 75317 O 135619 Oceanospirillales

0.22 66863756 1101685 F 28256 Halomonadaceae

0.17 52140030 6899761 G 2745 Halomonas

0.09 27457385 5275024 G1 2609666 unclassified Halomonas

0.00 1497330 1497330 S 2562282 Halomonas sp. Y2R2

0.00 1394579 1394579 S 2953657 Halomonas sp. DN3

0.00 1297347 1297347 S 1504981 Halomonas sp. KO116

0.00 1194132 1194132 S 2733487 Halomonas sp. MCCC 1A13316

0.00 1151975 1151975 S 1610576 Halomonas sp. R57-5

0.00 1068129 1068129 S 2785909 Halomonas sp. A40-4

0.00 1050418 1050418 S 1118153 Halomonas sp. GFAJ-1

0.00 1004362 1004362 S 2832500 Halomonas sp. FeN2

0.00 999771 999771 S 2749040 Halomonas sp. SH5A2

0.00 988562 988562 S 1883416 Halomonas sp. 1513

0.00 946321 946321 S 2773308 Halomonas sp. YLGW01

0.00 901861 901861 S 2952527 Halomonas sp. 3H

0.00 898590 898590 S 2956797 Halomonas sp. Y3

0.00 878216 878216 S 2587844 Halomonas sp. THAF5a

0.00 861233 861233 S 999141 Halomonas sp. TD01

0.00 778756 778756 S 2587849 Halomonas sp. THAF12

0.00 756109 756109 S 1609967 Halomonas sp. HG01

0.00 727489 727489 S 1971364 Halomonas sp. GT

0.00 604633 604633 S 2306583 Halomonas sp. JS92-SW72

0.00 512668 512668 S 2730360 Halomonas sp. PGE1

0.00 500428 500428 S 2855441 Halomonas sp. 18071143

0.00 357656 357656 S 2136172 Halomonas sp. SF2003

0.00 305097 305097 S 2883106 Halomonas sp. NyZ770

0.00 279159 279159 S 1761789 Halomonas sp. hl-4

0.00 265853 265853 S 1666906 Halomonas sp. HL-93

0.00 251310 251310 S 2014541 Halomonas sp. N3-2A

0.00 212581 212581 S 507763 Halomonas sp. MS1

0.00 182811 182811 S 2854257 Halomonas sp. SS10-MC5

0.00 168278 168278 S 2557993 Halomonas sp. XH26

0.00 128235 128235 S 1346287 Halomonas sp. A3H3

0.00 7284 7284 S 1250400 Halomonas sp. ZM3

0.00 3254 3254 S 1630300 Halomonas sp. Ant2

0.00 2180 2180 S 2730854 Halomonas sp. TA6

0.00 2139 2139 S 2730914 Halomonas sp. TA22

0.00 2077 2077 S 2730357 Halomonas sp. PA5

0.00 1353 1353 S 223901 Halomonas sp. 40

0.00 109 109 S 1949203 Halomonas sp. 'Soap Lake #6'

0.00 76 76 S 1962264 Halomonas sp. 'Soap Lake #7'

0.01 2178336 2178336 S 29570 Halomonas meridiana

0.00 1433846 1433846 S 1178482 Halomonas huangheensis

0.00 1247781 1247781 S 2497861 Halomonas tianxiuensis

0.00 1180808 1180808 S 1081866 Halomonas socia

0.00 1153886 1153886 S 2609667 Halomonas piezotolerans

0.00 1146824 0 S 2746 Halomonas elongata

0.00 1146824 1146824 S1 768066 Halomonas elongata DSM 2581

0.00 1131176 1131176 S 2733488 Halomonas sulfidivorans

0.00 1113526 1113526 S 44935 Halomonas venusta

0.00 1108181 1108181 S 507626 Halomonas chromatireducens

0.00 1067942 1067942 S 2852117 Halomonas profundi

0.00 1031205 1031205 S 475662 Halomonas beimenensis

0.00 1026604 1026604 S 664683 Halomonas titanicae

0.00 882737 882737 S 1897729 Halomonas aestuarii

0.00 867073 867073 S 29571 Halomonas subglaciescola

0.00 689200 689200 S 115561 Halomonas hydrothermalis

0.00 184130 184130 S 2733484 Halomonas sulfidoxydans

0.00 180127 180127 S 272774 Halomonas alkaliphila

0.00 159502 159502 S 213554 Halomonas campaniensis

0.01 4429090 1338015 G 204286 Cobetia

0.01 2450824 92469 G1 2609414 unclassified Cobetia

0.00 1197210 1197210 S 2686360 Cobetia sp. L2A1

0.00 571921 571921 S 2609415 Cobetia sp. cqz5-12

0.00 307180 307180 S 2758724 Cobetia sp. 4B

0.00 250558 250558 S 2661553 Cobetia sp. AM6

0.00 31486 31486 S 2053669 Cobetia sp. ICG0124

0.00 286524 286524 S 1055101 Cobetia pacifica

0.00 237445 237445 S 1055104 Cobetia amphilecti

0.00 116282 116282 S 28258 Cobetia marina

0.01 3174297 168557 G 504090 Kushneria

0.00 1120574 1120574 S 657387 Kushneria phosphatilytica

0.00 955864 955864 S 157779 Kushneria marisflavi

0.00 929302 929302 S 698828 Kushneria konosiri

0.00 1314744 104 F1 114403 Zymobacter group

0.00 918644 0 G 33073 Zymobacter

0.00 918644 918644 S 33074 Zymobacter palmae

0.00 260768 0 G 235572 Candidatus Portiera

0.00 260768 259053 S 91844 Candidatus Portiera aleyrodidarum

0.00 689 689 S1 1239881 Candidatus Portiera aleyrodidarum BT-QVLC

0.00 552 552 S1 1163752 Candidatus Portiera aleyrodidarum MED (Bemisia tabaci)

0.00 415 415 S1 1206109 Candidatus Portiera aleyrodidarum BT-B-HRs

0.00 59 59 S1 1297582 Candidatus Portiera aleyrodidarum TV

0.00 135228 3714 G 114185 Candidatus Carsonella

0.00 115112 46744 S 114186 Candidatus Carsonella ruddii

0.00 15394 15394 S1 387662 Candidatus Carsonella ruddii PV

0.00 12742 12742 S1 1202540 Candidatus Carsonella ruddii PC isolate NHV

0.00 10432 10432 S1 1202539 Candidatus Carsonella ruddii HT isolate Thao2000

0.00 10138 0 S1 134277 primary endosymbiont of Heteropsylla cubana

0.00 10138 10138 S2 1202538 Candidatus Carsonella ruddii HC isolate Thao2000

0.00 9820 9820 S1 1202536 Candidatus Carsonella ruddii CE isolate Thao2000

0.00 9723 9723 S1 1202537 Candidatus Carsonella ruddii CS isolate Thao2000

0.00 119 119 S1 667013 Candidatus Carsonella ruddii DC

0.00 16402 16402 S 2661587 Candidatus Carsonella ruddii (Diaphorina cf. continua)

0.00 1280363 0 G 376488 Halotalea

0.00 1280363 1280363 S 376489 Halotalea alkalilenta

0.00 1236247 0 G 404432 Salinicola

0.00 1236247 1236247 S 1771309 Salinicola tamaricis

0.00 1112808 0 G 1897649 Pistricoccus

0.00 1112808 1112808 S 1883414 Pistricoccus aurantiacus

0.00 1074492 0 G 42054 Chromohalobacter

0.00 1074492 0 S 158080 Chromohalobacter salexigens

0.00 1074492 1074492 S1 290398 Chromohalobacter salexigens DSM 3043

0.13 38273570 36934 F 135620 Oceanospirillaceae

0.05 14377792 501321 G 28253 Marinomonas

0.01 3701600 32614 G1 196814 unclassified Marinomonas

0.00 1401943 1401943 S 2066133 Marinomonas sp. CT5

0.00 1190529 1190529 S 2065312 Marinomonas sp. A3A

0.00 1076514 1076514 S 2071621 Marinomonas sp. FW-1

0.01 1834802 1834802 S 178399 Marinomonas primoryensis

0.00 1445925 0 S 119864 Marinomonas mediterranea

0.00 1445925 1445925 S1 717774 Marinomonas mediterranea MMB-1

0.00 1347379 1347379 S 2773454 Marinomonas algicola

0.00 1293491 1293491 S 383750 Marinomonas arctica

0.00 1189769 0 S 936476 Marinomonas posidonica

0.00 1189769 1189769 S1 491952 Marinomonas posidonica IVIA-Po-181

0.00 1071879 1071879 S 491948 Marinomonas rhizomae

0.00 1035925 1035925 S 2726122 Marinomonas profundi

0.00 955701 955701 S 491950 Marinomonas foliarum

0.02 6115999 69171 G 48075 Marinobacterium

0.00 1520131 1520131 S 1821621 Marinobacterium aestuarii

0.00 1517103 1517103 S 420402 Marinobacterium rhizophilum

0.00 1186692 1186692 S 48076 Marinobacterium georgiense

0.00 1080950 1080950 S 518898 Marinobacterium sediminicola

0.00 741952 0 G1 2644139 unclassified Marinobacterium

0.00 741952 741952 S 2668067 Marinobacterium sp. LSUCC0821

0.01 4297728 9722 G 515417 Amphritea

0.01 1608120 1608120 S 1524258 Amphritea ceti

0.00 1486302 1486302 S 355243 Amphritea atlantica

0.00 1193584 1191338 S 452627 Amphritea japonica

0.00 2246 2246 S1 1278309 Amphritea japonica ATCC BAA-1530

0.01 4027426 316978 G 187492 Thalassolituus

0.01 1765923 1305310 S 187493 Thalassolituus oleivorans

0.00 241420 241420 S1 1298593 Thalassolituus oleivorans MIL-1

0.00 219193 219193 S1 1208320 Thalassolituus oleivorans R6-15

0.00 1013054 0 G1 2624967 unclassified Thalassolituus

0.00 1013054 1013054 S 2597518 Thalassolituus sp. C2-1

0.00 931471 931471 S 671053 Thalassolituus marinus

0.01 3624046 3567 G 75687 Neptunomonas

0.00 1275353 0 S 417574 Neptunomonas japonica

0.00 1275353 1275353 S1 1441457 Neptunomonas japonica JAMM 1380

0.00 1237464 1237464 S 1572645 Neptunomonas phycophila

0.00 1107662 1107662 S 1031538 Neptunomonas concharum

0.00 1331752 0 G 1537406 Bacterioplanes

0.00 1331752 1331752 S 1249553 Bacterioplanes sanyensis

0.00 1281128 0 G 267849 Nitrincola

0.00 1281128 1281128 S 2614693 Nitrincola iocasae

0.00 1222540 0 G 2066071 Bacterioplanoides

0.00 1222540 0 G1 2630303 unclassified Bacterioplanoides

0.00 1222540 1222540 S 2829569 Bacterioplanoides sp. SCSIO 12839

0.00 1116843 0 G 599651 Bermanella

0.00 1116843 1116843 S 207949 Bermanella marisrubri

0.00 841382 0 G 2893036 Venatorbacter

0.00 841382 841382 S 2661630 Venatorbacter cucullus

0.03 8798803 239 F 224372 Alcanivoracaceae

0.02 7247413 1287476 G 59753 Alcanivorax

0.01 2491923 13867 G1 2638842 unclassified Alcanivorax

0.00 1216936 1216936 S 2014542 Alcanivorax sp. N3-2A

0.00 1171647 1171647 S 2259335 Alcanivorax sp. ALC70

0.00 89473 89473 S 1113728 Alcanivorax sp. NBRC 101098

0.00 1223770 0 S 1306787 Alcanivorax pacificus

0.00 1223770 1223770 S1 391936 Alcanivorax pacificus W11-5

0.00 1111604 0 S 285091 Alcanivorax dieselolei

0.00 1111604 1111604 S1 930169 Alcanivorax dieselolei B5

0.00 1040674 1040674 S 1094342 Alcanivorax xenomutans

0.00 91966 0 S 59754 Alcanivorax borkumensis

0.00 91966 91966 S1 393595 Alcanivorax borkumensis SK2

0.01 1551151 0 G 2025617 Ketobacter

0.01 1551151 1551151 S 1917421 Ketobacter alkanivorans

0.03 8136683 0 F 2066474 Endozoicomonadaceae

0.03 8136683 244402 G 305899 Endozoicomonas

0.01 3332187 1175 G1 2644528 unclassified Endozoicomonas

0.01 1717415 1717415 S 2872754 Endozoicomonas sp. 4G

0.01 1613597 1613597 S 2918516 Endozoicomonas sp. SCSIO W0465

0.00 1526097 1526097 S 1234143 Endozoicomonas euniceicola

0.00 1520771 1520771 S 1234144 Endozoicomonas gorgoniicola

0.00 1513226 0 S 1027273 Endozoicomonas montiporae

0.00 1513226 1513226 S1 570277 Endozoicomonas montiporae CL-33

0.01 4302927 370 F 255527 Saccharospirillaceae

0.01 2024524 0 G 1445504 Gynuella

0.01 2024524 0 S 1445505 Gynuella sunshinyii

0.01 2024524 2024524 S1 1445510 Gynuella sunshinyii YC6258

0.00 1141829 0 G 230494 Reinekea

0.00 1141829 1141829 S 1336806 Reinekea forsetii

0.00 1136204 0 G 231683 Saccharospirillum

0.00 1136204 1136204 S 2161747 Saccharospirillum mangrovi

0.01 4156032 0 F 224379 Hahellaceae

0.01 4156032 293154 G 158481 Hahella

0.01 1968660 0 S 158327 Hahella chejuensis

0.01 1968660 1968660 S1 349521 Hahella chejuensis KCTC 2396

0.01 1894218 0 G1 2624107 unclassified Hahella

0.01 1894218 1894218 S 1628392 Hahella sp. KA22

0.01 2029044 0 F 2898533 Zooshikellaceae

0.01 2029044 0 G 2768738 Spartinivicinus

0.01 2029044 2029044 S 2683272 Spartinivicinus ruber

0.01 1979640 0 F 191033 Oleiphilaceae

0.01 1979640 0 G 141450 Oleiphilus

0.01 1979640 1979640 S 141451 Oleiphilus messinensis

0.00 736586 0 F 449732 Litoricolaceae

0.00 736586 0 G 418700 Litoricola

0.00 736586 736586 S 418701 Litoricola lipolytica

0.40 121878780 0 O 2887326 Moraxellales

0.40 121878780 109743 F 468 Moraxellaceae

0.32 96686529 19362320 G 469 Acinetobacter

0.07 20797042 2945089 G1 196816 unclassified Acinetobacter

0.00 1171799 1171799 S 2714110 Acinetobacter sp. Marseille-Q1620

0.00 1048577 1048577 S 1646498 Acinetobacter sp. TTH0-4

0.00 1019013 1019013 S 1407071 Acinetobacter sp. TGL-Y2

0.00 1012768 1012768 S 1879049 Acinetobacter sp. WCHAc010034

0.00 858311 858311 S 2953738 Acinetobacter sp. Z1

0.00 811358 811358 S 2798861 Acinetobacter sp. CS-2

0.00 785354 785354 S 1808001 Acinetobacter sp. LoGeW2-3

0.00 782292 782292 S 2950074 Acinetobacter sp. C32I

0.00 781104 781104 S 2563897 Acinetobacter sp. 10FS3-1

0.00 758836 758836 S 1608473 Acinetobacter sp. NCu2D-2

0.00 720043 720043 S 2943497 Acinetobacter sp. Y-23

0.00 596554 596554 S 2773709 Acinetobacter sp. ASP199

0.00 557449 557449 S 2004646 Acinetobacter sp. WCHA55

0.00 522696 522696 S 2609668 Acinetobacter sp. C16S1

0.00 514757 514757 S 2923375 Acinetobacter sp. XS-4

0.00 470888 470888 S 2004647 Acinetobacter sp. WCHAc010052

0.00 447107 447107 S 2929509 Acinetobacter sp. NyZ410

0.00 392854 392854 S 2908637 Acinetobacter sp. SCLZS86

0.00 373948 373948 S 1758189 Acinetobacter sp. ACNIH2

0.00 353733 353733 S 1827285 Acinetobacter sp. MYb10

0.00 331587 331587 S 2930198 Acinetobacter sp. PK01

0.00 280992 280992 S 2913495 Acinetobacter sp. AHP123

0.00 272714 272714 S 2929514 Acinetobacter sp. YH16056_T

0.00 264675 264675 S 2004644 Acinetobacter sp. WCHA45

0.00 253319 253319 S 2601122 Acinetobacter sp. YH12138

0.00 247718 247718 S 2929512 Acinetobacter sp. YH16040_T

0.00 236984 236984 S 2871688 Acinetobacter sp. AS23

0.00 236224 236224 S 2905879 Acinetobacter sp. SH20PTE14

0.00 226299 226299 S 2853158 Acinetobacter sp. F9

0.00 206853 206853 S 2929515 Acinetobacter sp. YH12068_T

0.00 158759 158759 S 2743575 Acinetobacter sp. NEB 394

0.00 152059 152059 S 2708348 Acinetobacter sp. WY4

0.00 151525 151525 S 2079596 Acinetobacter sp. SWBY1

0.00 149510 149510 S 710648 Acinetobacter sp. Tol 5

0.00 144681 144681 S 2810070 Acinetobacter sp. Colony158

0.00 133889 133889 S 2836181 Acinetobacter sp. BHS4

0.00 124896 124896 S 1636603 Acinetobacter sp. ACNIH1

0.00 94809 94809 S 2725684 Acinetobacter sp. NEB149

0.00 88056 88056 S 2545797 Acinetobacter sp. FDAARGOS_724

0.00 64538 64538 S 2925837 Acinetobacter sp. LUNF3

0.00 15670 15670 S 2420300 Acinetobacter sp. FDAARGOS_493

0.00 13019 13019 S 2781976 Acinetobacter sp. Ac-14

0.00 12091 12091 S 2675741 Acinetobacter sp. BEC1-S18-ESBL-01

0.00 5092 5092 S 1280052 Acinetobacter sp. M131

0.00 2353 2353 S 2950076 Acinetobacter sp. C26M

0.00 1984 1984 S 2950075 Acinetobacter sp. C26G

0.00 1187 1187 S 472 Acinetobacter sp.

0.00 376 376 S 2420301 Acinetobacter sp. FDAARGOS_494

0.00 295 295 S 2420305 Acinetobacter sp. FDAARGOS_560

0.00 143 143 S 61312 Acinetobacter sp. SUN

0.00 135 135 S 156739 Acinetobacter sp. EB104

0.00 74 74 S 2905880 Acinetobacter sp. SH20PTE17

0.00 6 6 S 309867 Acinetobacter sp. LUH5605

0.06 18847826 2903747 G1 909768 Acinetobacter calcoaceticus/baumannii complex

0.02 6613321 6518640 S 470 Acinetobacter baumannii

0.00 29003 29003 S1 1400867 Acinetobacter baumannii ZW85-1

0.00 14302 14302 S1 1279013 Acinetobacter baumannii PR07

0.00 8749 8749 S1 1401639 Acinetobacter baumannii NCGM 237

0.00 8342 8342 S1 557600 Acinetobacter baumannii AB307-0294

0.00 5988 5988 S1 1370126 Acinetobacter baumannii DU202

0.00 5110 5110 S1 509173 Acinetobacter baumannii AYE

0.00 4811 4811 S1 945556 Acinetobacter baumannii D1279779

0.00 4776 4776 S1 400667 Acinetobacter baumannii ATCC 17978

0.00 2746 2746 S1 405416 Acinetobacter baumannii ACICU

0.00 1935 1935 S1 1096996 Acinetobacter baumannii BJAB0715

0.00 1802 1802 S1 696749 Acinetobacter baumannii 1656-2

0.00 1621 1621 S1 1096997 Acinetobacter baumannii BJAB0868

0.00 1361 1361 S1 1100841 Acinetobacter baumannii TYTH-1

0.00 941 941 S1 1096995 Acinetobacter baumannii BJAB07104

0.00 690 690 S1 1455315 Acinetobacter baumannii LAC-4

0.00 603 603 S1 889738 Acinetobacter baumannii MDR-TJ

0.00 572 572 S1 480119 Acinetobacter baumannii AB0057

0.00 533 533 S1 575584 Acinetobacter baumannii ATCC 19606 = CIP 70.34 = JCM 6841

0.00 502 502 S1 497978 Acinetobacter baumannii MDR-ZJ06

0.00 292 292 S1 1413216 Acinetobacter baumannii PKAB07

0.00 2 2 S1 1149135 Acinetobacter baumannii 107m

0.01 2924226 2827011 S 48296 Acinetobacter pittii

0.00 97215 97215 S1 871585 Acinetobacter pittii PHEA-2

0.01 2311477 2311477 S 1530123 Acinetobacter seifertii

0.01 2207681 2207681 S 471 Acinetobacter calcoaceticus

0.01 1572350 1494234 S 106654 Acinetobacter nosocomialis

0.00 78116 78116 S1 1343071 Acinetobacter nosocomialis M2

0.00 315024 315024 S 1785128 Acinetobacter lactucae

0.01 3977457 3910662 S 40214 Acinetobacter johnsonii

0.00 66795 66795 S1 1242245 Acinetobacter johnsonii XBB1

0.01 2815458 2815458 S 756892 Acinetobacter indicus

0.01 2111192 2111192 S 40215 Acinetobacter junii

0.01 1916532 1916532 S 29430 Acinetobacter haemolyticus

0.01 1654311 1614960 S 28090 Acinetobacter lwoffii

0.00 39351 39351 S1 1046625 Acinetobacter lwoffii WJ10621

0.01 1612792 1612792 S 108981 Acinetobacter schindleri

0.01 1552035 1552035 S 1879050 Acinetobacter wuhouensis

0.01 1541150 1420130 S 40216 Acinetobacter radioresistens

0.00 121020 121020 S1 981334 Acinetobacter radioresistens DSM 6976 = NBRC 102413 = CIP 103788

0.00 1244743 1244743 S 487316 Acinetobacter soli

0.00 1105612 1105612 S 1789224 Acinetobacter larvae

0.00 1010528 1010528 S 2006115 Acinetobacter piscicola

0.00 1004661 1004661 S 70346 Acinetobacter variabilis

0.00 984185 982429 S 202950 Acinetobacter baylyi

0.00 1756 1756 S1 62977 Acinetobacter baylyi ADP1

0.00 982832 982832 S 2715164 Acinetobacter shaoyimingii

0.00 941413 941413 S 202956 Acinetobacter towneri

0.00 929476 929476 S 202954 Acinetobacter tandoii

0.00 923068 923068 S 280145 Acinetobacter colistiniresistens

0.00 908556 908556 S 2715163 Acinetobacter lanii

0.00 902553 902553 S 1871111 Acinetobacter defluvii

0.00 895258 895258 S 106648 Acinetobacter bereziniae

0.00 891971 891971 S 2136182 Acinetobacter cumulans

0.00 856045 856045 S 106649 Acinetobacter guillouiae

0.00 823531 823531 S 70348 Acinetobacter dispersus

0.00 819927 1622 S 52133 Acinetobacter venetianus

0.00 818305 818305 S1 1197884 Acinetobacter venetianus VE-C3

0.00 817877 817877 S 1324350 Acinetobacter equi

0.00 787331 787331 S 134534 Acinetobacter gyllenbergii

0.00 727927 727927 S 2662362 Acinetobacter wanghuae

0.00 664337 664337 S 1839785 Acinetobacter portensis

0.00 556337 556337 S 108980 Acinetobacter ursingii

0.00 546378 546378 S 202951 Acinetobacter bouvetii

0.00 486794 486794 S 2004650 Acinetobacter chinensis

0.00 252341 252341 S 2053287 Acinetobacter pseudolwoffii

0.00 247200 0 S 1148157 Acinetobacter oleivorans

0.00 247200 247200 S1 436717 Acinetobacter oleivorans DR1

0.00 187533 187533 S 465797 Acinetobacter septicus

0.05 14513189 1688176 G 497 Psychrobacter

0.03 8799565 2843626 G1 196806 unclassified Psychrobacter

0.00 937507 937507 S 1028416 Psychrobacter sp. DAB_AL43B

0.00 672931 672931 S 1699623 Psychrobacter sp. P11G3

0.00 630760 630760 S 2772254 Psychrobacter sp. 28M-43

0.00 579838 579838 S 1699621 Psychrobacter sp. P11F6

0.00 567269 567269 S 2708350 Psychrobacter sp. WY6

0.00 399240 399240 S 571800 Psychrobacter sp. G

0.00 383929 383929 S 1699624 Psychrobacter sp. P11G5

0.00 362043 362043 S 1699622 Psychrobacter sp. P2G3

0.00 302407 302407 S 2733866 Psychrobacter sp. KCTC 72983

0.00 300974 300974 S 2565531 Psychrobacter sp. PraFG1

0.00 235379 235379 S 56811 Psychrobacter sp.

0.00 231264 231264 S 2203895 Psychrobacter sp. YP14

0.00 182131 182131 S 2517899 Psychrobacter sp. KH172YL61

0.00 136293 136293 S 1720344 Psychrobacter sp. AntiMn-1

0.00 16117 16117 S 1028414 Psychrobacter sp. DAB_AL32B

0.00 9042 9042 S 1028420 Psychrobacter sp. DAB_AL62B

0.00 5525 5525 S 1028419 Psychrobacter sp. DAB_AL60

0.00 1894 1894 S 2565557 Psychrobacter sp. PAMC27889

0.00 1396 1396 S 1028409 Psychrobacter sp. DAB_AL109bw

0.00 1140416 1140416 S 256326 Psychrobacter arenosus

0.00 955387 955387 S 861445 Psychrobacter sanguinis

0.00 862018 862018 S 45610 Psychrobacter urativorans

0.00 665785 0 S 334543 Psychrobacter arcticus

0.00 665785 665785 S1 259536 Psychrobacter arcticus 273-4

0.00 396504 395026 S 330922 Psychrobacter cryohalolentis

0.00 1478 1478 S1 335284 Psychrobacter cryohalolentis K5

0.00 3973 3973 S 261164 Psychrobacter alimentarius

0.00 1365 1365 S 256325 Psychrobacter maritimus

0.03 9406398 564205 G 475 Moraxella

0.01 3171568 3171568 S 34062 Moraxella osloensis

0.00 965104 688392 G1 2685852 unclassified Moraxella

0.00 273344 273344 S 2953752 Moraxella sp. FZFQ2102

0.00 1203 1203 S 2961618 Moraxella sp. FZLJ2107

0.00 1150 1150 S 2961890 Moraxella sp. FZLJ2109

0.00 1015 1015 S 77152 Moraxella sp. TA144

0.00 932676 921798 S 480 Moraxella catarrhalis

0.00 10878 10878 S1 1236608 Moraxella catarrhalis BBH18

0.00 869479 869479 S 386891 Moraxella bovoculi

0.00 771516 771364 S 476 Moraxella bovis

0.00 152 152 S1 387425 Moraxella bovis Epp63

0.00 643113 643113 S 34061 Moraxella cuniculi

0.00 639128 639128 S 478 Moraxella nonliquefaciens

0.00 606270 606270 S 2904121 Moraxella nasovis

0.00 243339 243339 S 29433 Moraxella ovis

0.00 1162921 0 G 2824158 Aquirhabdus

0.00 1162921 1162921 S 2283318 Aquirhabdus parva

0.20 62118094 119 O 135624 Aeromonadales

0.20 60336856 59164 F 84642 Aeromonadaceae

0.18 53551906 16179002 G 642 Aeromonas

0.02 6476059 6162696 S 654 Aeromonas veronii

0.00 172115 172115 S1 1347420 Aeromonas veronii Hm21

0.00 141248 141248 S1 998088 Aeromonas veronii B565

0.02 6331742 5518144 S 644 Aeromonas hydrophila

0.00 391733 391733 S1 1354302 Aeromonas hydrophila 4AK4

0.00 280243 152946 S1 196023 Aeromonas hydrophila subsp. hydrophila

0.00 125744 125744 S2 380703 Aeromonas hydrophila subsp. hydrophila ATCC 7966

0.00 1553 1553 S2 1321367 Aeromonas hydrophila subsp. hydrophila AL09-71

0.00 118869 118869 S1 1448139 Aeromonas hydrophila YL17

0.00 17501 17501 S1 1416915 Aeromonas hydrophila NJ-35

0.00 1976 1976 S1 1419584 Aeromonas hydrophila J-1

0.00 1698 1698 S1 1288394 Aeromonas hydrophila ML09-119

0.00 1578 1578 S1 1418107 Aeromonas hydrophila pc104A

0.02 5642066 222189 G1 257493 unclassified Aeromonas

0.00 812164 812164 S 2033032 Aeromonas sp. CA23

0.00 752586 752586 S 2033033 Aeromonas sp. CU5

0.00 661252 661252 S 2778064 Aeromonas sp. FDAARGOS 1415

0.00 295204 295204 S 2778054 Aeromonas sp. FDAARGOS 1405

0.00 292587 292587 S 2778058 Aeromonas sp. FDAARGOS 1409

0.00 226025 226025 S 1636609 Aeromonas sp. ASNIH4

0.00 217488 217488 S 2778056 Aeromonas sp. FDAARGOS 1407

0.00 182622 182622 S 2778059 Aeromonas sp. FDAARGOS 1410

0.00 173273 173273 S 2778066 Aeromonas sp. FDAARGOS 1417

0.00 160808 160808 S 2778053 Aeromonas sp. FDAARGOS 1404

0.00 158914 158914 S 2560028 Aeromonas sp. 1805

0.00 145021 145021 S 2778068 Aeromonas sp. FDAARGOS 1419

0.00 137606 137606 S 2778065 Aeromonas sp. FDAARGOS 1416

0.00 133961 133961 S 2778063 Aeromonas sp. FDAARGOS 1414

0.00 131673 131673 S 2778052 Aeromonas sp. FDAARGOS 1403

0.00 129818 129818 S 2778060 Aeromonas sp. FDAARGOS 1411

0.00 105483 105483 S 2560029 Aeromonas sp. 2692-1

0.00 95649 95649 S 2778057 Aeromonas sp. FDAARGOS 1408

0.00 89772 89772 S 1636607 Aeromonas sp. ASNIH2

0.00 88318 88318 S 2778051 Aeromonas sp. FDAARGOS 1402

0.00 80676 80676 S 1758179 Aeromonas sp. ASNIH5

0.00 73673 73673 S 2778055 Aeromonas sp. FDAARGOS 1406

0.00 57800 57800 S 1636606 Aeromonas sp. ASNIH1

0.00 49328 49328 S 1636608 Aeromonas sp. ASNIH3

0.00 49053 49053 S 2778067 Aeromonas sp. FDAARGOS 1418

0.00 48865 48865 S 2675707 Aeromonas sp. WP2-W18-CRE-05

0.00 37980 37980 S 1920107 Aeromonas sp. ASNIH7

0.00 20871 20871 S 1675689 Aeromonas sp. Ne-1

0.00 11346 11346 S 647 Aeromonas sp.

0.00 56 56 S 2127034 Aeromonas sp. pRIVM0001_VIM-1

0.00 5 5 S 1622273 Aeromonas sp. C3

0.01 3292903 2912559 S 645 Aeromonas salmonicida

0.00 173487 0 S1 96473 Aeromonas salmonicida subsp. pectinolytica

0.00 173487 173487 S2 1324960 Aeromonas salmonicida subsp. pectinolytica 34mel

0.00 151436 143375 S1 29491 Aeromonas salmonicida subsp. salmonicida

0.00 6183 6183 S2 382245 Aeromonas salmonicida subsp. salmonicida A449

0.00 1878 1878 S2 1076135 Aeromonas salmonicida subsp. salmonicida 01-B526

0.00 54614 54614 S1 197700 Aeromonas salmonicida subsp. masoucida

0.00 807 807 S1 80745 Aeromonas salmonicida subsp. smithia

0.01 2709409 2709409 S 648 Aeromonas caviae

0.01 2382434 2335937 S 651 Aeromonas media

0.00 46497 46497 S1 1208104 Aeromonas media WS

0.01 1647490 1647144 S 196024 Aeromonas dhakensis

0.00 346 346 S1 1156800 Aeromonas dhakensis AAK1

0.01 1642667 1642667 S 650 Aeromonas jandaei

0.01 1582568 1582568 S 73010 Aeromonas encheleia

0.00 1420944 1420944 S 29489 Aeromonas enteropelogenes

0.00 1387981 1387981 S 652 Aeromonas schubertii

0.00 1065344 1065344 S 648794 Aeromonas rivuli

0.00 1005211 1005211 S 218936 Aeromonas simiae

0.00 285862 285862 S 656 Aeromonas allosaccharophila

0.00 251599 251599 S 633415 Aeromonas sanarellii

0.00 238728 238728 S 948519 Aeromonas rivipollensis

0.00 8919 8919 S 646 Aeromonas sobria

0.00 926 926 S 633417 Aeromonas taiwanensis

0.00 52 52 S 105751 Aeromonas bestiarum

0.01 1949256 11197 G 225143 Oceanisphaera

0.00 1057635 1057635 S 1416627 Oceanisphaera profunda

0.00 880424 880424 S 1903694 Oceanisphaera avium

0.01 1568889 0 G 2802288 Dongshaea

0.01 1568889 1568889 S 2047966 Dongshaea marina

0.00 1172203 0 G 347533 Zobellella

0.00 1172203 1172203 S 347534 Zobellella denitrificans

0.00 1026702 0 G 43947 Tolumonas

0.00 1026702 0 S 43948 Tolumonas auensis

0.00 1026702 1026702 S1 595494 Tolumonas auensis DSM 9187

0.00 1008736 0 G 129577 Oceanimonas

0.00 1008736 0 G1 2636315 unclassified Oceanimonas

0.00 1008736 1008736 S 511062 Oceanimonas sp. GK1

0.01 1781119 0 F 83763 Succinivibrionaceae

0.01 1781119 0 G 83770 Succinivibrio

0.01 1781119 1781119 S 83771 Succinivibrio dextrinosolvens

0.18 53811145 27024 O 1706369 Cellvibrionales

0.07 22225859 14224 F 1706371 Cellvibrionaceae

0.03 8330215 9469 G 10 Cellvibrio

0.02 6895630 962545 G1 2624793 unclassified Cellvibrio

0.01 1711720 1711720 S 2303332 Cellvibrio sp. KY-GH-1

0.01 1651203 1651203 S 1987723 Cellvibrio sp. PSBB006

0.00 1410323 1410323 S 1945512 Cellvibrio sp. PSBB023

0.00 669665 669665 S 2964606 Cellvibrio sp. QJXJ

0.00 490174 490174 S 454662 Cellvibrio sp. KY-YJ-3

0.00 1425116 1423117 S 155077 Cellvibrio japonicus

0.00 1999 1999 S1 498211 Cellvibrio japonicus Ueda107

0.02 4615890 4486 G 2425 Teredinibacter

0.01 1630976 0 S 2426 Teredinibacter turnerae

0.01 1630976 1630976 S1 377629 Teredinibacter turnerae T7901

0.00 1515302 1515302 S 2731755 Teredinibacter haidensis

0.00 1465126 1465126 S 2731756 Teredinibacter purpureus

0.01 2580719 3552 G 447467 Simiduia

0.00 1341269 0 S 447471 Simiduia agarivorans

0.00 1341269 1341269 S1 1117647 Simiduia agarivorans SA1 = DSM 21679

0.00 1235898 0 G1 2639280 unclassified Simiduia

0.00 1235898 1235898 S 2909669 Simiduia sp. 21SJ11W-1

0.01 2206997 0 G 2036021 Agarilytica

0.01 2206997 2206997 S 1737490 Agarilytica rhodophyticola

0.01 1640592 0 G 1792291 Marinagarivorans

0.01 1640592 0 G1 2620290 unclassified Marinagarivorans

0.01 1640592 1640592 S 2721545 Marinagarivorans sp. GE09

0.01 1587019 0 G 316625 Saccharophagus

0.01 1587019 0 S 86304 Saccharophagus degradans

0.01 1587019 1587019 S1 203122 Saccharophagus degradans 2-40

0.00 1250203 0 G 940550 Gilvimarinus

0.00 1250203 0 G1 2642066 unclassified Gilvimarinus

0.00 1250203 1250203 S 2956798 Gilvimarinus sp. DA14

0.06 18934110 0 F 1706373 Microbulbiferaceae

0.06 18934110 342171 G 48073 Microbulbifer

0.03 9797374 66587 G1 2619833 unclassified Microbulbifer

0.00 1516136 1516136 S 359370 Microbulbifer sp. A4B17

0.00 1479545 1479545 S 2591606 Microbulbifer sp. GL-2

0.00 1383627 1383627 S 1516059 Microbulbifer sp. ALW1

0.00 1381603 1381603 S 2681547 Microbulbifer sp. SH-1

0.00 1354597 1354597 S 2587856 Microbulbifer sp. THAF38

0.00 1347682 1347682 S 2745199 Microbulbifer sp. YPW1

0.00 1267597 1267597 S 2904242 Microbulbifer sp. YPW16

0.00 1360232 1360232 S 266805 Microbulbifer variabilis

0.00 1293329 1293329 S 435905 Microbulbifer celer

0.00 1291017 1291017 S 260552 Microbulbifer agarilyticus

0.00 1236128 1236128 S 48074 Microbulbifer hydrolyticus

0.00 1234346 1234346 S 86173 Microbulbifer elongatus

0.00 1202673 1202673 S 252514 Microbulbifer thermotolerans

0.00 1176840 1176840 S 1769779 Microbulbifer aggregans

0.02 6396241 2839 F 1706372 Halieaceae

0.01 2647619 8517 G 1217416 Halioglobus

0.00 1360770 1360770 S 2601894 Halioglobus maricola

0.00 1278332 1278332 S 930805 Halioglobus japonicus

0.00 1437763 0 G 2678529 Kineobactrum

0.00 1437763 1437763 S 2708301 Kineobactrum salinum

0.00 1340290 0 G 393661 Congregibacter

0.00 1340290 0 S 393662 Congregibacter litoralis

0.00 1340290 1340290 S1 314285 Congregibacter litoralis KT71

0.00 967730 0 G 2847769 Aequoribacter

0.00 967730 967730 S 2518989 Aequoribacter fuscus

0.02 6227911 6499 F 1706375 Spongiibacteraceae

0.01 3508985 6821 G 630749 Spongiibacter

0.00 1273848 1273848 S 2794344 Spongiibacter nanhainus

0.00 1159663 1159663 S 1748242 Spongiibacter taiwanensis

0.00 1068653 0 G1 2631504 unclassified Spongiibacter

0.00 1068653 1068653 S 1620392 Spongiibacter sp. IMCC21906

0.00 1407321 0 G 1084558 Oceanicoccus

0.00 1407321 1407321 S 716816 Oceanicoccus sagamiensis

0.00 1305106 0 G 1434050 Zhongshania

0.00 1305106 1305106 S 1470434 Zhongshania aliphaticivorans

0.16 49649492 29898 O 135613 Chromatiales

0.07 20505431 98137 F 1046 Chromatiaceae

0.01 4438008 252153 G 1227 Nitrosococcus

0.00 1152820 0 S 133539 Nitrosococcus halophilus

0.00 1152820 1152820 S1 472759 Nitrosococcus halophilus Nc 4

0.00 1123272 1123272 S 1814290 Nitrosococcus wardiae

0.00 978948 7155 S 1229 Nitrosococcus oceani

0.00 971793 971793 S1 323261 Nitrosococcus oceani ATCC 19707

0.00 930815 0 S 473531 Nitrosococcus watsonii

0.00 930815 930815 S1 105559 Nitrosococcus watsonii C-113

0.01 3495095 2676 G 67575 Rheinheimera

0.01 2062554 245 G1 115860 unclassified Rheinheimera

0.00 1060225 1060225 S 1763998 Rheinheimera sp. F8

0.00 1002084 1002084 S 2545632 Rheinheimera sp. D18

0.00 1429865 1429865 S 2498451 Rheinheimera mangrovi

0.01 2242937 0 G 53392 Thiodictyon

0.01 2242937 2242937 S 1166950 Candidatus Thiodictyon syntrophicum

0.01 1874707 119738 G 85072 Allochromatium

0.00 933433 0 S 1049 Allochromatium vinosum

0.00 933433 933433 S1 572477 Allochromatium vinosum DSM 180

0.00 821536 821536 S 553982 Allochromatium tepidum

0.01 1685920 0 G 1056 Thiocapsa

0.01 1685920 1685920 S 521689 Thiocapsa bogorovii

0.00 1458199 0 G 13724 Thiocystis

0.00 1458199 0 S 73141 Thiocystis violascens

0.00 1458199 1458199 S1 765911 Thiocystis violascens DSM 198

0.00 1235816 0 G 156885 Thioflavicoccus

0.00 1235816 0 S 80679 Thioflavicoccus mobilis

0.00 1235816 1235816 S1 765912 Thioflavicoccus mobilis 8321

0.00 1083255 0 G 85076 Marichromatium

0.00 1083255 0 S 37487 Marichromatium purpuratum

0.00 1083255 1083255 S1 765910 Marichromatium purpuratum 984

0.00 880493 0 G 2828366 Caldichromatium

0.00 880493 880493 S 2699430 Caldichromatium japonicum

0.00 831888 0 G 85073 Thermochromatium

0.00 831888 0 S 1050 Thermochromatium tepidum

0.00 831888 831888 S1 316276 Thermochromatium tepidum ATCC 43061

0.00 628583 0 G 1980513 Candidatus Nitrosoglobus

0.00 628583 628583 S 1630141 Candidatus Nitrosoglobus terrae

0.00 552393 0 G 2796146 Candidatus Nitrosacidococcus

0.00 552393 552393 S 553981 Candidatus Nitrosacidococcus tergens

0.06 17453193 20037 F 72276 Ectothiorhodospiraceae

0.02 4928618 198836 G 106633 Thioalkalivibrio

0.00 1024549 0 S 186931 Thioalkalivibrio nitratireducens

0.00 1024549 1024549 S1 1255043 Thioalkalivibrio nitratireducens DSM 14787

0.00 1001462 0 S 1033854 Thioalkalivibrio sulfidiphilus

0.00 1001462 1001462 S1 396588 Thioalkalivibrio sulfidiphilus HL-EbGr7

0.00 965293 0 S 108010 Thioalkalivibrio paradoxus

0.00 965293 965293 S1 713585 Thioalkalivibrio paradoxus ARh 1

0.00 869329 0 G1 2621013 unclassified Thioalkalivibrio

0.00 869329 869329 S 396595 Thioalkalivibrio sp. K90mix

0.00 869149 869149 S 106634 Thioalkalivibrio versutus

0.01 3142757 16724 G 1765964 Acidihalobacter

0.00 1073336 1073336 S 2819280 Acidihalobacter yilgarnensis

0.00 1035158 1035158 S 2792603 Acidihalobacter aeolianus

0.00 1017539 1017539 S 1765967 Acidihalobacter ferrooxydans

0.01 2953645 6042 G 1335745 Spiribacter

0.01 1815351 795 G1 2629425 unclassified Spiribacter

0.00 1229144 1229144 S 2182432 Spiribacter sp. E85

0.00 585412 585412 S 2666185 Spiribacter sp. 2438

0.00 592734 592734 S 1335757 Spiribacter curvatus

0.00 539518 0 S 1335746 Spiribacter salinus

0.00 539518 539518 S1 1260251 Spiribacter salinus M19-40

0.01 1910552 179156 G 1051 Ectothiorhodospira

0.00 874815 0 G1 2684909 unclassified Ectothiorhodospira

0.00 874815 874815 S 1442136 Ectothiorhodospira sp. BSL-9

0.00 856581 856581 S 421628 Ectothiorhodospira haloalkaliphila

0.01 1685690 768 G 85108 Halorhodospira

0.00 871857 871857 S 1052 Halorhodospira halochloris

0.00 813065 0 S 1053 Halorhodospira halophila

0.00 813065 813065 S1 349124 Halorhodospira halophila SL1

0.00 1204853 0 G 406099 Aquisalimonas

0.00 1204853 0 G1 2644645 unclassified Aquisalimonas

0.00 1204853 1204853 S 2740807 Aquisalimonas sp. 2447

0.00 978638 0 G 133193 Alkalilimnicola

0.00 978638 0 S 351052 Alkalilimnicola ehrlichii

0.00 978638 978638 S1 187272 Alkalilimnicola ehrlichii MLHE-1

0.00 628403 0 G 233099 Ectothiorhodosinus

0.00 628403 628403 S 233100 Ectothiorhodosinus mongolicus

0.01 3408381 37 F 449719 Granulosicoccaceae

0.01 2434136 0 G 437504 Granulosicoccus

0.01 2434136 0 S 437505 Granulosicoccus antarcticus

0.01 2434136 2434136 S1 1192854 Granulosicoccus antarcticus IMCC3135

0.00 974208 0 G 1860077 Sulfuriflexus

0.00 974208 974208 S 1811807 Sulfuriflexus mobilis

0.01 2290433 0 F 1676141 Wenzhouxiangellaceae

0.01 2290433 1575 G 1676142 Wenzhouxiangella

0.00 1164009 0 G1 2613841 unclassified Wenzhouxiangella

0.00 1164009 1164009 S 2771012 Wenzhouxiangella sp. AB-CW3

0.00 1124849 1124849 S 1579979 Wenzhouxiangella marina

0.01 1691670 0 F 255526 Halothiobacillaceae

0.01 1691670 1410 G 109262 Halothiobacillus

0.00 891817 0 G1 2636392 unclassified Halothiobacillus

0.00 891817 891817 S 1860122 Halothiobacillus sp. LS2

0.00 798443 0 S 927 Halothiobacillus neapolitanus

0.00 798443 798443 S1 555778 Halothiobacillus neapolitanus c2

0.00 1314612 0 O1 451214 Chromatiales incertae sedis

0.00 1314612 697548 G 1273155 Thiohalobacter

0.00 356446 0 G1 2631224 unclassified Thiohalobacter

0.00 356446 356446 S 2795687 Thiohalobacter sp. COW1

0.00 260618 260618 S 585455 Thiohalobacter thiocyanaticus

0.00 1285357 0 F 1738654 Woeseiaceae

0.00 1285357 0 G 1738655 Woeseia

0.00 1285357 1285357 S 1548547 Woeseia oceani

0.00 921686 0 F 1096778 Thioalkalispiraceae

0.00 921686 0 G 2034504 Sulfurivermis

0.00 921686 921686 S 1972068 Sulfurivermis fontis

0.00 748831 0 F 2035710 Thioalkalibacteraceae

0.00 748831 0 G 2035712 Guyparkeria

0.00 748831 748831 S 47960 Guyparkeria halophila

0.15 46268884 0 O 135625 Pasteurellales

0.15 46268884 953467 F 712 Pasteurellaceae

0.03 10166782 1168287 G 724 Haemophilus

0.01 3627757 3479877 S 729 Haemophilus parainfluenzae

0.00 147880 147880 S1 862965 Haemophilus parainfluenzae T3T1

0.01 2213179 2144051 S 727 Haemophilus influenzae

0.00 26883 26883 S1 1232659 Haemophilus influenzae 2019

0.00 16768 16768 S1 725 Haemophilus influenzae biotype aegyptius

0.00 7458 7458 S1 862964 Haemophilus influenzae 10810

0.00 5856 5856 S1 281310 Haemophilus influenzae 86-028NP

0.00 4539 4539 S1 1295140 Haemophilus influenzae CGSHiCZ412602

0.00 3088 3088 S1 262727 Haemophilus influenzae R2846

0.00 2058 2058 S1 262728 Haemophilus influenzae R2866

0.00 1160 1160 S1 1334187 Haemophilus influenzae KR494

0.00 669 669 S1 935897 Haemophilus influenzae F3047

0.00 649 649 S1 866630 Haemophilus influenzae F3031

0.00 1029887 1029887 S 726 Haemophilus haemolyticus

0.00 711174 710465 S 730 [Haemophilus] ducreyi

0.00 709 709 S1 233412 [Haemophilus] ducreyi 35000HP

0.00 585856 585856 S 249188 Haemophilus pittmaniae

0.00 573916 573916 S 735 Haemophilus parahaemolyticus

0.00 233837 0 G1 2609962 unclassified Haemophilus

0.00 233837 233837 S 712310 Haemophilus sp. oral taxon 036

0.00 22889 22889 S 197575 Haemophilus aegyptius

0.02 6564793 1363466 G 745 Pasteurella

0.01 2987982 2212635 S 747 Pasteurella multocida

0.00 702753 120705 S1 44283 Pasteurella multocida subsp. multocida

0.00 558454 558454 S2 1304873 Pasteurella multocida subsp. multocida OH4807

0.00 7762 7762 S2 1455592 Pasteurella multocida subsp. multocida PMTB2.1

0.00 4739 4739 S2 1331042 Pasteurella multocida subsp. multocida P1062

0.00 3312 3312 S2 1132496 Pasteurella multocida subsp. multocida str. HN06

0.00 2658 2658 S2 584721 Pasteurella multocida subsp. multocida str. 3480

0.00 1658 1658 S2 1436299 Pasteurella multocida subsp. multocida str. HN07

0.00 1601 1601 S2 272843 Pasteurella multocida subsp. multocida str. Pm70

0.00 1095 1095 S2 1147130 Pasteurella multocida subsp. multocida str. HB03

0.00 769 769 S2 1339255 Pasteurella multocida subsp. multocida HB01

0.00 34477 34477 S1 115545 Pasteurella multocida subsp. septica

0.00 30053 28978 S1 123812 Pasteurella multocida subsp. gallicida

0.00 564 564 S2 1169409 Pasteurella multocida subsp. gallicida P1059

0.00 511 511 S2 1169410 Pasteurella multocida subsp. gallicida X73

0.00 5473 5473 S1 1304890 Pasteurella multocida OH1905

0.00 2591 2591 S1 1075089 Pasteurella multocida 36950

0.00 953133 953133 S 753 Pasteurella canis

0.00 574265 574265 S 2827233 Pasteurella atlantica

0.00 560294 560294 S 97481 Pasteurella skyensis

0.00 124452 124452 S 754 Pasteurella dagmatis

0.00 655 0 G1 2621516 unclassified Pasteurella

0.00 655 655 S 2844383 Pasteurella sp. XG20

0.00 546 546 S 760 Pasteurella stomatis

0.02 6380230 1100959 G 75984 Mannheimia

0.01 1615544 1603241 S 75985 Mannheimia haemolytica

0.00 4252 4252 S1 1249526 Mannheimia haemolytica USDA-ARS-USMARC-185

0.00 1728 1728 S1 1311760 Mannheimia haemolytica D174

0.00 1584 1584 S1 1261126 Mannheimia haemolytica D153

0.00 1564 1564 S1 1311759 Mannheimia haemolytica D171

0.00 1053 1053 S1 1366053 Mannheimia haemolytica USMARC_2286

0.00 714 714 S1 1316932 Mannheimia haemolytica M42548

0.00 708 708 S1 1249531 Mannheimia haemolytica USDA-ARS-USMARC-183

0.00 700 700 S1 1222034 Mannheimia haemolytica USDA-ARS-USMARC-184

0.00 1376612 1376612 S 85402 Mannheimia granulomatis

0.00 1139526 914429 S 85404 Mannheimia varigena

0.00 88851 88851 S1 1433287 Mannheimia varigena USDA-ARS-USMARC-1296

0.00 73628 73628 S1 1434214 Mannheimia varigena USDA-ARS-USMARC-1312

0.00 62618 62618 S1 1434215 Mannheimia varigena USDA-ARS-USMARC-1388

0.00 395807 194 G1 2645054 unclassified Mannheimia

0.00 359659 359659 S 1432056 Mannheimia sp. USDA-ARS-USMARC-1261

0.00 35954 35954 S 2679995 Mannheimia sp. ZY171111

0.00 392578 392578 S 2770636 Mannheimia bovis

0.00 208916 208916 S 111844 Mannheimia pernigra

0.00 150288 150288 S 2679994 Mannheimia ovis

0.02 5165951 747168 G 713 Actinobacillus

0.00 987606 896048 S 715 Actinobacillus pleuropneumoniae

0.00 28532 23700 S1 754345 Actinobacillus pleuropneumoniae serovar 8

0.00 4832 4832 S2 754257 Actinobacillus pleuropneumoniae serovar 8 str. 405

0.00 26949 0 S1 754347 Actinobacillus pleuropneumoniae serovar 10

0.00 26949 26949 S2 754259 Actinobacillus pleuropneumoniae serovar 10 str. D13039

0.00 18382 0 S1 754344 Actinobacillus pleuropneumoniae serovar 6

0.00 18382 18382 S2 754256 Actinobacillus pleuropneumoniae serovar 6 str. Femo

0.00 5317 0 S1 44294 Actinobacillus pleuropneumoniae serovar 5

0.00 5317 5317 S2 416269 Actinobacillus pleuropneumoniae serovar 5b str. L20

0.00 3875 0 S1 209841 Actinobacillus pleuropneumoniae serovar 7

0.00 3875 3875 S2 537457 Actinobacillus pleuropneumoniae serovar 7 str. AP76

0.00 3441 0 S1 434270 Actinobacillus pleuropneumoniae serovar 3

0.00 3441 3441 S2 434271 Actinobacillus pleuropneumoniae serovar 3 str. JL03

0.00 1255 0 S1 40325 Actinobacillus pleuropneumoniae serovar 1

0.00 1255 1255 S2 228399 Actinobacillus pleuropneumoniae serovar 1 str. 4074

0.00 1149 0 S1 754348 Actinobacillus pleuropneumoniae serovar 11

0.00 1149 1149 S2 754260 Actinobacillus pleuropneumoniae serovar 11 str. 56153

0.00 977 0 S1 754346 Actinobacillus pleuropneumoniae serovar 9

0.00 977 977 S2 754258 Actinobacillus pleuropneumoniae serovar 9 str. CVJ13261

0.00 919 0 S1 34063 Actinobacillus pleuropneumoniae serovar 2

0.00 919 919 S2 871925 Actinobacillus pleuropneumoniae serovar 2 str. S1536

0.00 762 0 S1 754343 Actinobacillus pleuropneumoniae serovar 4

0.00 762 762 S2 754255 Actinobacillus pleuropneumoniae serovar 4 str. M62

0.00 682390 0 G1 2644856 unclassified Actinobacillus

0.00 682390 682390 S 2774188 Actinobacillus sp. GY-402

0.00 600222 600222 S 189834 Actinobacillus porcitonsillarum

0.00 537485 537485 S 51161 Actinobacillus delphinicola

0.00 488886 488886 S 51049 Actinobacillus indolicus

0.00 468400 461266 S 716 Actinobacillus suis

0.00 6034 6034 S1 696748 Actinobacillus suis H91-0380

0.00 1100 1100 S1 743972 Actinobacillus suis ATCC 33415

0.00 439694 438506 S 718 Actinobacillus equuli

0.00 1188 1188 S1 202947 Actinobacillus equuli subsp. equuli

0.00 214100 214100 S 720 Actinobacillus lignieresii

0.01 4230749 450633 G 416916 Aggregatibacter

0.00 1364967 1325836 S 714 Aggregatibacter actinomycetemcomitans

0.00 18791 18791 S1 1407647 Aggregatibacter actinomycetemcomitans NUM4039

0.00 14356 14356 S1 668336 Aggregatibacter actinomycetemcomitans D11S-1

0.00 4578 4578 S1 694569 Aggregatibacter actinomycetemcomitans D7S-1

0.00 1406 1406 S1 272556 Aggregatibacter actinomycetemcomitans HK1651

0.00 1184467 928264 S 732 Aggregatibacter aphrophilus

0.00 157796 157796 S1 634176 Aggregatibacter aphrophilus NJ8700

0.00 98407 98407 S1 985008 Aggregatibacter aphrophilus ATCC 33389

0.00 990050 86549 G1 2639383 unclassified Aggregatibacter

0.00 398033 398033 S 2820817 Aggregatibacter sp. 2125159857

0.00 265533 265533 S 2866570 Aggregatibacter sp. Marseille-P9115

0.00 239935 239935 S 712150 Aggregatibacter sp. oral taxon 513

0.00 240632 239574 S 739 Aggregatibacter segnis

0.00 1058 1058 S1 888057 Aggregatibacter segnis ATCC 33393

0.01 2636714 118646 G 292486 Avibacterium

0.00 1036861 1036861 S 728 Avibacterium paragallinarum

0.00 950914 392715 G1 2685287 unclassified Avibacterium

0.00 302640 302640 S 2911526 Avibacterium sp. 20-132

0.00 255559 255559 S 2911527 Avibacterium sp. 21-595

0.00 530293 530293 S 762 Avibacterium volantium

0.01 2397377 11997 G 2094023 Glaesserella

0.01 1734156 1641876 S 738 Glaesserella parasuis

0.00 65764 65764 S1 1275971 Glaesserella parasuis D74

0.00 13211 13211 S1 557723 Glaesserella parasuis SH0165

0.00 12317 12317 S1 456298 Glaesserella parasuis 29755

0.00 988 988 S1 1117322 Glaesserella parasuis str. Nagasaki

0.00 651224 0 G1 2629322 unclassified Glaesserella

0.00 651224 651224 S 2030797 Glaesserella sp. 15-184

0.00 1477299 3464 G 1960084 Rodentibacter

0.00 752945 752945 S 1906744 Rodentibacter heylii

0.00 720890 720890 S 2778911 Rodentibacter haemolyticus

0.00 940071 0 G 476528 Bibersteinia

0.00 940071 708408 S 47735 Bibersteinia trehalosi

0.00 177489 177489 S1 1263832 Bibersteinia trehalosi USDA-ARS-USMARC-190

0.00 42525 42525 S1 1263829 Bibersteinia trehalosi USDA-ARS-USMARC-188

0.00 6990 6990 S1 1263831 Bibersteinia trehalosi USDA-ARS-USMARC-189

0.00 4659 4659 S1 1171377 Bibersteinia trehalosi USDA-ARS-USMARC-192

0.00 901287 0 G 214906 Histophilus

0.00 901287 900153 S 731 Histophilus somni

0.00 1134 1134 S1 205914 Histophilus somni 129PT

0.00 814394 512148 G 697331 Basfia

0.00 159489 0 S 157673 [Mannheimia] succiniciproducens

0.00 159489 159489 S1 221988 [Mannheimia] succiniciproducens MBEL55E

0.00 142757 142757 S 653940 Basfia succiniciproducens

0.00 759687 0 G 155493 Gallibacterium

0.00 759687 0 S 750 Gallibacterium anatis

0.00 759687 759687 S1 1005058 Gallibacterium anatis UMN179

0.00 621460 0 G 1649317 Frederiksenia

0.00 621460 621460 S 123824 Frederiksenia canicola

0.00 598964 0 G 109471 Bisgaardia

0.00 598964 598964 S 109472 Bisgaardia hudsonensis

0.00 583735 0 G 1249016 Otariodibacter

0.00 583735 583735 S 1032623 Otariodibacter oris

0.00 549999 0 G 2899790 Mergibacter

0.00 549999 549999 S 221402 Mergibacter septicus

0.00 525903 0 G 1520333 Vespertiliibacter

0.00 525903 525903 S 1443036 Vespertiliibacter pulmonis

0.00 22 0 F1 1524964 Pasteurellaceae incertae sedis

0.00 22 0 F2 310966 [Pasteurella] aerogenes-[Pasteurella] mairii-[Actinobacillus] rossii complex

0.00 22 22 S 749 [Pasteurella] aerogenes

0.12 36239948 1669 O 118969 Legionellales

0.10 31266995 202459 F 444 Legionellaceae

0.09 28857484 798026 G 445 Legionella

0.02 5835420 4785860 S 446 Legionella pneumophila

0.00 497192 497192 S1 91890 Legionella pneumophila subsp. pascullei

0.00 332720 310138 S1 91891 Legionella pneumophila subsp. pneumophila

0.00 18609 18609 S2 1312904 Legionella pneumophila subsp. pneumophila LPE509

0.00 3018 3018 S2 1199191 Legionella pneumophila subsp. pneumophila str. Thunder Bay

0.00 955 955 S2 272624 Legionella pneumophila subsp. pneumophila str. Philadelphia 1

0.00 149445 149445 S1 91892 Legionella pneumophila subsp. fraseri

0.00 32951 0 S1 66976 Legionella pneumophila serogroup 1

0.00 32951 32951 S2 423212 Legionella pneumophila 2300/99 Alcoy

0.00 31235 31235 S1 400673 Legionella pneumophila str. Corby

0.00 3515 3515 S1 297245 Legionella pneumophila str. Lens

0.00 1362 1362 S1 297246 Legionella pneumophila str. Paris

0.00 1140 0 S1 66987 Legionella pneumophila serogroup 12

0.00 1140 1140 S2 933093 Legionella pneumophila subsp. pneumophila ATCC 43290

0.01 3111185 4744 G1 2622702 unclassified Legionella

0.00 1113271 1113271 S 2755562 Legionella sp. PC997

0.00 1039404 1039404 S 2746060 Legionella sp. PC1000

0.00 953766 953766 S 2662448 Legionella sp. MW5194

0.01 2050893 2050893 S 28087 Legionella sainthelensi

0.00 1410215 1410215 S 454 Legionella israelensis

0.00 1406100 1391567 S 450 Legionella longbeachae

0.00 14533 14533 S1 661367 Legionella longbeachae NSW150

0.00 1319520 0 S 96230 Legionella fallonii

0.00 1319520 1319520 S1 1212491 Legionella fallonii LLAP-10

0.00 1294988 1294988 S 452 Legionella spiritensis

0.00 1106002 1106002 S 96232 Legionella lytica

0.00 1103442 1103442 S 28082 Legionella anisa

0.00 1084703 1084703 S 28084 Legionella cherrii

0.00 1051753 1051753 S 66969 Legionella waltersii

0.00 1032994 1032994 S 449 Legionella hackeliae

0.00 986326 986326 S 2708020 Legionella antarctica

0.00 953354 953354 S 1867846 Legionella clemsonensis

0.00 932828 932828 S 456 Legionella jordanis

0.00 928252 928252 S 45065 Legionella geestiana

0.00 897286 897286 S 45067 Legionella lansingensis

0.00 820901 0 S 29423 Legionella oakridgensis

0.00 820901 820901 S1 1268635 Legionella oakridgensis ATCC 33761 = DSM 21215

0.00 629290 629290 S 45056 Legionella adelaidensis

0.00 104006 104006 S 2005262 Legionella endosymbiont of Polyplax serrata

0.00 1143078 0 G 461 Fluoribacter

0.00 1143078 1005933 S 463 Fluoribacter dumoffii

0.00 77658 77658 S1 1094714 Fluoribacter dumoffii Tex-KL

0.00 59487 59487 S1 1094715 Fluoribacter dumoffii NY 23

0.00 1063974 0 G 465 Tatlockia

0.00 1063974 1063974 S 451 Tatlockia micdadei

0.02 4971284 195482 F 118968 Coxiellaceae

0.01 1830637 752 G 776 Coxiella

0.00 871385 788429 S 777 Coxiella burnetii

0.00 53308 53308 S1 434922 Coxiella burnetii Dugway 5J108-111

0.00 12868 12868 S1 1321945 Coxiella burnetii str. Namibia

0.00 8056 8056 S1 1293501 Coxiella burnetii Z3055

0.00 4051 4051 S1 1401251 Coxiella burnetii str. Schperling

0.00 1335 1335 S1 434923 Coxiella burnetii CbuG_Q212

0.00 911 911 S1 360116 Coxiella burnetii 'MSU Goat Q177'

0.00 890 890 S1 434924 Coxiella burnetii CbuK_Q154

0.00 801 801 S1 360115 Coxiella burnetii RSA 331

0.00 736 736 S1 227377 Coxiella burnetii RSA 493

0.00 492236 34 G1 2676648 unclassified Coxiella (in: Bacteria)

0.00 301772 301772 S 2749996 Coxiella endosymbiont of Amblyomma nuttalli

0.00 187409 187409 S 2487929 Coxiella endosymbiont of Amblyomma sculptum

0.00 3021 3021 S 325775 Coxiella endosymbiont of Amblyomma americanum

0.00 466264 466264 S 2054173 Candidatus Coxiella mudrowiae

0.01 1666576 703 G 254245 Aquicella

0.00 886752 886752 S 254247 Aquicella siphonis

0.00 779121 779121 S 254246 Aquicella lusitana

0.00 973055 38062 G 59195 Rickettsiella

0.00 511446 0 G1 2637345 unclassified Rickettsiella

0.00 511446 511446 S 2856608 Rickettsiella endosymbiont of Dermanyssus gallinae

0.00 423547 423547 S 676208 Candidatus Rickettsiella viridis

0.00 305534 1 F1 134284 unclassified Coxiellaceae

0.00 302515 302515 S 1592897 Coxiella-like endosymbiont

0.00 3018 3018 S 1987500 Coxiella-like endosymbiont of Amblyomma americanum

0.11 34088603 1702 O 72273 Thiotrichales

0.05 16484566 442434 F 135616 Piscirickettsiaceae

0.01 3979699 6119 G 2039723 Thiomicrorhabdus

0.00 870166 870166 S 2267253 Thiomicrorhabdus indica

0.00 801372 801372 S 2739063 Thiomicrorhabdus xiamenensis

0.00 787246 787246 S 2211106 Thiomicrorhabdus aquaedulcis

0.00 783501 783501 S 2791037 Thiomicrorhabdus immobilis

0.00 731295 731295 S 2580412 Thiomicrorhabdus sediminis

0.01 2955357 29110 G 40222 Methylophaga

0.00 1131964 1131964 S 754476 Methylophaga nitratireducenticrescens

0.00 972273 972273 S 2881052 Methylophaga pinxianii

0.00 821353 821353 S 754477 Methylophaga frappieri

0.00 657 657 S 40223 Methylophaga thalassica

0.01 2389079 22293 G 933 Thiomicrospira

0.00 736912 736912 S 406020 Thiomicrospira microaerophila

0.00 654770 0 S 92245 Thiomicrospira aerophila

0.00 654770 654770 S1 717772 Thiomicrospira aerophila AL3

0.00 598896 0 S 147268 Thiomicrospira cyclica

0.00 598896 598896 S1 717773 Thiomicrospira cyclica ALM1

0.00 376208 0 G1 2643099 unclassified Thiomicrospira

0.00 376208 376208 S 1803865 Thiomicrospira sp. S5

0.01 2050409 0 G 1237 Piscirickettsia

0.01 2050409 2047869 S 1238 Piscirickettsia salmonis

0.00 2540 2540 S1 1227812 Piscirickettsia salmonis LF-89 = ATCC VR-1361

0.01 1914197 2431 G 28884 Hydrogenovibrio

0.00 770809 770809 S 28885 Hydrogenovibrio marinus

0.00 749918 749918 S 39765 Hydrogenovibrio crunogenus

0.00 391039 391039 S 265883 Hydrogenovibrio thermophilus

0.00 1116340 609466 G 34067 Cycloclasticus

0.00 307996 69059 G1 2621467 unclassified Cycloclasticus

0.00 131670 131670 S 728003 Cycloclasticus sp. PY97N

0.00 107267 107267 S 385025 Cycloclasticus sp. P1

0.00 198878 0 S 1329899 Cycloclasticus zancles

0.00 198878 198878 S1 1198232 Cycloclasticus zancles 78-ME

0.00 819829 0 G 2781120 Thiosulfativibrio

0.00 819829 819829 S 2675053 Thiosulfativibrio zosterae

0.00 817222 0 G 2781121 Thiosulfatimonas

0.00 817222 817222 S 2675054 Thiosulfatimonas sediminis

0.03 10559595 27296 F 34064 Francisellaceae

0.03 9285511 2090154 G 262 Francisella

0.00 1468110 2965 G1 2610885 unclassified Francisella

0.00 566161 566161 S 1395624 Francisella sp. LA112445

0.00 565994 565994 S 1547445 Francisella sp. FSC1006

0.00 331331 331331 S 2589986 Francisella sp. Scap27

0.00 1659 1659 S 475375 Francisella sp. MA067296

0.00 1077413 498686 S 263 Francisella tularensis

0.00 531059 359797 S1 264 Francisella tularensis subsp. novicida

0.00 64103 64103 S2 1386968 Francisella tularensis subsp. novicida PA10-7858

0.00 40630 40630 S2 984129 Francisella cf. novicida Fx1

0.00 33092 33092 S2 1450527 Francisella tularensis subsp. novicida D9876

0.00 30933 30933 S2 401614 Francisella tularensis subsp. novicida U112

0.00 2504 2504 S2 1452728 Francisella tularensis subsp. novicida F6168

0.00 34481 23674 S1 119857 Francisella tularensis subsp. holarctica

0.00 6484 6484 S2 1432652 Francisella tularensis subsp. holarctica PHIT-FT049

0.00 1374 1374 S2 1232394 Francisella tularensis subsp. holarctica F92

0.00 1100 1100 S2 393011 Francisella tularensis subsp. holarctica OSU18

0.00 881 881 S2 376619 Francisella tularensis subsp. holarctica LVS

0.00 495 495 S2 458234 Francisella tularensis subsp. holarctica FTNF002-00

0.00 473 473 S2 351581 Francisella tularensis subsp. holarctica FSC200

0.00 13187 6012 S1 119856 Francisella tularensis subsp. tularensis

0.00 2020 1124 S2 177416 Francisella tularensis subsp. tularensis SCHU S4

0.00 896 896 S3 1341656 Francisella tularensis subsp. tularensis str. SCHU S4 substr. NR-28534

0.00 1539 700 S2 1001534 Francisella tularensis subsp. tularensis TI0902

0.00 839 839 S3 1001542 Francisella tularensis subsp. tularensis TIGB03

0.00 1272 1272 S2 1133671 Francisella tularensis subsp. tularensis WY-00W4114

0.00 723 723 S2 543737 Francisella tularensis subsp. tularensis MA00-2987

0.00 624 624 S2 510831 Francisella tularensis subsp. tularensis NE061598

0.00 514 514 S2 393115 Francisella tularensis subsp. tularensis FSC198

0.00 483 483 S2 418136 Francisella tularensis subsp. tularensis WY96-3418

0.00 962454 860668 S 28110 Francisella philomiragia

0.00 101681 101681 S1 539329 Francisella philomiragia subsp. philomiragia ATCC 25015

0.00 105 105 S1 484022 Francisella philomiragia subsp. philomiragia ATCC 25017

0.00 573070 573070 S 573570 Francisella uliginis

0.00 560594 560594 S 549298 Francisella halioticida

0.00 546128 396955 S 622488 Francisella hispaniensis

0.00 149173 149173 S1 1088883 Francisella hispaniensis FSC454

0.00 534121 534121 S 1542390 Francisella frigiditurris

0.00 377600 0 S 954 Francisella persica

0.00 377600 377600 S1 1086726 Francisella persica ATCC VR-331

0.00 356822 356822 S 2007306 Francisella adeliensis

0.00 255699 250743 S 299583 Francisella orientalis

0.00 2571 2571 S1 1163389 Francisella orientalis str. Toba 04

0.00 927 927 S1 1356861 Francisella orientalis LADL 07-285A

0.00 800 800 S1 1449091 Francisella orientalis FNO24

0.00 658 658 S1 1390363 Francisella orientalis FNO12

0.00 152848 0 S 657445 Francisella noatunensis

0.00 152848 0 S1 360196 Francisella noatunensis subsp. noatunensis

0.00 152848 152848 S2 1089434 Francisella noatunensis subsp. noatunensis FSC774

0.00 130455 130455 S 573569 Francisella salina

0.00 117940 117940 S 2249302 Francisella marina

0.00 75344 75344 S 2599927 Francisella salimarina

0.00 6759 6759 S 2016517 Francisella opportunistica

0.00 1246788 170721 G 1869285 Allofrancisella

0.00 426536 426536 S 594679 Allofrancisella guangzhouensis

0.00 333101 333101 S 1085647 Allofrancisella inopinata

0.00 316430 316430 S 1085644 Allofrancisella frigidaquae

0.02 7028920 49 F 135617 Thiotrichaceae

0.02 5712896 469322 G 1030 Thiothrix

0.00 1247349 1247349 S 2891210 Thiothrix litoralis

0.00 1139684 1139684 S 96472 Thiothrix winogradskyi

0.00 1075711 1075711 S 2735563 Thiothrix subterranea

0.00 889796 889796 S 111769 Thiothrix unzii

0.00 836087 836087 S 2823902 Candidatus Thiothrix anitrata

0.00 54947 54947 S 111770 Thiothrix fructosivorans

0.00 1315975 0 G 1021 Beggiatoa

0.00 1315975 1315975 S 288004 Beggiatoa leptomitoformis

0.00 13820 0 F 2056687 Fastidiosibacteraceae

0.00 13820 0 G 28905 Caedibacter

0.00 13820 13820 S 28907 Caedibacter taeniospiralis

0.10 30779057 0 O 135618 Methylococcales

0.10 30779057 321882 F 403 Methylococcaceae

0.04 12177525 1205213 G 416 Methylomonas

0.01 4273291 664831 G1 2608980 unclassified Methylomonas

0.00 1472875 1472875 S 2785785 Methylomonas sp. LL1

0.00 859124 859124 S 107637 Methylomonas sp. LW13

0.00 785790 785790 S 2812647 Methylomonas sp. EFPC1

0.00 490671 490671 S 1727196 Methylomonas sp. DH-1

0.00 1518057 0 S 421 Methylomonas methanica

0.00 1518057 1518057 S1 857087 Methylomonas methanica MC09

0.00 1450422 1450422 S 1538553 Methylomonas denitrificans

0.00 1388668 1388668 S 2608981 Methylomonas rhizoryzae

0.00 1240444 1240444 S 702114 Methylomonas koyamae

0.00 1101430 1101430 S 1173101 Methylomonas paludis

0.02 4766356 197399 G 413 Methylococcus

0.01 2387796 722 G1 2618889 unclassified Methylococcus

0.00 1387709 1387709 S 2812648 Methylococcus sp. EFPC2

0.00 999365 999365 S 2860258 Methylococcus sp. Mc7

0.00 1307600 1207218 S 414 Methylococcus capsulatus

0.00 100382 100382 S1 243233 Methylococcus capsulatus str. Bath

0.00 873561 873561 S 2681310 Methylococcus geothermalis

0.01 2588952 266 G 39773 Methylomicrobium

0.00 1342573 0 S 39775 Methylomicrobium album

0.00 1342573 1342573 S1 686340 Methylomicrobium album BG8

0.00 1246113 0 G1 2631527 unclassified Methylomicrobium

0.00 1246113 1246113 S 2049332 Methylomicrobium sp. wino1

0.01 2021305 464067 G 2822410 Methylotuvimicrobium

0.00 815167 815167 S 95641 Methylotuvimicrobium buryatense

0.00 742071 0 S 271065 Methylotuvimicrobium alcaliphilum

0.00 742071 742071 S1 1091494 Methylotuvimicrobium alcaliphilum 20Z

0.01 1782886 0 G 73778 Methylocaldum

0.01 1782886 1782886 S 1432792 Methylocaldum marinum

0.01 1557400 0 G 1760987 Methylomagnum

0.01 1557400 1557400 S 1760988 Methylomagnum ishizawai

0.00 1518961 0 G 762296 Methylovulum

0.00 1518961 1518961 S 1704499 Methylovulum psychrotolerans

0.00 1418913 0 G 429 Methylobacter

0.00 1418913 0 G1 2635283 unclassified Methylobacter

0.00 1418913 1418913 S 2839024 Methylobacter sp. S3L5C

0.00 1408059 0 G 1808977 Candidatus Methylospira

0.00 1408059 1408059 S 1808979 Candidatus Methylospira mobilis

0.00 1216818 0 G 1295378 Methylogaea

0.00 1216818 1216818 S 1295382 Methylogaea oryzae

0.05 13901205 7552 C1 118884 Gammaproteobacteria incertae sedis

0.01 3383463 10339 C2 32036 sulfur-oxidizing symbionts

0.00 1112154 0 G 393764 Candidatus Endoriftia

0.00 1112154 1112154 S 393765 Candidatus Endoriftia persephone

0.00 733258 733258 S 2360 Bathymodiolus thermophilus thioautotrophic gill symbiont

0.00 582104 7755 G 1541743 Candidatus Ruthia

0.00 297962 0 S 386487 Candidatus Ruthia magnifica

0.00 297962 297962 S1 413404 Candidatus Ruthia magnifica str. Cm (Calyptogena magnifica)

0.00 276387 276387 S 2738852 Candidatus Ruthia endofausta

0.00 507178 74899 G 2732587 Candidatus Vesicomyosocius

0.00 217277 0 G1 2763321 unclassified Candidatus Vesicomyosocius

0.00 217277 217277 S 2732590 Candidatus Vesicomyosocius sp. SY067_SCS001

0.00 215002 215002 S 412965 Candidatus Vesicomyosocius okutanii

0.00 438430 0 S 113267 Bathymodiolus septemdierum thioautotrophic gill symbiont

0.00 438430 438430 S1 1303921 endosymbiont of Bathymodiolus septemdierum str. Myojin knoll

0.00 1273127 0 G 745410 Gallaecimonas

0.00 1273127 1273127 S 2291597 Gallaecimonas mangrovi

0.00 1239900 0 F 412032 Celerinatantimonadaceae

0.00 1239900 0 G 412033 Celerinatantimonas

0.00 1239900 1239900 S 412034 Celerinatantimonas diazotrophica

0.00 1237922 0 G 349742 Sedimenticola

0.00 1237922 1237922 S 1543721 Sedimenticola thiotaurini

0.00 1057018 0 G 1524249 Pseudohongiella

0.00 1057018 1057018 S 1249552 Pseudohongiella spirulinae

0.00 1055925 0 G 2738850 Candidatus Reidiella

0.00 1055925 1055925 S 2738883 Candidatus Reidiella endopervernicosa

0.00 983875 0 G 1608298 Thiolapillus

0.00 983875 983875 S 1076588 Thiolapillus brandeum

0.00 963767 67 G 655184 Candidatus Thioglobus

0.00 506623 0 G1 2644172 unclassified Candidatus Thioglobus

0.00 506623 506623 S 2508687 Candidatus Thioglobus sp. NP1

0.00 457077 457077 S 1705394 Candidatus Thioglobus autotrophicus

0.00 923509 0 G 112008 Ignatzschineria

0.00 923509 0 G1 2644162 unclassified Ignatzschineria

0.00 923509 923509 S 2923279 Ignatzschineria sp. HR5S32

0.00 642503 0 G 2841677 Candidatus Pseudothioglobus

0.00 642503 491306 S 1427364 Candidatus Pseudothioglobus singularis

0.00 151197 151197 S1 1125411 Candidatus Pseudothioglobus singularis PS1

0.00 600762 0 G 198346 Candidatus Baumannia

0.00 600762 401729 S 186490 Candidatus Baumannia cicadellinicola

0.00 199033 199033 S1 374463 Baumannia cicadellinicola str. Hc (Homalodisca coagulata)

0.00 413185 0 G 1076625 Candidatus Steffania

0.00 413185 413185 S 1076626 Candidatus Steffania adelgidicola

0.00 63133 229 G 204619 Candidatus Nardonella

0.00 23470 23470 S 1972134 endosymbiont of Sipalinus gigas

0.00 22109 22109 S 1971488 endosymbiont of Pachyrhynchus infernalis

0.00 17325 15602 S 1971485 Candidatus Nardonella dryophthoridicola

0.00 1723 1723 S1 1972133 endosymbiont of Rhynchophorus ferrugineus

0.00 55564 0 G 2817383 Candidatus Azoamicus

0.00 55564 55564 S 2652803 Candidatus Azoamicus ciliaticola

0.02 5390498 0 O 1240482 Orbales

0.02 5390498 13500 F 1240483 Orbaceae

0.01 3789017 20474 G 1193503 Gilliamella

0.01 2256685 50142 G1 2685620 unclassified Gilliamella

0.00 778607 778607 S 2704653 Gilliamella sp. ESL0405

0.00 765392 765392 S 2704654 Gilliamella sp. ESL0441

0.00 662544 662544 S 2704655 Gilliamella sp. ESL0443

0.00 1511858 1511858 S 1196095 Gilliamella apicola

0.00 815070 0 G 2894762 Zophobihabitans

0.00 815070 815070 S 1635327 Zophobihabitans entericus

0.00 772911 0 G 1335631 Frischella

0.00 772911 772911 S 1267021 Frischella perrara

0.02 4655280 114 O 1775403 Nevskiales

0.01 3619590 2958 F 568386 Sinobacteraceae

0.01 1582676 0 G 413435 Solimonas

0.01 1582676 0 G1 2637139 unclassified Solimonas

0.01 1582676 1582676 S 2303331 Solimonas sp. K1W22B-7

0.00 1101708 0 G 2893022 Flagellatimonas

0.00 1101708 1101708 S 2806210 Flagellatimonas centrodinii

0.00 932248 0 G 1861863 Sinimarinibacterium

0.00 932248 0 G1 2621506 unclassified Sinimarinibacterium

0.00 932248 932248 S 2698684 Sinimarinibacterium sp. NLF-5-8

0.00 1035576 0 F 2689614 Steroidobacteraceae

0.00 1035576 0 G 469322 Steroidobacter

0.00 1035576 1035576 S 465721 Steroidobacter denitrificans

0.01 3913650 0 O 1692040 Acidiferrobacterales

0.01 3913650 1627 F 1692041 Acidiferrobacteraceae

0.01 1863911 143319 G 986106 Acidiferrobacter

0.00 865948 865948 S 163359 Acidiferrobacter thiooxydans

0.00 854644 0 G1 2640868 unclassified Acidiferrobacter

0.00 854644 854644 S 1281578 Acidiferrobacter sp. SPIII_3

0.00 1184829 0 G 1692042 Sulfurifustis

0.00 1184829 1184829 S 1675686 Sulfurifustis variabilis

0.00 863283 0 G 1744881 Sulfuricaulis

0.00 863283 863283 S 1620215 Sulfuricaulis limicola

0.01 3270090 0 O 2887327 Kangiellales

0.01 3270090 0 F 1920240 Kangiellaceae

0.01 3270090 26332 G 261963 Kangiella

0.00 875795 0 S 261964 Kangiella koreensis

0.00 875795 875795 S1 523791 Kangiella koreensis DSM 16069

0.00 812311 812311 S 1561924 Kangiella profundi

0.00 779143 779143 S 1144748 Kangiella sediminilitoris

0.00 776509 776509 S 914150 Kangiella geojedonensis

0.01 2596718 0 O 135615 Cardiobacteriales

0.01 2596718 445 F 868 Cardiobacteriaceae

0.01 1547008 25451 G 2717 Cardiobacterium

0.00 761797 761797 S 2718 Cardiobacterium hominis

0.00 759760 0 G1 2648856 unclassified Cardiobacterium

0.00 759760 759760 S 2866573 Cardiobacterium sp. Marseille-Q4385

0.00 620439 0 G 13275 Suttonella

0.00 620439 0 G1 2908647 unclassified Suttonella

0.00 620439 620439 S 2908648 Suttonella sp. R2A3

0.00 428826 0 G 869 Dichelobacter

0.00 428826 398853 S 870 Dichelobacter nodosus

0.00 29973 29973 S1 246195 Dichelobacter nodosus VCS1703A

0.00 1488168 33830 C1 33811 unclassified Gammaproteobacteria

0.00 762771 762771 S 1248727 endosymbiont of unidentified scaly snail isolate Monju

0.00 355551 0 S 596095 Abyssogena phaseoliformis symbiont

0.00 355551 355551 S1 1235283 Abyssogena phaseoliformis symbiont OG214

0.00 316513 0 S 883811 Isorropodon fossajaponicum symbiont

0.00 316513 316513 S1 1235284 Isorropodon fossajaponicum endosymbiont JTNG4

0.00 19503 19503 S 650377 endosymbiont of Euscepes postfasciatus

0.00 1243218 0 O 742030 Salinisphaerales

0.00 1243218 0 F 742031 Salinisphaeraceae

0.00 1243218 0 G 180541 Salinisphaera

0.00 1243218 0 G1 2649847 unclassified Salinisphaera

0.00 1243218 1243218 S 2183911 Salinisphaera sp. LB1

0.00 926990 0 O 1934945 Immundisolibacterales

0.00 926990 0 F 1934946 Immundisolibacteraceae

0.00 926990 0 G 1934947 Immundisolibacter

0.00 926990 926990 S 1810504 Immundisolibacter cernigliae

0.00 314965 0 O 2975284 Candidatus Comchoanobacterales

0.00 314965 0 F 2975285 Candidatus Comchoanobacteraceae

0.00 314965 0 G 2975286 Candidatus Comchoanobacter

0.00 314965 314965 S 2919598 Candidatus Comchoanobacter bicostacola

4.46 1363063090 5345827 C 28211 Alphaproteobacteria

2.34 715877730 4652291 O 356 Hyphomicrobiales

0.66 202221230 1416874 F 82115 Rhizobiaceae

0.50 151282893 3501718 F1 227290 Rhizobium/Agrobacterium group

0.33 101008798 23515717 G 379 Rhizobium

0.10 31077973 5756476 G1 2613769 unclassified Rhizobium

0.01 1877429 1877429 S 2775403 Rhizobium sp. TH2

0.01 1845470 1845470 S 2849093 Rhizobium sp. WYJ-E13

0.01 1694651 1694651 S 2613770 Rhizobium sp. BG4

0.01 1543515 1543515 S 1571470 Rhizobium sp. ACO-34A

0.00 1505705 1505705 S 2028343 Rhizobium sp. 11515TR

0.00 1442710 1442710 S 2806346 Rhizobium sp. SL42

0.00 1433846 1433846 S 2603277 Rhizobium sp. WL3

0.00 1389845 1389845 S 2795216 Rhizobium sp. AB2/73

0.00 1286012 1286012 S 1223565 Rhizobium sp. Pop5

0.00 1188806 1188806 S 2731096 Rhizobium sp. NZLR1

0.00 1073993 1073993 S 2020312 Rhizobium sp. CIAT894

0.00 1042197 1042197 S 1981173 Rhizobium sp. NXC14

0.00 1024865 1024865 S 2917727 Rhizobium sp. C104

0.00 948991 948991 S 2731112 Rhizobium sp. NLR16a

0.00 827753 827753 S 2918527 Rhizobium sp. K102

0.00 774911 774911 S 2819999 Rhizobium sp. L51/94

0.00 628361 628361 S 2815360 Rhizobium sp. X9

0.00 591749 591749 S 2785056 Rhizobium sp. 007

0.00 524213 524213 S 2048897 Rhizobium sp. NXC24

0.00 479450 479450 S 1301032 Rhizobium sp. IE4771

0.00 407869 407869 S 2020311 Rhizobium sp. Kim5

0.00 333385 333385 S 2590777 Rhizobium sp. NIBRBAC000502774

0.00 300625 300625 S 2020313 Rhizobium sp. TAL182

0.00 280613 280613 S 2291939 Rhizobium sp. ZX09

0.00 217845 217845 S 2819994 Rhizobium sp. B230/85

0.00 126773 126773 S 1914541 Rhizobium sp. Y9

0.00 118218 118218 S 1703969 Rhizobium sp. N324

0.00 92458 92458 S 2364271 Rhizobium sp. CCGE531

0.00 89004 89004 S 2895568 Rhizobium sp. RCAM05350

0.00 80370 80370 S 2819993 Rhizobium sp. B21/90

0.00 61045 61045 S 555319 Rhizobium sp. T136

0.00 39348 39348 S 2364272 Rhizobium sp. CCGE532

0.00 15765 15765 S 1703960 Rhizobium sp. N113

0.00 11002 11002 S 1869170 Rhizobium sp. S41

0.00 7171 7171 S 2613771 Rhizobium sp. BG6

0.00 3216 3216 S 2819997 Rhizobium sp. K1/93

0.00 3023 3023 S 2819996 Rhizobium sp. K15/93

0.00 1774 1774 S 1703968 Rhizobium sp. N871

0.00 1764 1764 S 1703970 Rhizobium sp. N541

0.00 1670 1670 S 1703971 Rhizobium sp. N941

0.00 974 974 S 1703967 Rhizobium sp. N741

0.00 943 943 S 1703962 Rhizobium sp. N1341

0.00 781 781 S 1703964 Rhizobium sp. N621

0.00 717 717 S 1703965 Rhizobium sp. N6212

0.00 397 397 S 1703966 Rhizobium sp. N731

0.00 275 275 S 1703961 Rhizobium sp. N1314

0.05 16071320 7944558 S 384 Rhizobium leguminosarum

0.02 4615042 2119099 S1 386 Rhizobium leguminosarum bv. trifolii

0.00 769858 769858 S2 395492 Rhizobium leguminosarum bv. trifolii WSM2304

0.00 758630 758630 S2 395491 Rhizobium leguminosarum bv. trifolii WSM1325

0.00 533098 533098 S2 754523 Rhizobium leguminosarum bv. trifolii WSM1689

0.00 431418 431418 S2 1033991 Rhizobium leguminosarum bv. trifolii CB782

0.00 2939 2939 S2 754521 Rhizobium leguminosarum bv. trifolii TA1

0.01 3511720 2850193 S1 387 Rhizobium leguminosarum bv. viciae

0.00 463573 463573 S2 936136 Rhizobium leguminosarum bv. viciae 248

0.00 197954 197954 S2 216596 Rhizobium leguminosarum bv. viciae 3841

0.01 3609581 965449 S 29449 Rhizobium etli

0.01 1540532 1540532 S1 538025 Rhizobium etli 8C-3

0.00 402456 0 S1 147700 Rhizobium etli bv. phaseoli

0.00 402456 402456 S2 1432049 Rhizobium etli bv. phaseoli str. IE4803

0.00 377854 0 S1 323733 Rhizobium etli bv. mimosae

0.00 377854 377854 S2 1328306 Rhizobium etli bv. mimosae str. Mim1

0.00 181075 181075 S1 347834 Rhizobium etli CFN 42

0.00 142215 142215 S1 491916 Rhizobium etli CIAT 652

0.01 2510555 1423137 S 56730 Rhizobium gallicum

0.00 1087418 0 S1 142627 Rhizobium gallicum bv. gallicum

0.00 1087418 1087418 S2 1041138 Rhizobium gallicum bv. gallicum R602sp

0.01 2411842 2128104 S 396 Rhizobium phaseoli

0.00 283738 283738 S1 526949 Rhizobium phaseoli Brasil 5

0.01 2116699 2116699 S 1312183 Rhizobium jaguaris

0.01 1941840 513400 S 398 Rhizobium tropici

0.00 1428440 1428440 S1 698761 Rhizobium tropici CIAT 899

0.01 1857299 1857299 S 50338 Rhizobium sullae

0.01 1669844 1669844 S 648995 Rhizobium pusense

0.01 1614055 1614055 S 1138189 Rhizobium bangladeshense

0.00 1474362 1474362 S 379684 Rhizobium pseudoryzae

0.00 1460900 1460900 S 451876 Rhizobium rhizoryzae

0.00 1205205 1205205 S 240521 Rhizobium daejeonense

0.00 1179606 0 S 1184720 Rhizobium anhuiense

0.00 1179606 1179606 S1 2018689 Rhizobium anhuiense bv. trifolii

0.00 1175905 1175905 S 1368430 Rhizobium rosettiformans

0.00 1157368 1157368 S 1138194 Rhizobium lentis

0.00 1135293 1135293 S 1138190 Rhizobium binae

0.00 1096374 1096374 S 2583231 Rhizobium indicum

0.00 805066 805066 S 1538158 Rhizobium acidisoli

0.00 634221 634221 S 2267833 Rhizobium oryzihabitans

0.00 423693 423693 S 2081791 Rhizobium ruizarguesonis

0.00 407790 407790 S 1076926 Rhizobium laguerreae

0.00 381772 381772 S 1538159 Rhizobium hidalgonense

0.00 65673 65673 S 348824 Rhizobium favelukesii

0.00 7378 7378 S 1120045 Rhizobium grahamii

0.00 1467 1467 S 1967781 Rhizobium esperanzae

0.12 37129681 4708629 G 357 Agrobacterium

0.05 15889793 942046 G1 1183400 Agrobacterium tumefaciens complex

0.04 12856196 12257954 S 358 Agrobacterium tumefaciens

0.00 345245 345245 S1 311403 Agrobacterium radiobacter K84

0.00 250581 250581 S1 1300225 Agrobacterium tumefaciens WRT31

0.00 2416 2416 S1 1435057 Agrobacterium tumefaciens LBA4213 (Ach5)

0.01 1672865 1671649 S 1176649 Agrobacterium fabrum

0.00 1216 1216 S1 176299 Agrobacterium fabrum str. C58

0.00 411085 227170 S 1183401 Agrobacterium fabacearum

0.00 183915 183915 S1 1300224 Agrobacterium fabacearum P4

0.00 4065 4065 S 1183410 Agrobacterium tomkonis

0.00 3536 3536 S 1183411 Agrobacterium genomosp. 6

0.02 6330700 5826515 S 373 Agrobacterium vitis

0.00 504185 504185 S1 311402 Agrobacterium vitis S4

0.01 2779162 826378 G1 2632611 unclassified Agrobacterium

0.00 633231 633231 S 2580515 Agrobacterium sp. T29

0.00 367647 367647 S 2664893 Agrobacterium sp. MA01

0.00 351151 351151 S 1842536 Agrobacterium sp. RAC06

0.00 319752 319752 S 2579248 Agrobacterium sp. CGMCC 11546

0.00 280538 280538 S 2820002 Agrobacterium sp. S7/73

0.00 465 465 S 361 Agrobacterium sp.

0.01 2142933 2142933 S 2735528 Agrobacterium vaccinii

0.01 1553938 1553938 S 160699 Agrobacterium larrymoorei

0.00 1399282 1399282 S 28099 Agrobacterium rubi

0.00 1262161 1262161 S 359 Agrobacterium rhizogenes

0.00 1047912 1047912 S 1183413 Agrobacterium salinitolerans

0.00 15171 15171 S 1183412 Agrobacterium deltaense

0.02 7432190 17704 G 1525371 Neorhizobium

0.01 4379258 2081077 S 399 Neorhizobium galegae

0.00 1164418 0 S1 323656 Neorhizobium galegae bv. officinalis

0.00 1164418 1164418 S2 1028801 Neorhizobium galegae bv. officinalis bv. officinalis str. HAMBI 1141

0.00 1133763 0 S1 323655 Neorhizobium galegae bv. orientalis

0.00 1133763 1133763 S2 1028800 Neorhizobium galegae bv. orientalis str. HAMBI 540

0.01 3035228 2146 G1 2629175 unclassified Neorhizobium

0.01 1747243 1747243 S 1825976 Neorhizobium sp. NCHU2750

0.00 1285839 1285839 S 2060726 Neorhizobium sp. SOG26

0.01 2210506 394626 G 1903858 Pseudorhizobium

0.00 920718 920718 S 1335061 Pseudorhizobium flavum

0.00 895162 895162 S 1125847 Pseudorhizobium banfieldiae

0.11 34677338 420724 F1 227292 Sinorhizobium/Ensifer group

0.06 18937888 1825449 G 28105 Sinorhizobium

0.02 5596378 4996739 S 382 Sinorhizobium meliloti

0.00 154577 154577 S1 693982 Sinorhizobium meliloti AK83

0.00 154275 154275 S1 1235461 Sinorhizobium meliloti GR4

0.00 96890 96890 S1 1401243 Sinorhizobium meliloti RU11/001

0.00 76634 76634 S1 1041157 Sinorhizobium meliloti WSM1022

0.00 53987 53987 S1 698936 Sinorhizobium meliloti BL225C

0.00 53657 53657 S1 707241 Sinorhizobium meliloti SM11

0.00 3738 3738 S1 1230587 Sinorhizobium meliloti Rm41

0.00 3443 3443 S1 266834 Sinorhizobium meliloti 1021

0.00 2438 2438 S1 1286640 Sinorhizobium meliloti 2011

0.02 5340231 0 G1 663276 Sinorhizobium fredii group

0.02 5340231 2604008 S 380 Sinorhizobium fredii

0.00 1510291 1510291 S1 1185652 Sinorhizobium fredii USDA 257

0.00 343261 343261 S1 1128334 Sinorhizobium fredii CCBAU 83666

0.00 328600 328600 S1 1128331 Sinorhizobium fredii CCBAU 45436

0.00 320709 320709 S1 1117943 Sinorhizobium fredii HH103

0.00 220265 220265 S1 1128330 Sinorhizobium fredii CCBAU 25509

0.00 13097 13097 S1 882101 Sinorhizobium fredii GR64

0.01 2136526 1762936 S 110321 Sinorhizobium medicae

0.00 214764 214764 S1 935558 Sinorhizobium medicae WSM1115

0.00 158826 158826 S1 366394 Sinorhizobium medicae WSM419

0.01 2135883 62 G1 2613772 unclassified Sinorhizobium

0.01 1631452 1631452 S 2613773 Sinorhizobium sp. BG8

0.00 370526 370526 S 794846 Sinorhizobium sp. CCBAU 05631

0.00 96469 96469 S 430451 Sinorhizobium sp. M14

0.00 37374 37374 S 1449788 Sinorhizobium sp. LM21

0.01 1903421 1375462 S 194963 Sinorhizobium americanum

0.00 527959 527959 S1 1408224 Sinorhizobium americanum CCGM7

0.05 15318726 2153821 G 106591 Ensifer

0.02 6109452 5533671 S 106592 Ensifer adhaerens

0.00 575781 575781 S1 1416753 Ensifer adhaerens OV14

0.01 2082077 379 G1 2633371 unclassified Ensifer

0.00 1418815 1418815 S 2811423 Ensifer sp. PDNC004

0.00 662883 662883 S 555315 Ensifer sp. T173

0.01 1975065 1975065 S 375549 Ensifer mexicanus

0.01 1559963 1559963 S 1752398 Ensifer alkalisoli

0.00 1438348 0 S 716925 Ensifer sojae

0.00 1438348 1438348 S1 716928 Ensifer sojae CCBAU 05684

0.03 9036394 345941 G 323620 Shinella

0.02 5520761 46029 G1 2643062 unclassified Shinella

0.01 1681407 1681407 S 879274 Shinella sp. HZN7

0.00 1355060 1355060 S 2877941 Shinella sp. XGS7

0.00 1315344 1315344 S 2715959 Shinella sp. PSBB067

0.00 1122921 1122921 S 2925841 Shinella sp. H4-D48

0.01 1958649 1958649 S 352475 Shinella zoogloeoides

0.00 1211043 1211043 S 2871820 Shinella oryzae

0.01 1837964 9917 G 34019 Liberibacter

0.00 443950 442100 S 1273132 Liberibacter crescens

0.00 1850 1850 S1 1215343 Liberibacter crescens BT-1

0.00 366419 361608 S 34021 Candidatus Liberibacter asiaticus

0.00 2144 2144 S1 1174529 Candidatus Liberibacter asiaticus str. gxpsy

0.00 1729 1729 S1 931202 Candidatus Liberibacter asiaticus str. Ishi-1

0.00 938 938 S1 537021 Candidatus Liberibacter asiaticus str. psy62

0.00 350832 0 S 556287 Candidatus Liberibacter solanacearum

0.00 350832 350832 S1 658172 Candidatus Liberibacter solanacearum CLso-ZC1

0.00 343744 336622 S 34020 Candidatus Liberibacter africanus

0.00 7122 7122 S1 1277257 Candidatus Liberibacter africanus PTSAPSY

0.00 323102 0 S 309868 Candidatus Liberibacter americanus

0.00 323102 323102 S1 1261131 Candidatus Liberibacter americanus str. Sao Paulo

0.00 1445699 0 G 1648508 Ciceribacter

0.00 1425369 1425369 S 1969821 Ciceribacter thiooxidans

0.00 20330 0 S 448181 Ciceribacter selenitireducens

0.00 20330 20330 S1 1336235 Ciceribacter selenitireducens ATCC BAA-1503

0.00 1274626 0 G 2661800 Georhizobium

0.00 1274626 1274626 S 2341112 Georhizobium profundi

0.00 1249442 0 G 2853332 Peteryoungia

0.00 1249442 1249442 S 1813451 Peteryoungia desertarenae

0.55 166915020 1351935 F 41294 Nitrobacteraceae

0.47 144216328 28175134 G 374 Bradyrhizobium

0.19 57590023 8227616 G1 2631580 unclassified Bradyrhizobium

0.01 2165944 2165944 S 2782654 Bradyrhizobium sp. 186

0.01 2157594 2157594 S 2782665 Bradyrhizobium sp. 200

0.01 2106742 2106742 S 288000 Bradyrhizobium sp. BTAi1

0.01 2078792 2078792 S 2898149 Bradyrhizobium sp. A19

0.01 2062053 2062053 S 1325100 Bradyrhizobium sp. CCBAU 51753

0.01 2013935 2013935 S 2782641 Bradyrhizobium sp. 170

0.01 1954522 1954522 S 858422 Bradyrhizobium sp. CCBAU 051011

0.01 1800770 1800770 S 115808 Bradyrhizobium sp. ORS 285

0.01 1765105 1765105 S 114615 Bradyrhizobium sp. ORS 278

0.01 1742953 1742953 S 2057741 Bradyrhizobium sp. SK17

0.01 1735029 1735029 S 1325120 Bradyrhizobium sp. CCBAU 53421

0.01 1730858 1730858 S 2976824 Bradyrhizobium sp. CB3035

0.01 1617356 1617356 S 1521768 Bradyrhizobium sp. WD16

0.01 1593488 1593488 S 2782662 Bradyrhizobium sp. 195

0.00 1525216 1525216 S 1325112 Bradyrhizobium sp. CCBAU 53340

0.00 1524853 1524853 S 1197460 Bradyrhizobium sp. 6(2017)

0.00 1413197 1413197 S 1404888 Bradyrhizobium sp. 1(2017)

0.00 1284321 1284321 S 1223566 Bradyrhizobium sp. CCGE-LA001

0.00 1283964 1283964 S 2976823 Bradyrhizobium sp. CB1024

0.00 1233805 1233805 S 1325114 Bradyrhizobium sp. CCBAU 53351

0.00 1208864 1208864 S 2976822 Bradyrhizobium sp. CB1015

0.00 1127629 1127629 S 1404443 Bradyrhizobium sp. 41S5

0.00 1021772 1021772 S 574727 Bradyrhizobium sp. C-145

0.00 1003783 1003783 S 1325102 Bradyrhizobium sp. CCBAU 51765

0.00 979568 979568 S 2590772 Bradyrhizobium sp. I71

0.00 958275 958275 S 2782659 Bradyrhizobium sp. 191

0.00 793921 793921 S 376 Bradyrhizobium sp.

0.00 667783 667783 S 1404649 Bradyrhizobium sp. 323S2

0.00 665320 665320 S 2823807 Bradyrhizobium sp. 144S4

0.00 646283 646283 S 55395 Bradyrhizobium sp. NC92

0.00 612771 612771 S 1390132 Bradyrhizobium sp. WBAH42

0.00 579812 579812 S 2782678 Bradyrhizobium sp. 4

0.00 574884 574884 S 2782674 Bradyrhizobium sp. 40

0.00 543529 543529 S 2493093 Bradyrhizobium sp. LCT2

0.00 527209 527209 S 2782660 Bradyrhizobium sp. 192

0.00 519248 519248 S 1325111 Bradyrhizobium sp. CCBAU 53338

0.00 426845 426845 S 2782655 Bradyrhizobium sp. 187

0.00 268749 268749 S 2816456 Bradyrhizobium sp. 1S5

0.00 257049 257049 S 2819348 Bradyrhizobium sp. 38S5

0.00 209600 209600 S 2782643 Bradyrhizobium sp. 172

0.00 203358 203358 S 2599805 Bradyrhizobium sp. SG09

0.00 192228 192228 S 2782686 Bradyrhizobium sp. CW1

0.00 173003 173003 S 2599819 Bradyrhizobium sp. TM102

0.00 167849 167849 S 2782609 Bradyrhizobium sp. 131

0.00 153670 153670 S 2782629 Bradyrhizobium sp. 155

0.00 59912 59912 S 1325083 Bradyrhizobium sp. CCBAU 21365

0.00 3669 3669 S 2782652 Bradyrhizobium sp. 183

0.00 3547 3547 S 2171500 Bradyrhizobium sp. WBOS08

0.00 3510 3510 S 2171498 Bradyrhizobium sp. WBOS04

0.00 3500 3500 S 2782653 Bradyrhizobium sp. 184

0.00 3294 3294 S 2171499 Bradyrhizobium sp. WBOS07

0.00 3275 3275 S 319017 Bradyrhizobium sp. WSM471

0.00 3018 3018 S 2171497 Bradyrhizobium sp. WBOS02

0.00 2623 2623 S 2578113 Bradyrhizobium sp. KBS0725

0.00 2560 2560 S 2578114 Bradyrhizobium sp. KBS0727

0.03 7830913 7823125 S 1355477 Bradyrhizobium diazoefficiens

0.00 7788 7788 S1 224911 Bradyrhizobium diazoefficiens USDA 110

0.02 7025198 7025198 S 1437360 Bradyrhizobium erythrophlei

0.02 5410610 5410610 S 858423 Bradyrhizobium arachidis

0.01 3812470 3386313 S 375 Bradyrhizobium japonicum

0.00 399045 399045 S1 476282 Bradyrhizobium japonicum SEMIA 5079

0.00 27112 27112 S1 1037409 Bradyrhizobium japonicum USDA 6

0.01 3057429 3057429 S 2840469 Bradyrhizobium sediminis

0.01 2605949 2605949 S 255045 Bradyrhizobium canariense

0.01 2318152 2318152 S 722472 Bradyrhizobium lablabi

0.01 2014563 2014563 S 1274631 Bradyrhizobium icense

0.01 1977434 0 S 44255 Bradyrhizobium oligotrophicum

0.01 1977434 1977434 S1 1245469 Bradyrhizobium oligotrophicum S58

0.01 1804927 1804927 S 2748629 Bradyrhizobium quebecense

0.01 1781183 1781183 S 190148 Bradyrhizobium paxllaeri

0.01 1761803 1761803 S 1404864 Bradyrhizobium cosmicum

0.01 1696022 1696022 S 83637 Bradyrhizobium genosp. L

0.01 1556413 1556413 S 83627 Bradyrhizobium genosp. B

0.01 1548893 1548893 S 1404411 Bradyrhizobium septentrionale

0.00 1506850 1506850 S 1549949 Bradyrhizobium vignae

0.00 1474472 1474472 S 1325090 Bradyrhizobium guangdongense

0.00 1426649 1426649 S 1325095 Bradyrhizobium guangzhouense

0.00 1302479 1302479 S 1404367 Bradyrhizobium symbiodeficiens

0.00 1244537 1244537 S 1325115 Bradyrhizobium guangxiense

0.00 1131205 1131205 S 244734 Bradyrhizobium betae

0.00 1070894 1070894 S 993502 Bradyrhizobium daqingense

0.00 1030665 1030665 S 1404768 Bradyrhizobium amphicarpaeae

0.00 927163 927163 S 1325107 Bradyrhizobium zhanjiangense

0.00 619524 1874 S 29448 Bradyrhizobium elkanii

0.00 528472 528472 S1 398525 Bradyrhizobium elkanii USDA 76

0.00 89178 89178 S1 1275962 Bradyrhizobium elkanii USDA 61

0.00 514774 514774 S 931866 Bradyrhizobium ottawaense

0.03 10177195 47237 G 1073 Rhodopseudomonas

0.02 6985399 5139537 S 1076 Rhodopseudomonas palustris

0.00 1366122 1366122 S1 316058 Rhodopseudomonas palustris HaA2

0.00 476328 476328 S1 395960 Rhodopseudomonas palustris TIE-1

0.00 3412 3412 S1 258594 Rhodopseudomonas palustris CGA009

0.01 1610329 0 G1 2638247 unclassified Rhodopseudomonas

0.01 1610329 1610329 S 340268 Rhodopseudomonas sp. SK50-23

0.01 1534230 1534230 S 475937 Rhodopseudomonas boonkerdii

0.02 4686282 788525 G 1395974 Tardiphaga

0.01 2828485 1505416 G1 2631404 unclassified Tardiphaga

0.00 929976 929976 S 1404741 Tardiphaga sp. 37S4

0.00 132364 132364 S 2592814 Tardiphaga sp. vice154

0.00 104590 104590 S 2592817 Tardiphaga sp. vice304

0.00 83663 83663 S 2592815 Tardiphaga sp. vice278

0.00 72476 72476 S 2592816 Tardiphaga sp. vice352

0.00 1069272 1069272 S 943830 Tardiphaga robiniae

0.01 2800540 199 G 1033 Afipia

0.01 1683749 0 G1 2642050 unclassified Afipia

0.01 1683749 1683749 S 1882747 Afipia sp. GAS231

0.00 1116592 1090923 S 40137 Afipia carboxidovorans

0.00 23137 23137 S1 504832 Afipia carboxidovorans OM5

0.00 2532 2532 S1 1031710 Afipia carboxidovorans OM4

0.01 2309721 18045 G 911 Nitrobacter

0.00 1369814 0 S 912 Nitrobacter hamburgensis

0.00 1369814 1369814 S1 323097 Nitrobacter hamburgensis X14

0.00 921862 0 S 913 Nitrobacter winogradskyi

0.00 921862 921862 S1 323098 Nitrobacter winogradskyi Nb-255

0.00 1373019 0 G 1649510 Variibacter

0.00 1373019 1373019 S 1333996 Variibacter gotjawalensis

0.36 110082294 363441 F 69277 Phyllobacteriaceae

0.26 80769599 10113463 G 68287 Mesorhizobium

0.18 55256495 6238194 G1 325217 unclassified Mesorhizobium

0.01 1877342 1877342 S 2082387 Mesorhizobium sp. Pch-S

0.01 1820286 1820286 S 2654248 Mesorhizobium sp. INR15

0.01 1718832 1718832 S 2865838 Mesorhizobium sp. AR07

0.01 1560506 1560506 S 2880934 Mesorhizobium sp. PAMC28654

0.01 1551830 1551830 S 2493673 Mesorhizobium sp. M1B.F.Ca.ET.045.04.1.1

0.01 1533726 1533726 S 2865839 Mesorhizobium sp. AR10

0.00 1395793 1395793 S 2589974 Mesorhizobium sp. B2-1-1

0.00 1394383 1394383 S 2865837 Mesorhizobium sp. AR02

0.00 1385780 1385780 S 2589976 Mesorhizobium sp. B1-1-8

0.00 1385601 1385601 S 2777475 Mesorhizobium sp. J8

0.00 1375448 1375448 S 2493672 Mesorhizobium sp. M1E.F.Ca.ET.045.02.1.1

0.00 1361795 1361795 S 2493676 Mesorhizobium sp. M3A.F.Ca.ET.080.04.2.1

0.00 1335412 1335412 S 2589888 Mesorhizobium sp. B4-1-4

0.00 1327396 1327396 S 2589967 Mesorhizobium sp. B2-1-8

0.00 1325290 1325290 S 2108445 Mesorhizobium sp. DCY119

0.00 1325129 1325129 S 2589903 Mesorhizobium sp. B2-8-5

0.00 1321659 1321659 S 2483404 Mesorhizobium sp. NZP2077

0.00 1308596 1308596 S 2744521 Mesorhizobium sp. L-2-11

0.00 1297400 1297400 S 2744518 Mesorhizobium sp. 131-2-1

0.00 1279682 1279682 S 2493669 Mesorhizobium sp. M1D.F.Ca.ET.043.01.1.1

0.00 1278338 1278338 S 2584466 Mesorhizobium sp. 8

0.00 1276051 1276051 S 2493680 Mesorhizobium sp. M1A.F.Ca.IN.022.06.1.1

0.00 1255252 1255252 S 2493678 Mesorhizobium sp. M7D.F.Ca.US.005.01.1.1

0.00 1229840 1229840 S 2493675 Mesorhizobium sp. M4B.F.Ca.ET.058.02.1.1

0.00 1226789 1226789 S 2493677 Mesorhizobium sp. M6A.T.Cr.TU.016.01.1.1

0.00 1168986 1168986 S 2654249 Mesorhizobium sp. NBSH29

0.00 1147760 1147760 S 2744516 Mesorhizobium sp. 113-3-3

0.00 1143811 1143811 S 2493679 Mesorhizobium sp. M8A.F.Ca.ET.057.01.1.1

0.00 1139713 1139713 S 2493668 Mesorhizobium sp. M9A.F.Ca.ET.002.03.1.2

0.00 1109711 1109711 S 2744515 Mesorhizobium sp. 113-1-2

0.00 1058180 1058180 S 2493681 Mesorhizobium sp. M7A.F.Ce.TU.012.03.2.1

0.00 983997 983997 S 2483403 Mesorhizobium sp. NZP2298

0.00 933753 933753 S 2744517 Mesorhizobium sp. 113-3-9

0.00 918362 918362 S 2493671 Mesorhizobium sp. M2A.F.Ca.ET.043.05.1.1

0.00 790118 790118 S 1854057 Mesorhizobium sp. AA22

0.00 655477 655477 S 2744523 Mesorhizobium sp. L-8-10

0.00 636786 636786 S 2744522 Mesorhizobium sp. L-8-3

0.00 580624 580624 S 2493674 Mesorhizobium sp. M2A.F.Ca.ET.046.03.2.1

0.00 502916 502916 S 2493670 Mesorhizobium sp. M2A.F.Ca.ET.043.02.1.1

0.00 370891 370891 S 278153 Mesorhizobium sp. WSM1497

0.00 296108 296108 S 2744520 Mesorhizobium sp. 131-3-5

0.00 225712 225712 S 2744519 Mesorhizobium sp. 131-2-5

0.00 207240 207240 S 2483402 Mesorhizobium sp. NZP2234

0.01 2162561 1304204 S 593909 Mesorhizobium opportunistum

0.00 858357 858357 S1 536019 Mesorhizobium opportunistum WSM2075

0.01 1906346 768307 S 39645 Mesorhizobium ciceri

0.00 689775 689775 S1 682633 Mesorhizobium ciceri ca181

0.00 448264 308566 S1 278148 Mesorhizobium ciceri biovar biserrulae

0.00 139698 139698 S2 765698 Mesorhizobium ciceri biovar biserrulae WSM1271

0.01 1715423 211409 S 381 Mesorhizobium loti

0.00 1259552 1259552 S1 935548 Mesorhizobium loti R88b

0.00 244462 244462 S1 935546 Mesorhizobium loti NZP2037

0.01 1633939 0 S 71433 Mesorhizobium amorphae

0.01 1633939 1633939 S1 1082933 Mesorhizobium amorphae CCNWGS0123

0.01 1625923 1625923 S 2725666 Mesorhizobium terrae

0.00 1458240 1187996 S 28104 Mesorhizobium huakuii

0.00 270244 270244 S1 763057 Mesorhizobium huakuii 7653R

0.00 1322401 1322401 S 2775404 Mesorhizobium onobrychidis

0.00 1311244 787179 S 2066070 Mesorhizobium japonicum

0.00 521825 521825 S1 266835 Mesorhizobium japonicum MAFF 303099

0.00 2240 2240 S1 935547 Mesorhizobium japonicum R7A

0.00 1166680 1166680 S 1777866 Mesorhizobium erdmanii

0.00 1094032 0 S 536018 Mesorhizobium australicum

0.00 1094032 1094032 S1 754035 Mesorhizobium australicum WSM2073

0.00 2852 2852 S 1777867 Mesorhizobium jarvisii

0.03 9653637 118437 G 28100 Phyllobacterium

0.02 5901870 37314 G1 2638441 unclassified Phyllobacterium

0.01 1657400 1657400 S 2978392 Phyllobacterium sp. A18/5-2

0.01 1558837 1558837 S 2718938 Phyllobacterium sp. 628

0.00 1513856 1513856 S 555317 Phyllobacterium sp. T1293

0.00 1134463 1134463 S 555316 Phyllobacterium sp. T1018

0.01 3633330 3633330 S 1867719 Phyllobacterium zundukense

0.02 4763264 1464421 G 31988 Aminobacter

0.01 2835253 125636 G1 2644704 unclassified Aminobacter

0.01 1651160 1651160 S 374606 Aminobacter sp. MSH1

0.00 682261 682261 S 2774562 Aminobacter sp. SR38

0.00 376196 376196 S 2666139 Aminobacter sp. MDW-2

0.00 463590 463590 S 83263 Aminobacter aminovorans

0.01 4000694 28084 G 245876 Nitratireductor

0.01 2776056 686 G1 2641084 unclassified Nitratireductor

0.00 1394203 1394203 S 1756988 Nitratireductor sp. OM-1

0.00 1381167 1381167 S 2599600 Nitratireductor sp. SY7

0.00 1196554 1196554 S 430679 Nitratireductor kimnyeongensis

0.01 3205463 9382 G 2911176 Aquibium

0.01 1686242 1686242 S 675281 Aquibium microcysteis

0.00 1509839 1509839 S 1670800 Aquibium oceanicum

0.01 2859830 6386 G 274591 Hoeflea

0.00 1517989 0 G1 2614931 unclassified Hoeflea

0.00 1517989 1517989 S 1620421 Hoeflea sp. IMCC20628

0.00 1335455 0 S 244596 Hoeflea phototrophica

0.00 1335455 1335455 S1 411684 Hoeflea phototrophica DFL-43

0.00 1250147 0 G 1649463 Lentilitoribacter

0.00 1250147 0 G1 2647570 unclassified Lentilitoribacter

0.00 1250147 1250147 S 2305987 Lentilitoribacter sp. Alg239-R112

0.00 1172522 0 G 1594166 Oricola

0.00 1172522 1172522 S 2742145 Oricola thermophila

0.00 1032905 0 G 2712688 Salaquimonas

0.00 1032905 1032905 S 2712698 Salaquimonas pukyongi

0.00 1010792 0 G 1915401 Roseitalea

0.00 1010792 1010792 S 1852022 Roseitalea porphyridii

0.16 49650440 453719 F 119045 Methylobacteriaceae

0.10 29717076 4661489 G 407 Methylobacterium

0.01 3370518 3324 G1 2615210 unclassified Methylobacterium

0.01 1541951 1541951 S 2603276 Methylobacterium sp. WL1

0.00 1277330 1277330 S 2202826 Methylobacterium sp. 17Sr1-1

0.00 287783 287783 S 1479019 Methylobacterium sp. C1

0.00 209772 209772 S 739141 Methylobacterium sp. XJLW

0.00 50358 50358 S 409 Methylobacterium sp.

0.01 2925356 2925356 S 270351 Methylobacterium aquaticum

0.01 2359553 0 S 114616 Methylobacterium nodulans

0.01 2359553 2359553 S1 460265 Methylobacterium nodulans ORS 2060

0.01 2059276 2059276 S 2051553 Methylobacterium currus

0.01 1800235 1800235 S 2202825 Methylobacterium durans

0.01 1595283 0 S 39956 Methylobacterium mesophilicum

0.01 1595283 1595283 S1 908290 Methylobacterium mesophilicum SR1.6/6

0.01 1592452 1592452 S 374432 Methylobacterium tardum

0.00 1470331 1470331 S 269660 Methylobacterium brachiatum

0.00 1432621 1432621 S 2202828 Methylobacterium radiodurans

0.00 1366295 1366295 S 1775910 Methylobacterium indicum

0.00 1352379 1174942 S 334852 Methylobacterium oryzae

0.00 177437 177437 S1 693986 Methylobacterium oryzae CBMB20

0.00 1316329 1316329 S 410 Methylobacterium organophilum

0.00 1213938 1213938 S 2202827 Methylobacterium terrae

0.00 819922 511748 S 31998 Methylobacterium radiotolerans

0.00 308174 308174 S1 426355 Methylobacterium radiotolerans JCM 2831

0.00 381099 381099 S 418223 Methylobacterium phyllosphaerae

0.03 9847990 2232498 G 2282523 Methylorubrum

0.01 3107969 2504913 S 223967 Methylorubrum populi

0.00 603056 603056 S1 441620 Methylorubrum populi BJ001

0.01 2987654 1003842 S 408 Methylorubrum extorquens

0.00 570335 570335 S1 440085 Methylorubrum extorquens CM4

0.00 502477 502477 S1 661410 Methylorubrum extorquens DM4

0.00 501932 501932 S1 272630 Methylorubrum extorquens AM1

0.00 409068 409068 S1 419610 Methylorubrum extorquens PA1

0.00 1348847 648 G1 2648467 unclassified Methylorubrum

0.00 1195365 1195365 S 2897334 Methylorubrum sp. B1-46

0.00 152834 152834 S 2938232 Methylorubrum sp. GM97

0.00 171022 171022 S 29429 Methylorubrum zatmanii

0.03 9631655 150506 G 186650 Microvirga

0.02 5846659 44324 G1 2617746 unclassified Microvirga

0.01 2807979 2807979 S 2807101 Microvirga sp. VF16

0.01 1730784 1730784 S 2740529 Microvirga sp. R24

0.00 1263572 1263572 S 2082949 Microvirga sp. 17 mud 1-3

0.01 2580350 2580350 S 1882682 Microvirga ossetica

0.00 1054140 1054140 S 2651334 Microvirga thermotolerans

0.07 21107718 65369 F 2821832 Stappiaceae

0.02 6585800 12777 G 150830 Roseibium

0.02 4892659 4892659 S 187304 Roseibium aggregatum

0.01 1680364 0 S 388408 Roseibium alexandrii

0.01 1680364 1680364 S1 244592 Roseibium alexandrii DFL-11

0.02 5694497 0 G 478070 Labrenzia

0.02 5694497 9454 G1 2648686 unclassified Labrenzia

0.01 1969795 1969795 S 2587861 Labrenzia sp. THAF82

0.01 1921274 1921274 S 2590016 Labrenzia sp. PHM005

0.01 1793974 1793974 S 2021862 Labrenzia sp. VG12

0.01 3236204 532112 G 258255 Pseudovibrio

0.00 1364391 1364391 S 1898042 Pseudovibrio brasiliensis

0.00 1339701 0 G1 2627060 unclassified Pseudovibrio

0.00 1339701 1339701 S 911045 Pseudovibrio sp. FO-BEG1

0.01 2819819 6581 G 152161 Stappia

0.00 1447358 1447358 S 538381 Stappia indica

0.00 1365880 0 G1 2629676 unclassified Stappia

0.00 1365880 1365880 S 1881061 Stappia sp. ES.058

0.01 2706029 0 G 227873 Pannonibacter

0.01 2706029 2196250 S 121719 Pannonibacter phragmitetus

0.00 509779 509779 S1 1402210 Pannonibacter phragmitetus BB

0.06 19838033 86077 F 2831106 Devosiaceae

0.05 14799554 248362 G 46913 Devosia

0.02 7522827 25884 G1 196773 unclassified Devosia

0.00 1483010 1483010 S 1736675 Devosia sp. A16

0.00 1251938 1251938 S 2917991 Devosia sp. JXJ CY 41

0.00 1247096 1247096 S 2499144 Devosia sp. 1566

0.00 1218757 1218757 S 2806348 Devosia sp. SL43

0.00 1156912 1156912 S 2759954 Devosia sp. MC521

0.00 1139230 1139230 S 2083786 Devosia sp. I507

0.00 1374519 1374519 S 191302 Devosia neptuniae

0.00 1263406 1263406 S 400770 Devosia ginsengisoli

0.00 1218678 1218678 S 2860336 Devosia salina

0.00 1145719 1145719 S 2657486 Devosia beringensis

0.00 1039667 1039667 S 2774137 Devosia rhizoryzae

0.00 986376 986376 S 2801335 Devosia oryziradicis

0.00 1302092 0 G 1573407 Paradevosia

0.00 1302092 1302092 S 1335043 Paradevosia shaoguanensis

0.00 1271672 0 G 1827478 Youhaiella

0.00 1271672 1271672 S 1447062 Youhaiella tibetensis

0.00 1200614 0 G 1082930 Pelagibacterium

0.00 1200614 1197271 S 531813 Pelagibacterium halotolerans

0.00 3343 3343 S1 1082931 Pelagibacterium halotolerans B2

0.00 1178024 0 G 623276 Maritalea

0.00 1178024 1178024 S 454601 Maritalea myrionectae

0.06 17726079 147862 F 335928 Xanthobacteraceae

0.01 4076409 780793 G 279 Xanthobacter

0.01 1795482 1795482 S 2528964 Xanthobacter dioxanivorans

0.00 782957 0 G1 2623496 unclassified Xanthobacter

0.00 782957 782957 S 2419844 Xanthobacter sp. YC-JY1

0.00 716523 716523 S 280 Xanthobacter autotrophicus

0.00 654 654 S 281 Xanthobacter flavus

0.01 3742884 40534 G 99 Ancylobacter

0.00 1352998 1352998 S 223390 Ancylobacter polymorphus

0.00 1348645 1348645 S 1745854 Ancylobacter pratisalsi

0.00 1000707 0 G1 2626613 unclassified Ancylobacter

0.00 1000707 1000707 S 1850374 Ancylobacter sp. TS-1

0.01 3036221 9823 G 152053 Starkeya

0.01 1779683 0 G1 2615126 unclassified Starkeya

0.01 1779683 1779683 S 2709380 Starkeya sp. ORNL1

0.00 1246715 495 S 921 Starkeya novella

0.00 1246220 1246220 S1 639283 Starkeya novella DSM 506

0.01 2943715 5915 G 556257 Pseudolabrys

0.01 1630660 1630660 S 331696 Pseudolabrys taiwanensis

0.00 1307140 0 G1 2638514 unclassified Pseudolabrys

0.00 1307140 1307140 S 2562284 Pseudolabrys sp. FHR47

0.01 2245802 0 G 204476 Labrys

0.01 2245802 0 G1 2688601 unclassified Labrys (in: Bacteria)

0.01 2245802 2245802 S 2789216 Labrys sp. KNU-23

0.01 1533186 0 G 6 Azorhizobium

0.01 1533186 0 S 7 Azorhizobium caulinodans

0.01 1533186 1533186 S1 438753 Azorhizobium caulinodans ORS 571

0.06 17279193 12310 F 118882 Brucellaceae

0.05 14931004 3031 F1 2826938 Brucella/Ochrobactrum group

0.05 14877004 3030208 G 234 Brucella

0.01 2787328 609529 G1 2632610 unclassified Brucella

0.00 1064887 1064887 S 2840456 Brucella sp. BTU1

0.00 163173 163173 S 1844051 Brucella sp. 09RB8910

0.00 161350 161350 S 2975052 Brucella sp. 2716

0.00 150883 150883 S 1149953 Brucella sp. 10RB9215

0.00 135266 135266 S 2769351 Brucella sp. 6810

0.00 128419 128419 S 2592625 Brucella sp. 2280

0.00 107989 107989 S 2691913 Brucella sp. BO3

0.00 101283 101283 S 1149952 Brucella sp. 09RB8471

0.00 84107 84107 S 693750 Brucella sp. BO2

0.00 77946 77946 S 2821140 Brucella sp. 458

0.00 1257 1257 S 2975050 Brucella sp. 1315

0.00 876 876 S 2975051 Brucella sp. 2594

0.00 363 363 S 1891098 Brucella sp. 2002734562

0.01 2661898 2659336 S 529 Brucella anthropi

0.00 2562 2562 S1 439375 Brucella anthropi ATCC 49188

0.01 1607618 1607618 S 271865 [Ochrobactrum] quorumnocens

0.01 1600752 1600752 S 94625 Brucella intermedia

0.00 1068403 1068403 S 419475 Brucella pseudogrignonensis

0.00 991283 991283 S 370111 Brucella pseudintermedia

0.00 631983 631983 S 571256 Brucella pituitosa

0.00 150511 141409 S 29459 Brucella melitensis

0.00 2980 0 S1 644337 Brucella melitensis bv. 1

0.00 2980 2980 S2 224914 Brucella melitensis bv. 1 str. 16M

0.00 1996 0 S1 644340 Brucella melitensis bv. 3

0.00 1996 1996 S2 520466 Brucella melitensis bv. 3 str. Ether

0.00 1887 1887 S1 1029825 Brucella melitensis NI

0.00 1221 309 S1 941967 Brucella melitensis M28

0.00 912 912 S2 703352 Brucella melitensis M5-90

0.00 1018 322 S1 645169 Brucella melitensis bv. 2

0.00 687 687 S2 520465 Brucella melitensis bv. 2 str. 63/9

0.00 9 9 S2 546272 Brucella melitensis ATCC 23457

0.00 106464 106464 S 1218315 Brucella inopinata

0.00 73543 29324 S 29461 Brucella suis

0.00 32197 31898 S1 645170 Brucella suis bv. 2

0.00 299 299 S2 470137 Brucella suis ATCC 23445

0.00 6212 5666 S1 1567496 Brucella suis bv. 3

0.00 546 546 S2 520487 Brucella suis bv. 3 str. 686

0.00 1873 1480 S1 644346 Brucella suis bv. 1

0.00 393 393 S2 1004954 Brucella suis bv. 1 str. S2

0.00 1153 1153 S1 1567502 Brucella suis bv. 5

0.00 854 854 S1 204722 Brucella suis 1330

0.00 834 834 S1 1567501 Brucella suis bv. 4

0.00 567 567 S1 1171378 Brucella suis 019

0.00 529 529 S1 1112912 Brucella suis VBI22

0.00 64739 58256 S 235 Brucella abortus

0.00 2336 2336 S1 1210454 Brucella abortus 104M

0.00 774 0 S1 644332 Brucella abortus bv. 2

0.00 774 774 S2 520450 Brucella abortus bv. 2 str. 86/8/59

0.00 741 741 S1 1104320 Brucella abortus A13334

0.00 541 0 S1 1567505 Brucella abortus bv. 9

0.00 541 541 S2 520455 Brucella abortus bv. 9 str. C68

0.00 508 508 S1 359391 Brucella abortus 2308

0.00 450 450 S1 430066 Brucella abortus S19

0.00 413 0 S1 1567504 Brucella abortus bv. 6

0.00 413 413 S2 520454 Brucella abortus bv. 6 str. 870

0.00 371 0 S1 35802 Brucella abortus bv. 1

0.00 371 371 S2 262698 Brucella abortus bv. 1 str. 9-941

0.00 349 349 S1 1198700 Brucella abortus RB51-AHVLA

0.00 36891 0 S 236 Brucella ovis

0.00 36891 36891 S1 444178 Brucella ovis ATCC 25840

0.00 27025 16405 S 120577 Brucella ceti

0.00 9259 9259 S1 1407053 Brucella ceti TE28753-12

0.00 1361 1361 S1 1423891 Brucella ceti TE10759-12

0.00 14153 10188 S 120576 Brucella pinnipedialis

0.00 3965 3965 S1 520461 Brucella pinnipedialis B2/94

0.00 13596 0 S 444163 Brucella microti

0.00 13596 13596 S1 568815 Brucella microti CCM 4915

0.00 10609 8427 S 36855 Brucella canis

0.00 982 982 S1 1408887 Brucella canis str. Oliveri

0.00 830 830 S1 1104321 Brucella canis HSK A52141

0.00 370 370 S1 483179 Brucella canis ATCC 23365

0.00 50969 0 G 528 Ochrobactrum

0.00 50969 0 G1 239106 unclassified Ochrobactrum

0.00 50969 50969 S 1449781 Ochrobactrum sp. LM19

0.01 2335879 673013 G 354349 Pseudochrobactrum

0.00 1112327 134456 G1 2647013 unclassified Pseudochrobactrum

0.00 522625 522625 S 2879116 Pseudochrobactrum sp. XF203

0.00 455246 455246 S 2864102 Pseudochrobactrum sp. Wa41.01b-1

0.00 550539 550539 S 2834768 Pseudochrobactrum algeriensis

0.05 16444226 5072 F 45401 Hyphomicrobiaceae

0.02 4829274 9325 G 81 Hyphomicrobium

0.01 2242788 19847 S 53399 Hyphomicrobium denitrificans

0.00 1151512 1151512 S1 670307 Hyphomicrobium denitrificans 1NES1

0.00 1071429 1071429 S1 582899 Hyphomicrobium denitrificans ATCC 51888

0.00 1464247 0 G1 2619925 unclassified Hyphomicrobium

0.00 1464247 1464247 S 717785 Hyphomicrobium sp. MC1

0.00 1112914 0 S 1427356 Hyphomicrobium nitrativorans

0.00 1112914 1112914 S1 1029756 Hyphomicrobium nitrativorans NL23

0.01 2470570 0 G 29407 Rhodoplanes

0.01 2454179 0 G1 2619116 unclassified Rhodoplanes

0.01 2454179 2454179 S 674703 Rhodoplanes sp. Z2-YC6860

0.00 16391 16391 S 444923 Rhodoplanes piscinae

0.01 2240156 7404 G 2827482 Caenibius

0.00 1226752 0 S 169176 Caenibius tardaugens

0.00 1226752 1226752 S1 1219035 Caenibius tardaugens NBRC 16725

0.00 1006000 0 G1 2827483 unclassified Caenibius

0.00 1006000 1006000 S 2872646 Caenibius sp. WL

0.01 1916922 120552 G 1484898 Methyloceanibacter

0.00 932965 932965 S 1384459 Methyloceanibacter caenitepidi

0.00 863405 0 G1 2617503 unclassified Methyloceanibacter

0.00 863405 863405 S 2170729 Methyloceanibacter sp. wino2

0.01 1563662 0 G 45402 Aquabacter

0.01 1563662 0 G1 2663263 unclassified Aquabacter

0.01 1563662 1563662 S 2820278 Aquabacter sp. L1I39

0.00 1257798 0 G 119044 Filomicrobium

0.00 1257798 1257798 S 1608628 Candidatus Filomicrobium marinum

0.00 1192849 0 G 1068 Rhodomicrobium

0.00 1192849 0 S 1069 Rhodomicrobium vannielii

0.00 1192849 1192849 S1 648757 Rhodomicrobium vannielii ATCC 17100

0.00 967923 0 G 1485594 Methyloligella

0.00 967923 0 G1 2625955 unclassified Methyloligella

0.00 967923 967923 S 2742204 Methyloligella sp. GL2

0.05 15624968 0 F 772 Bartonellaceae

0.05 15624968 1354416 G 773 Bartonella

0.01 3763655 2396721 G1 2645622 unclassified Bartonella

0.00 377753 377753 S 2759660 Bartonella sp. HY038

0.00 372763 372763 S 2979331 Bartonella sp. HY406

0.00 354872 354872 S 1933904 Bartonella sp. WD16.2

0.00 220849 220849 S 515256 Bartonella sp. 1-1C

0.00 10692 10692 S 1933910 Bartonella sp. A1379B

0.00 6973 6973 S 1933905 Bartonella sp. CDC_skunk

0.00 6495 6495 S 1933912 Bartonella sp. Raccoon60

0.00 5631 5631 S 596995 Bartonella sp. TT0105

0.00 2086 2086 S 1933909 Bartonella sp. 114

0.00 1744 1744 S 2979321 Bartonella sp. HY329

0.00 1695 1695 S 2979320 Bartonella sp. HY328

0.00 1641 1641 S 1933908 Bartonella sp. 11B

0.00 1360 1360 S 1933906 Bartonella sp. JB15

0.00 1198 1198 S 1933911 Bartonella sp. Coyote22sub2

0.00 1182 1182 S 1933907 Bartonella sp. JB63

0.00 1034782 1034782 S 2750929 Bartonella apihabitans

0.00 807317 807317 S 2750995 Bartonella choladocola

0.00 796804 462482 S 33047 Bartonella vinsonii

0.00 334322 0 S1 40933 Bartonella vinsonii subsp. berkhoffii

0.00 334322 334322 S2 1094497 Bartonella vinsonii subsp. berkhoffii str. Winnie

0.00 643766 641970 S 38323 Bartonella henselae

0.00 1796 1796 S1 283166 Bartonella henselae str. Houston-1

0.00 628182 628182 S 2267275 Bartonella krasnovii

0.00 574672 546302 S 803 Bartonella quintana

0.00 27654 27654 S1 1225179 Bartonella quintana RM-11

0.00 716 716 S1 283165 Bartonella quintana str. Toulouse

0.00 505461 505461 S 33046 Bartonella taylorii

0.00 493497 741 S 33045 Bartonella grahamii

0.00 492756 492756 S1 634504 Bartonella grahamii as4aup

0.00 486773 486773 S 2893471 Bartonella machadoae

0.00 463633 0 S 388640 Bartonella australis

0.00 463633 463633 S1 1094489 Bartonella australis AUST/NH1

0.00 438043 438043 S 2961895 Bartonella harrusi

0.00 423389 423389 S 1318743 Bartonella ancashensis

0.00 414100 408898 S 85701 Bartonella tribocorum

0.00 5202 5202 S1 382640 Bartonella tribocorum CIP 105476

0.00 412384 412384 S 52764 Bartonella alsatica

0.00 407355 0 S 111504 Bartonella birtlesii

0.00 407355 407355 S1 1095900 Bartonella birtlesii IBS 325

0.00 400399 399719 S 774 Bartonella bacilliformis

0.00 680 680 S1 360095 Bartonella bacilliformis KC583

0.00 357072 0 S 155194 Bartonella bovis

0.00 357072 357072 S1 1094491 Bartonella bovis 91-4

0.00 352003 0 S 165694 Bartonella schoenbuchensis

0.00 352003 352003 S1 687861 Bartonella schoenbuchensis R1

0.00 325496 0 S 56426 Bartonella clarridgeiae

0.00 325496 325496 S1 696125 Bartonella clarridgeiae 73

0.00 290717 290717 S 807 Bartonella elizabethae

0.00 251052 251052 S 2133959 Bartonella kosoyi

0.05 15566841 13518 F 255475 Aurantimonadaceae

0.02 7395113 172286 G 414371 Aureimonas

0.01 3960671 4961 G1 2615206 unclassified Aureimonas

0.00 1495916 1495916 S 1349819 Aureimonas sp. AU20

0.00 1420624 1420624 S 2826993 Aureimonas sp. SA4125

0.00 1035310 1035310 S 2816454 Aureimonas sp. OT7

0.00 2994 2994 S 1638161 Aureimonas sp. AU12

0.00 384 384 S 1637747 Aureimonas sp. AU40

0.00 251 251 S 1638165 Aureimonas sp. N4

0.00 231 231 S 1638164 Aureimonas sp. D3

0.00 1198463 1198463 S 370622 Aureimonas altamirensis

0.00 1067709 1067709 S 1701758 Aureimonas populi

0.00 994590 994590 S 2758041 Aureimonas mangrovi

0.00 1394 0 S 401562 Aureimonas ureilytica

0.00 1394 1394 S1 1121028 Aureimonas ureilytica DSM 18598 = NBRC 106430

0.02 6925609 662889 G 293088 Martelella

0.01 2578665 1647529 G1 2629616 unclassified Martelella

0.00 921451 921451 S 686597 Martelella sp. AD-3

0.00 5008 5008 S 2740297 Martelella sp. NC18

0.00 4677 4677 S 2740298 Martelella sp. NC20

0.00 1461480 0 S 293089 Martelella mediterranea

0.00 1461480 1461480 S1 1122214 Martelella mediterranea DSM 17316

0.00 1347845 1347845 S 1486262 Martelella endophytica

0.00 874730 874730 S 2583532 Martelella lutilitoris

0.00 1232601 0 G 182269 Aurantimonas

0.00 1232601 0 G1 2638230 unclassified Aurantimonas

0.00 1232601 1232601 S 2906072 Aurantimonas sp. HBX-1

0.04 12029636 0 F 2831100 Boseaceae

0.04 12029636 96837 G 85413 Bosea

0.03 10083856 214589 G1 2653178 unclassified Bosea

0.01 1895841 1895841 S 2599640 Bosea sp. F3-2

0.01 1807134 1807134 S 1867715 Bosea sp. Tri-49

0.01 1592312 1592312 S 2015316 Bosea sp. AS-1

0.01 1537037 1537037 S 1792307 Bosea sp. PAMC 26642

0.01 1533598 1533598 S 1842539 Bosea sp. RAC05

0.00 1503345 1503345 S 2020412 Bosea sp. ANAM02

0.01 1848943 1848943 S 1526658 Bosea vaviloviae

0.04 11730731 21496 F 31993 Methylocystaceae

0.02 7386687 685970 G 133 Methylocystis

0.00 1407731 1407731 S 391905 Methylocystis heyeri

0.00 1396381 1396381 S 655015 Methylocystis bryophila

0.00 1325129 20076 G1 2625913 unclassified Methylocystis

0.00 867789 867789 S 187303 Methylocystis sp. SC2

0.00 437264 437264 S 743836 Methylocystis sp. SB2

0.00 1311827 1311827 S 134 Methylocystis parvus

0.00 1259649 1259649 S 173366 Methylocystis rosea

0.01 2754430 28227 G 425 Methylosinus

0.00 1409625 0 S 426 Methylosinus trichosporium

0.00 1409625 1409625 S1 595536 Methylosinus trichosporium OB3b

0.00 1316578 0 G1 2624500 unclassified Methylosinus

0.00 1316578 1316578 S 2699395 Methylosinus sp. C49

0.01 1568118 0 G 2041902 Chenggangzhangella

0.01 1568118 1568118 S 1437009 Chenggangzhangella methanolivorans

0.02 6055234 4761 F 45404 Beijerinckiaceae

0.01 2594097 6052 G 120652 Methylocella

0.00 1313910 1313910 S 227605 Methylocella tundrae

0.00 1274135 0 S 199596 Methylocella silvestris

0.00 1274135 1274135 S1 395965 Methylocella silvestris BL2

0.01 2089865 1126 G 1156568 Methylovirgula

0.00 1106884 0 G1 2640612 unclassified Methylovirgula

0.00 1106884 1106884 S 2822761 Methylovirgula sp. HY1

0.00 981855 981855 S 569860 Methylovirgula ligni

0.00 1366511 0 G 532 Beijerinckia

0.00 1366511 0 S 533 Beijerinckia indica

0.00 1366511 0 S1 31994 Beijerinckia indica subsp. indica

0.00 1366511 1366511 S2 395963 Beijerinckia indica subsp. indica ATCC 9039

0.02 5229229 289 O1 119042 Hyphomicrobiales incertae sedis

0.01 2172022 0 G 169055 Nordella

0.01 2172022 0 G1 2623691 unclassified Nordella

0.01 2172022 2172022 S 2712222 Nordella sp. HKS 07

0.01 1806857 0 G 1734920 Pseudorhodoplanes

0.01 1806857 1806857 S 1235591 Pseudorhodoplanes sinuspersici

0.00 925989 0 G 2874214 Terrihabitans

0.00 925989 925989 S 708113 Terrihabitans soli

0.00 324072 0 G 573657 Candidatus Hodgkinia

0.00 324072 324072 S 573658 Candidatus Hodgkinia cicadicola

0.02 4818427 0 F 2843305 Phreatobacteraceae

0.02 4818427 29548 G 1632780 Phreatobacter

0.01 2095753 2095753 S 1940610 Phreatobacter stygius

0.00 1446477 0 G1 2644610 unclassified Phreatobacter

0.00 1446477 1446477 S 2570229 Phreatobacter sp. NMCR1094

0.00 1246649 1246649 S 1868589 Phreatobacter cathodiphilus

0.01 4576054 5312 F 2813035 Parvibaculaceae

0.00 1359810 0 G 2838250 Kaustia

0.00 1359810 1359810 S 2593653 Kaustia mangrovi

0.00 1129216 0 G 256616 Parvibaculum

0.00 1129216 0 S 256618 Parvibaculum lavamentivorans

0.00 1129216 1129216 S1 402881 Parvibaculum lavamentivorans DS-1

0.00 1075505 0 G 1541445 Candidatus Phaeomarinobacter

0.00 1075505 1075505 S 1458461 Candidatus Phaeomarinobacter ectocarpi

0.00 1006211 0 G 1742974 Pyruvatibacter

0.00 1006211 1006211 S 1712261 Pyruvatibacter mobilis

0.01 2293554 0 F 2036754 Chelatococcaceae

0.01 2293554 312490 G 28209 Chelatococcus

0.00 1139334 0 G1 2638111 unclassified Chelatococcus

0.00 1139334 1139334 S 1702325 Chelatococcus sp. CO-6

0.00 841730 841730 S 444444 Chelatococcus daeguensis

0.01 2215467 0 F 2723775 Lichenihabitantaceae

0.01 2215467 538729 G 2723776 Lichenihabitans

0.00 924028 924028 S 2528642 Lichenihabitans psoromatis

0.00 752710 0 G1 2880931 unclassified Lichenihabitans

0.00 752710 752710 S 2880932 Lichenihabitans sp. PAMC28606

0.01 2096979 0 F 2831090 Blastochloridaceae

0.01 2096979 23680 G 59282 Blastochloris

0.00 1054748 1054748 S 2233851 Blastochloris tepida

0.00 1018551 1018551 S 1079 Blastochloris viridis

0.01 1604103 0 F 2843308 Pleomorphomonadaceae

0.01 1604103 0 G 1572860 Hartmannibacter

0.01 1604103 1604103 S 1482074 Hartmannibacter diazotrophicus

0.01 1574876 0 F 2685818 Amorphaceae

0.01 1574876 0 G 1904377 Acuticoccus

0.01 1574876 0 G1 2631034 unclassified Acuticoccus

0.01 1574876 1574876 S 2928472 Acuticoccus sp. I52.16.1

0.01 1542355 0 F 655351 Cohaesibacteraceae

0.01 1542355 0 G 655352 Cohaesibacter

0.01 1542355 0 G1 2631913 unclassified Cohaesibacter

0.01 1542355 1542355 S 1798205 Cohaesibacter sp. ES.047

0.01 1528418 0 F 2831111 Kaistiaceae
[truncated: 1,639,574 more chars]
