# Supplementary material for: Insights into gut microbiomes in stem cell transplantation by comprehensive shotgun long-read sequencing
Source: Sci Rep. 2024 Feb 19;14:4068. doi: 10.1038/s41598-024-53506-1 (PMC10876974; doi:10.1038/s41598-024-53506-1)
Supplement: Supplementary file 14 — Supplementary Information 2. [file 41598_2024_53506_MOESM14_ESM.docx]

# Supplementary Information

**Supplementary Figure 1. Composition estimates for the Zymo Gut Microbiome Standard.** Abundance estimation was carried out using Kraken2 and the MetaGut v. 1.0 database, for the minimap2 estimates we used either the fraction of reads or the fraction of bases while using only primary alignments to the Zymo provided reference sequences. The theoretical composition as reported by Zymo is plotted in the first column.

**Supplementary Figure 2. Composition Estimates for Lifelines Deep Samples.** Abundance estimation was carried out using Kraken2 with the MetaGut v. 1.0 database. The boxplot shows the fraction of species belonging to the *Bacteroides* and *Phocaeicola* genera for the Lifelines samples and for the control group samples of this study.

**Supplementary Figure 3. Validation rates stratified by sampling time point.** Groups (control, pre-Tx, leukopenia, reconstitution) are the same as in Figure 2. Non-fungal eukaryota excludes chordata and plants. For “binary” we considered a taxon to be validated when more than 20% of the associated reads could be validated. In this case we counted all reads as validated. “For continuous” we report the actual (weighted) validation rates.

**Supplementary Figure 4. Comparison of crAssphage abundance estimations.** Reads were mapped against crAssphage references using minimap2 retaining only primary alignments that aligned at least 70 % of the query with an identity of above 70 %. We used the fraction of primary alignments divided by the number of query reads as a rough estimate for the abundance. For comparison the default abundances provided by Kraken2 and the MetaGut v. 1.0 database are shown.

**Supplementary Figure 5. Distribution of reads across specific crAssphage references.** Barplots show the overall fraction of reads that are mapped to crAssphage. The heatmap is limited to the top 100 crAssphage sequences based on the mean of the assigned fractions and shows only ambiguous assignments (normalized to 100%). Sample-reference pairs with ambiguous assignments are colored gray. Since mapping quality directly corresponds to the uniqueness of each mapping, we used a threshold of mapping quality > 5 to determine reads that can be assigned to a specific reference.

**Supplementary Figure 6. Read migration from Kraken2 to minimap2.** Fraction of Kraken2 taxon assignments for reads that were assigned to crAssphage using minimap2 and a custom database of curated crAssphage references. For visual clarity, only the top 20 taxa are shown.

**Supplementary Figure 7. Marker taxa.** Contains abundance plots per patient for selected marker taxa using a binomial distribution to model confidence intervals based on alpha=0.05. Patients are shown with time (days) relative to alloHSCT on the x-axis and all control samples are collectively shown on the right side of the figure. Relative frequency and confidence intervals for α=0.05 of validated reads for the specified marker taxon are depicted on the y-axis. Each investigated taxon is shown on a separate page.

**Supplementary Figure 8. Share of Overlap Simulated Genomes ANI.** Shows the share of sequence overlap for pairs of simulated read sets for five bacterial taxa relative to different coverage values.

**Supplementary Figure 9. aANI-lowFreq** **distance quality relative to coverage.** The inner x-axis contains the FastANI genome distance and the inner y-axis the calculated aANI-lowFreq distance. Only distances with a shared overlap of the sequences above 70% are shown which removes all distances with simulated coverage 5 or 10.

**Supplementary Figure 10. Overview of high confidence strains level distances.** Distances for sequential samples within patients as histograms (column a), aANI-lowFreq distances for sequential samples within patients relative to the elapsed time (column b, in days), aANI-lowFreq distances between pre-Tx samples of different patients (column c). In addition we plotted the respective distances from the simulation of *Bacteroides vulgatus* and *Escherichia coli* references (see Methods) and marked the highest observed aANI-lowFreq distance (right border) of the two preceding histograms in the row. Significant strain replacement events are marked with a star.

**Supplementary Figure 11. Sampling Timepoints.** For visual clarity time is shown on a symmetrical logarithmic scale and some overlapping labels were omitted. The patients are arranged depending on the composition of their pre TX sample (Cluster 1: lavender, Cluster 2: sage, Cluster 3: peach). Clinical adverse events are marked in gray with symbols (see legend).

**Supplementary Figure 12. Illumina Nanopore Agreement.** X/Y Plots showing the abundance for each sample that was processed with both Nanopore and Illumina and yielded at least 10.000 reads for each technology. Each sample was scaled down to 10.000 reads by random sampling. Mapping based validation was not utilized here.

**Supplementary Table 1. Zymo Abundances.** Theoretical composition of the “Zymo Gut Microbiome Standard” as well as abundances based on Kraken2 mappings to our comprehensive database as well as minimap2 based mapping to the references provided by Zymo (using one estimate based on read counts and a second estimate based on nucleotide counts).

**Supplementary Table 2. Patient Statistics.** Metadata for each patient identifier. This contains primarily information about clinical outcomes and is used as an input file for the Jupyter Notebook based downstream analysis.

**Supplementary Table 3. Sample Statistics.** Metadata for each sample including sequencing statistics, top-level abundance estimates and cluster association. This is used as an input file for the Jupyter Notebook based downstream analysis and provided for reproducibility.

**Supplementary Table 4. Results Mann Whitney U.** Mann-Whitney U tests analyzing significant differentiation between pre-Tx clusters based on sample statistics

**Supplementary Table 5. Presence_Above_0.05_Bacteria.** Tables listing the presence of validated species detected at an abundance exceeding 5%, stratified by domain-like groups.

**Supplementary Table 6. Presence_Above_0.05_Fungi.** Tables listing the presence of validated species detected at an abundance exceeding 5%, stratified by domain-like groups.

**Supplementary Table 7. Presence_Above_0.05_Viruses.** Tables listing the presence of validated species detected at an abundance exceeding 5%, stratified by domain-like groups.

**Supplementary Table 8. Pre-therapies.** Information about treatments administered to the patient cohort pre-transplantation.

**Supplementary Table 9. Literature List Marker taxa.** List of literature references that report findings (regarding HSCT outcomes and IBD) correlated to specific taxonomic entities.

**Supplementary Table 10. Database Overview.** Overview of sequences and their length used for building the exhaustive metagenomics Kraken2 database, stratified by domain.

**Supplementary Table 11. Zymo Gut Microbiome Standard Validation.** Overview of calibration experiment for the validation method. Contains Kraken2 classifications as well as minimap2 classifications using the Zymo references and the MetaGut v. 1.0 database respectively.

**Supplementary Table 12. FastANI Assembly Top-Level.** Assembled contigs were compared to the Zymo References using FastANI. Each contig was assigned to the reference with the highest ANI. A weighted ANI was calculated based on the contig length for each reference.

**Supplementary Table 13. Benchmarking of the Kraken2 Filter.** Table showing evaluation of the validation method for experiments on Kraken2 databases with specific taxa removed.

**Supplementary Table 14. Taxonomic Profiles.** Table showing taxonomic profiles (abundance estimates) post validation for each individual sample. Different domains are listed separately on sheets.

**Supplementary Table 15. Mean Abundances.**  Shows the mean abundance of species aggregated by Timephase, Pre-Tx Cluster and a combination of both respectively (each presented on a different sheet).

**Supplementary Note 1. Cohort Description.** Additional information about the cohort makeup.

**Supplementary Note 2. In-Depth Analysis of Toxoplasma Content.** Additional method description and presentation of results for the analysis of Kraken2 based Toxoplasma classifications.

**Supplementary File 1. MetaGut v. 1.0 Database Contents.** All contained reference sequences are listed in the Kraken2 report format (Columns in order: Fraction of minimizers at subtree, number of minimizers at subtree, number of minimizers at node, taxonomic level, taxonomic id, taxonomic name).
